# Spatiotemporal Variation in Distance Dependent Animal Movement Contacts: One Size Doesn’t Fit All

**DOI:** 10.1371/journal.pone.0164008

**Published:** 2016-10-19

**Authors:** Peter Brommesson, Uno Wennergren, Tom Lindström

**Affiliations:** Department of Physics, Chemistry and Biology, Linköping University, Linköping, Sweden; The University of Melbourne, AUSTRALIA

## Abstract

The structure of contacts that mediate transmission has a pronounced effect on the outbreak dynamics of infectious disease and simulation models are powerful tools to inform policy decisions. Most simulation models of livestock disease spread rely to some degree on predictions of animal movement between holdings. Typically, movements are more common between nearby farms than between those located far away from each other. Here, we assessed spatiotemporal variation in such distance dependence of animal movement contacts from an epidemiological perspective. We evaluated and compared nine statistical models, applied to Swedish movement data from 2008. The models differed in at what level (if at all), they accounted for regional and/or seasonal heterogeneities in the distance dependence of the contacts. Using a kernel approach to describe how probability of contacts between farms changes with distance, we developed a hierarchical Bayesian framework and estimated parameters by using Markov Chain Monte Carlo techniques. We evaluated models by three different approaches of model selection. First, we used Deviance Information Criterion to evaluate their performance relative to each other. Secondly, we estimated the log predictive posterior distribution, this was also used to evaluate their relative performance. Thirdly, we performed posterior predictive checks by simulating movements with each of the parameterized models and evaluated their ability to recapture relevant summary statistics. Independent of selection criteria, we found that accounting for regional heterogeneity improved model accuracy. We also found that accounting for seasonal heterogeneity was beneficial, in terms of model accuracy, according to two of three methods used for model selection. Our results have important implications for livestock disease spread models where movement is an important risk factor for between farm transmission. We argue that modelers should refrain from using methods to simulate animal movements that assume the same pattern across all regions and seasons without explicitly testing for spatiotemporal variation.

## Introduction

Outbreaks of infectious livestock diseases can have a severe impact in terms of both economics and animal welfare. For instance, foot-and-mouth-disease (FMD), a viral disease that effect all animals with cloven hooves, can cause fevers, cause blisters in mouth and feet and sometimes lameness. The cost of the FMD outbreak in United Kingdom in 2001 has been estimated at more than £8 billion and more than four million animals were slaughtered [1]. Another major concern is classical swine fever (CSF), which causes high fever, huddling, anorexia and often mortality within weeks. The CSF outbreak in the Netherlands in 1997/1998, resulted in slaughter of at least 11 million pigs and total costs estimated at US $2.3 billion [2].

In order to reduce the impact of livestock diseases, mathematical models are powerful tools that may be used to inform policy. Modeling can be used to localize hotspots where the disease is more likely to spread [3], evaluate different control strategies [4] [5] and inform decisions that minimize the risk of an outbreak [6]. Analytical SIR models that assume a mass-action mixing process with equal probability for contact with other premises provide important theoretical insight, but have less implication in terms of addressing specific policy questions. Therefore, researchers are increasingly using stochastic simulation models that can account for important heterogeneity in the contact pattern [7]. Such heterogeneities can include assortative contact pattern depending on farm characteristics such as herd sizes and/or production types as well as spatial aspects that accounts for distance between premises [8–10]. In order to improve the reliability of epidemic models, it is essential that valid assumptions are made in regards to this contact structure. Predictions can vary with the underlying assumptions of the models and their parametrization [4, 11, 12] and erroneous assumptions can make models misguiding in terms of informing policy decisions.

Depending on the pathogen, livestock animal diseases can have several different transmission routes, including vectors, wildlife and direct contacts [13]. However, animal movement contacts are of particular importance for most infectious diseases [14, 15]. Consequently, it is essential that models make accurate assumptions about animal movement contacts when models are used for epidemiological prediction.

Contacts via livestock animal movements are generally distance dependent [16–21]; the longer the distance between two premises is, the lower the probability is of a movement to occur between them. Because of the high probability of between-herd infection via livestock movements, reliable estimation of this distance dependence is an integral part of most disease spread models. Some models, such as InterSpread Plus [22], do this by creating a look-up table based on empirical data. Other models implement kernel functions [23–25].

The importance of livestock movement for disease transmission varies between diseases. For epidemic outbreaks of transboundary diseases such as FMD or CSF, a nation wide movement ban would generally be instigated upon detection in previously disease free countries. As such, the primary effect of movement on disease transmission occurs during the silent spread phase. For endemic diseases however, animal movements can enhance the persistence of the pathogen by continuously spreading the disease between farms. Examples of endemic diseases where animal movement have been demonstrated as a risk factor include Bovine Viral Diarrhea [26, 27], Bovine Herpes [27], and Bovine Tuberculosis [15].

Previous studies have demonstrated seasonal and regional variation in the frequency of cattle movements [16, 28, 29]. While less studied, regional and temporal heterogeneity in terms of distance dependence of movements could also be expected. For instance, if the underlying mechanisms that determine the distance dependence in movement differ between regions, using a single kernel for the entire country may be inaccurate. Similarly, the distance dependence of movements during the summer may differ from that during the winter. If such heterogeneity exists, failing to recognize them in disease modeling will reduce the accuracy of predictions and potentially provide erroneous guidance for policy decisions.

The aim of this study was to provide insight into regional and/or seasonal variation in distance dependence of animal movement contacts and pinpoint whether they need to be accounted for in disease spread models. We addressed this by analyzing Swedish cattle movements and selected between nine models based on the granularity of the regional and temporal heterogeneities in distance dependence. Building on a kernel approach introduced by Lindström et al. [9, 19], parameters were fitted in a (Hierarchical) Bayesian framework using Markov Chain Monte Carlo (MCMC) methods and we used Deviance Information Criterion (DIC) [30] and log pointwise predictive density (lppd) evaluation [31] for model selection. To ensure that the model selection was robust, we also conducted a third test based on posterior prediction of relevant summary statistics.

## Materials and Methods

### Data

**Fig 1:**
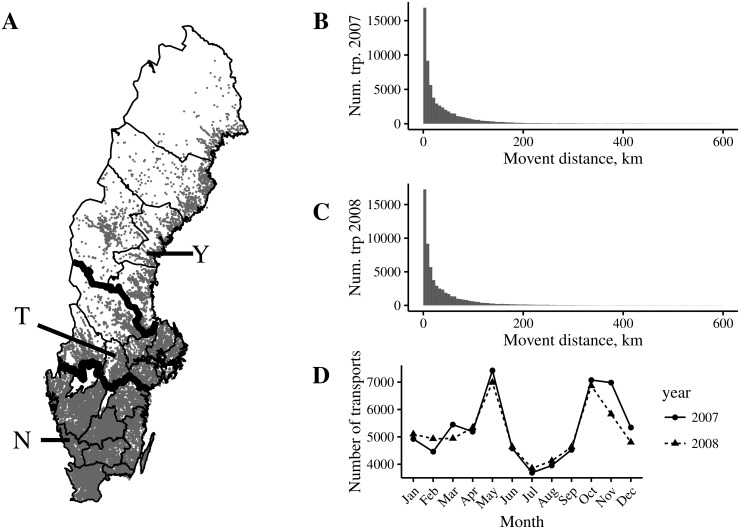
(A) Premises locations and county borders in Sweden. Thick borders indicate the borders separating the three lands of Sweden, from south to north: Götaland, Svealand and Norrland. Counties selected as examples in other figures are also pointed out (Y, T and N). (B) Frequency of movement distances in Sweden 2007. x-axis is truncated at 600 km and 527 movements of distances 600 km to 1265 km are not shown. (C) Frequency of movement distances in Sweden 2008. x-axis is truncated at 600 km and 432 movements of distances 600 km to 1293 km are not shown. (D) Number of transports per month in 2007 and 2008, respectively.

We used data of cattle transports between non-abattoir premises in Sweden (excluding the island Gotland) year 2007 and 2008, where the latter year’s data used for validation only. Here we considered premises, that were active during 2007 to 2008. An active premises, is here defined as in [28]. I.e. a premises is considered to be active if it had reported any movement of cattle to or from the premises (including movement to slaughter), or if it had reported births or deaths during the period. Premises where information of coordinates were missing, or where there was a mismatch between coordinates and the reported county, were also removed from data. In total, the data consisted of 24238 active premises. 63599 and 62029 livestock animal transports for year 2007 and 2008, respectively, were included. Fig 1 shows (A) premises locations and region borders, (B-C) frequency of movement distances and (D) the number of livestock animal movements per month. In Fig 1(B) and 1(C), x-axes were truncated at 600 km and 527 transports of distances 600 km to 1265 km and 432 transports of distances 600 km to 1293 km a, respectively, is not shown due to their, in comparison, low frequency.

The size of the premises was obtained by taking the most recent available data from observations made in July 2005, June 2006, July 2006 and December 2008. If no data were available, we used the median size (33 heads of cattle) of all premises in the data. This was the case for 3% of the premises data.

The data was originally provided by the Swedish Board of Agriculture and was used in edited form in [28].

### Bayesian models

We created nine candidate models based on the different combinations of level of granularity of the seasonal and regional heterogeneities in the relative shipment distances. That is, the models differ in at what level they are considering the spatial and temporal variability in the distance dependence.

We assumed the probability of shipments to decrease monotonically with distance between farms and modeled this with a power exponential distribution of the form,

$$g\left(D_{ij}|a,b\right)\propto e^{-{\left(\frac{D_{ij}}{a}\right)}^{b}}.$$ (1)

Here, *D*_*ij*_ is the Euclidean distance between farms *i* and *j*, and *a* and *b* are parameters determining the form of the kernel. However, it is not straight forward to interpret these parameters or to quantify differences between estimates. Instead we seek measures to quantify the scale and shape of the kernel.

In ecology, kernels are commonly quantified by moment statistics [32–34]. In particular, raw moments, which evaluate the kernel characteristics from the point of origin (here the shipping farm), are useful because of their relationship to invasion speed [35]. Simple diffusion tends to a Gaussian distribution, and the second raw moment *m*_*i*_ = **E**[*D*^*i*^] is proportional to the diffusion constant (*d* = 4*m*_2_ for two-dimensional kernels). The invasion speed will thus be proportional to *m*_2_, and this holds also for deviations from the Gaussian distribution, as long as the tails of the kernels are exponentially bounded [35]. It is further convenient to describe the kernel shape by its kurtosis, *κ*, measured as the fourth moment, normalized by the square of the second moment, $\kappa =m_{4}/m_{2}^{2}$. In general, kurtosis of a distribution provides a scale free measure of shape, mostly determined by the tail of the kernel [36]. These definitions and interpretations are straightforward in continuous space. Defining appropriate measures of kernel characteristics is more cumbersome for point pattern landscapes, which is commonly used for epidemiological models. That is, farm positions are represented as discrete locations with specified coordinates. When using a kernel approach to model transports between farms, the kernel describes the underlying process whereby the probability of a destination for a transport changes with distance. However, realized transport distances will be the result of both the underlying process, modeled by the kernel, and the farm locations, represented by coordinates. Simply put, a transport can only end up at a distance where there is a farm to receive it. The effect of kernel characteristics on the speed of biological invasions (including epidemics) in point pattern landscapes are less well studied. The effect of kernel characteristics on invasion speed depend on landscape characteristics [33]. Yet, we still rely on moment statistics to describe the kernel specified in Eq (1), but do so for communication purposes rather than theoretical expectations about the effect that they have on the invasion speed of an epidemic spreading through the contacts that transports mediate. By defining the scale of the kernel as $\sigma \equiv \sqrt{m_{2}}$, we obtain a measurement with a specified unit, here m. Thus, we can compare e.g. estimates for different seasons and if *σ*_1_ = *x* and *σ*_2_ = 2*x* we can say that the kernel with *σ*_2_ is stretched out by a factor of two compared to *σ*_1_. Further, we use *κ* as a measurement of kernel shape. If e.g. two kernels have parameters *σ*_1_ = *σ*_2_ and *κ*_1_ > *κ*_2_, the kernel with *κ*_1_ will have higher probability of short distance movements and at the same time a fat tail of the kernel describing higher probability of producing long distance movement. This is similar to what have been used in [32]. It should however be stressed again that realized movements will also depend on the position of farms, and as such the kernel *σ* and *κ* should be interpreted as measurements that quantify the underlying distance dependence in contact probabilities.

**Fig 2:**
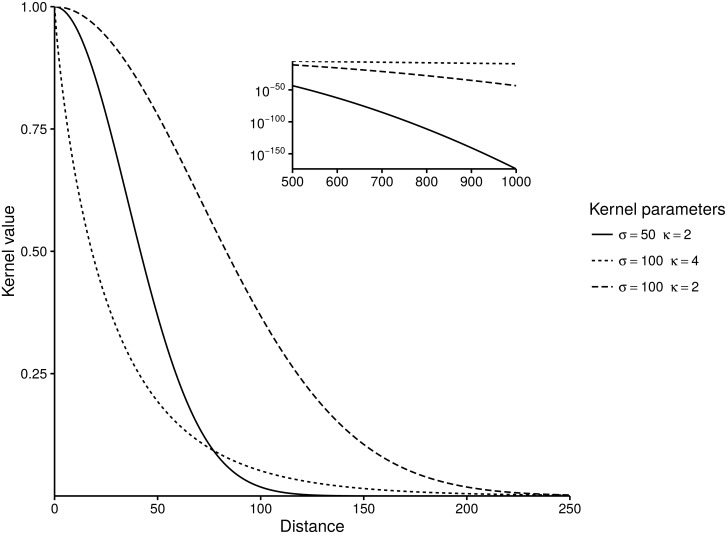
Kernel shape for three parameter sets, inset figure shows kernel values (on the log scale) for larger distances.

For kernels of the form shown in Eq (1), *σ* and *κ* are calculated as

$$\sigma =\sqrt{m_{2}}=a\sqrt{\frac{\Gamma \left(\frac{4}{b}\right)}{\Gamma \left(\frac{2}{b}\right)}}\text{ and }\kappa =\frac{m_{4}}{m_{2}^{2}}=\frac{\Gamma \left(\frac{6}{b}\right)\Gamma \left(\frac{2}{b}\right)}{\Gamma {\left(\frac{4}{b}\right)}^{2}},$$ (2)

where Γ indicates the gamma function. Fig 2 shows examples of the kernel for different *σ* and *κ*.

We estimated parameters in a Bayesian framework, thus obtaining probability distributions for quantities of interest rather than point estimates. This is beneficial when models are used for predicted purposes because parameter uncertainty can be included in the predictions.

The models will be denoted M_*ρω*_ where subscripts *ρ* and *ω* indicates the level of regional and seasonal granularity respectively. Subscripts s, l, c, y, q and m, correspond to Sweden, lands, county, year, quarter and month, respectively. Here, lands denotes three collection of counties in Sweden (see Fig 1). We here start by outlining the likelihood functions for each of the models and then present each of the (hierarchical) Bayesian models below.

#### Seasonally and regionally invariant model—M_sy_

For model M_sy_, we assumed that no seasonal or regional variation exist in the contact pattern. We let **T** denote the set of all observed transports, and let **N** denote the set of active premises. The probability of a transport *t* ∈ **T** of distance $D_{o_{t},d_{t}}$, originating at premises *o*_*t*_ ∈ **N** and destinating at premises *d*_*t*_ ∈ **N**, *d*_*t*_ ≠ *o*_*t*_ is

$$f_{t}\left(D_{o_{t},d_{t}}|a,b\right)=\frac{e^{-{\left(\frac{D_{o_{t},d_{t}}}{a}\right)}^{b}}}{\sum_{\begin{array}{l} j\in N\\ j\neq o_{t} \end{array}}e^{{\left(\frac{D_{o_{t},j}}{a}\right)}^{b}}}.$$ (3)

We acknowledge that distance is not the only factor of determining the movement probabilities. Specifically, it is likely that premises with a large number of animals are more likely to receive animals. Thus, a function of the size of the premises was used as a weight when calculating the probability of a transport ending at a particular farm. The size component was introduced to assert that regional differences were not an artefact of the sizes of the premises. A weight function $W\;\left(w_{d_{t}}\right)$, where $w_{d_{t}}$ is the number of cattle on the destination premises for transport *t*, was introduced and four weight functions were used. We evaluated our models using $W\left(w_{e_{t}}\right)=w_{e_{t}}^{\xi }$, for *ξ* ∈ {0, 0.5, 1, 1.5}, where 0 indicate no effect of farm size, 0.5 indicate sublinear effect, 1 indicate linear effect and 1.5 indicate superlinear effect. Using the notation **Θ** = (*σ*, *κ*), the likelihood for model M_sy_ is defined as

$$L_{1}\left(\Theta |D_{t\in T}\right)=\underset{t\in T}{\prod }\frac{W\left(w_{d_{t}}\right)e^{-{\left(\frac{D_{o_{t},d_{t}}}{a}\right)}^{b}}}{\sum_{\begin{array}{l} j\in N\\ j\neq o_{t} \end{array}}W\left(w_{j}\right)e^{-{\left(\frac{D_{o_{t},j}}{a}\right)}^{b}}}$$ (4)

#### Seasonal variation models—M_sq_ and M_sm_

In the second model, we allowed the movement patterns to vary seasonally. This was accounted for by introducing season specific **Θ**_*ω*_ = (*σ*_*ω*_, *κ*_*ω*_) for season *ω* ∈ **Ω**, where the set of seasons was denoted **Ω**. As such, this model (and the ones below) can be described as random effects models. For models M_sq_ and M_sm_, the set **Ω** was equal to set of quarters and months, respectively. Further, the set of transports occurring in season *ω*, was denoted **T**_*ω*_. Hence, the probability of all movements in season *ω* is written as

$$l\left(\Theta_{\omega }|D_{t\in T_{\omega }}\right)=\underset{t\in T_{\omega }}{\prod }\frac{W\left(w_{d_{t}}\right)e^{-{\left(\frac{D_{o_{t},d_{t}}}{a_{\omega }}\right)}^{b_{\omega }}}}{\sum_{\begin{array}{l} j\in N\\ j\neq s_{t} \end{array}}W\left(w_{j}\right)e^{-{\left(\frac{D_{o_{t},j}}{a_{\omega }}\right)}^{b_{\omega }}}}$$ (5)

and the likelihood of all movements as

$$L_{2}\left(\Theta_{\Omega }|D_{t\in T}\right)=\prod_{\omega \in \Omega }l\left(\Theta_{\omega }|D_{t\in T_{\omega }}\right).$$ (6)

#### Regional variation models—M_ly_ and M_cy_

Analogously, in the model where regional differences were considered, every region was modeled as having its unique set of kernel parameters **Θ**_*ρ*_ = (*σ*_*ρ*_, *κ*_*ρ*_) for all regions *ρ* ∈ **P**, where **P** indicates the set of all considered regions. The regions in models M_ly_ and M_cy_, are three collections of counties (the lands of Sweden) and the counties of Sweden, respectively. The set of transports originating from region *ρ* was denoted **T**_*ρ*_. Hence, probability of movements from a specific county is written as

$$l\left(\Theta_{\rho }|D_{t\in T_{\rho }}\right)=\underset{t\in T_{\rho }}{\prod }\frac{W\left(w_{d_{t}}\right)e^{-{\left(\frac{D_{o_{t},d_{t}}}{a_{\rho }}\right)}^{b_{\rho }}}}{\sum_{\begin{array}{l} j\in N\\ j\neq o_{t} \end{array}}W\left(w_{j}\right)e^{-{\left(\frac{D_{o_{t},j}}{a_{\rho }}\right)}^{b_{\rho }}}}$$ (7)

and the likelihood of all transports as

$$L_{3}\left(\Theta_{P}|D_{t\in T}\right)=\prod_{\rho \in P}l\left(\Theta_{\rho }|D_{t\in T_{\rho }}\right).$$ (8)

#### Regional and seasonal variation models—M_lq_, M_lm_, M_cq_ and M_cm_

To capture the assumed seasonal and regional heterogeneities, we modeled the displacement kernels with to have separate parameters for both different regions as well as different seasons. In this model, the notation **Θ**_*ρω*_ = (*σ*_*ρω*_, *κ*_*ρω*_) is used for the sought parameters and the set of observed transports, originating in region *ρ* season *ω*, is denoted **T**_*ρω*_. Thus, the probability of movements for a particular combination of region and season can be written as

$$l\left(\Theta_{\rho \omega }|D_{t\in T_{\rho \omega }}\right)=\underset{t\in T_{\rho \omega }}{\prod }\frac{W\left(w_{d_{t}}\right)e^{-{\left(\frac{D_{o_{t},d_{t}}}{a_{\rho \omega }}\right)}^{b_{\rho \omega }}}}{\sum_{\begin{array}{l} j\in N\\ j\neq o_{t} \end{array}}W\left(w_{j}\right)e^{-{\left(\frac{D_{o_{t},j}}{a_{\rho \omega }}\right)}^{b_{\rho \omega }}}}$$ (9)

and consequently the likelihood is defined as

$$L_{4}\left(\Theta_{P\Omega }|D_{t\in T}\right)=\prod_{\rho \in P}\prod_{\omega \in \Omega }l\left(\Theta_{\rho \omega }|D_{t\in T_{\rho \omega }}\right).$$ (10)

### Bayesian modeling and prior elicitation

#### Model M_sy_

To express the probability density function of **Θ** (the model where no heterogeneity in space or time are considered), the prior probability for **Θ** was chosen as *Ψ*(**Θ**) = *Ψ*_*σ*_ (*σ*|*α*_*σ*_, *β*_*σ*_) *Ψ*_*κ*−4/3_ (*κ*|*α*_*κ*_, *β*_*κ*_). With *Ψ*_*σ*_ and *Ψ*_*κ*−4/3_ as prior distributions for *σ* and *κ* respectively. The distribution *Ψ*_*σ*_ was specified as a gamma distribution with shape and scale parameters *α*_*σ*_ and *β*_*σ*_. Since the lower limit of *κ* is 4/3, *Ψ*_*κ*−4/3_ was chosen to be a shifted gamma distribution with shape and scale parameters *α*_*κ*_ and *β*_*κ*_. That is,

$$\begin{array}{cc} \Psi_{\sigma }\left(\sigma |\alpha_{\sigma },\beta_{\sigma }\right) & =\frac{\beta_{\sigma }^{-\alpha_{\sigma }}}{\Gamma \left(\alpha_{\sigma }\right)}\sigma^{\alpha_{\sigma }-1}e^{-\frac{\sigma }{\beta_{\sigma }}}\;\text{and}\\ \Psi_{\kappa -4/3}\left(\kappa |\alpha_{\kappa },\beta_{\kappa }\right) & =\frac{\beta_{\kappa }^{-\alpha_{\kappa }}}{\Gamma (\alpha_{\kappa })}{\left(\kappa -4/3\right)}^{\alpha_{\kappa }-1}e^{-\frac{\kappa -4/3}{\beta_{\sigma }}}. \end{array}$$ (11)

To keep the prior distributions vague, we chose the parameters as (*α*_*σ*_, *β*_*σ*_) = (0.001, 1000) and (*α*_*κ*_, *β*_*κ*_) = (0.001, 1000) (*σ* ∼ *Gamma*(0.001, 1000) and *κ* − 4/3 ∼ *Gamma*(0.001, 1000)). Thus, the joint posterior probability distribution was written as

$$\begin{array}{cc} & p\left(\Theta |D_{t\in T}\right)\propto L_{1}\left(\Theta |D_{t\in T}\right)\Psi \left(\Theta \right)=\\ & \prod_{t\in T}\frac{W\left(w_{d_{t}}\right)e^{-{\left(\frac{D_{o_{t},d_{t}}}{a}\right)}^{b}}}{\sum_{\begin{array}{l} j\in N\\ j\neq o_{t} \end{array}}W\left(w_{j}\right)e^{-{\left(\frac{D_{o_{t},j}}{a}\right)}^{b}}}\Psi_{\sigma }\left(\sigma |\alpha_{\sigma },\beta_{\sigma }\right)\Psi_{\kappa -4/3}\left(\kappa |\alpha_{\kappa },\beta_{\kappa }\right). \end{array}$$ (12)

#### Models M_sq_, M_sm_ and M_ly_, M_cy_

In the models with season and region specific parameters, respectively, the joint posterior distribution was modeled as a product of the probability of movements for each season and region, respectively. Further, since the transport data were divided into different subsets with less amount of data in each set, hierarchical priors were implemented. Thus, the parameters *σ*_*ω*_, *κ*_*ω*_, *σ*_*ρ*_ and *κ*_*ρ*_, respectively, were viewed as samples of a common population distribution (details can be found in [37]). That is, the prior parameters *α*_*σ*_, *β*_*σ*_ *α*_*κ*_ and *β*_*κ*_, were modeled to come from hyper prior distributions *p*(*α*_*σ*_), *p*(*β*_*σ*_), *p*(*α*_*κ*_) and *p*(*βκ*), respectively. The posterior distribution for models M_qs_ and M_ms_ (seasonal heterogeneity assumed) is written as

$$\begin{array}{cc} & p(\Theta_{1},\ldots ,\Theta_{\left|\Omega \right|},\alpha_{\sigma },\beta_{\sigma },\alpha_{\kappa },\beta_{\kappa }|D_{t\in T})\propto \\ & \prod_{\omega \in \Omega }l\left(\Theta_{\omega }|D_{t\in T_{\omega }}\right)\Psi_{\sigma }\left(\sigma_{\omega }|\alpha_{\sigma },\beta_{\sigma }\right)\Psi_{\kappa -4/3}\left(\kappa_{\omega }|\alpha_{\kappa },\beta_{\kappa }\right)p\left(\alpha_{\sigma }\right)p\left(\beta_{\sigma }\right)p\left(\alpha_{\kappa }\right)p\left(\beta_{\kappa }\right). \end{array}$$ (13)

Analogously, the joint probability distribution for models M_yl_ and M_yc_ (regional heterogeneity assumed) is written as

$$\begin{array}{cc} & p(\Theta_{1},\ldots ,\Theta_{\left|P\right|},\alpha_{\sigma },\beta_{\sigma },\alpha_{\kappa },\beta_{\kappa }|D_{t\in T})\propto \\ & \prod_{\rho \in P}l\left(\Theta_{\rho }|D_{t\in T_{\rho }}\right)\Psi_{\sigma }\left(\sigma_{\rho }|\alpha_{\sigma },\beta_{\sigma }\right)\Psi_{\kappa -4/3}\left(\kappa_{\rho }|\alpha_{\kappa },\beta_{\kappa }\right)p\left(\alpha_{\sigma }\right)p\left(\beta_{\sigma }\right)p\left(\alpha_{\kappa }\right)p\left(\beta_{\kappa }\right), \end{array}$$ (14)

i.e. similar as Eq 13, but data separated by region rather than season.

#### Models M_lq_, M_lm_, M_cq_ and M_cm_

A similar approach was used when both seasonal and regional variability was taken into account. As with models M_qs_, M_ms_ and M_yl_, M_yc_, a hierarchical structure was implemented, but the prior was here defined for all parameters specific for each combination of region and season. Thus, the joint posterior probabilities were written as

$$\begin{array}{cc} & p(\Theta_{1,1},\ldots ,\Theta_{\left|P||\Omega \right|},\alpha_{\sigma },\beta_{\sigma },\alpha_{\kappa },\beta_{\kappa }|D_{t\in T})\propto \\ & \prod_{\rho \in P}\prod_{\omega \in \Omega }l\left(\Theta_{\rho \omega }|D_{t\in T_{\rho \omega }}\right)\Psi_{\sigma }\left(\sigma_{\rho \omega }|\alpha_{\sigma },\beta_{\sigma }\right)\Psi_{\kappa -4/3}\left(\kappa_{\rho \omega }|\alpha_{\kappa },\beta_{\kappa }\right)p\left(\alpha_{\sigma }\right)p\left(\beta_{\sigma }\right)p\left(\alpha_{\kappa }\right)p\left(\beta_{\kappa }\right). \end{array}$$ (15)

When using hierarchical priors, the priors for *σ*_*ρ*_, *σ*_*ω*_, *σ*_*ρω*_ and *κ*_*ρ*_, *κ*_*ω*_, *κ*_*ρω*_ were chosen as gamma and shifted gamma distributions, respectively (i.e. of the same form as in the case with no regional or seasonal heterogeneity). However, instead of fixed shape and scale parameters for the prior distributions, the parameters were modeled to come from gamma distributions with parameters, shape = 0.001 and scale = 1000. That is, *α*_*σ*_, *β*_*σ*_, *α*_*κ*_, *β*_*κ*_ ∼ *Gamma*(0.001, 1000). The motivation for the choice of parameter values of the hyper priors were to form vague, but proper hyper prior densities.

### Computation

None of the full Bayesian models (Eqs 12, 13, 14, 15) has a known form, and we computed the posterior distribution of the parameters of interest with Markov Chain Monte Carlo (MCMC) methods. To achieve this, we used Metropolis-Hastings algorithms [38] with component wise random walk updates [39] with a bivariate Gaussian proposal on the log scale of the **Θ** parameters in all four models. For the prior parameters (*α*_*σ*_, *β*_*σ*_) and (*α*_*κ*_, *β*_*κ*_) in the models with seasonal and/or regional heterogeneities, we used bivariate Gaussian proposals on the linear scale. Component wise random walk updates were used for these parameters as well. We computed 1,000,000 iterations and discarded the first 200,000 iterations as burn in. The figure in supplementary material S3 Fig. shows Markov chains for the hyper parameters (shape and scale) for *σ*_*ρ*_ and *κ*_*ρ*_ in model M_cy_.

To facilitate good mixing, we used an adaptive proposal algorithm described by Garthwaite et al. [40] to obtain a long term acceptance rate of 0.234 [41]. This algorithm has been shown to be efficient for high dimensional models [17].

To speed up calculations, we did not use exact distances but approximations. We approximated distances by splitting the distance between 0 km to the maximum possible distance (1484 km) into 50 intervals of the same length. Further, since higher precision is more important at short distances, we split the first interval into 50 sub intervals. The second interval in to 49 sub intervals and so on until we came to the point where we split one interval in halves. The remaining intervals were also split in halves, thus we have sub intervals describing unique range of distances. A distance of length within the range of a sub interval, were approximated the length equal to the mid point of the sub interval. The maximum deviation from the true distance ranged from approximately 300 m at shortest distance to 10 km at longest distance.

The models were implemented in C++ and parallelized using OpenMP 4.0. Calculations were made at the Triolith and Gamma clusters at Swedish National Infrastructure for Computing (SNIC) at Linköping University [42]. Computation times varied from approximately 1.5h for the simplest model, to approximately 10h for the most complex model on a computation node with 16 cores.

### Model selection

Model selection was performed by three different methods. First, we calculated the Deviance Information Criterion (DIC) for each of the nine candidate models. DIC was chosen in favor over other criterion such as Akaike Information Criterion (AIC) and Bayesian Information Criterion (BIC), due to its advantages when considering hierarchical Bayesian models [30]. Both AIC and BIC requires a specified number of parameters, which isn’t predefined for hierarchical models. DIC instead estimates the effective number of parameters. The interpretation is however similar to that of AIC or BIC in that the model with the lowest DIC score is considered to best fit the data.

Secondly, we estimated the log predictive posterior density [31] for 2008 year’s data (lppd_2008_) for the models. As in [31], we sought

$$\text{lppd}=\sum_{i=1}^{n}\text{log}\int p\left(y_{i}|\Theta \right)p_{post}\left(\Theta \right)d\Theta ,$$ (16)

where *y*_1_, …, *y*_*n*_ are data points and *p*_*post*_(**Θ**) is the posterior distribution. We estimated lppd_2008_ by calculating the computed lppd (clppd) [31], as

$${\text{llpd}}_{2008}=\text{clppd}=\sum_{i=1}^{n}\text{log}\left(\frac{1}{S}\sum_{s=1}^{S}p\left(y_{i}|\Theta_{s}\right)\right).$$ (17)

Here, the data points *y*_*i*_ are the observed movements in 2008 and **Θ**_*s*_ are simulation draws from the posterior distribution. Here, we used *S* = 10000. This provides us with a relative measure of the predictive ability. The interpretation is, the higher value, the better prediction.

Thirdly, we performed an analysis based on the ability of models to reproduce relevant summary statistics. Focusing on county and month specific movements, i.e. the highest spatiotemporal resolution considered in the analysis, we investigated if models were able to accurately predict the median (*p*_50_) and upper 95 percentile (*p*_95_) of 2008 year’s observed movement distances. For this purpose, we generated 1000 movement networks per model, each parameterized by a random draw from the posterior distribution of parameters as represented by the MCMC analysis. The generated movements were chosen to have the same origin premises as the 2008 year’s movement data. As such, we do not make any assumptions about the origin farms, modeling only the focal process of this study: the prediction of movement distances. Considering the 240 combinations of county and month, we scored the models by the percentages of observed values of *p*_50_ and *p*_95_ encapsulated by the range of the 95% central credibility interval of posterior predictive distributions.

## Results

Our analysis selected models with spatial granularity at county level, as the three most preferred models, with support of both DIC, lppd and posterior predictive accuracy. At temporal level, the results are more ambiguous. Different selection criteria, support different levels of seasonal granularity. Table 1 shows DIC and lppd_2008_ estimates as well as percentages of counties and month where summary statistics *p*_50_ and *p*_95_ were encapsulated by the 95% central credibility interval of corresponding posterior predictive distributions. The linear correction for farm sizes, i.e. $W\left(w_{d_{t}}\right)=w_{d_{t}}^{1.0}$, consistently outperformed other corrections with minimum DIC difference of 2786, compared to the corresponding model with alternative functions for $W\;\left(w_{d_{t}}\right)$. This is interpreted as inconsequential support for alternative corrections and we therefore present results for models with $W\left(w_{d_{t}}\right)=w_{d_{t}}^{1.0}$.

**Table 1:** Table of DIC scores, lppd values and the proportion of 95*^th^* percentile and 50*^th^* percentile, encapsulated by the corresponding posterior predictive distributions 95% central credibility intervals.

Model	DIC	lppd_2008_	p_95_ acc.	p_50_ acc.
M_sy_	698,045	-419,001	42.1%	48.8%
M_sq_	698,059	-418,976	38.3%	47.1%
M_sm_	697,785	-418,935	38.3%	47.5%
M_ly_	688,692	-418,138	58.3%	46.3%
M_cy_	680,905	-417,299	65.0%	66.7%
M_lq_	688,517	-418,152	55.0%	47.1%
M_lm_	688,580	-418,158	55.0%	45.8%
M_cq_	680,458	-417,399	64.6%	64.6%
M_cm_	680,881	-417,627	65.0%	75.0%

**Fig 3:**
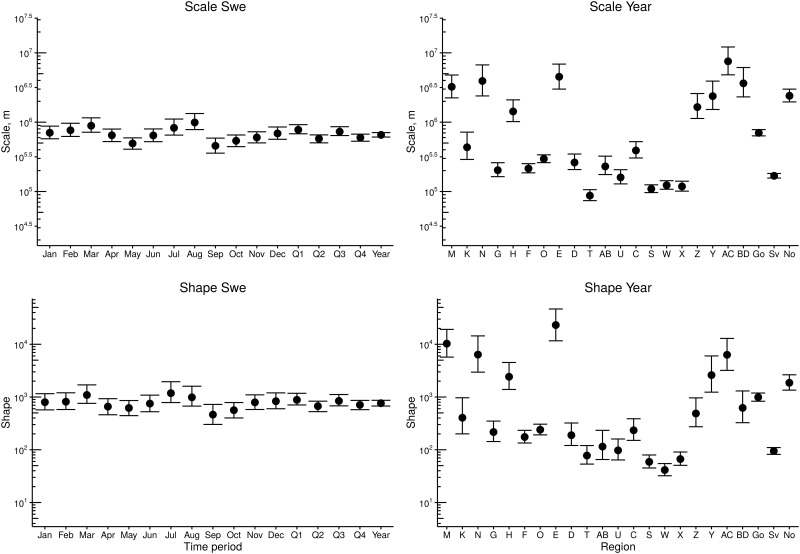
95% credibility intervals of shape (upper row) and scale (lower row), for M_*sq*_ and M_*sm*_ (left panel), accounting for seasonal variability in movements; and M_*ly*_ and M_*cy*_ (right panel), accounting for regional variability. Corresponding parameter values of M_*sy*_ are shown to the far right in the left panel.

Fig 3 shows the seasonal and regional variations in the parameter estimates from models with seasonal variability (models M_sq_ and M_sm_) and regional variability (models M_ly_ and M_cy_), respectively, indicated by median and 95% credibility intervals (CI). For comparison, parameter estimates for model M_sy_, i.e. where neither regional nor seasonal differentiation is considered, are shown on the far right of the left panels. The figure illustrates considerable spatial variation with non-overlapping CIs for estimates specific to each region, thereby giving further support to the notion that regional differentiation should be considered. Non-overlapping CIs at the temporal scale exists, but not as prominent as on the regional scale.

**Fig 4:**
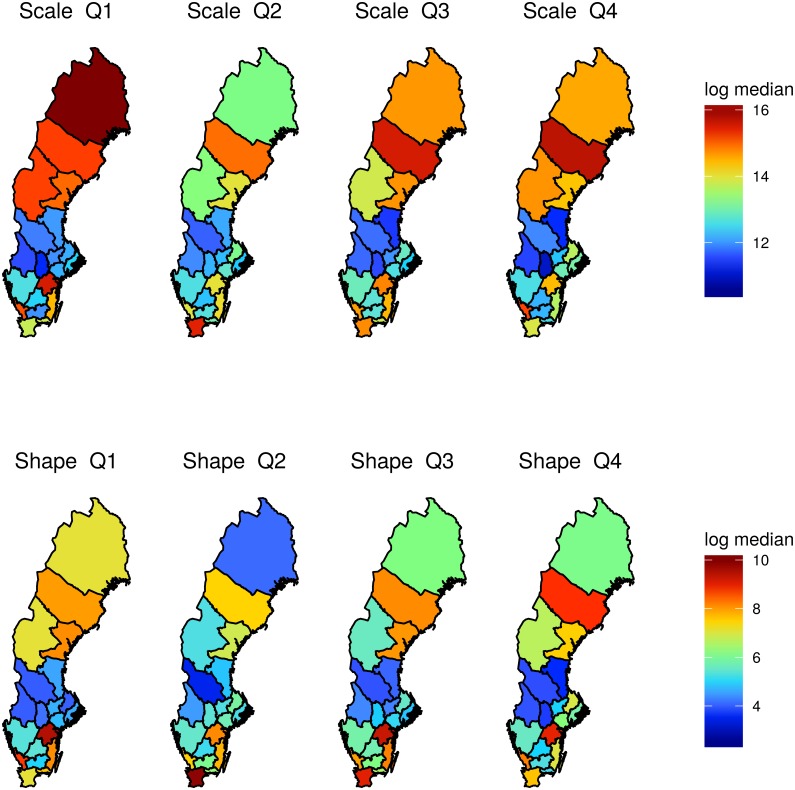
Median shape and scale estimates for counties in Sweden in quarter Q1 to Q4, presented on the log scale.

Fig 4 further illustrates regional variations for quarter Q1 to Q4 in Model M_*cq*_. Different colors correspond to different median values of scale and shape on the log scale.

**Fig 5:**
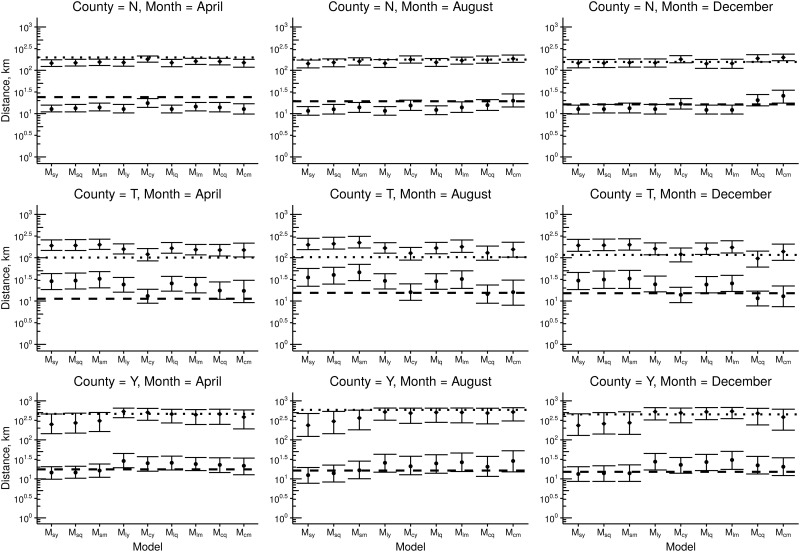
Medians and corresponding 95% credibility intervals of the posterior predictive distribution of the 50th (dots) and the 95th (diamonds) percentiles of movement distances for selected months and counties, based on parameters from 2007. Dashed and dotted lines indicate the corresponding quantities in the observed data from 2008.

Fig 5 shows the predicted summary statistics for the nine models, for three different counties in April, August and December, illustrating the method of calculating accuracy of predictions for *p*_50_ and *p*_95_. The error bars indicate the interval between the 2.5^*th*^ and 97.5^*th*^ percentiles of the posterior predictive distributions and the medians corresponding to the *p*_50_ and *p*_95_ estimates are indicated as dots and diamonds, respectively. The observed quantities for *p*_50_ and *p*_95_ year 2008 are indicated with dashed and dotted lines, respectively. For example, Fig 5 shows that for county T and December, *p*_50_ and *p*_95_ were encapsulated by CIs generated with M_cy_, M_cq_ and M_cm_, whereas using any of the other models did not provide CIs that encapsulate the observed values. As another example, for county Y and December, all models succeeded in encapsulating the *p*_95_ and only M_ly_, M_lq_ and M_lm_ failed in encapsulating *p*_50_. The counties were chosen based on location to represent the southern (N), middle (T), and northern (Y) parts of Sweden. These counties are annotated in Fig 1.

## Discussion

Animal movement is of particular importance for livestock disease spread between farms because of high transmission risk [14] and the ability to carry diseases over long distances [25, 43]. It is therefore of great importance to sufficiently capture the contact pattern in disease simulation models that are used to inform policy when livestock movement is an important pathway for between farm transmission.

The aim of this study was to demonstrate the importance of regional and seasonal differences in the distance dependent component of the contact pattern. For this purpose, we compared nine predictive models for cattle movements, fitted to data of all reported shipments of one year in Sweden. Our results clearly showed that spatial factors are important when modeling distance dependence in cattle movements, whereas temporal factors may be less important.

Table 1 shows that based on DIC score, M_cm_, which accounts for both regional and temporal variability, is the preferred model. The DIC score indicates that regional variability at the finest granularity, i.e. county, is preferred, and that temporal variability should be accounted for at medium granularity (quarter). The DIC difference between M_cq_ and the nearest model is 423, indicating inconsequential support for alternative models.

Though the DIC scores clearly shows that M_cq_ fit the data best, it does not provide easily interpreted information on the difference in predictive abilities among the candidate models. We therefore performed two additional model selection analyses, using parameter estimates from 2007 year’s data, and 2008 year’s data for validation. First, we estimated lppd:s for the models (Table 1). The llpd_2008_ values indicate that M_cy_ is the preferred model which is the model where we accounted for regional differences at county level, but where we did not assume temporal variability.

Secondly, comparing observed summary statistics of movement distances, to the corresponding posterior predictive distributions of these statistics. We focused on the medians and upper 95% percentiles movement distances for each month and county and compared the models by their ability to predict the observed 2008 year’s data from estimates based on data from 2007. Table 1 shows that model selection based on these summary statistics would lead to M_cm_ as most preferred model. That is, when we account for the highest of the considered levels of granularity in spatial and temporal variability.

Figs 3 and 4 show that although both seasonal and regional variations in parameter estimates exist, the variability is more evident when considering regional location. This is in agreement with the model selection presented in Table 1. The considered methods for model selection clearly prefers models with spatial variability at county level, but the preferred level of seasonal variability is harder to determine. The three methods for model selection all agree that regardless of the considered temporal granularity, the preferred model is the model accounting for spatial variability at county level, and the least preferred model does not account for spatial heterogeneities. Further, the preferred model based on llpd_2008_ value, does not consider temporal heterogeneities whereas preferred models evaluated by DIC and summary statistics for *p*_50_ and *p*_95_ do, but at different granularity.

Animals are shipped between holdings for several purposes, including e.g. grazing, breeding, fattening before slaughter, and farmers increasing or decreasing herd sizes. The underlying factors that determine animal movement distances can be expected to vary with the reason for shipment. For instance, the economic and social factors that regulate the shipment of a single breeding animal are different from those that determine shipment of dairy herd bulls for the purpose of fattening before slaughter. The spatial, and to some extent seasonal, variability in shipment distances we reveal here is therefore likely a result of regional and seasonal differences in cattle production. Cattle farming in Sweden is highly seasonal (Fig 1) [16], with calves generally born in spring. Animals are often kept indoors during the winter and are shipped for grazing around April or May. The underlying purpose of shipments varies over the year, and as such, the contribution of the different transports to the data used for kernel fitting will vary seasonally. Though seasonal heterogeneity in distance dependence is observed, it is not clear if, or to what extent, it should be accounted for. When using DIC based on the 2007 data, i.e. the data used for parameter estimation, the intermediate seasonal granularity was selected. Thus, we can say that for the analyzed data, there was some seasonal variation. However, the DIC scores are relatively similar, and there appears to be no benefit for predictive purposes. When using the models for prediction of the subsequent year’s transports, the lppd_2008_ scores selects the model with no temporal variability as the preferred model. These results suggest less importance of seasonal variation in distance dependence, and is somewhat surprising, given the seasonality in farming practices in Sweden.

Our method focuses on the relative distance dependence by normalizing probabilities over all possible destination farms. Eq 3 describes how the relative probability of destination farm changes with distance. Thus, the regional differences we observe in shape and scale (Fig 3) are not merely the result of differences in farm densities and average distances between farms. Instead, we propose that regional differences in production systems could explain the observed spatial variability. For pigs, it has been demonstrated that the distance dependence in contact probability varies among production types [9]. No available data exist on production types for cattle in Sweden, preventing us from explicitly analyzing how production types affect shipment distances. Farming is however generally more intensive in the south compared to the north [16], and we expect that the observed spatial component is largely an effect of differences in farming practices. This hypothesis can however not be tested in the absence of more detailed data. We argue that lack of detailed data is not a unique situation for Sweden and that this framework can be suitable when analyzing distance dependencies in other countries (e.g. France, Belgium and United Kingdom) where regional heterogeneity in farming practices exist [44].

The lack of detailed data on farming practices at herd level and the underlying reasons for an individual shipment, can with this framework to some extent be circumvented. We here show that by accounting for regional, and to some extent seasonal differences, the ability to predict shipment distances is improved considerably.

The aim of this study was to investigate the presence of spatiotemporal variability in movement distances, using Swedish shipment data as a case study. For this purpose, we used a low dimensional model and showed that when regional variability is accounted for, the predictions can be improved considerably (Fig 3 and Table 1). The importance of accounting for temporal variability is more ambiguous, and differs with criteria for model selection. In order to further improve prediction of between farm movement, additional covariates may be necessary. We here adjusted contact probabilities by herd size, but acknowledge that additional factors are important. The framework is however flexible, and can be expanded to account for additional factors when reliable data is available.

Our study provides important insight for modelers of livestock diseases. Prediction of animal movement is an integral part of most stochastic disease spread models, and various approaches have been implemented, including resampling of observed shipments [45], creating look-up tables [22] or parametric estimation of contact probabilities [17, 25]. Our results show the importance of accounting for regional, and to lesser extent also seasonal variations when doing so. Previous studies have primarily looked at spatiotemporal dynamics of shipment frequency [15, 16, 28, 29], but we here demonstrate that the distance component also vary with region, and to a lesser extent with season.

The implications of these findings for disease modeling varies with the disease as well as with the question that models are used to answer. For outbreaks of FMD or CSF, a nationwide movement ban would be constituted upon detection. As such, epidemic models primarily need to account for movement in the silent spread phase. For instance, during the 2001 outbreak of FMD in the UK, most transmission occurred post movement ban, and thus other factors were more important [46]. This indicates that factors other than animal movement are more important to account for when modeling. However, a major reason for the large and prolonged outbreak was that the disease was already widespread upon detection, making it difficult to contain, and much of this spread occurred though animal movements [1]. Accounting for spatiotemporal variability in animal movement contacts can therefore be important also for modeling of outbreaks of such transboundary diseases, particularly for issues related to detection time. For endemic diseases or outbreaks of diseases without movement bans, livestock movement can continue to spread pathogens between farms. Models are commonly used to rank different control options [4, 12, 47], incorporating realistic movement pattern in models will be important when these models are used to compare control actions for such diseases. For instance, if spatial heterogeneity in the contact pattern isn’t accounted for, models may provide erroneous policy recommendations about which farms to target for control and/or surveillance.

Movement is typically one of several pathways that can transmit the disease between farms. Other transmission processes typically also show spatiotemporal variability, and heterogeneity in the movement contact pattern can interact with other processes. Here, we particularly identified the importance of spatial variability in animal movements with the temporal component of lesser or no importance, depending on the selection criteria. The same pattern might not be present in other countries, and we propose that seasonal and regional variability should be tested for before assuming a homogeneous contact structure for modeling purposes.

We argue that the kernel approach is ideally suited for this, and has some important benefits over direct use of movement data for disease modeling. Some models incorporate animal movements by resampling observed movements [45], but when considering finer spatial or temporal resolution, the number of movements that can be resampled for a region or period are reduced. Thus, the variability in the contact pattern can be underestimated. Other models create look-up tables of movement distances [22], which poses a similar problem. When considering fine-grained spatiotemporal resolution, the tables for a specific region or period may be based on few movements. This may underestimate the variability in possible contacts in epidemic models.

Also, an advantage of our kernel approach is that it separates the underlying distance dependence in animal movement contacts from the spatial distribution of farms. Directly using the data to create look-up tables may be problematic when there is spatial heterogeneity in farm density, as is the case for Sweden Fig 1. The observed distribution of transports may largely be the result of the spatial configuration of farms, and the distribution of possible destination farms is not the same for e.g. shipping farms in sparse and dense areas. The kernel approach circumvents this issue by modeling the relative probability of destination farms as a function of distance from the shipping farm, and realized movement distances are modeled as unique for each farm location. Thus, we propose that the framework considered here is a promising approach and could be used to improve epidemiological models where animal movement is an important factor.

## Supporting Information

S1 Fig Median shape and scale estimates for counties in Sweden at monthly, quarterly and annual level, presented on the log scale. (PDF)

S2 Fig Medians and corresponding 95% credibility intervals of the posterior predictive distribution of the 50th (dots) and the 95th (diamonds) percentiles of movement distances per month and county, based on parameters from 2007. Dashed and dotted lines indicate the corresponding quantities in the observed data from 2008.
(PDF)

S3 Fig Markov chains for hyper prior parameters for model M_cy_. Top and bottom rows show, from left to right, hyper prior shape and scale parameters for *σ* and *κ*, respectively.
(TIF)
