# Supplementary material for: Spatiotemporal Variation in Distance Dependent Animal Movement Contacts: One Size Doesn’t Fit All
Source: PLoS One. 2016 Oct 19;11(10):e0164008. doi: 10.1371/journal.pone.0164008 (PMC5070834; doi:10.1371/journal.pone.0164008)
Supplement: S1 Fig — (PDF) [file pone.0164008.s001.pdf]

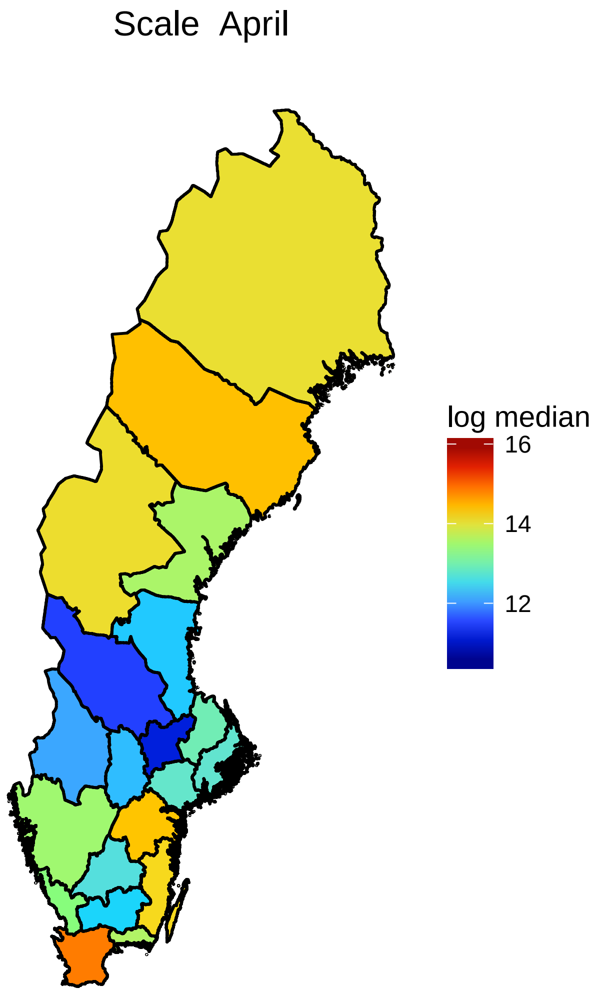
Scale April
log median
16
14
12

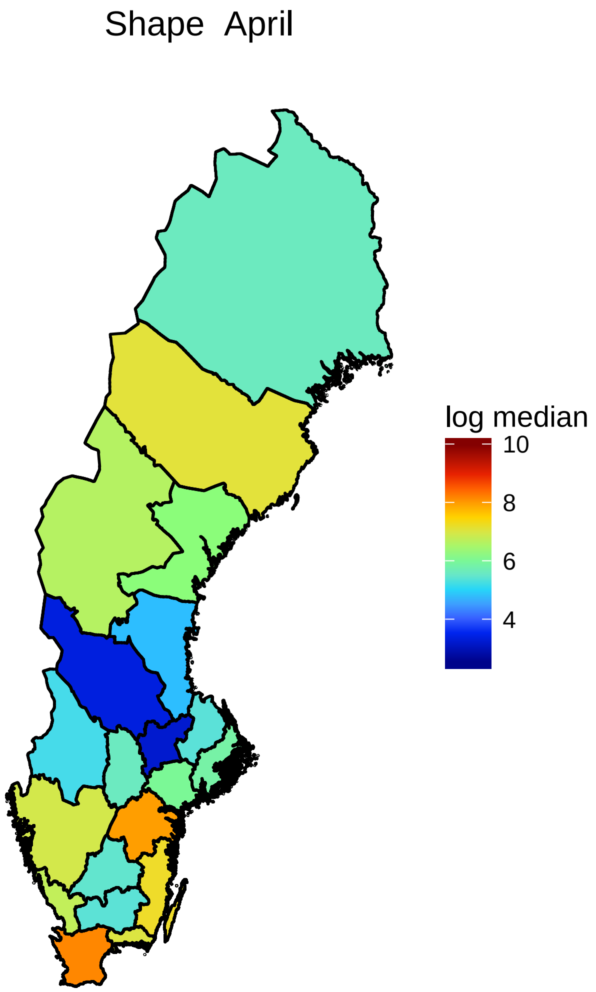
Shape April
log median
10
8
6
4

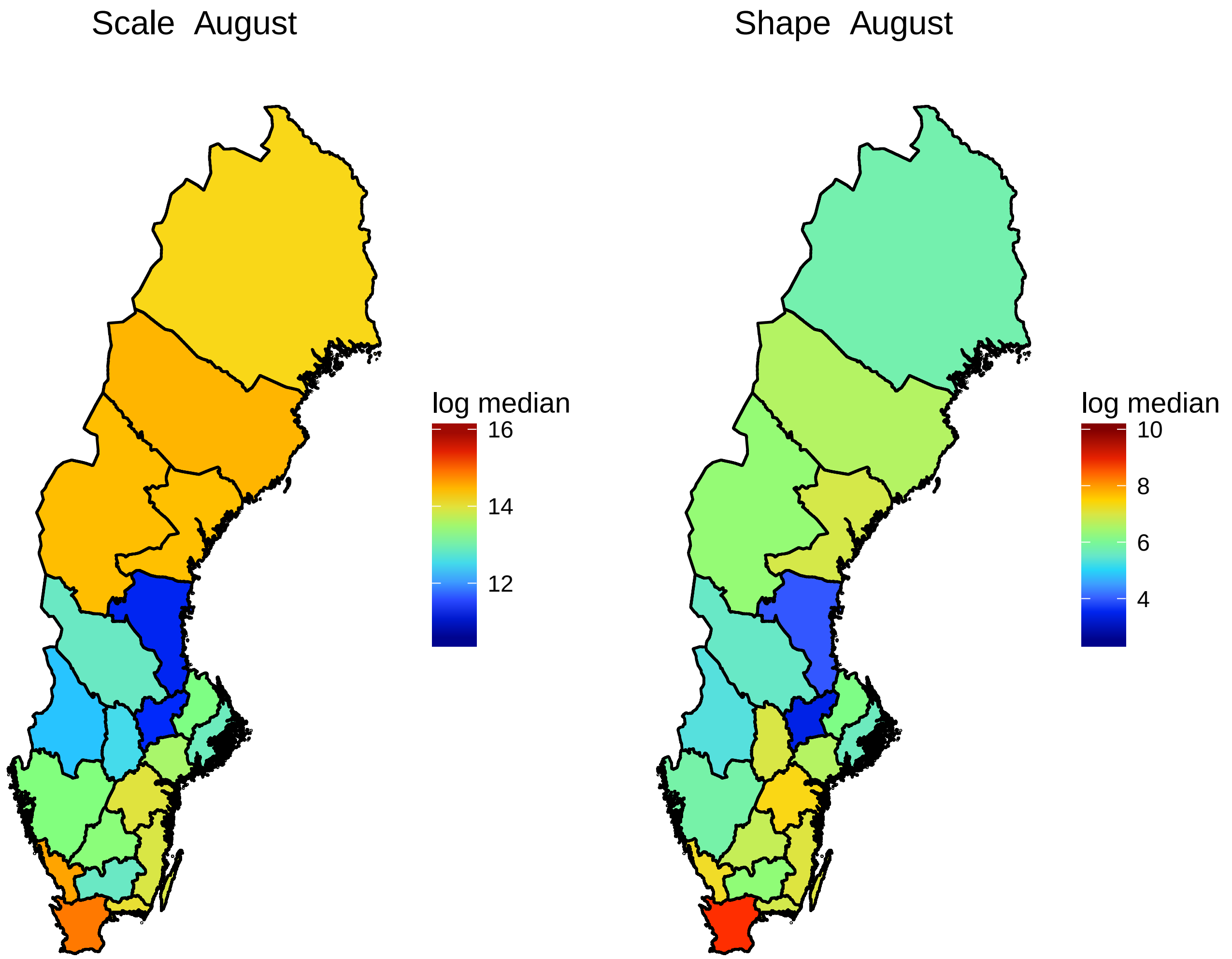
Scale August
log median
16
14
12
Shape August
log median
10
8
6
4

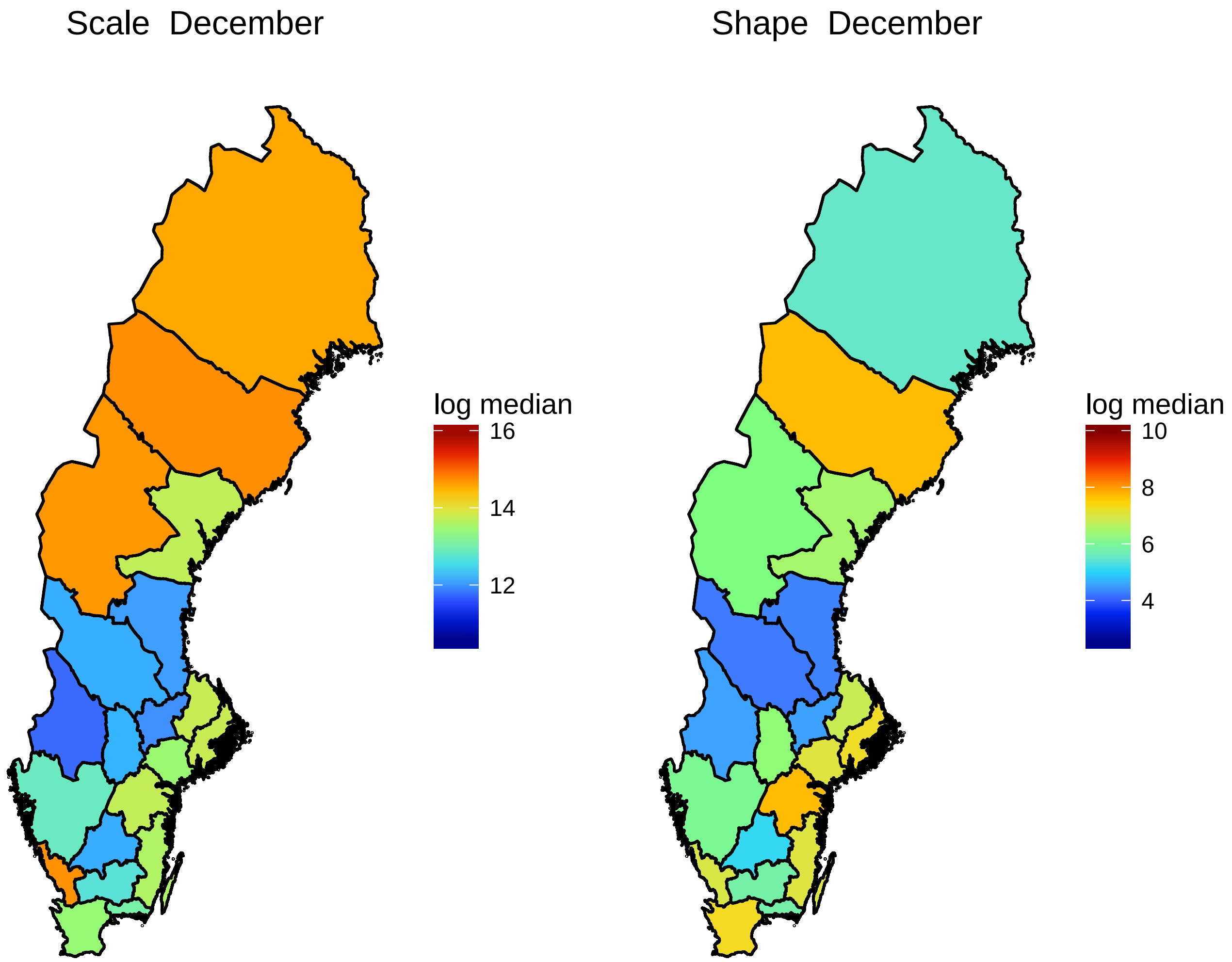

Scale December
log median
16
14
12
Shape December
log median
10
8
6
4

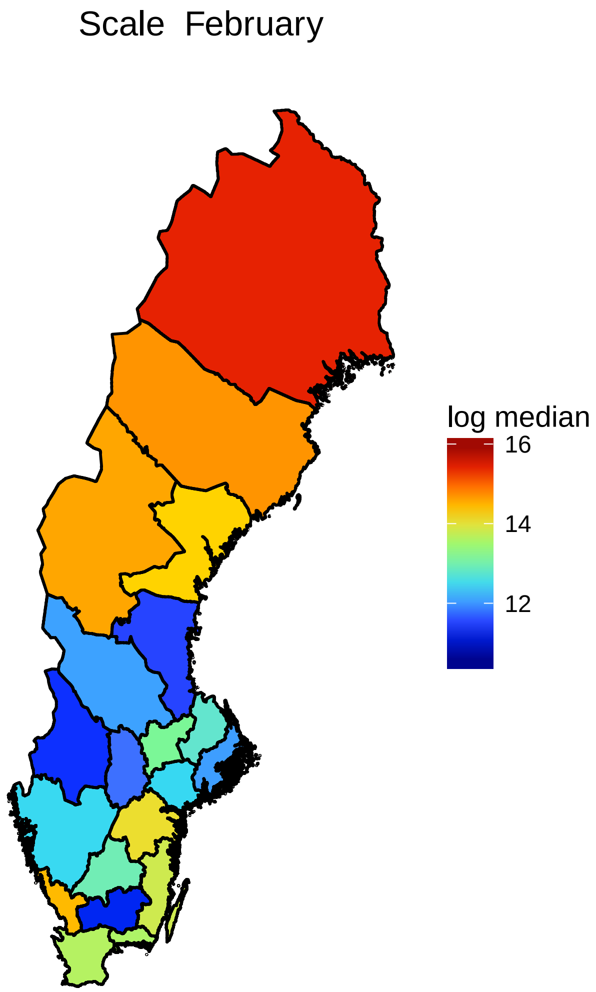
Scale February
log median
16
14
12

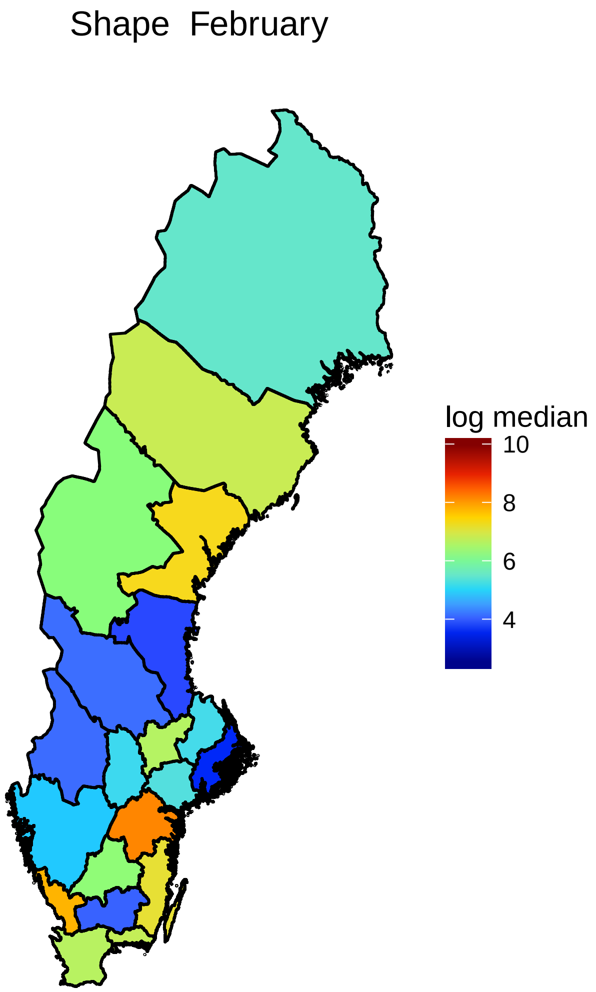
Shape February
log median
10
8
6
4

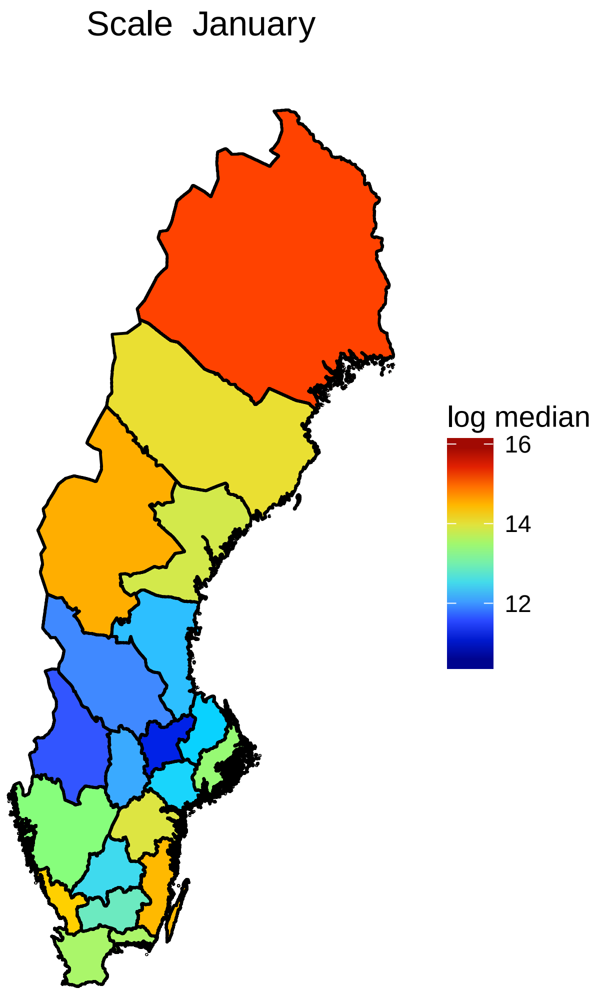
Scale January
log median
16
14
12

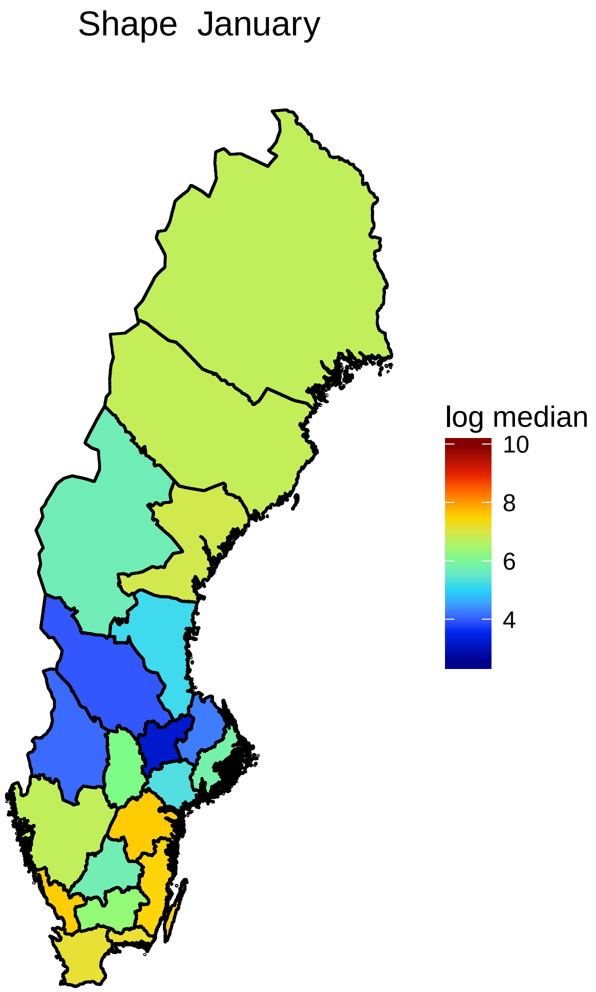
Shape January
log median
10
8
6
4

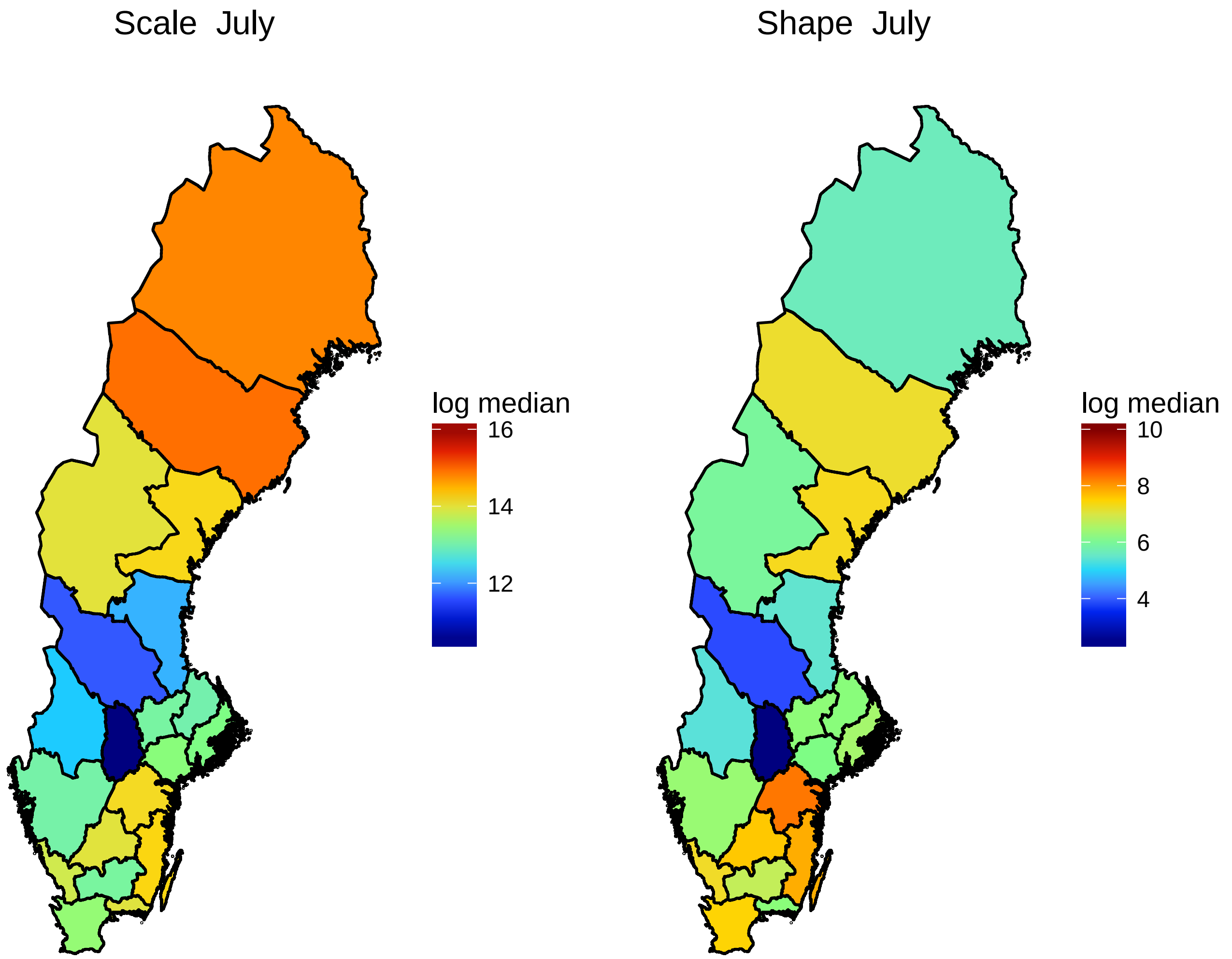

Scale July
log median
16
14
12
Shape July
log median
10
8
6
4

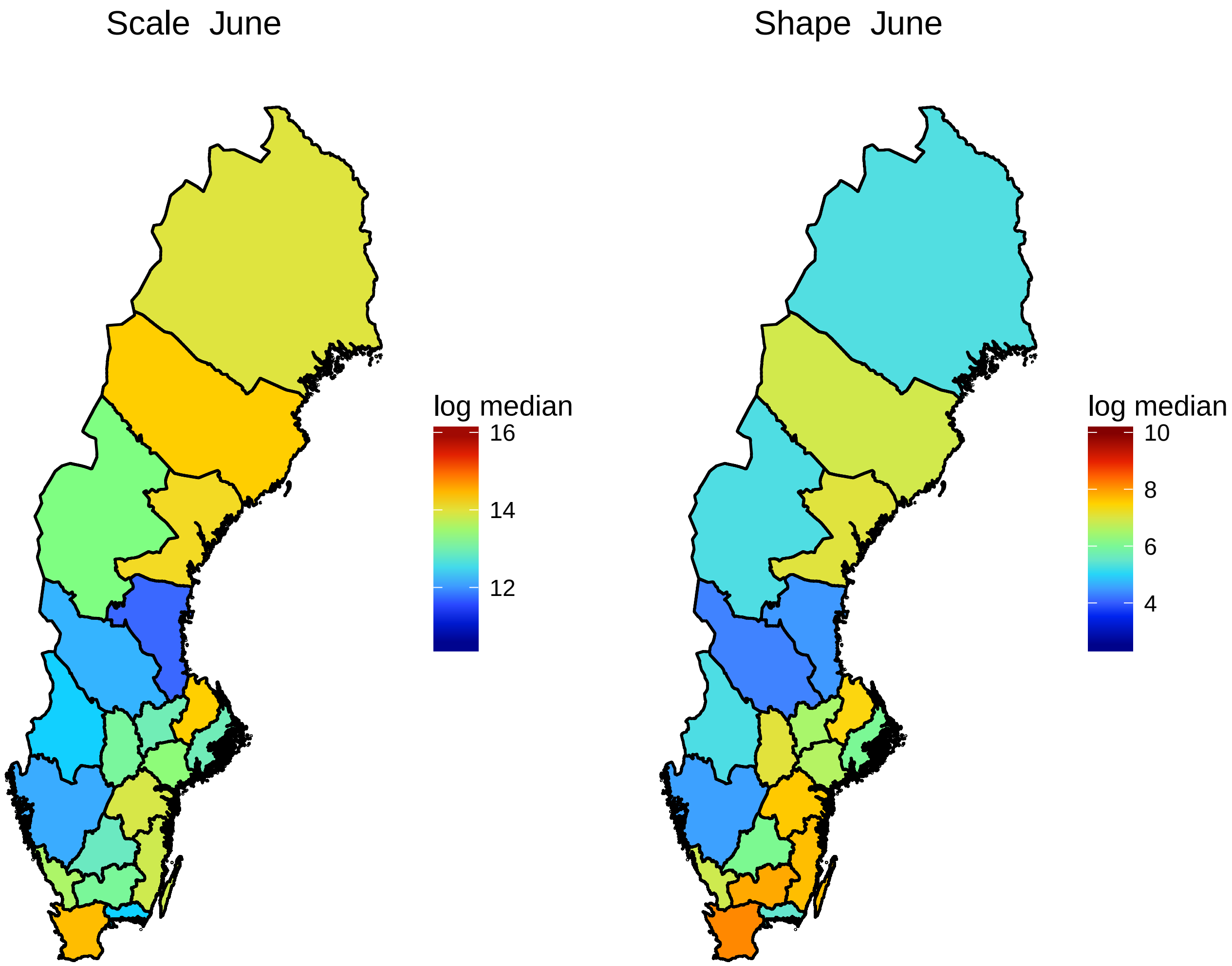
Scale June
log median
16
14
12
Shape June
log median
10
8
6
4

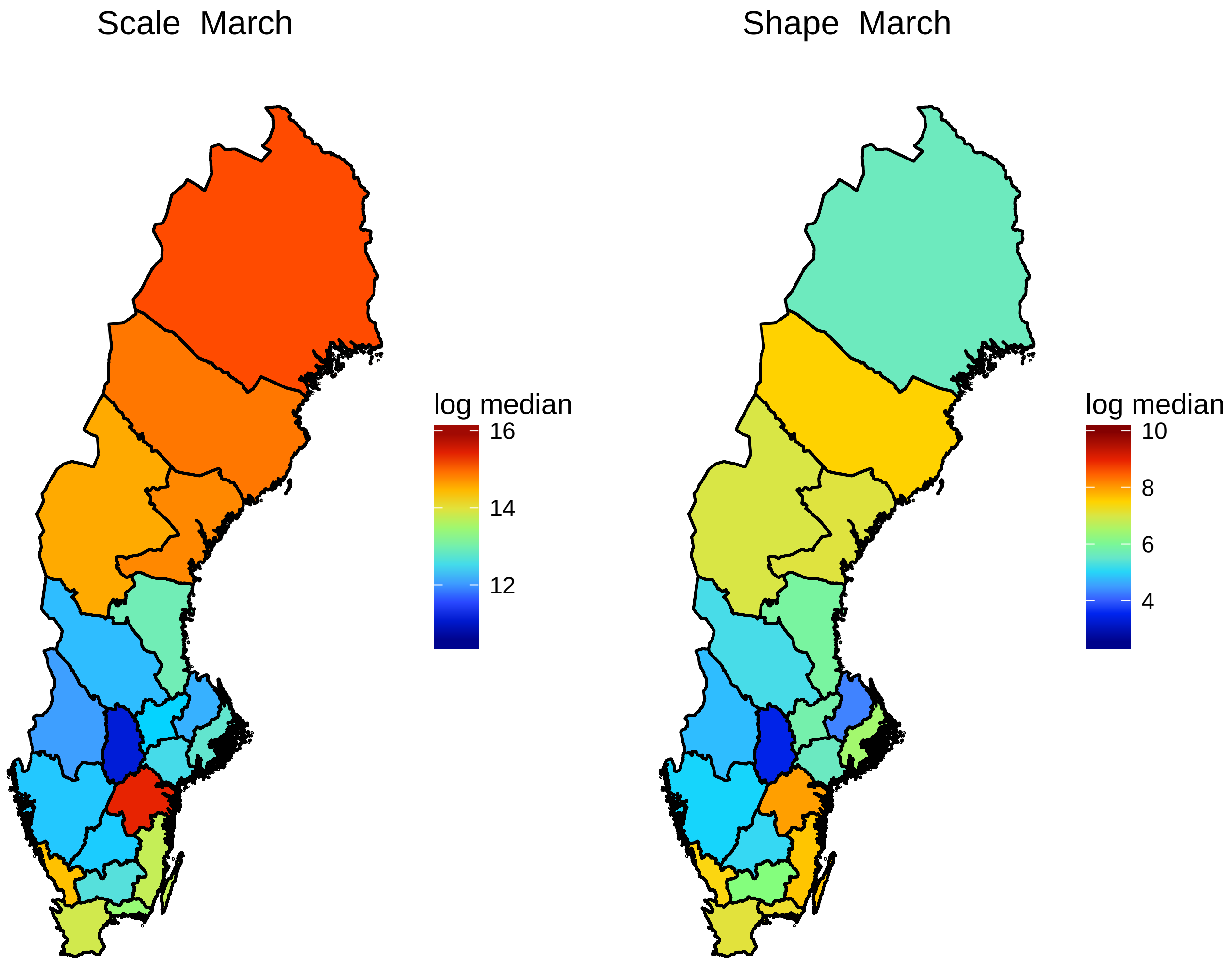
Scale March
log median
16
14
12
Shape March
log median
10
8
6
4

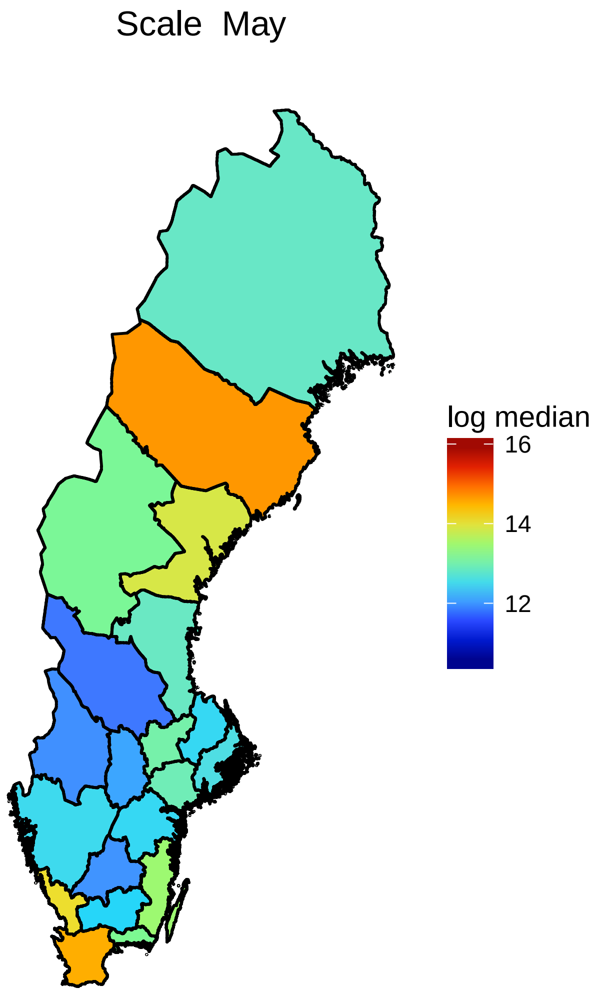

Scale May
log median
16
14
12

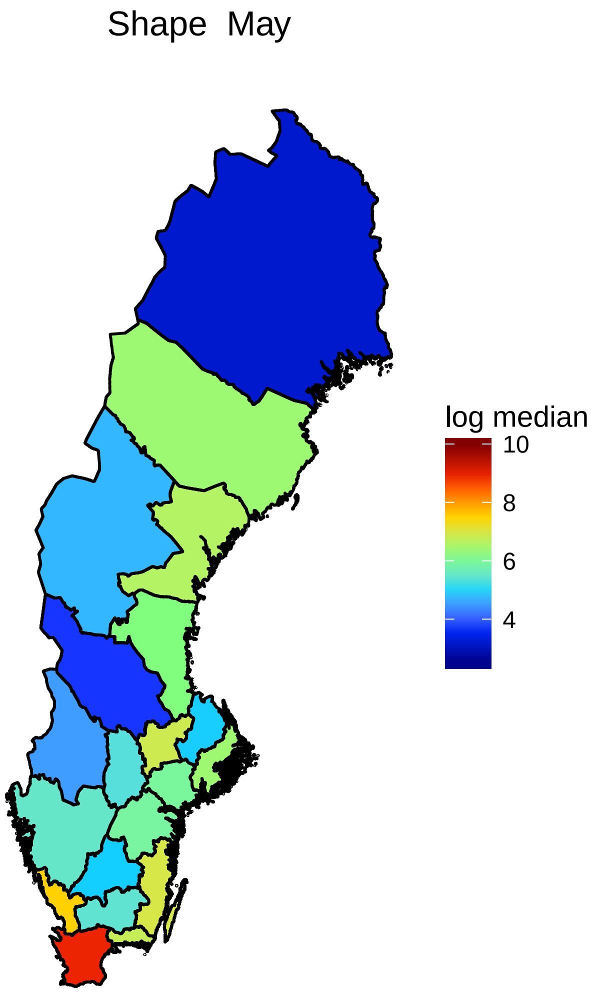

Shape May
log median
10
8
6
4

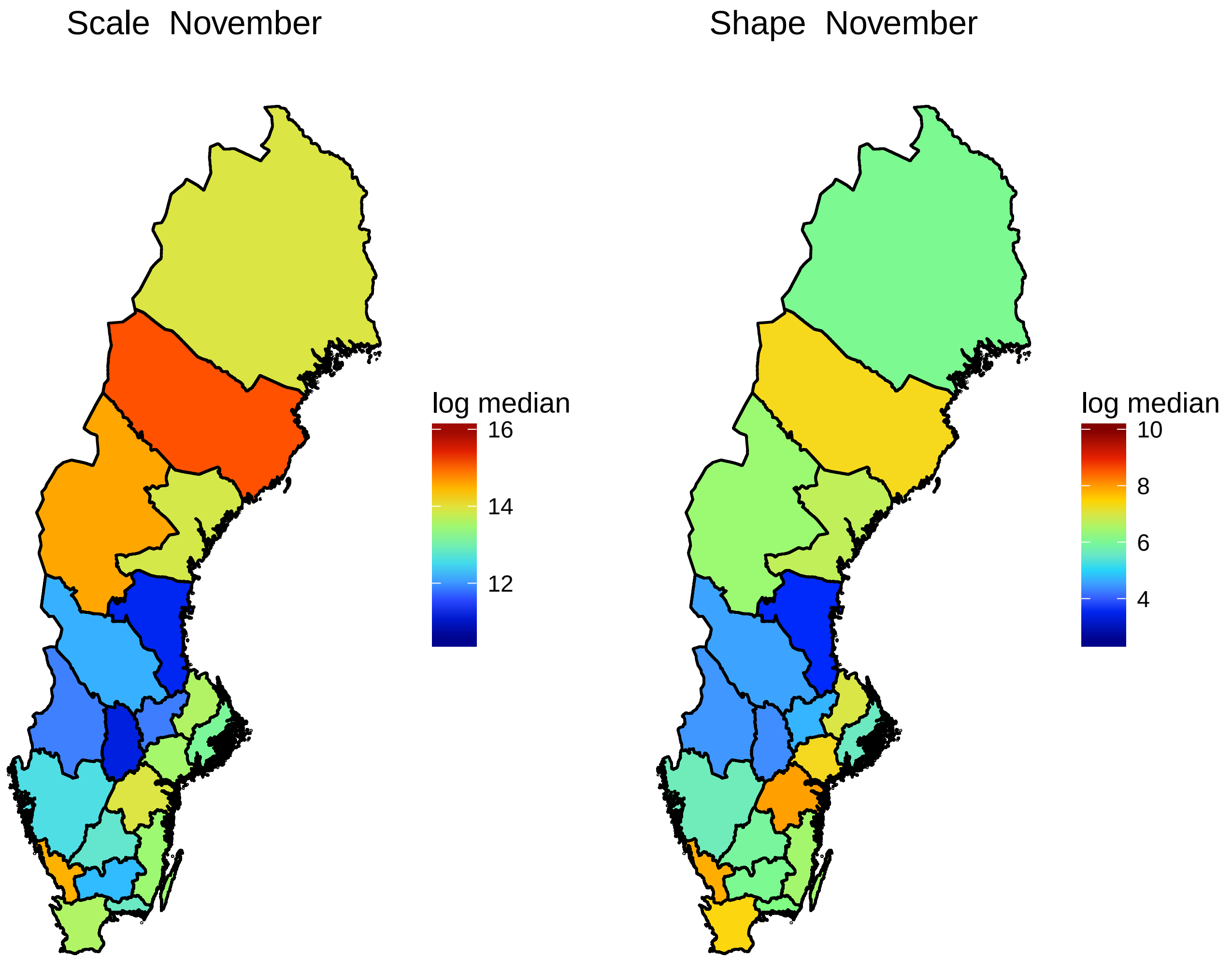

Scale November
log median
16
14
12
Shape November
log median
10
8
6
4

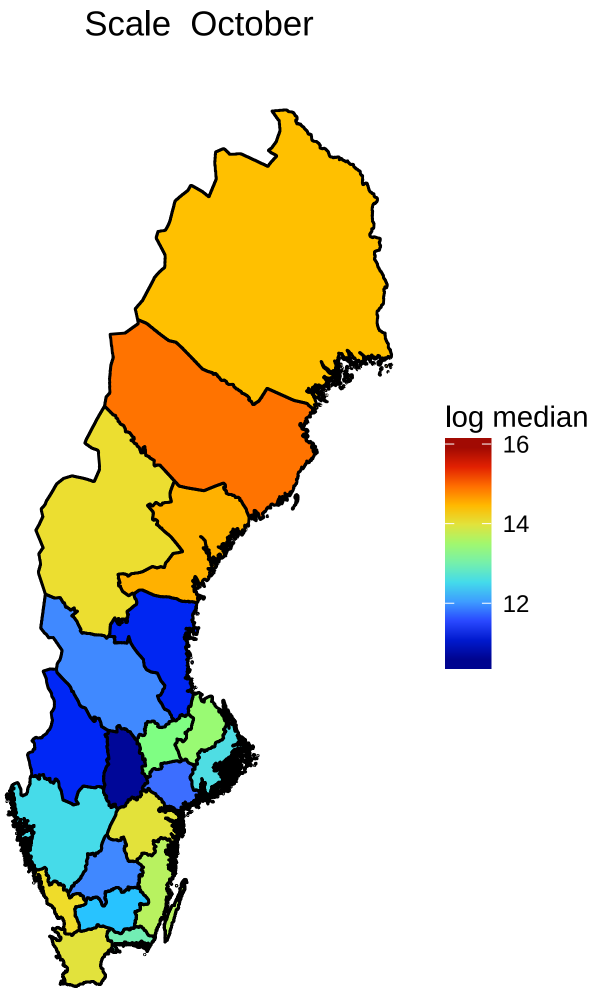

Scale October
log median
16
14
12

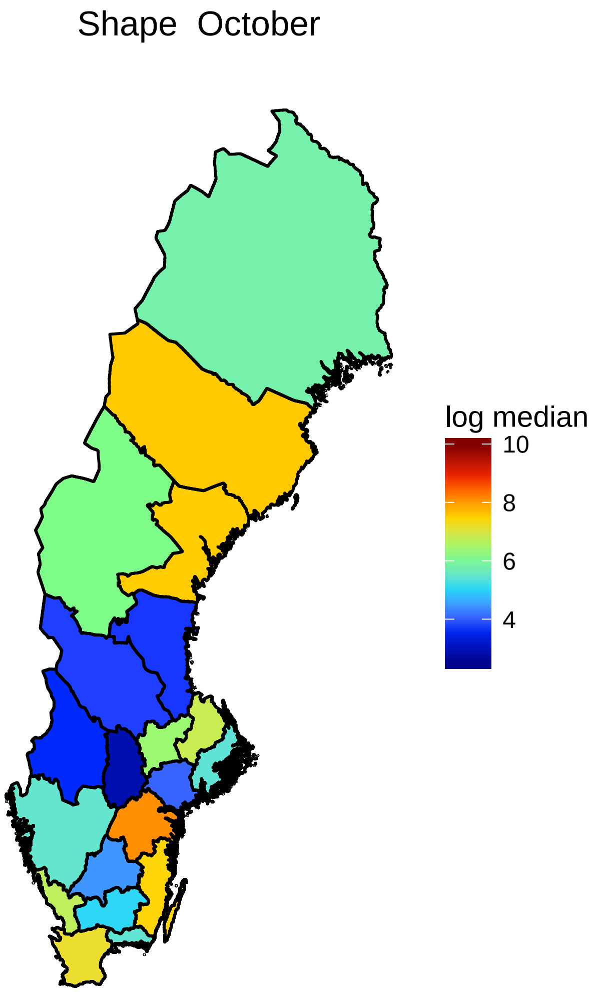

Shape October
log median
10
8
6
4

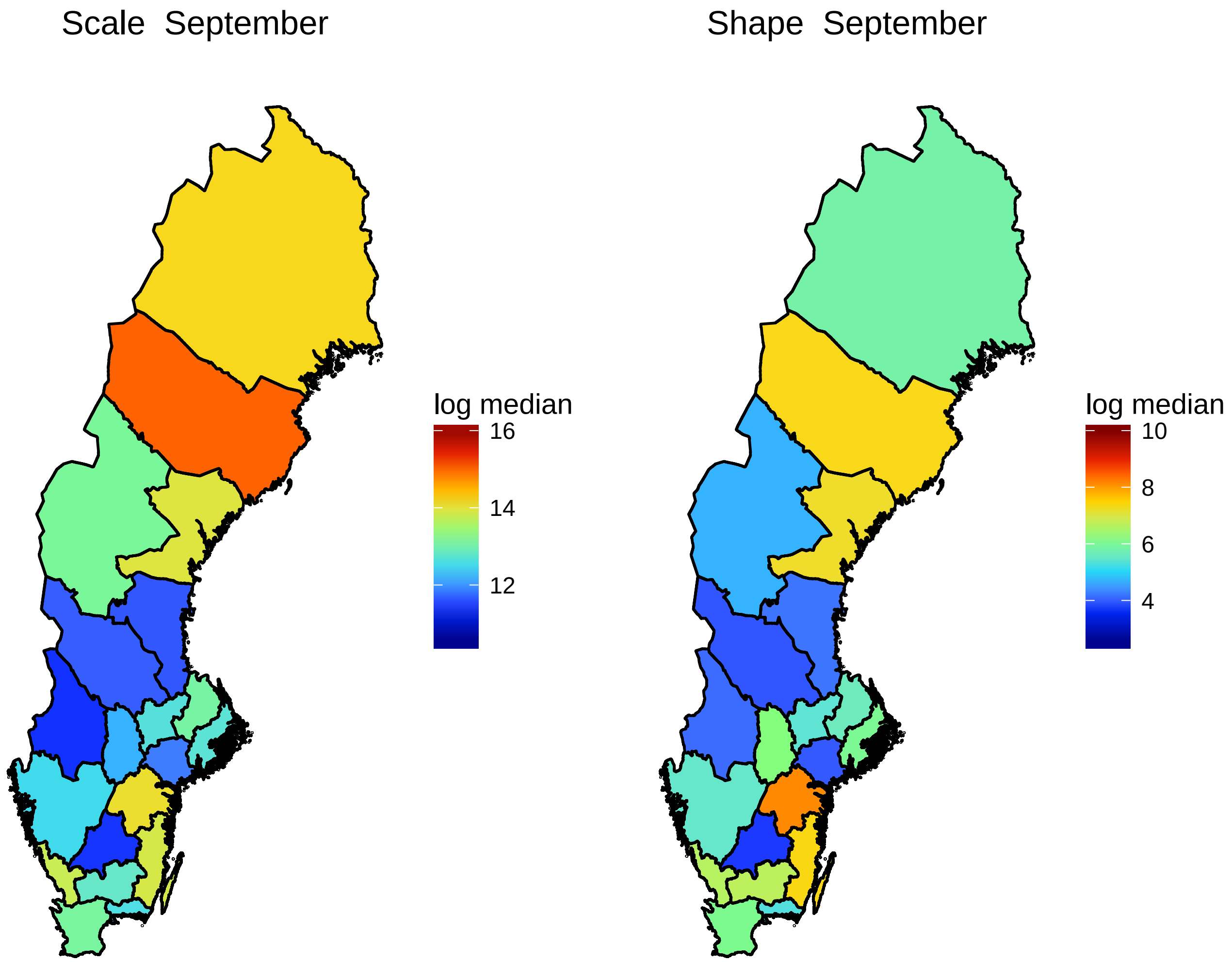

Scale September
log median
16
14
12
Shape September
log median
10
8
6
4

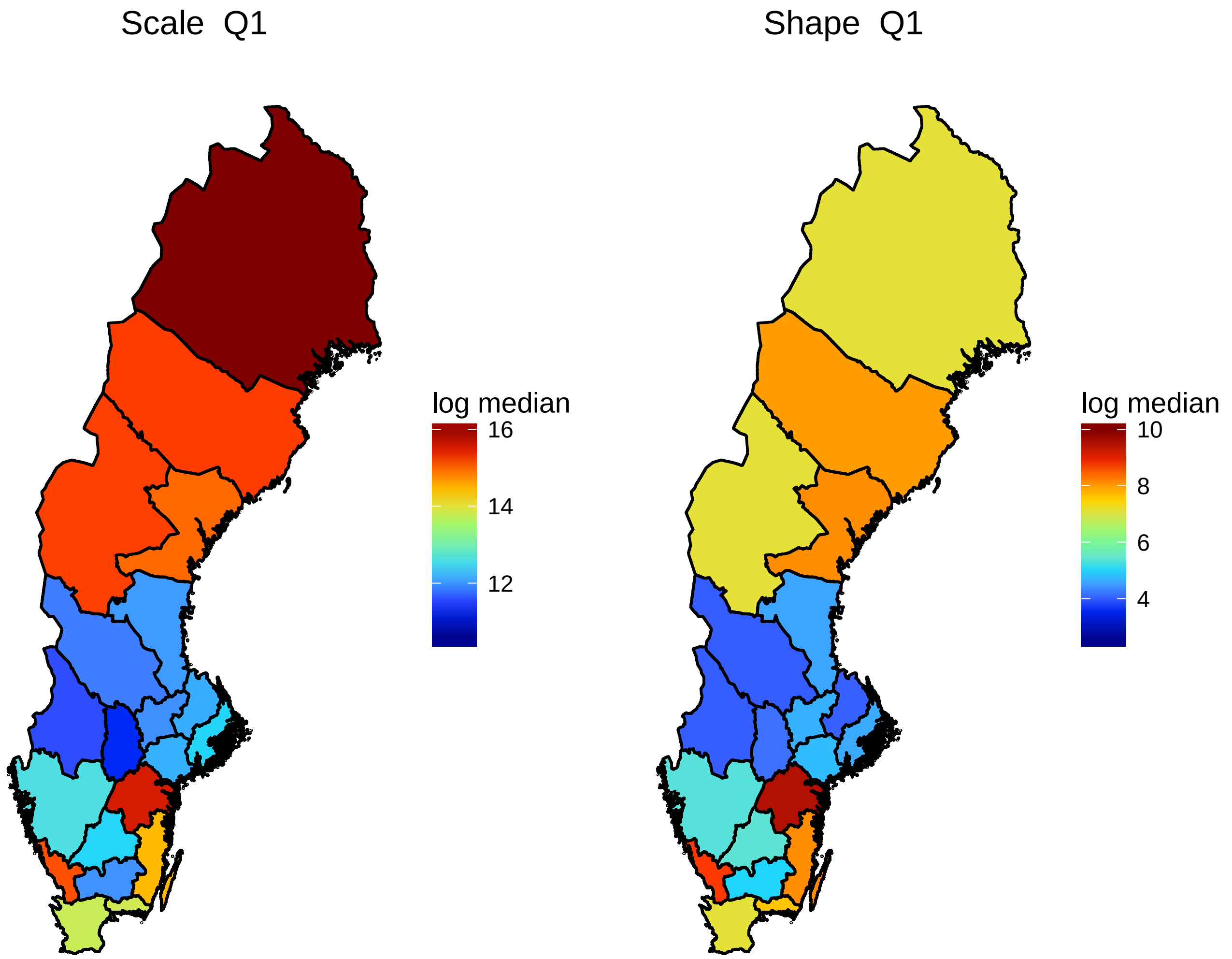

Scale Q1
log median
16
14
12
Shape Q1
log median
10
8
6
4

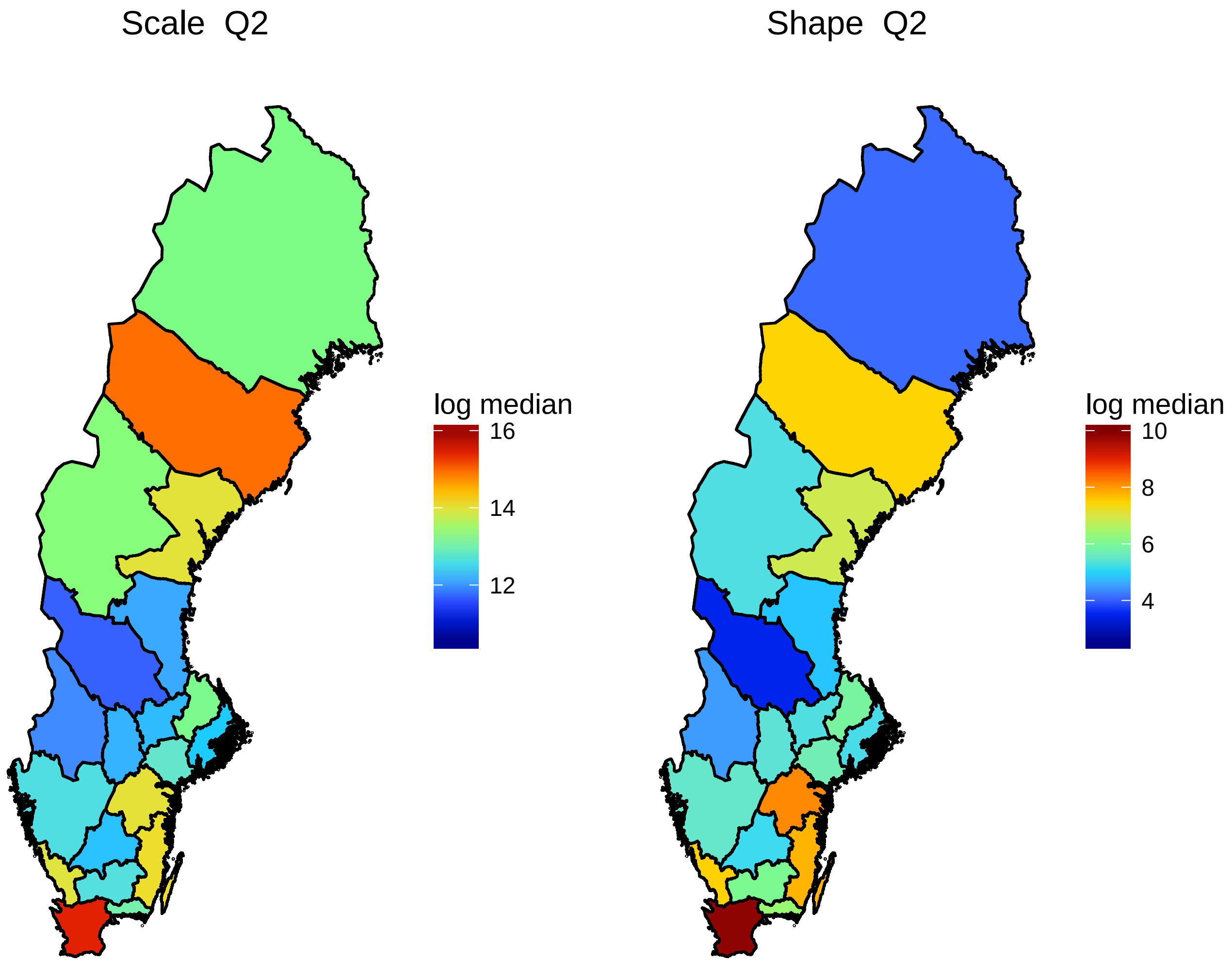

Scale Q2
log median
16
14
12
Shape Q2
log median
10
8
6
4

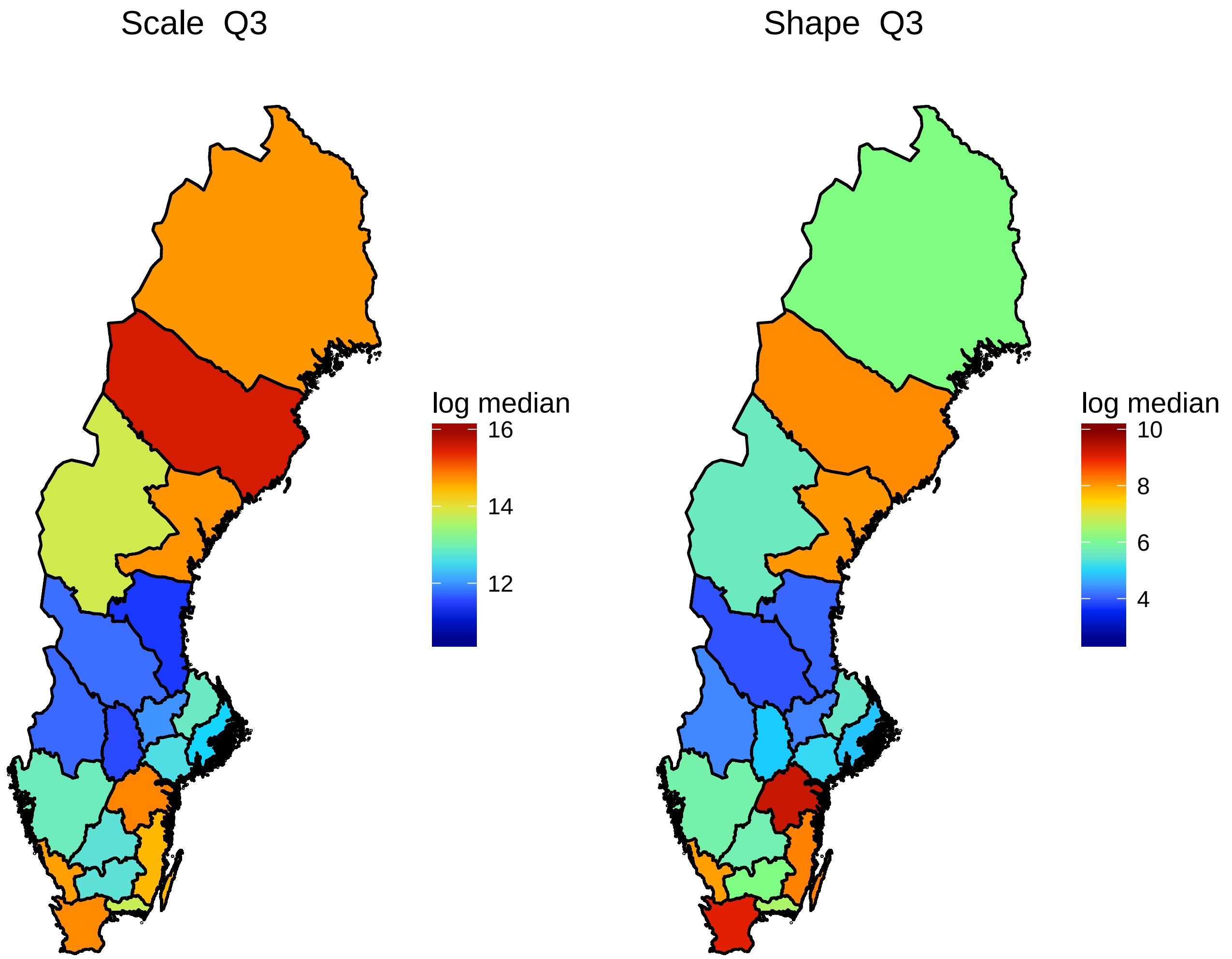
Scale Q3
log median
16
14
12
Shape Q3
log median
10
8
6
4

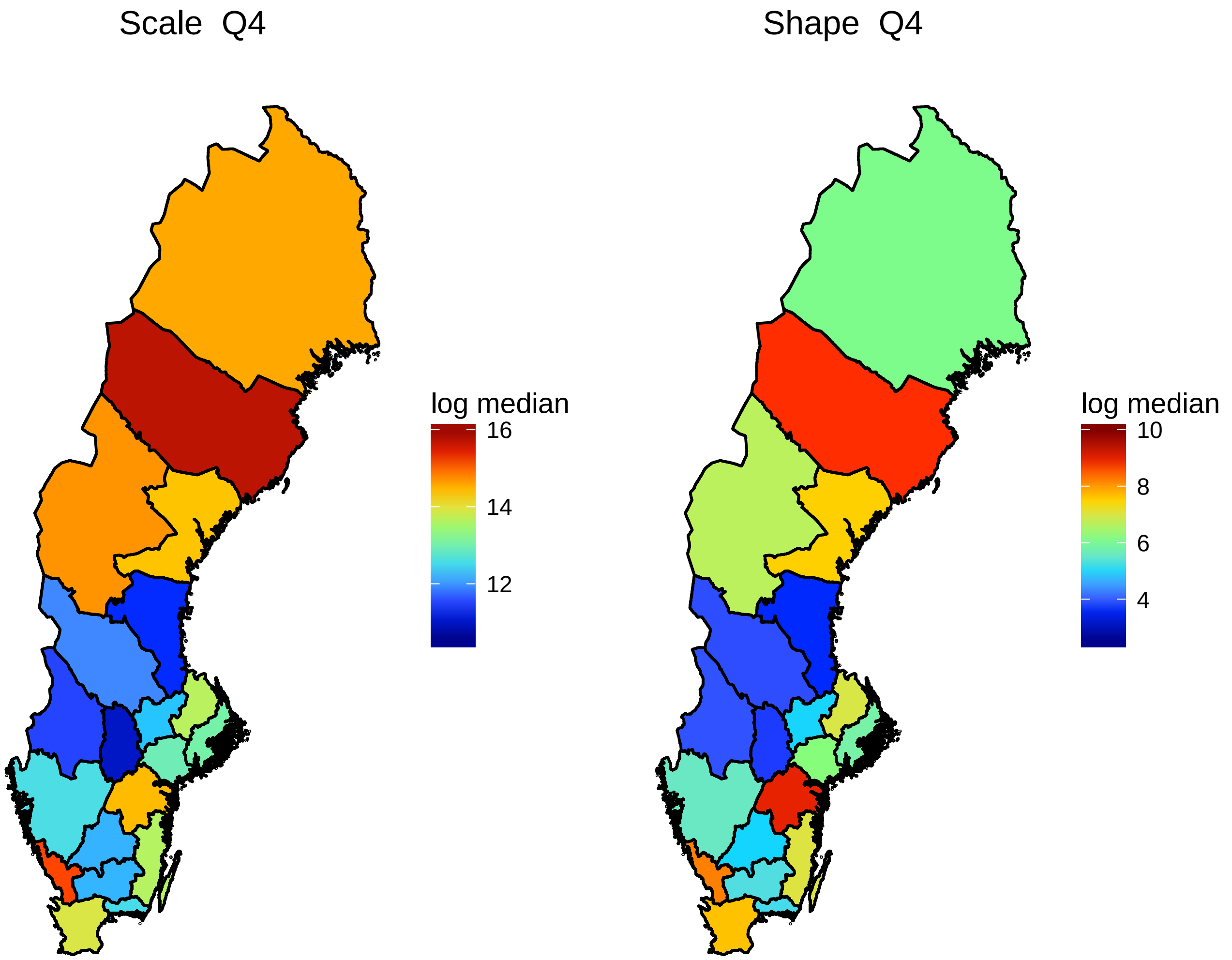
Scale Q4
log median
16
14
12
Shape Q4
log median
10
8
6
4

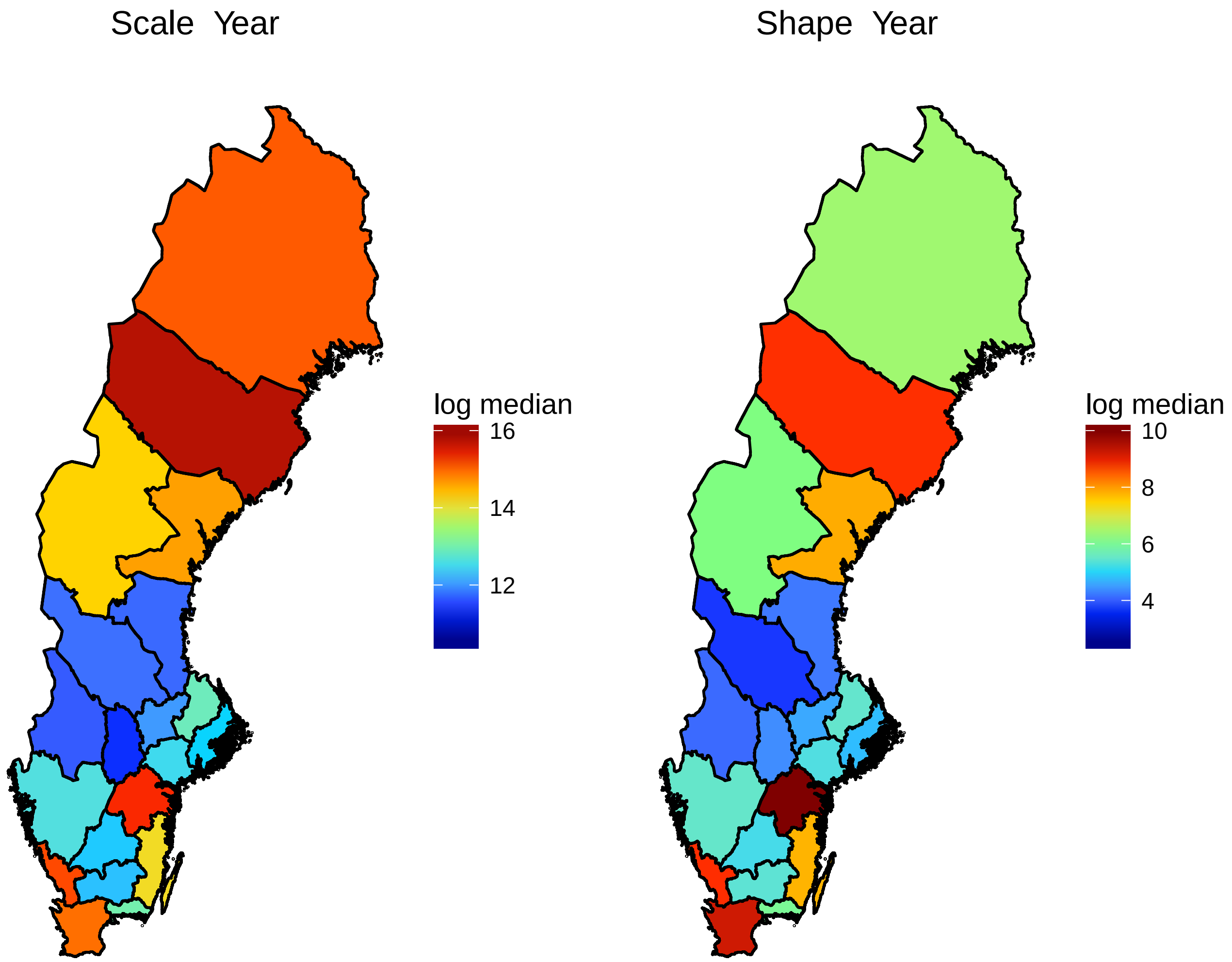
Scale Year
log median
16
14
12
Shape Year
log median
10
8
6
4
