# Supplementary material for: Spatiotemporal Variation in Distance Dependent Animal Movement Contacts: One Size Doesn’t Fit All
Source: PLoS One. 2016 Oct 19;11(10):e0164008. doi: 10.1371/journal.pone.0164008 (PMC5070834; doi:10.1371/journal.pone.0164008)
Supplement: S2 Fig — Dashed and dotted lines indicate the corresponding quantities in the observed data from 2008. (PDF) [file pone.0164008.s002.pdf]

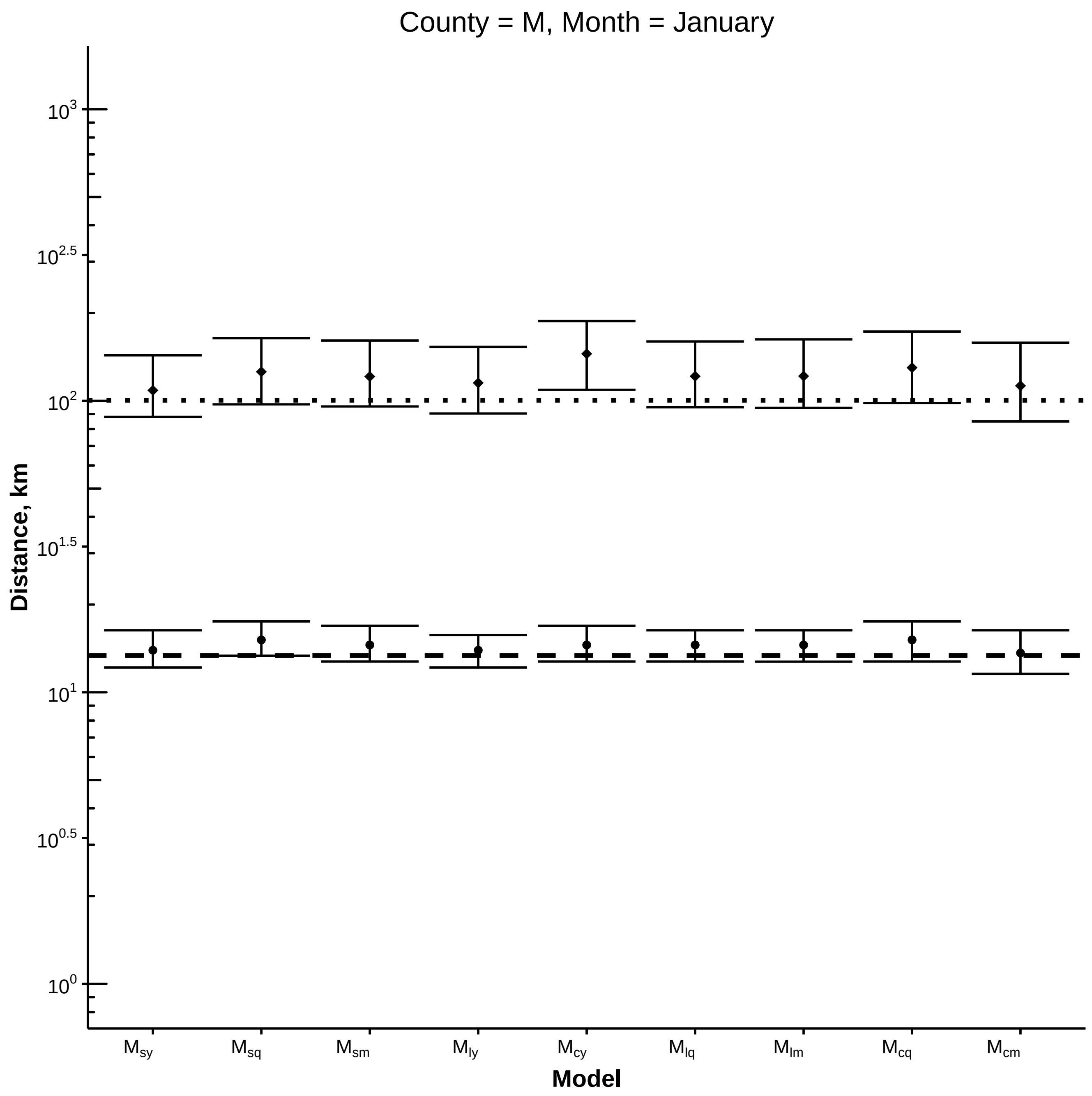

County = M, Month = January
Distance, km
10^3
10^2.5
10^2
10^1.5
10^1
10^0.5
10^0
Msy
Msq
Msm
Mly
Mcy
Mlq
Mlm
Mcq
Mcm
Model

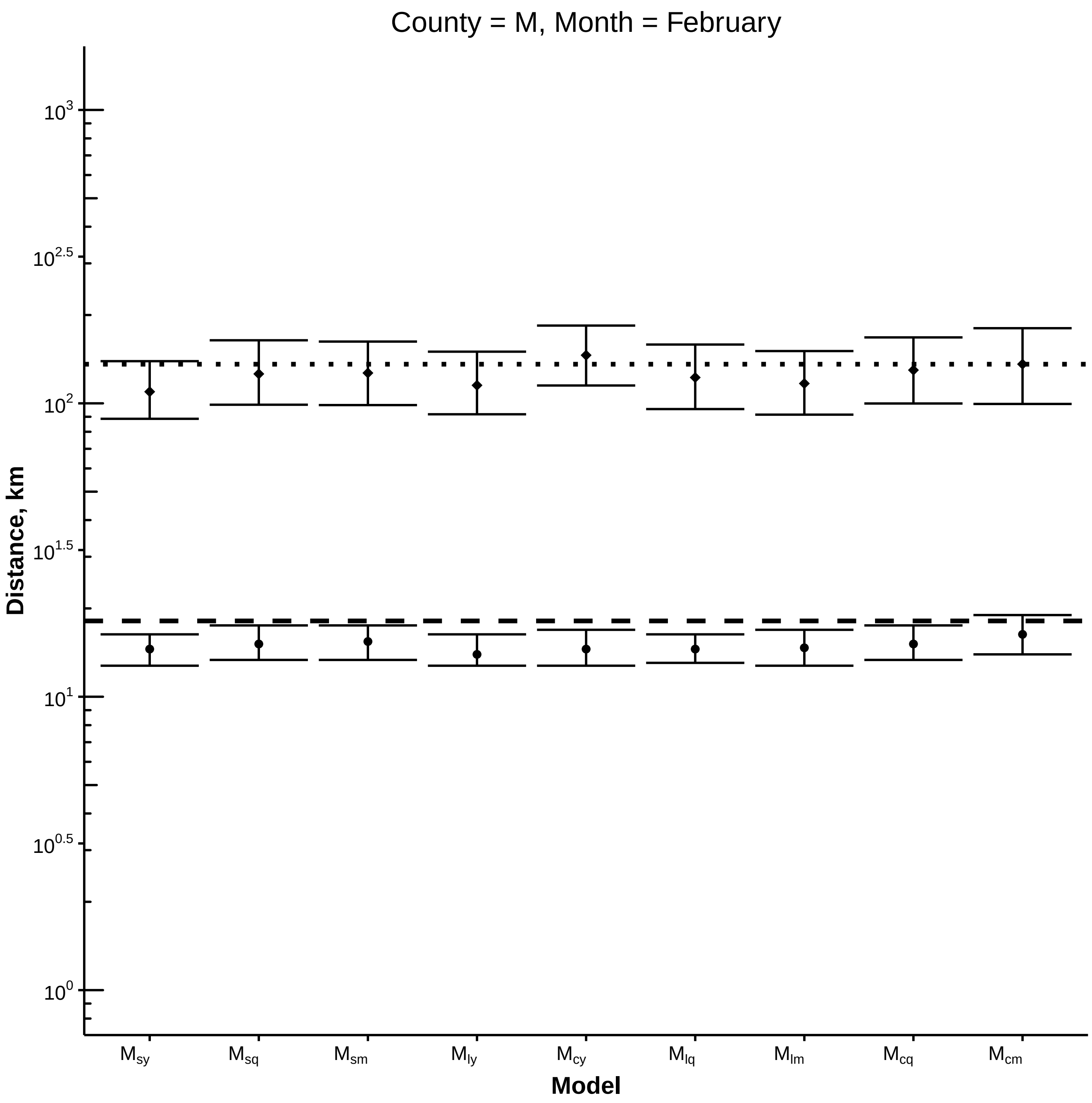

County = M, Month = February
Distance, km
Model
10^3
$10^{2.5}$
10^2
$10^{1.5}$
10^1
$10^{0.5}$
10^0
M_{sy}
M_{sq}
M_{sm}
M_{ly}
M_{cy}
M_{lq}
M_{lm}
M_{cq}
M_{cm}

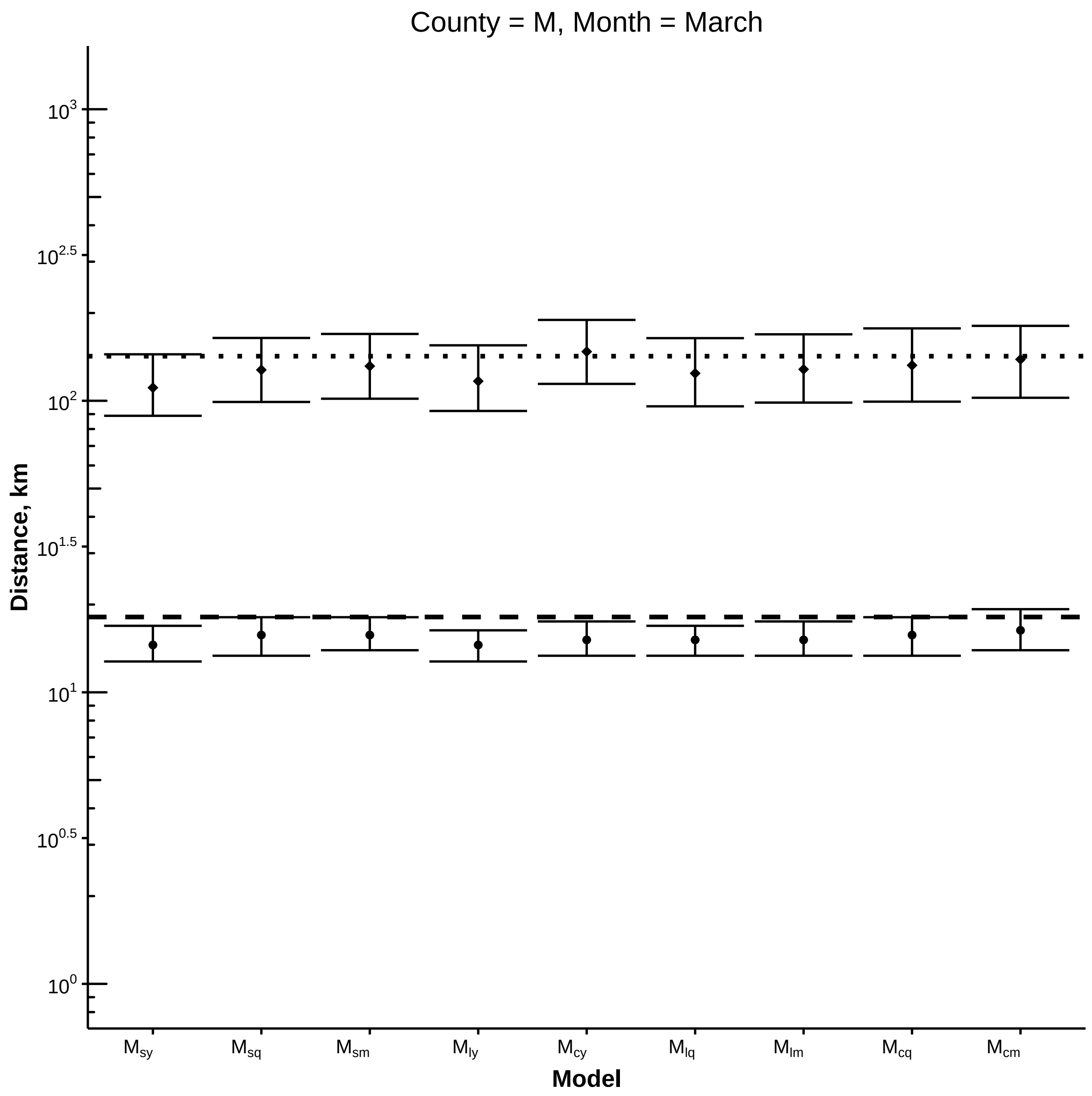
County = M, Month = March
Distance, km
10^3
10^2.5
10^2
10^1.5
10^1
10^0.5
10^0
Model
Msy
Msq
Msm
Mly
Mcy
Mlq
Mlm
Mcq
Mcm

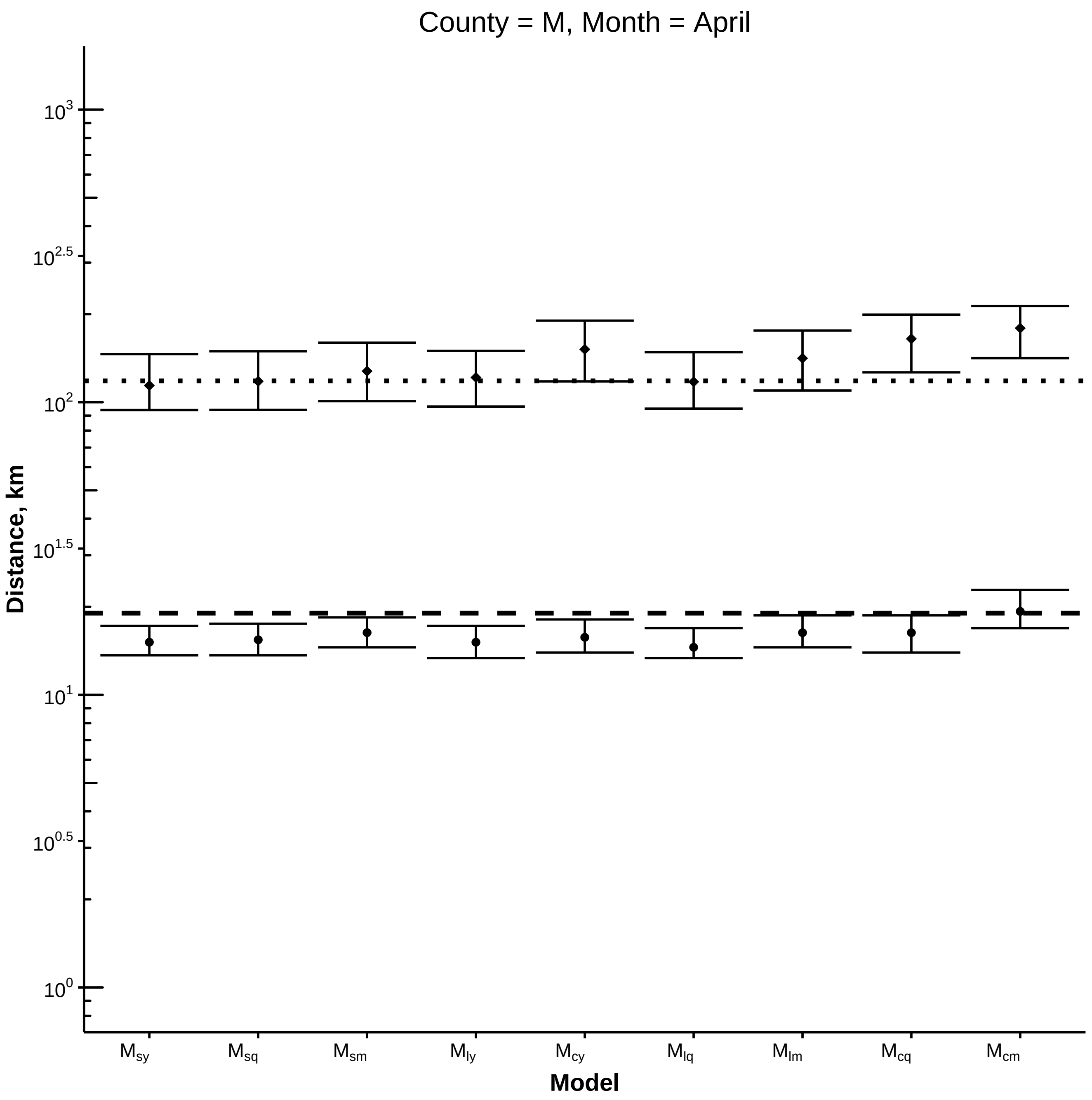

County = M, Month = April
Distance, km
Model
10^3
$10^{2.5}$
10^2
$10^{1.5}$
10^1
$10^{0.5}$
10^0
M_{sy}
M_{sq}
M_{sm}
M_{ly}
M_{cy}
M_{lq}
M_{lm}
M_{cq}
M_{cm}

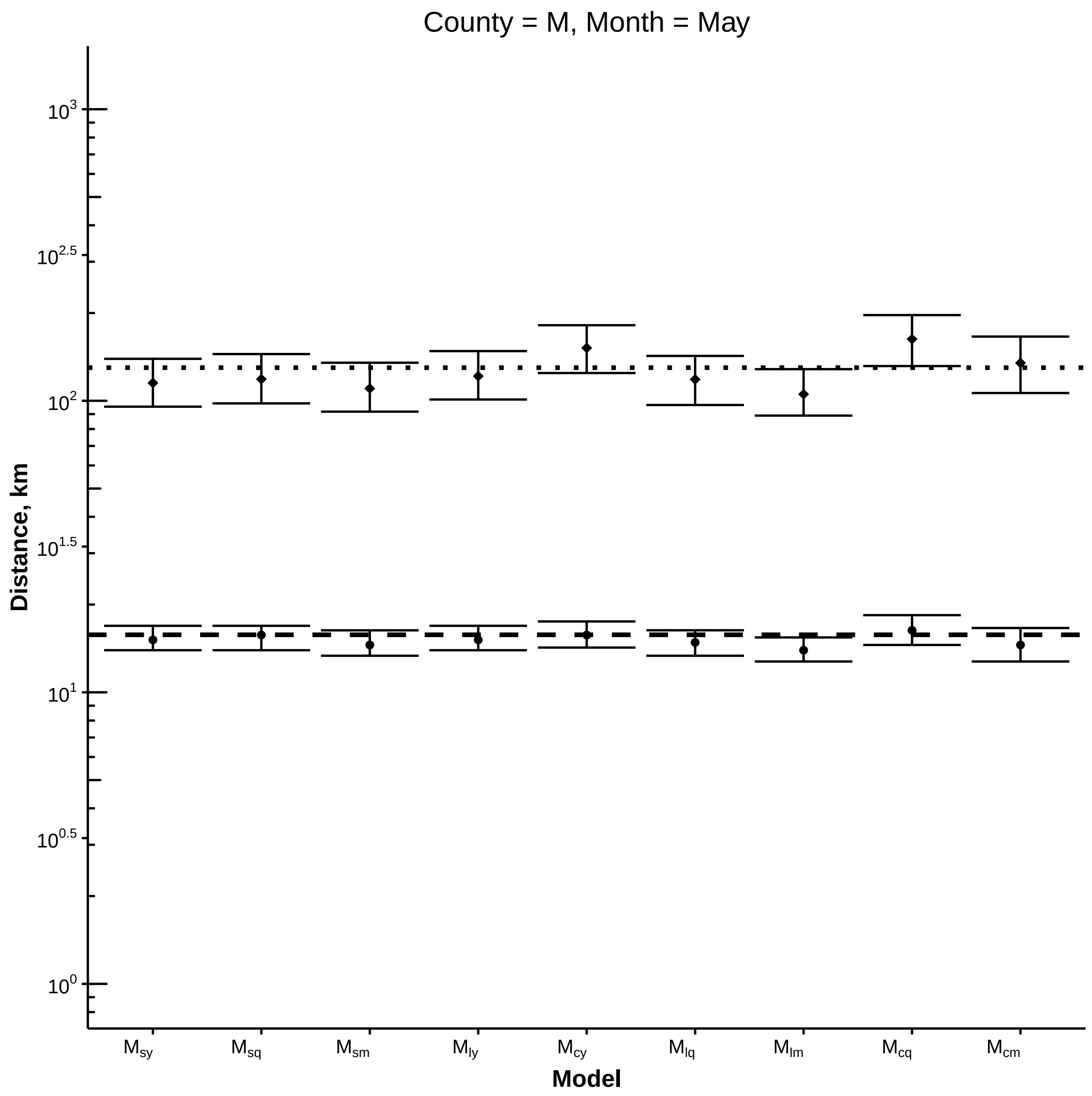

County = M, Month = May
Distance, km
10^3
$10^{2.5}$
10^2
$10^{1.5}$
10^1
$10^{0.5}$
10^0
M_{sy}
M_{sq}
M_{sm}
M_{ly}
M_{cy}
M_{lq}
M_{lm}
M_{cq}
M_{cm}
Model

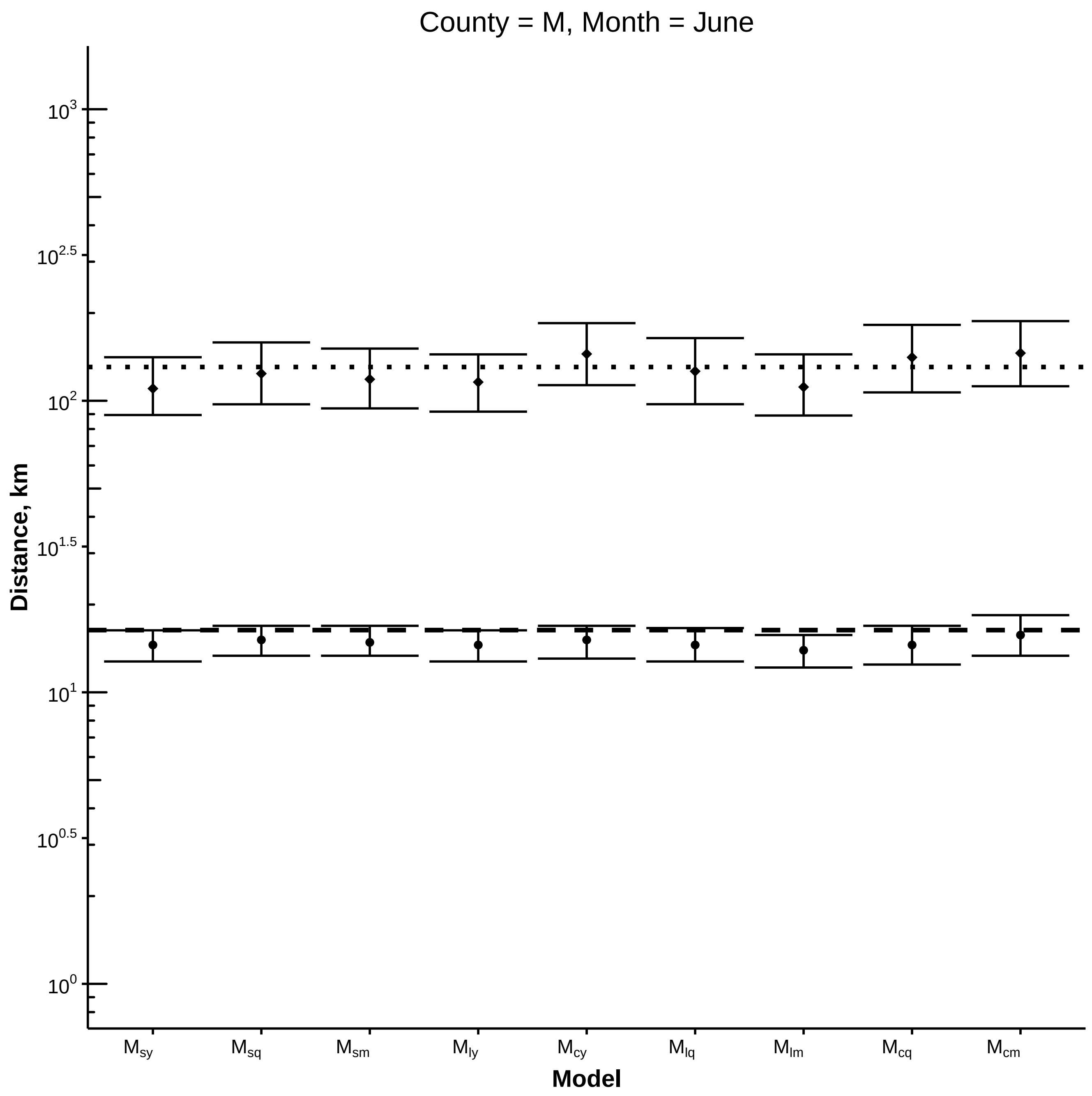

County = M, Month = June
Distance, km
10^3
10^2.5
10^2
10^1.5
10^1
10^0.5
10^0
Msy
Msq
Msm
Mly
Mcy
Mlq
Mlm
Mcq
Mcm
Model

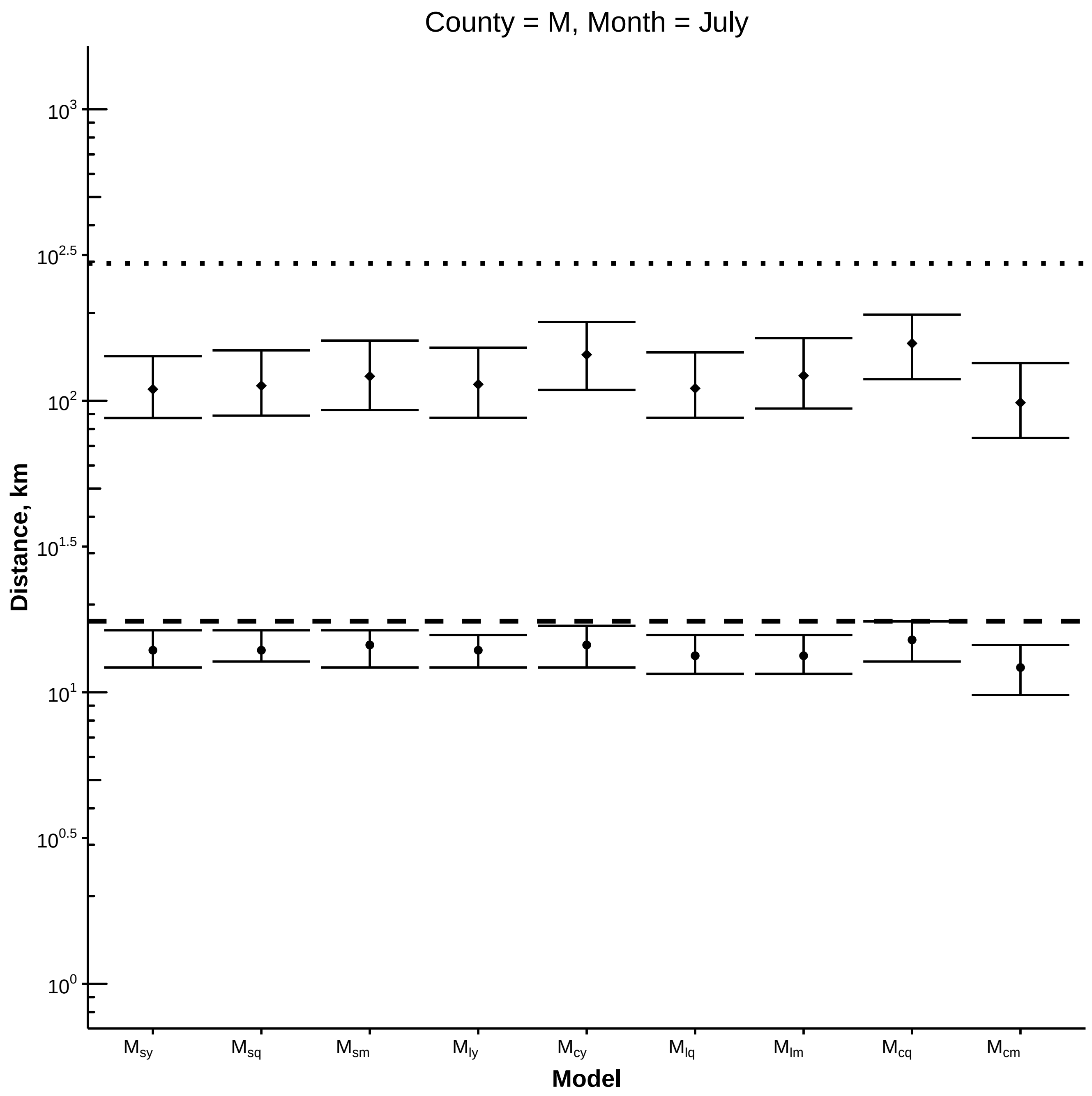

County = M, Month = July
Distance, km
Model
10^3
$10^{2.5}$
10^2
$10^{1.5}$
10^1
$10^{0.5}$
10^0
M_{sy}
M_{sq}
M_{sm}
M_{ly}
M_{cy}
M_{lq}
M_{lm}
M_{cq}
M_{cm}

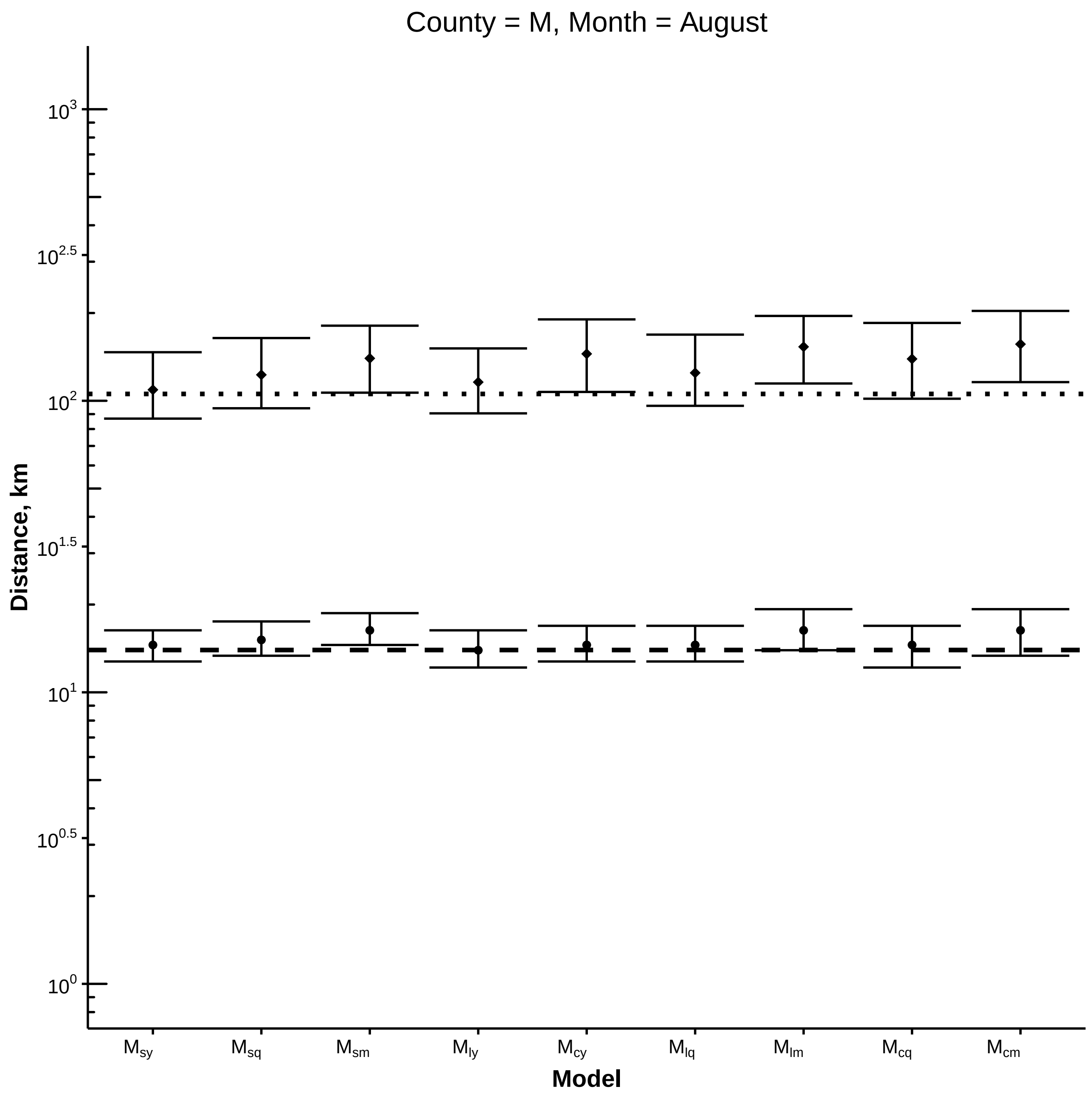
County = M, Month = August
Distance, km
Model
10^3
$10^{2.5}$
10^2
$10^{1.5}$
10^1
$10^{0.5}$
10^0
M_{sy}
M_{sq}
M_{sm}
M_{ly}
M_{cy}
M_{lq}
M_{lm}
M_{cq}
M_{cm}

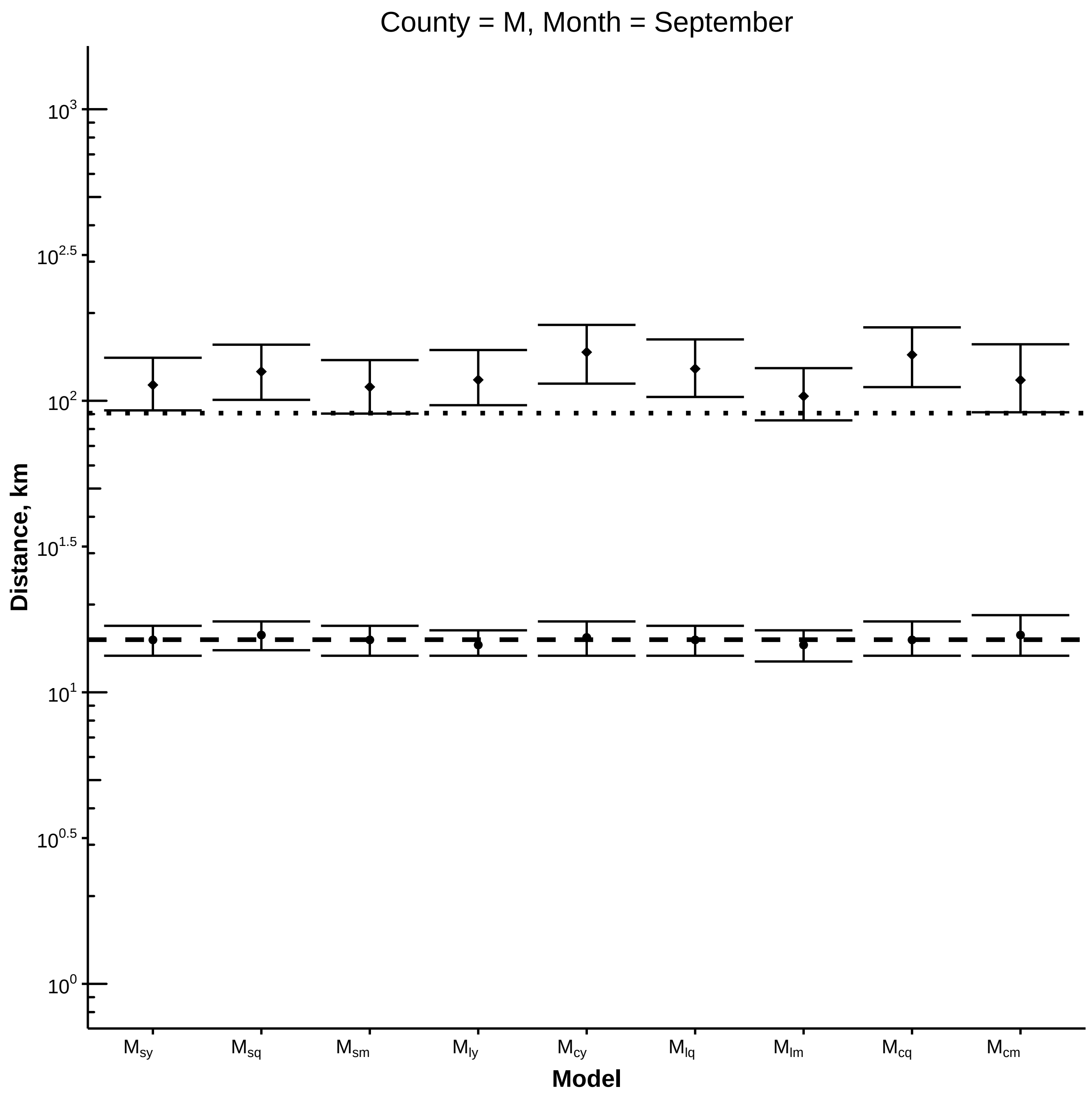

County = M, Month = September
Distance, km
10^{3}
$10^{2.5}$
10^{2}
$10^{1.5}$
10^{1}
$10^{0.5}$
10^{0}
M_{sy}
M_{sq}
M_{sm}
M_{ly}
M_{cy}
M_{lq}
M_{lm}
M_{cq}
M_{cm}
Model

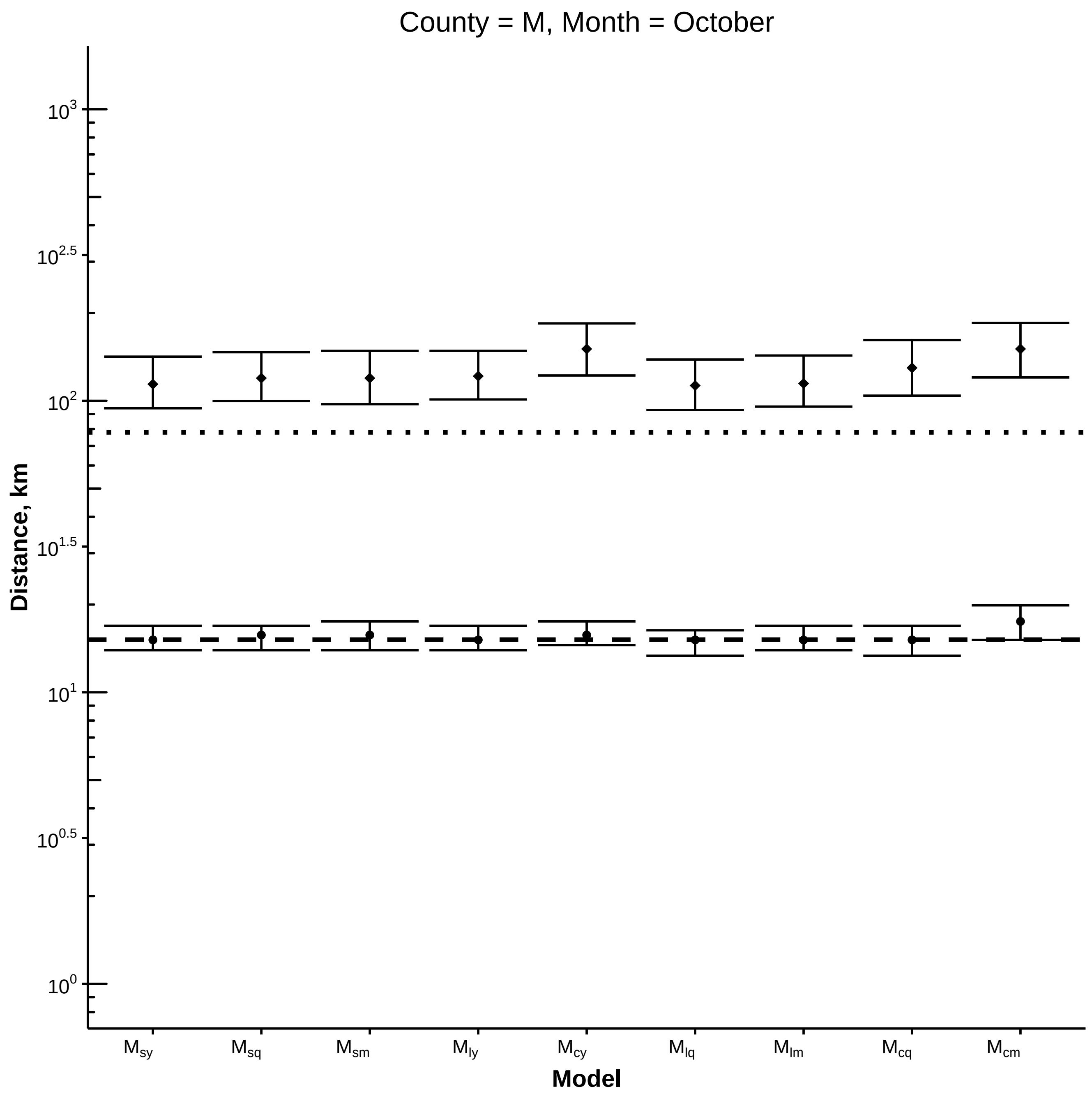
County = M, Month = October
Distance, km
10^3
10^2.5
10^2
10^1.5
10^1
10^0.5
10^0
Msy
Msq
Msm
Mly
Mcy
Mlq
Mlm
Mcq
Mcm
Model

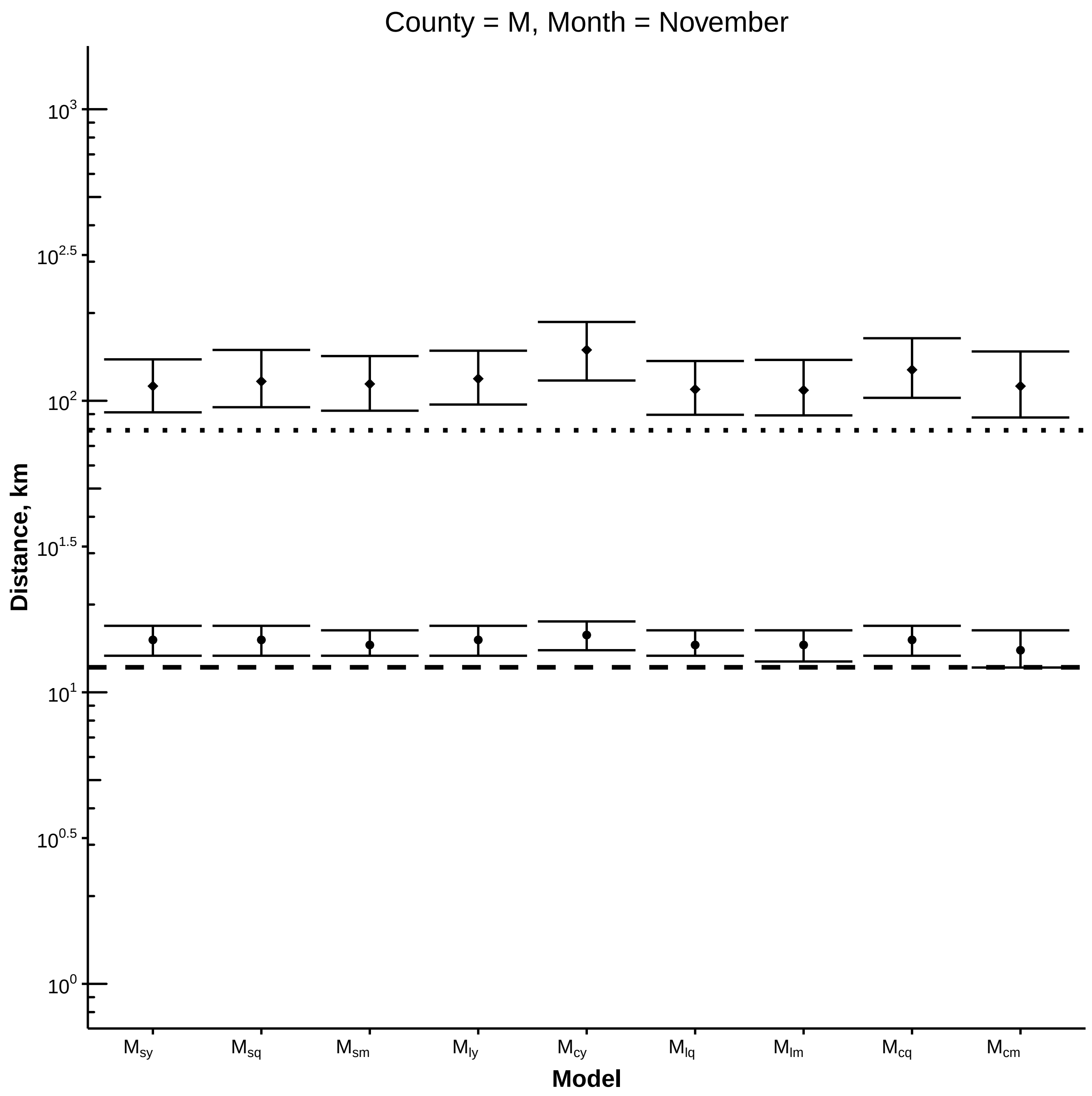
County = M, Month = November
Distance, km
10^3
$10^{2.5}$
10^2
$10^{1.5}$
10^1
$10^{0.5}$
10^0
M_{sy}
M_{sq}
M_{sm}
M_{ly}
M_{cy}
M_{lq}
M_{lm}
M_{cq}
M_{cm}
Model

County = M, Month = December

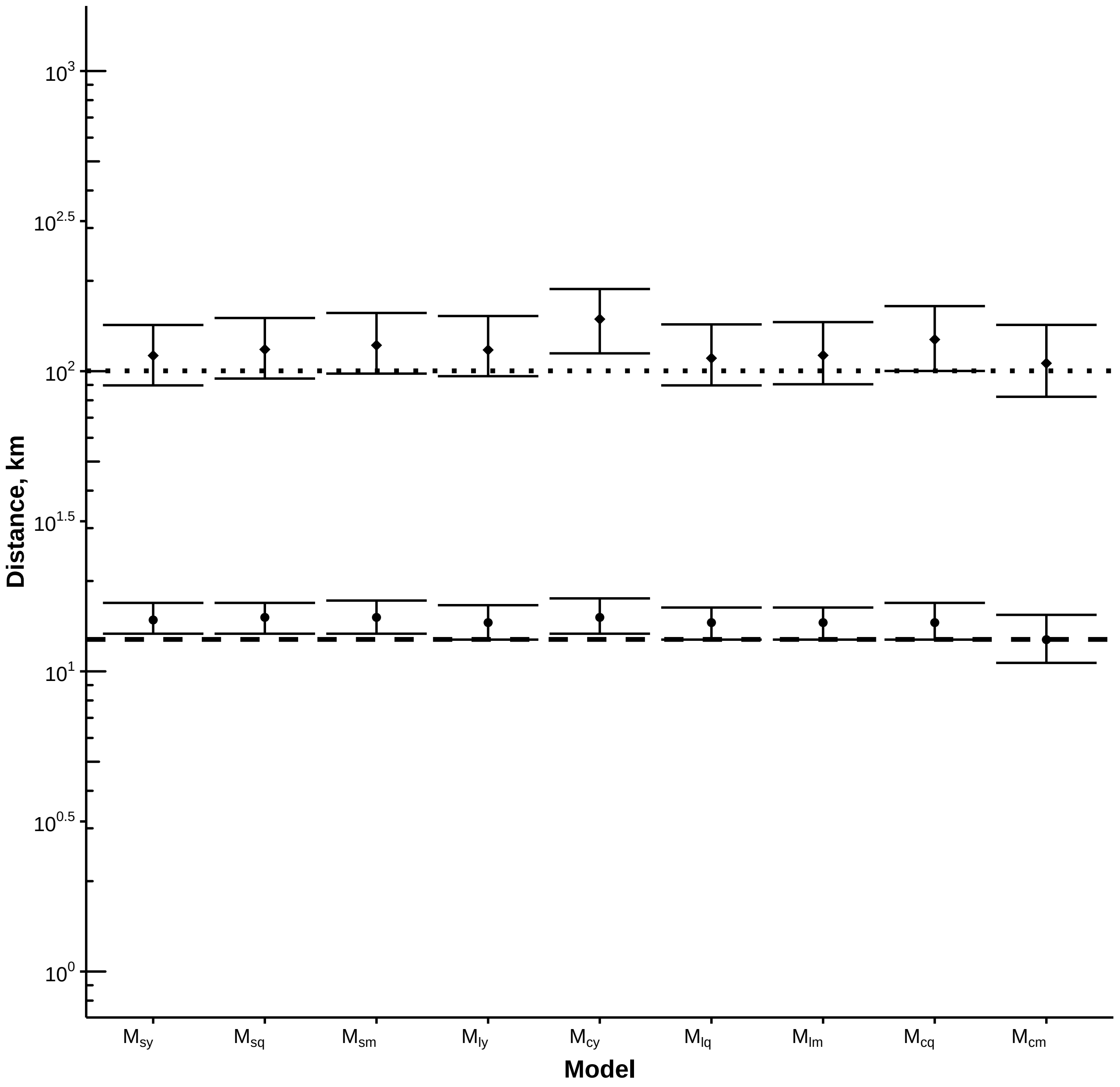

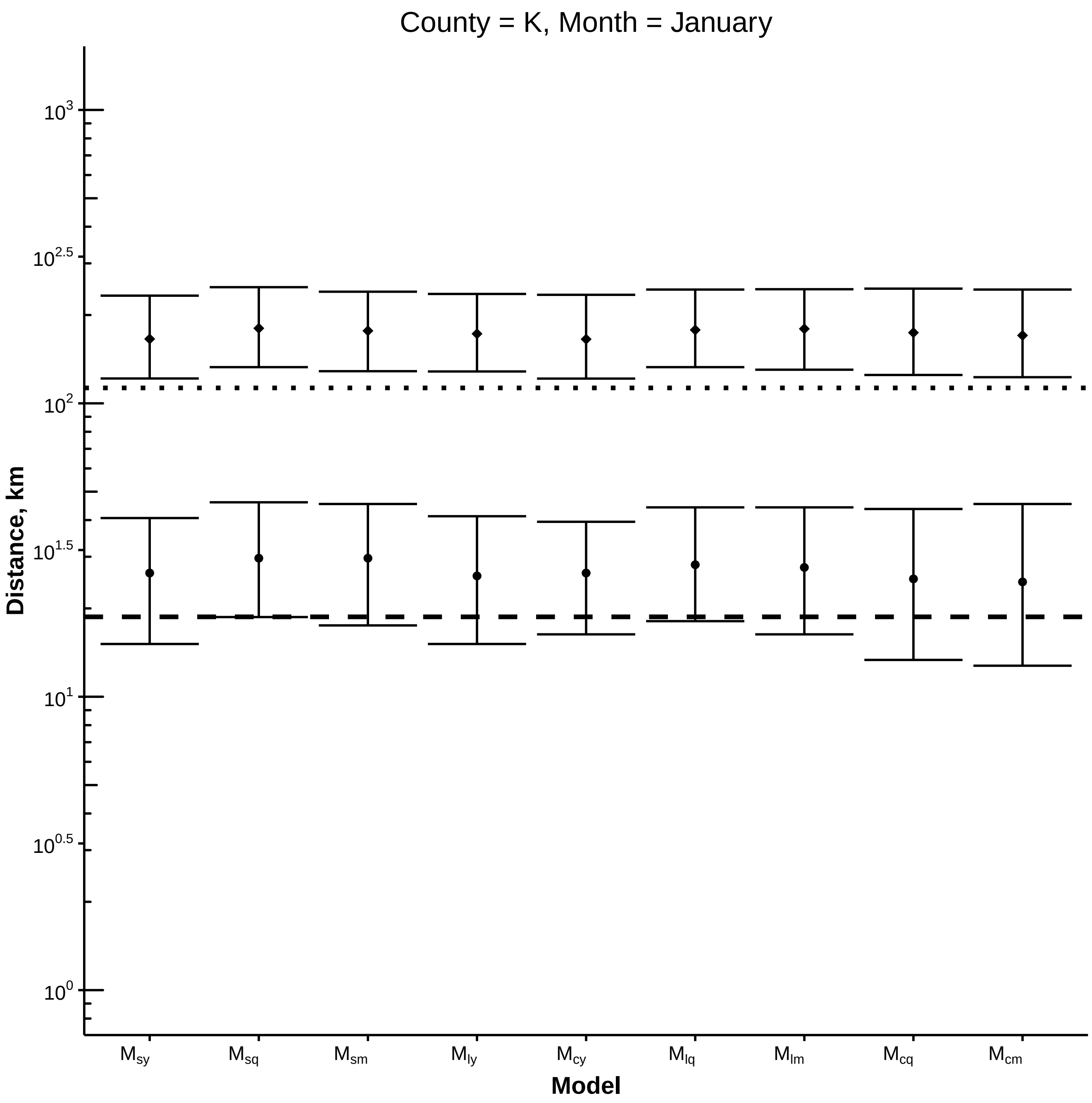

County = K, Month = January
Distance, km
10^3
$10^{2.5}$
10^2
$10^{1.5}$
10^1
$10^{0.5}$
10^0
M_{sy}
M_{sq}
M_{sm}
M_{ly}
M_{cy}
M_{lq}
M_{lm}
M_{cq}
M_{cm}
Model

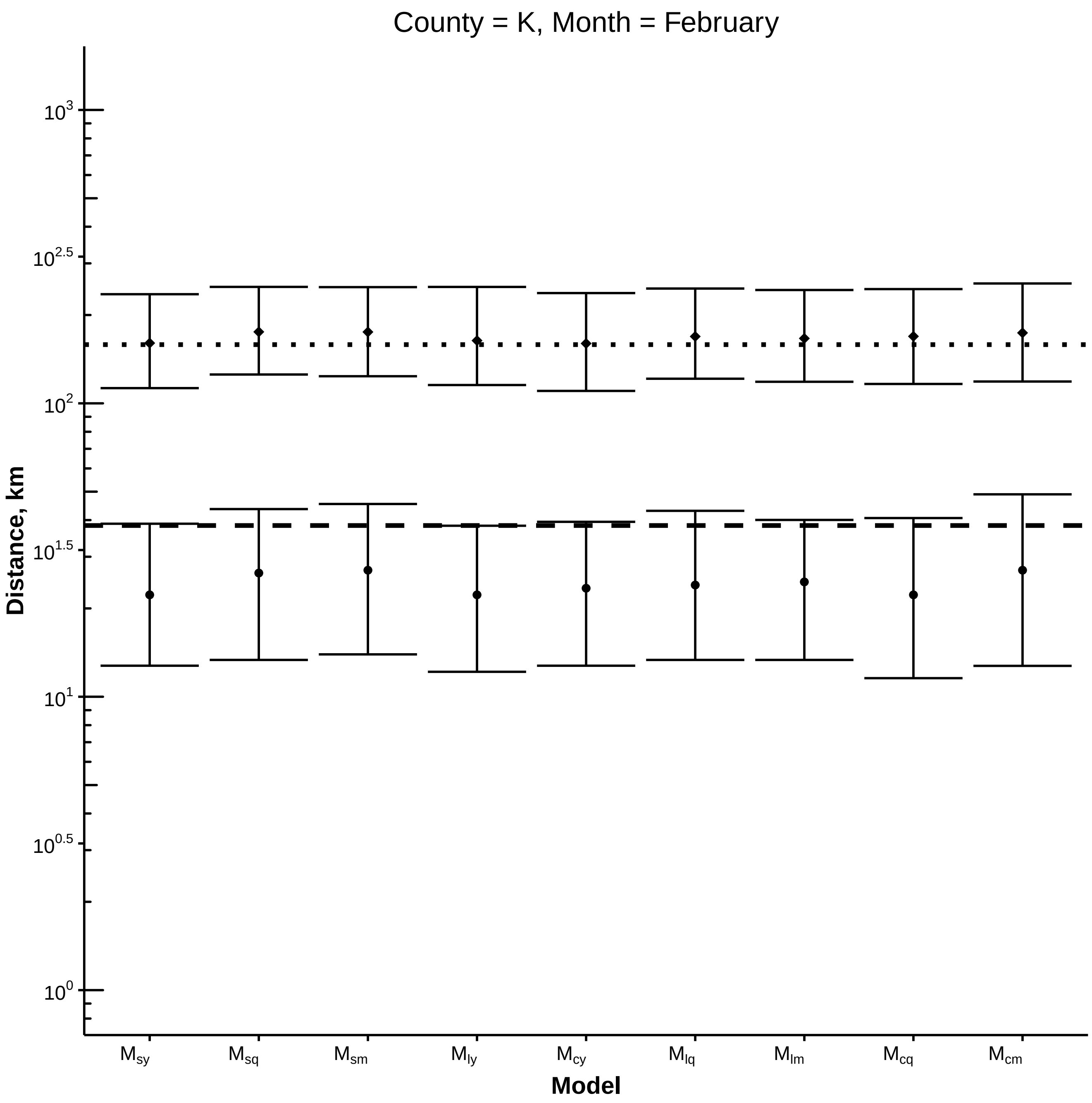

County = K, Month = February
Distance, km
10^3
$10^{2.5}$
10^2
$10^{1.5}$
10^1
$10^{0.5}$
10^0
M_{sy}
M_{sq}
M_{sm}
M_{ly}
M_{cy}
M_{lq}
M_{lm}
M_{cq}
M_{cm}
Model

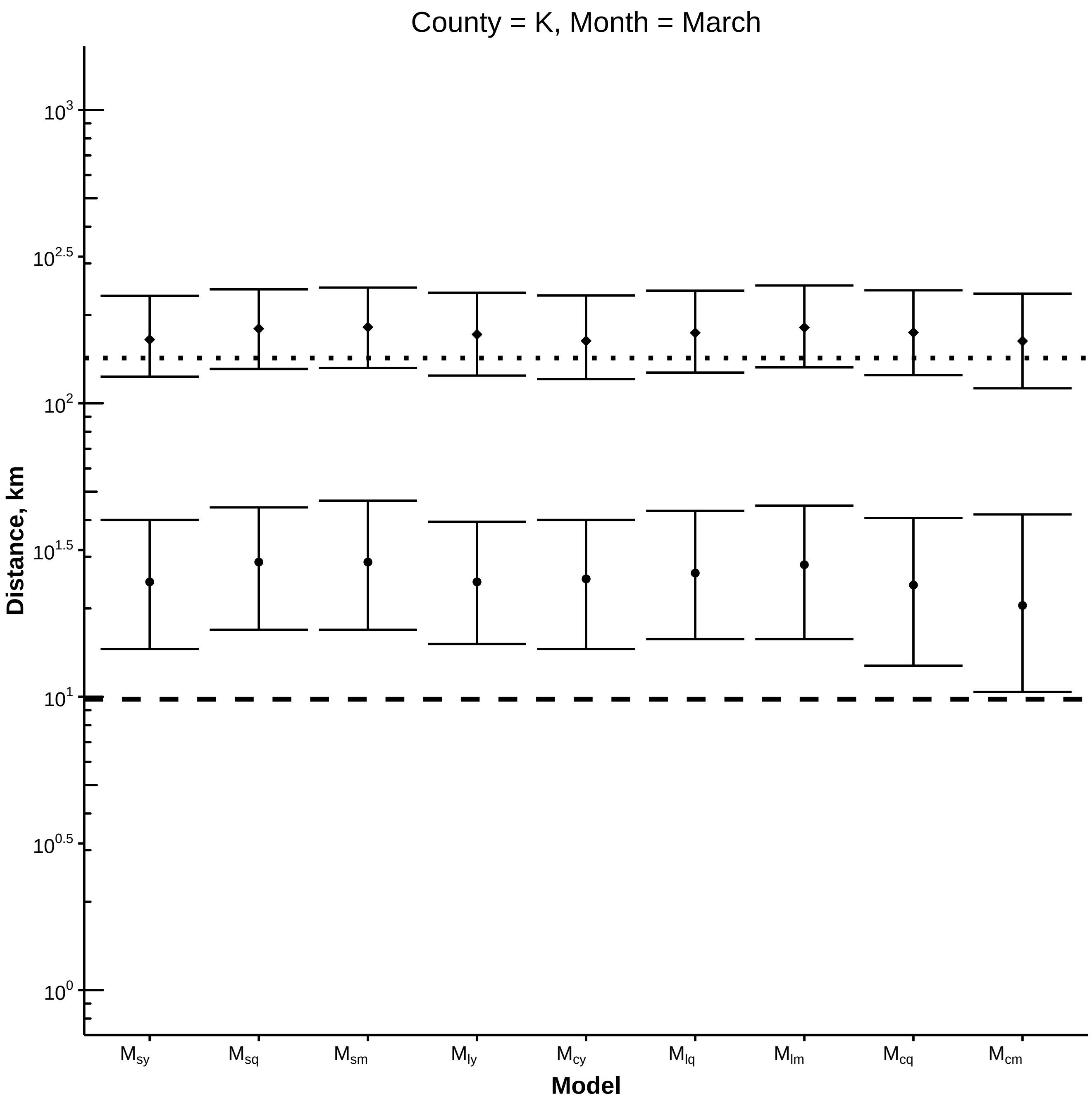

County = K, Month = March
Distance, km
10^3
$10^{2.5}$
10^2
$10^{1.5}$
10^1
$10^{0.5}$
10^0
M_{sy}
M_{sq}
M_{sm}
M_{ly}
M_{cy}
M_{lq}
M_{lm}
M_{cq}
M_{cm}
Model

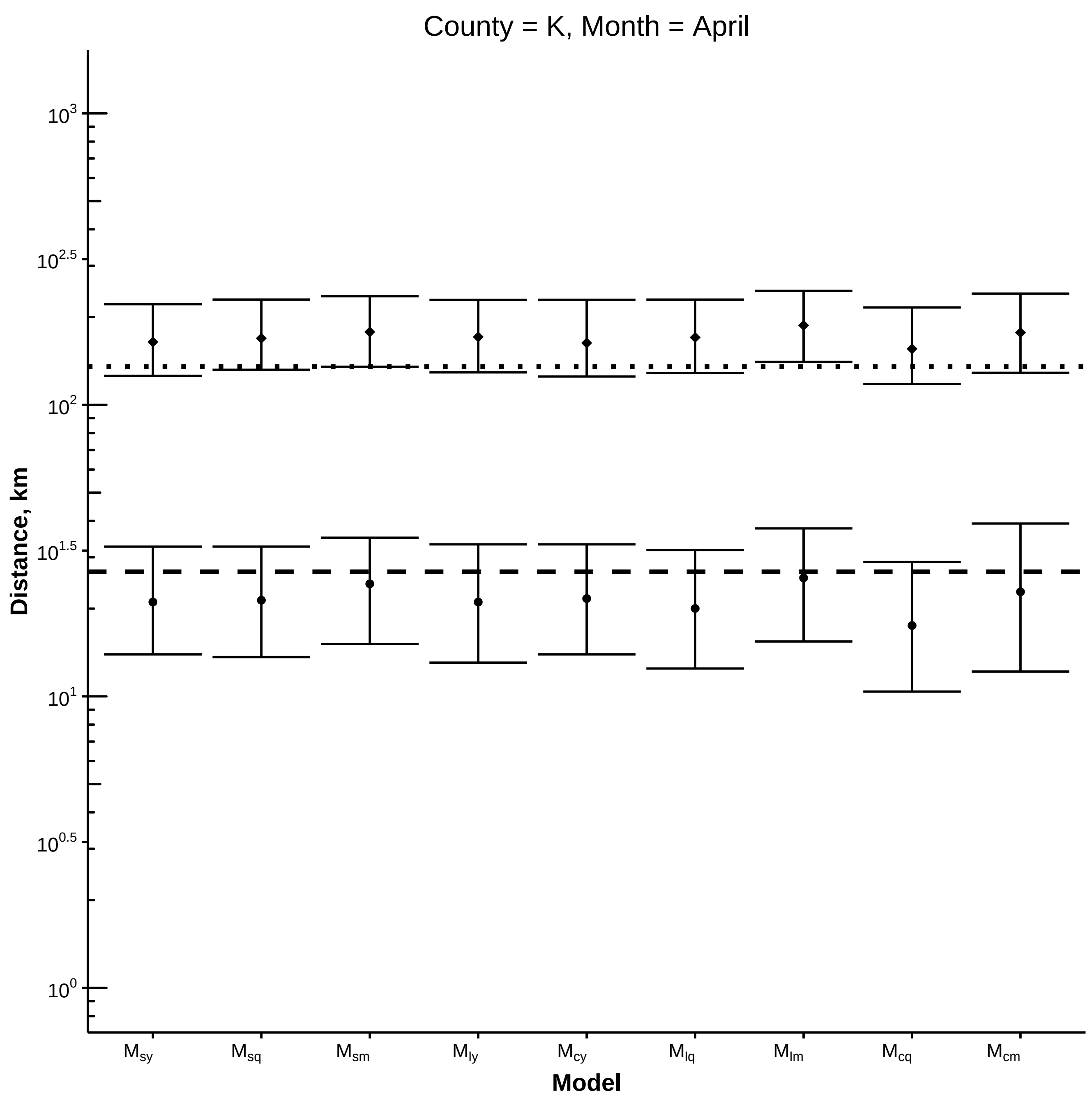

County = K, Month = April
Distance, km
10^3
$10^{2.5}$
10^2
$10^{1.5}$
10^1
$10^{0.5}$
10^0
M_{sy}
M_{sq}
M_{sm}
M_{ly}
M_{cy}
M_{lq}
M_{lm}
M_{cq}
M_{cm}
Model

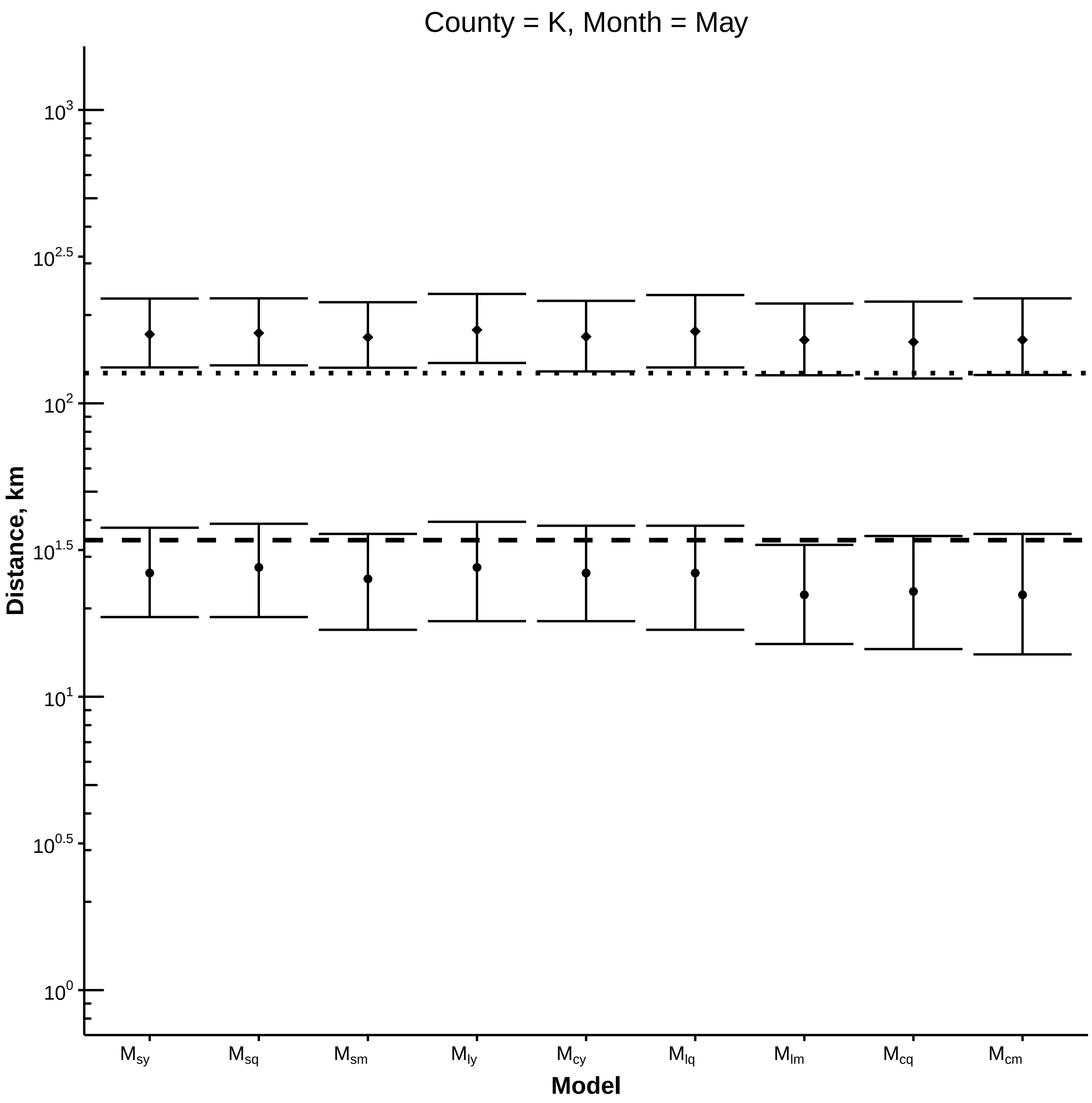

County = K, Month = May
Distance, km
10^3
$10^{2.5}$
10^2
$10^{1.5}$
10^1
$10^{0.5}$
10^0
M_{sy}
M_{sq}
M_{sm}
M_{ly}
M_{cy}
M_{lq}
M_{lm}
M_{cq}
M_{cm}
Model

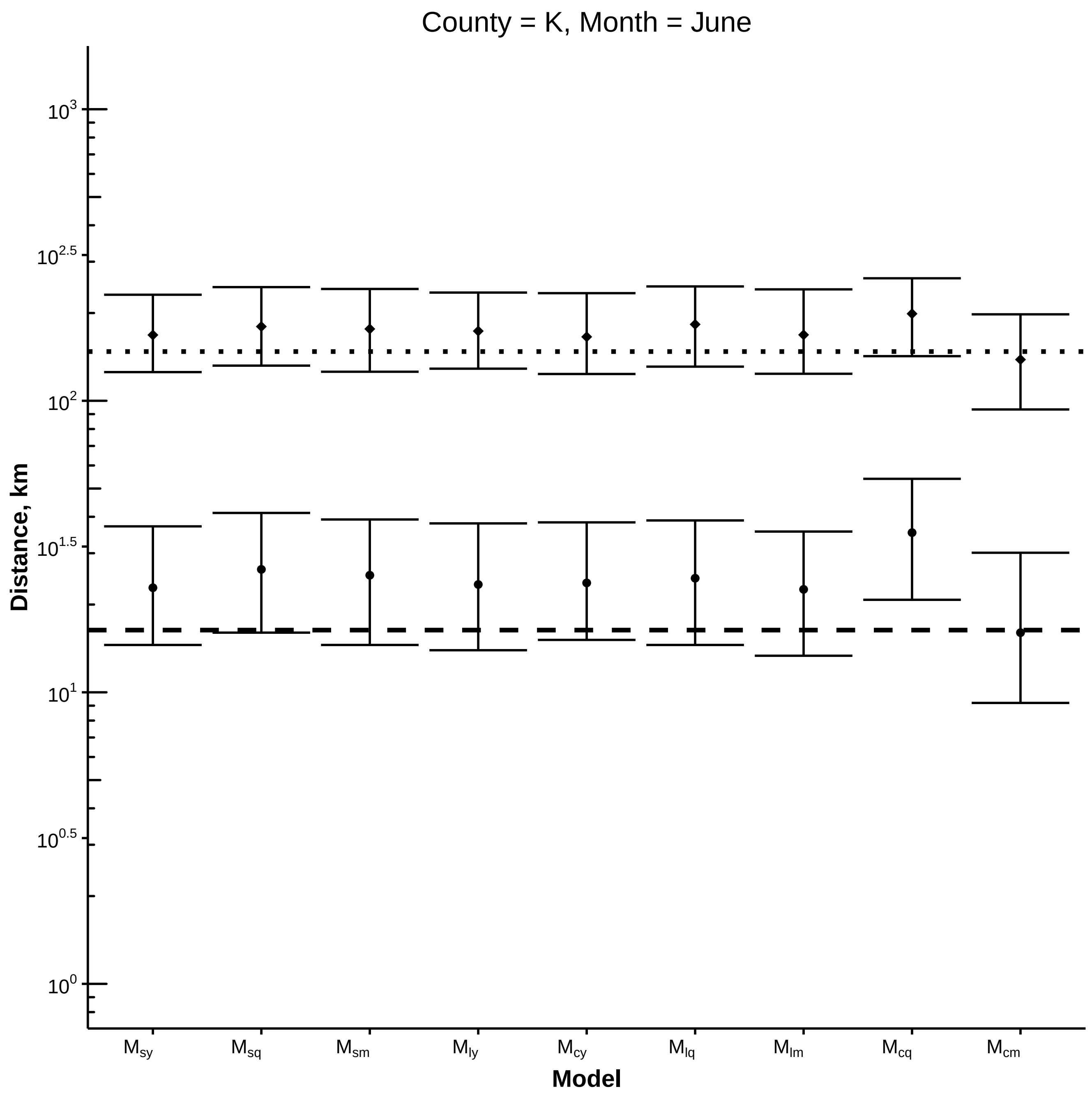
County = K, Month = June
Distance, km
10^3
$10^{2.5}$
10^2
$10^{1.5}$
10^1
$10^{0.5}$
10^0
M_{sy}
M_{sq}
M_{sm}
M_{ly}
M_{cy}
M_{lq}
M_{lm}
M_{cq}
M_{cm}
Model

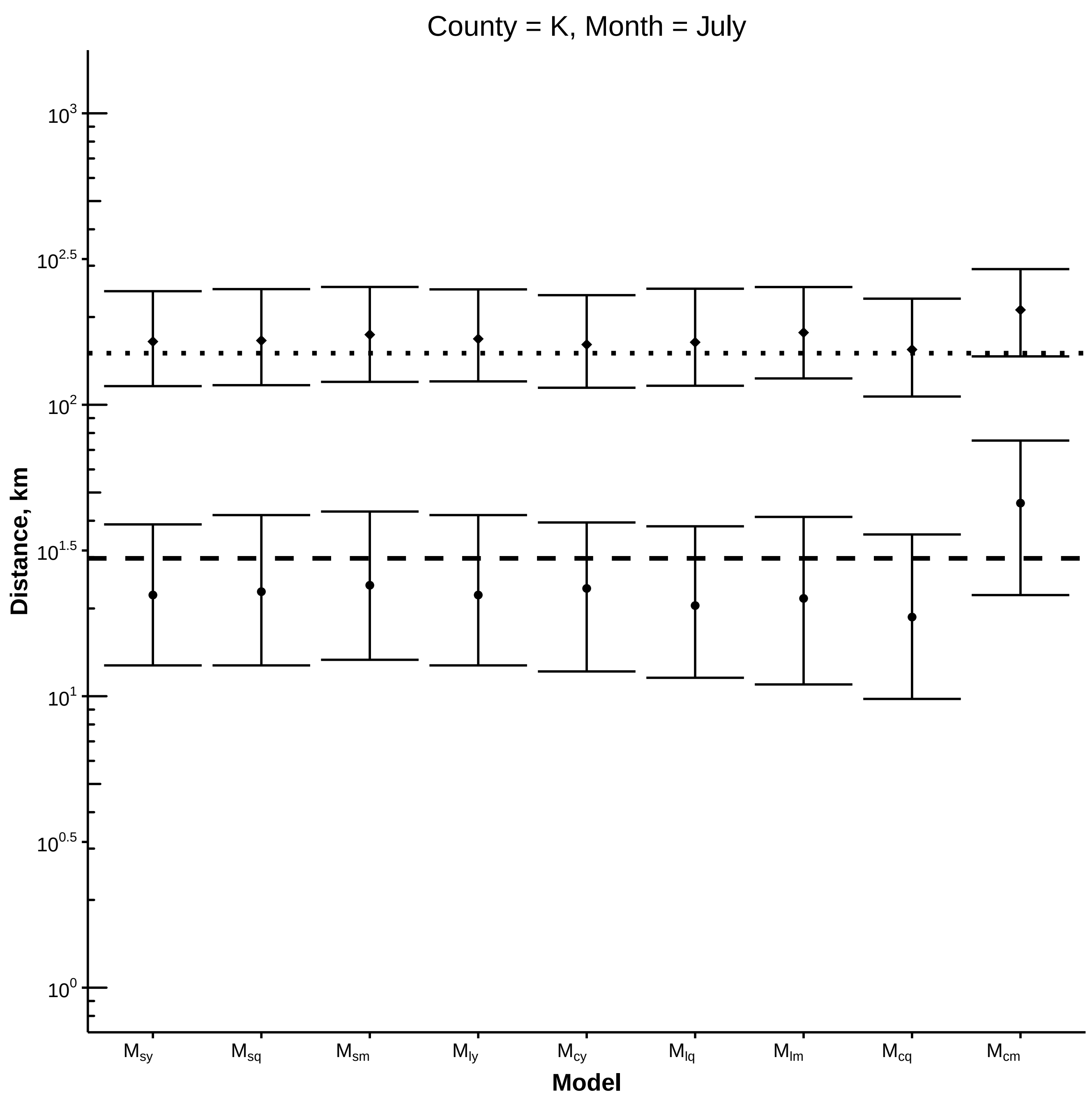
County = K, Month = July
10^3
$10^{2.5}$
10^2
$10^{1.5}$
10^1
$10^{0.5}$
10^0
Distance, km
M_{sy}
M_{sq}
M_{sm}
M_{ly}
M_{cy}
M_{lq}
M_{lm}
M_{cq}
M_{cm}
Model

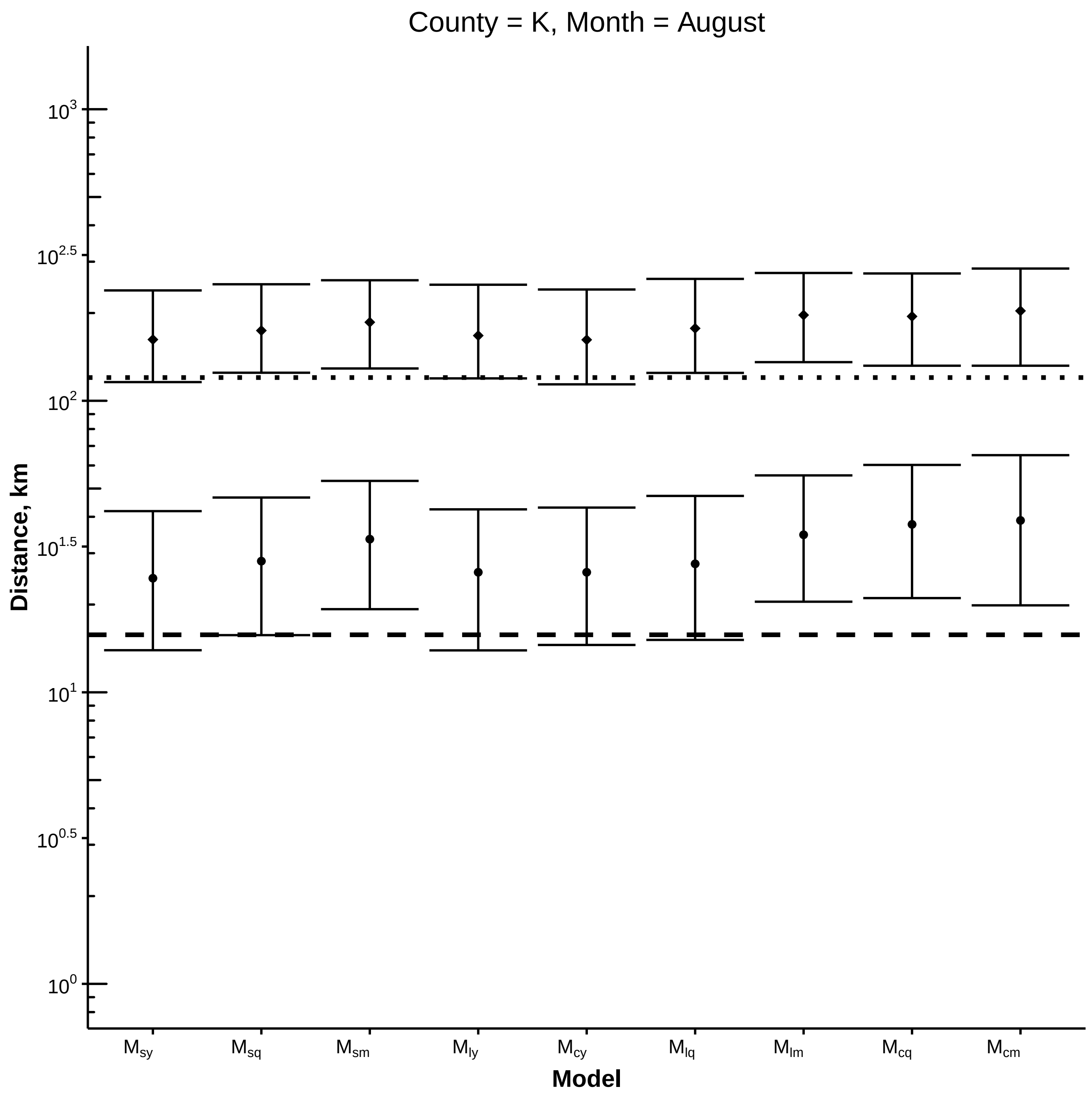
County = K, Month = August
Distance, km
10^3
$10^{2.5}$
10^2
$10^{1.5}$
10^1
$10^{0.5}$
10^0
M_{sy}
M_{sq}
M_{sm}
M_{ly}
M_{cy}
M_{lq}
M_{lm}
M_{cq}
M_{cm}
Model

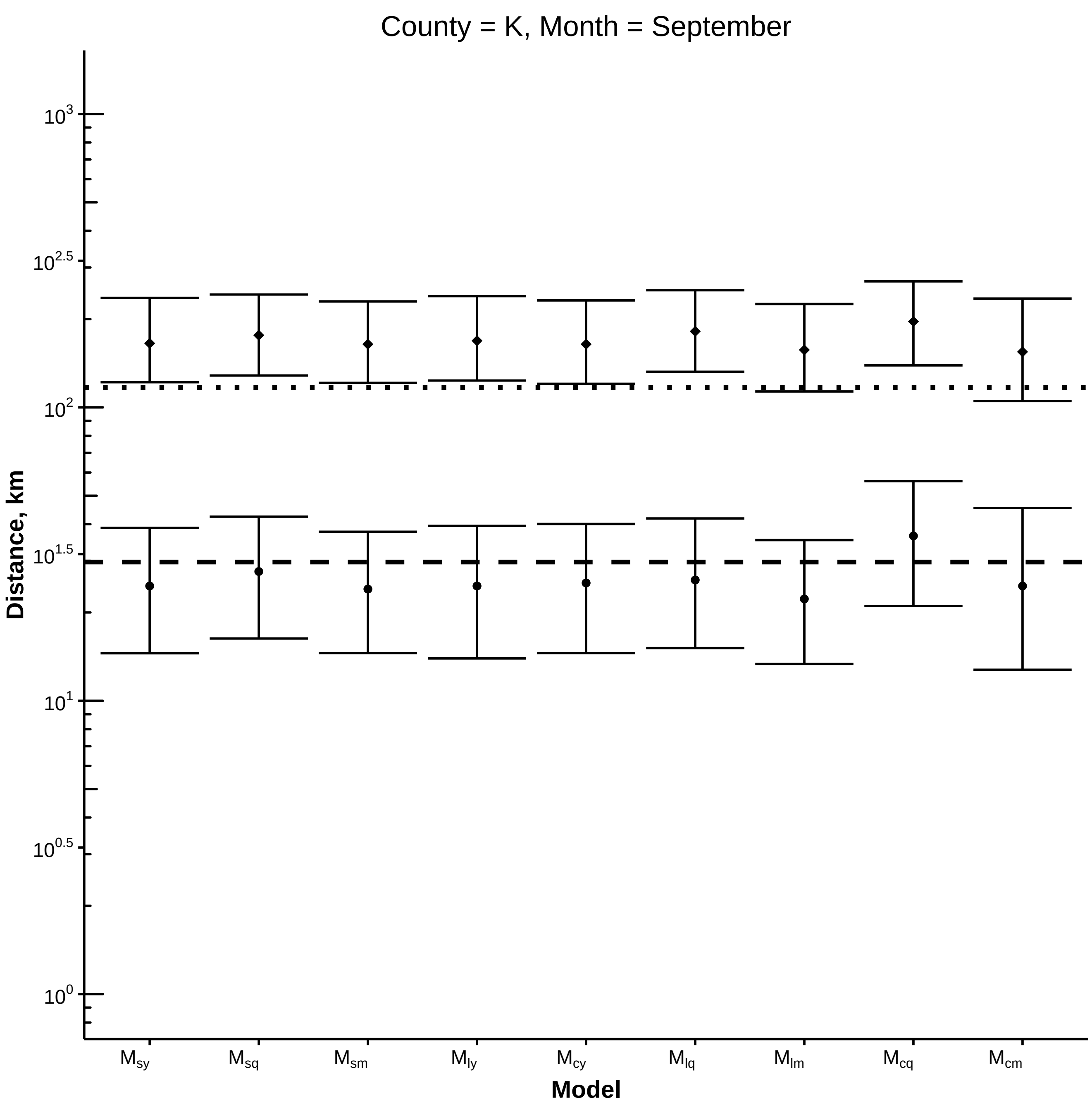
County = K, Month = September
Distance, km
10^3
$10^{2.5}$
10^2
$10^{1.5}$
10^1
$10^{0.5}$
10^0
M_{sy}
M_{sq}
M_{sm}
M_{ly}
M_{cy}
M_{lq}
M_{lm}
M_{cq}
M_{cm}
Model

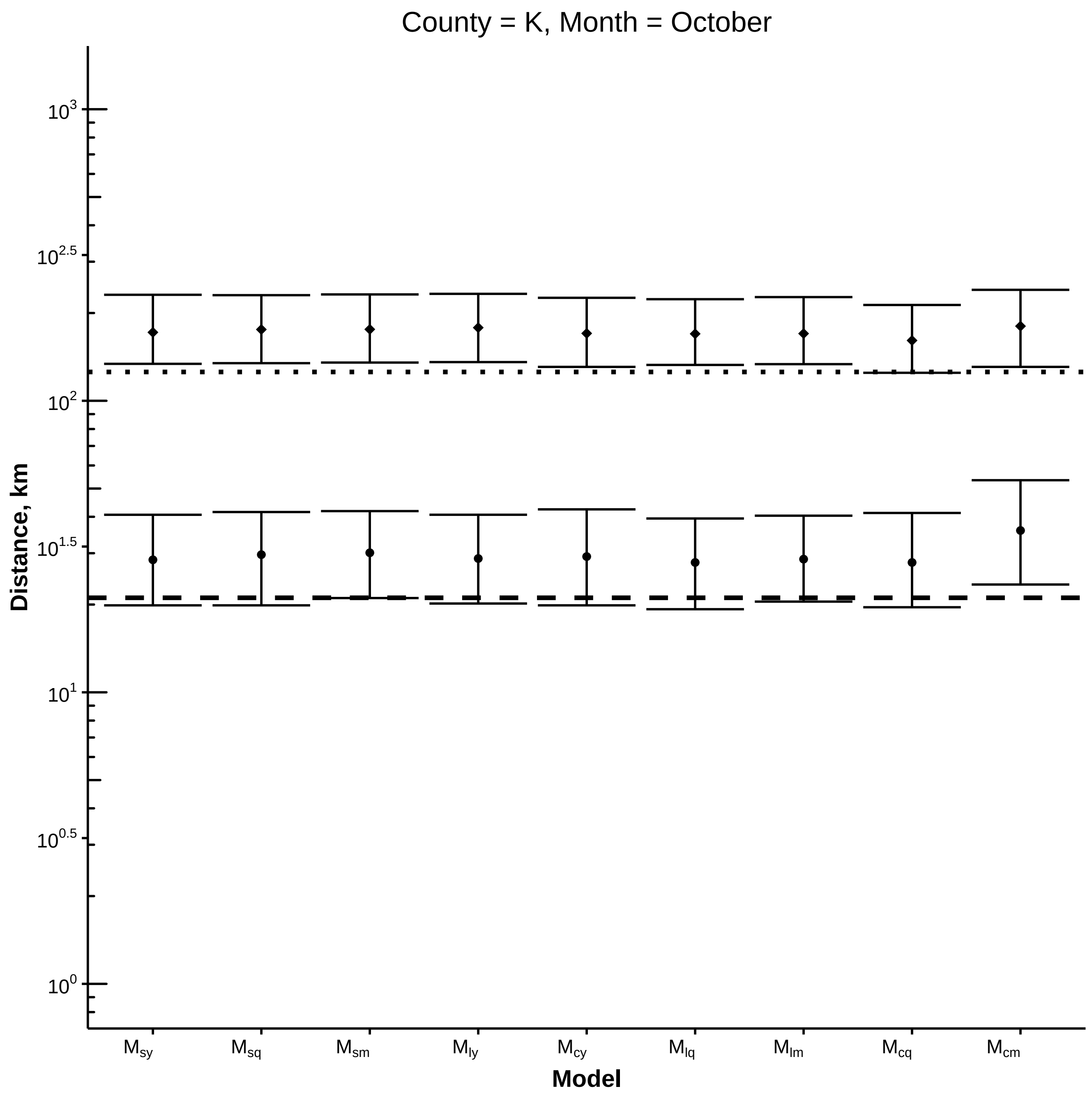

County = K, Month = October
10^3
$10^{2.5}$
10^2
$10^{1.5}$
10^1
$10^{0.5}$
10^0
Distance, km
M_{sy}
M_{sq}
M_{sm}
M_{ly}
M_{cy}
M_{lq}
M_{lm}
M_{cq}
M_{cm}
Model

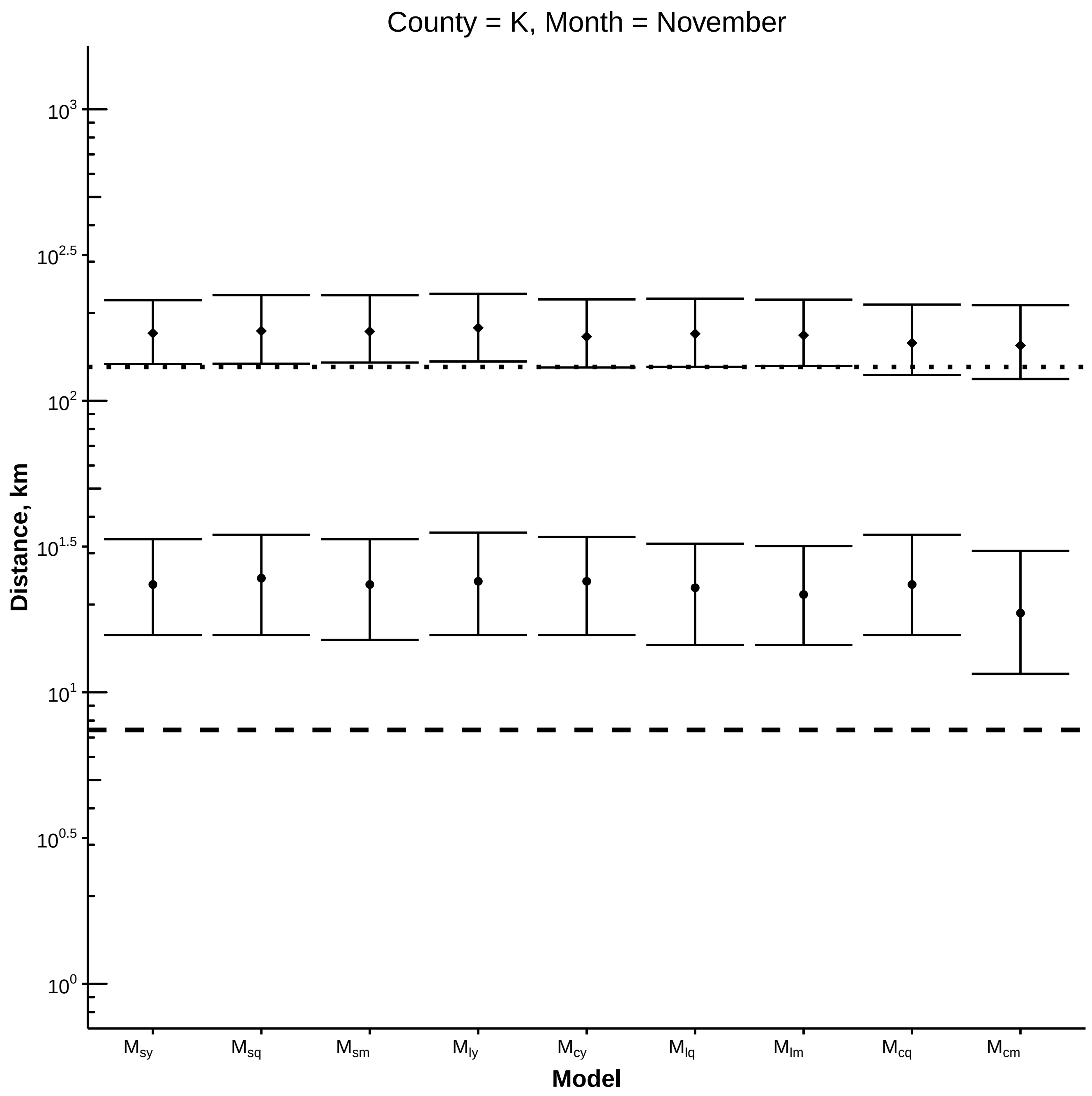

County = K, Month = November
Distance, km
10^3
$10^{2.5}$
10^2
$10^{1.5}$
10^1
$10^{0.5}$
10^0
M_{sy}
M_{sq}
M_{sm}
M_{ly}
M_{cy}
M_{lq}
M_{lm}
M_{cq}
M_{cm}
Model

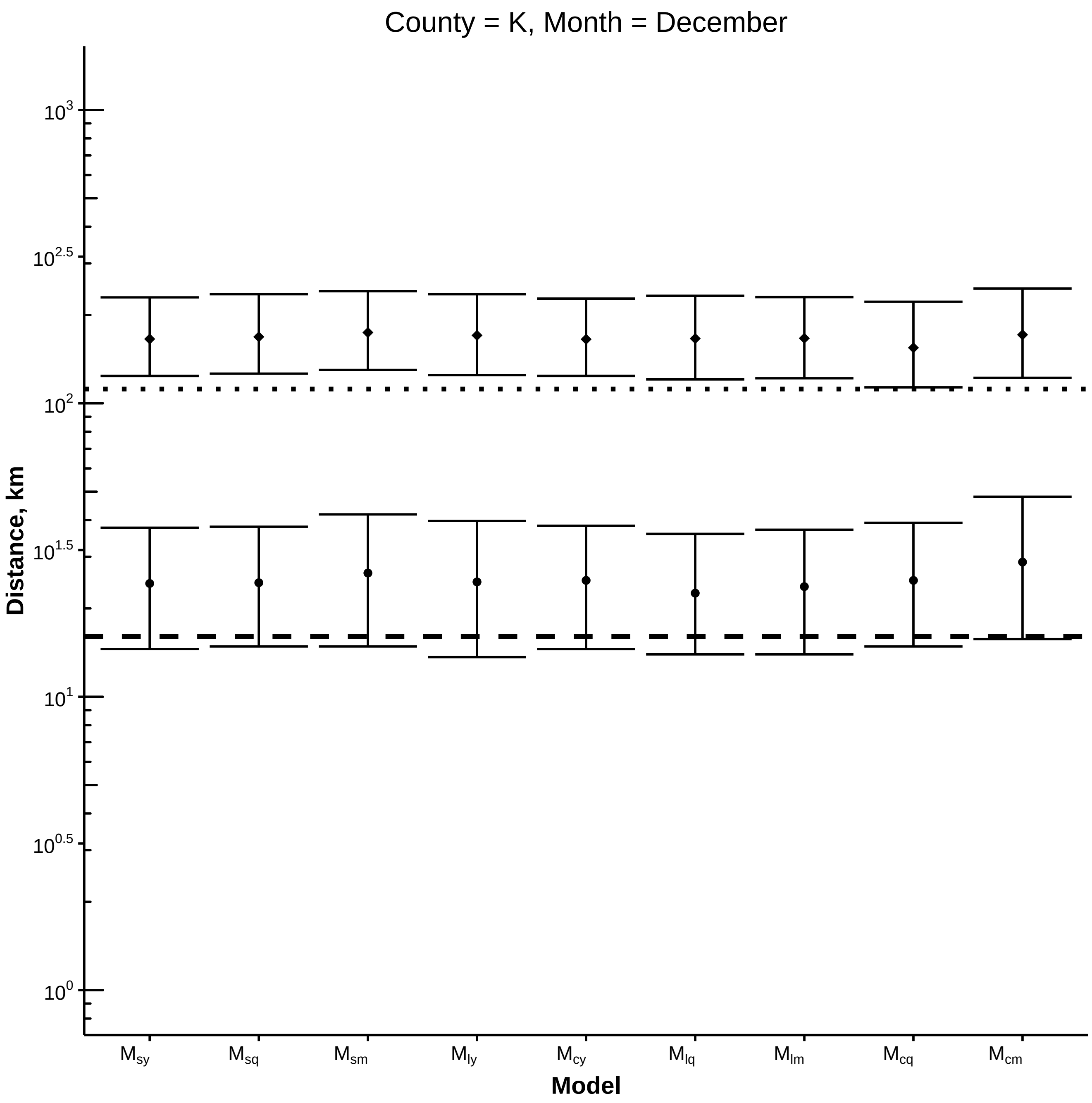

County = K, Month = December
Distance, km
10^3
$10^{2.5}$
10^2
$10^{1.5}$
10^1
$10^{0.5}$
10^0
M_{sy}
M_{sq}
M_{sm}
M_{ly}
M_{cy}
M_{lq}
M_{lm}
M_{cq}
M_{cm}
Model

County = N, Month = January

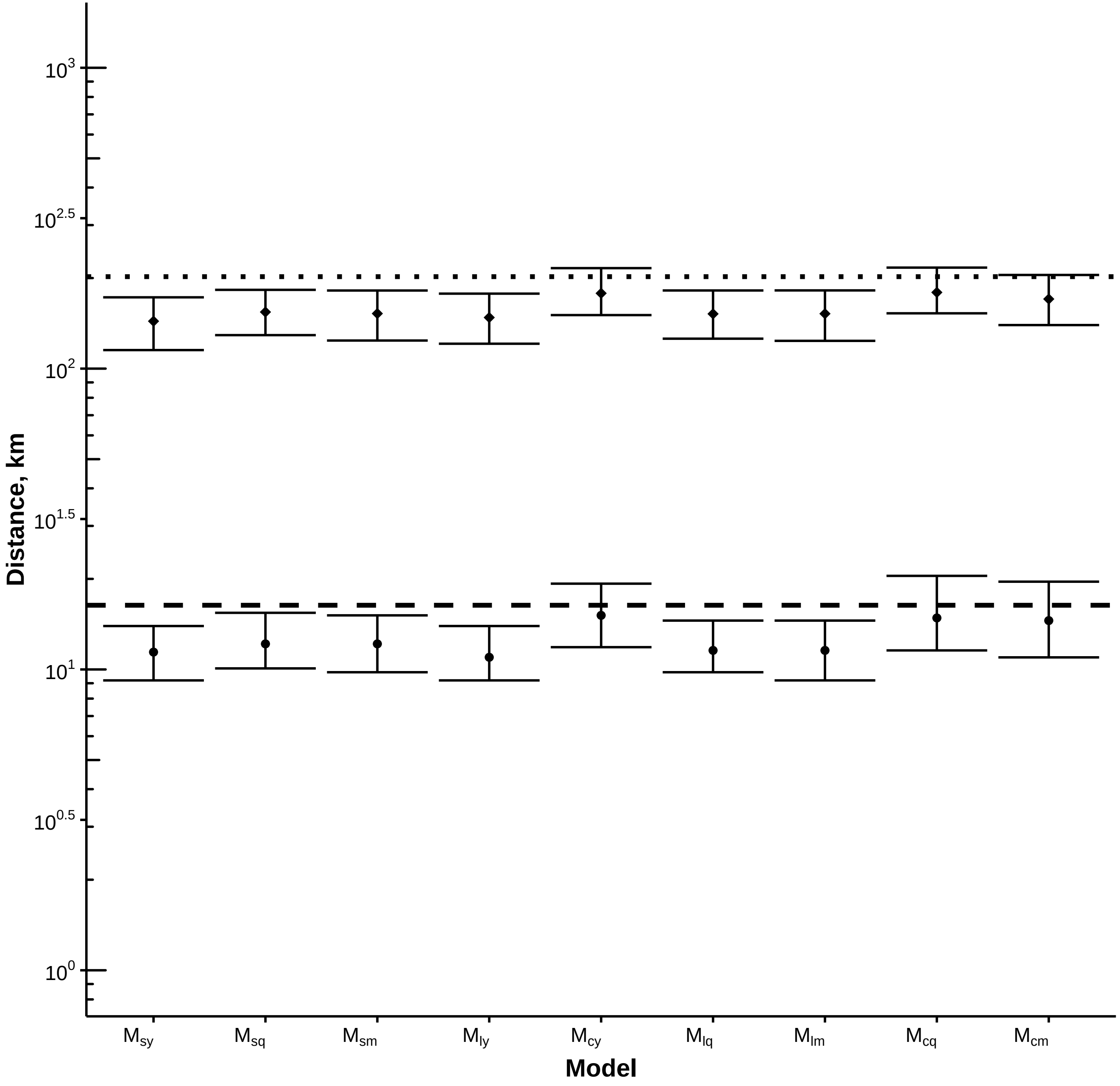

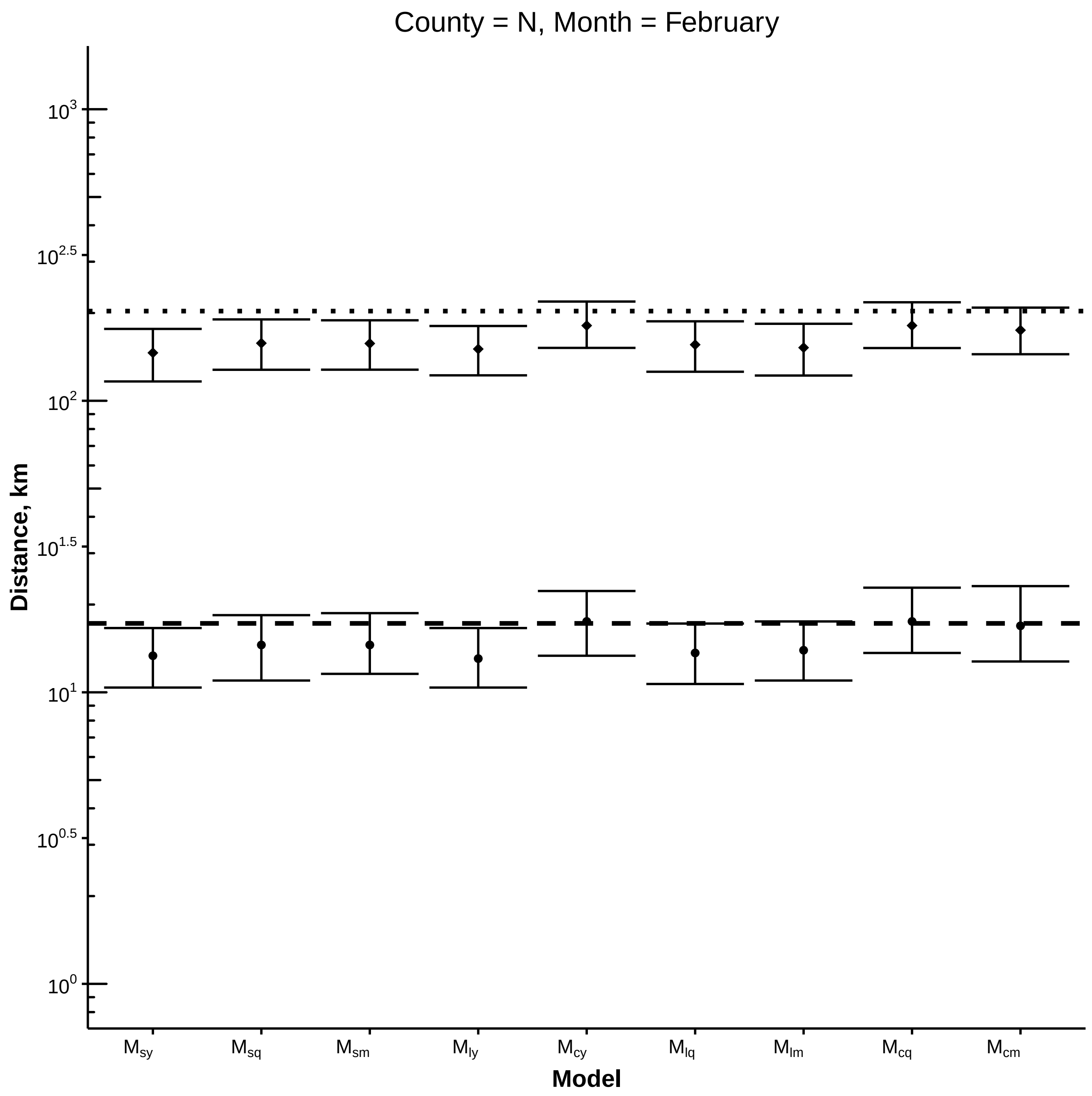
County = N, Month = February
Distance, km
10^3
10^2.5
10^2
10^1.5
10^1
10^0.5
10^0
M_sy
M_sq
M_sm
M_ly
M_cy
M_lq
M_lm
M_cq
M_cm
Model

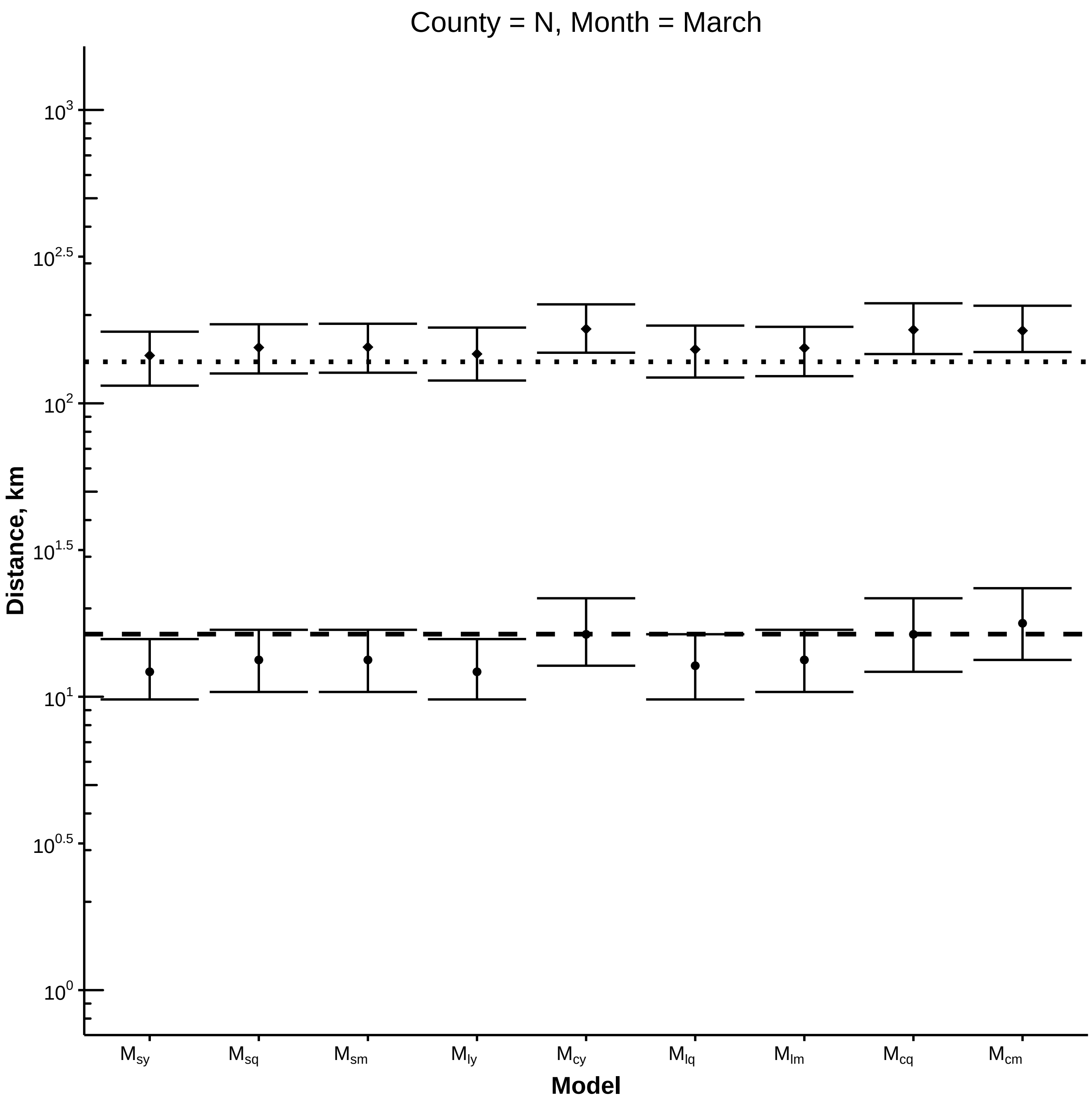
County = N, Month = March
Distance, km
10^3
$10^{2.5}$
10^2
$10^{1.5}$
10^1
$10^{0.5}$
10^0
M_{sy}
M_{sq}
M_{sm}
M_{ly}
M_{cy}
M_{lq}
M_{lm}
M_{cq}
M_{cm}
Model

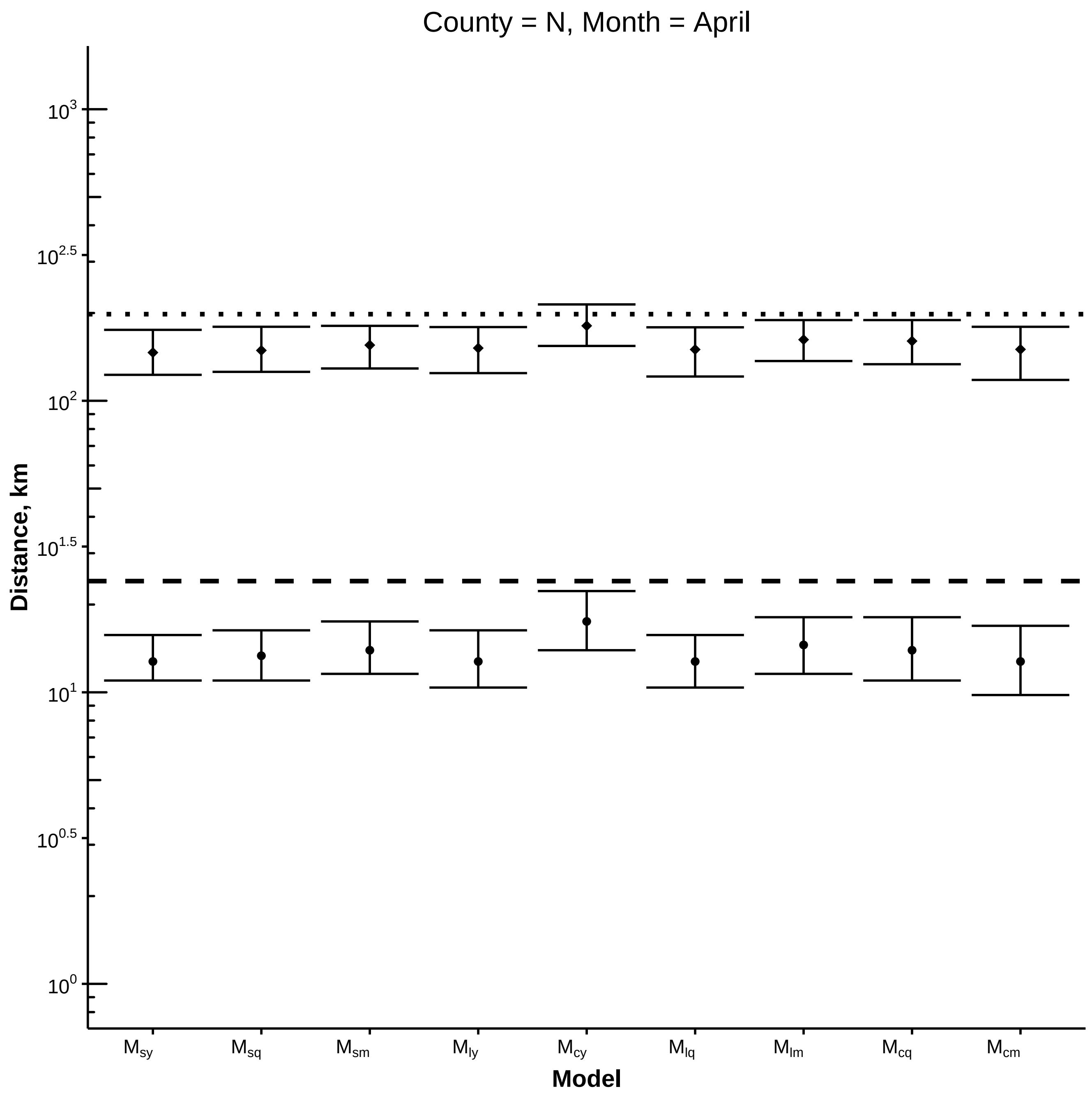
County = N, Month = April
Distance, km
10^3
$10^{2.5}$
10^2
$10^{1.5}$
10^1
$10^{0.5}$
10^0
M_{sy}
M_{sq}
M_{sm}
M_{ly}
M_{cy}
M_{lq}
M_{lm}
M_{cq}
M_{cm}
Model

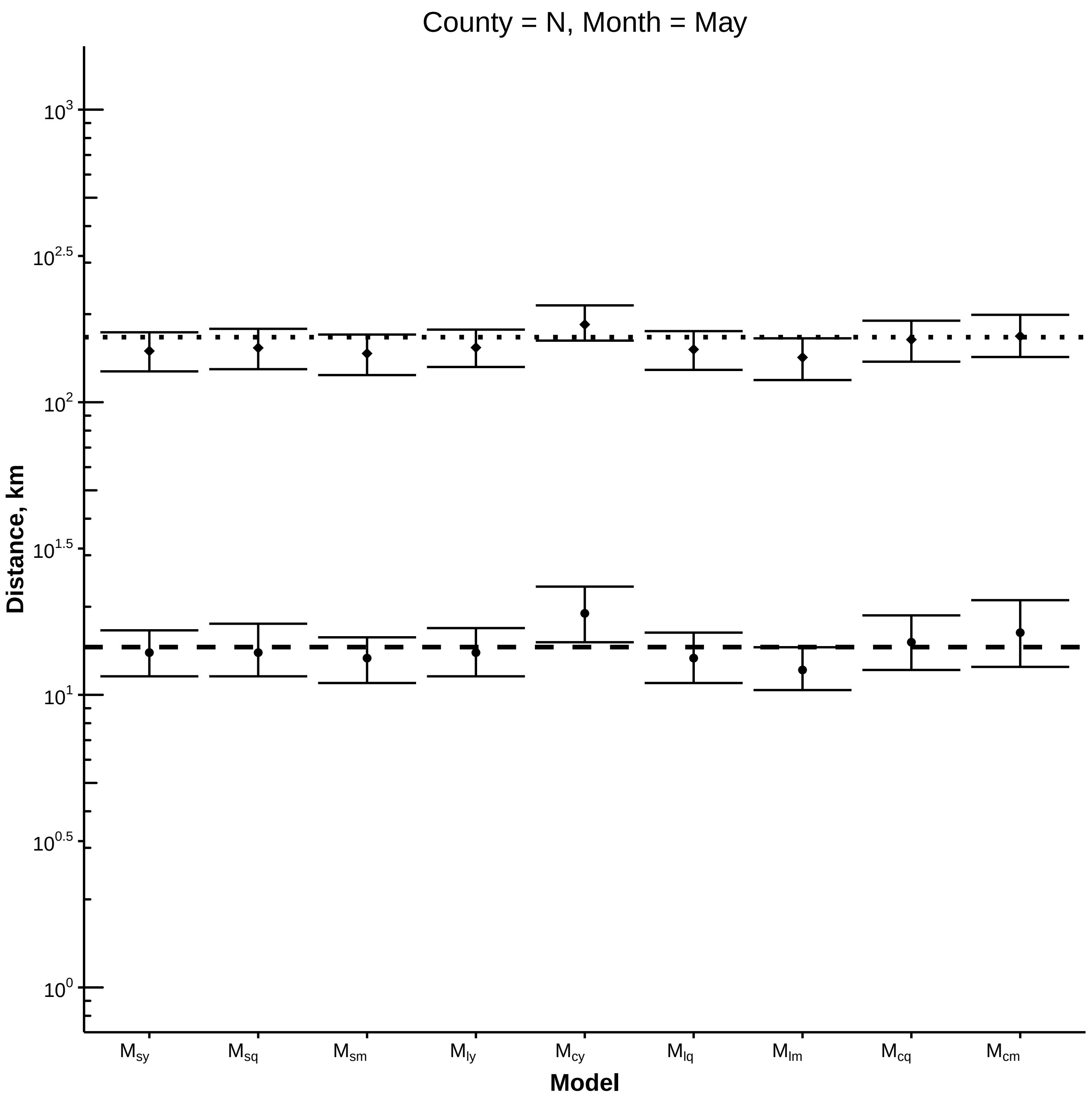

County = N, Month = May
Distance, km
10^3
$10^{2.5}$
10^2
$10^{1.5}$
10^1
$10^{0.5}$
10^0
M_{sy}
M_{sq}
M_{sm}
M_{ly}
M_{cy}
M_{lq}
M_{lm}
M_{cq}
M_{cm}
Model

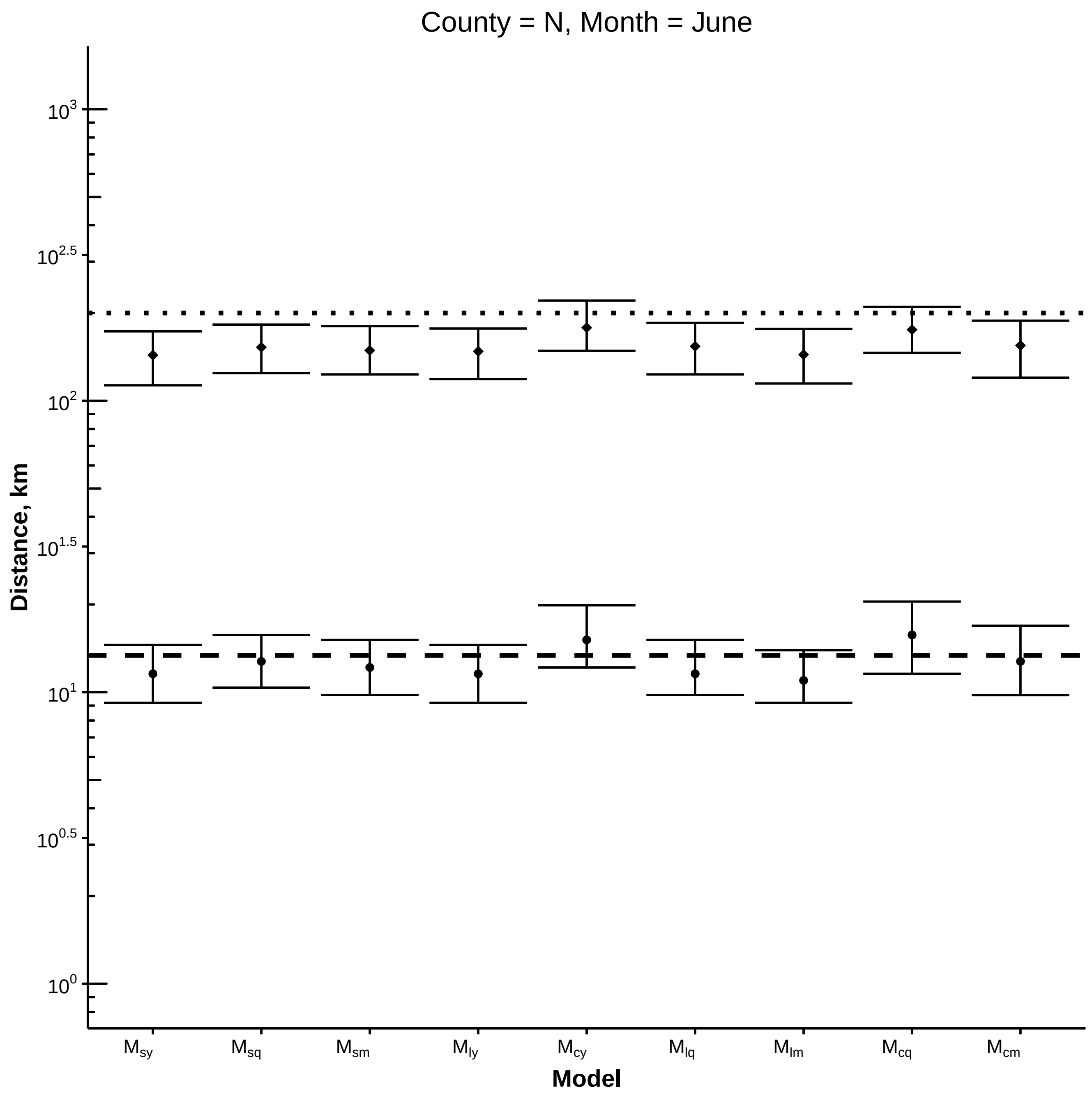

County = N, Month = June
Distance, km
10^3
10^2.5
10^2
10^1.5
10^1
10^0.5
10^0
Msy
Msq
Msm
Mly
Mcy
Mlq
Mlm
Mcq
Mcm
Model

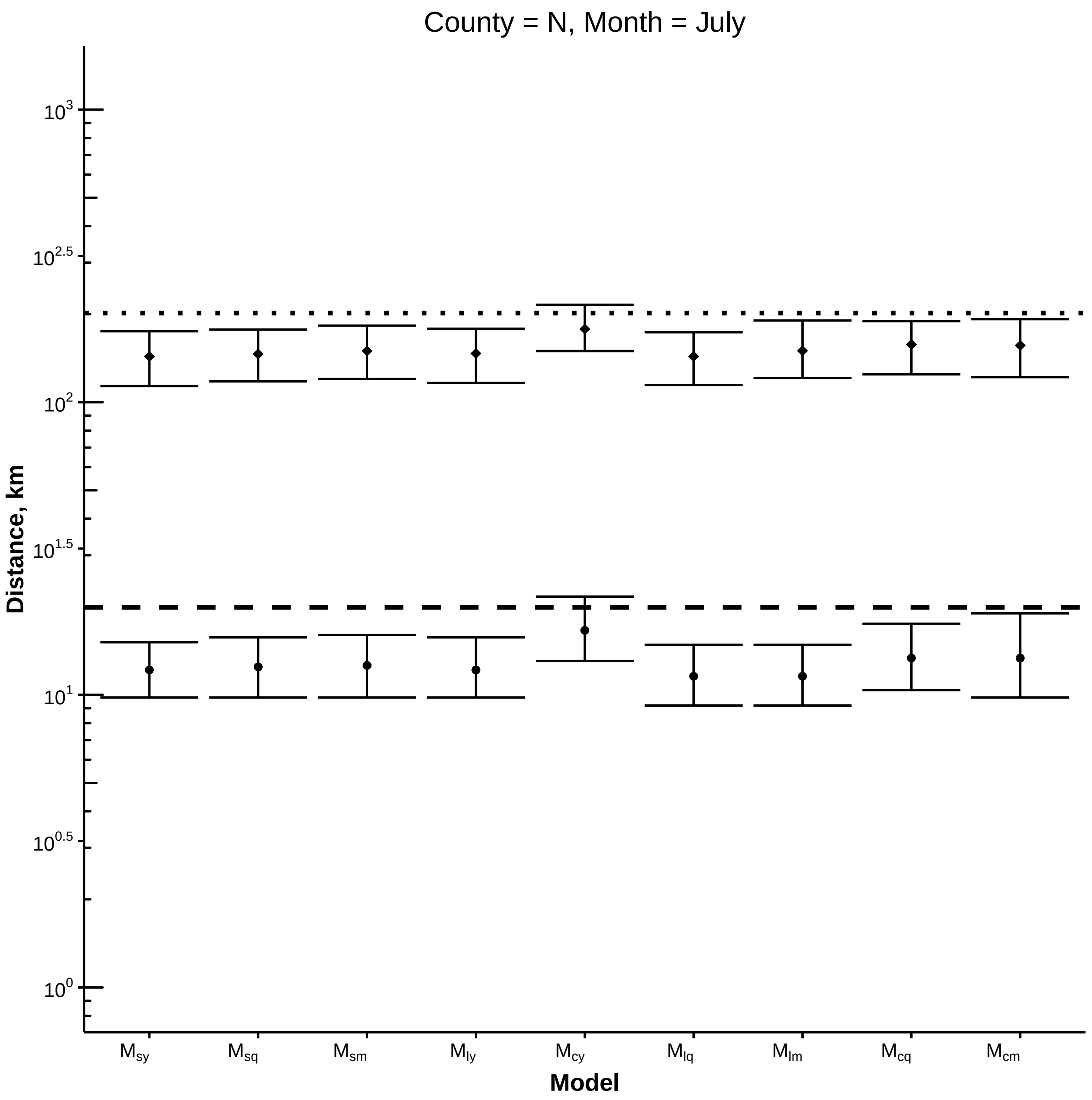
County = N, Month = July
Distance, km
10^{3}
$10^{2.5}$
10^{2}
$10^{1.5}$
10^{1}
$10^{0.5}$
10^{0}
M_{sy}
M_{sq}
M_{sm}
M_{ly}
M_{cy}
M_{lq}
M_{lm}
M_{cq}
M_{cm}
Model

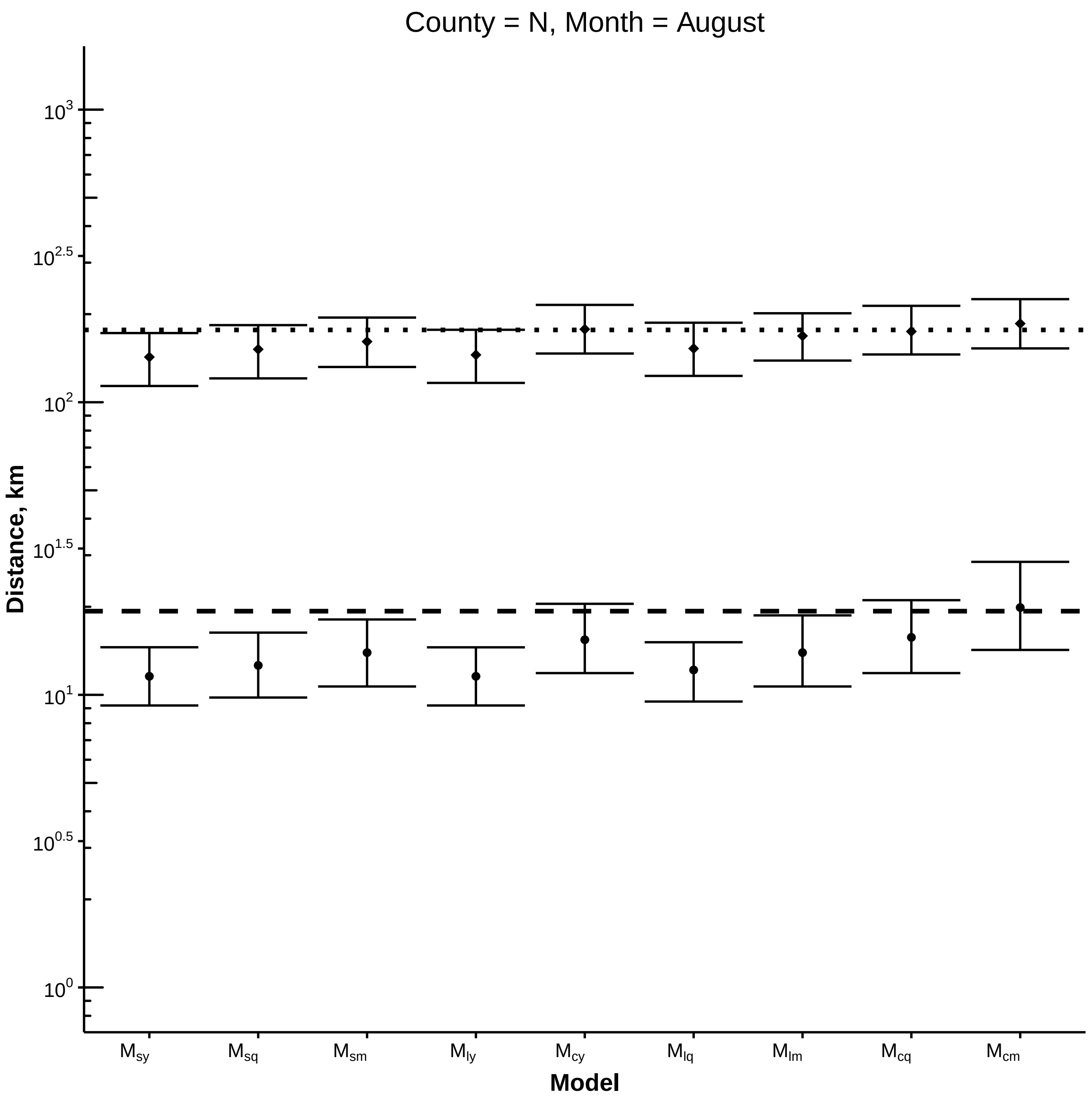

County = N, Month = August
Distance, km
10^3
$10^{2.5}$
10^2
$10^{1.5}$
10^1
$10^{0.5}$
10^0
M_{sy}
M_{sq}
M_{sm}
M_{ly}
M_{cy}
M_{lq}
M_{lm}
M_{cq}
M_{cm}
Model

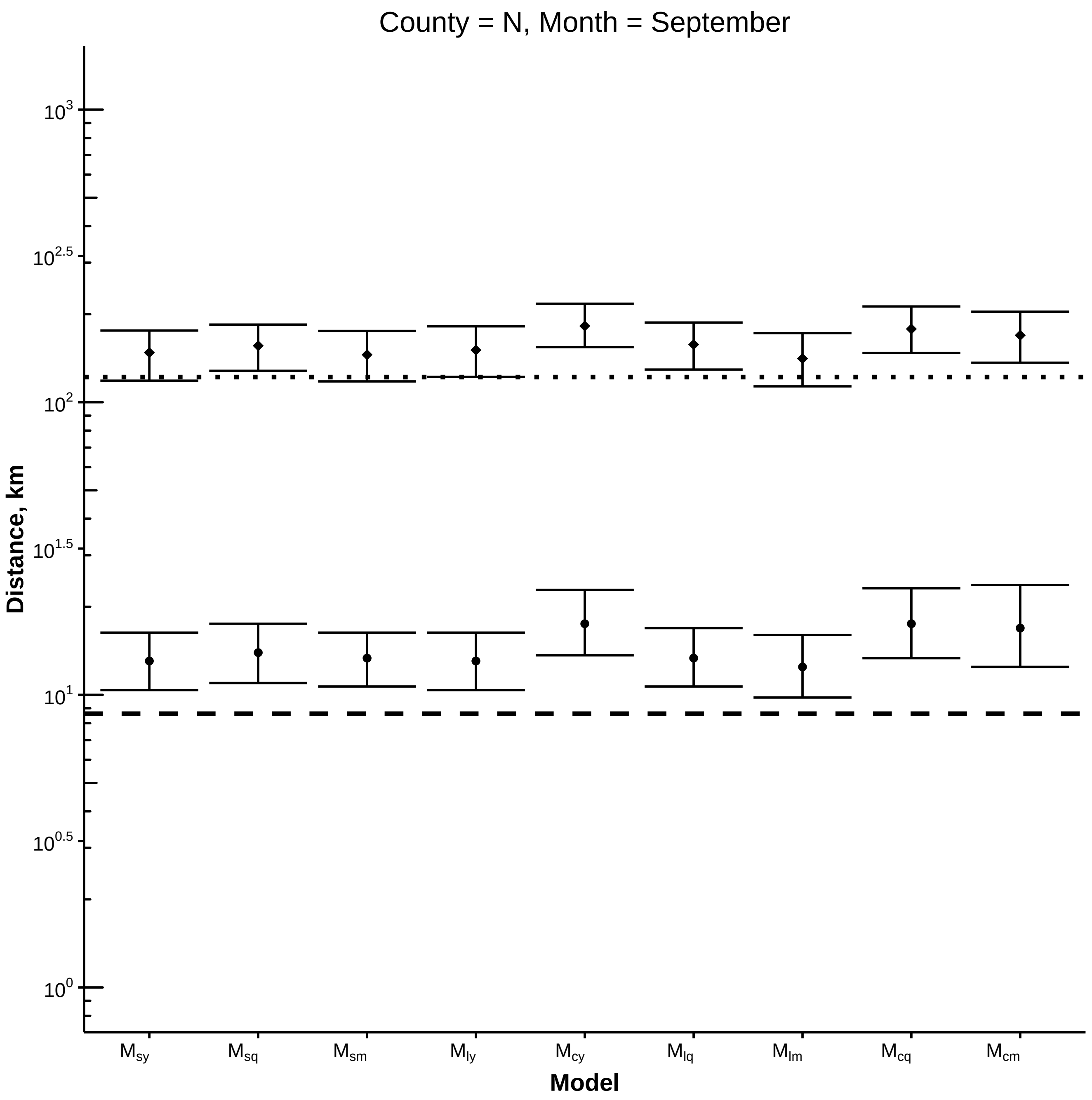

County = N, Month = September
Distance, km
10^3
$10^{2.5}$
10^2
$10^{1.5}$
10^1
$10^{0.5}$
10^0
M_{sy}
M_{sq}
M_{sm}
M_{ly}
M_{cy}
M_{lq}
M_{lm}
M_{cq}
M_{cm}
Model

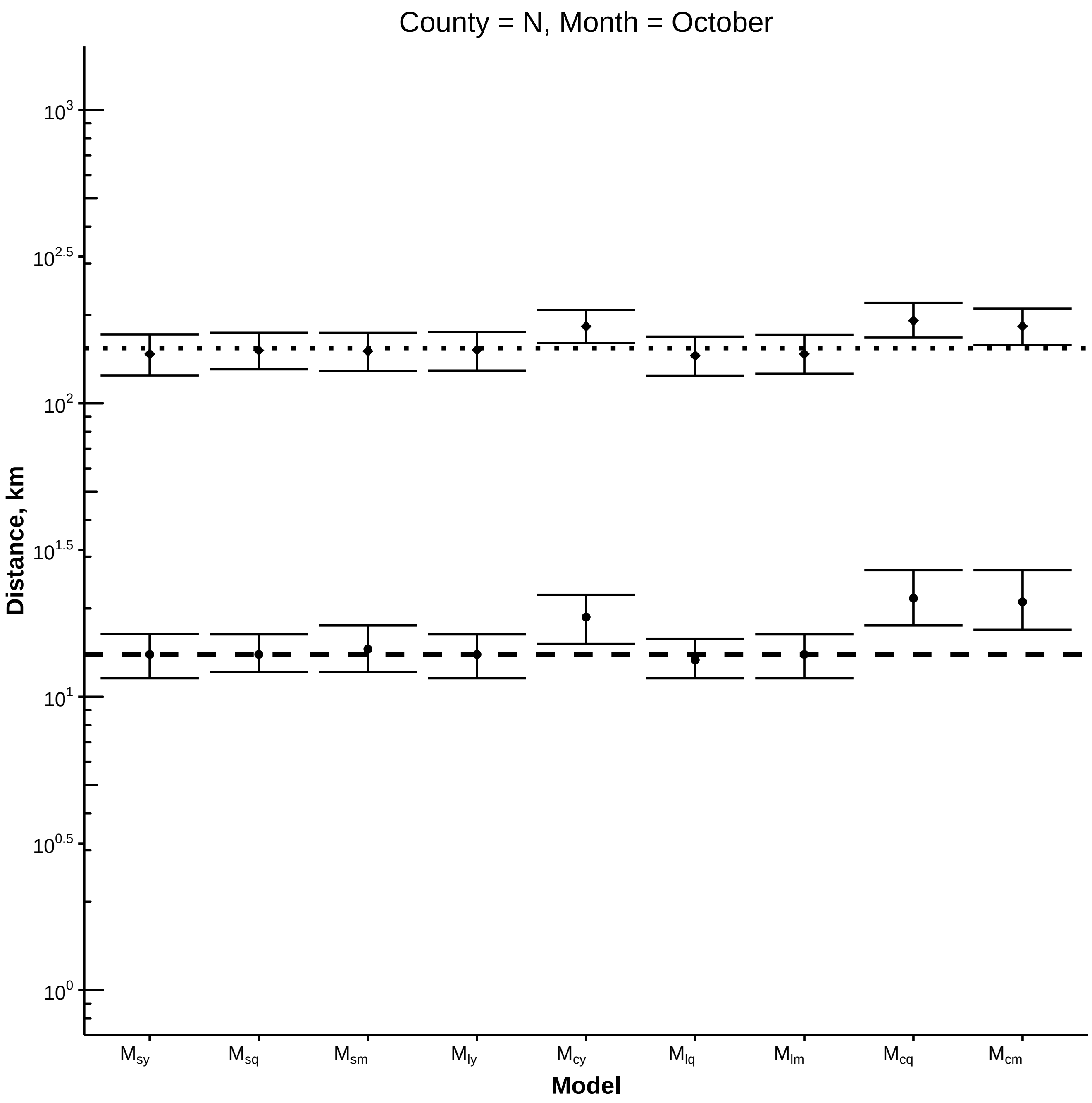

County = N, Month = October
Distance, km
10^3
$10^{2.5}$
10^2
$10^{1.5}$
10^1
$10^{0.5}$
10^0
M_{sy}
M_{sq}
M_{sm}
M_{ly}
M_{cy}
M_{lq}
M_{lm}
M_{cq}
M_{cm}
Model

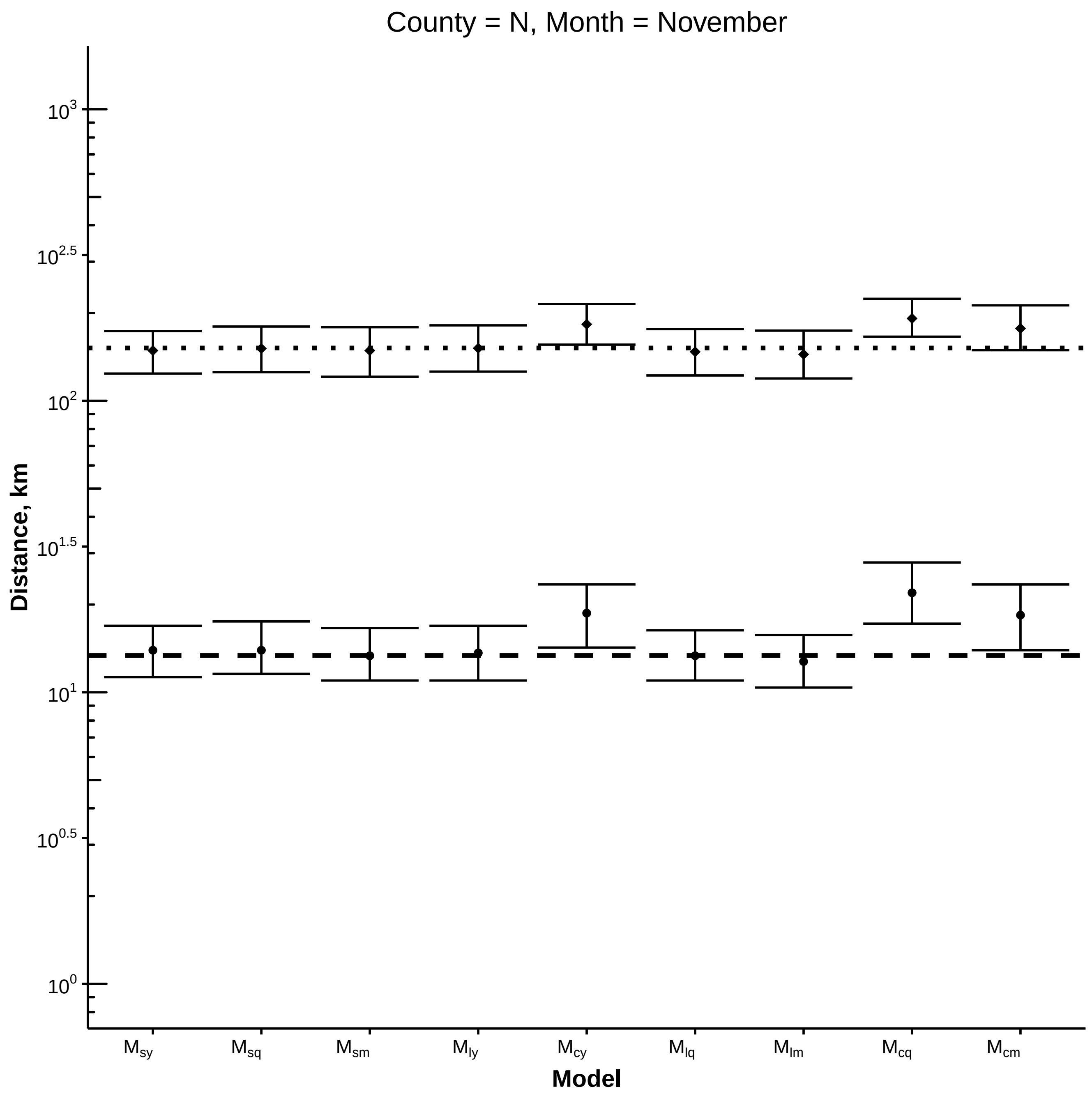

County = N, Month = November
Distance, km
10^3
$10^{2.5}$
10^2
$10^{1.5}$
10^1
$10^{0.5}$
10^0
M_{sy}
M_{sq}
M_{sm}
M_{ly}
M_{cy}
M_{lq}
M_{lm}
M_{cq}
M_{cm}
Model

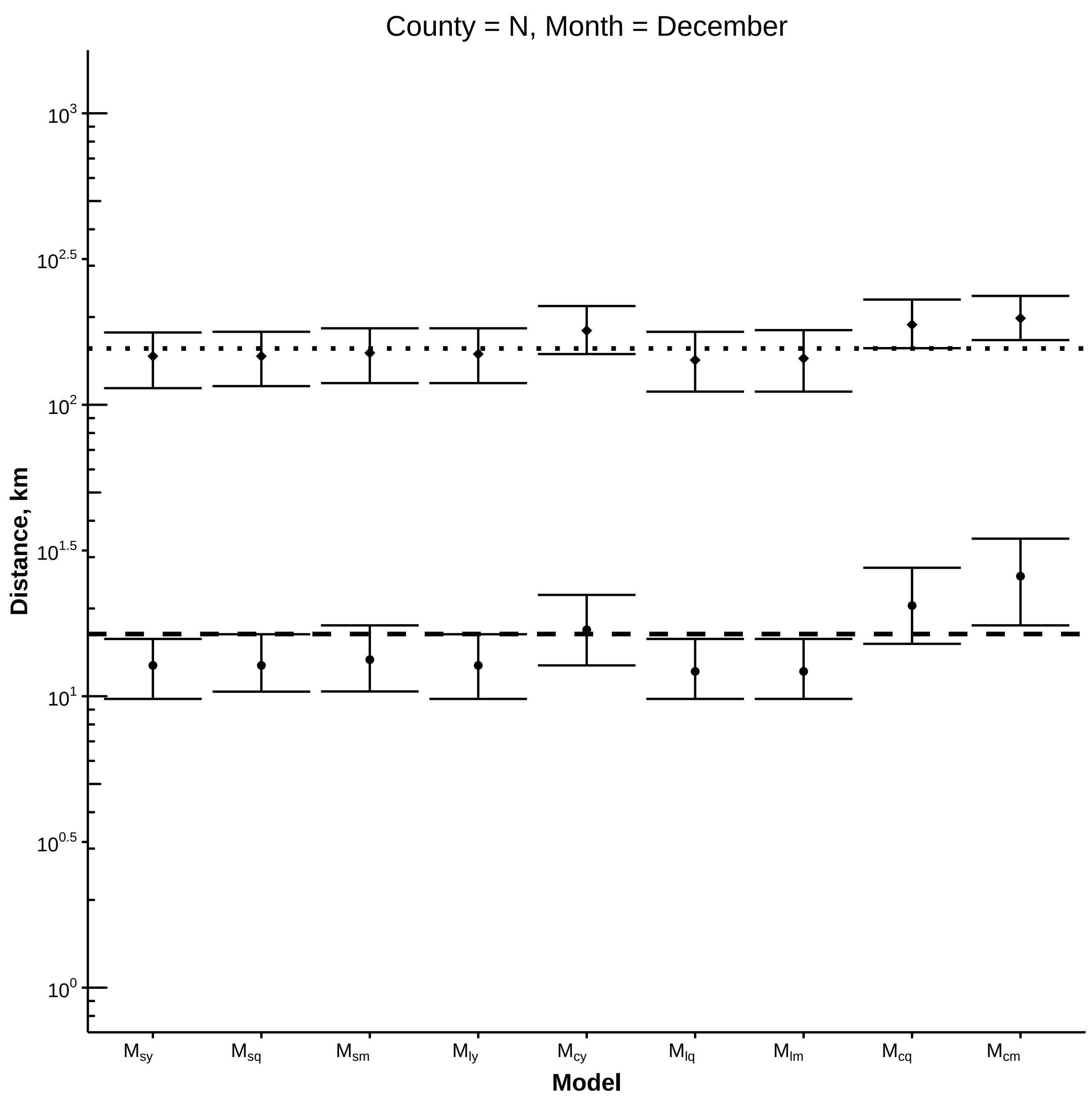

County = N, Month = December
Distance, km
10^3
10^2.5
10^2
10^1.5
10^1
10^0.5
10^0
M_sy
M_sq
M_sm
M_ly
M_cy
M_lq
M_lm
M_cq
M_cm
Model

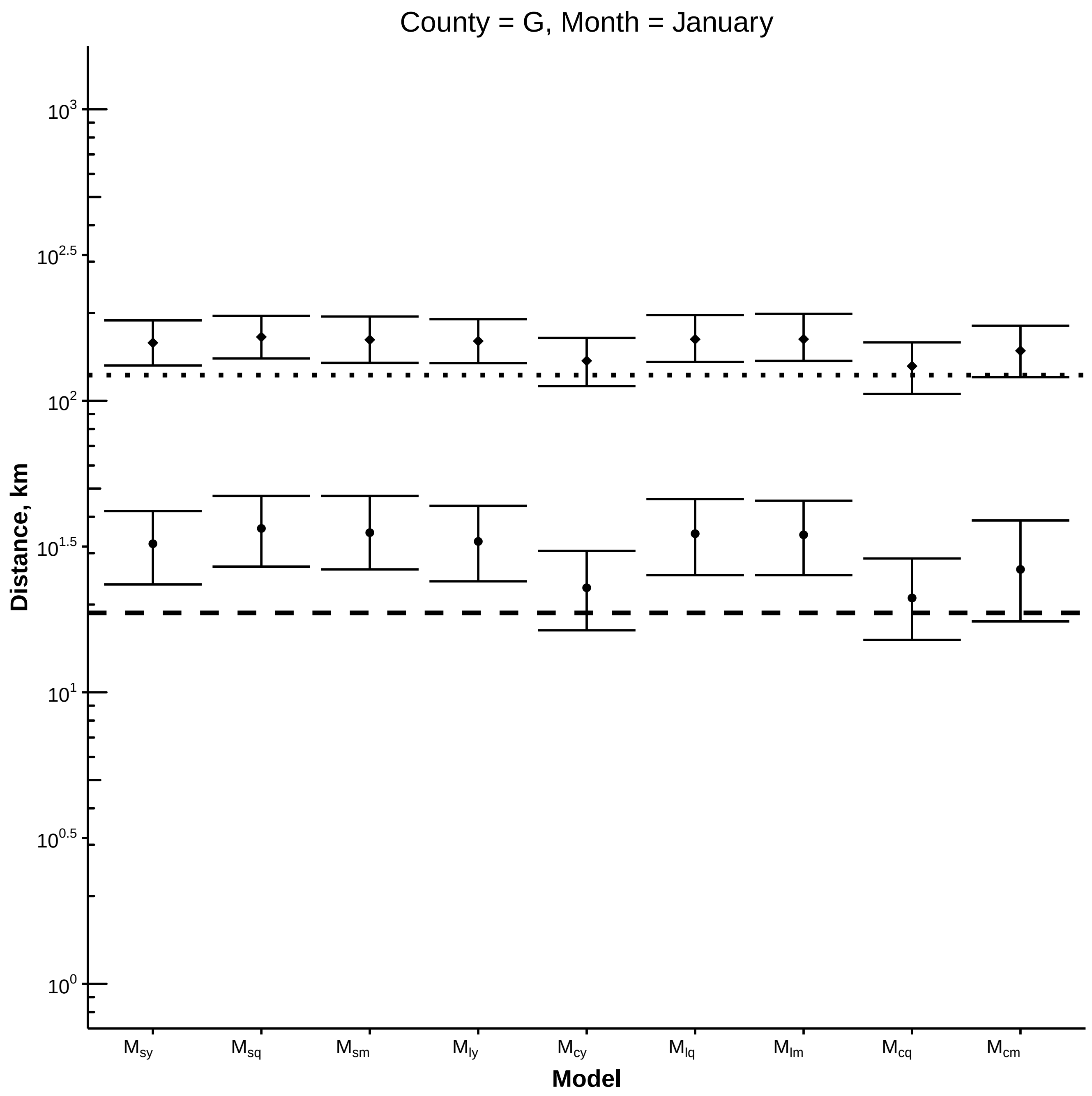
County = G, Month = January
Distance, km
10^3
$10^{2.5}$
10^2
$10^{1.5}$
10^1
$10^{0.5}$
10^0
M_{sy}
M_{sq}
M_{sm}
M_{ly}
M_{cy}
M_{lq}
M_{lm}
M_{cq}
M_{cm}
Model

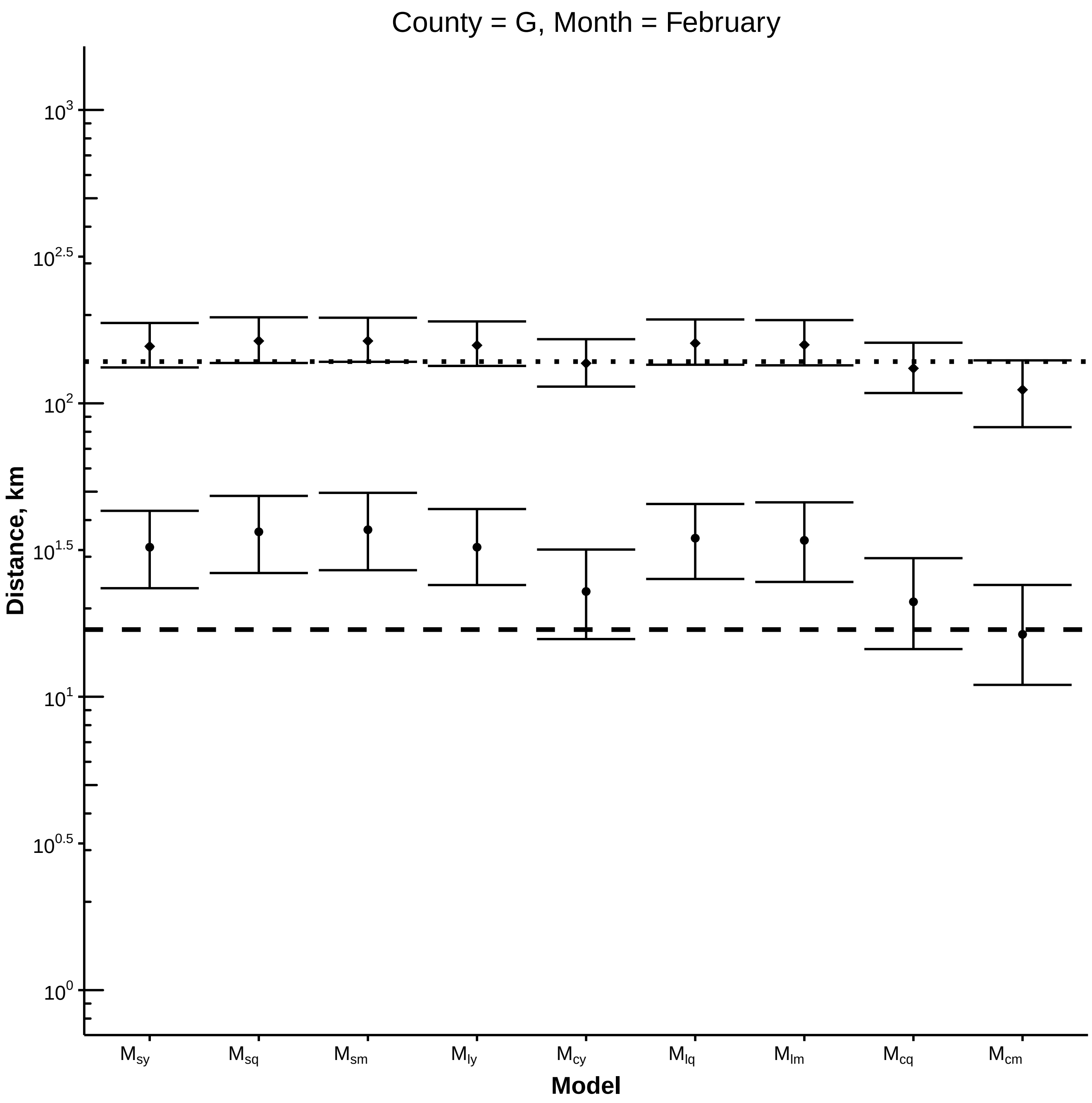
County = G, Month = February
Distance, km
10^3
$10^{2.5}$
10^2
$10^{1.5}$
10^1
$10^{0.5}$
10^0
M_{sy}
M_{sq}
M_{sm}
M_{ly}
M_{cy}
M_{lq}
M_{lm}
M_{cq}
M_{cm}
Model

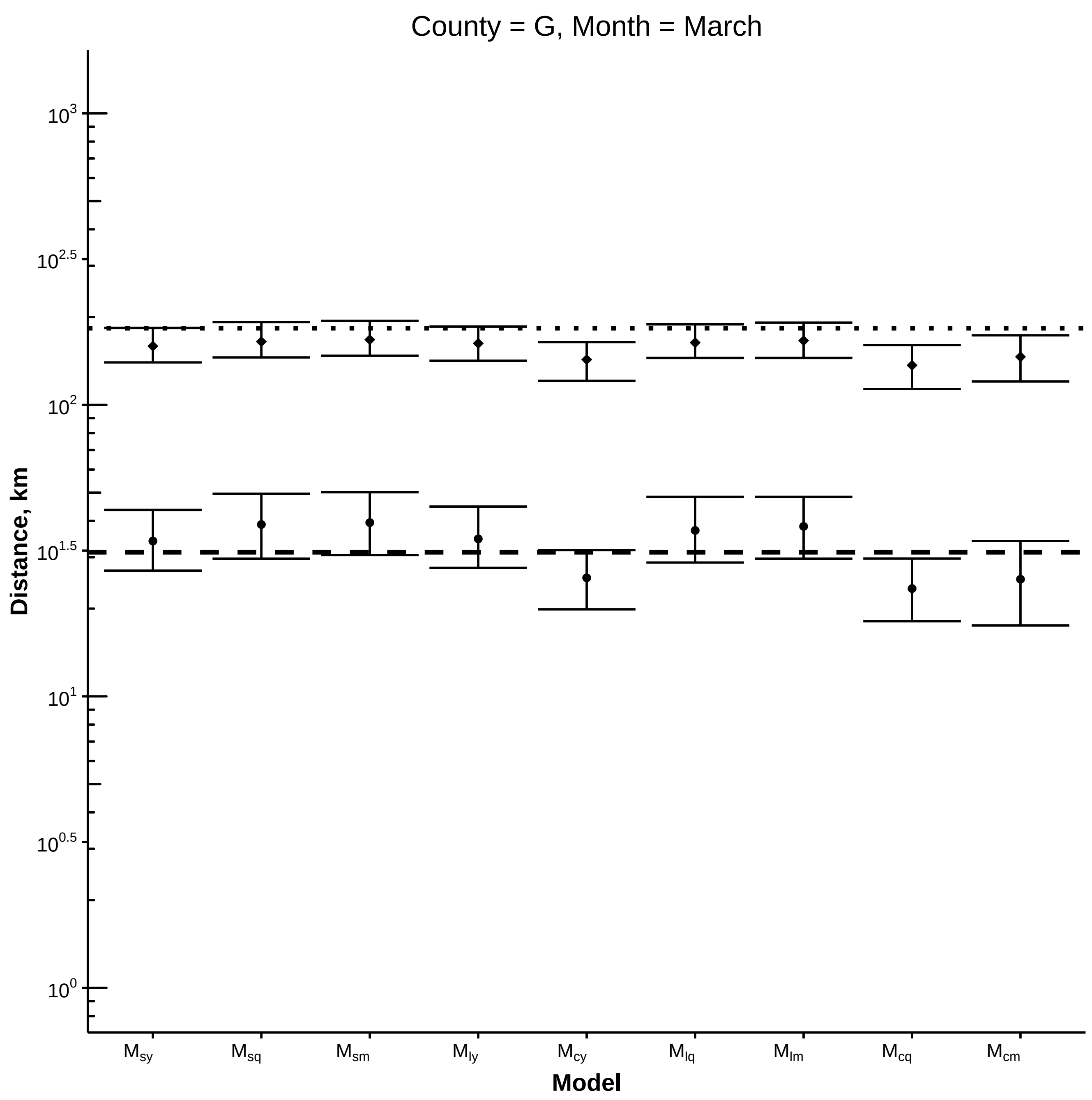
County = G, Month = March
Distance, km
10^3
$10^{2.5}$
10^2
$10^{1.5}$
10^1
$10^{0.5}$
10^0
M_{sy}
M_{sq}
M_{sm}
M_{ly}
M_{cy}
M_{lq}
M_{lm}
M_{cq}
M_{cm}
Model

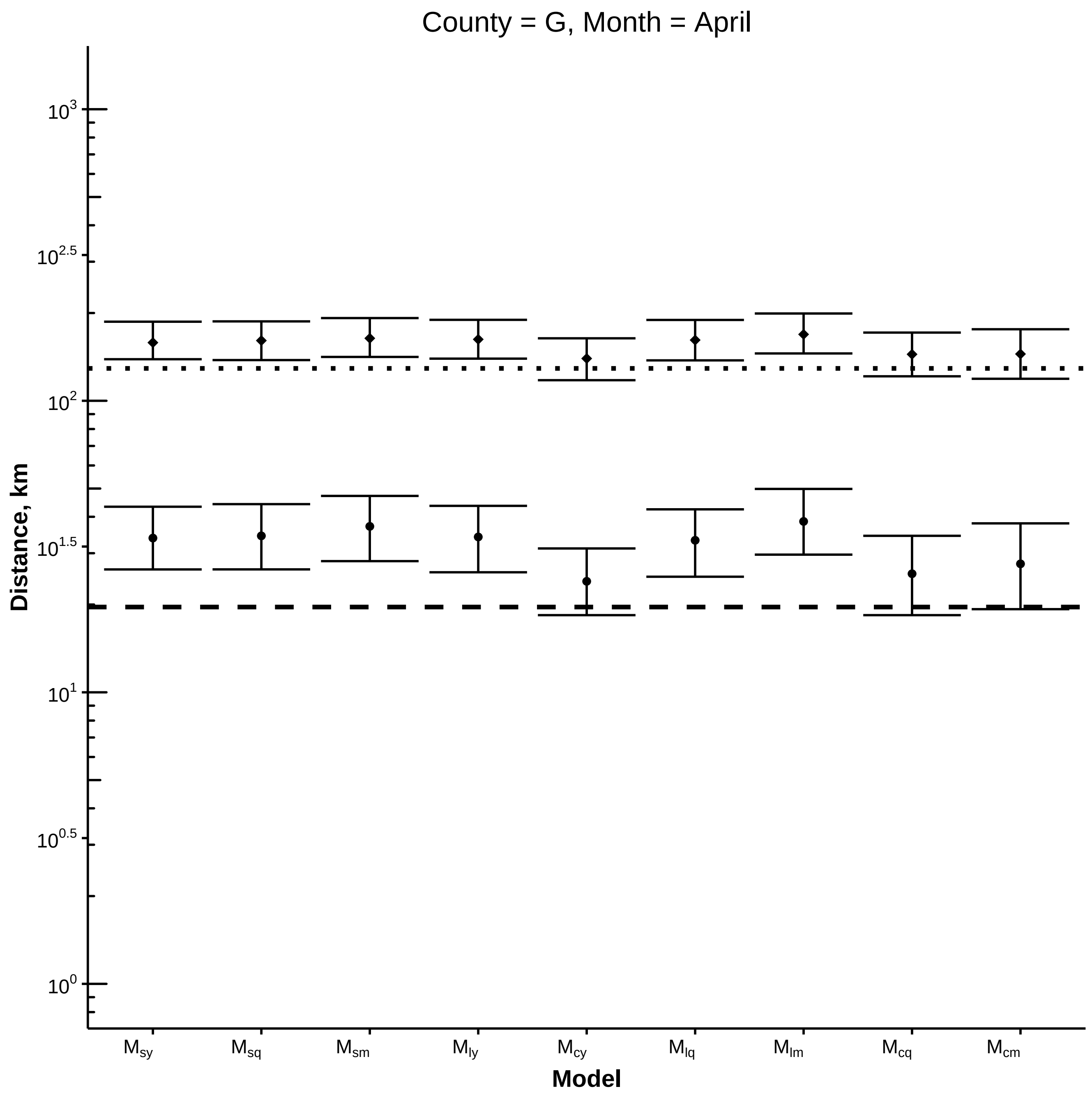

County = G, Month = April
Distance, km
10^3
$10^{2.5}$
10^2
$10^{1.5}$
10^1
$10^{0.5}$
10^0
M_{sy}
M_{sq}
M_{sm}
M_{ly}
M_{cy}
M_{lq}
M_{lm}
M_{cq}
M_{cm}
Model

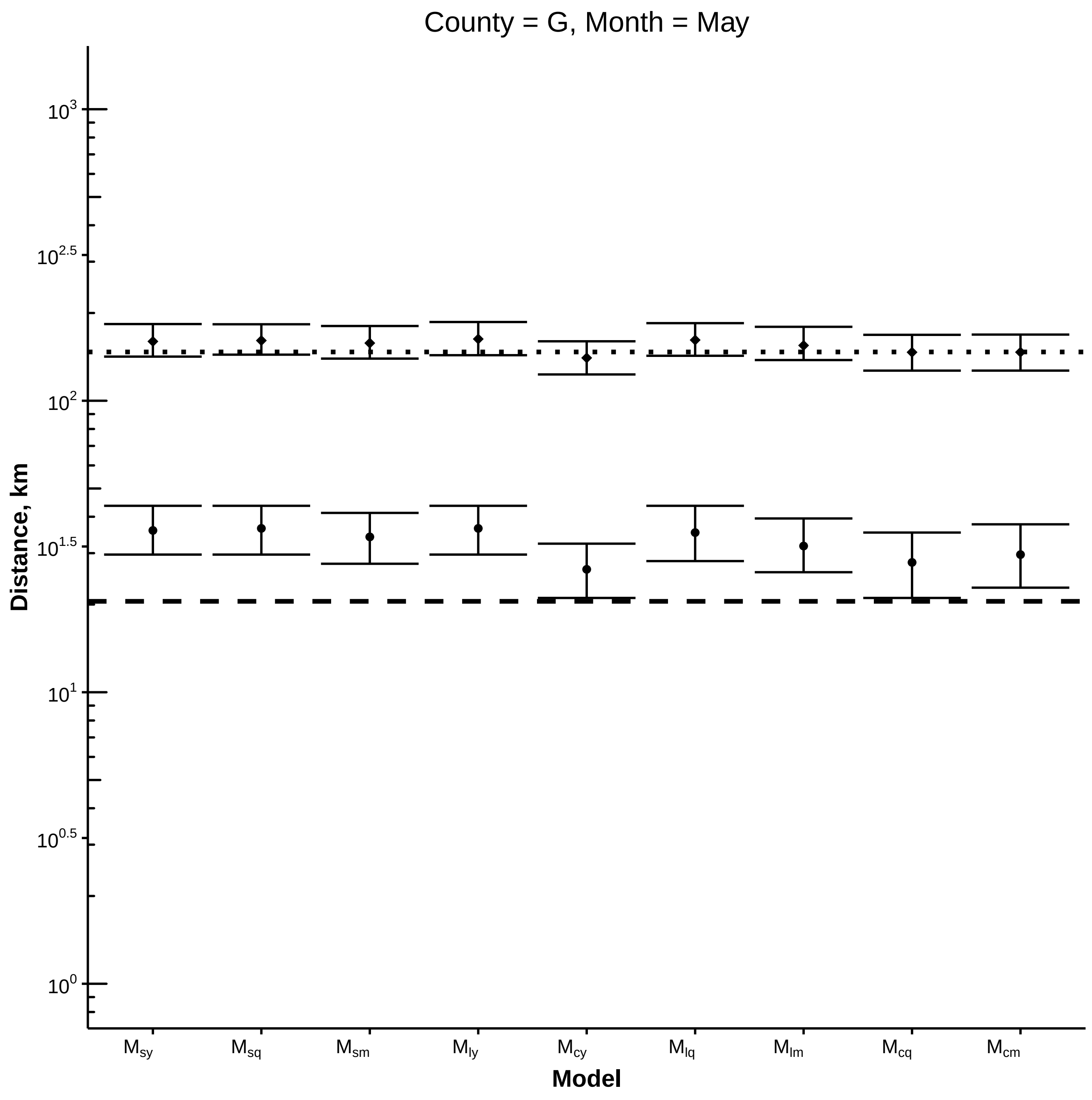

County = G, Month = May
Distance, km
10^3
$10^{2.5}$
10^2
$10^{1.5}$
10^1
$10^{0.5}$
10^0
M_{sy}
M_{sq}
M_{sm}
M_{ly}
M_{cy}
M_{lq}
M_{lm}
M_{cq}
M_{cm}
Model

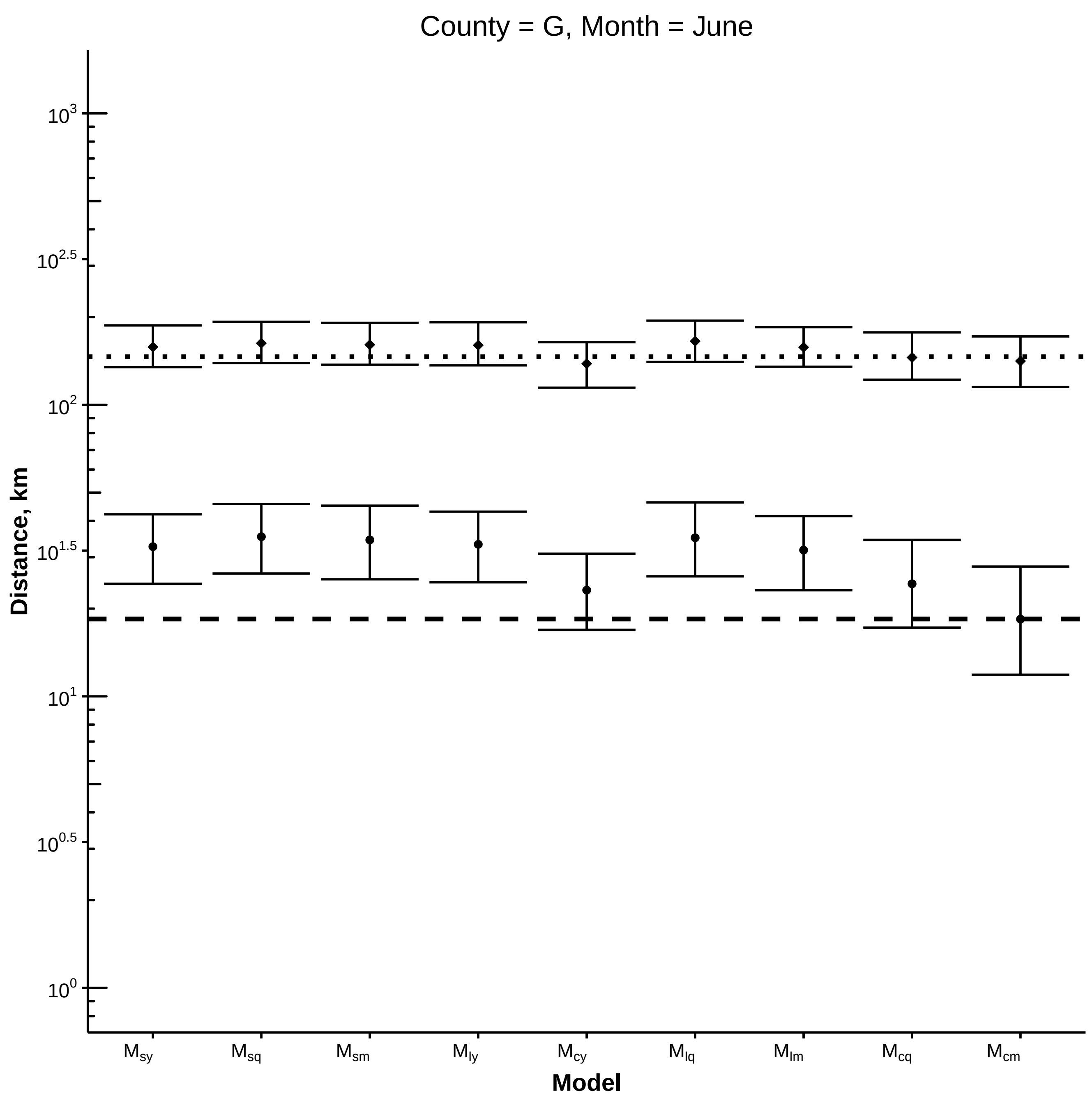

County = G, Month = June
Distance, km
10^3
$10^{2.5}$
10^2
$10^{1.5}$
10^1
$10^{0.5}$
10^0
M_{sy}
M_{sq}
M_{sm}
M_{ly}
M_{cy}
M_{lq}
M_{lm}
M_{cq}
M_{cm}
Model

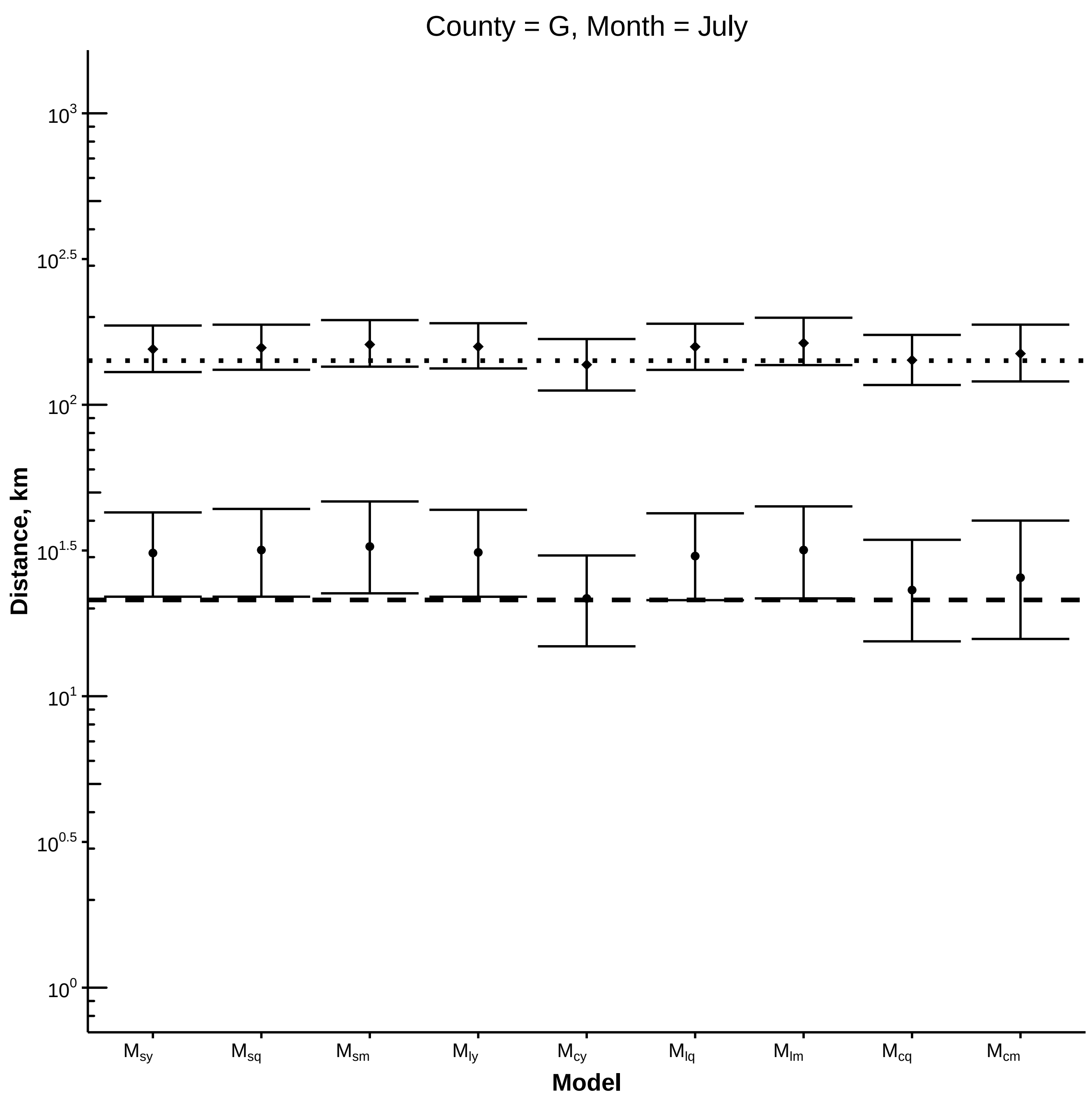

County = G, Month = July
Distance, km
10^3
$10^{2.5}$
10^2
$10^{1.5}$
10^1
$10^{0.5}$
10^0
M_{sy}
M_{sq}
M_{sm}
M_{ly}
M_{cy}
M_{lq}
M_{lm}
M_{cq}
M_{cm}
Model

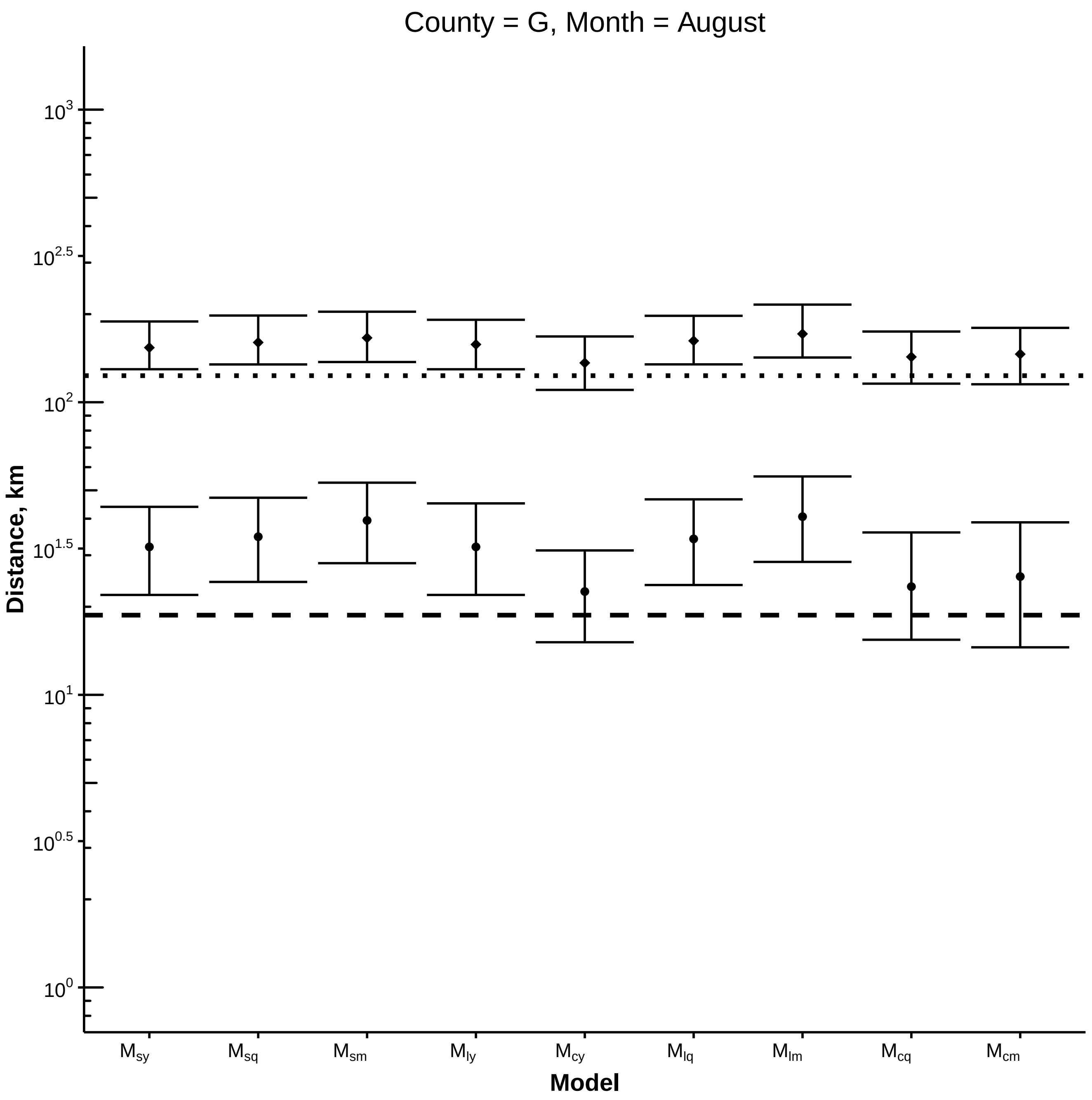

County = G, Month = August
Distance, km
10^3
10^2.5
10^2
10^1.5
10^1
10^0.5
10^0
Msy
Msq
Msm
Mly
Mcy
Mlq
Mlm
Mcq
Mcm
Model

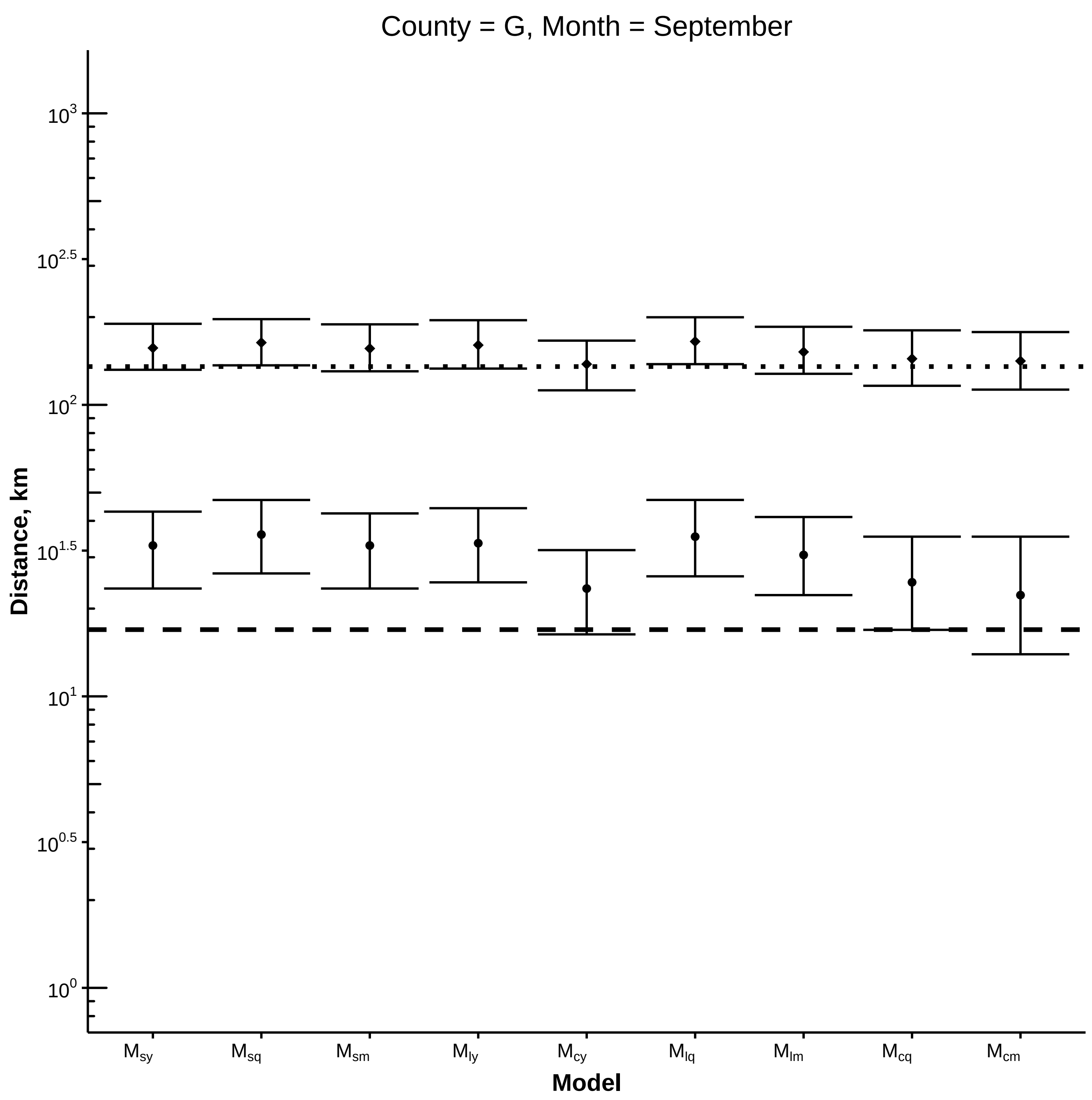

County = G, Month = September
Distance, km
10^3
$10^{2.5}$
10^2
$10^{1.5}$
10^1
$10^{0.5}$
10^0
M_{sy}
M_{sq}
M_{sm}
M_{ly}
M_{cy}
M_{lq}
M_{lm}
M_{cq}
M_{cm}
Model

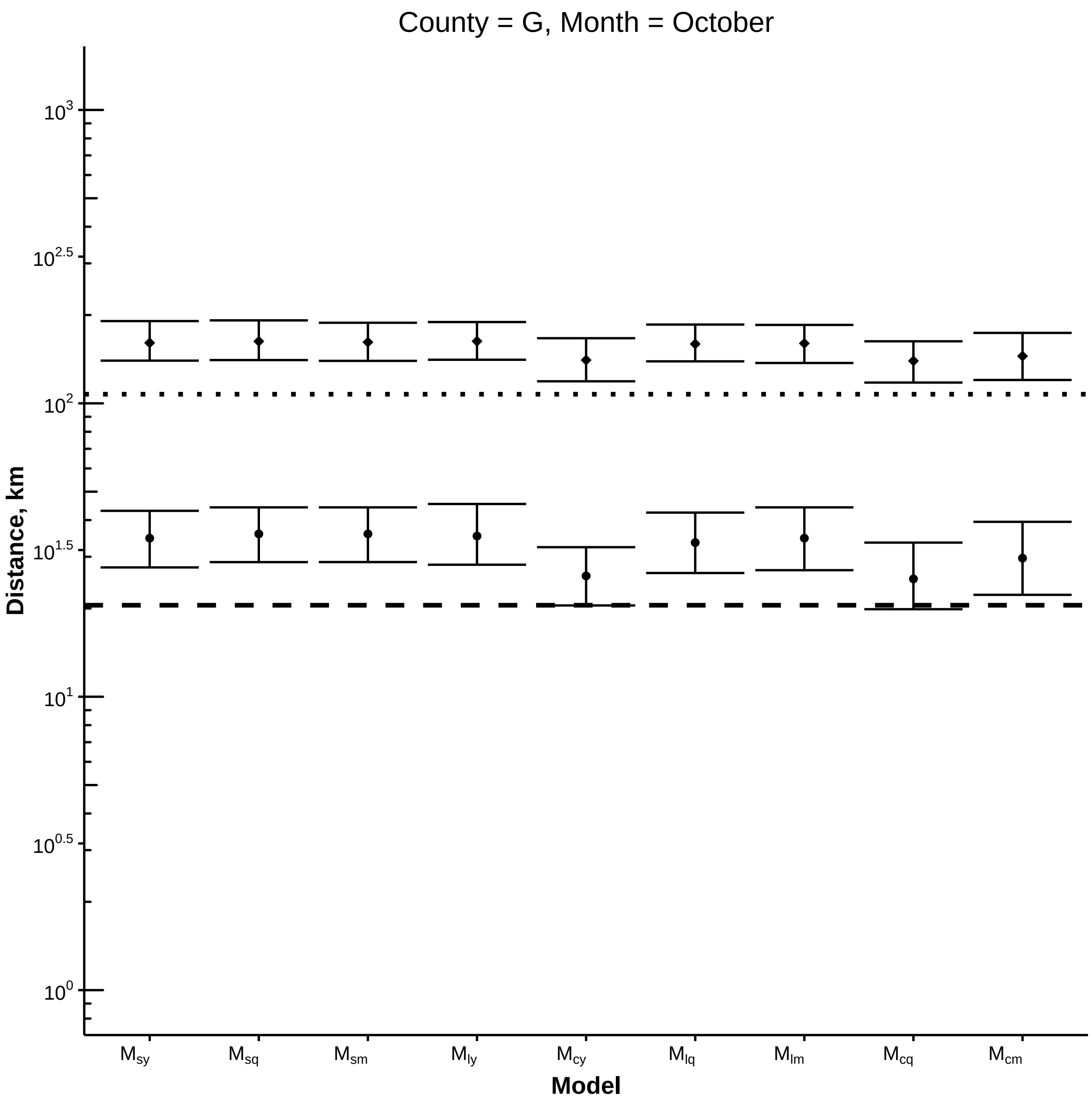
County = G, Month = October
Distance, km
10^3
$10^{2.5}$
10^2
$10^{1.5}$
10^1
$10^{0.5}$
10^0
M_{sy}
M_{sq}
M_{sm}
M_{ly}
M_{cy}
M_{lq}
M_{lm}
M_{cq}
M_{cm}
Model

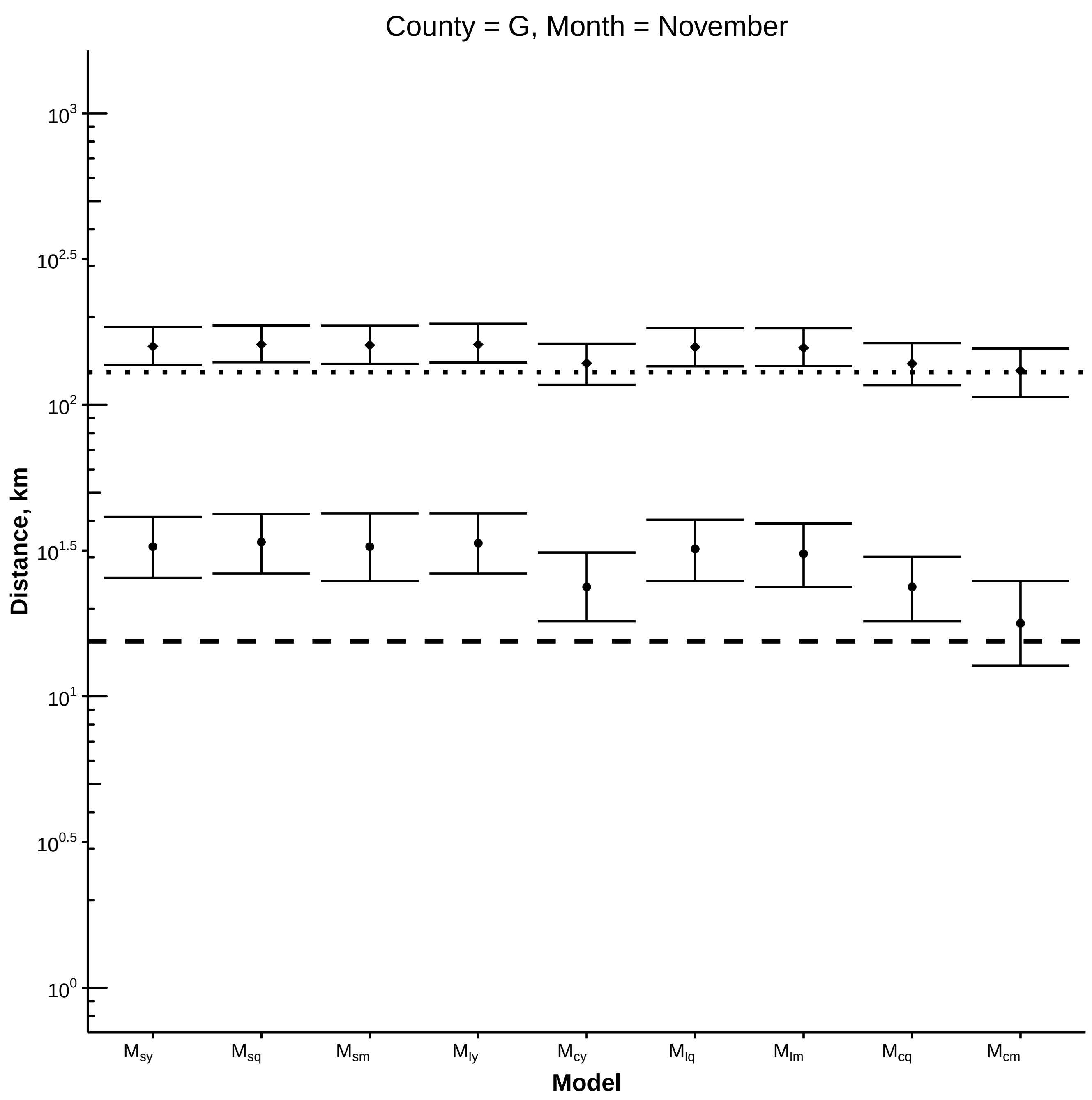

County = G, Month = November
Distance, km
10^3
$10^{2.5}$
10^2
$10^{1.5}$
10^1
$10^{0.5}$
10^0
M_{sy}
M_{sq}
M_{sm}
M_{ly}
M_{cy}
M_{lq}
M_{lm}
M_{cq}
M_{cm}
Model

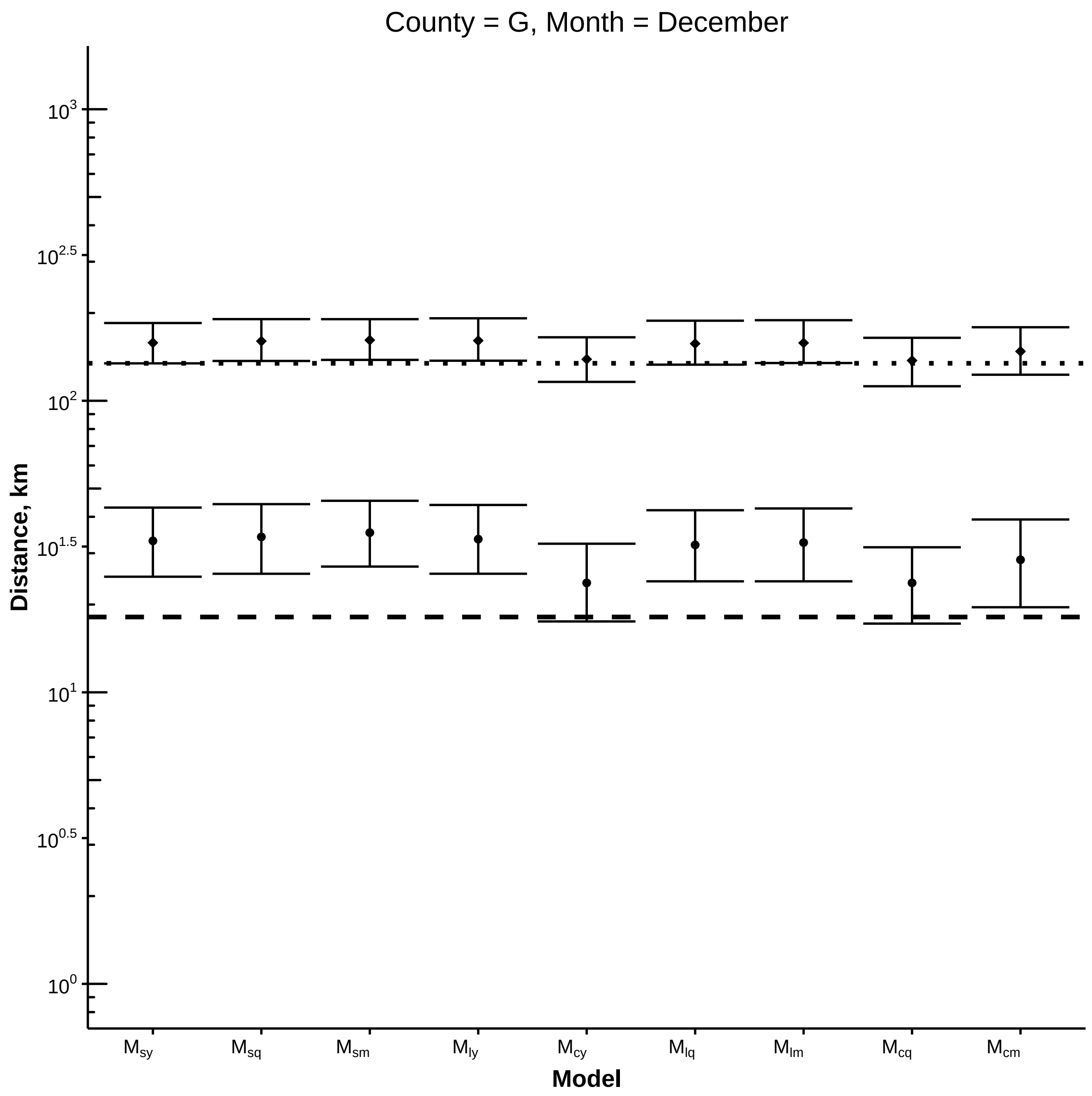

County = G, Month = December
Distance, km
Model
10^0
$10^{0.5}$
10^1
$10^{1.5}$
10^2
$10^{2.5}$
10^3
M_{sy}
M_{sq}
M_{sm}
M_{ly}
M_{cy}
M_{lq}
M_{lm}
M_{cq}
M_{cm}

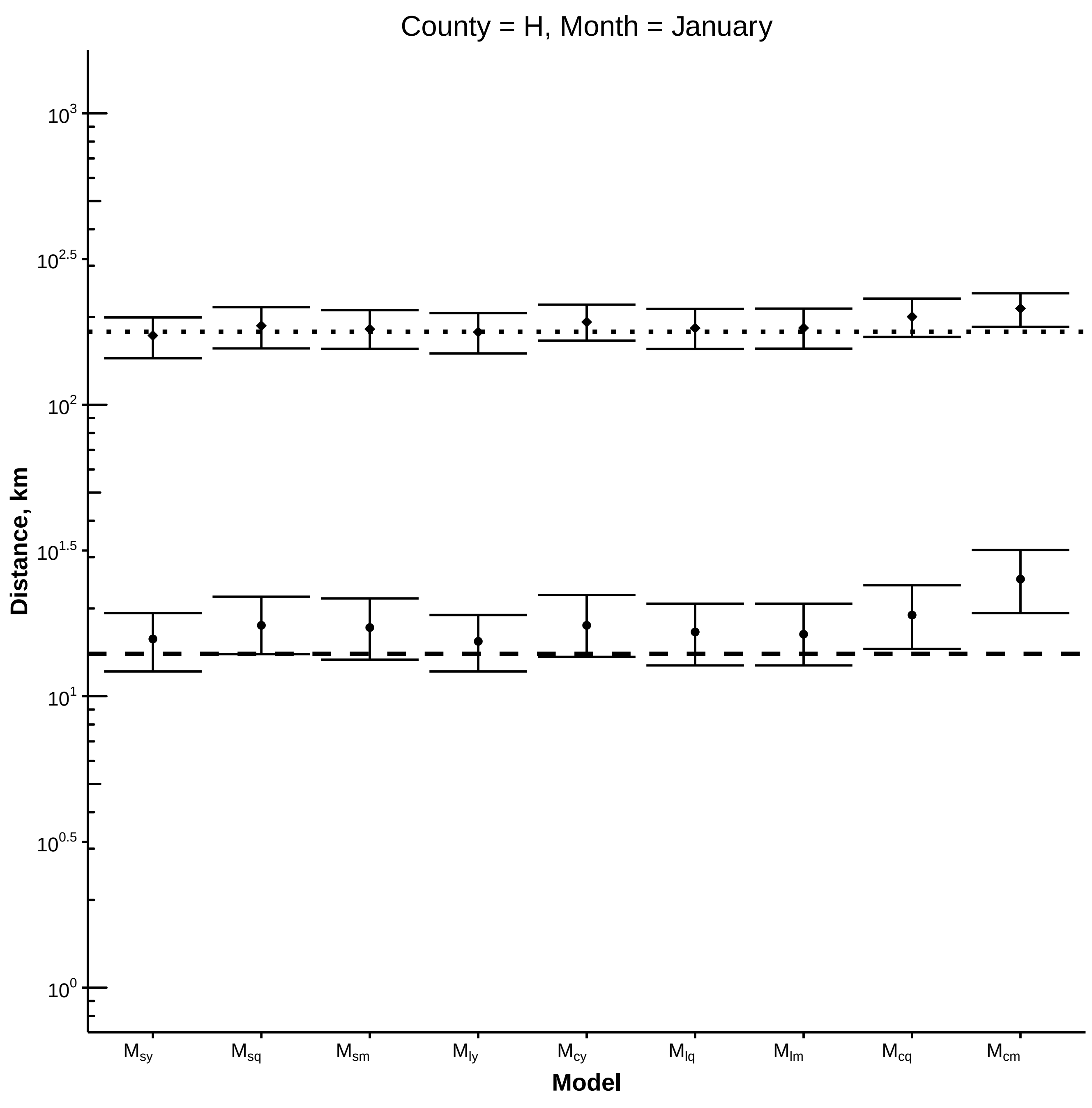
County = H, Month = January
Distance, km
10^3
$10^{2.5}$
10^2
$10^{1.5}$
10^1
$10^{0.5}$
10^0
M_{sy} M_{sq} M_{sm} M_{ly} M_{cy} M_{lq} M_{lm} M_{cq} M_{cm}
Model

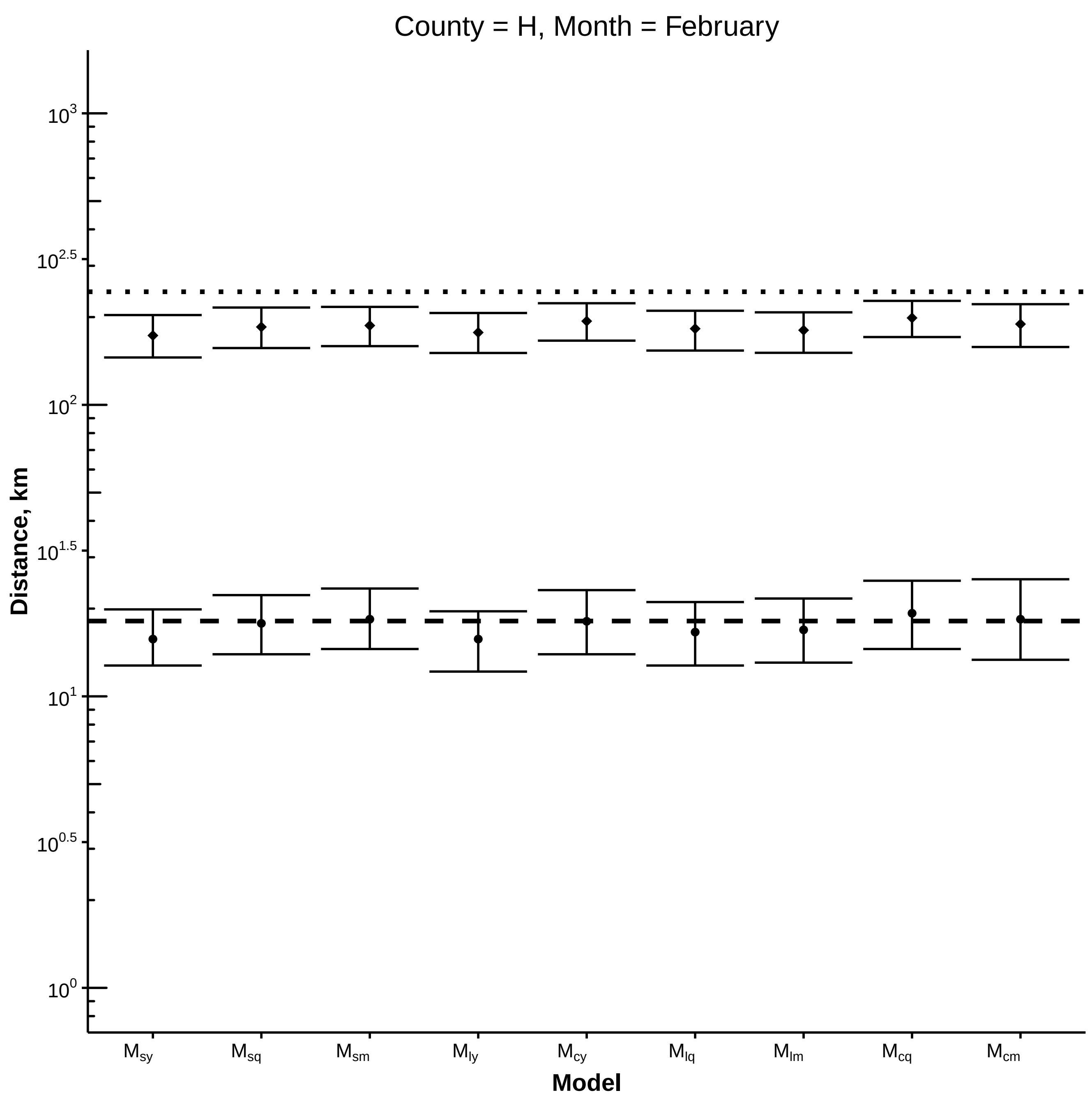
County = H, Month = February
Distance, km
10^3
$10^{2.5}$
10^2
$10^{1.5}$
10^1
$10^{0.5}$
10^0
M_{sy}
M_{sq}
M_{sm}
M_{ly}
M_{cy}
M_{lq}
M_{lm}
M_{cq}
M_{cm}
Model

County = H, Month = March

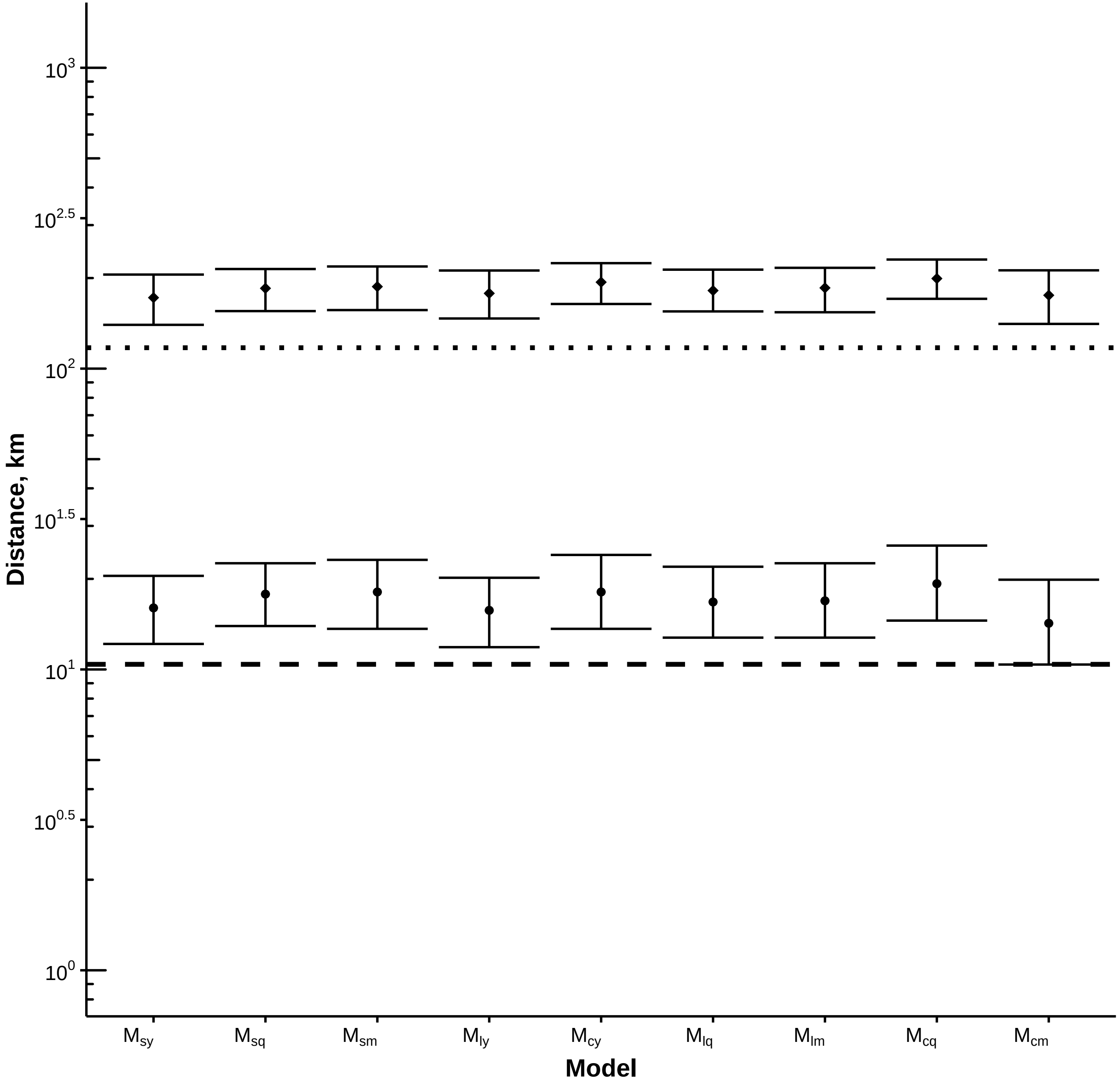

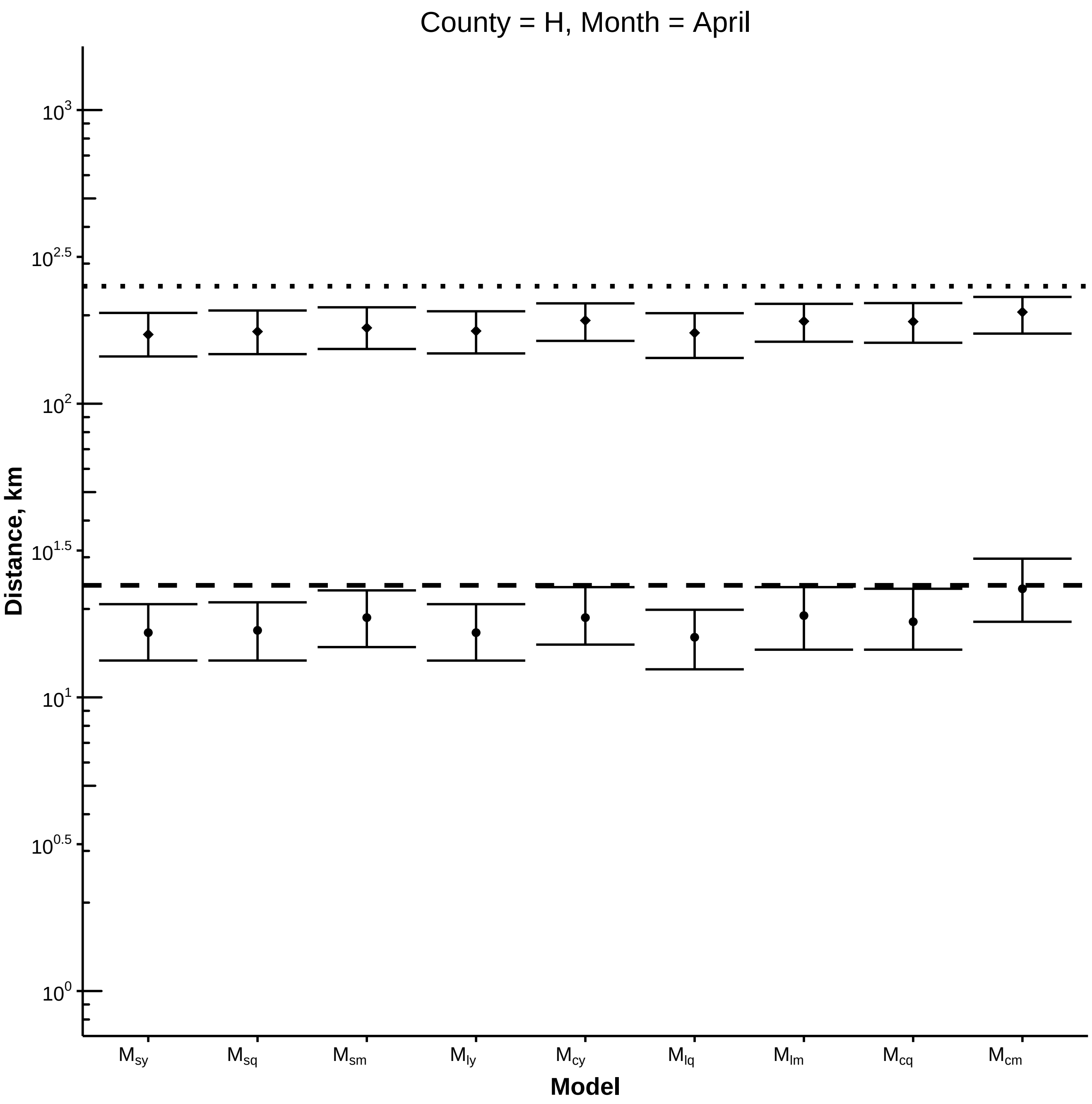
County = H, Month = April
Distance, km
10^3
10^2.5
10^2
10^1.5
10^1
10^0.5
10^0
M_sy
M_sq
M_sm
M_ly
M_cy
M_lq
M_lm
M_cq
M_cm
Model

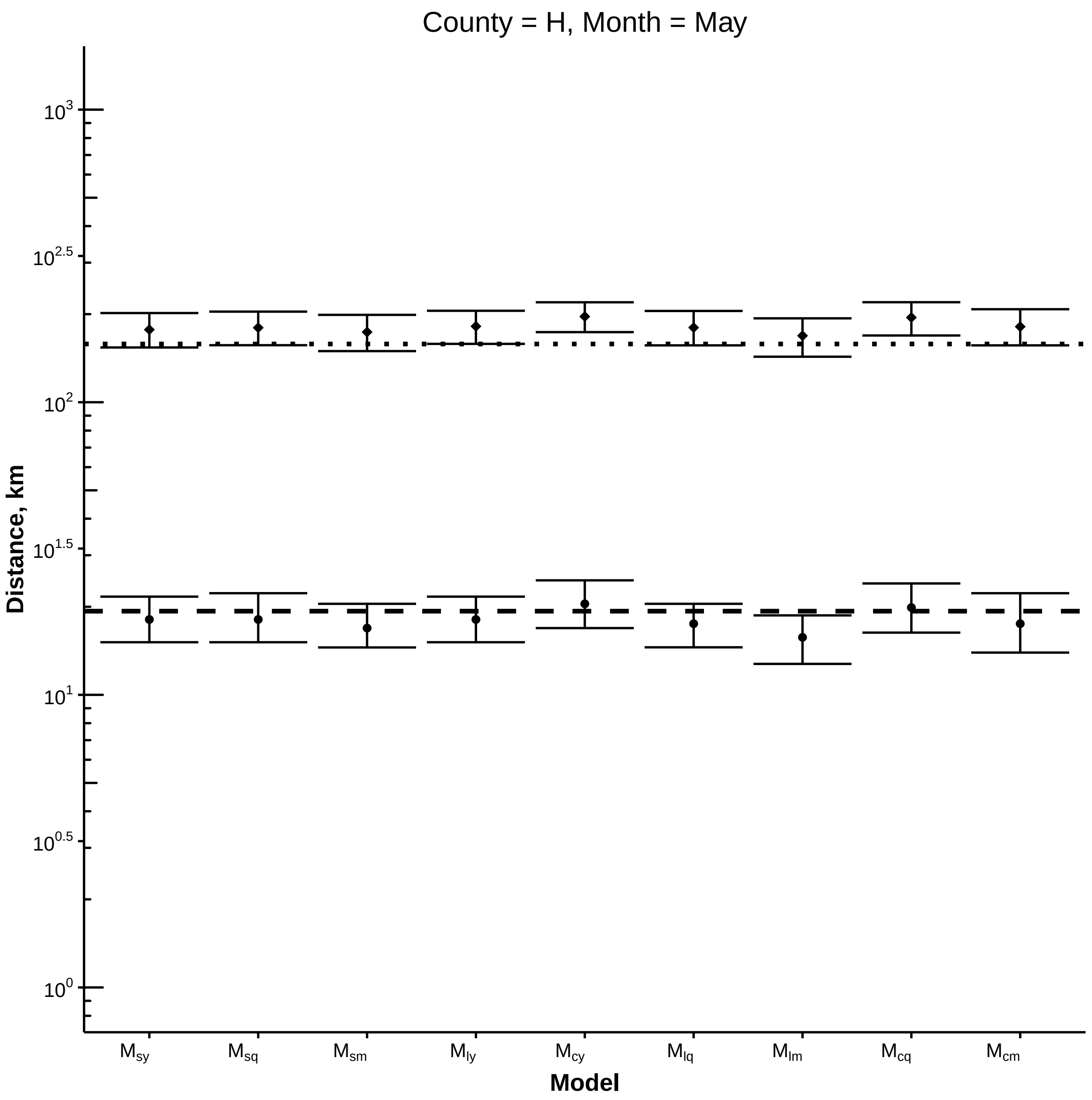
County = H, Month = May
Distance, km
10^3
$10^{2.5}$
10^2
$10^{1.5}$
10^1
$10^{0.5}$
10^0
M_{sy}
M_{sq}
M_{sm}
M_{ly}
M_{cy}
M_{lq}
M_{lm}
M_{cq}
M_{cm}
Model

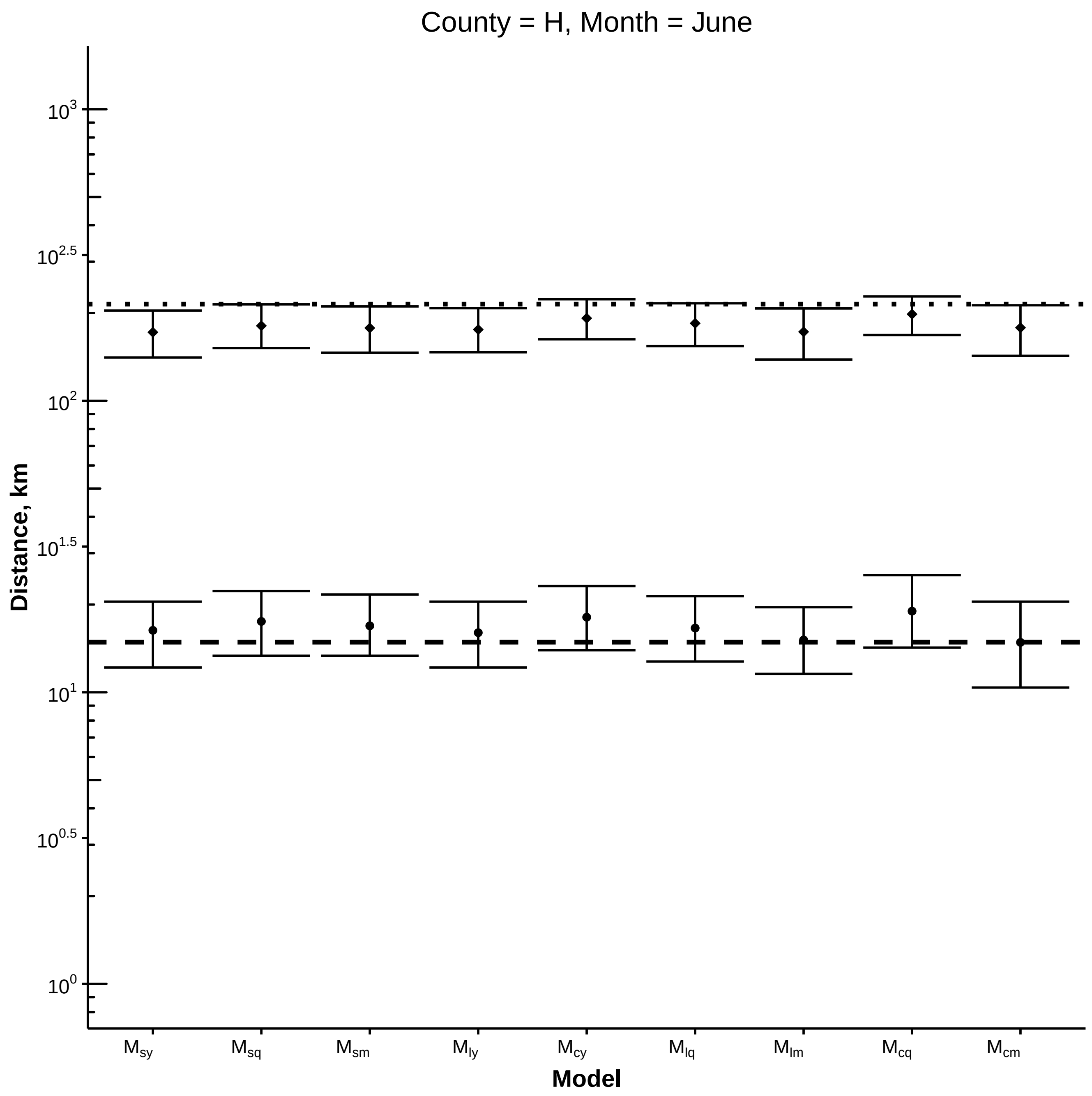

County = H, Month = June
Distance, km
10^3
10^2.5
10^2
10^1.5
10^1
10^0.5
10^0
M_sy
M_sq
M_sm
M_ly
M_cy
M_lq
M_lm
M_cq
M_cm
Model

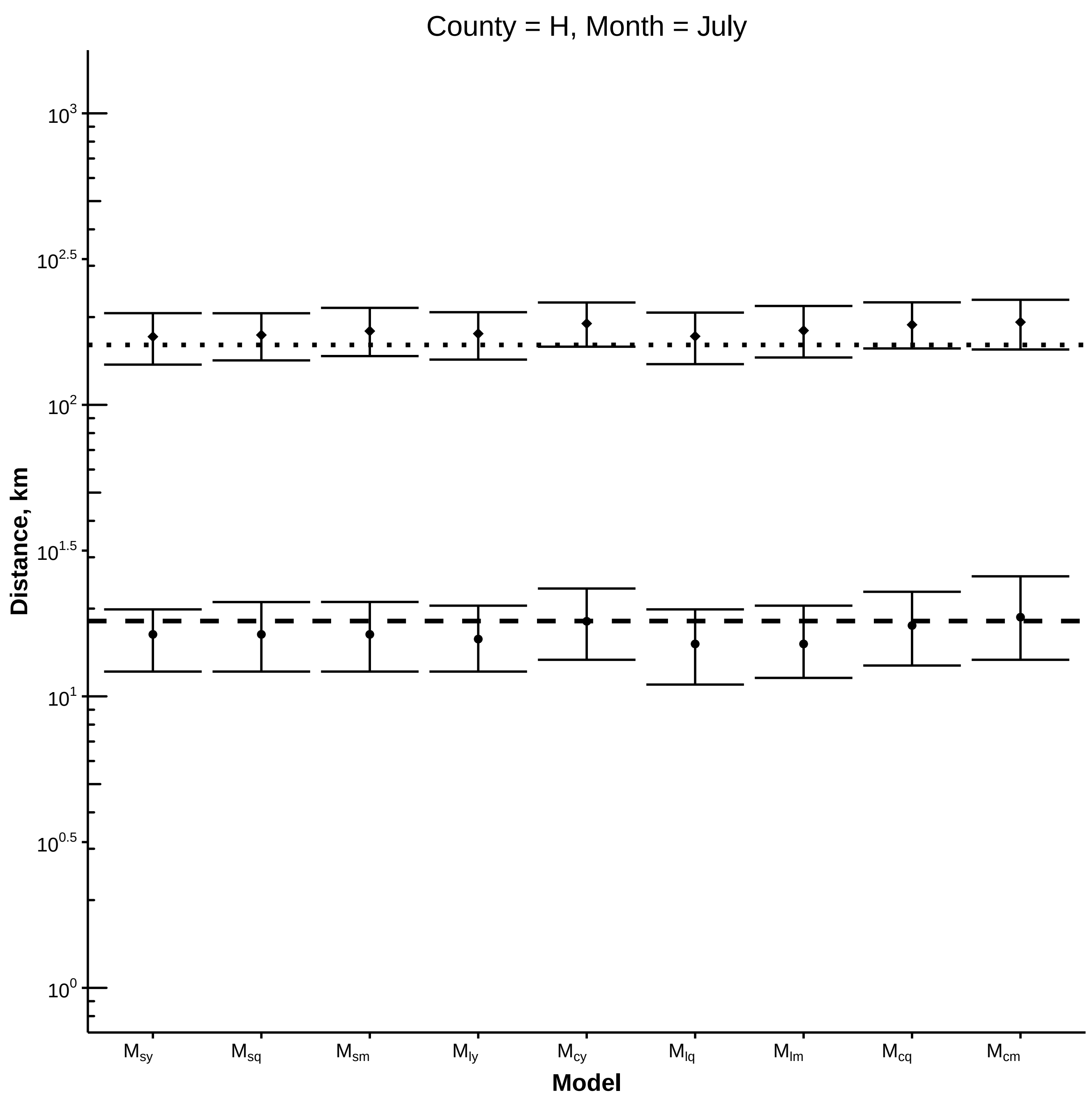

County = H, Month = July
Distance, km
10^3
$10^{2.5}$
10^2
$10^{1.5}$
10^1
$10^{0.5}$
10^0
M_{sy}
M_{sq}
M_{sm}
M_{ly}
M_{cy}
M_{lq}
M_{lm}
M_{cq}
M_{cm}
Model

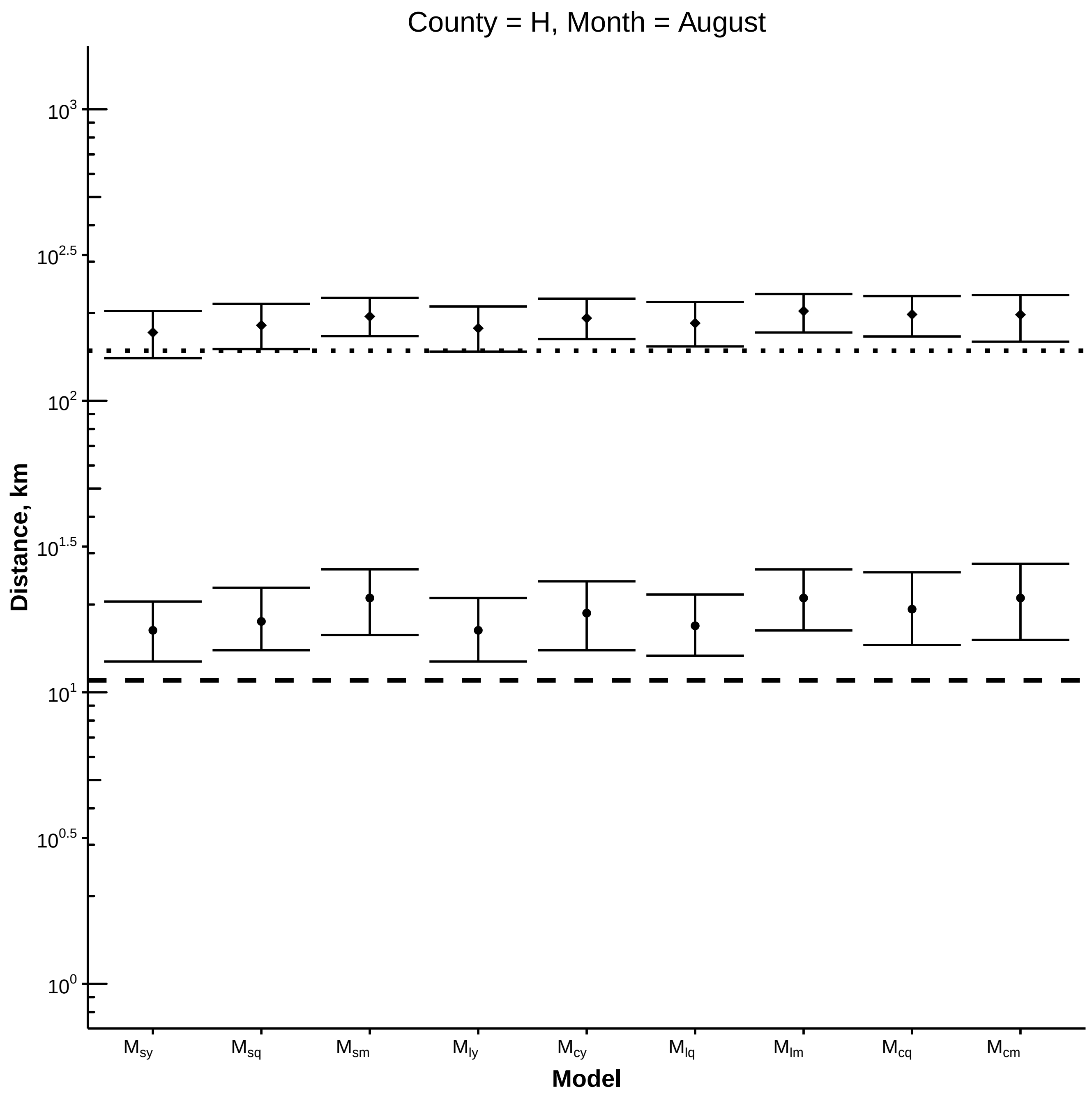

County = H, Month = August
Distance, km
10^3
$10^{2.5}$
10^2
$10^{1.5}$
10^1
$10^{0.5}$
10^0
M_{sy}
M_{sq}
M_{sm}
M_{ly}
M_{cy}
M_{lq}
M_{lm}
M_{cq}
M_{cm}
Model

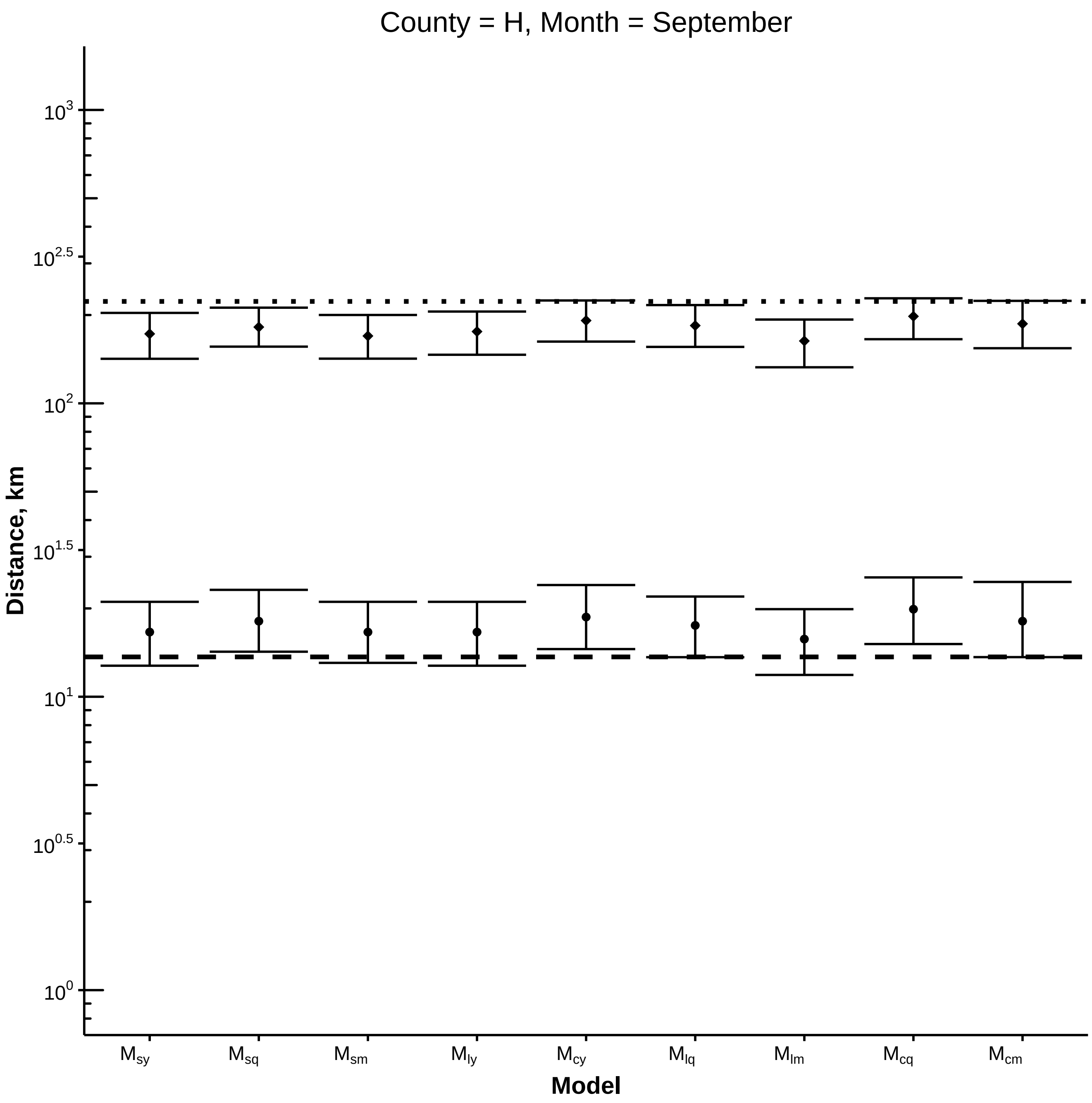
County = H, Month = September
Distance, km
10^3
$10^{2.5}$
10^2
$10^{1.5}$
10^1
$10^{0.5}$
10^0
M_{sy}
M_{sq}
M_{sm}
M_{ly}
M_{cy}
M_{lq}
M_{lm}
M_{cq}
M_{cm}
Model

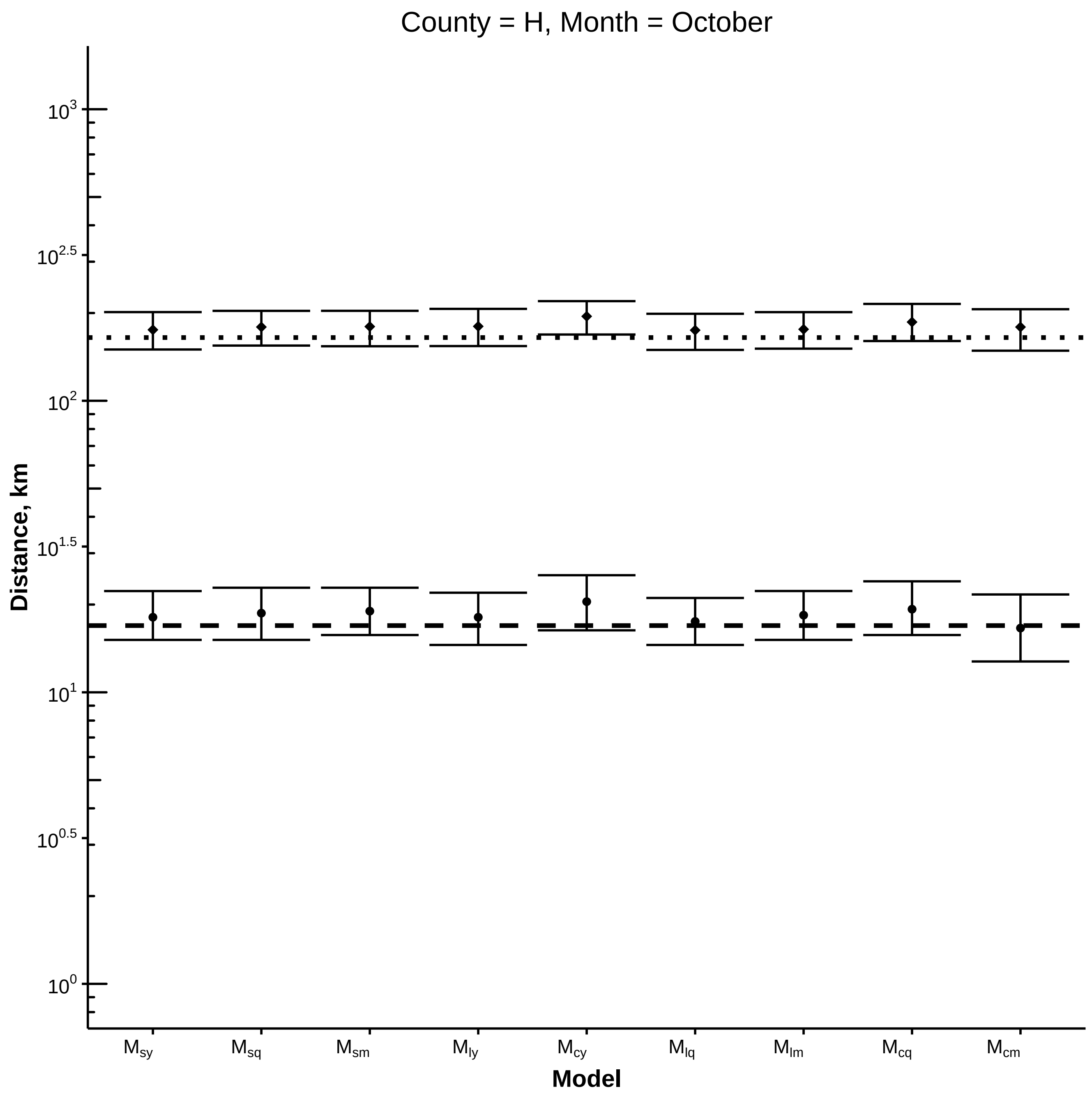

County = H, Month = October
Distance, km
10^3
10^2.5
10^2
10^1.5
10^1
10^0.5
10^0
M_sy
M_sq
M_sm
M_ly
M_cy
M_lq
M_lm
M_cq
M_cm
Model

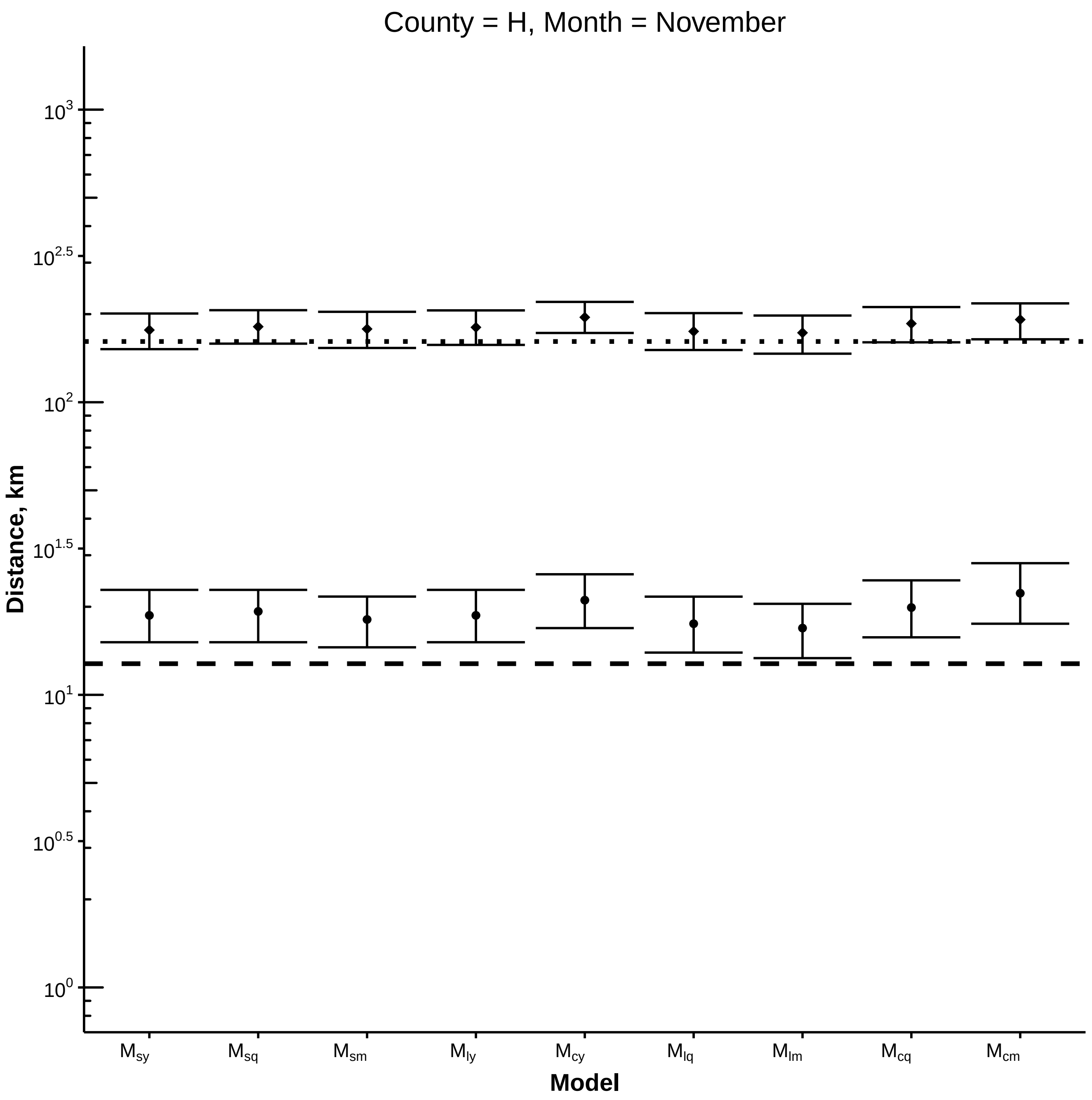

County = H, Month = November
Distance, km
10^3
$10^{2.5}$
10^2
$10^{1.5}$
10^1
$10^{0.5}$
10^0
M_{sy}
M_{sq}
M_{sm}
M_{ly}
M_{cy}
M_{lq}
M_{lm}
M_{cq}
M_{cm}
Model

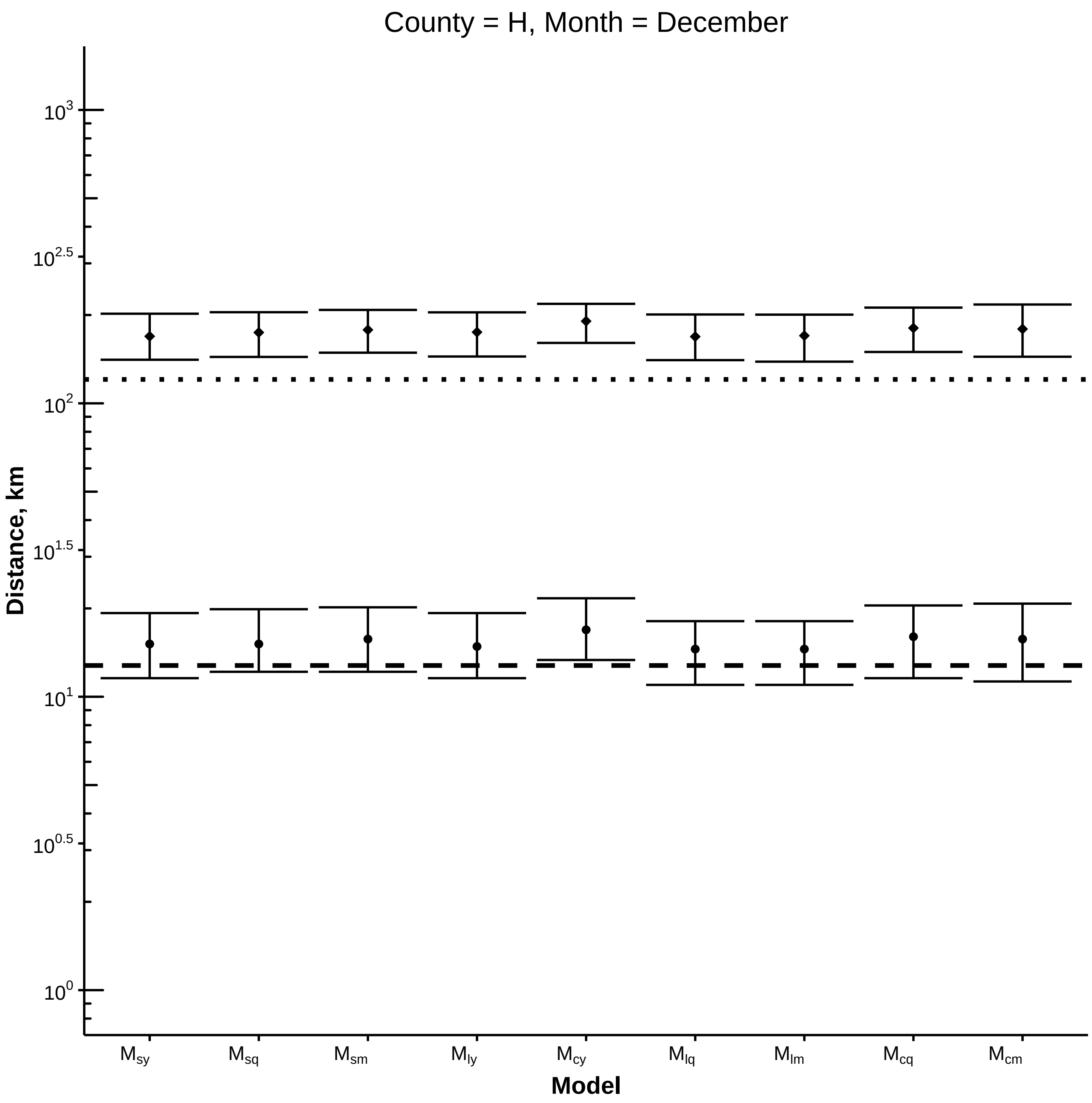

County = H, Month = December
Distance, km
10^3
10^2.5
10^2
10^1.5
10^1
10^0.5
10^0
Msy
Msq
Msm
Mly
Mcy
Mlq
Mlm
Mcq
Mcm
Model

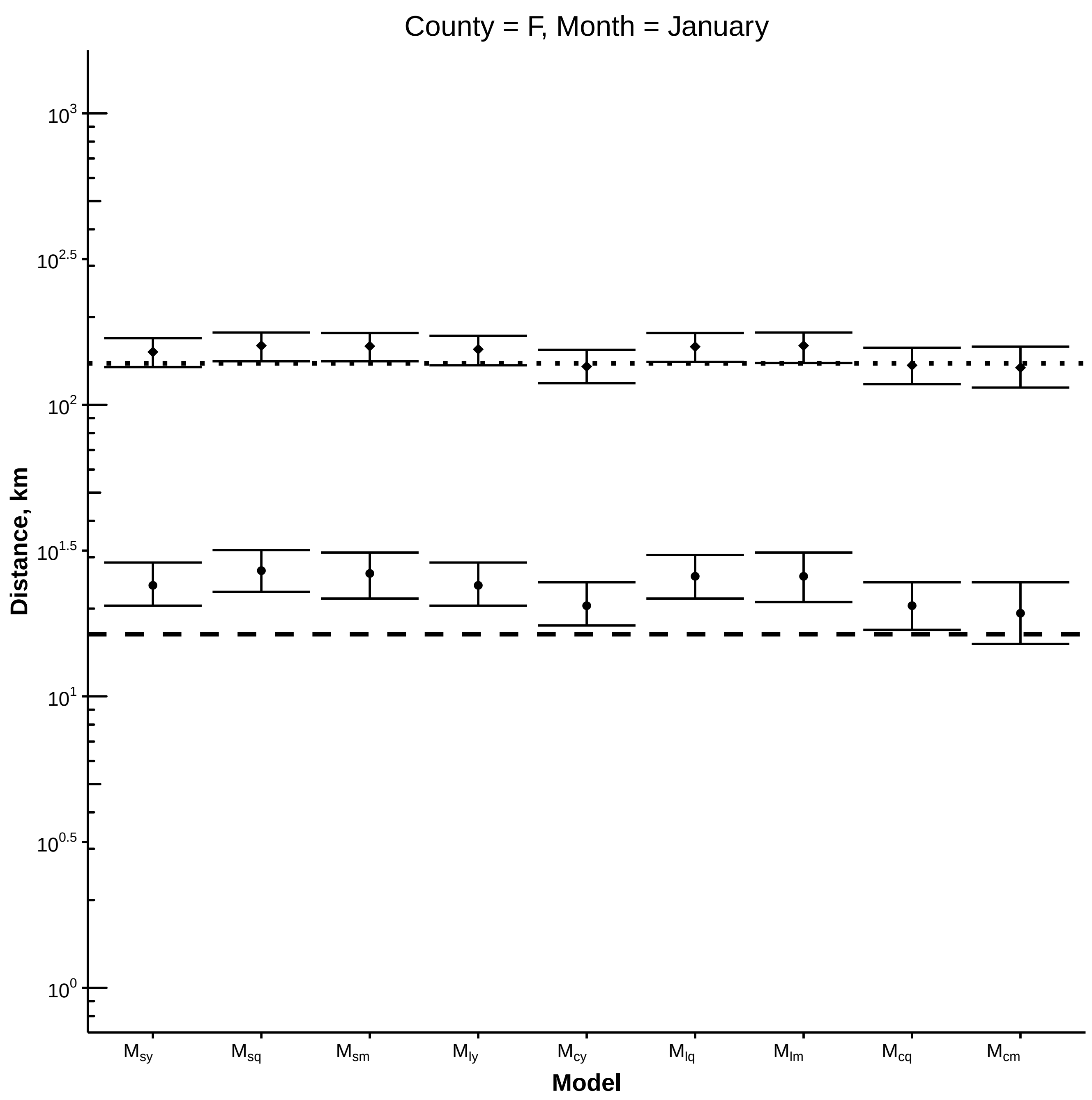

County = F, Month = January
Distance, km
10^3
$10^{2.5}$
10^2
$10^{1.5}$
10^1
$10^{0.5}$
10^0
M_{sy}
M_{sq}
M_{sm}
M_{ly}
M_{cy}
M_{lq}
M_{lm}
M_{cq}
M_{cm}
Model

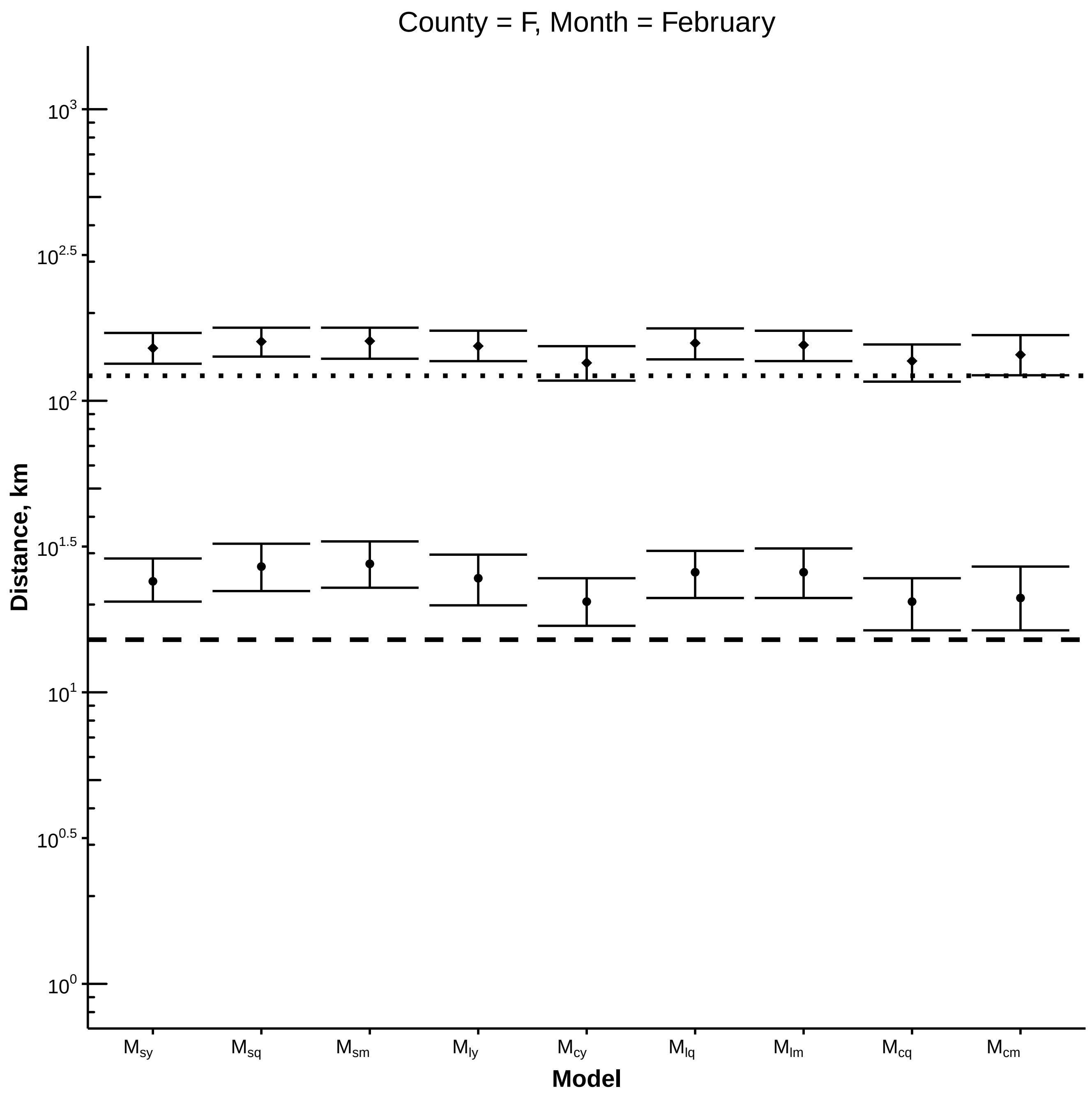

County = F, Month = February
Distance, km
Model
10^3
$10^{2.5}$
10^2
$10^{1.5}$
10^1
$10^{0.5}$
10^0
M_{sy}
M_{sq}
M_{sm}
M_{ly}
M_{cy}
M_{lq}
M_{lm}
M_{cq}
M_{cm}

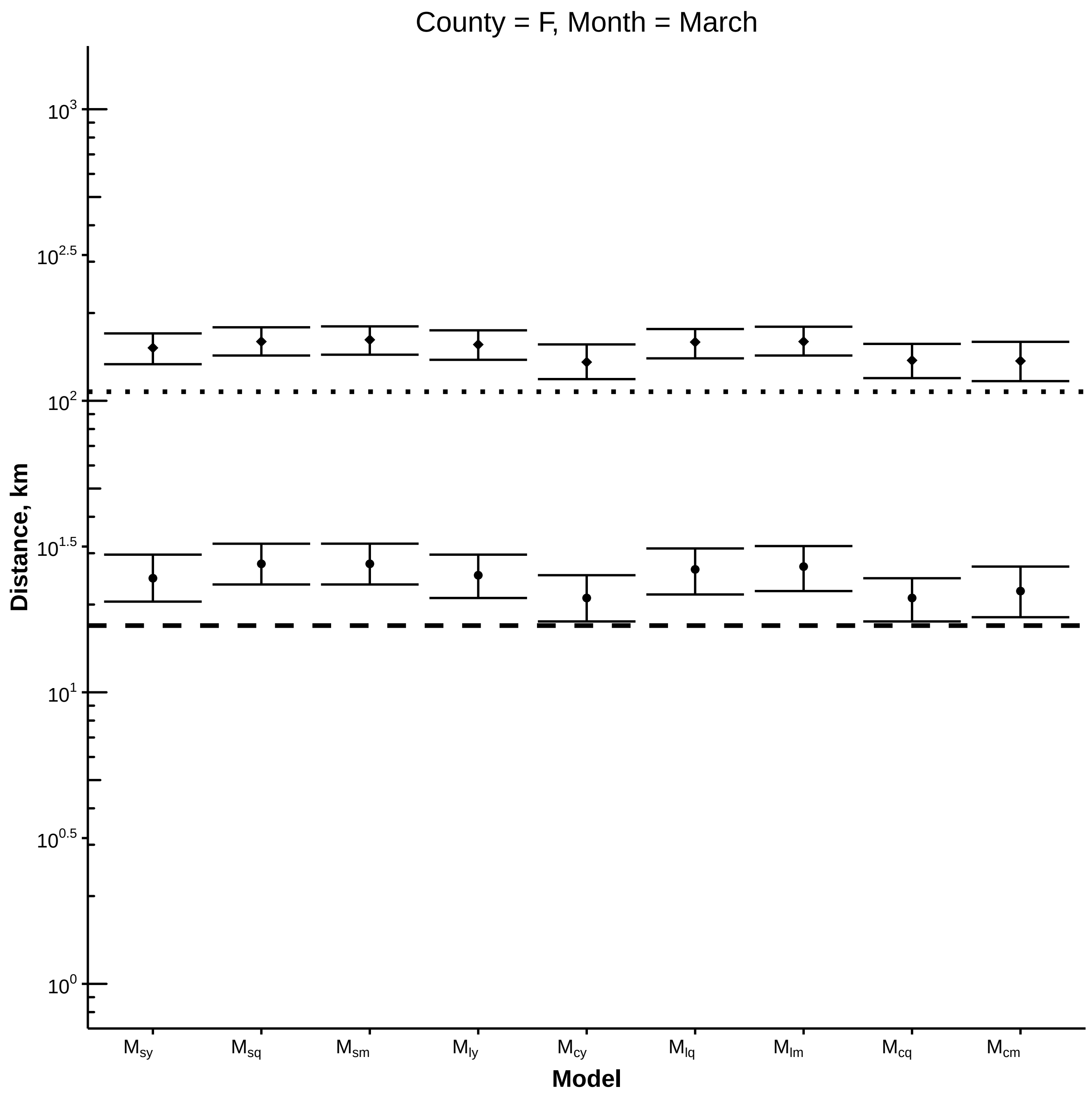
County = F, Month = March
Distance, km
10^3
$10^{2.5}$
10^2
$10^{1.5}$
10^1
$10^{0.5}$
10^0
M_{sy}
M_{sq}
M_{sm}
M_{ly}
M_{cy}
M_{lq}
M_{lm}
M_{cq}
M_{cm}
Model

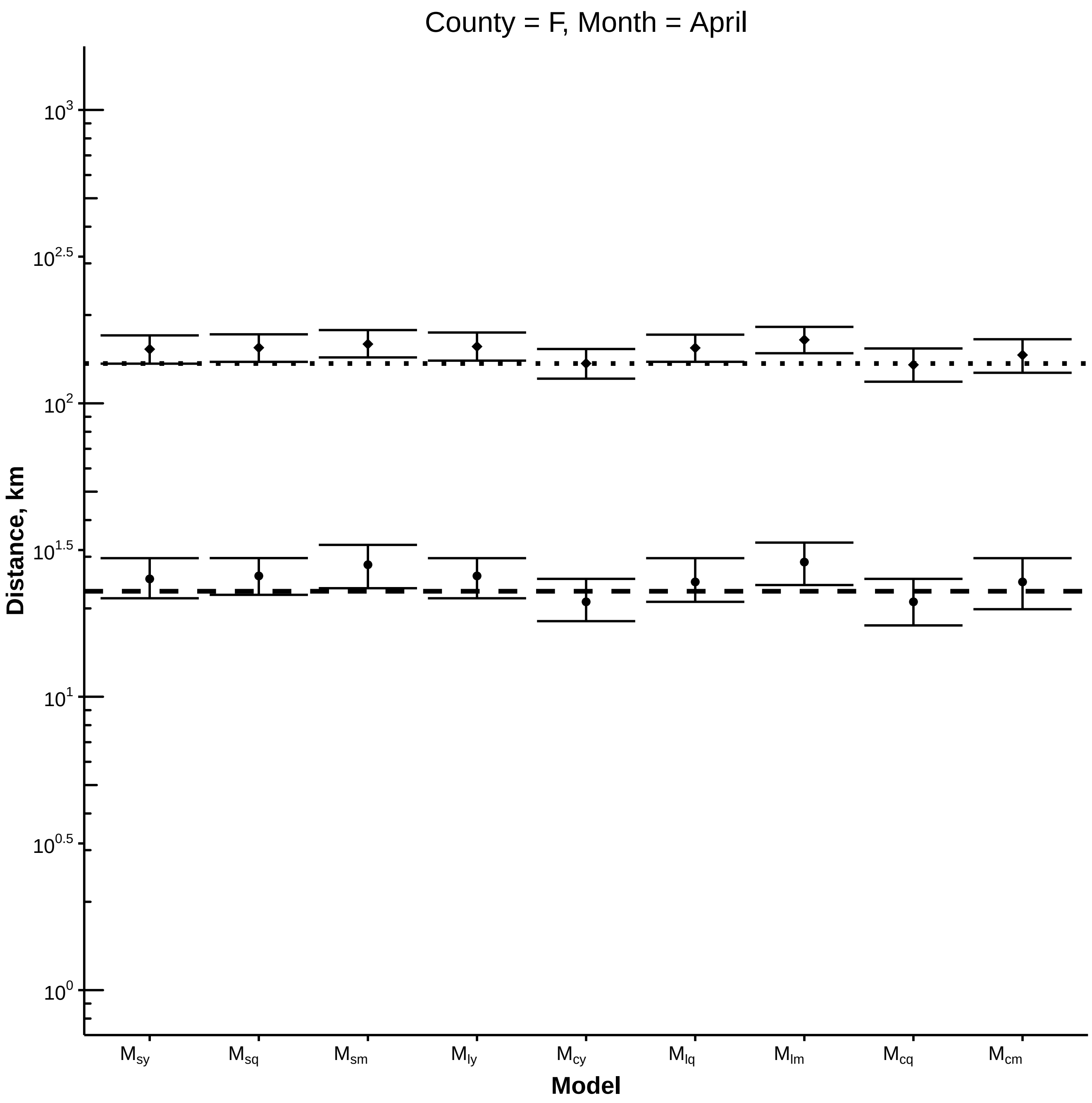
County = F, Month = April
Distance, km
10^3
$10^{2.5}$
10^2
$10^{1.5}$
10^1
$10^{0.5}$
10^0
M_{sy}
M_{sq}
M_{sm}
M_{ly}
M_{cy}
M_{lq}
M_{lm}
M_{cq}
M_{cm}
Model

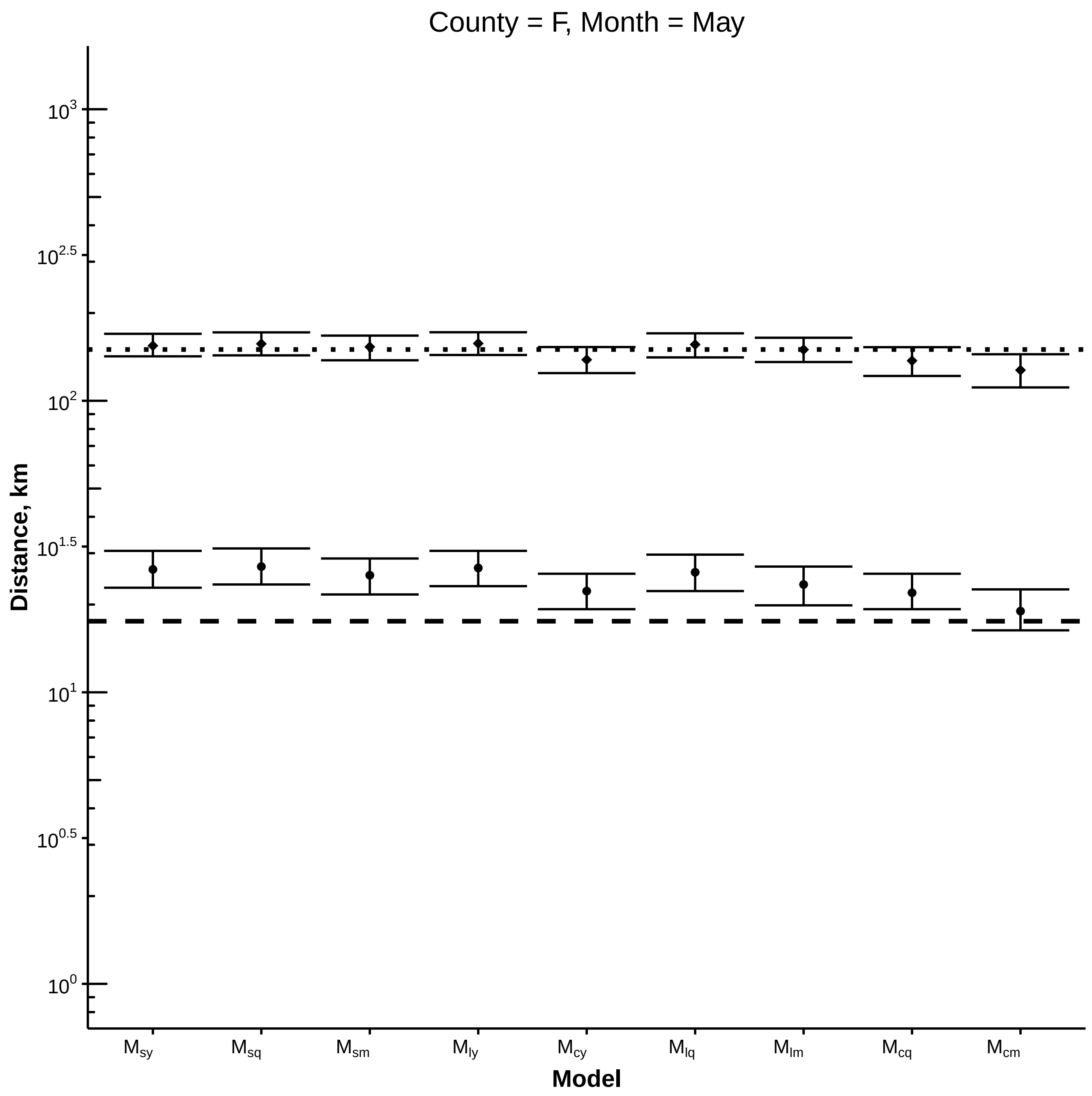

County = F, Month = May
Distance, km
10^3
$10^{2.5}$
10^2
$10^{1.5}$
10^1
$10^{0.5}$
10^0
M_{sy}
M_{sq}
M_{sm}
M_{ly}
M_{cy}
M_{lq}
M_{lm}
M_{cq}
M_{cm}
Model

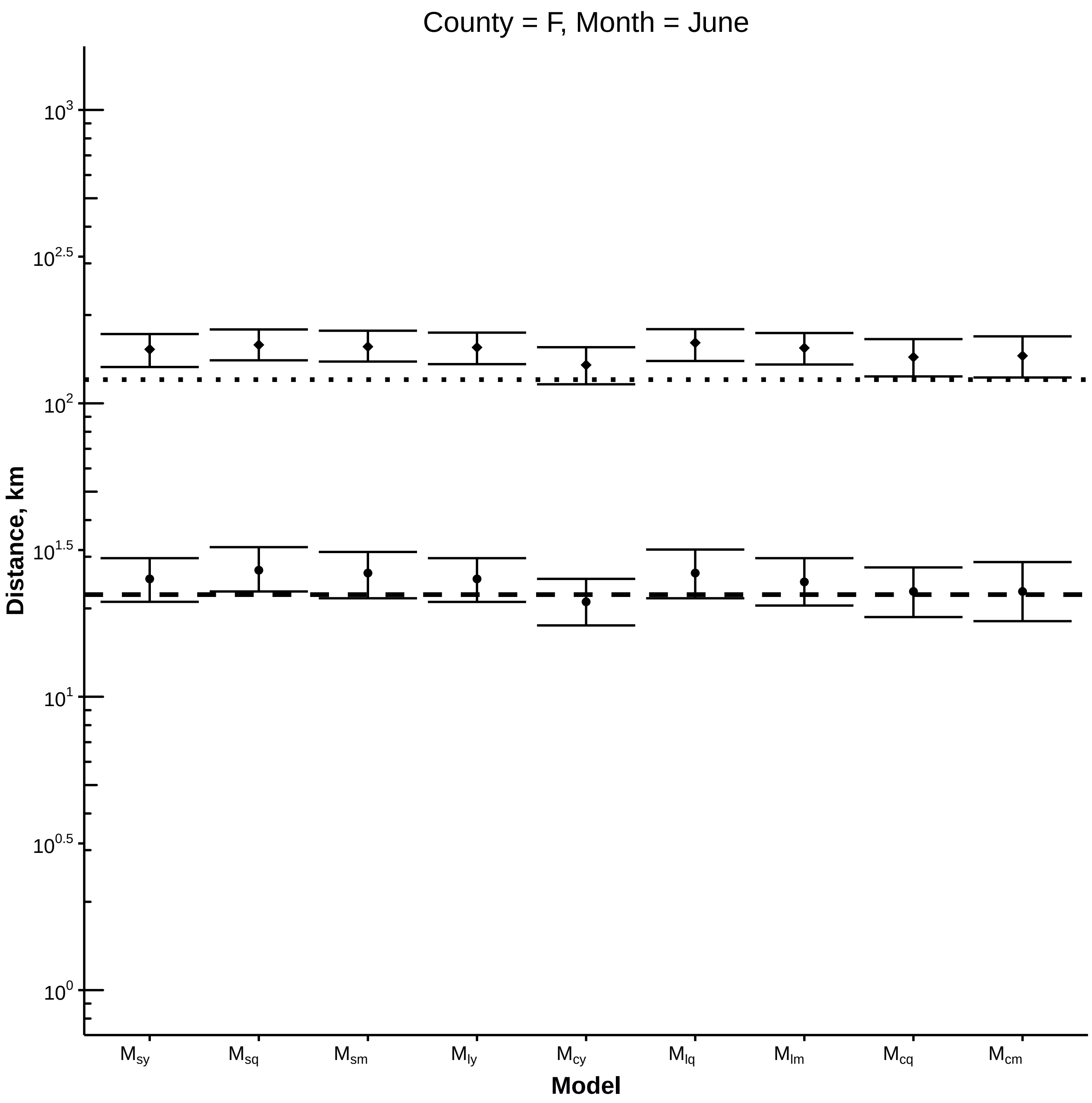

County = F, Month = June
Distance, km
10^3
$10^{2.5}$
10^2
$10^{1.5}$
10^1
$10^{0.5}$
10^0
M_{sy}
M_{sq}
M_{sm}
M_{ly}
M_{cy}
M_{lq}
M_{lm}
M_{cq}
M_{cm}
Model

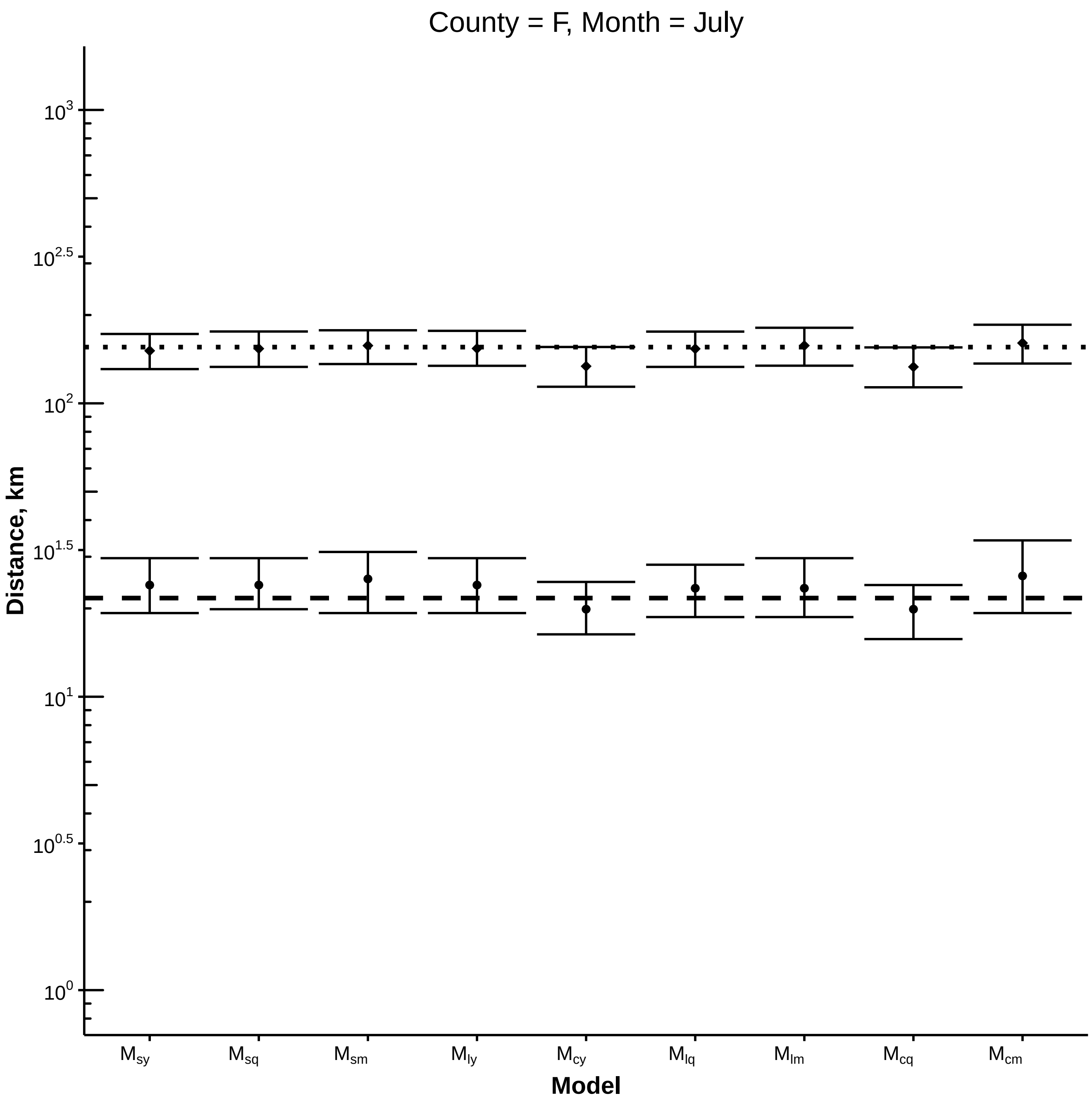
County = F, Month = July
Distance, km
10^{3}
$10^{2.5}$
10^{2}
$10^{1.5}$
10^{1}
$10^{0.5}$
10^{0}
M_{sy}
M_{sq}
M_{sm}
M_{ly}
M_{cy}
M_{lq}
M_{lm}
M_{cq}
M_{cm}
Model

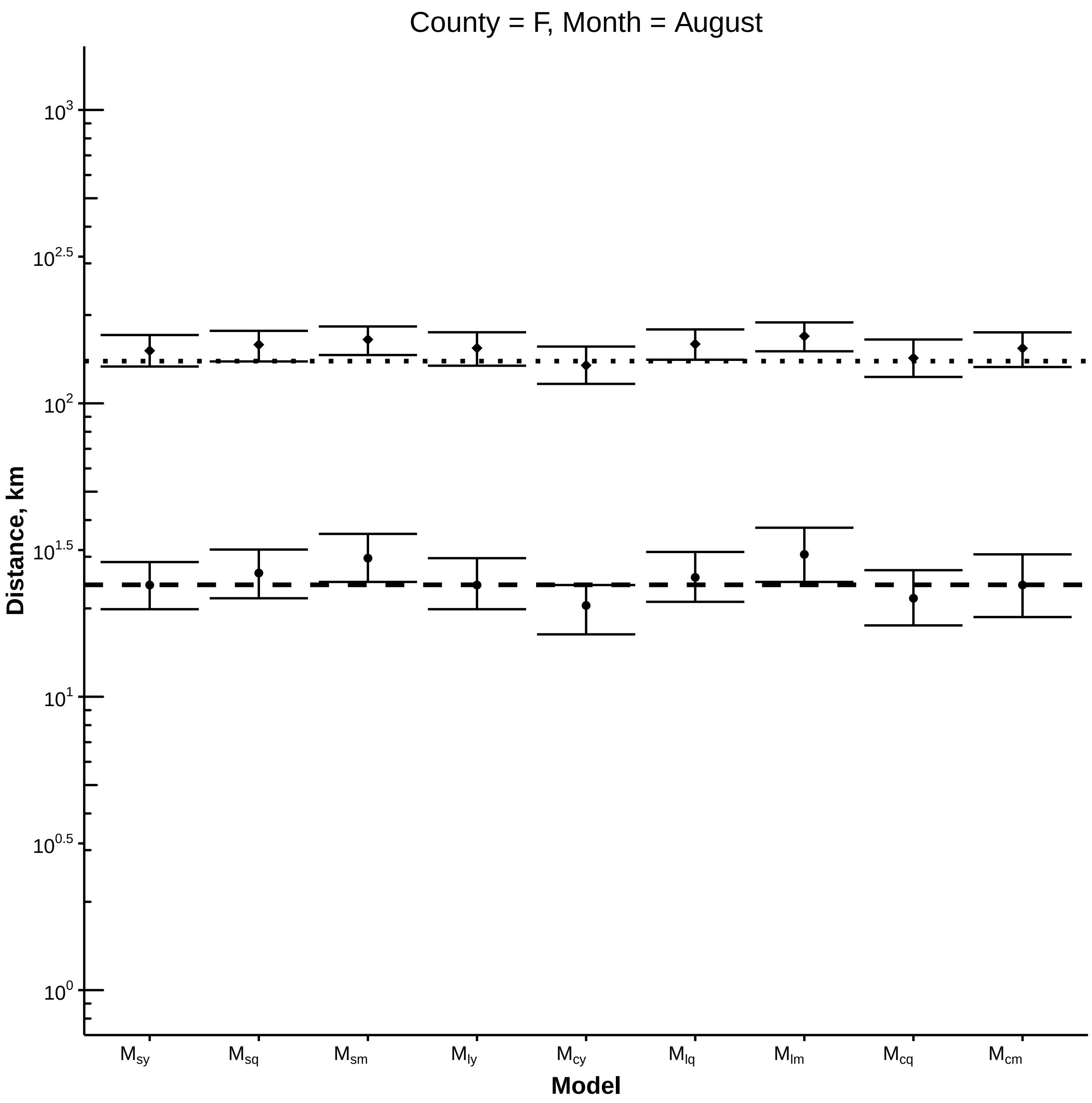

County = F, Month = August
Distance, km
10^{3}
$10^{2.5}$
10^{2}
$10^{1.5}$
10^{1}
$10^{0.5}$
10^{0}
M_{sy}
M_{sq}
M_{sm}
M_{ly}
M_{cy}
M_{lq}
M_{lm}
M_{cq}
M_{cm}
Model

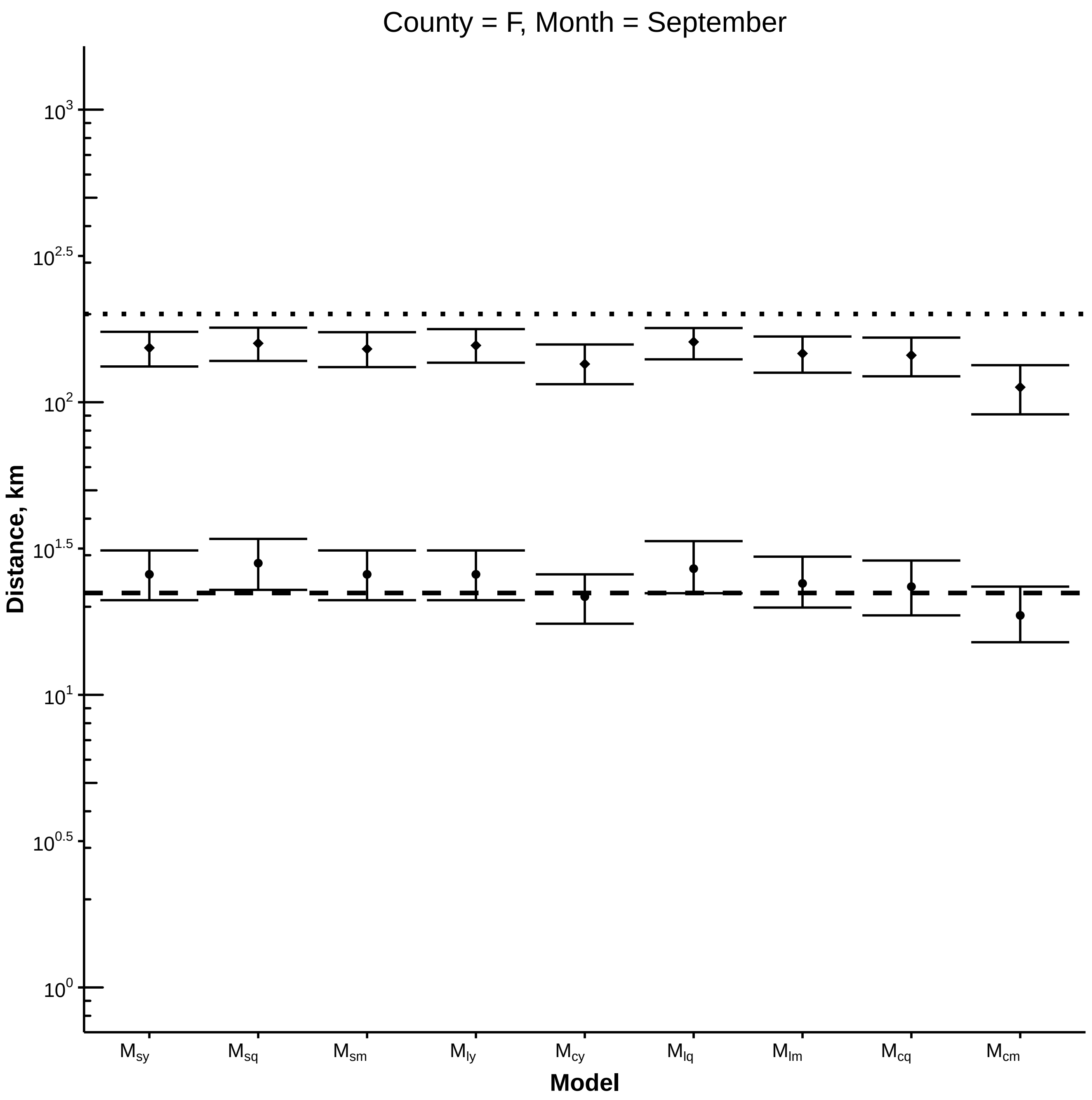

County = F, Month = September
Distance, km
10^3
$10^{2.5}$
10^2
$10^{1.5}$
10^1
$10^{0.5}$
10^0
M_{sy}
M_{sq}
M_{sm}
M_{ly}
M_{cy}
M_{lq}
M_{lm}
M_{cq}
M_{cm}
Model

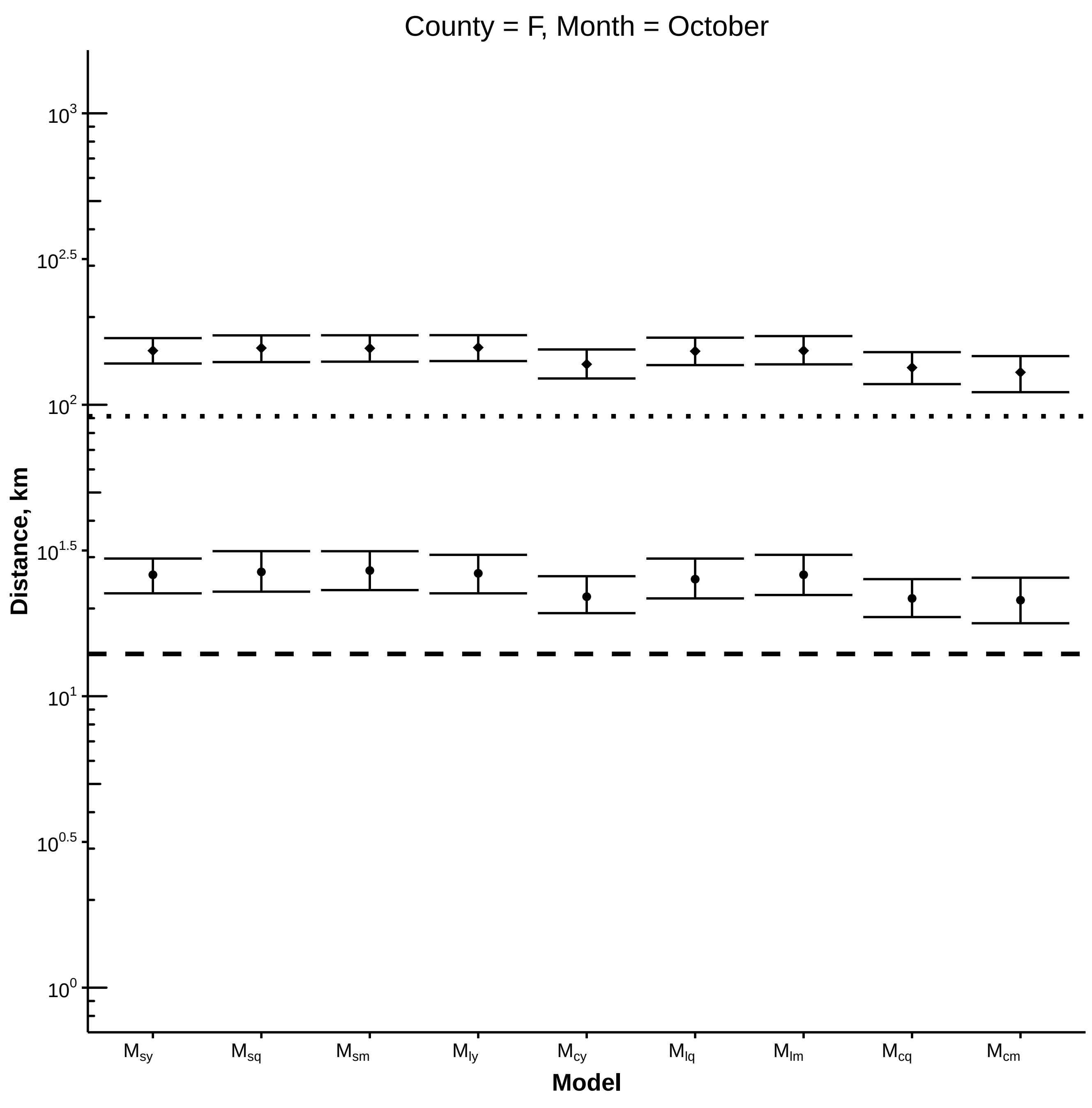
County = F, Month = October
Distance, km
10^3
$10^{2.5}$
10^2
$10^{1.5}$
10^1
$10^{0.5}$
10^0
M_{sy}
M_{sq}
M_{sm}
M_{ly}
M_{cy}
M_{lq}
M_{lm}
M_{cq}
M_{cm}
Model

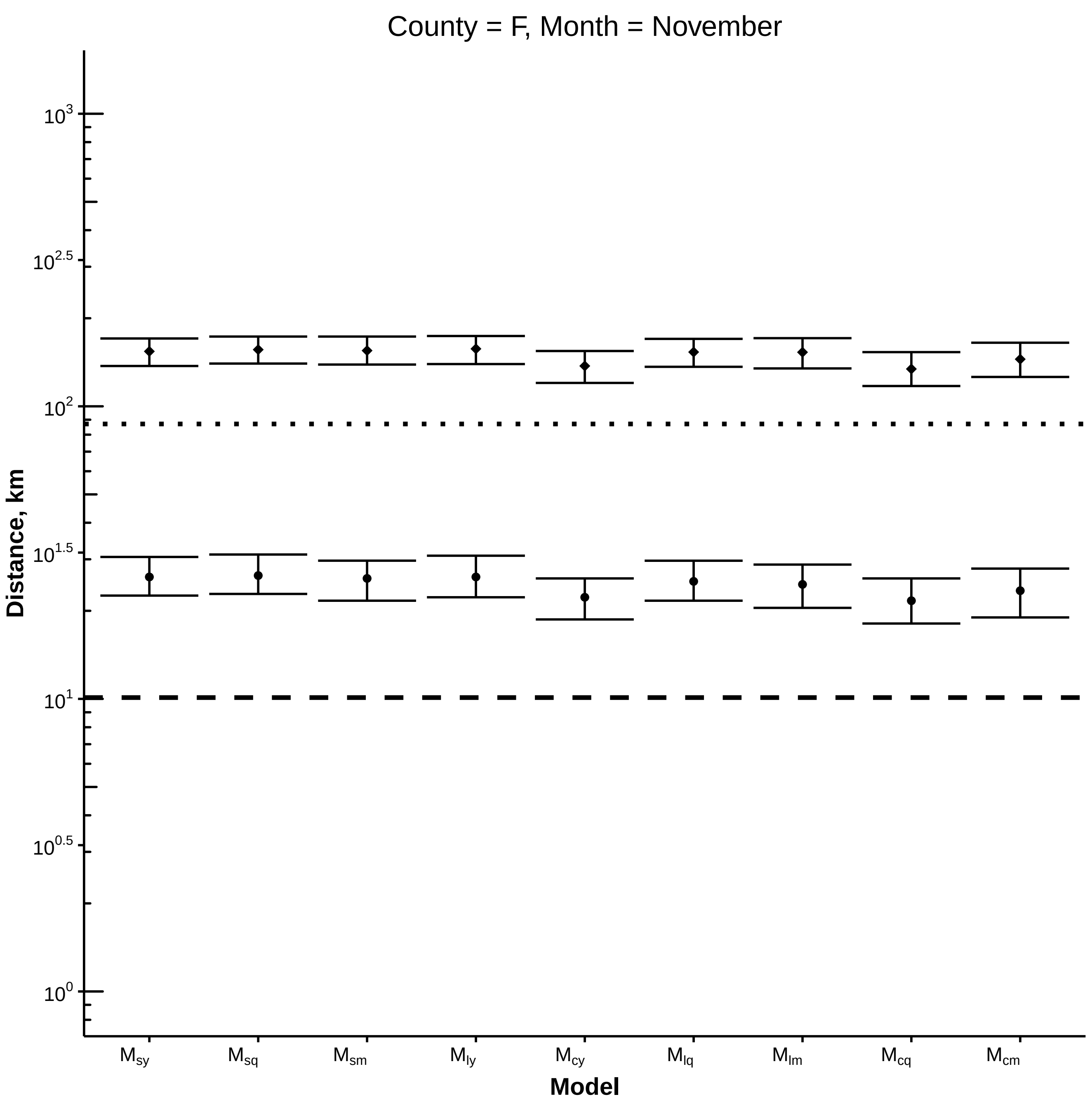

County = F, Month = November
Distance, km
Model
10^{3}
$10^{2.5}$
10^{2}
$10^{1.5}$
10^{1}
$10^{0.5}$
10^{0}
M_{sy}
M_{sq}
M_{sm}
M_{ly}
M_{cy}
M_{lq}
M_{lm}
M_{cq}
M_{cm}

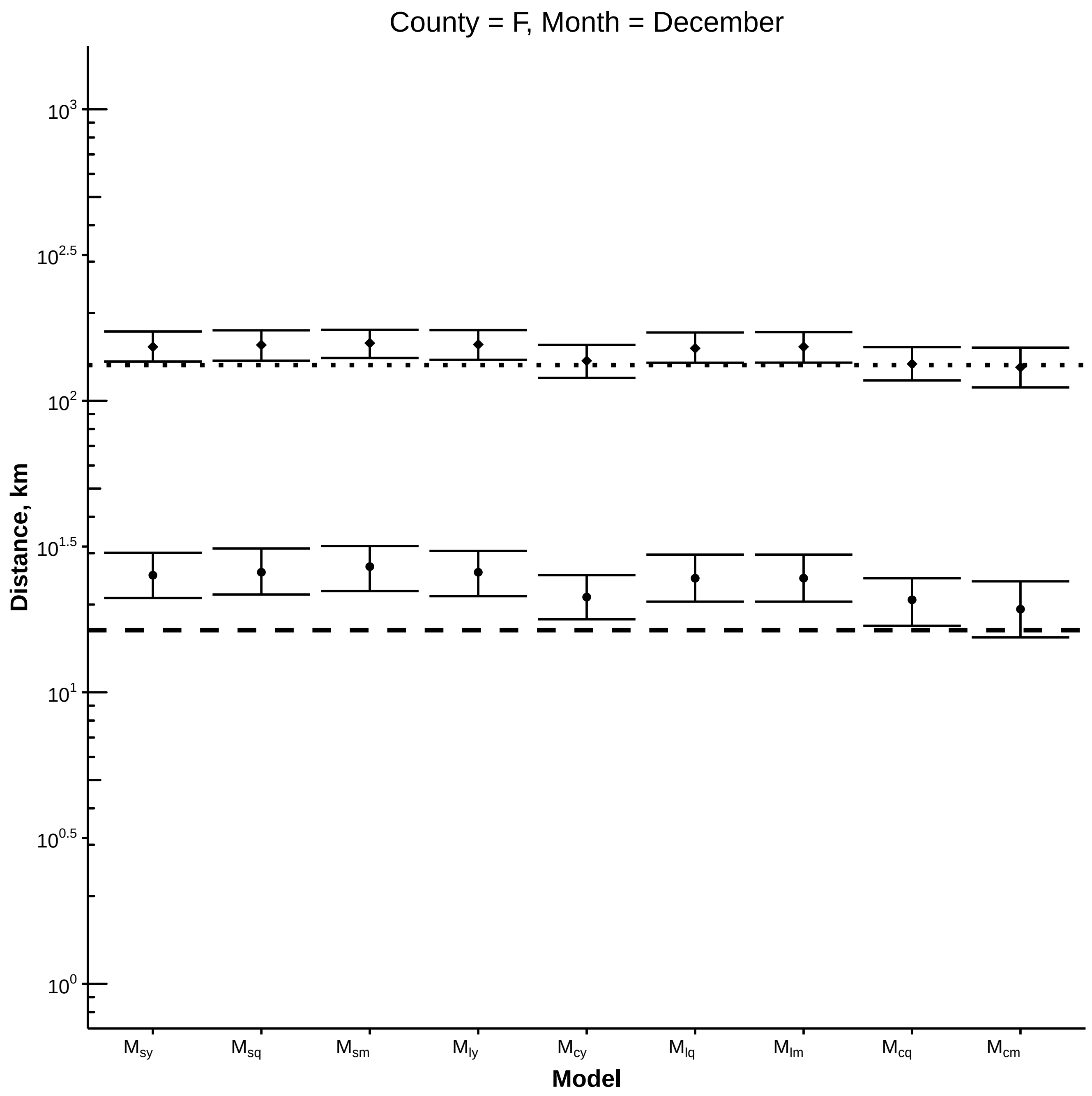
County = F, Month = December
Distance, km
10^3
$10^{2.5}$
10^2
$10^{1.5}$
10^1
$10^{0.5}$
10^0
M_{sy}
M_{sq}
M_{sm}
M_{ly}
M_{cy}
M_{lq}
M_{lm}
M_{cq}
M_{cm}
Model

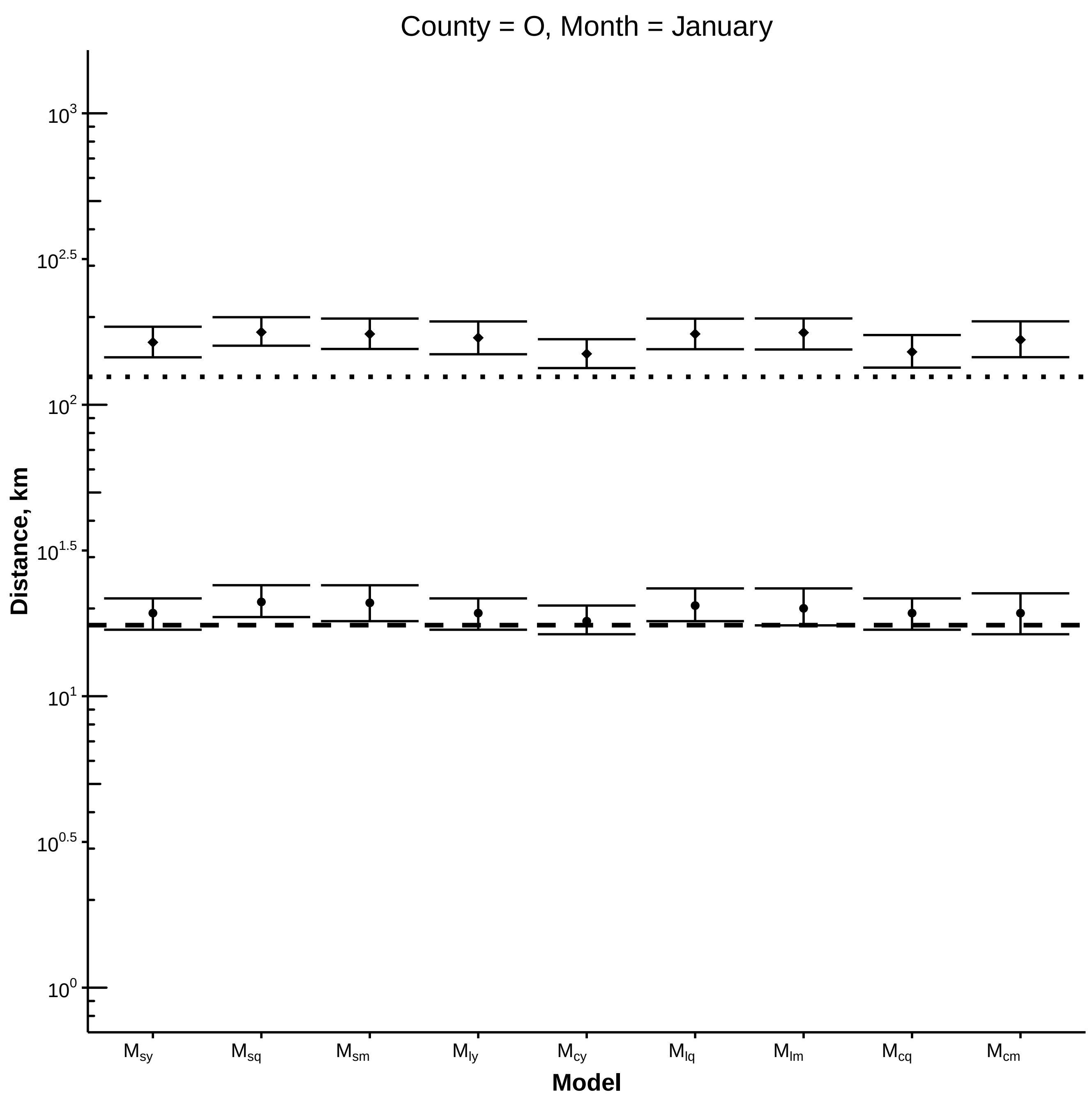

County = O, Month = January
Distance, km
10^3
10^2.5
10^2
10^1.5
10^1
10^0.5
10^0
Msy
Msq
Msm
Mly
Mcy
Mlq
Mlm
Mcq
Mcm
Model

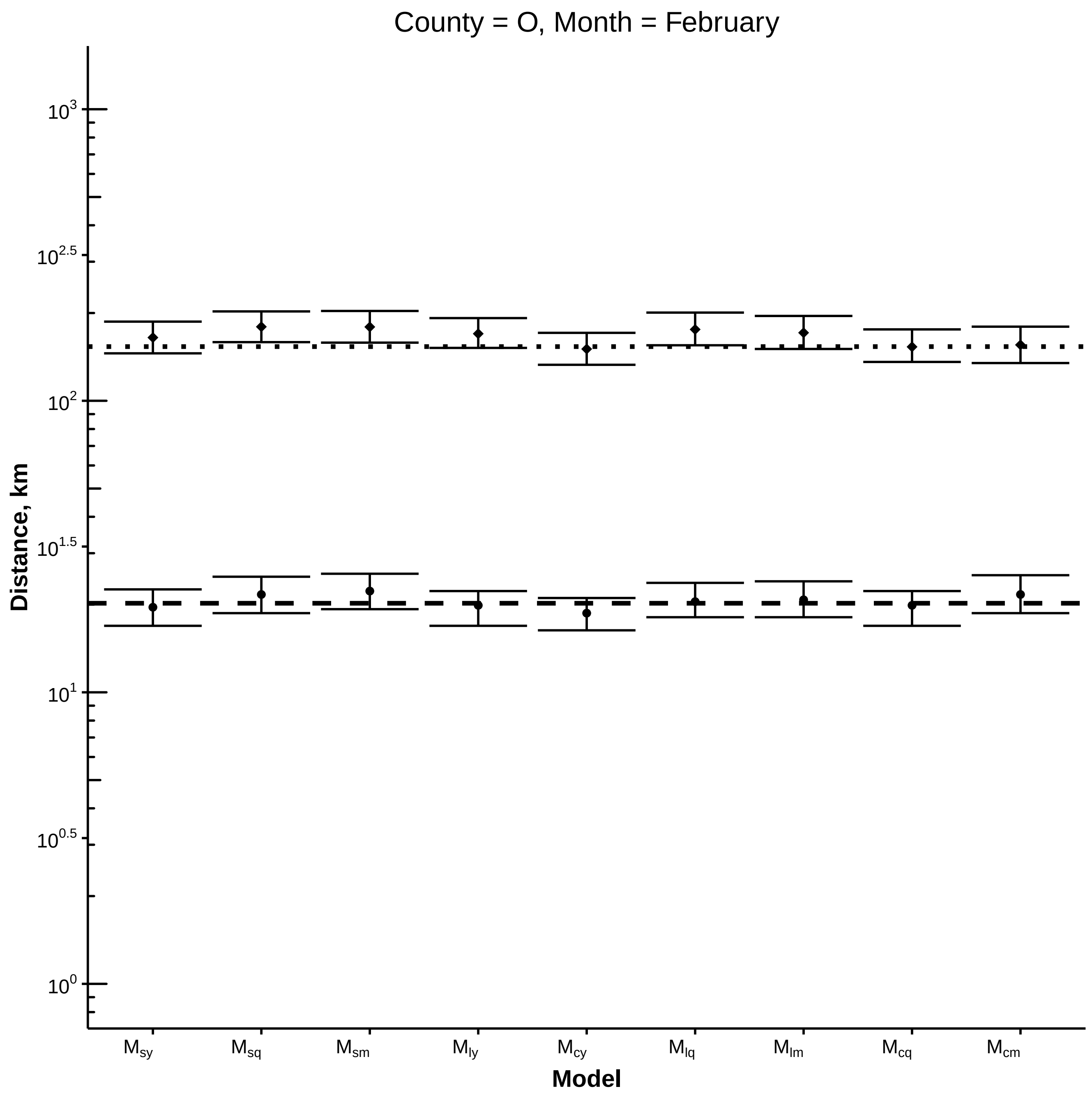
County = O, Month = February
Distance, km
10^3
$10^{2.5}$
10^2
$10^{1.5}$
10^1
$10^{0.5}$
10^0
M_{sy}
M_{sq}
M_{sm}
M_{ly}
M_{cy}
M_{lq}
M_{lm}
M_{cq}
M_{cm}
Model

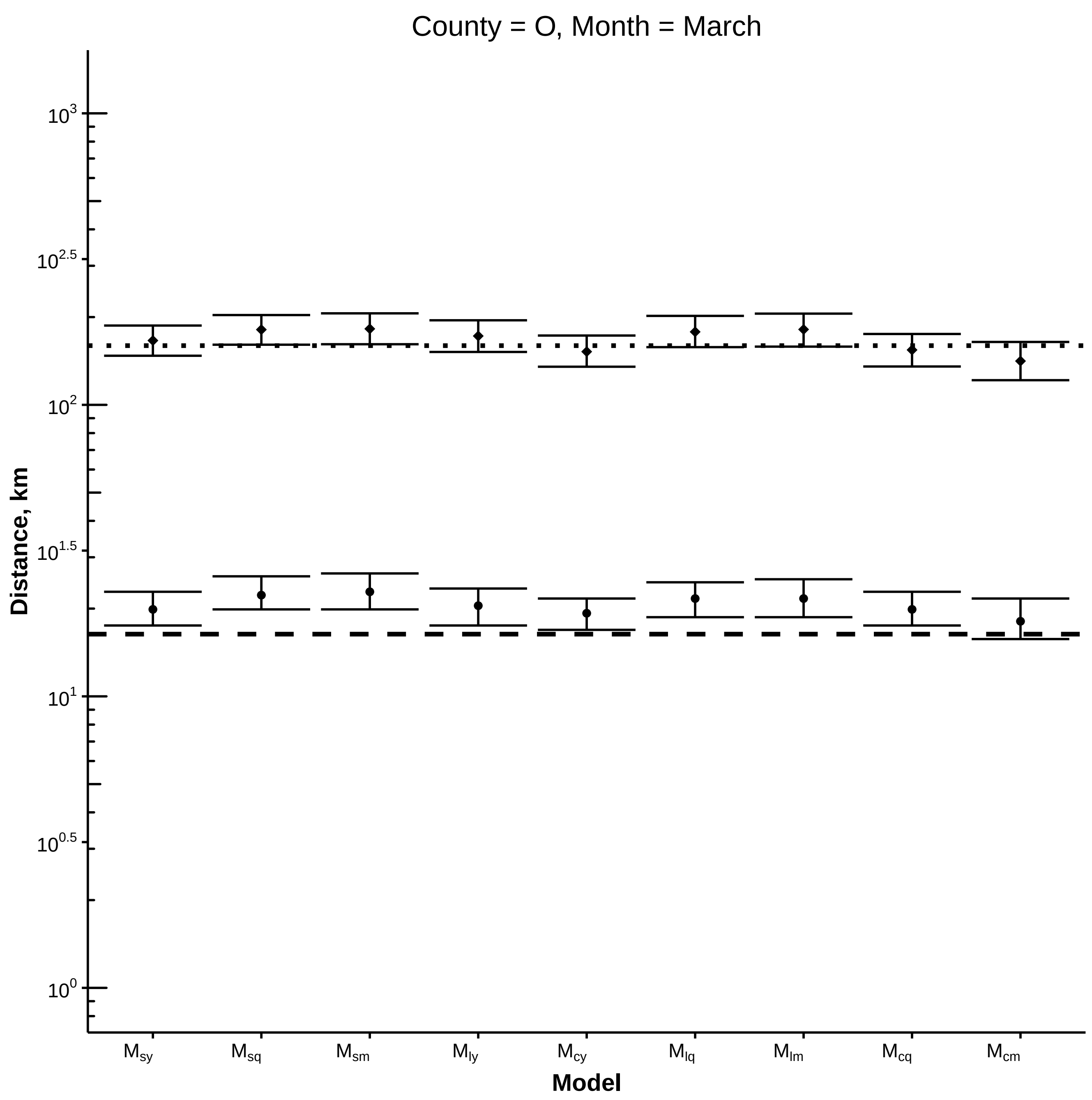

County = O, Month = March
Distance, km
10^3
$10^{2.5}$
10^2
$10^{1.5}$
10^1
$10^{0.5}$
10^0
M_{sy}
M_{sq}
M_{sm}
M_{ly}
M_{cy}
M_{lq}
M_{lm}
M_{cq}
M_{cm}
Model

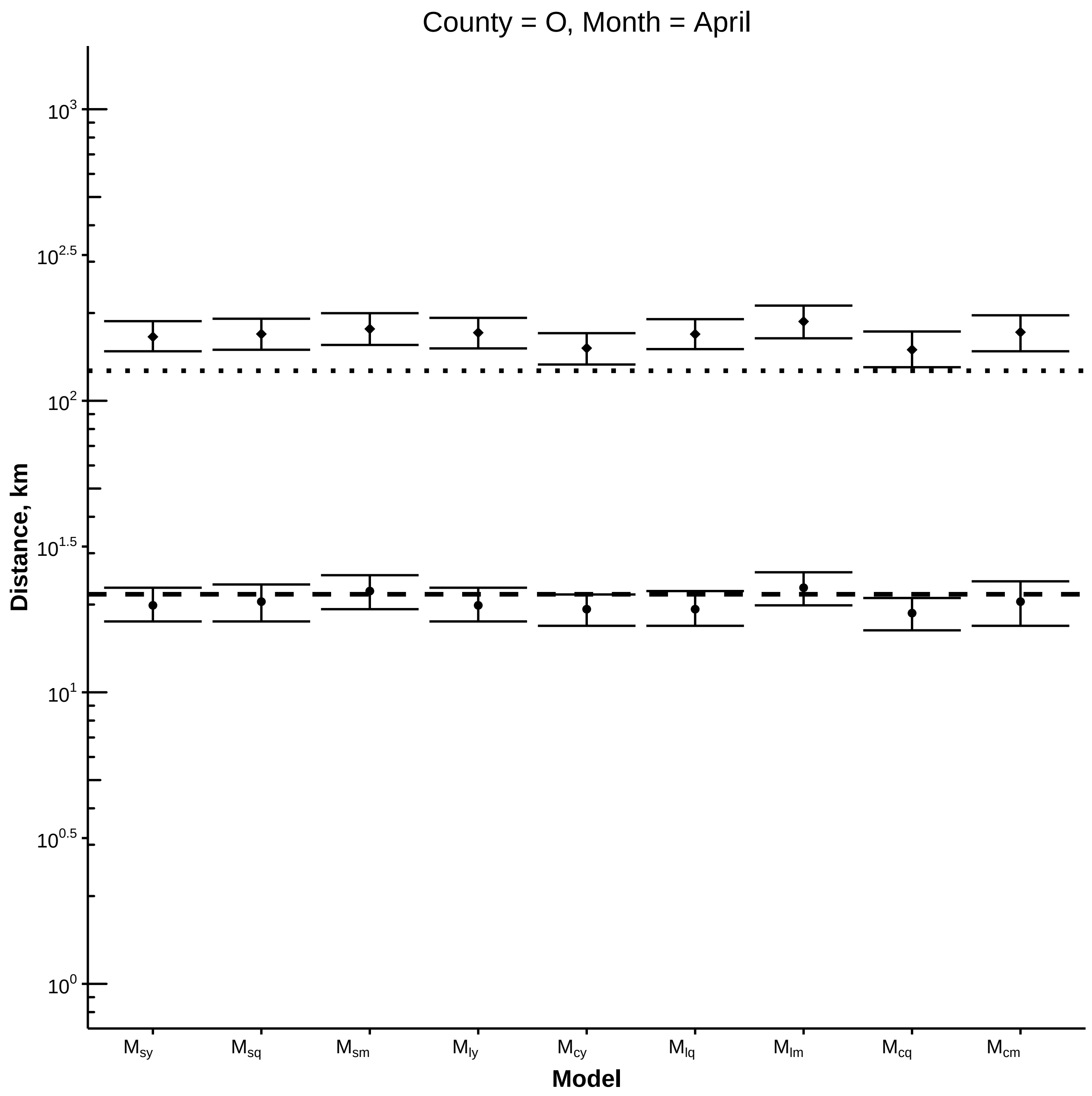
County = O, Month = April
Distance, km
10^{3}
$10^{2.5}$
10^{2}
$10^{1.5}$
10^{1}
$10^{0.5}$
10^{0}
M_{sy}
M_{sq}
M_{sm}
M_{ly}
M_{cy}
M_{lq}
M_{lm}
M_{cq}
M_{cm}
Model

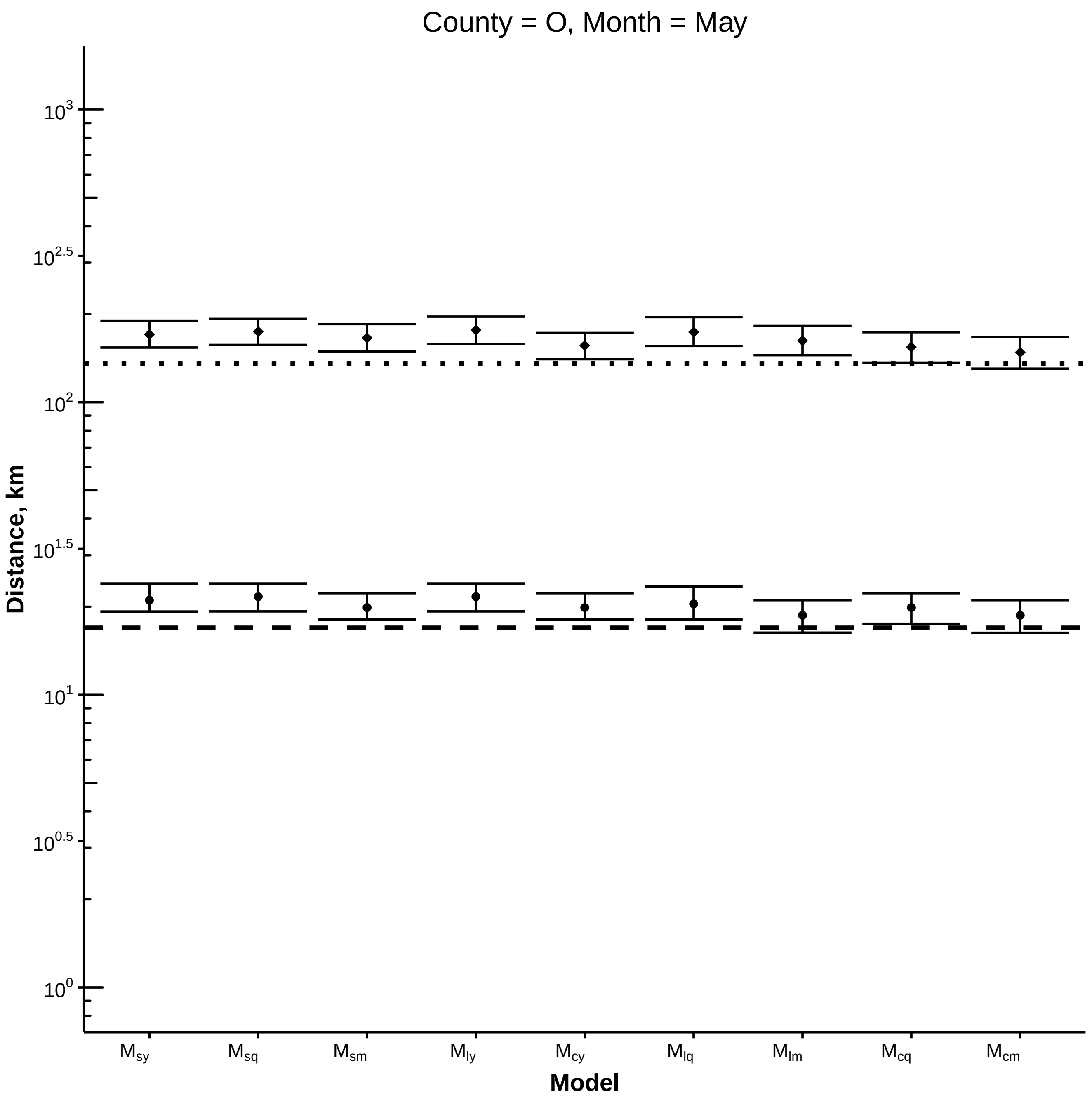
County = O, Month = May
Distance, km
10^3
$10^{2.5}$
10^2
$10^{1.5}$
10^1
$10^{0.5}$
10^0
M_{sy}
M_{sq}
M_{sm}
M_{ly}
M_{cy}
M_{lq}
M_{lm}
M_{cq}
M_{cm}
Model

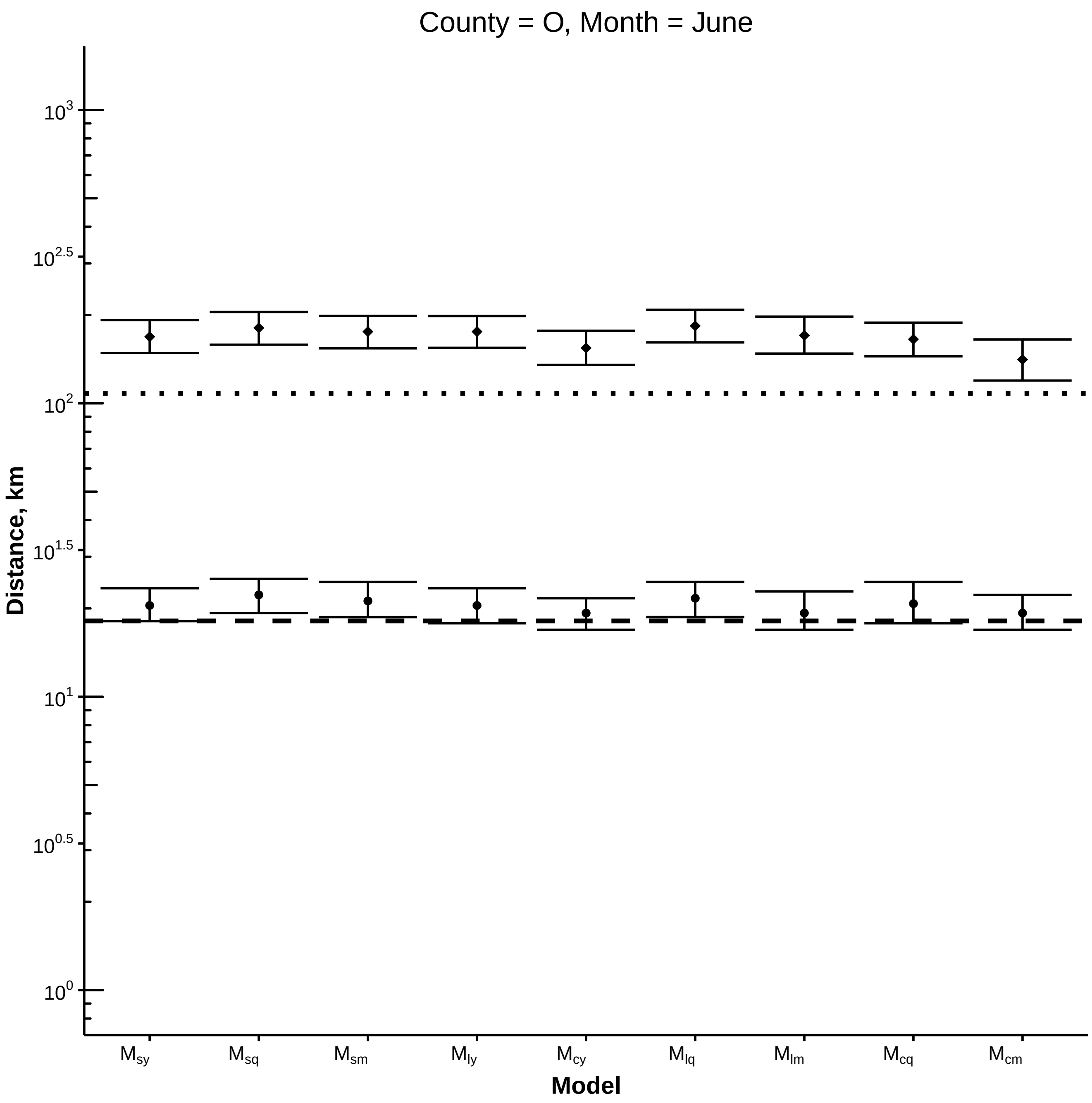
County = O, Month = June
Distance, km
10^3
$10^{2.5}$
10^2
$10^{1.5}$
10^1
$10^{0.5}$
10^0
M_{sy} M_{sq} M_{sm} M_{ly} M_{cy} M_{lq} M_{lm} M_{cq} M_{cm}
Model

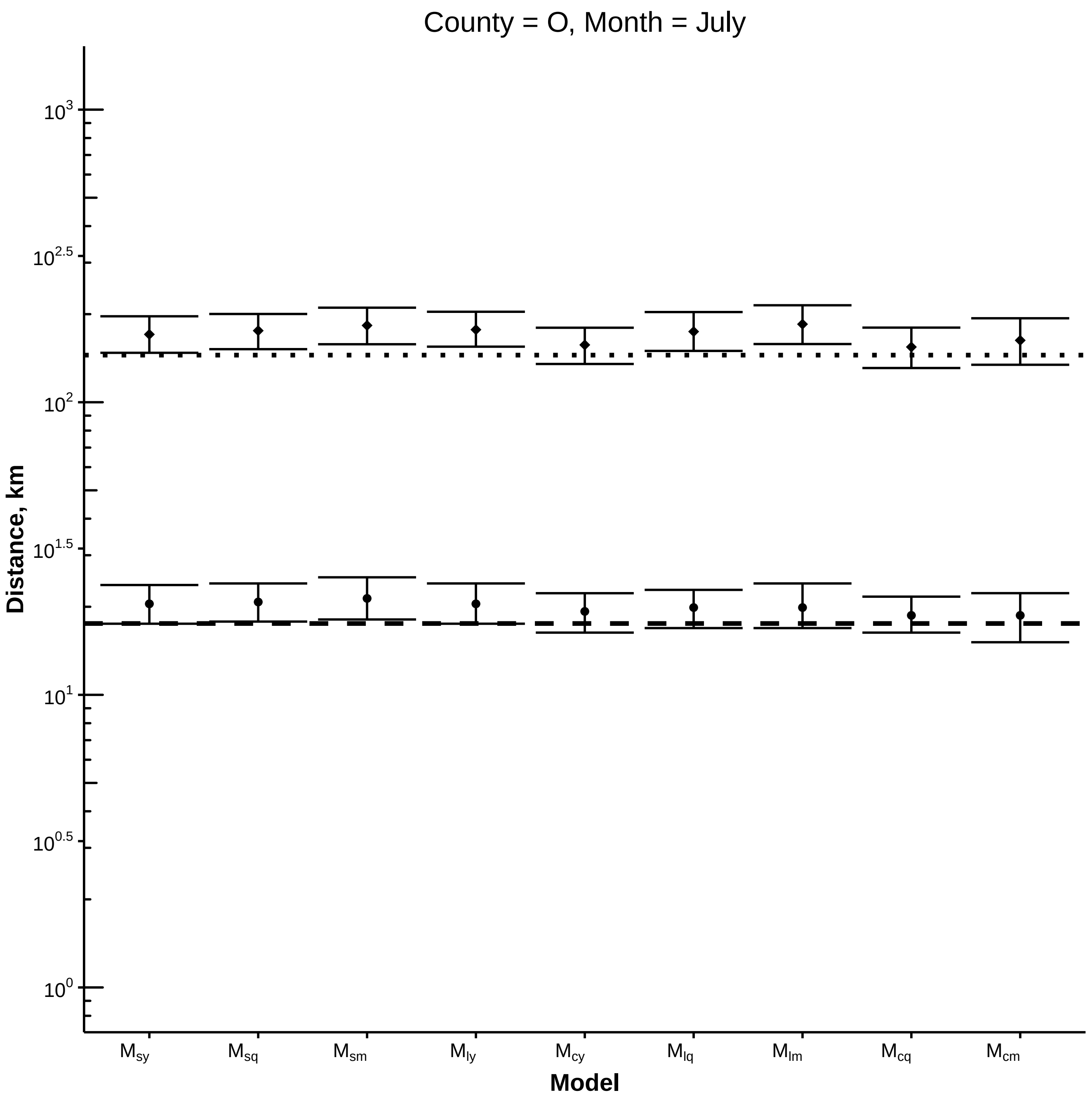

County = O, Month = July
Distance, km
10^3
10^2.5
10^2
10^1.5
10^1
10^0.5
10^0
M_sy
M_sq
M_sm
M_ly
M_cy
M_lq
M_lm
M_cq
M_cm
Model

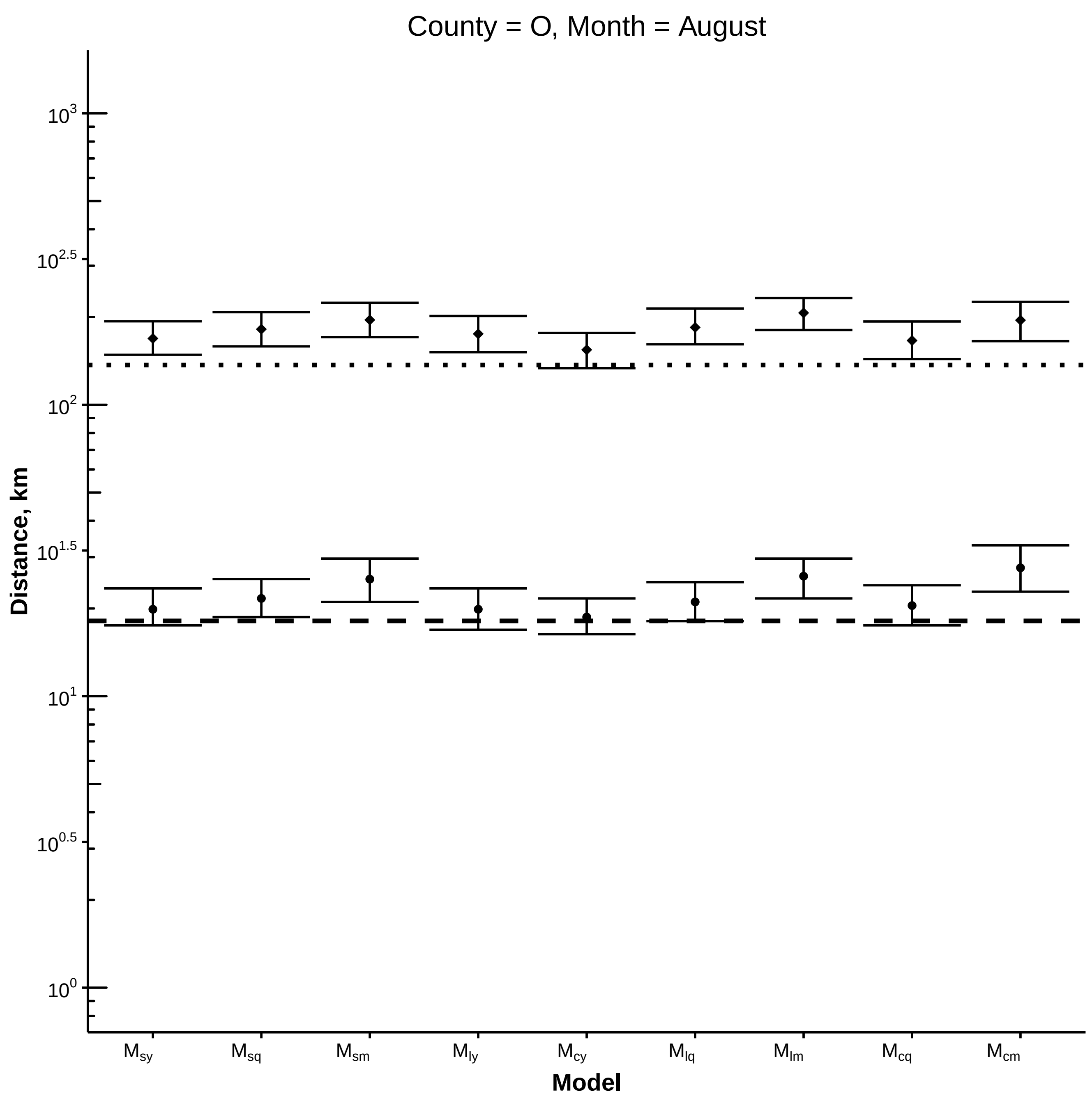
County = O, Month = August
Distance, km
10^3
$10^{2.5}$
10^2
$10^{1.5}$
10^1
$10^{0.5}$
10^0
M_{sy}
M_{sq}
M_{sm}
M_{ly}
M_{cy}
M_{lq}
M_{lm}
M_{cq}
M_{cm}
Model

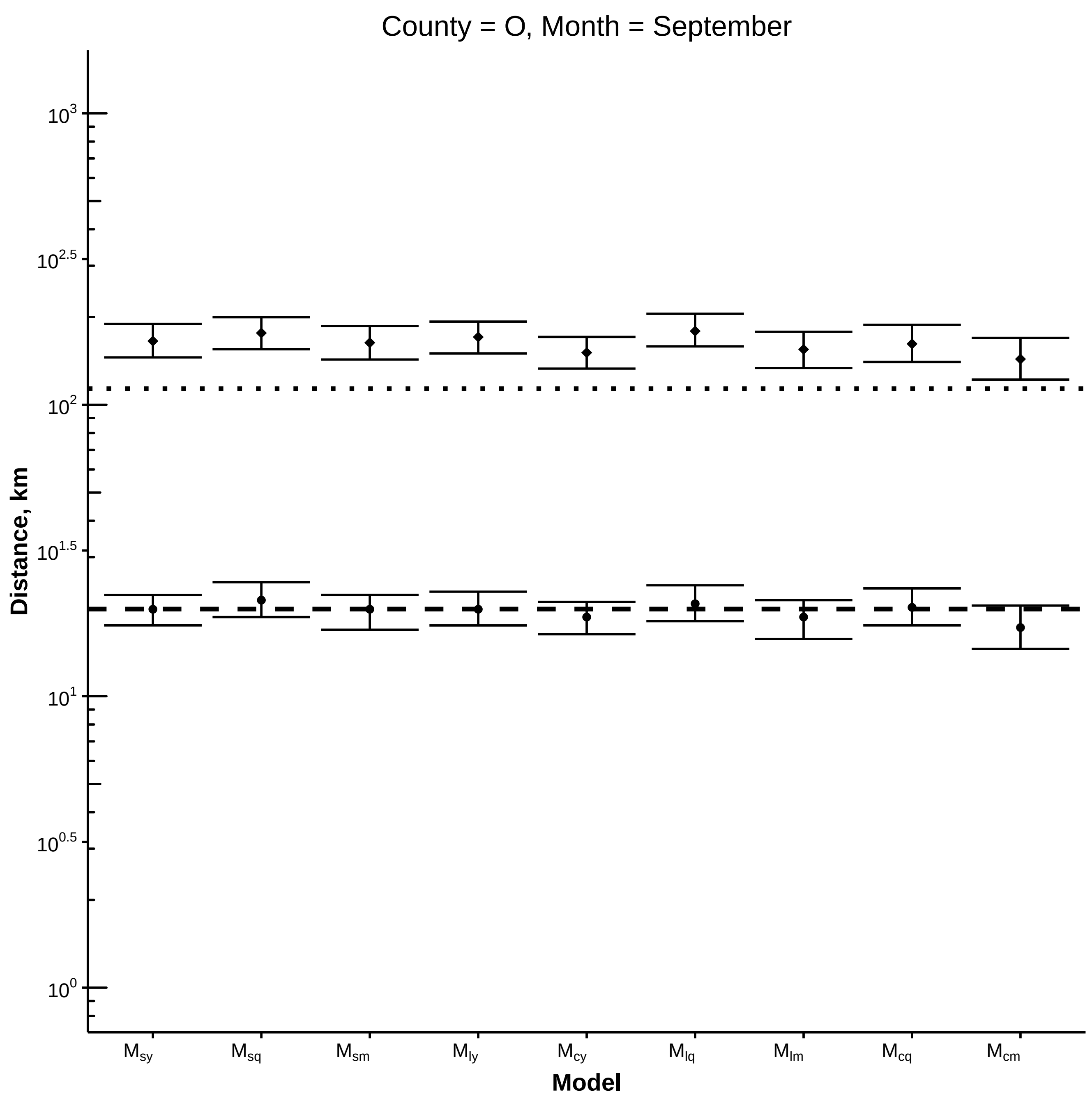

County = O, Month = September
Distance, km
10^3
$10^{2.5}$
10^2
$10^{1.5}$
10^1
$10^{0.5}$
10^0
M_{sy}
M_{sq}
M_{sm}
M_{ly}
M_{cy}
M_{lq}
M_{lm}
M_{cq}
M_{cm}
Model

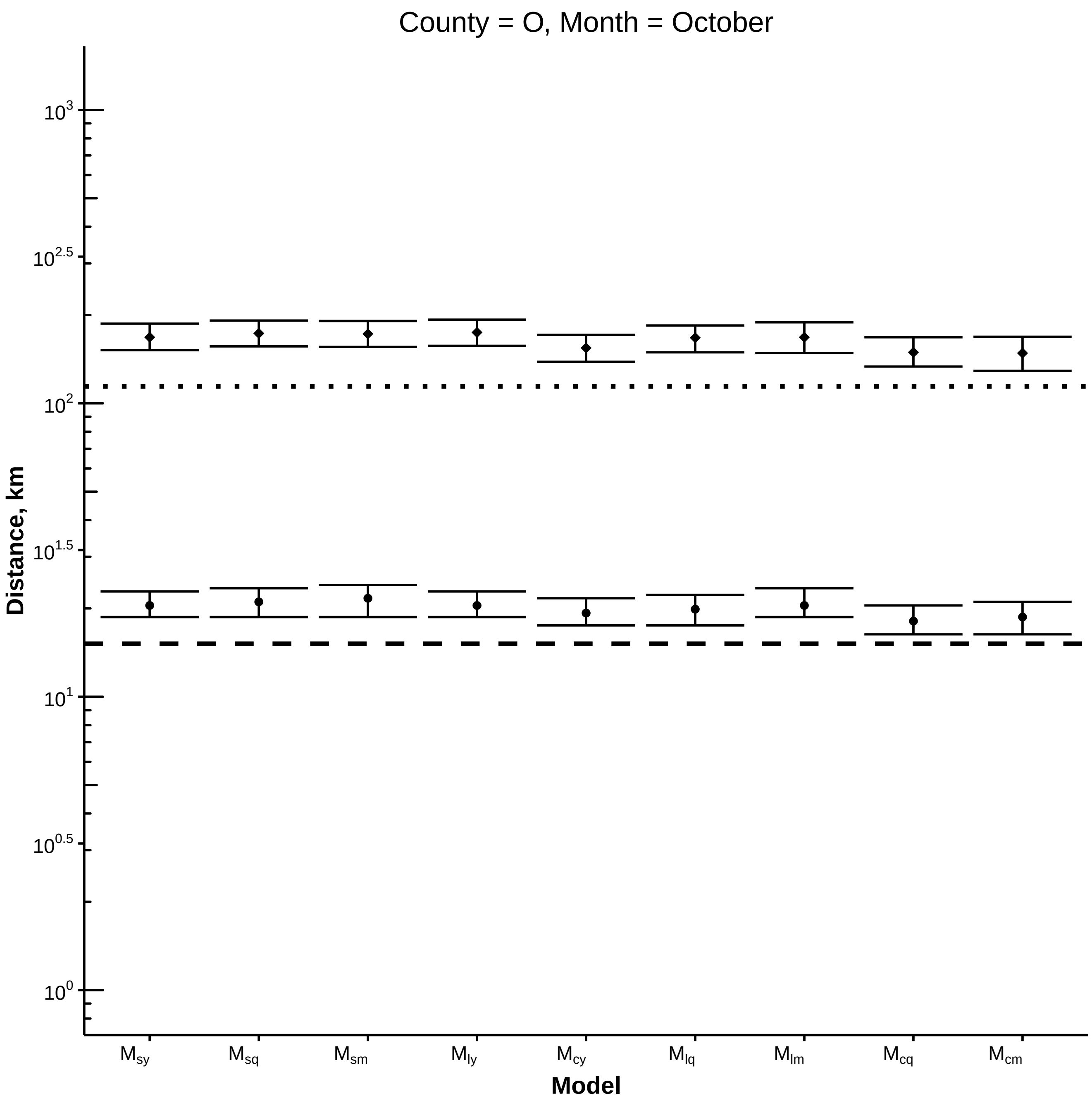

County = O, Month = October
10^3
$10^{2.5}$
10^2
$10^{1.5}$
10^1
$10^{0.5}$
10^0
Distance, km
M_{sy}
M_{sq}
M_{sm}
M_{ly}
M_{cy}
M_{lq}
M_{lm}
M_{cq}
M_{cm}
Model

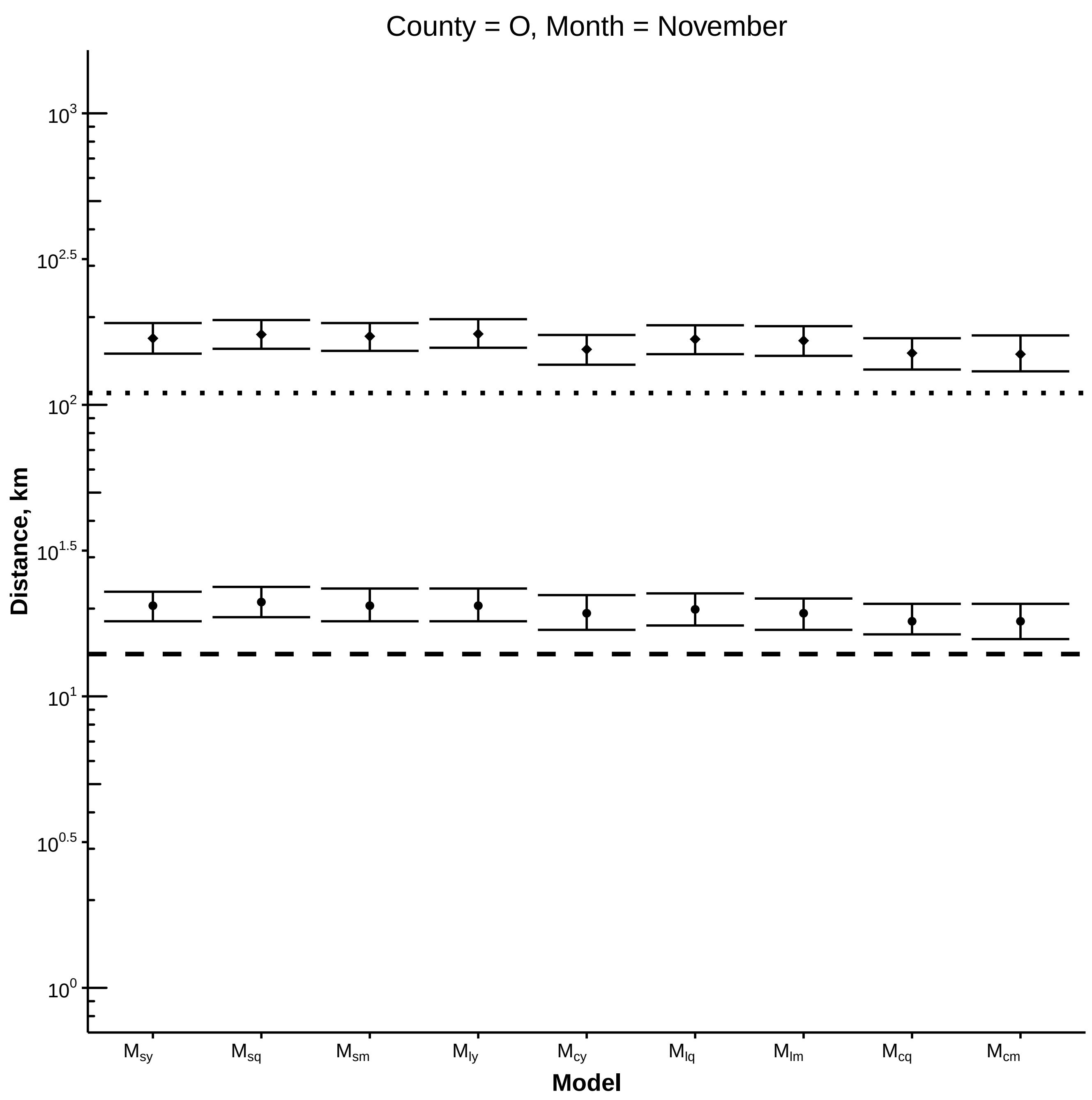
County = O, Month = November
Distance, km
10^3
$10^{2.5}$
10^2
$10^{1.5}$
10^1
$10^{0.5}$
10^0
M_{sy}
M_{sq}
M_{sm}
M_{ly}
M_{cy}
M_{lq}
M_{lm}
M_{cq}
M_{cm}
Model

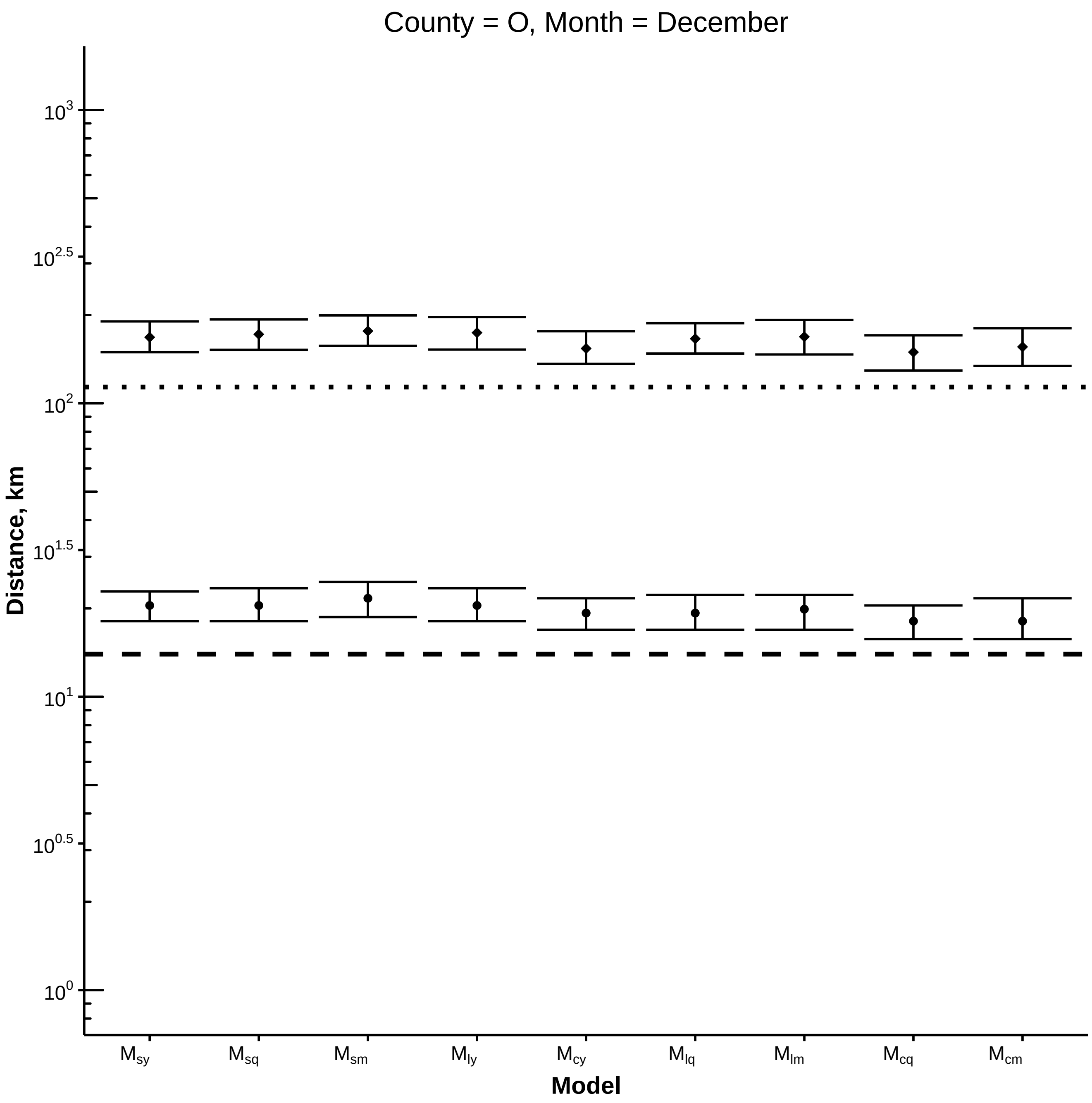

County = O, Month = December
Distance, km
Model
10^3
$10^{2.5}$
10^2
$10^{1.5}$
10^1
$10^{0.5}$
10^0
M_{sy}
M_{sq}
M_{sm}
M_{ly}
M_{cy}
M_{lq}
M_{lm}
M_{cq}
M_{cm}

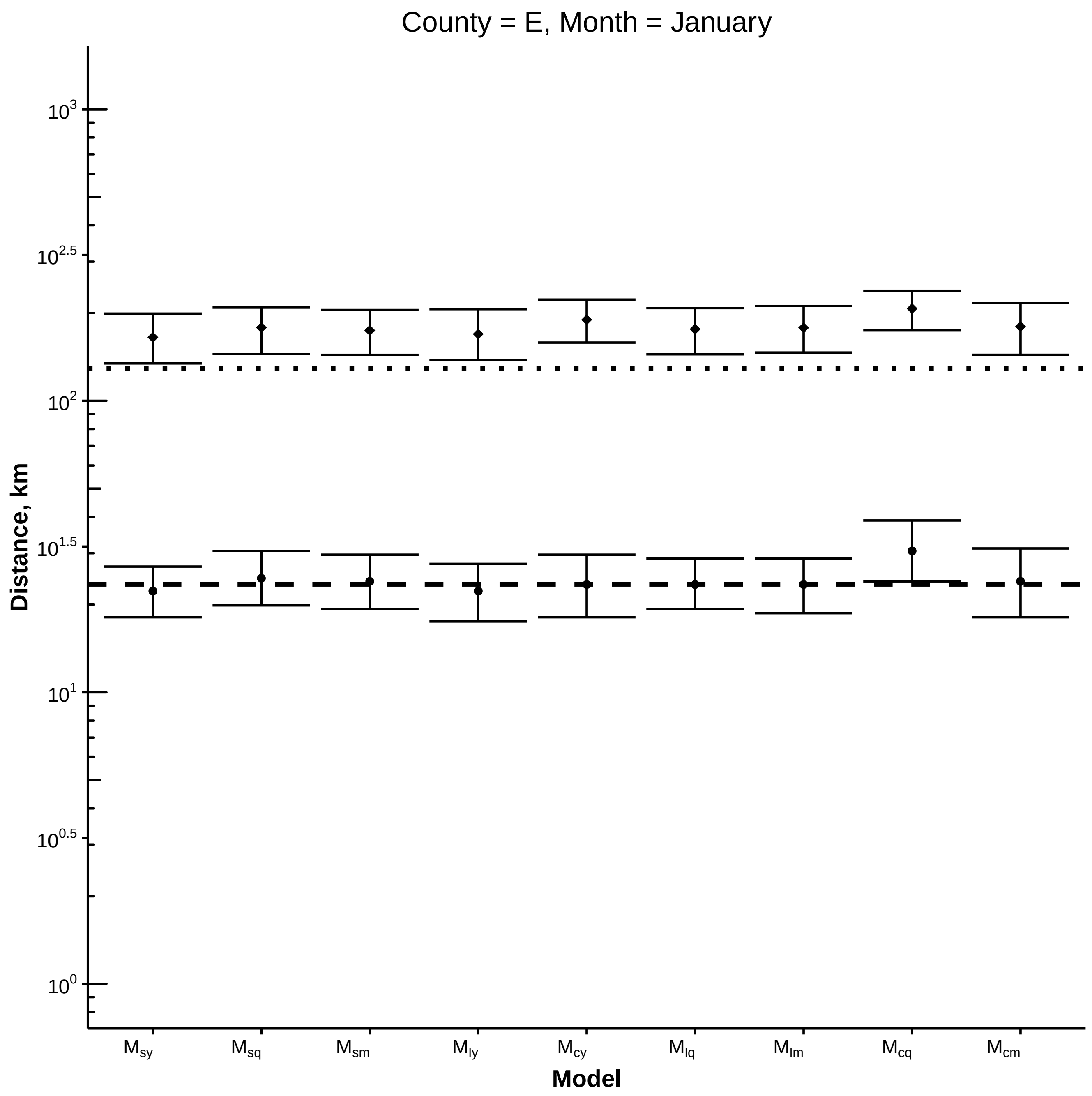

County = E, Month = January
Distance, km
Model
10^3
$10^{2.5}$
10^2
$10^{1.5}$
10^1
$10^{0.5}$
10^0
M_{sy}
M_{sq}
M_{sm}
M_{ly}
M_{cy}
M_{lq}
M_{lm}
M_{cq}
M_{cm}

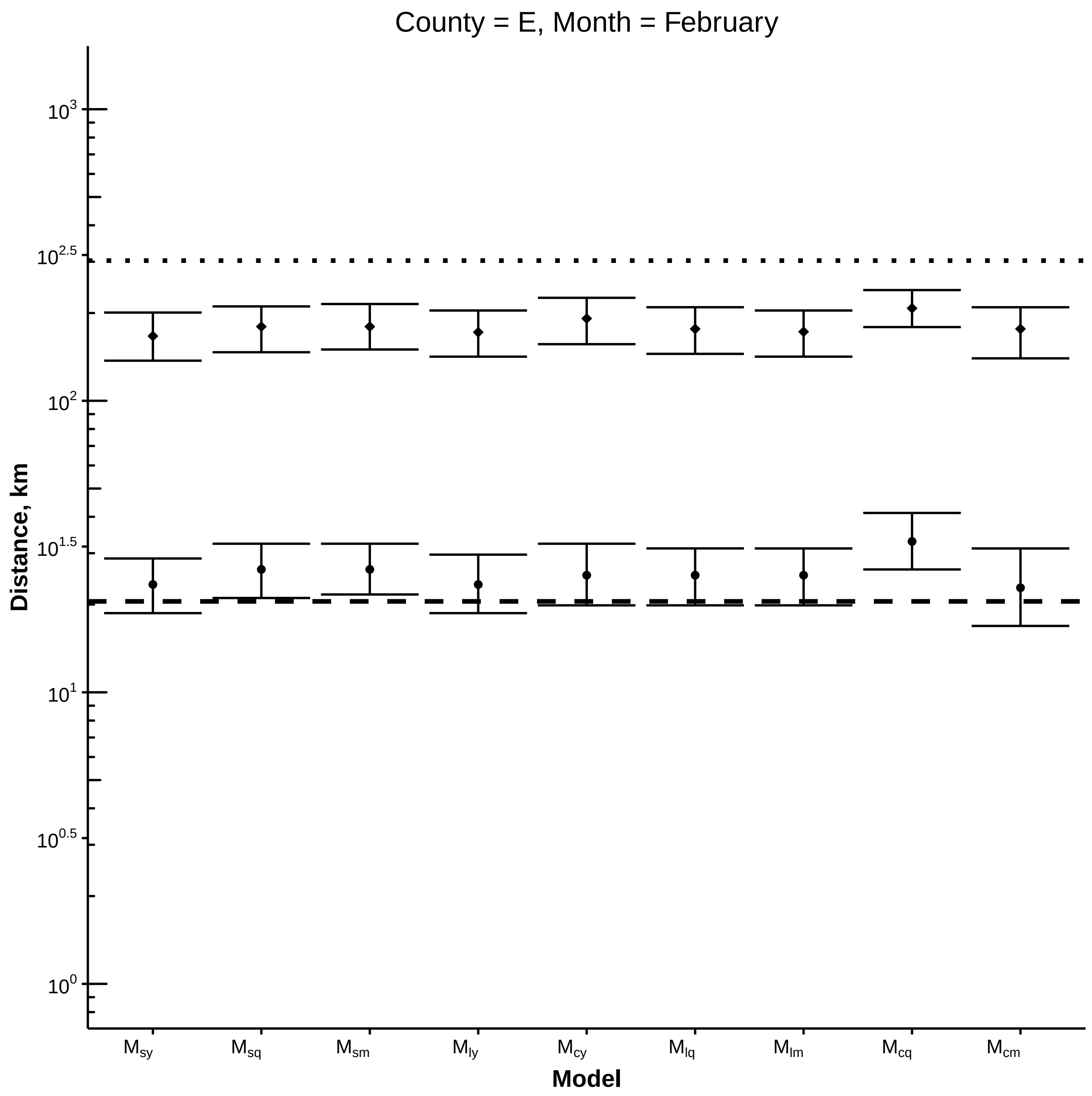

County = E, Month = February
Distance, km
Model
10^3
$10^{2.5}$
10^2
$10^{1.5}$
10^1
$10^{0.5}$
10^0
M_{sy}
M_{sq}
M_{sm}
M_{ly}
M_{cy}
M_{lq}
M_{lm}
M_{cq}
M_{cm}

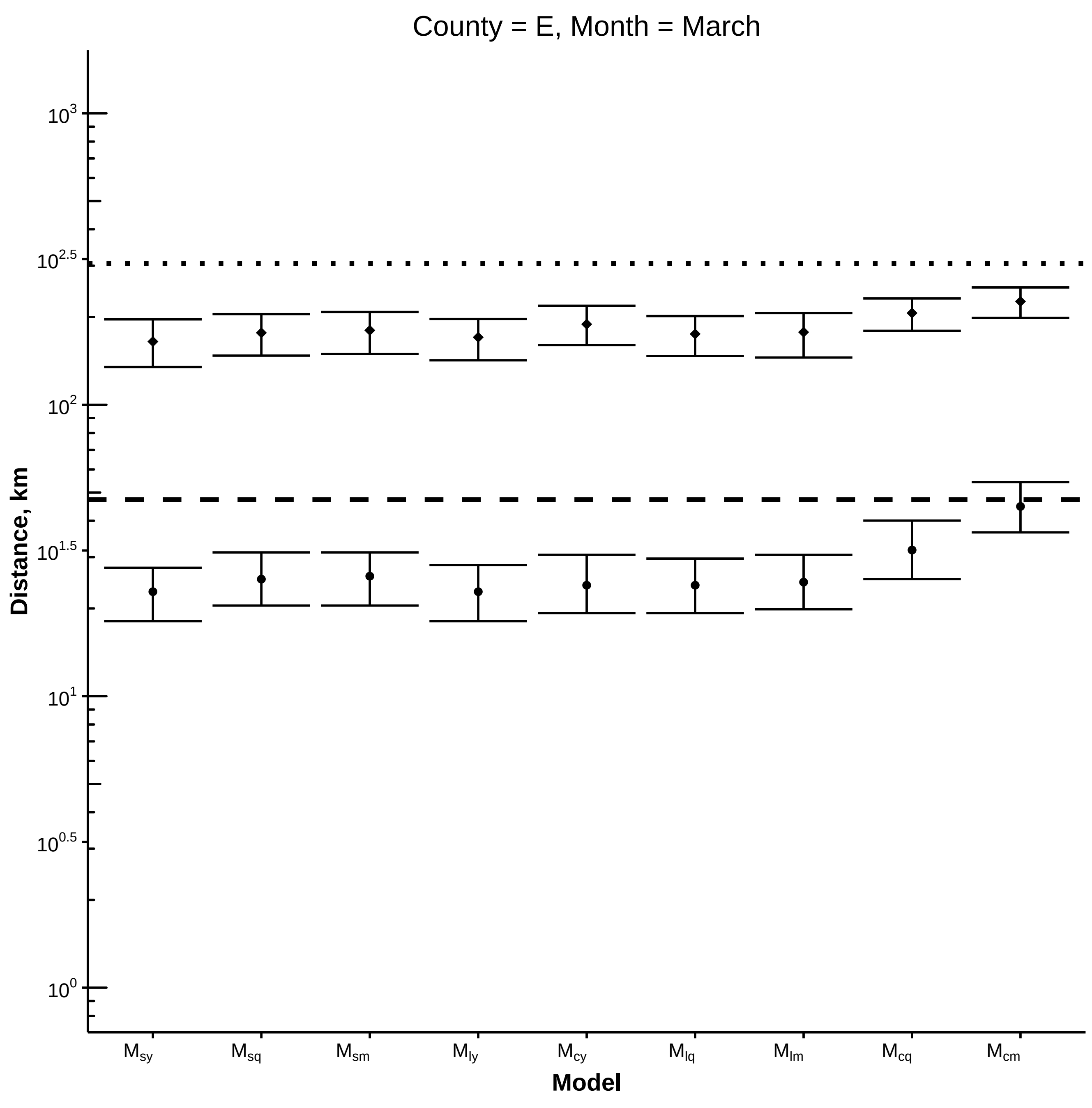

County = E, Month = March
Distance, km
10^3
$10^{2.5}$
10^2
$10^{1.5}$
10^1
$10^{0.5}$
10^0
M_{sy}
M_{sq}
M_{sm}
M_{ly}
M_{cy}
M_{lq}
M_{lm}
M_{cq}
M_{cm}
Model

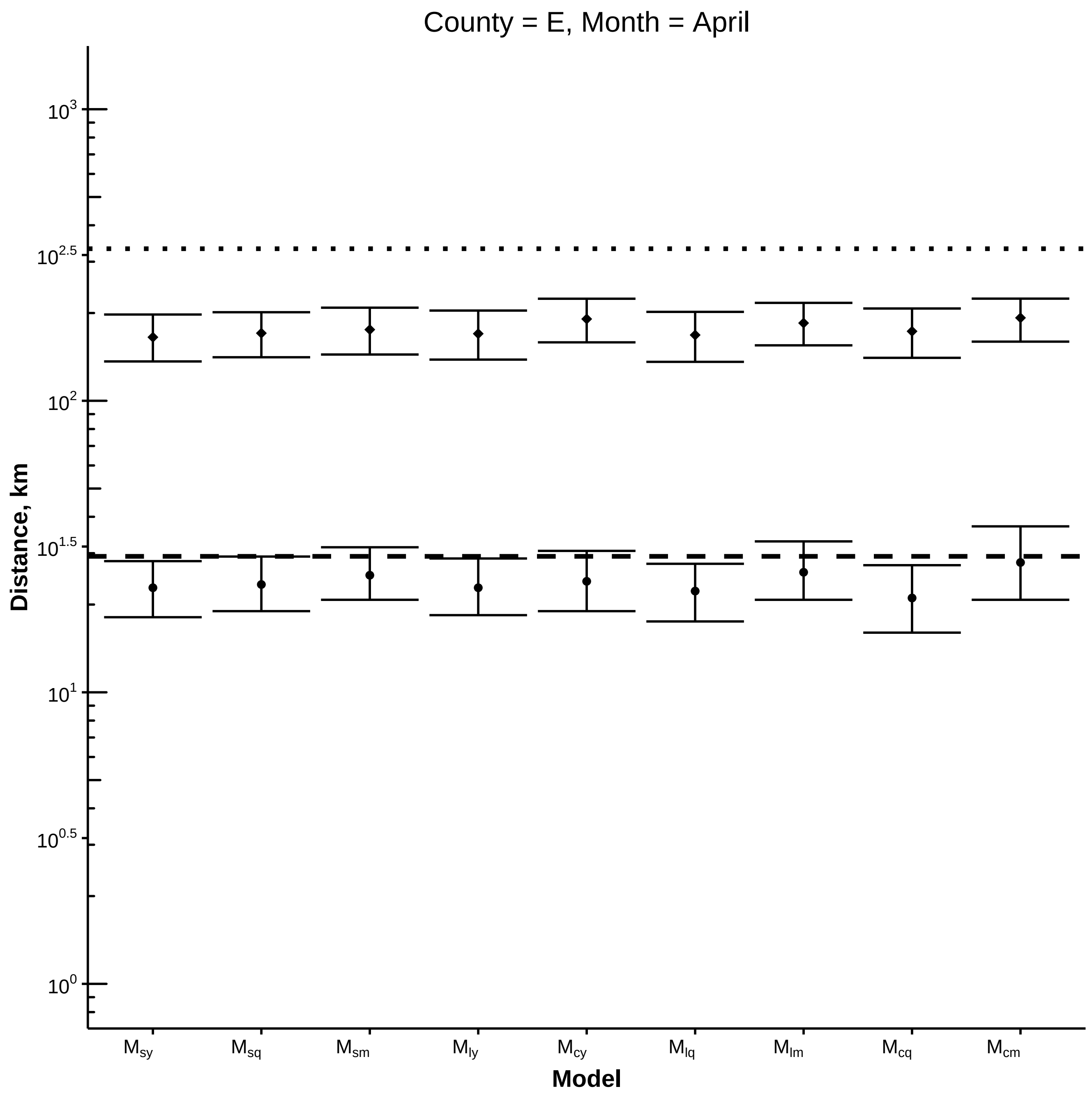
County = E, Month = April
Distance, km
10^3
$10^{2.5}$
10^2
$10^{1.5}$
10^1
$10^{0.5}$
10^0
M_{sy}
M_{sq}
M_{sm}
M_{ly}
M_{cy}
M_{lq}
M_{lm}
M_{cq}
M_{cm}
Model

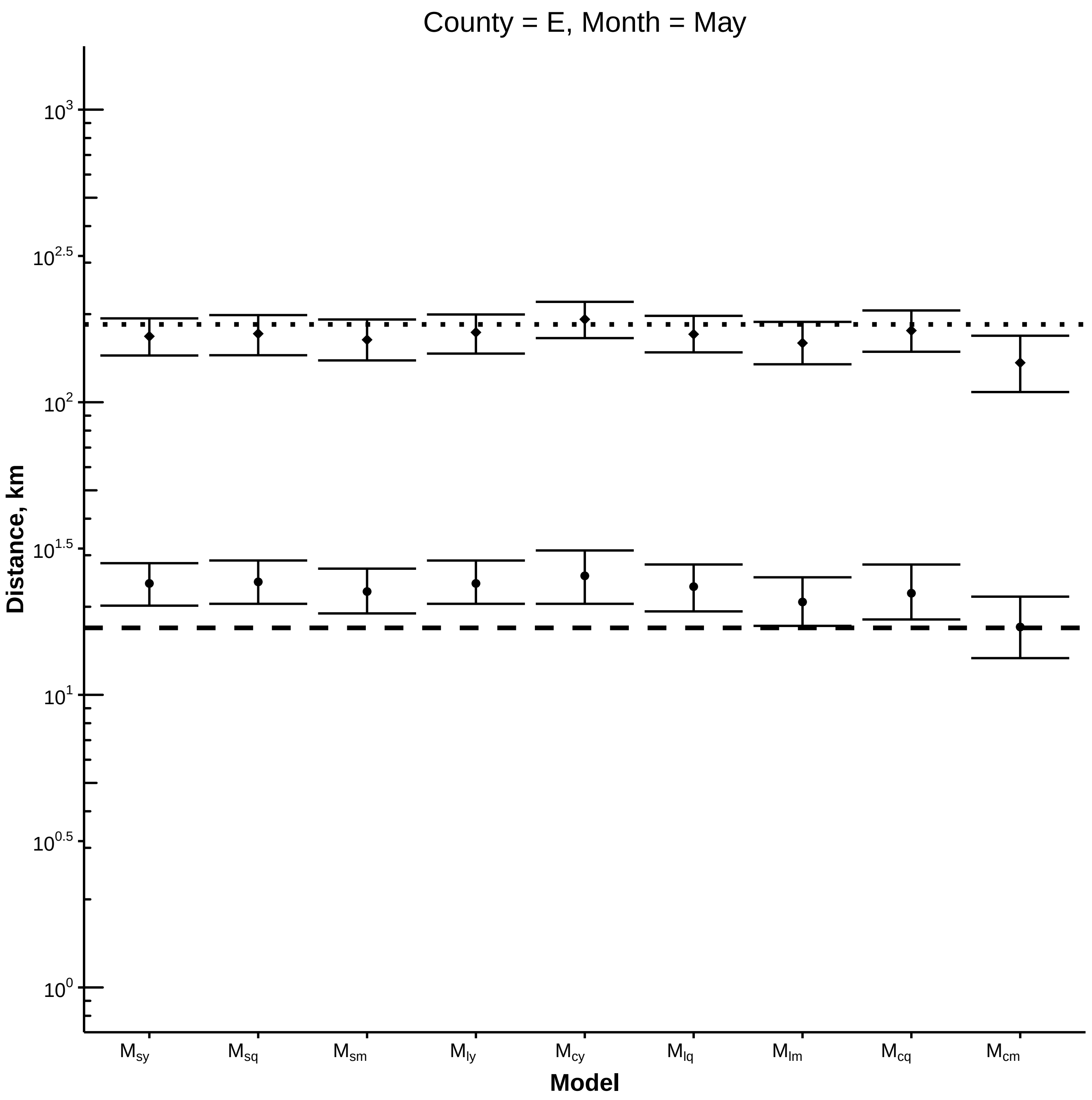
County = E, Month = May
Distance, km
10^3
$10^{2.5}$
10^2
$10^{1.5}$
10^1
$10^{0.5}$
10^0
M_{sy}
M_{sq}
M_{sm}
M_{ly}
M_{cy}
M_{lq}
M_{lm}
M_{cq}
M_{cm}
Model

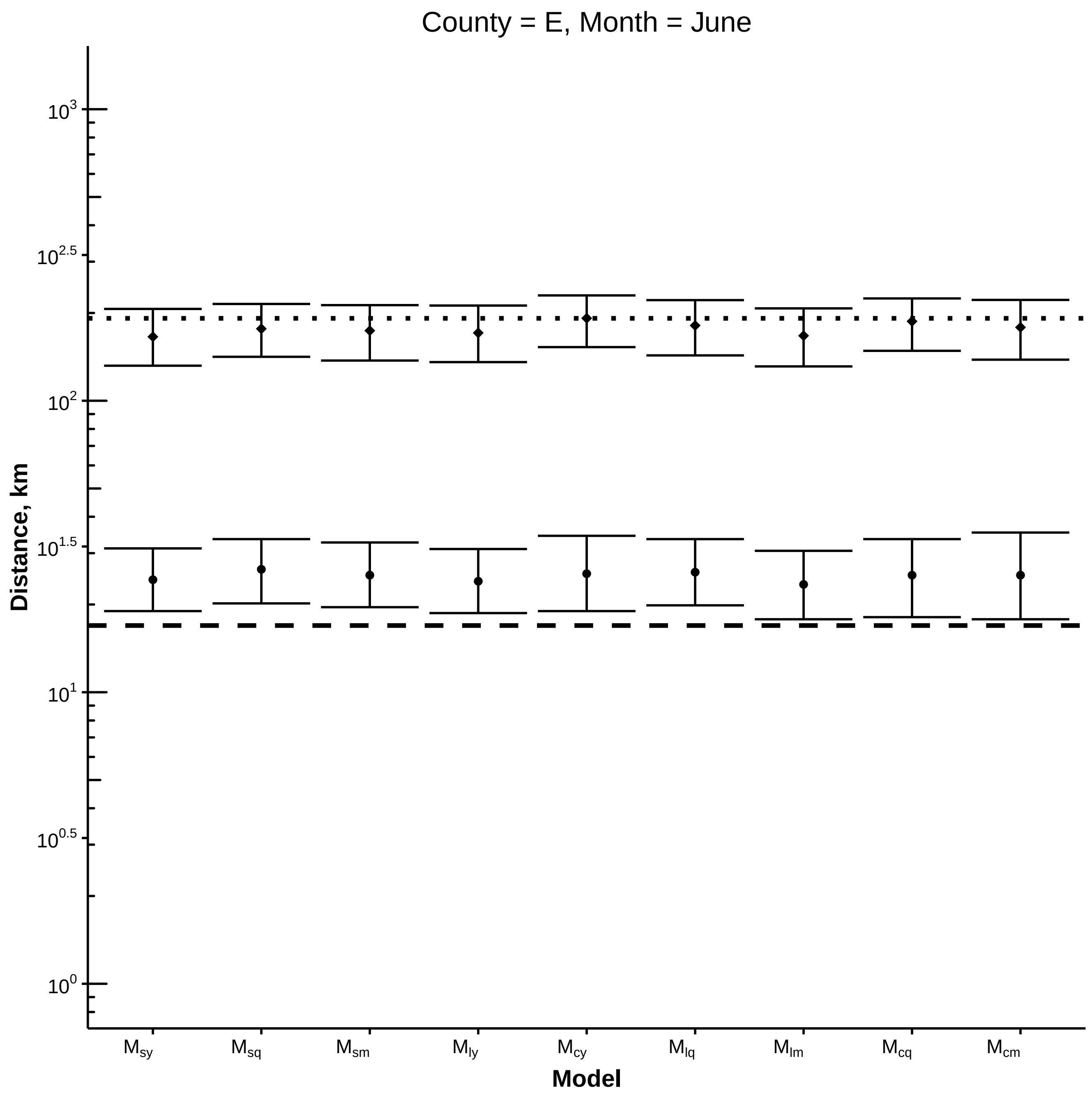

County = E, Month = June
Distance, km
10^3
$10^{2.5}$
10^2
$10^{1.5}$
10^1
$10^{0.5}$
10^0
M_{sy}
M_{sq}
M_{sm}
M_{ly}
M_{cy}
M_{lq}
M_{lm}
M_{cq}
M_{cm}
Model

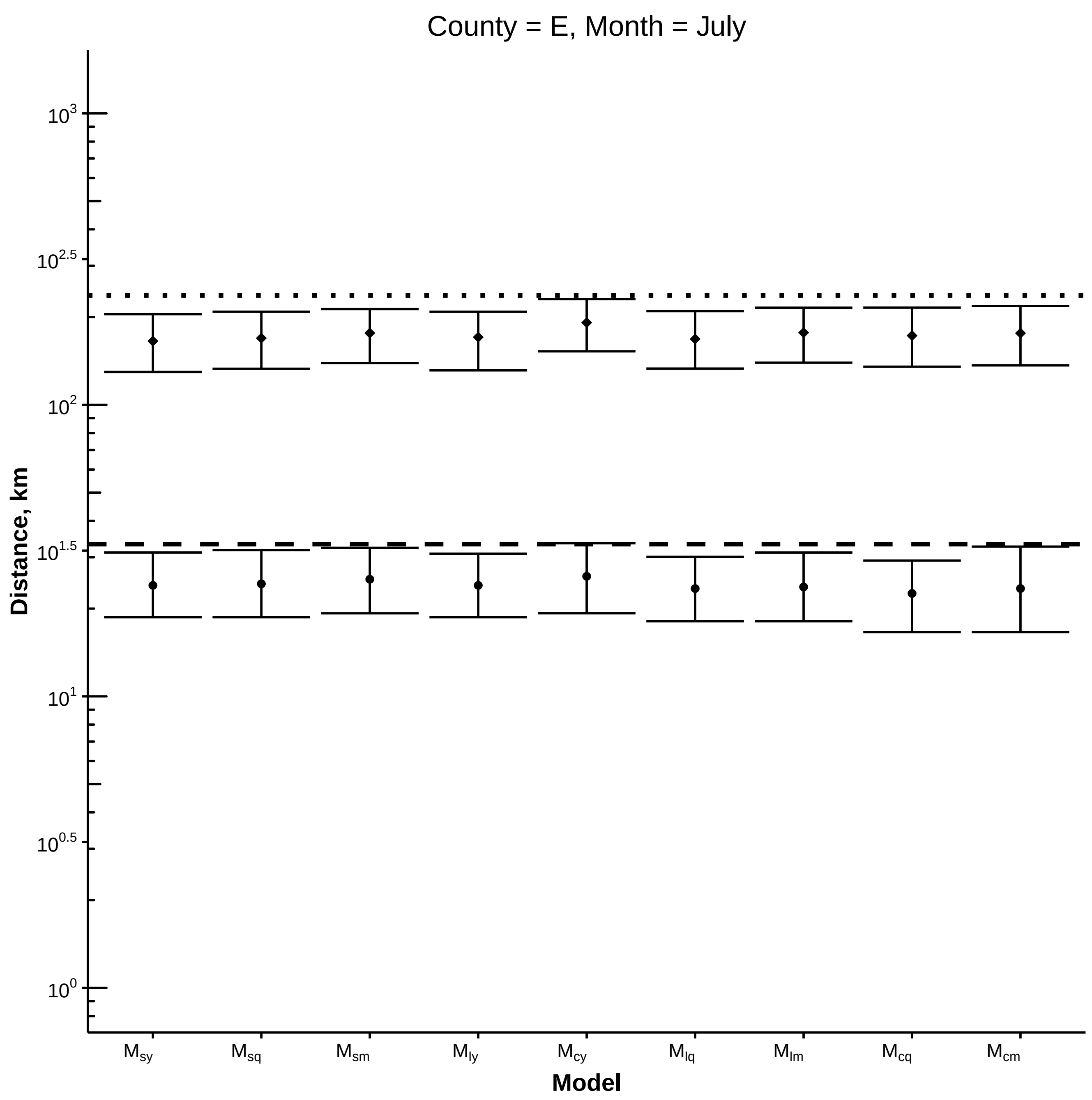
County = E, Month = July
Distance, km
10^3
$10^{2.5}$
10^2
$10^{1.5}$
10^1
$10^{0.5}$
10^0
M_{sy}
M_{sq}
M_{sm}
M_{ly}
M_{cy}
M_{lq}
M_{lm}
M_{cq}
M_{cm}
Model

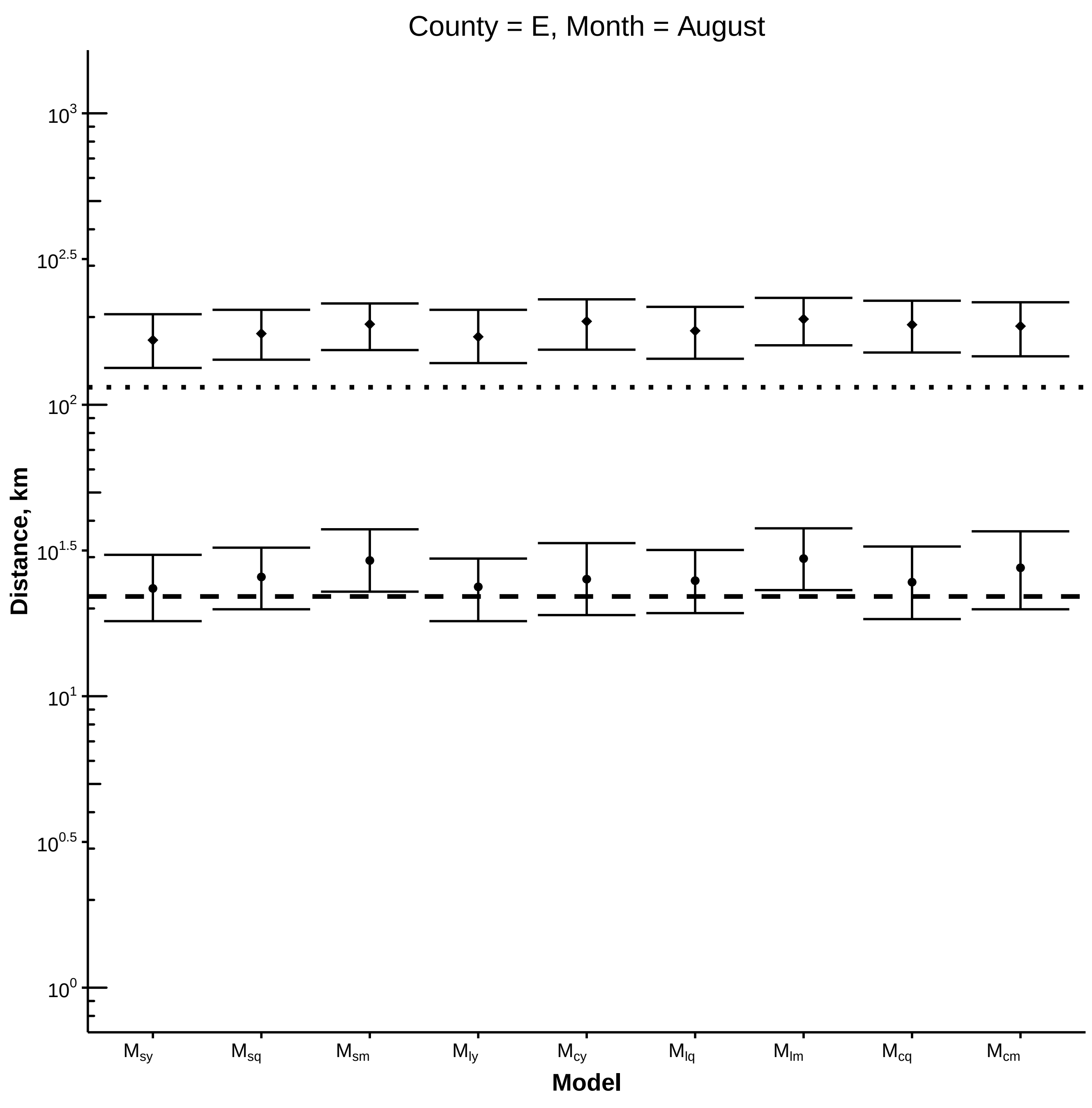

County = E, Month = August
Distance, km
10^3
$10^{2.5}$
10^2
$10^{1.5}$
10^1
$10^{0.5}$
10^0
M_{sy}
M_{sq}
M_{sm}
M_{ly}
M_{cy}
M_{lq}
M_{lm}
M_{cq}
M_{cm}
Model

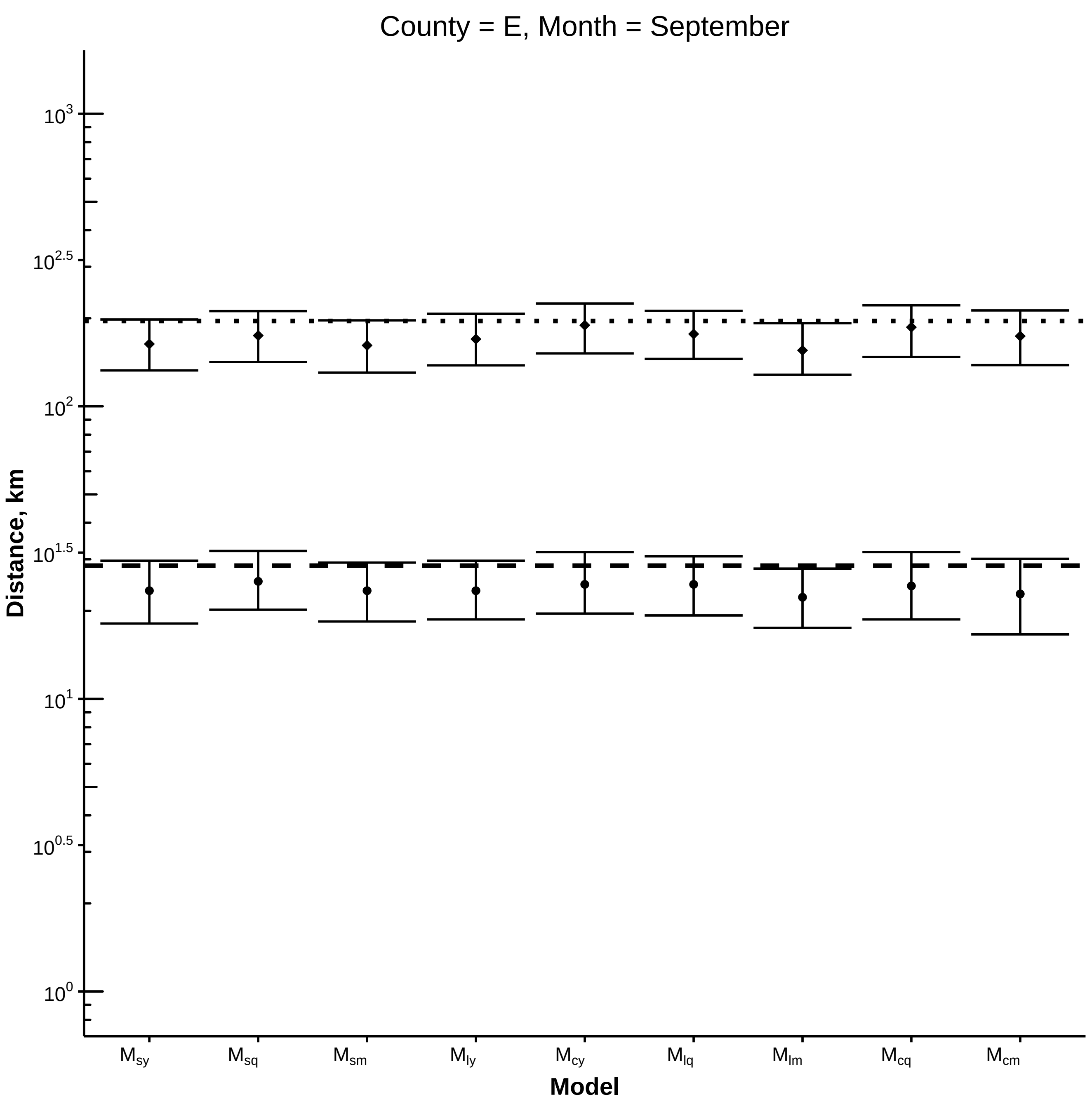

County = E, Month = September
Distance, km
10^3
$10^{2.5}$
10^2
$10^{1.5}$
10^1
$10^{0.5}$
10^0
M_{sy}
M_{sq}
M_{sm}
M_{ly}
M_{cy}
M_{lq}
M_{lm}
M_{cq}
M_{cm}
Model

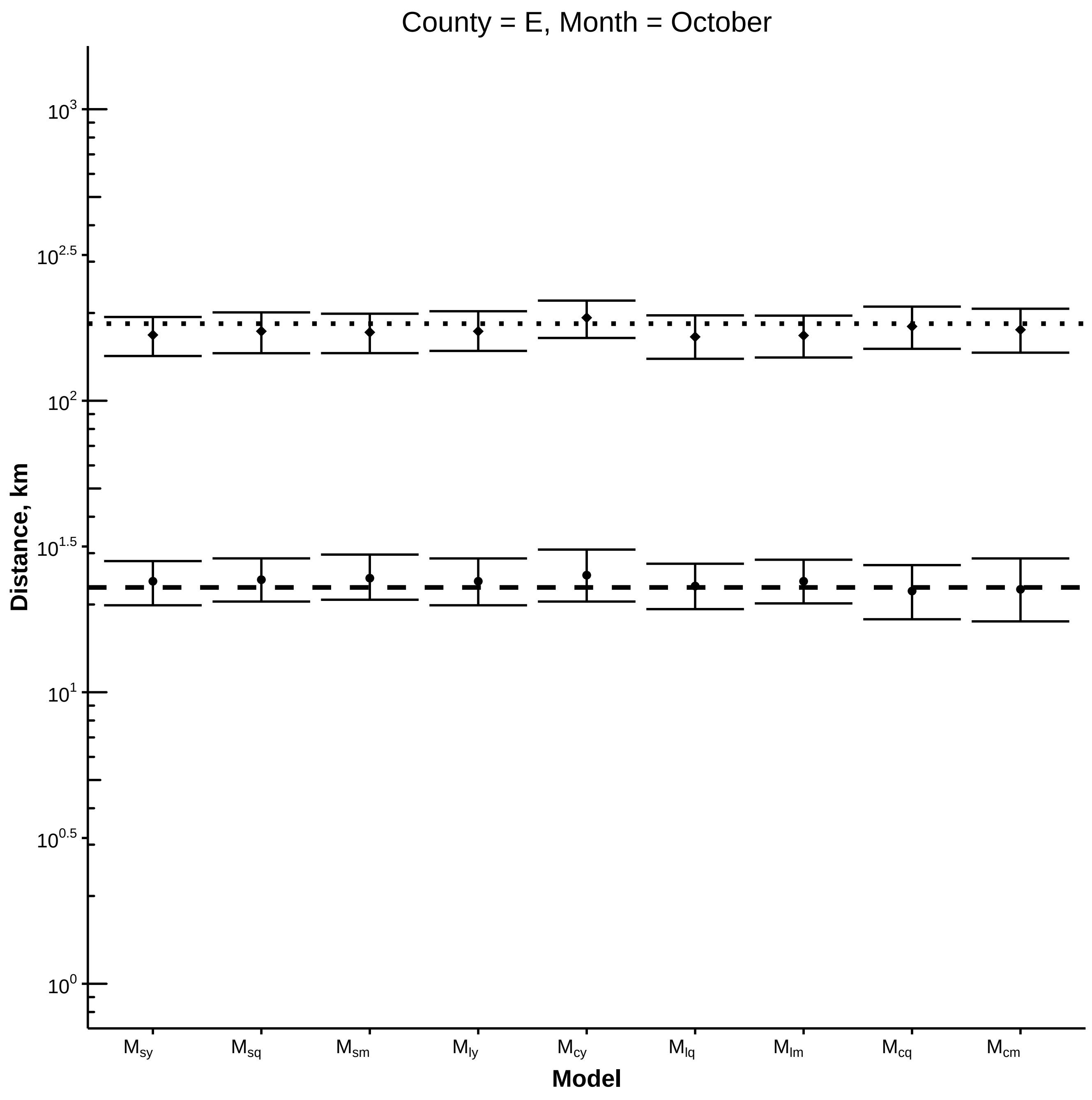
County = E, Month = October
Distance, km
10^{3}
$10^{2.5}$
10^{2}
$10^{1.5}$
10^{1}
$10^{0.5}$
10^{0}
M_{sy}
M_{sq}
M_{sm}
M_{ly}
M_{cy}
M_{lq}
M_{lm}
M_{cq}
M_{cm}
Model

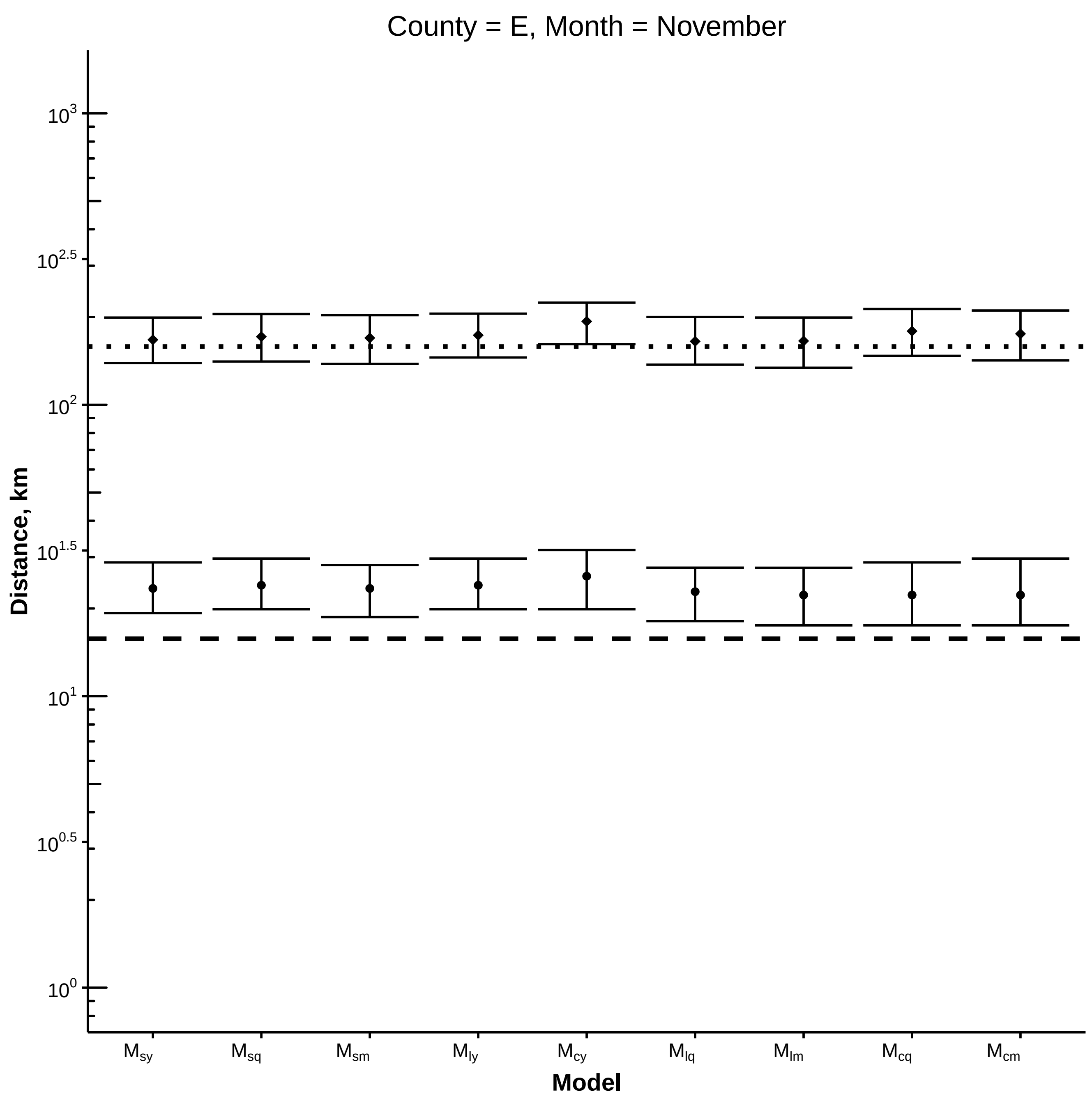

County = E, Month = November
Distance, km
10^{3}
$10^{2.5}$
10^{2}
$10^{1.5}$
10^{1}
$10^{0.5}$
10^{0}
M_{sy}
M_{sq}
M_{sm}
M_{ly}
M_{cy}
M_{lq}
M_{lm}
M_{cq}
M_{cm}
Model

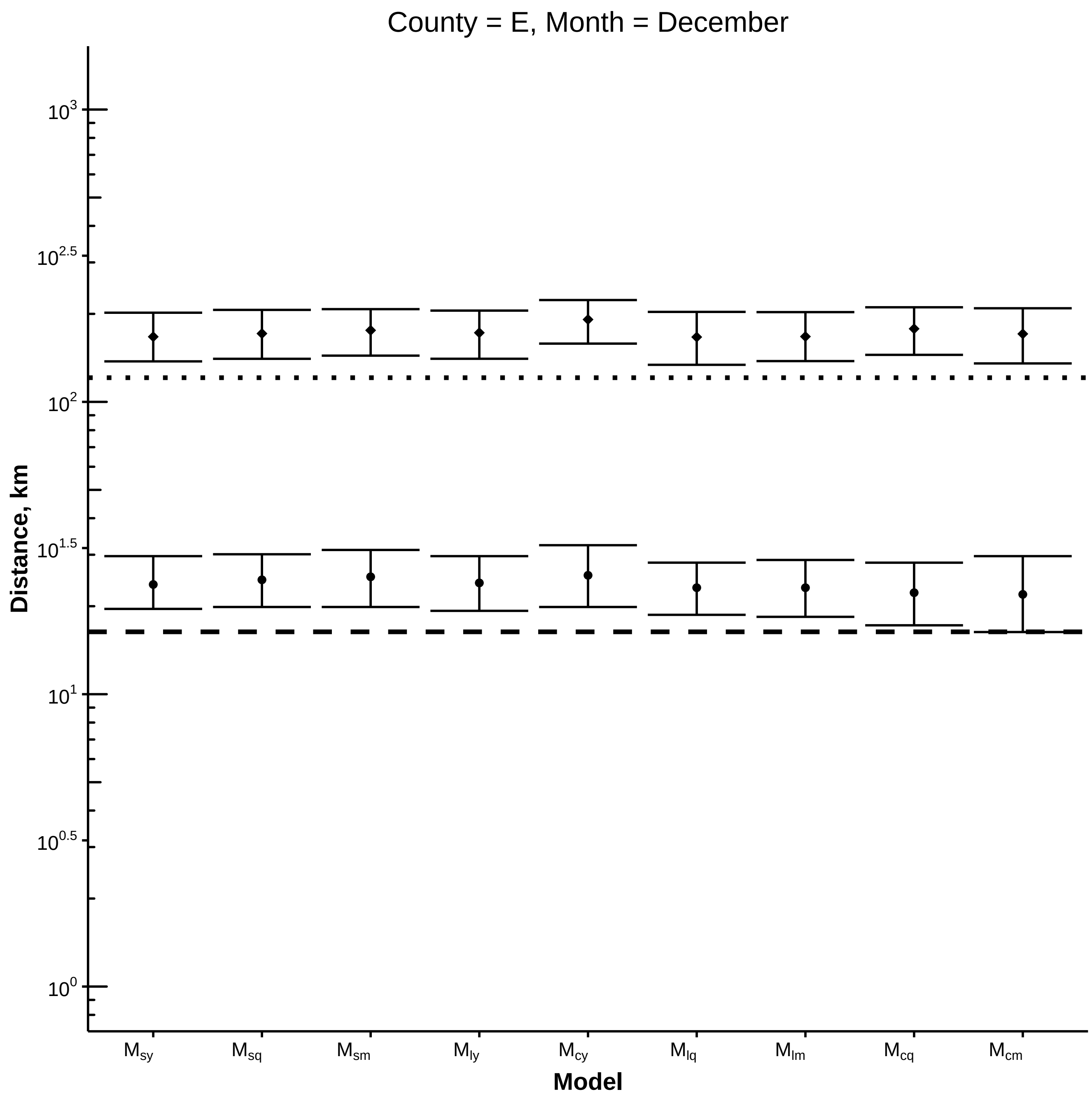
County = E, Month = December
Distance, km
10^3
$10^{2.5}$
10^2
$10^{1.5}$
10^1
$10^{0.5}$
10^0
M_{sy}
M_{sq}
M_{sm}
M_{ly}
M_{cy}
M_{lq}
M_{lm}
M_{cq}
M_{cm}
Model

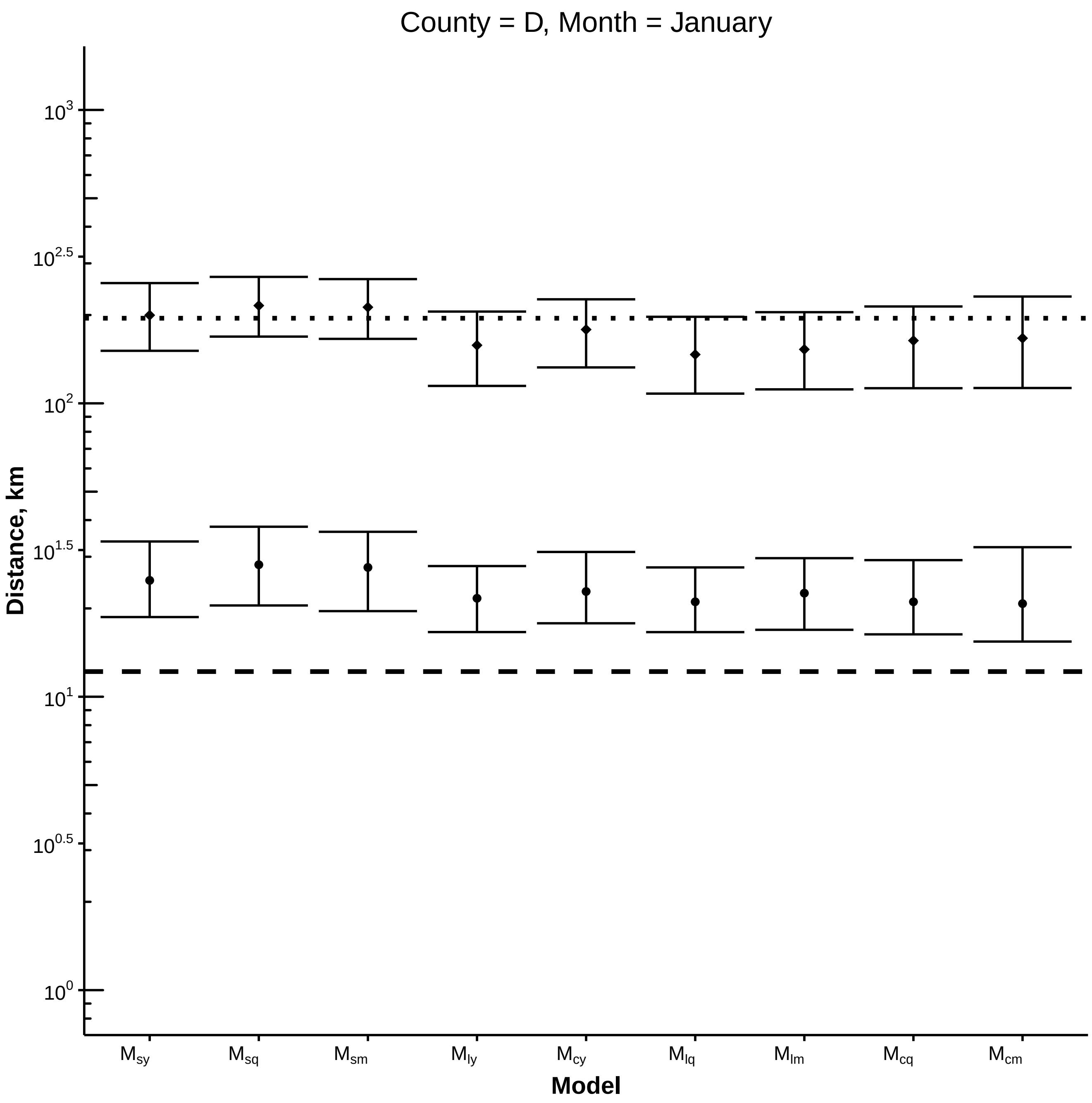
County = D, Month = January
Distance, km
10^3
$10^{2.5}$
10^2
$10^{1.5}$
10^1
$10^{0.5}$
10^0
M_{sy}
M_{sq}
M_{sm}
M_{ly}
M_{cy}
M_{lq}
M_{lm}
M_{cq}
M_{cm}
Model

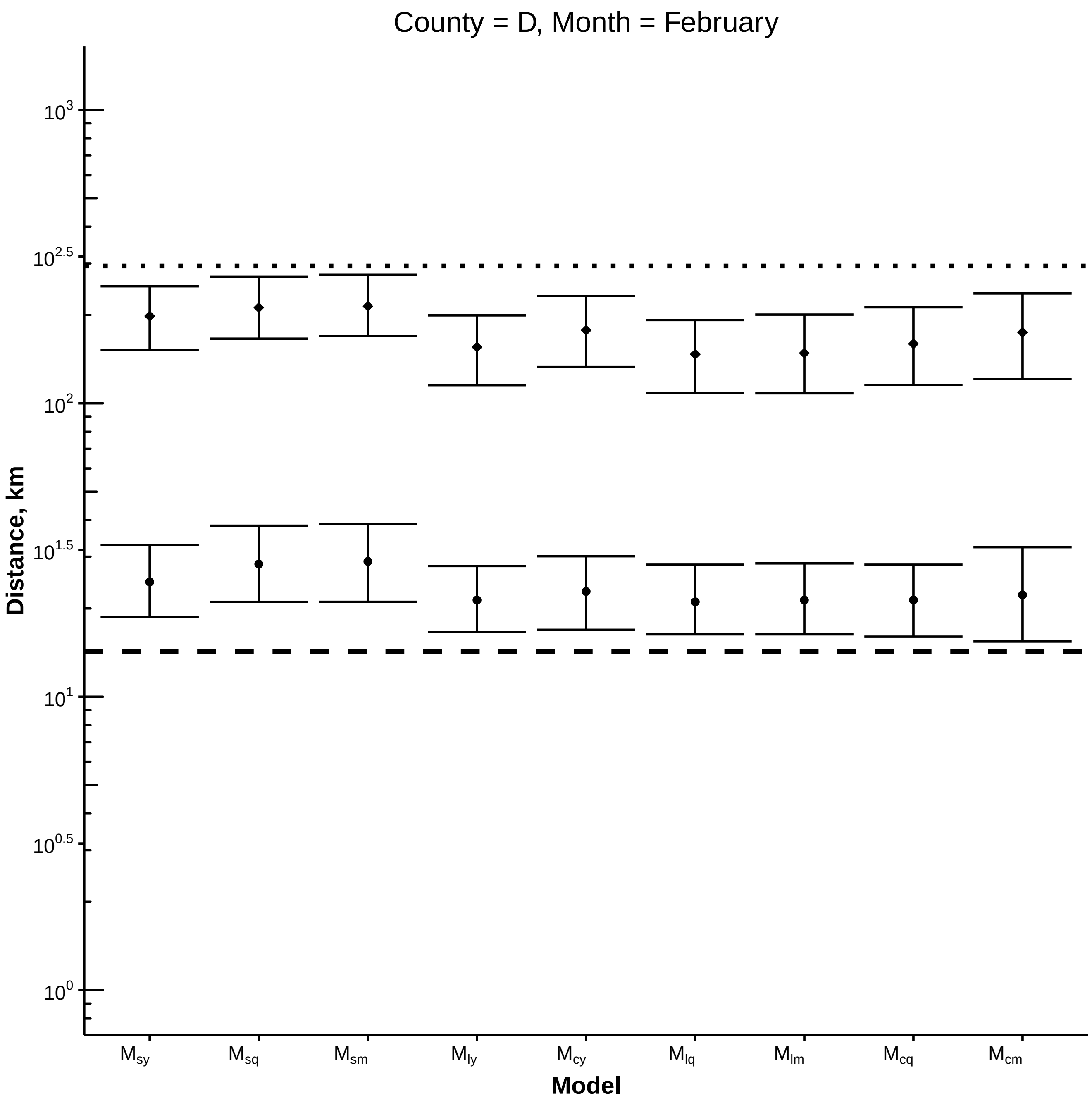
County = D, Month = February
Distance, km
10^3
$10^{2.5}$
10^2
$10^{1.5}$
10^1
$10^{0.5}$
10^0
M_{sy}
M_{sq}
M_{sm}
M_{ly}
M_{cy}
M_{lq}
M_{lm}
M_{cq}
M_{cm}
Model

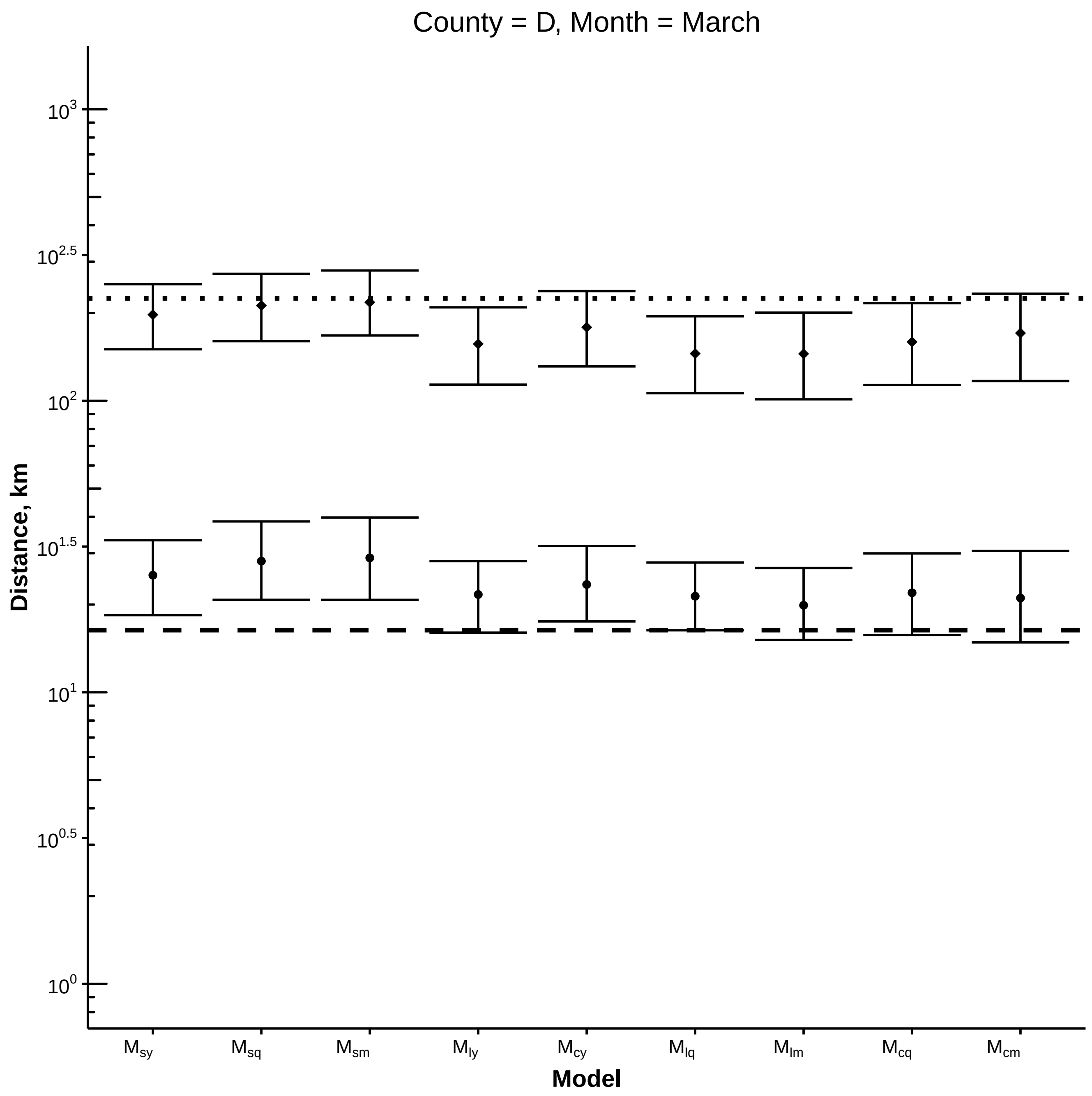
County = D, Month = March
Distance, km
10^3
$10^{2.5}$
10^2
$10^{1.5}$
10^1
$10^{0.5}$
10^0
M_{sy}
M_{sq}
M_{sm}
M_{ly}
M_{cy}
M_{lq}
M_{lm}
M_{cq}
M_{cm}
Model

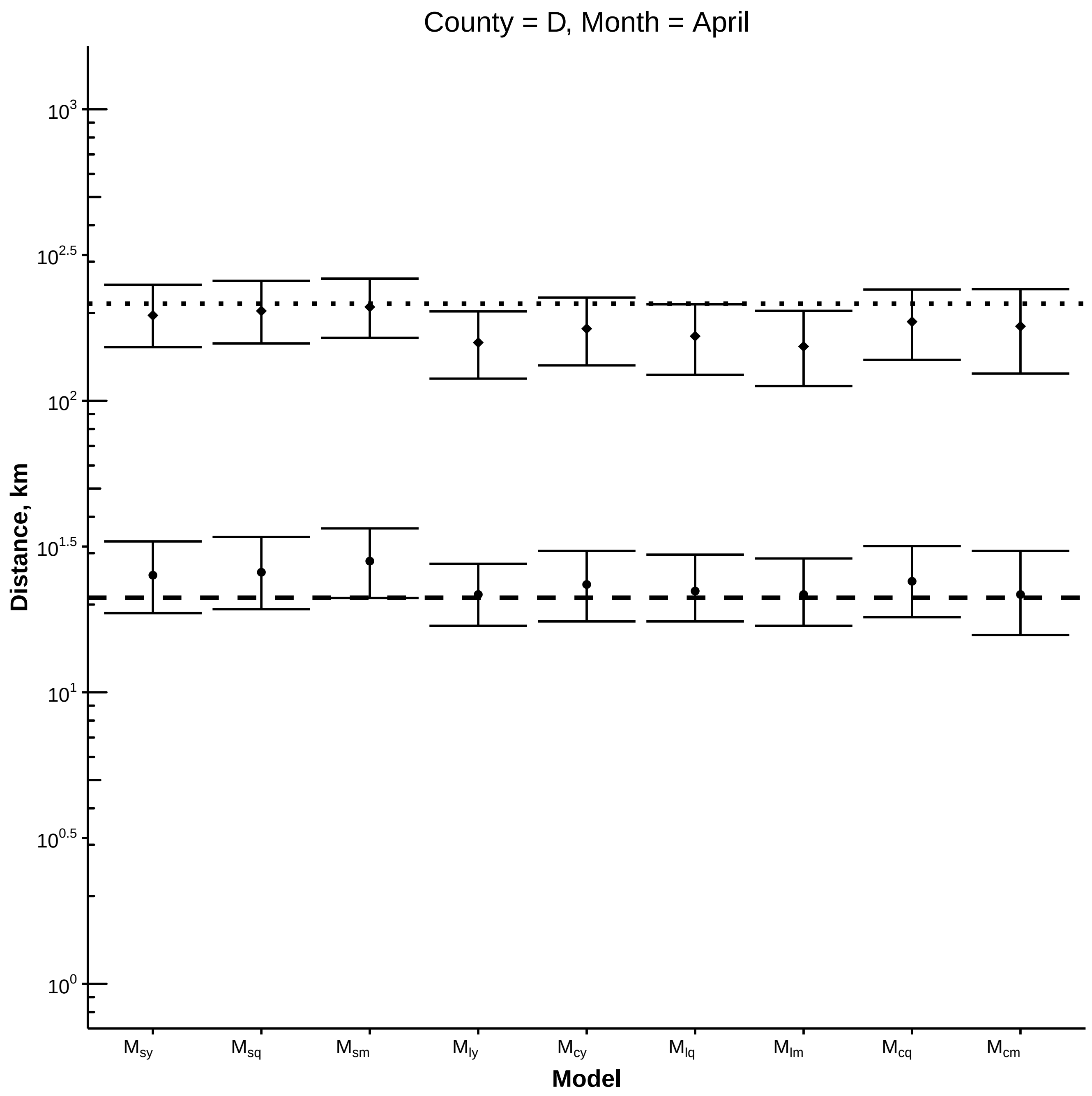

County = D, Month = April
Distance, km
10^3
$10^{2.5}$
10^2
$10^{1.5}$
10^1
$10^{0.5}$
10^0
M_{sy}
M_{sq}
M_{sm}
M_{ly}
M_{cy}
M_{lq}
M_{lm}
M_{cq}
M_{cm}
Model

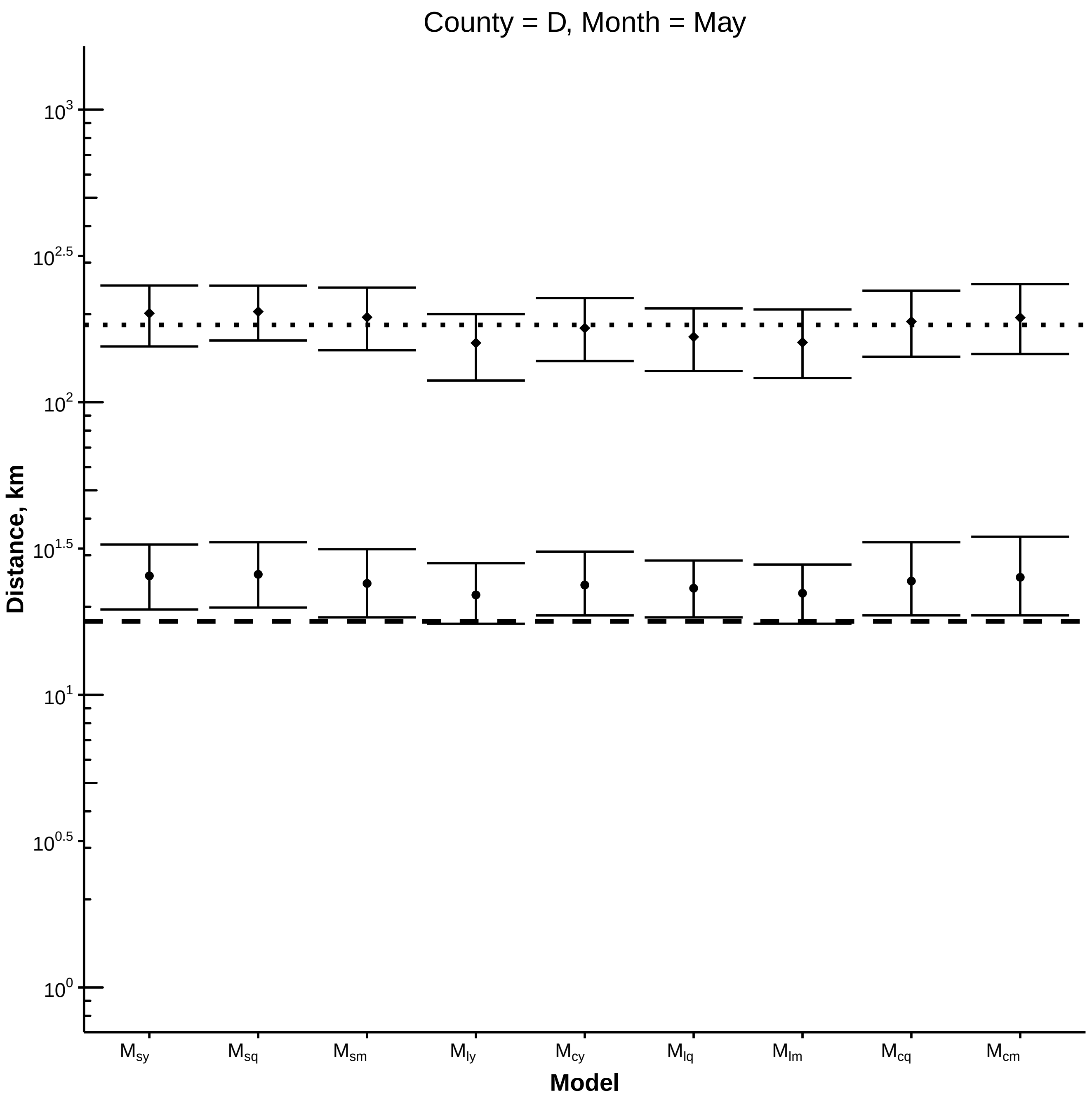
County = D, Month = May
Distance, km
10^3
$10^{2.5}$
10^2
$10^{1.5}$
10^1
$10^{0.5}$
10^0
M_{sy}
M_{sq}
M_{sm}
M_{ly}
M_{cy}
M_{lq}
M_{lm}
M_{cq}
M_{cm}
Model

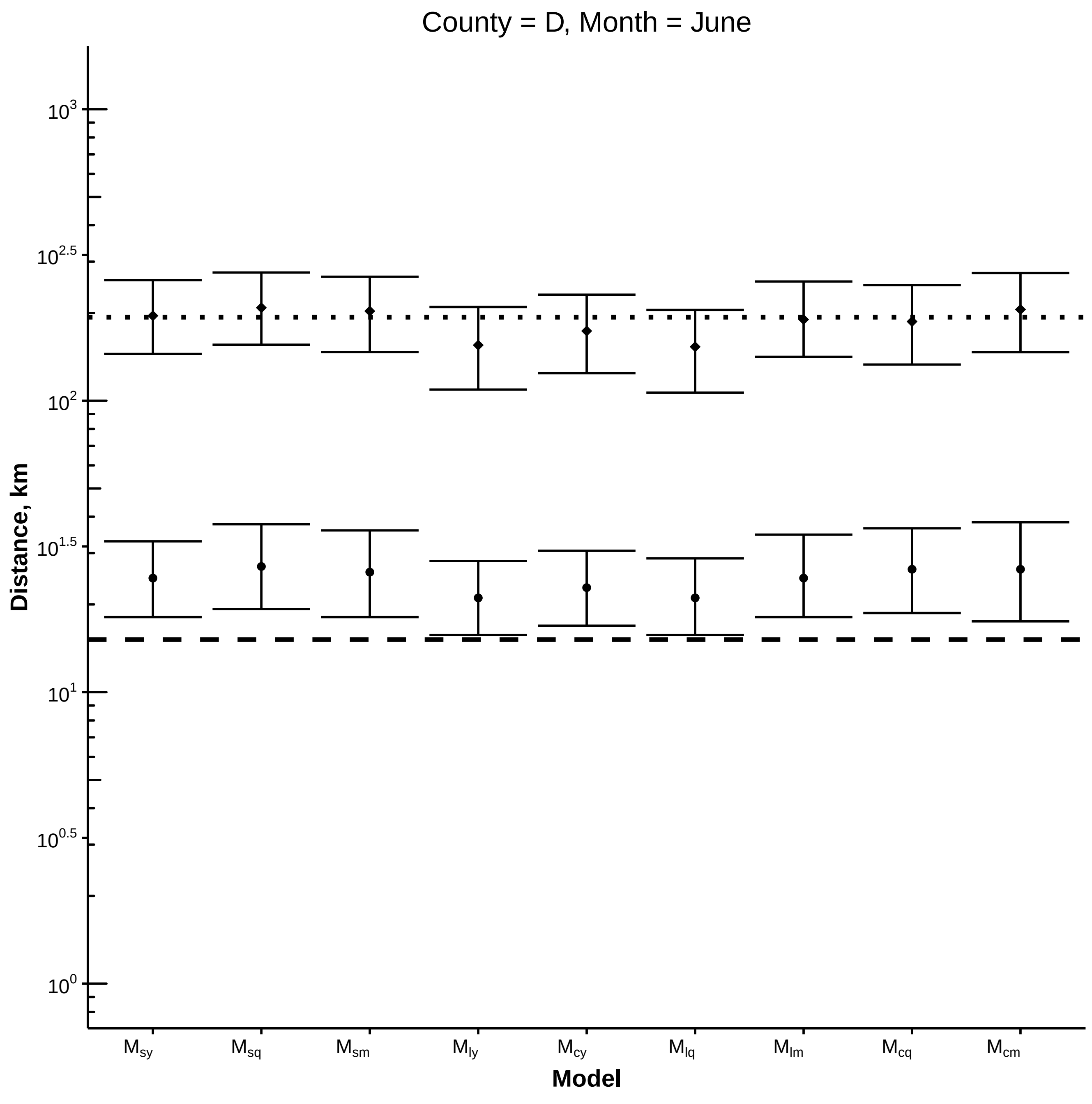

County = D, Month = June
Distance, km
10^3
$10^{2.5}$
10^2
$10^{1.5}$
10^1
$10^{0.5}$
10^0
M_{sy}
M_{sq}
M_{sm}
M_{ly}
M_{cy}
M_{lq}
M_{lm}
M_{cq}
M_{cm}
Model

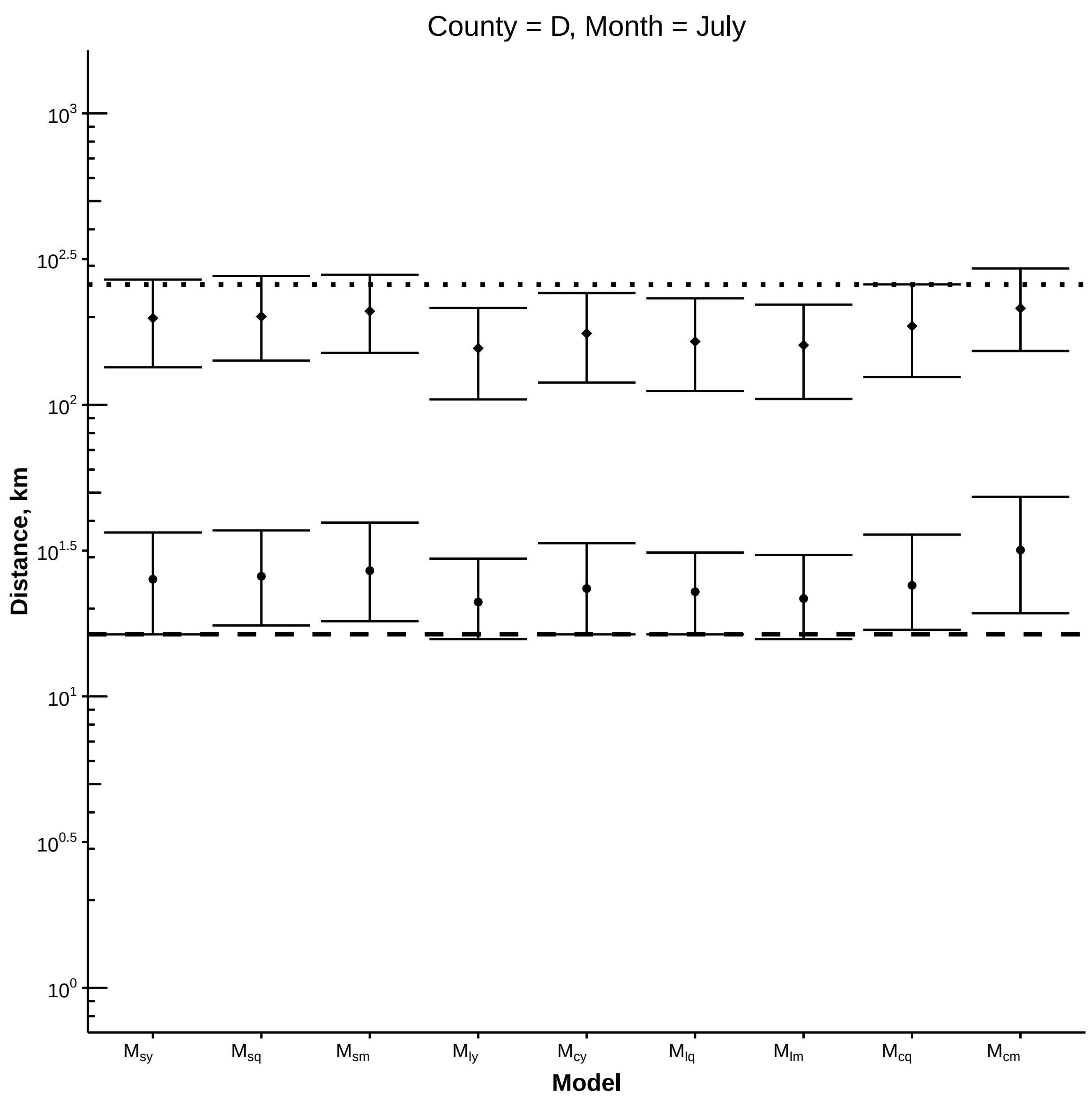
County = D, Month = July
Distance, km
10^3
$10^{2.5}$
10^2
$10^{1.5}$
10^1
$10^{0.5}$
10^0
M_{sy}
M_{sq}
M_{sm}
M_{ly}
M_{cy}
M_{lq}
M_{lm}
M_{cq}
M_{cm}
Model

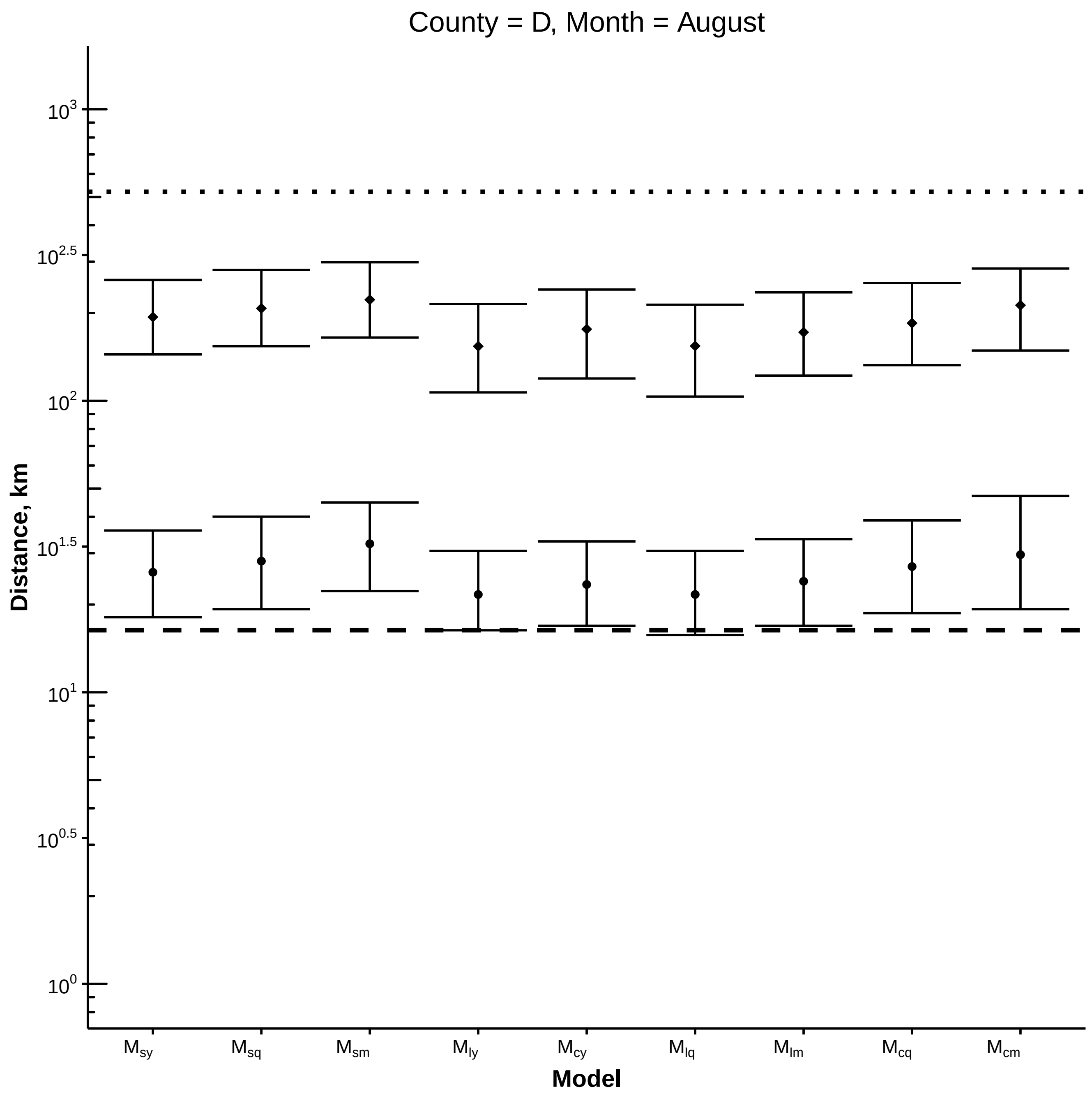

County = D, Month = August
Distance, km
10^3
$10^{2.5}$
10^2
$10^{1.5}$
10^1
$10^{0.5}$
10^0
M_{sy}
M_{sq}
M_{sm}
M_{ly}
M_{cy}
M_{lq}
M_{lm}
M_{cq}
M_{cm}
Model

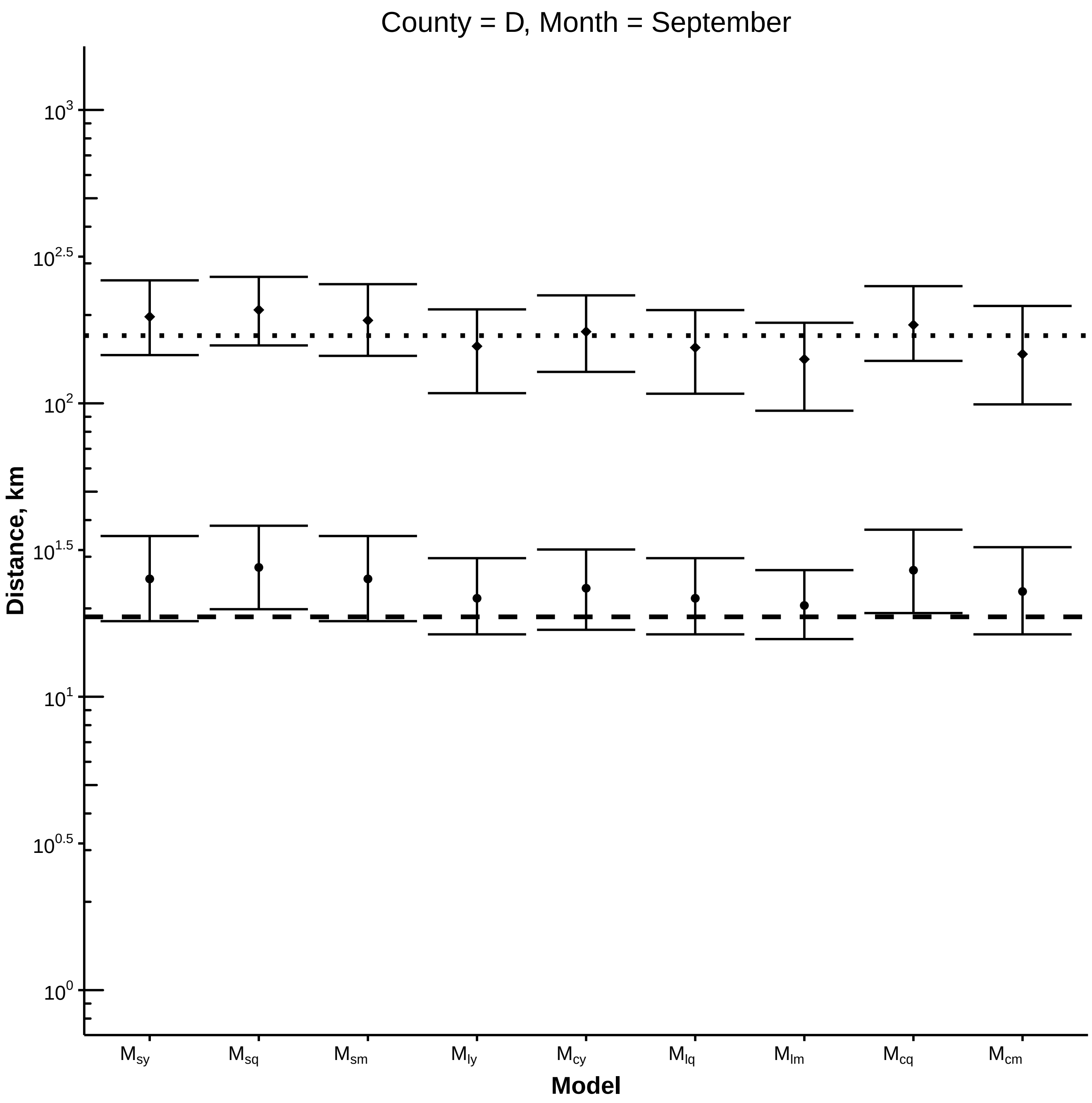

County = D, Month = September
Distance, km
10^3
$10^{2.5}$
10^2
$10^{1.5}$
10^1
$10^{0.5}$
10^0
M_{sy}
M_{sq}
M_{sm}
M_{ly}
M_{cy}
M_{lq}
M_{lm}
M_{cq}
M_{cm}
Model

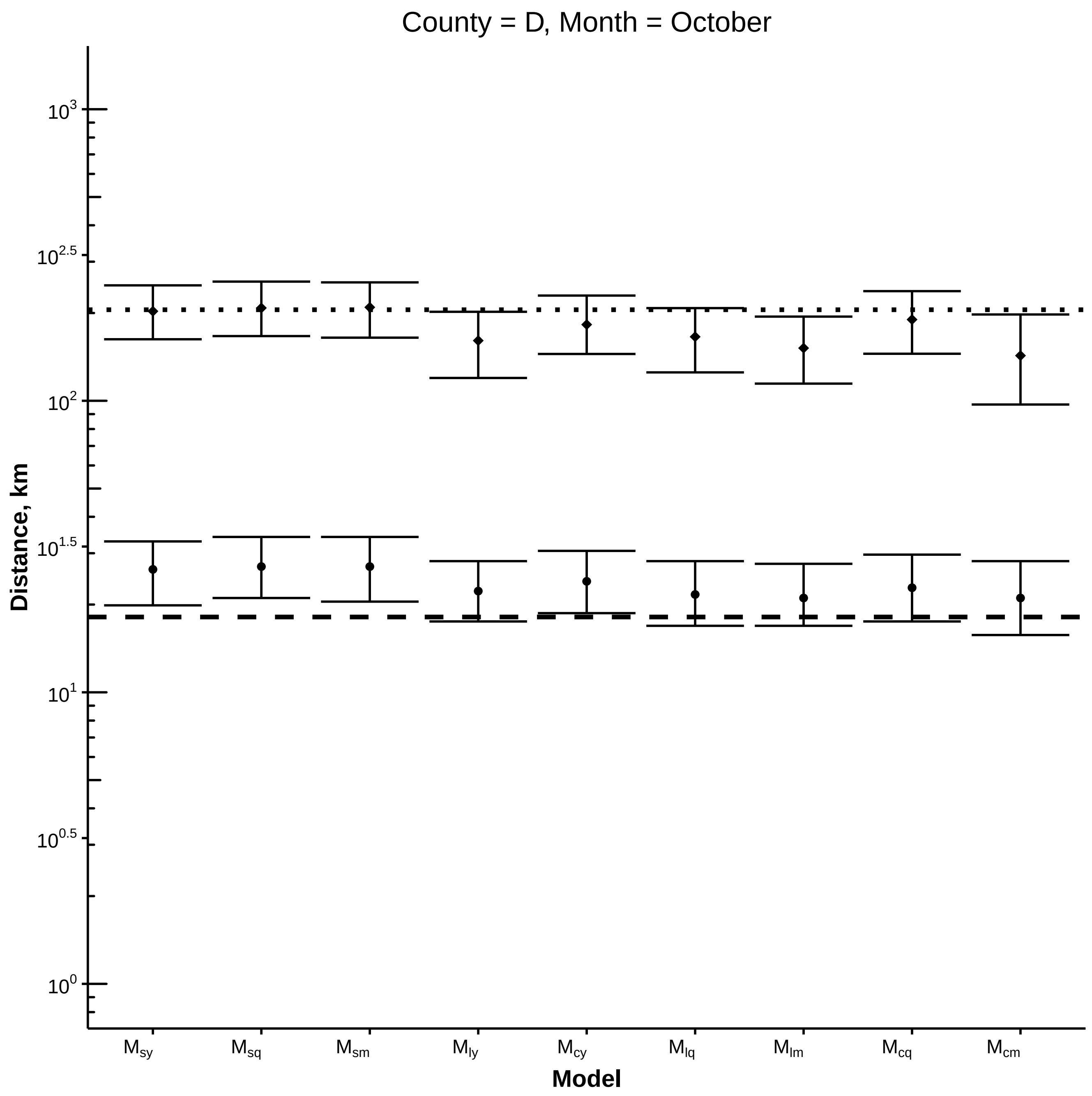
County = D, Month = October
Distance, km
10^3
$10^{2.5}$
10^2
$10^{1.5}$
10^1
$10^{0.5}$
10^0
M_{sy}
M_{sq}
M_{sm}
M_{ly}
M_{cy}
M_{lq}
M_{lm}
M_{cq}
M_{cm}
Model

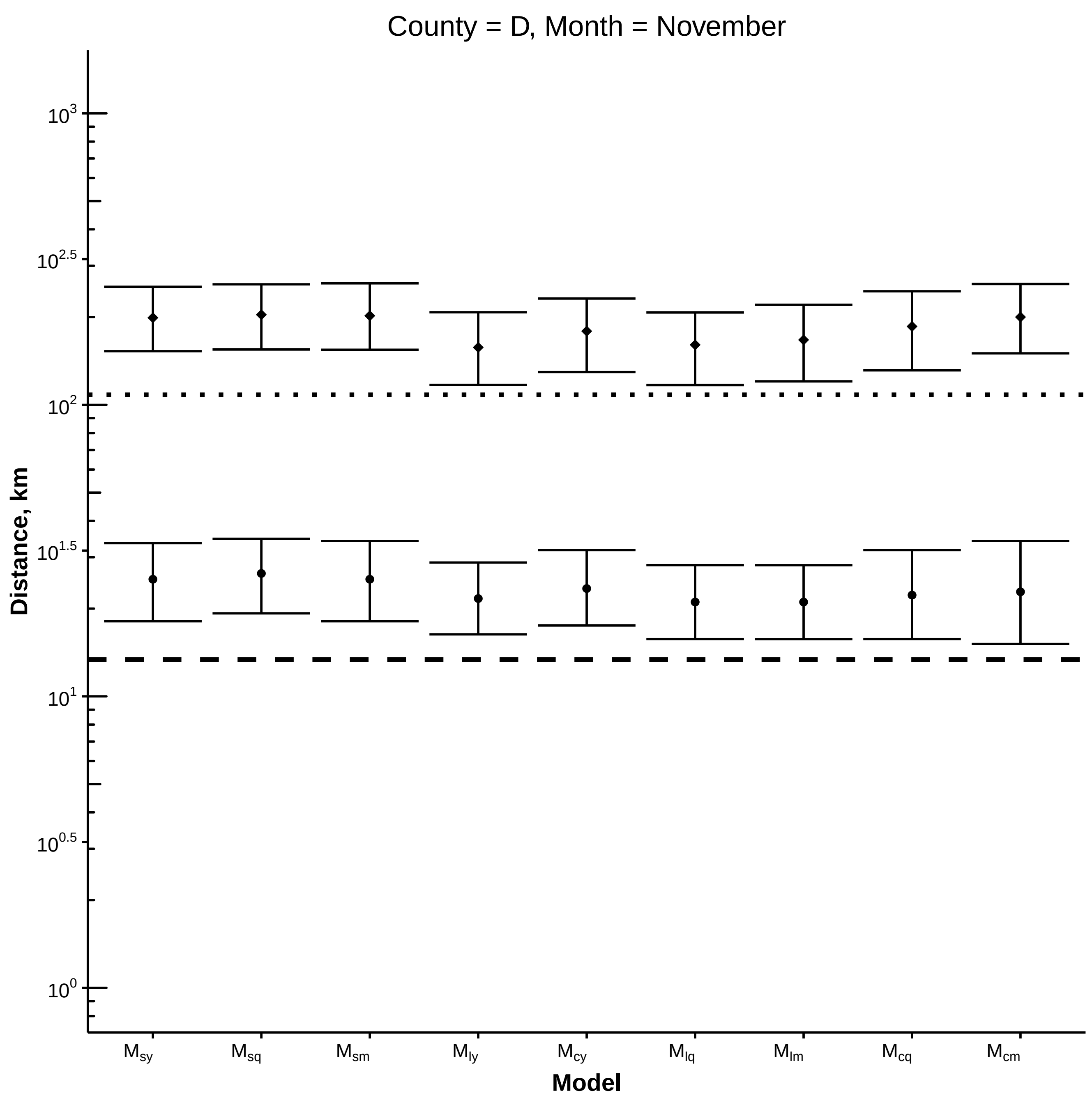
County = D, Month = November
Distance, km
10^3
$10^{2.5}$
10^2
$10^{1.5}$
10^1
$10^{0.5}$
10^0
M_{sy}
M_{sq}
M_{sm}
M_{ly}
M_{cy}
M_{lq}
M_{lm}
M_{cq}
M_{cm}
Model

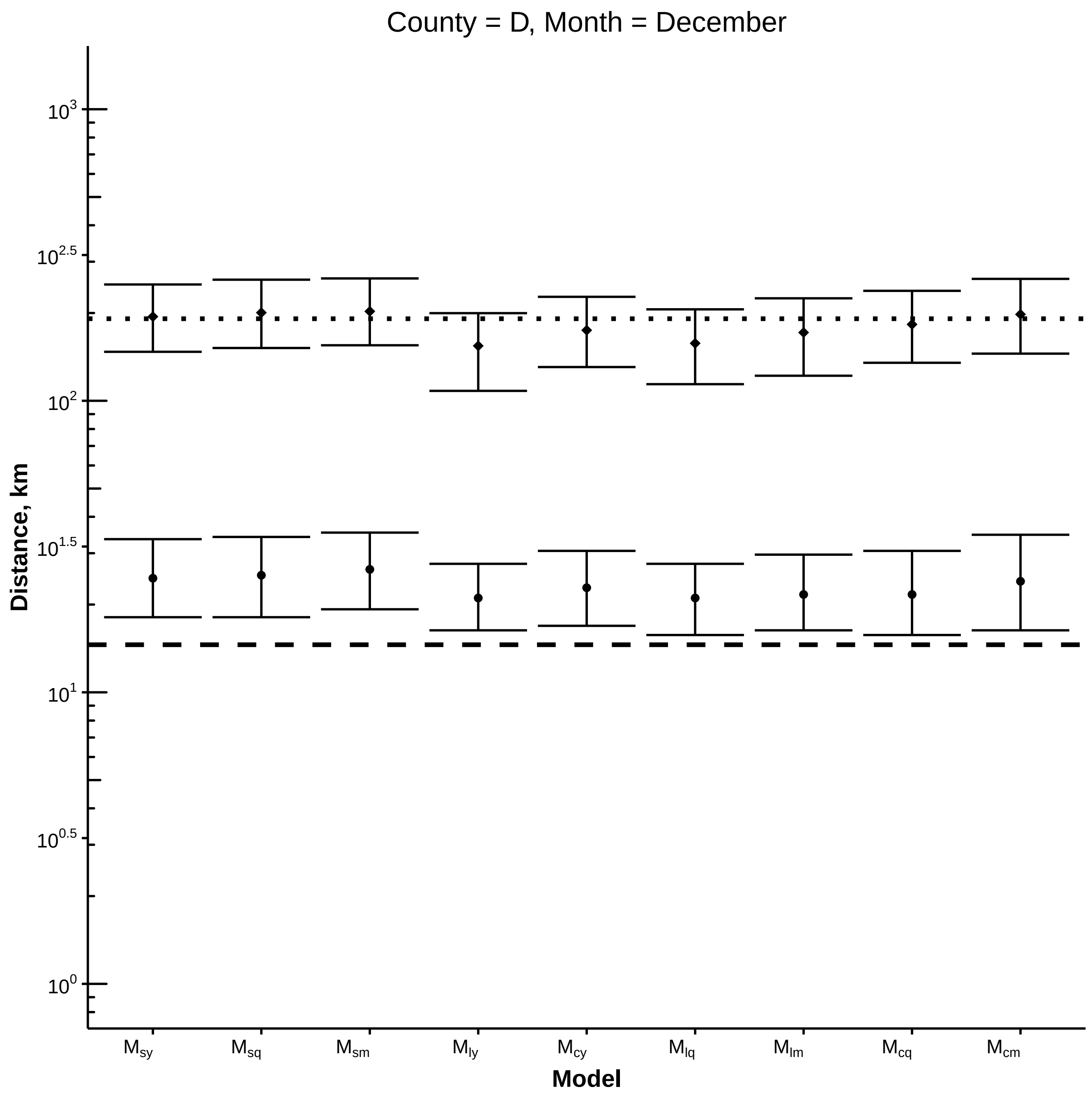
County = D, Month = December
Distance, km
10^3
$10^{2.5}$
10^2
$10^{1.5}$
10^1
$10^{0.5}$
10^0
M_{sy}
M_{sq}
M_{sm}
M_{ly}
M_{cy}
M_{lq}
M_{lm}
M_{cq}
M_{cm}
Model

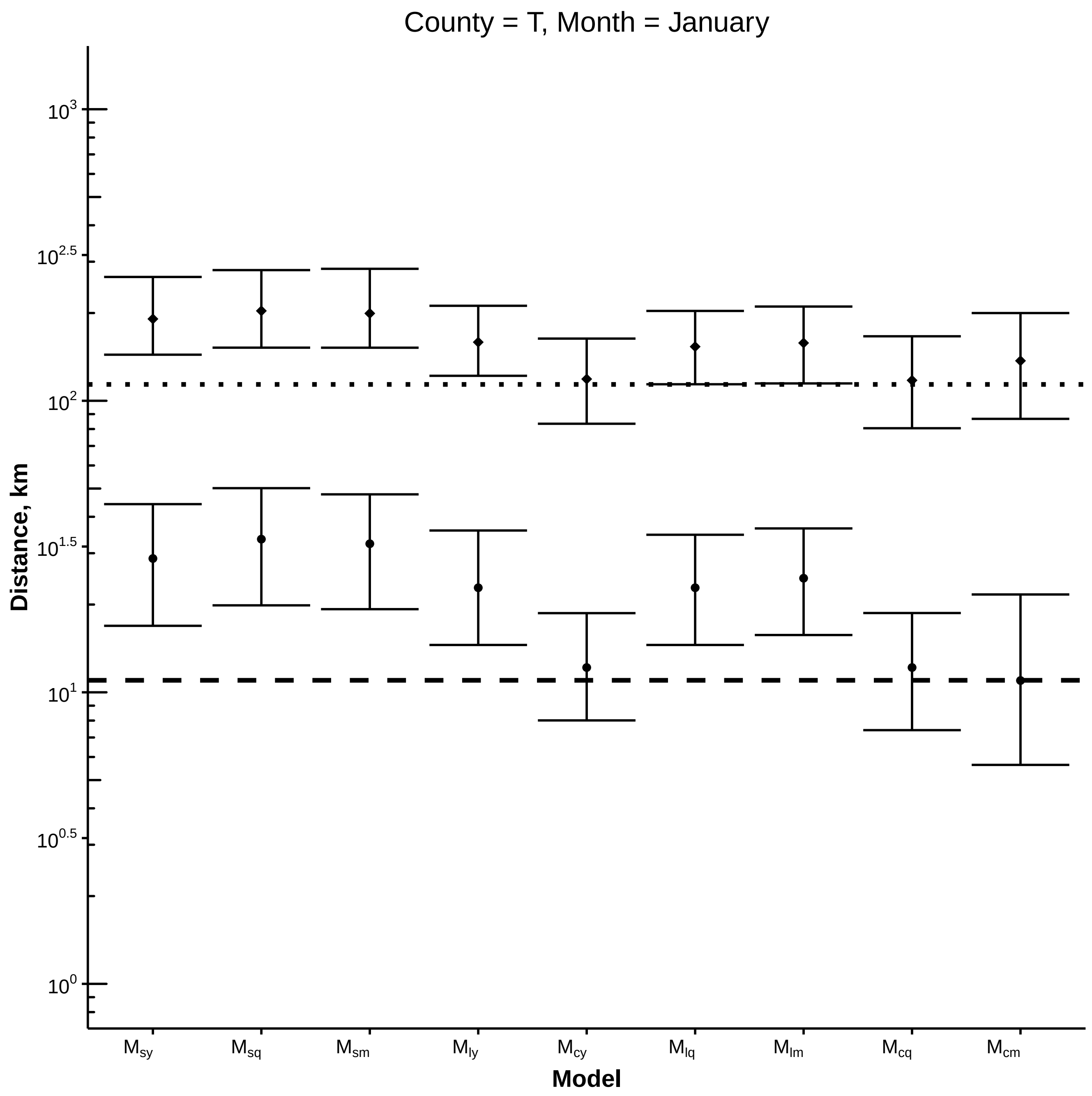
County = T, Month = January
Distance, km
10^3
$10^{2.5}$
10^2
$10^{1.5}$
10^1
$10^{0.5}$
10^0
M_{sy}
M_{sq}
M_{sm}
M_{ly}
M_{cy}
M_{lq}
M_{lm}
M_{cq}
M_{cm}
Model

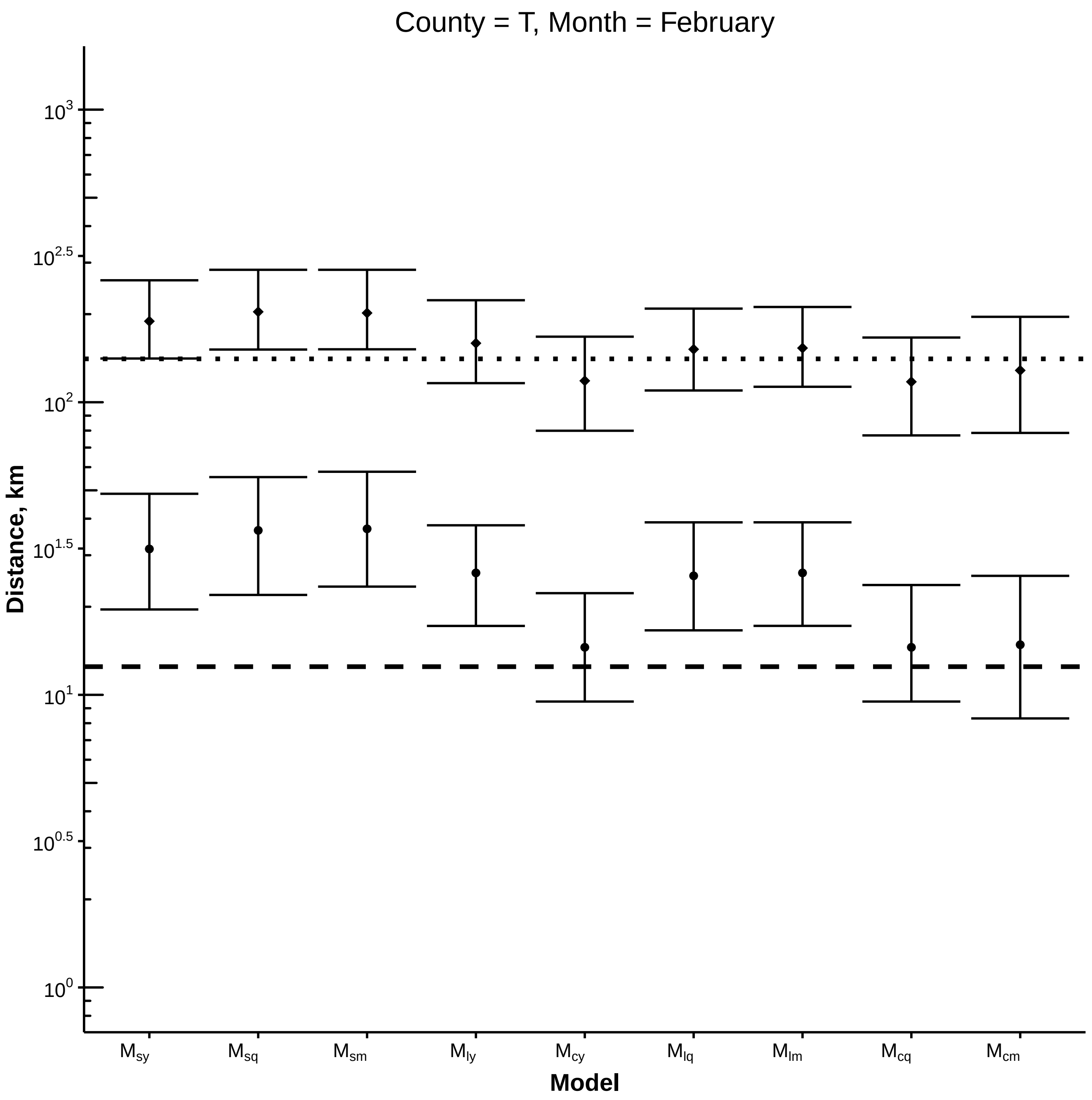

County = T, Month = February
Distance, km
10^3
$10^{2.5}$
10^2
$10^{1.5}$
10^1
$10^{0.5}$
10^0
M_{sy}
M_{sq}
M_{sm}
M_{ly}
M_{cy}
M_{lq}
M_{lm}
M_{cq}
M_{cm}
Model

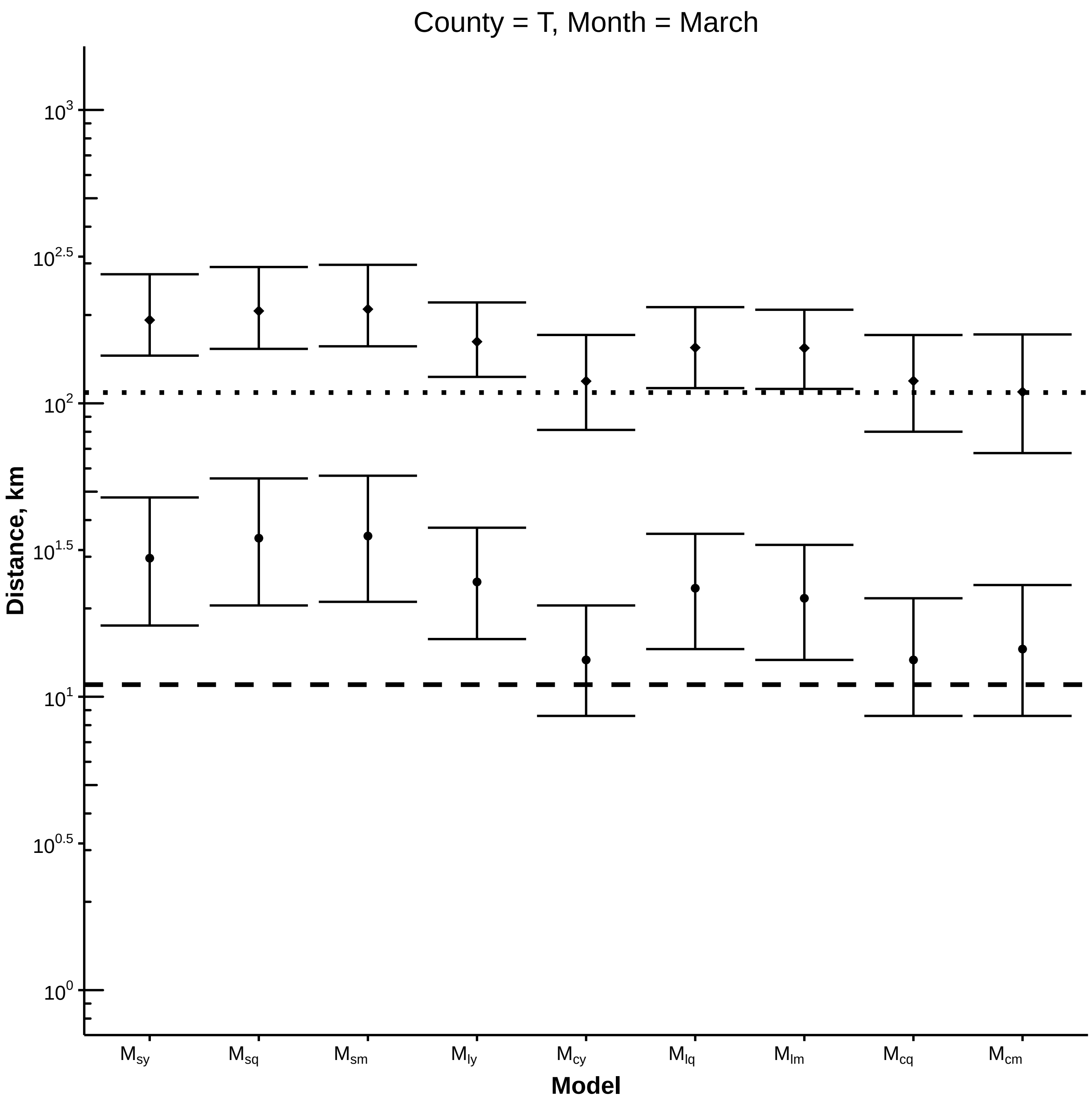

County = T, Month = March
Distance, km
10^3
$10^{2.5}$
10^2
$10^{1.5}$
10^1
$10^{0.5}$
10^0
M_{sy}
M_{sq}
M_{sm}
M_{ly}
M_{cy}
M_{lq}
M_{lm}
M_{cq}
M_{cm}
Model

County = T, Month = April

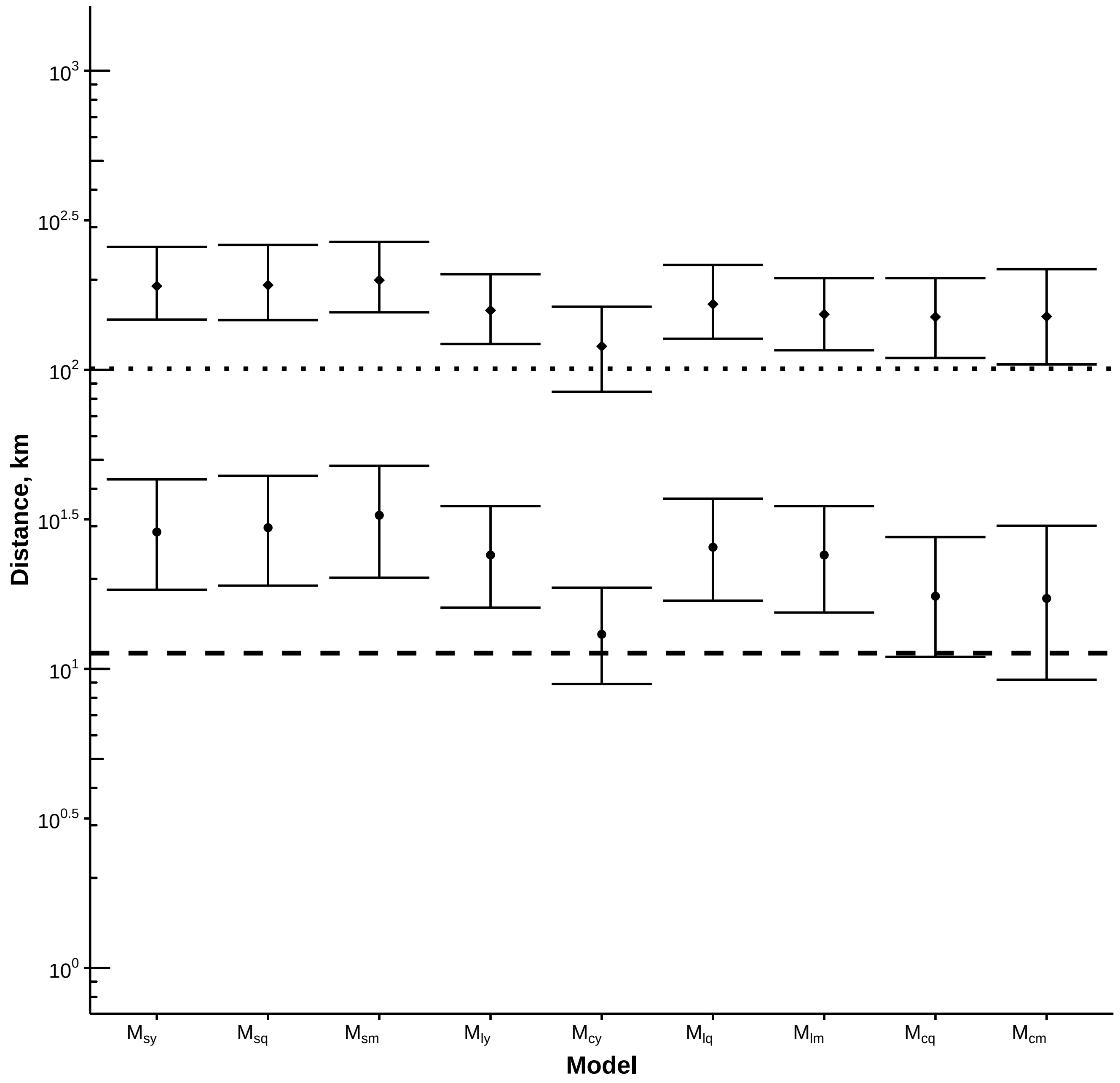

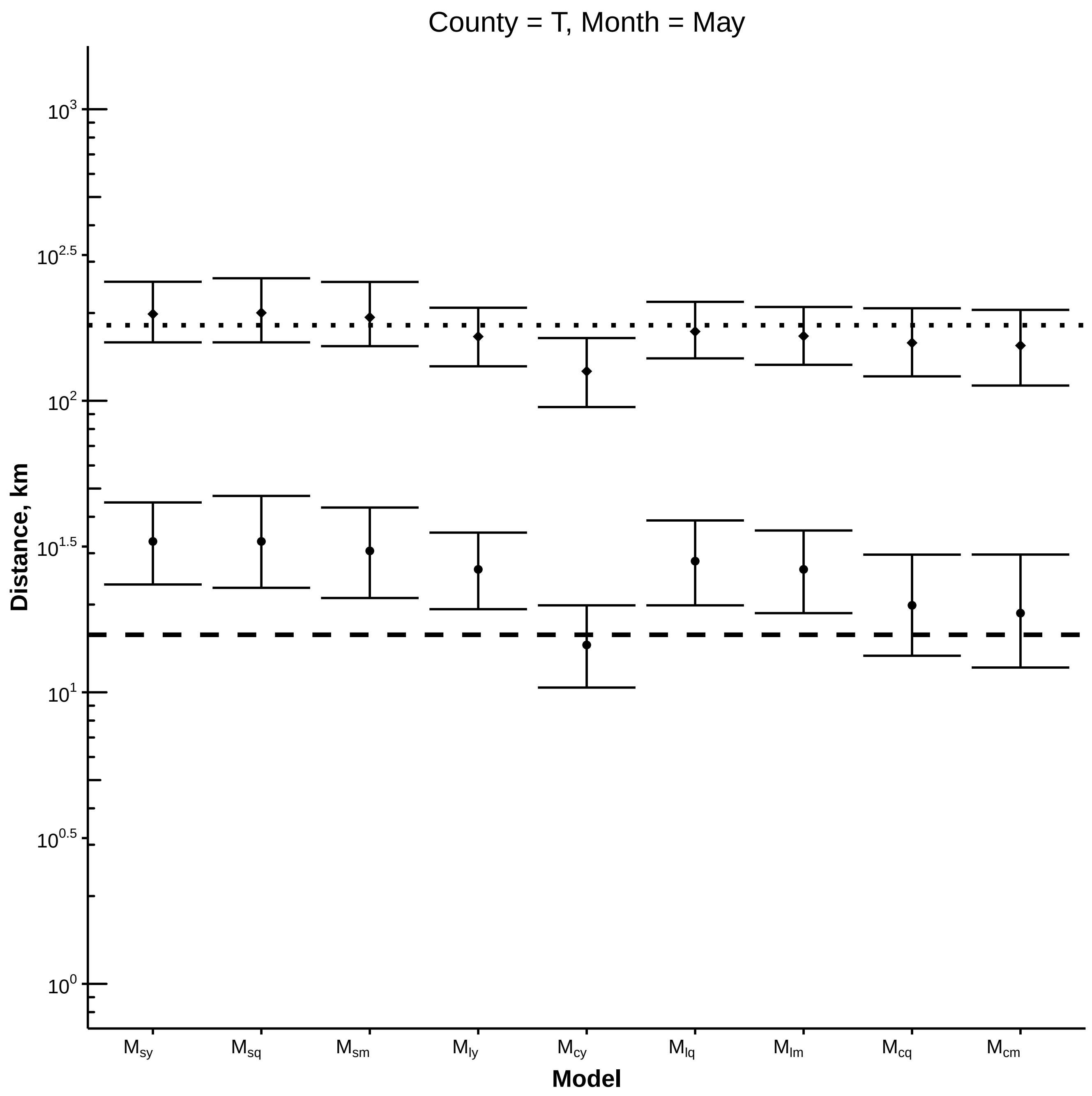

County = T, Month = May
Distance, km
10^3
$10^{2.5}$
10^2
$10^{1.5}$
10^1
$10^{0.5}$
10^0
M_{sy}
M_{sq}
M_{sm}
M_{ly}
M_{cy}
M_{lq}
M_{lm}
M_{cq}
M_{cm}
Model

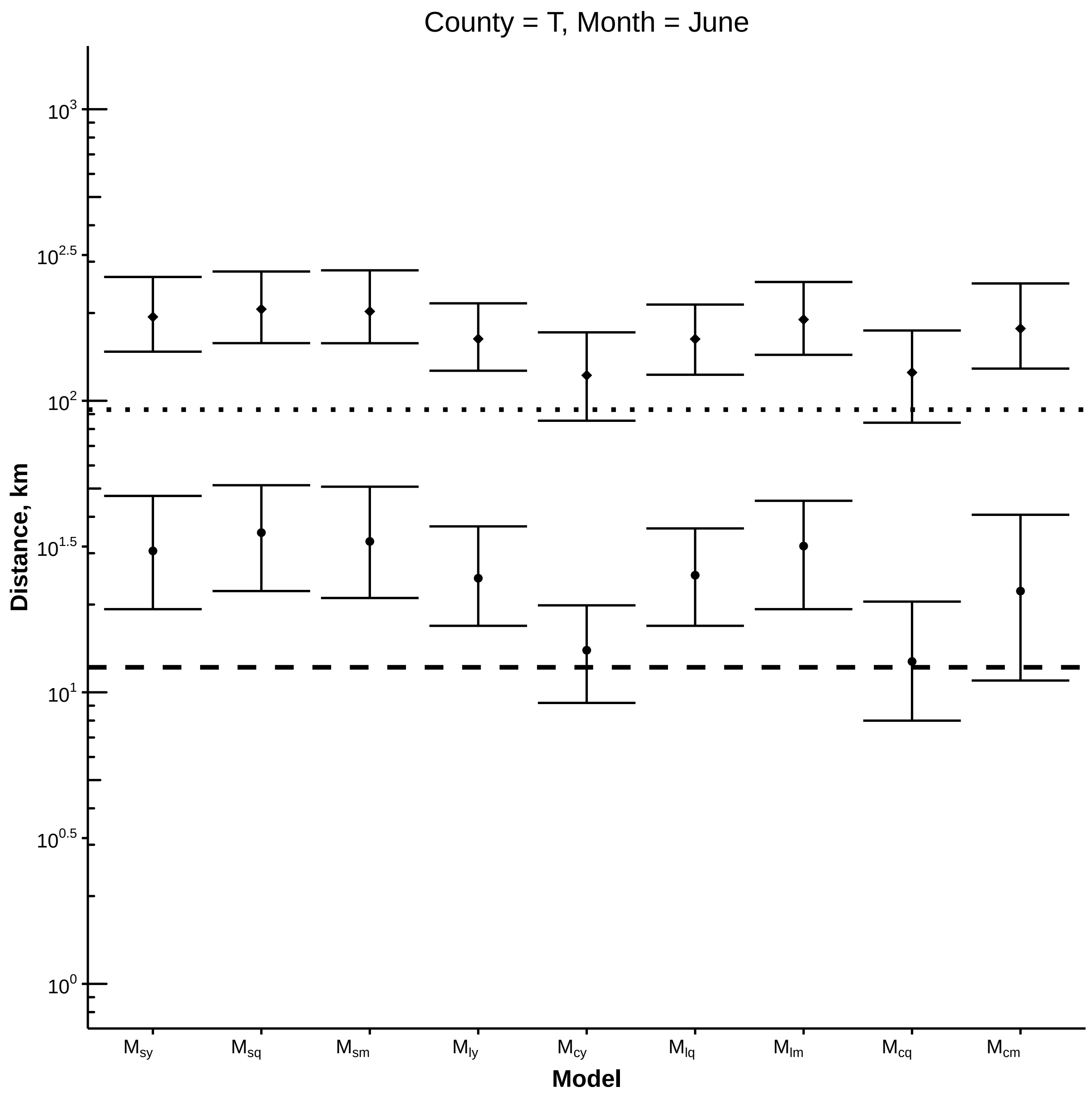

County = T, Month = June
Distance, km
10^3
$10^{2.5}$
10^2
$10^{1.5}$
10^1
$10^{0.5}$
10^0
M_{sy}
M_{sq}
M_{sm}
M_{ly}
M_{cy}
M_{lq}
M_{lm}
M_{cq}
M_{cm}
Model

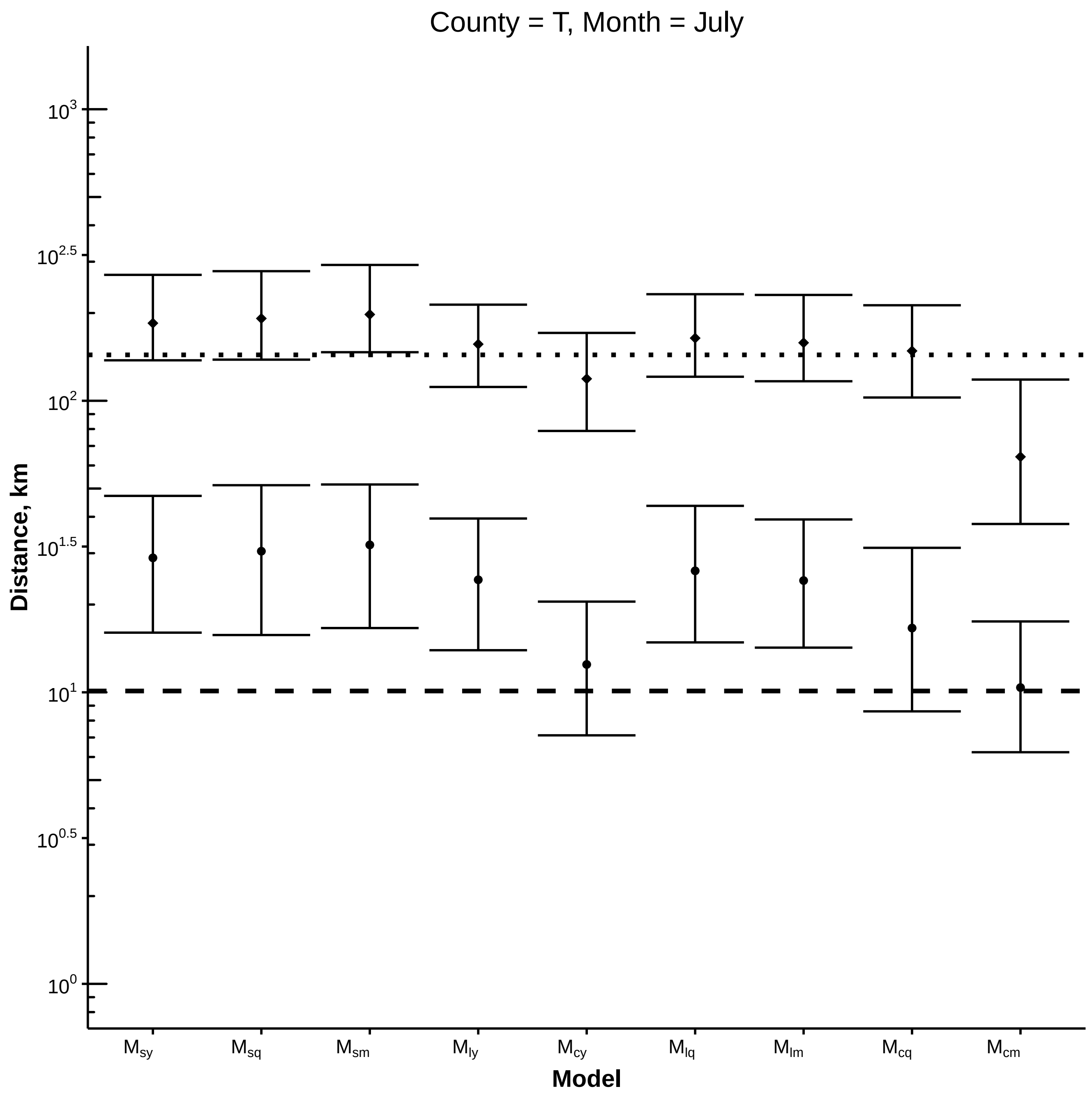

County = T, Month = July
Distance, km
10^3
10^2.5
10^2
10^1.5
10^1
10^0.5
10^0
Msy
Msq
Msm
Mly
Mcy
Mlq
Mlm
Mcq
Mcm
Model

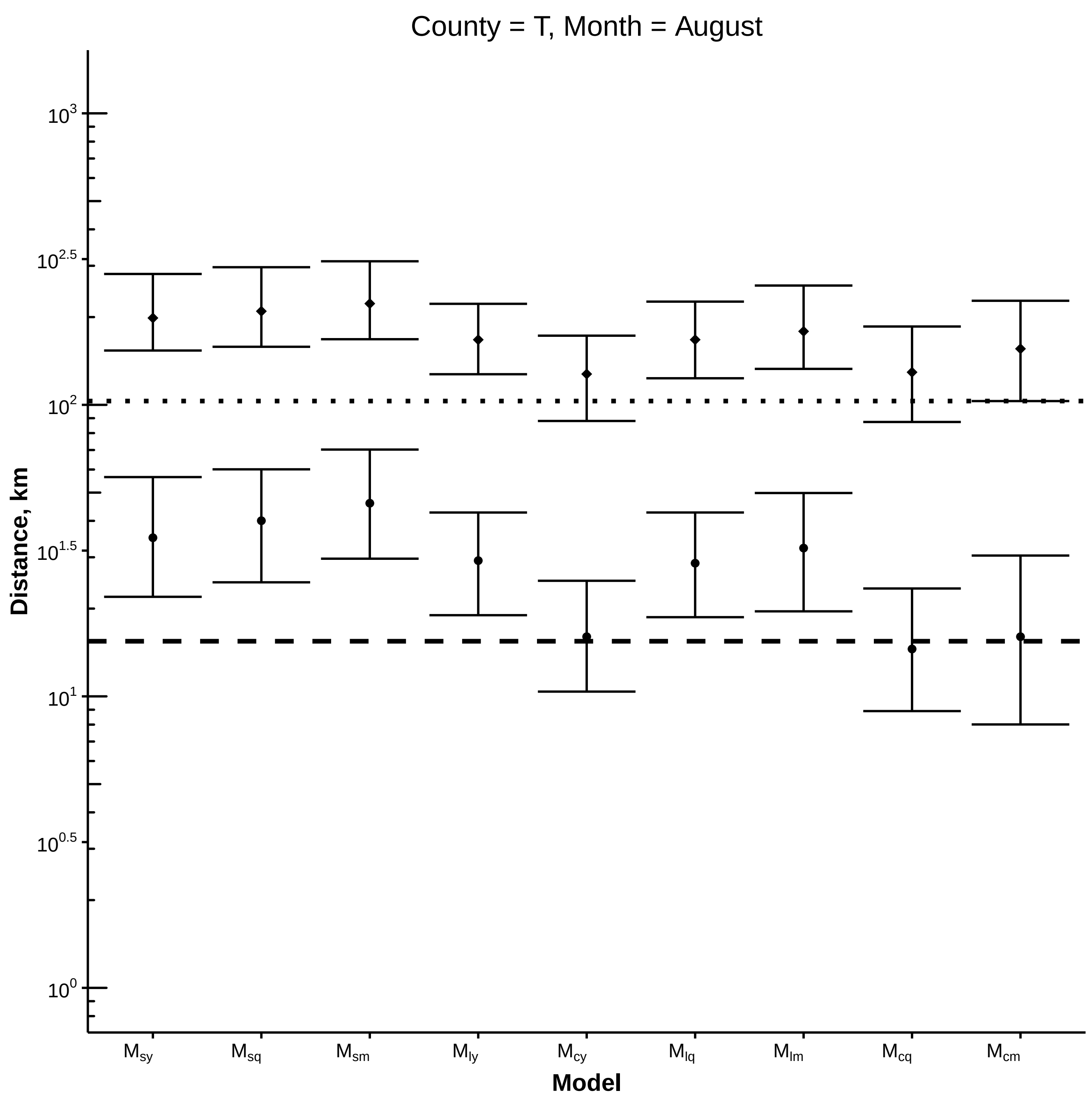
County = T, Month = August
Distance, km
10^3
10^2.5
10^2
10^1.5
10^1
10^0.5
10^0
M_sy
M_sq
M_sm
M_ly
M_cy
M_lq
M_lm
M_cq
M_cm
Model

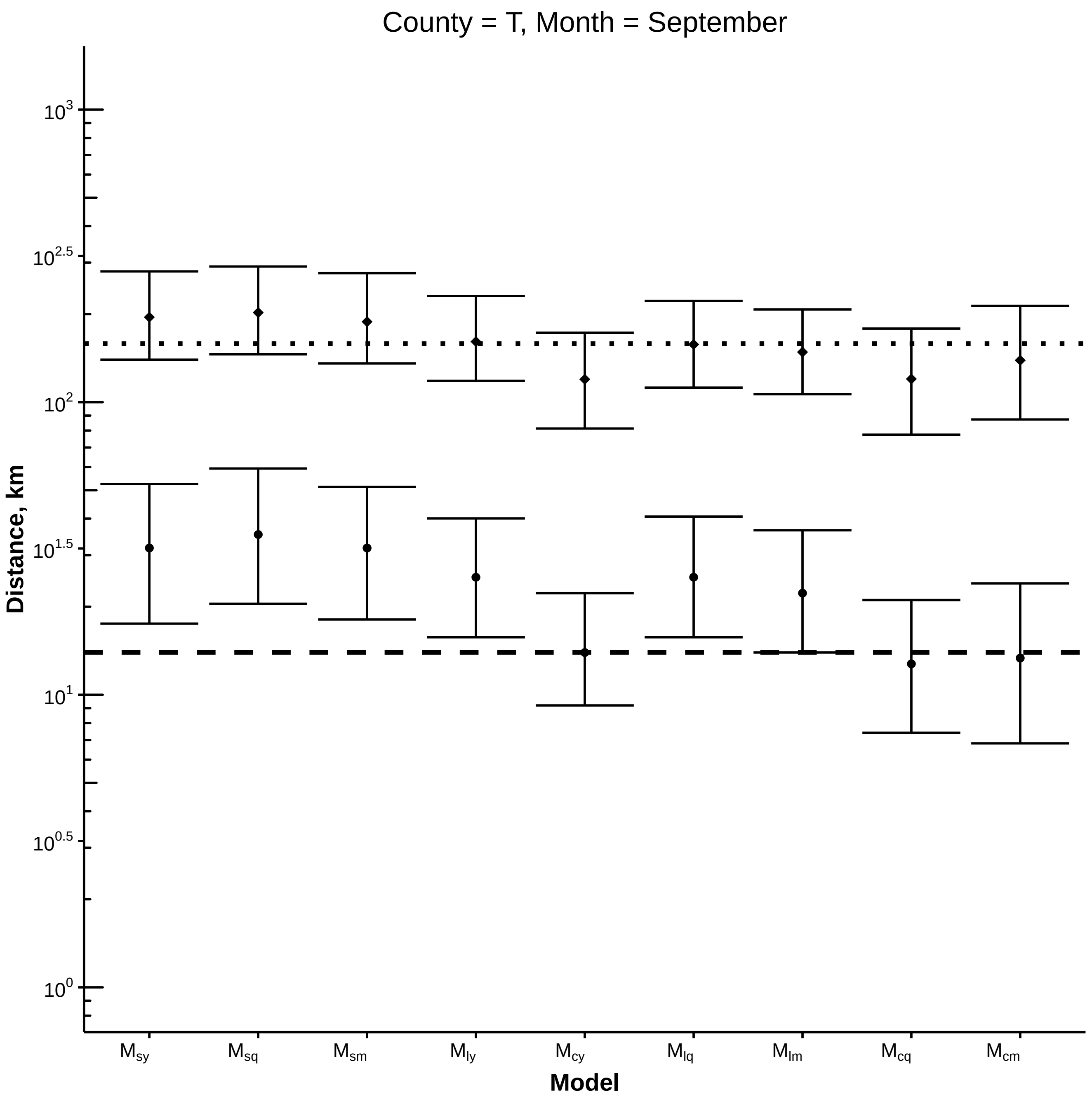

County = T, Month = September
Distance, km
10^3
$10^{2.5}$
10^2
$10^{1.5}$
10^1
$10^{0.5}$
10^0
M_{sy}
M_{sq}
M_{sm}
M_{ly}
M_{cy}
M_{lq}
M_{lm}
M_{cq}
M_{cm}
Model

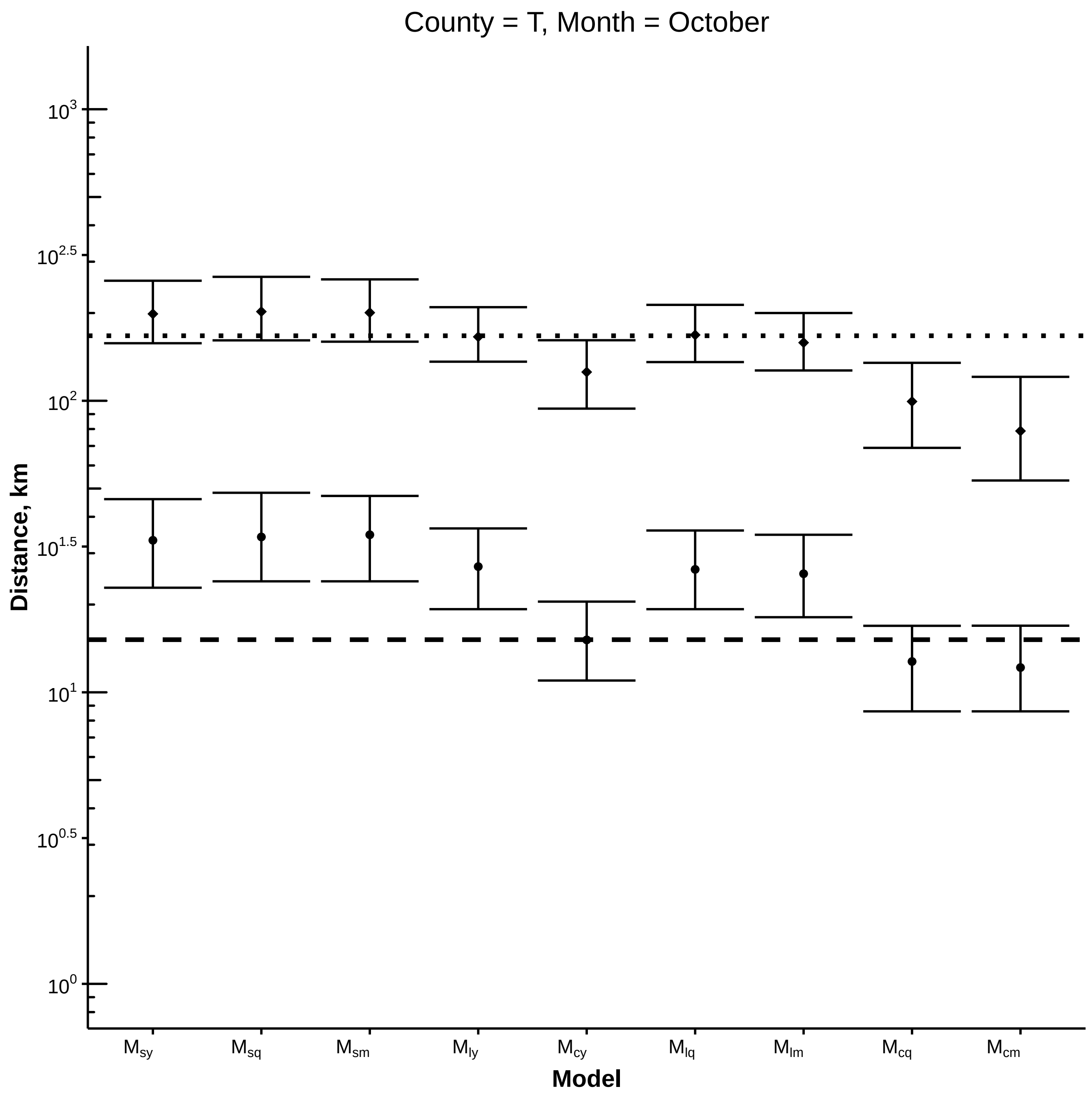
County = T, Month = October
Distance, km
10^3
$10^{2.5}$
10^2
$10^{1.5}$
10^1
$10^{0.5}$
10^0
Model
M_{sy}
M_{sq}
M_{sm}
M_{ly}
M_{cy}
M_{lq}
M_{lm}
M_{cq}
M_{cm}

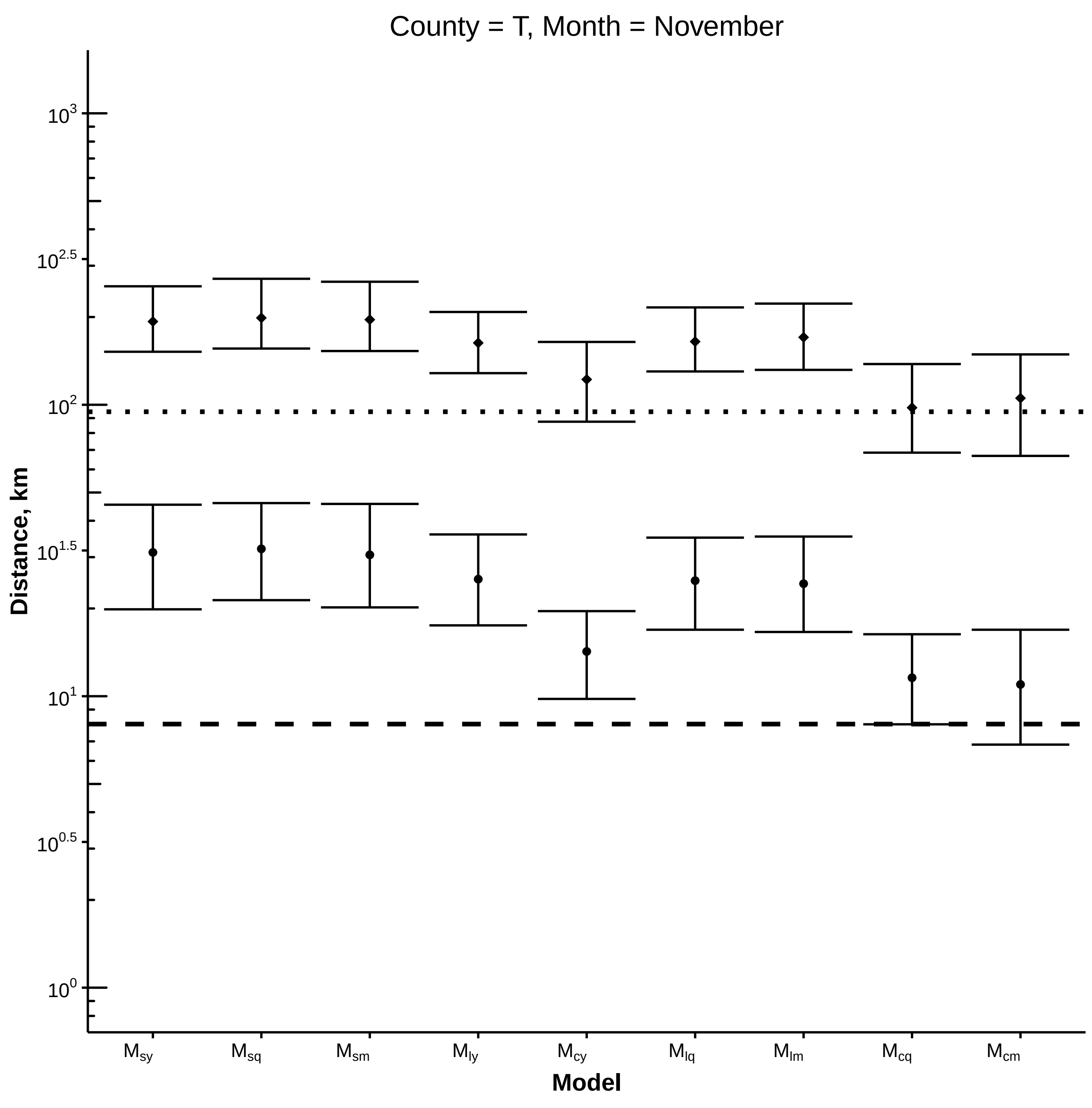
County = T, Month = November
Distance, km
10^3
$10^{2.5}$
10^2
$10^{1.5}$
10^1
$10^{0.5}$
10^0
M_{sy}
M_{sq}
M_{sm}
M_{ly}
M_{cy}
M_{lq}
M_{lm}
M_{cq}
M_{cm}
Model

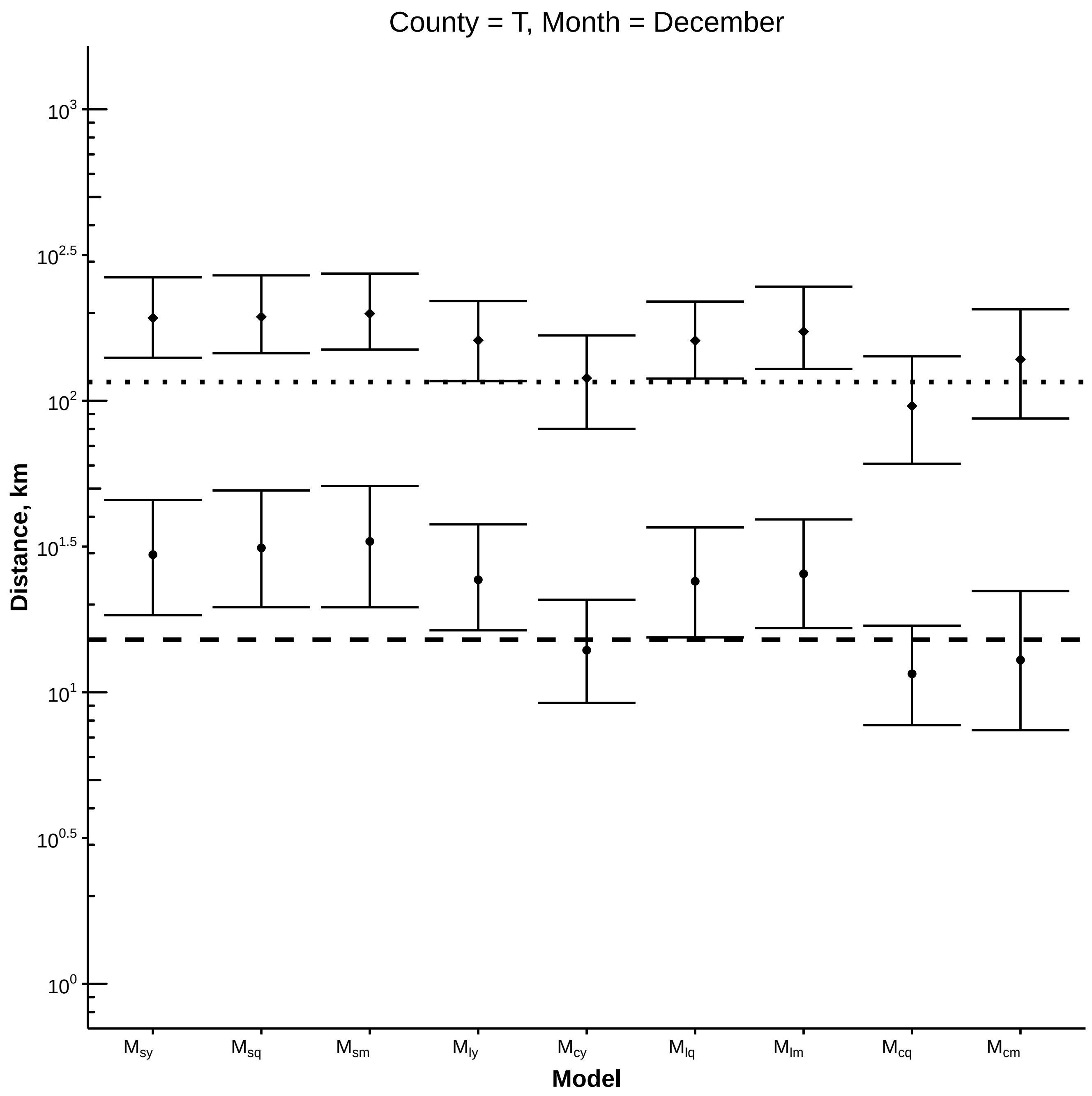

County = T, Month = December
Distance, km
10^3
$10^{2.5}$
10^2
$10^{1.5}$
10^1
$10^{0.5}$
10^0
M_{sy}
M_{sq}
M_{sm}
M_{ly}
M_{cy}
M_{lq}
M_{lm}
M_{cq}
M_{cm}
Model

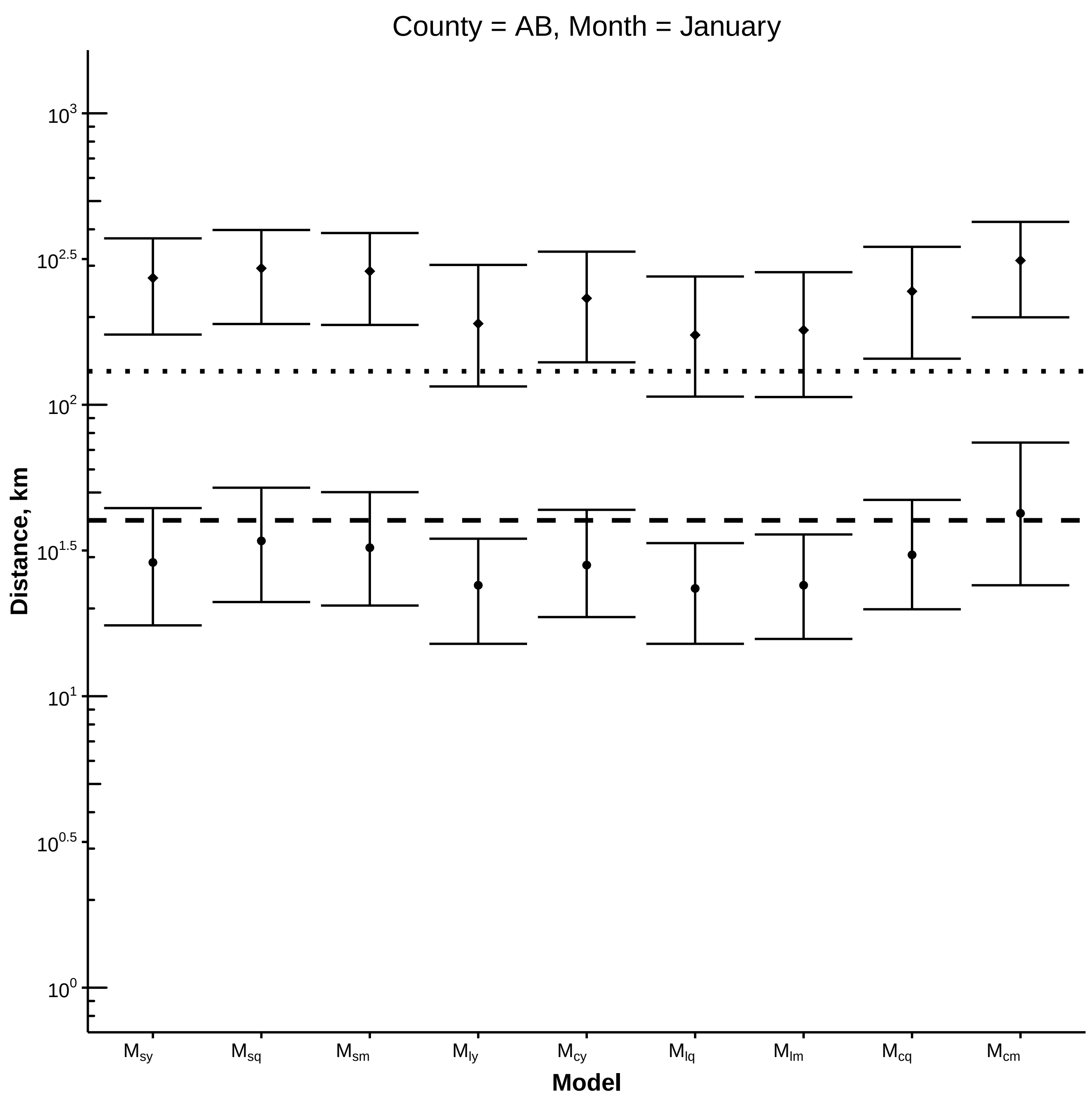

County = AB, Month = January
Distance, km
10^3
$10^{2.5}$
10^2
$10^{1.5}$
10^1
$10^{0.5}$
10^0
M_{sy}
M_{sq}
M_{sm}
M_{ly}
M_{cy}
M_{lq}
M_{lm}
M_{cq}
M_{cm}
Model

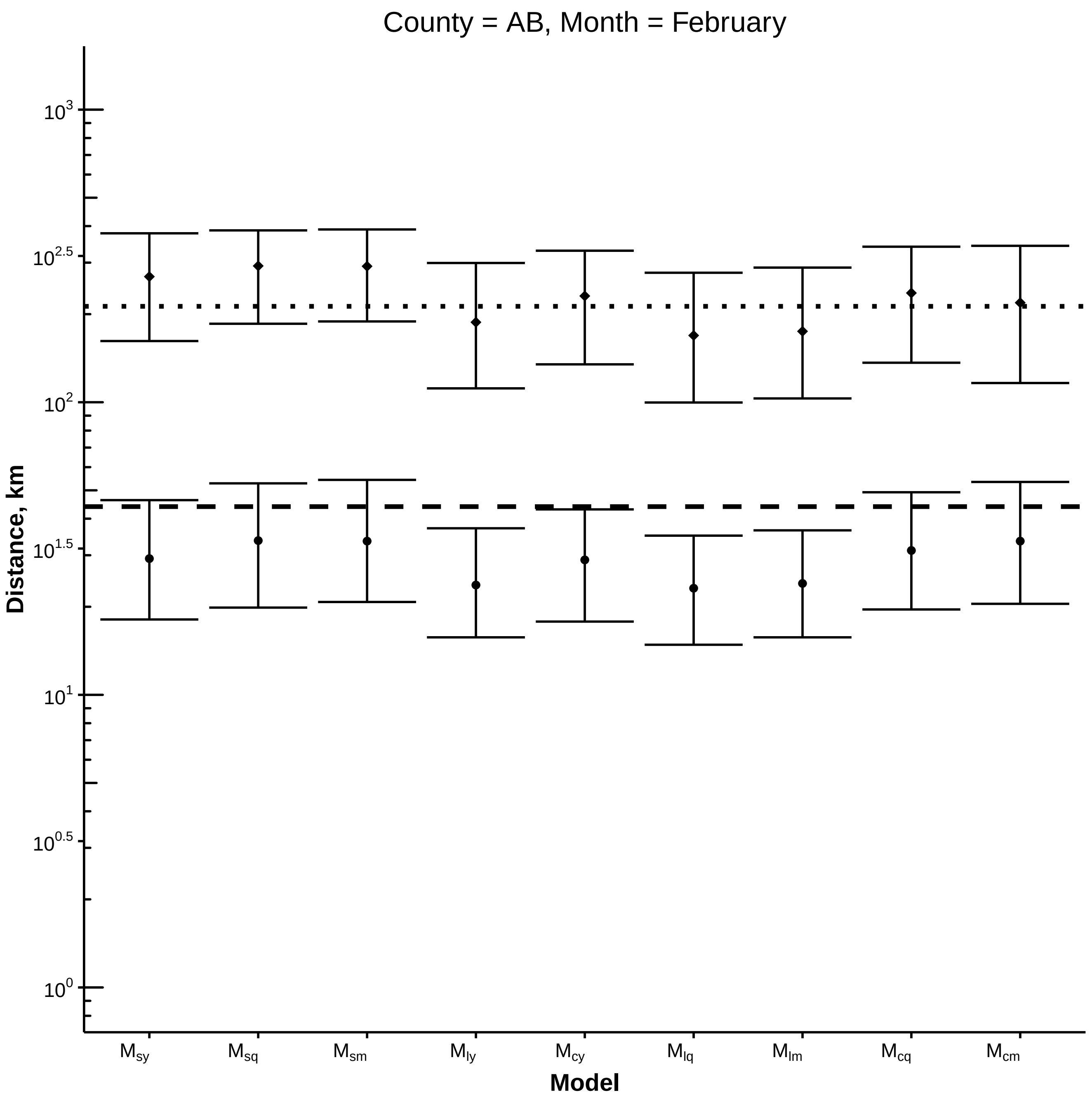

County = AB, Month = February
Distance, km
10^3
$10^{2.5}$
10^2
$10^{1.5}$
10^1
$10^{0.5}$
10^0
M_{sy}
M_{sq}
M_{sm}
M_{ly}
M_{cy}
M_{lq}
M_{lm}
M_{cq}
M_{cm}
Model

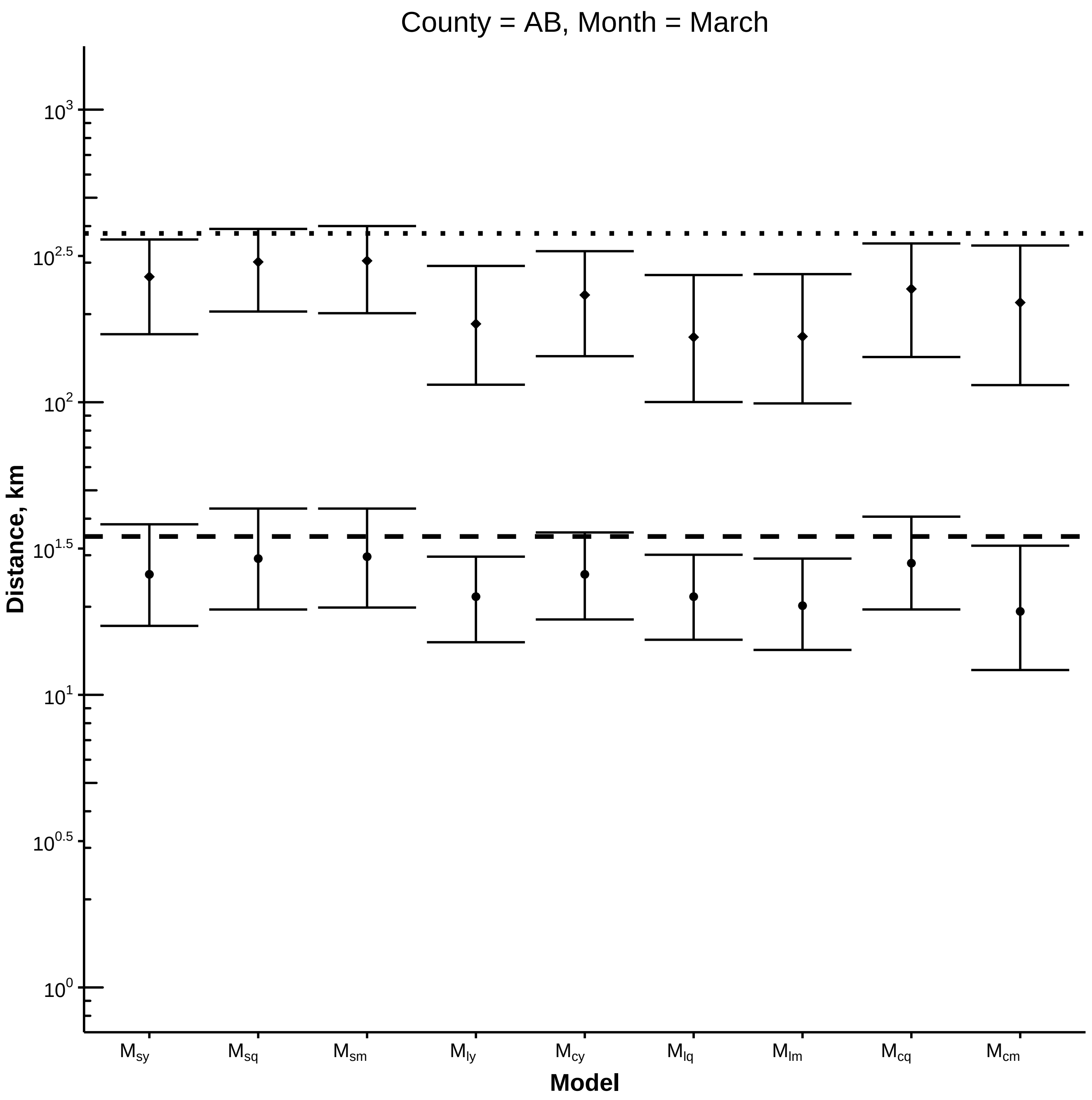

County = AB, Month = March
Distance, km
10^3
$10^{2.5}$
10^2
$10^{1.5}$
10^1
$10^{0.5}$
10^0
M_{sy}
M_{sq}
M_{sm}
M_{ly}
M_{cy}
M_{lq}
M_{lm}
M_{cq}
M_{cm}
Model

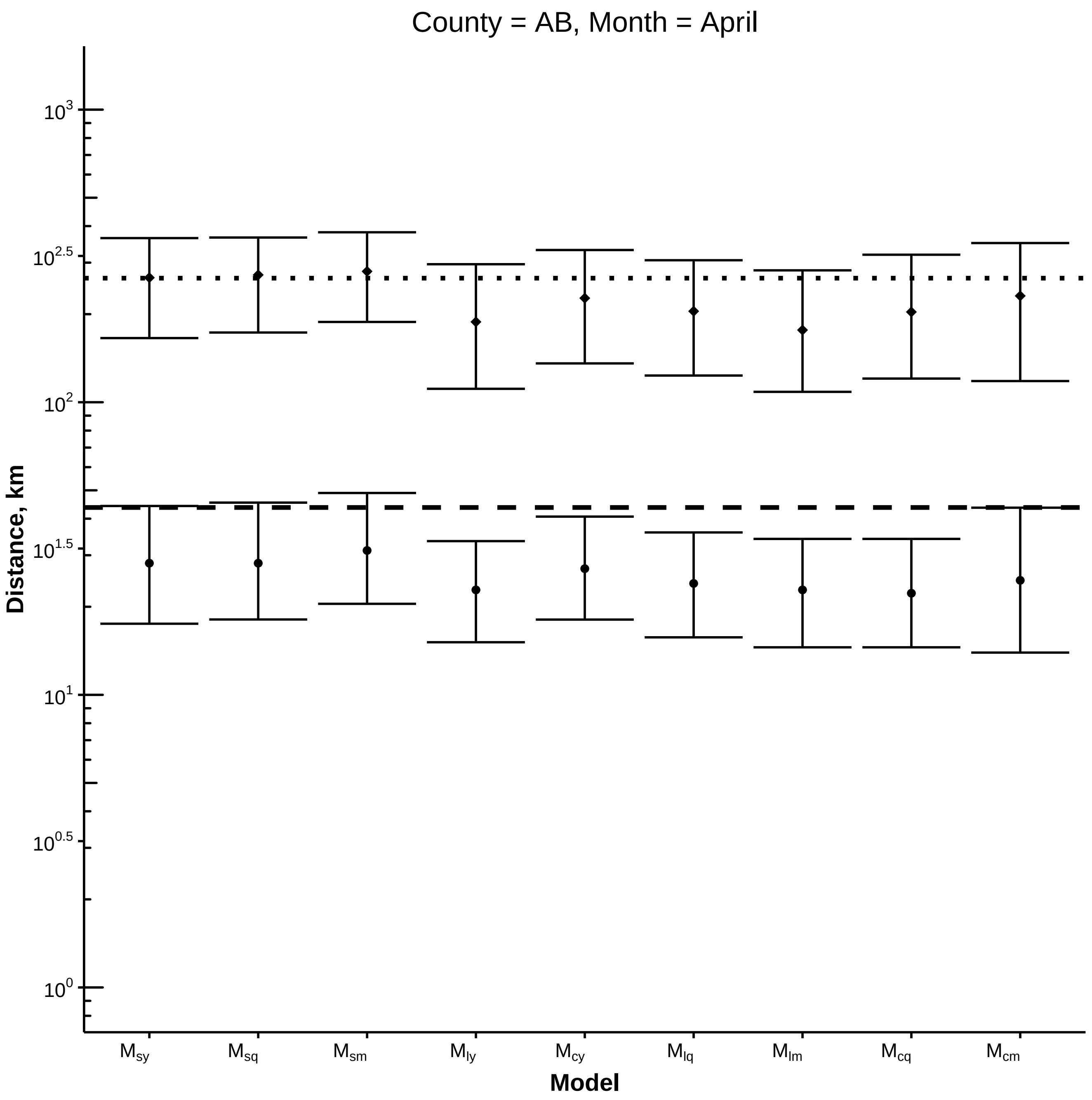

County = AB, Month = April
Distance, km
10^3
$10^{2.5}$
10^2
$10^{1.5}$
10^1
$10^{0.5}$
10^0
M_{sy}
M_{sq}
M_{sm}
M_{ly}
M_{cy}
M_{lq}
M_{lm}
M_{cq}
M_{cm}
Model

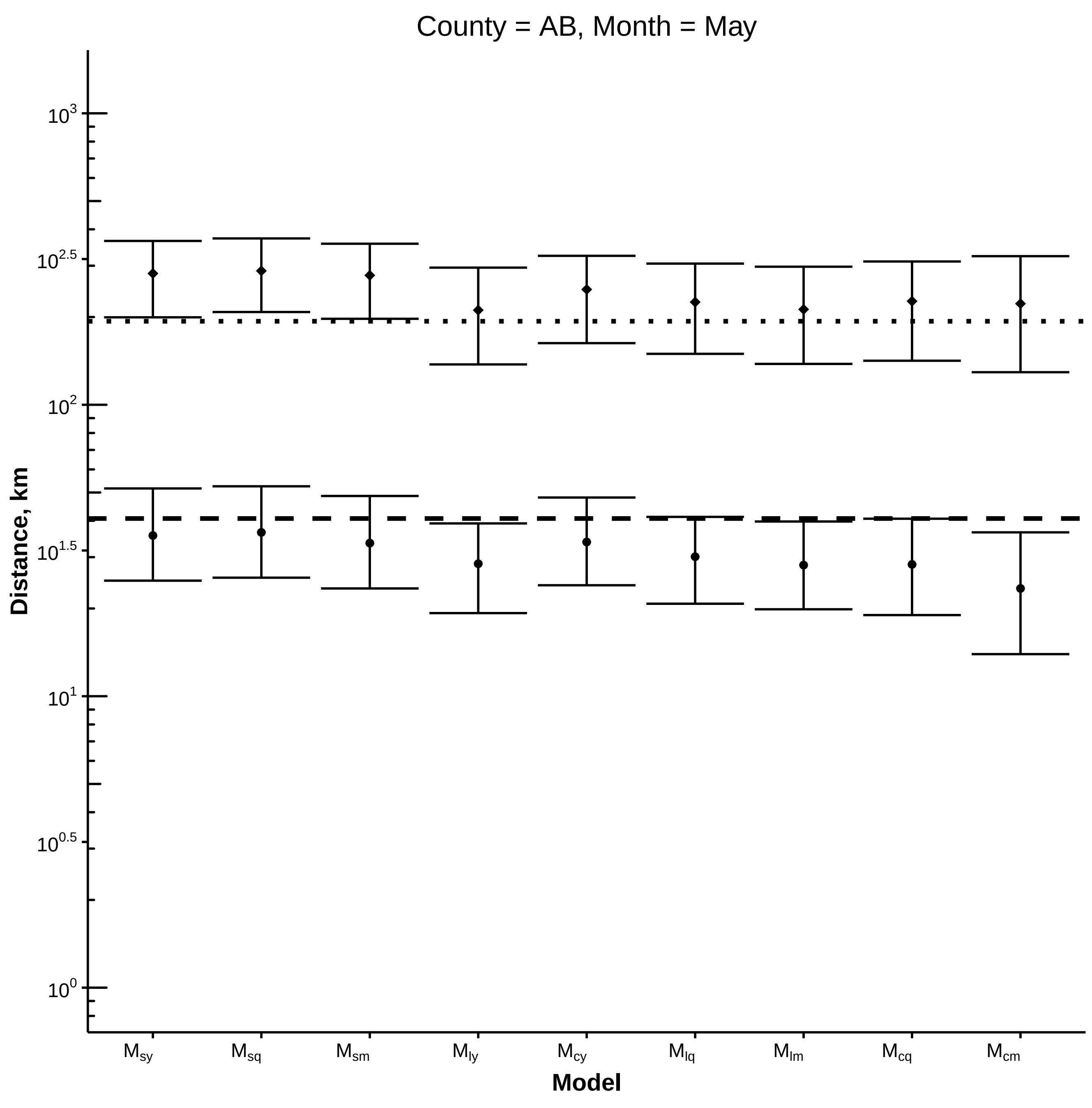

County = AB, Month = May
Distance, km
10^{3}
$10^{2.5}$
10^{2}
$10^{1.5}$
10^{1}
$10^{0.5}$
10^{0}
M_{sy}
M_{sq}
M_{sm}
M_{ly}
M_{cy}
M_{lq}
M_{lm}
M_{cq}
M_{cm}
Model

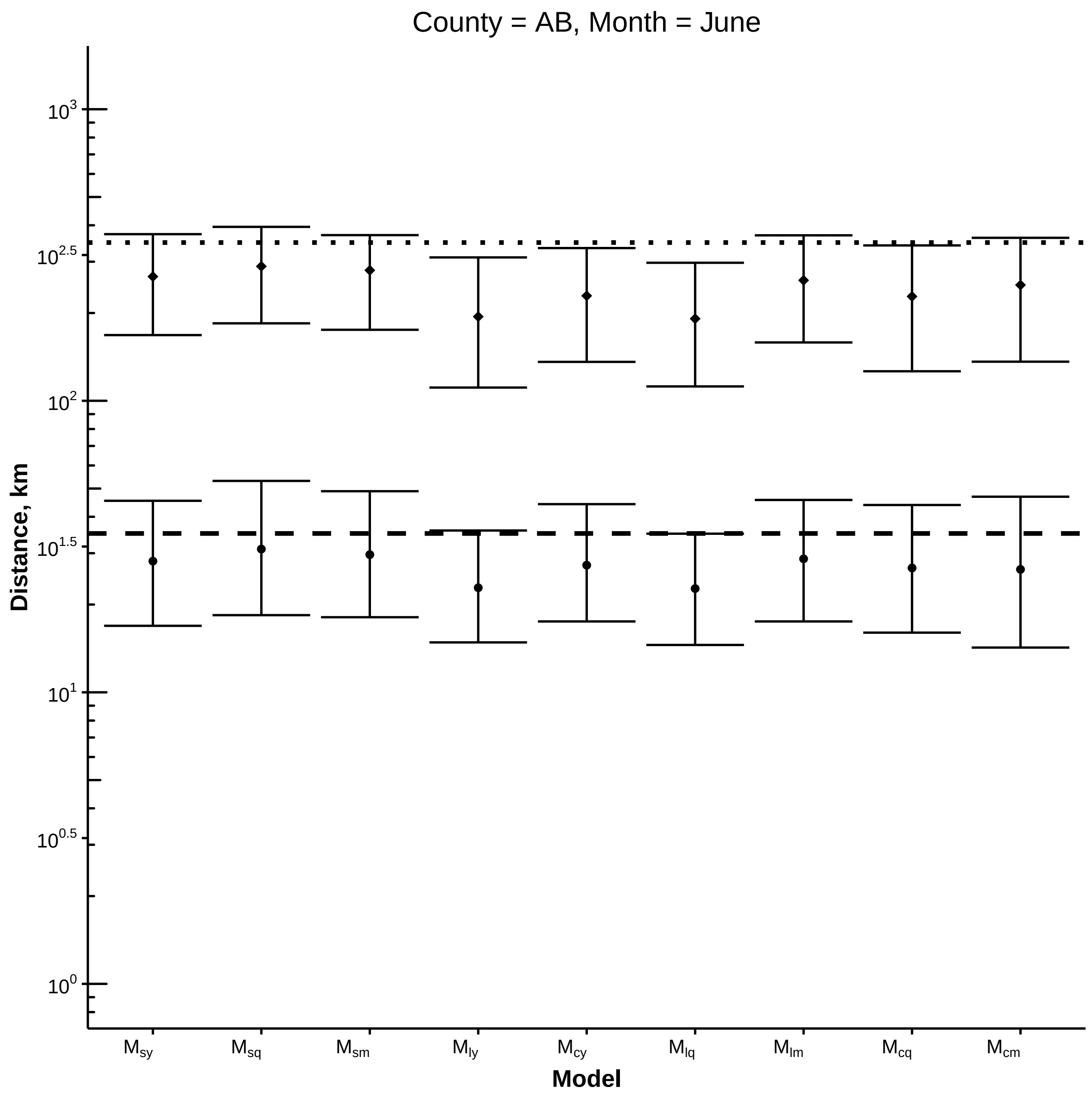

County = AB, Month = June
Distance, km
10^3
10^2.5
10^2
10^1.5
10^1
10^0.5
10^0
M_sy
M_sq
M_sm
M_ly
M_cy
M_lq
M_lm
M_cq
M_cm
Model

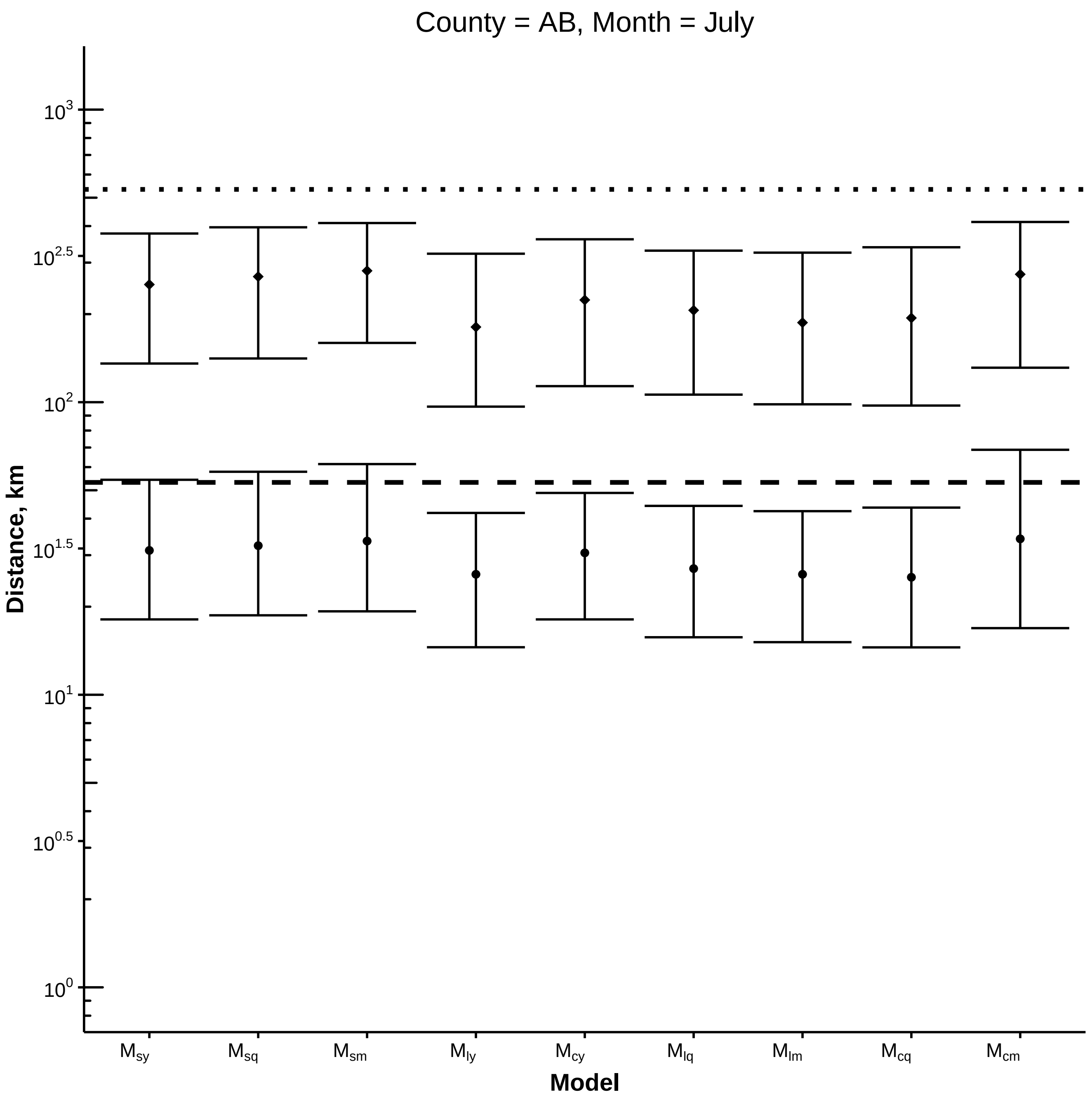

County = AB, Month = July
Distance, km
10^3
$10^{2.5}$
10^2
$10^{1.5}$
10^1
$10^{0.5}$
10^0
M_{sy}
M_{sq}
M_{sm}
M_{ly}
M_{cy}
M_{lq}
M_{lm}
M_{cq}
M_{cm}
Model

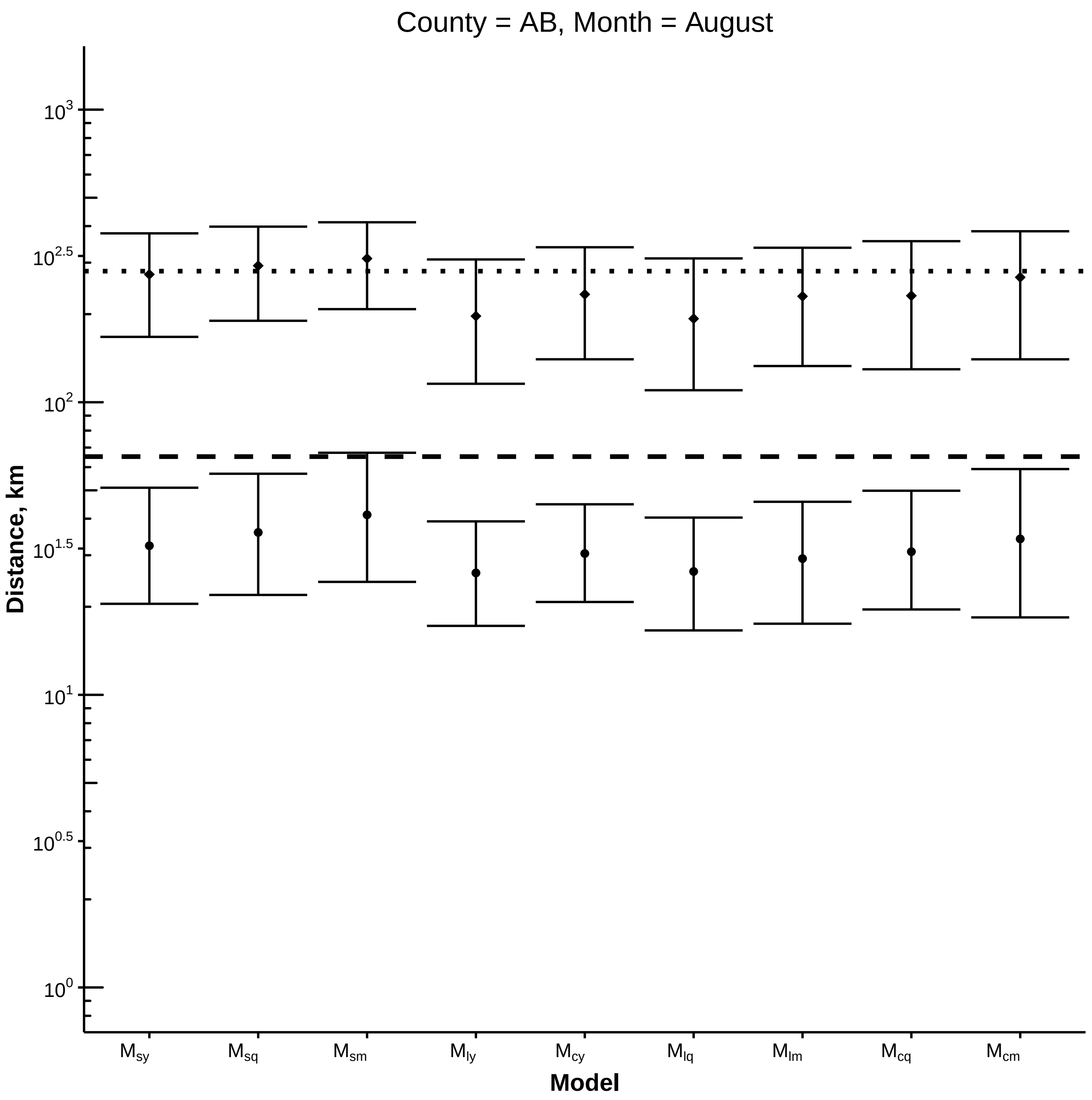

County = AB, Month = August
Distance, km
10^3
$10^{2.5}$
10^2
$10^{1.5}$
10^1
$10^{0.5}$
10^0
M_{sy}
M_{sq}
M_{sm}
M_{ly}
M_{cy}
M_{lq}
M_{lm}
M_{cq}
M_{cm}
Model

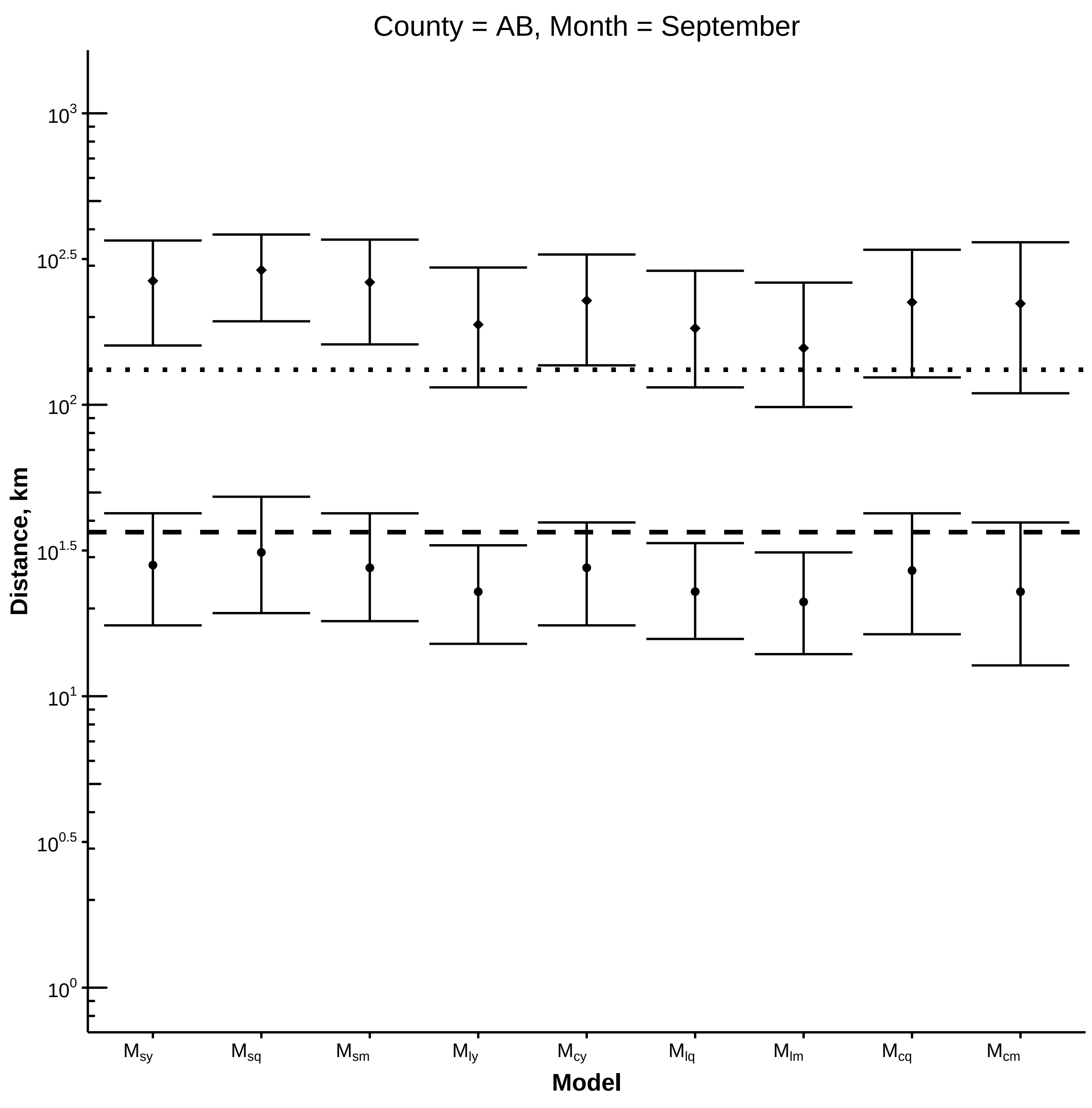

County = AB, Month = September
Distance, km
10^3
$10^{2.5}$
10^2
$10^{1.5}$
10^1
$10^{0.5}$
10^0
M_{sy}
M_{sq}
M_{sm}
M_{ly}
M_{cy}
M_{lq}
M_{lm}
M_{cq}
M_{cm}
Model

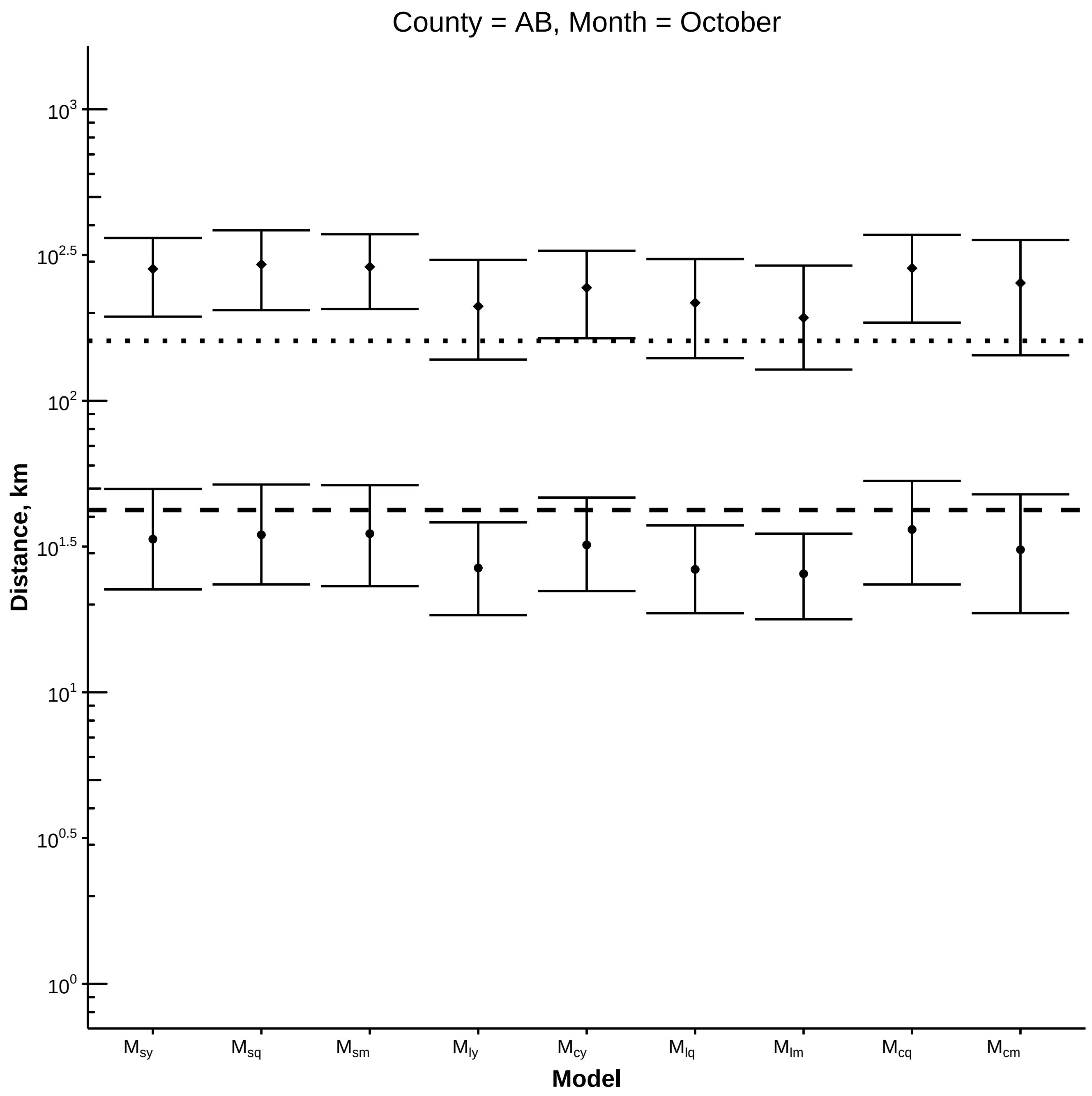

County = AB, Month = October
Distance, km
10^3
$10^{2.5}$
10^2
$10^{1.5}$
10^1
$10^{0.5}$
10^0
M_{sy}
M_{sq}
M_{sm}
M_{ly}
M_{cy}
M_{lq}
M_{lm}
M_{cq}
M_{cm}
Model

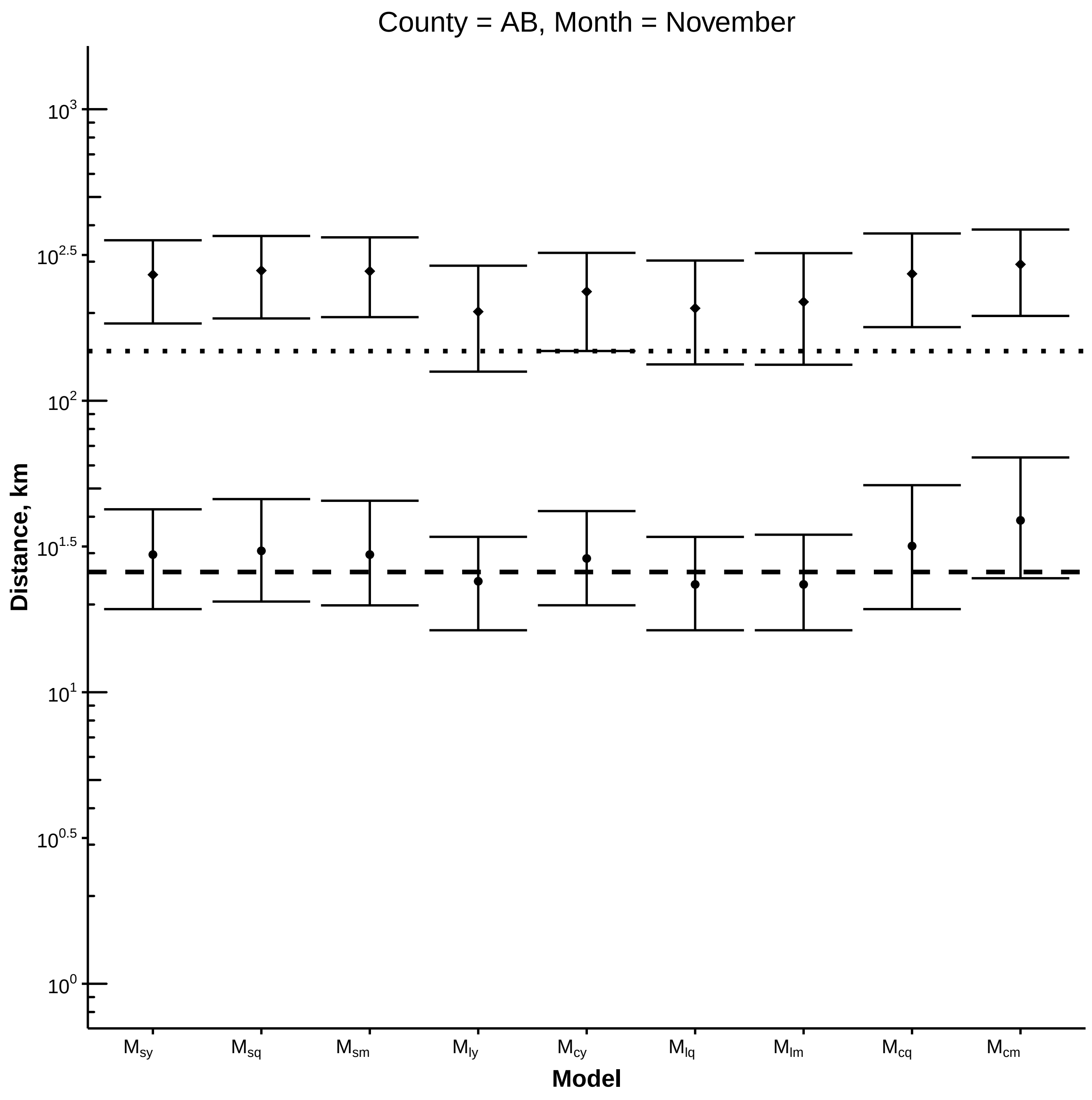

County = AB, Month = November
Distance, km
10^3
$10^{2.5}$
10^2
$10^{1.5}$
10^1
$10^{0.5}$
10^0
M_{sy}
M_{sq}
M_{sm}
M_{ly}
M_{cy}
M_{lq}
M_{lm}
M_{cq}
M_{cm}
Model

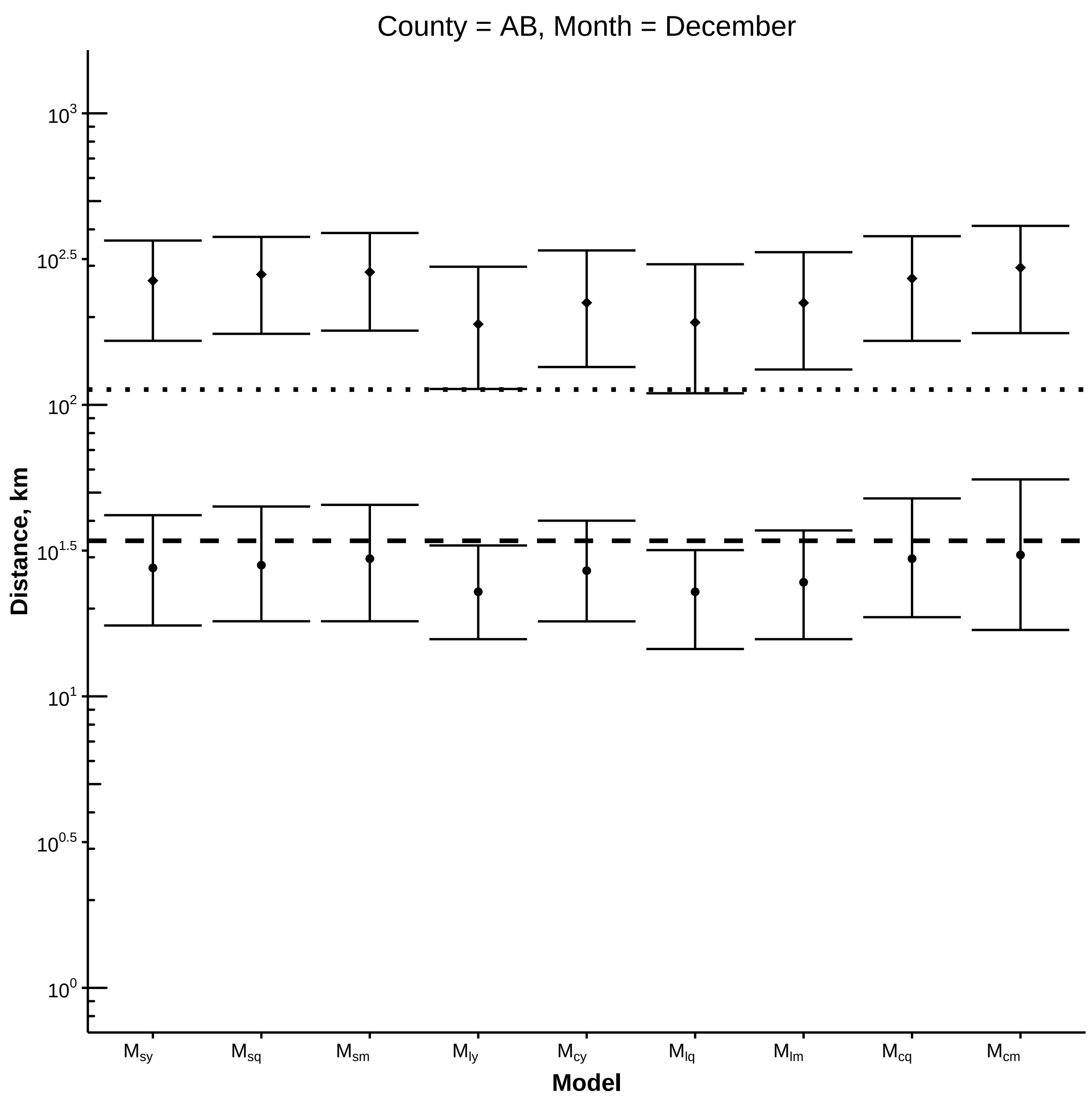

County = AB, Month = December
Distance, km
10^3
10^2.5
10^2
10^1.5
10^1
10^0.5
10^0
M_sy
M_sq
M_sm
M_ly
M_cy
M_lq
M_lm
M_cq
M_cm
Model

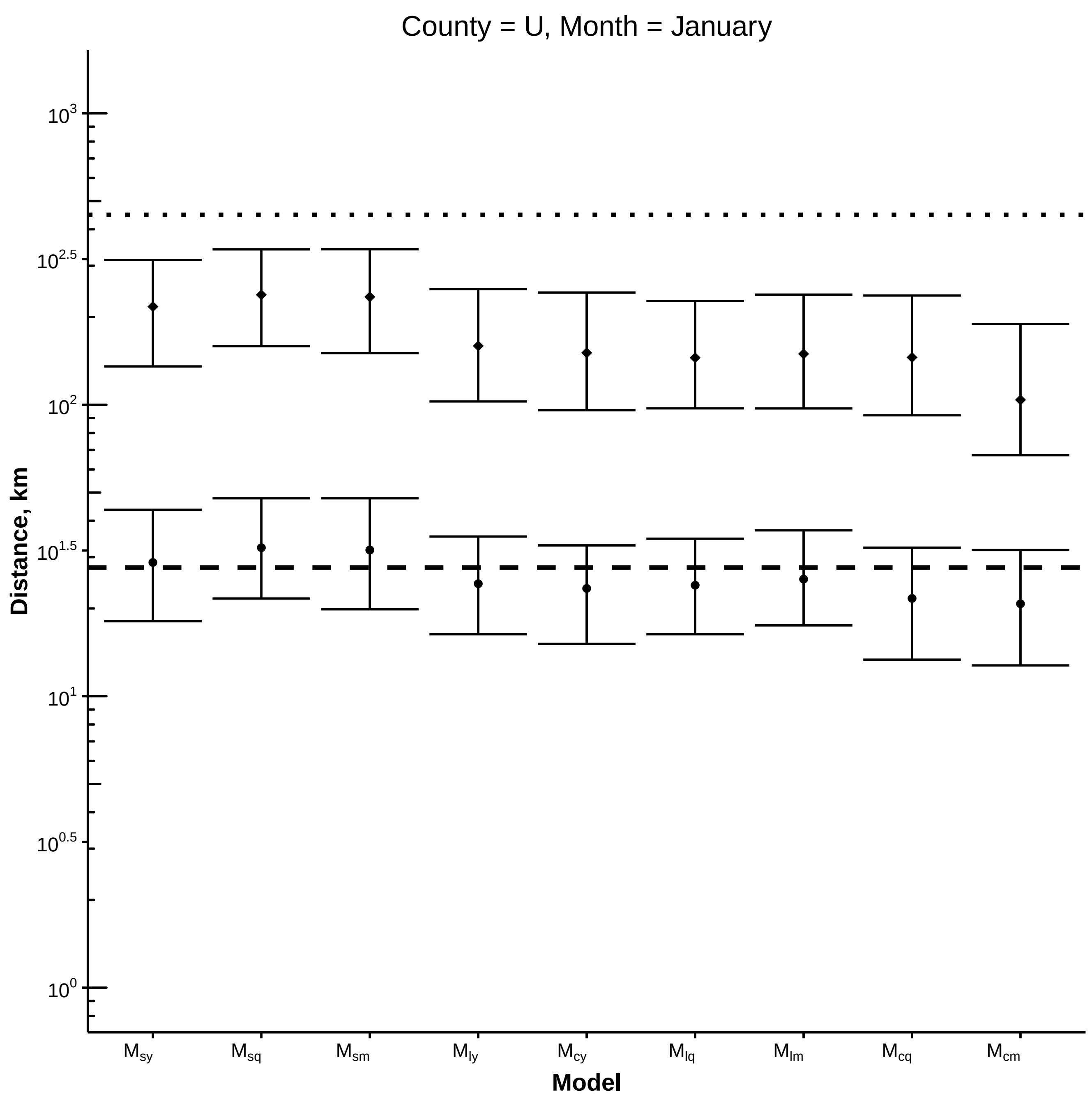

County = U, Month = January
Distance, km
10^{3}
$10^{2.5}$
10^{2}
$10^{1.5}$
10^{1}
$10^{0.5}$
10^{0}
M_{sy}
M_{sq}
M_{sm}
M_{ly}
M_{cy}
M_{lq}
M_{lm}
M_{cq}
M_{cm}
Model

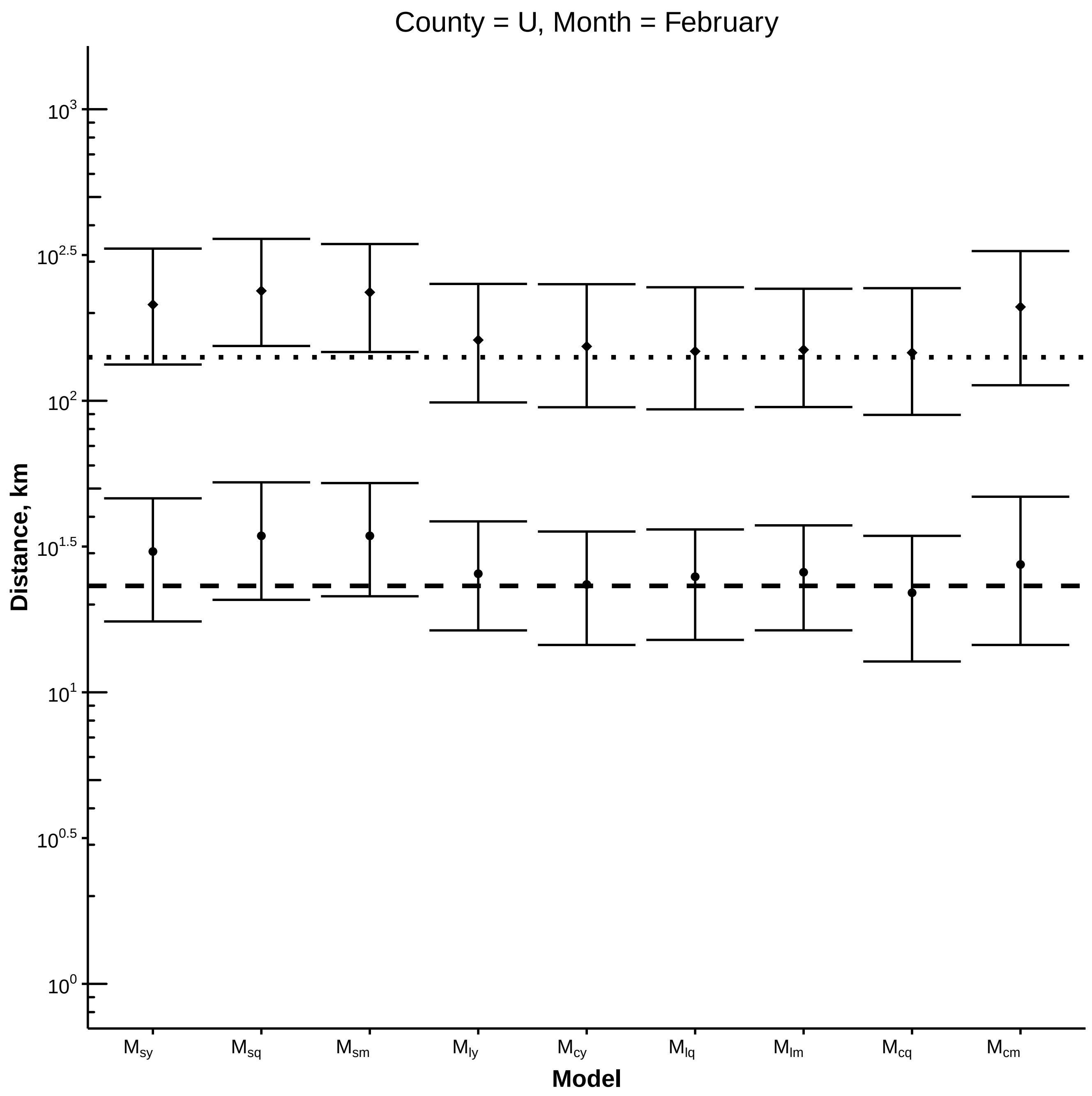

County = U, Month = February
Distance, km
10^3
$10^{2.5}$
10^2
$10^{1.5}$
10^1
$10^{0.5}$
10^0
Model
M_{sy}
M_{sq}
M_{sm}
M_{ly}
M_{cy}
M_{lq}
M_{lm}
M_{cq}
M_{cm}

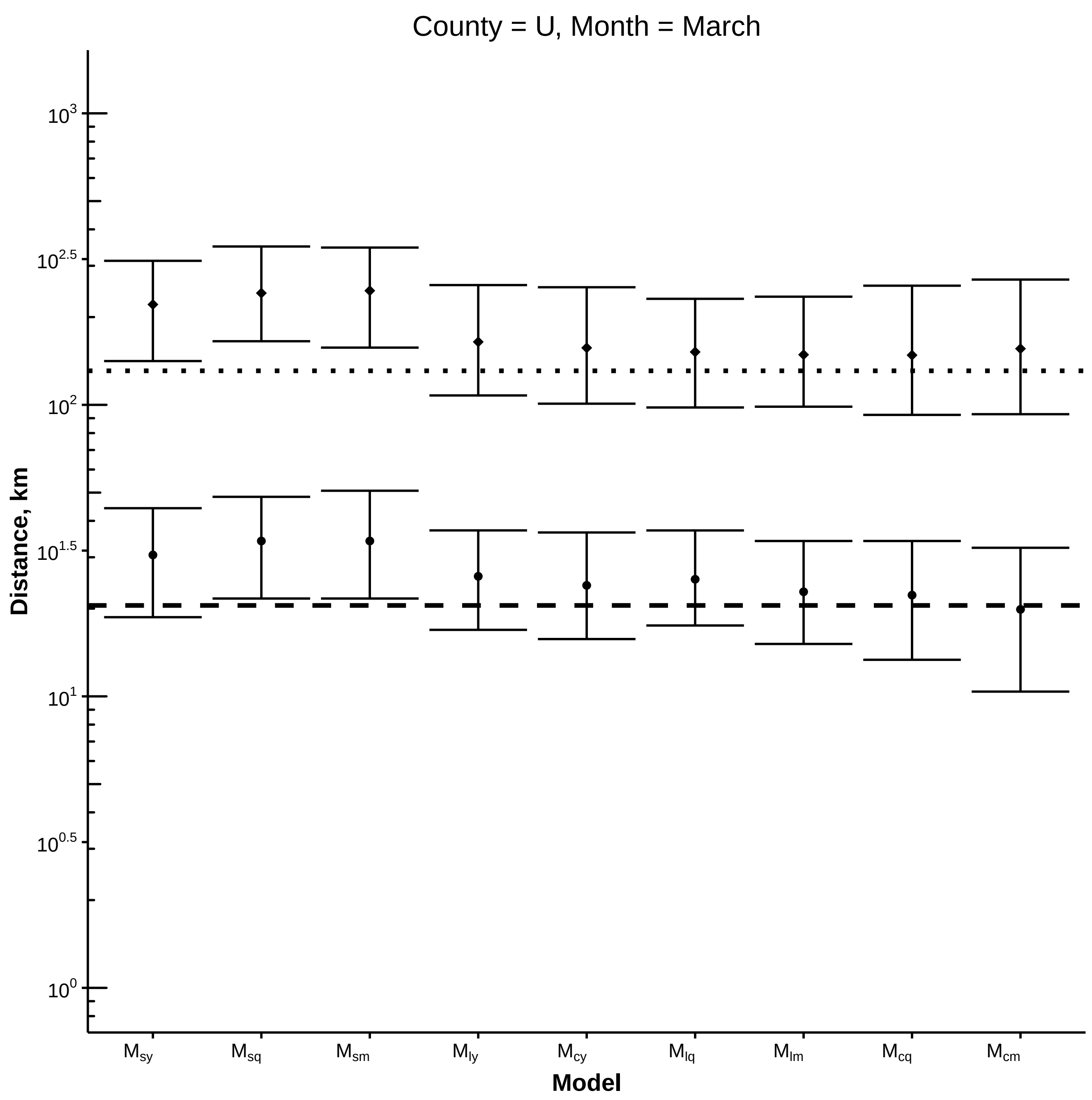

County = U, Month = March
Distance, km
10^3
$10^{2.5}$
10^2
$10^{1.5}$
10^1
$10^{0.5}$
10^0
M_{sy}
M_{sq}
M_{sm}
M_{ly}
M_{cy}
M_{lq}
M_{lm}
M_{cq}
M_{cm}
Model

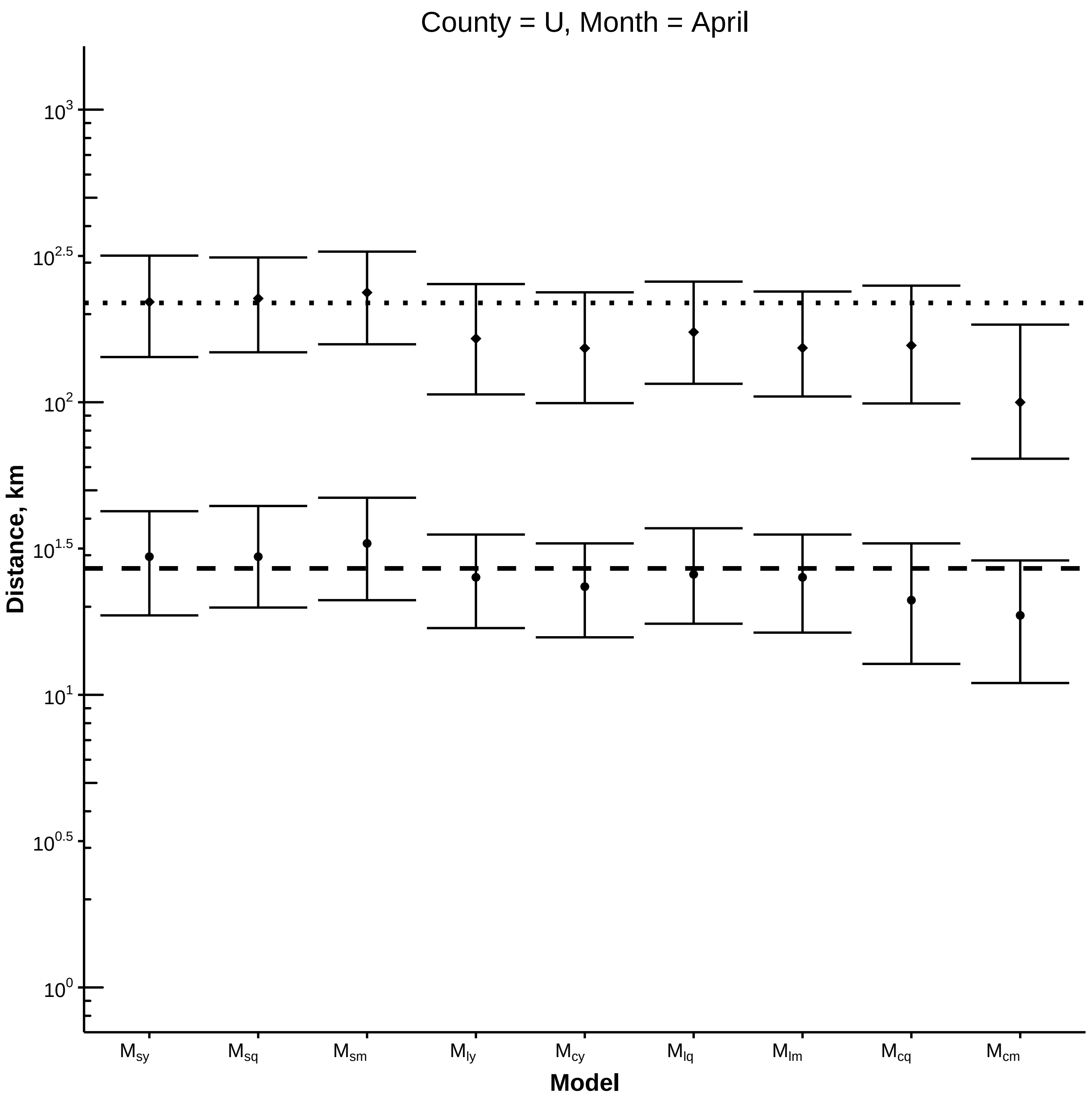

County = U, Month = April
Distance, km
10^3
$10^{2.5}$
10^2
$10^{1.5}$
10^1
$10^{0.5}$
10^0
M_{sy}
M_{sq}
M_{sm}
M_{ly}
M_{cy}
M_{lq}
M_{lm}
M_{cq}
M_{cm}
Model

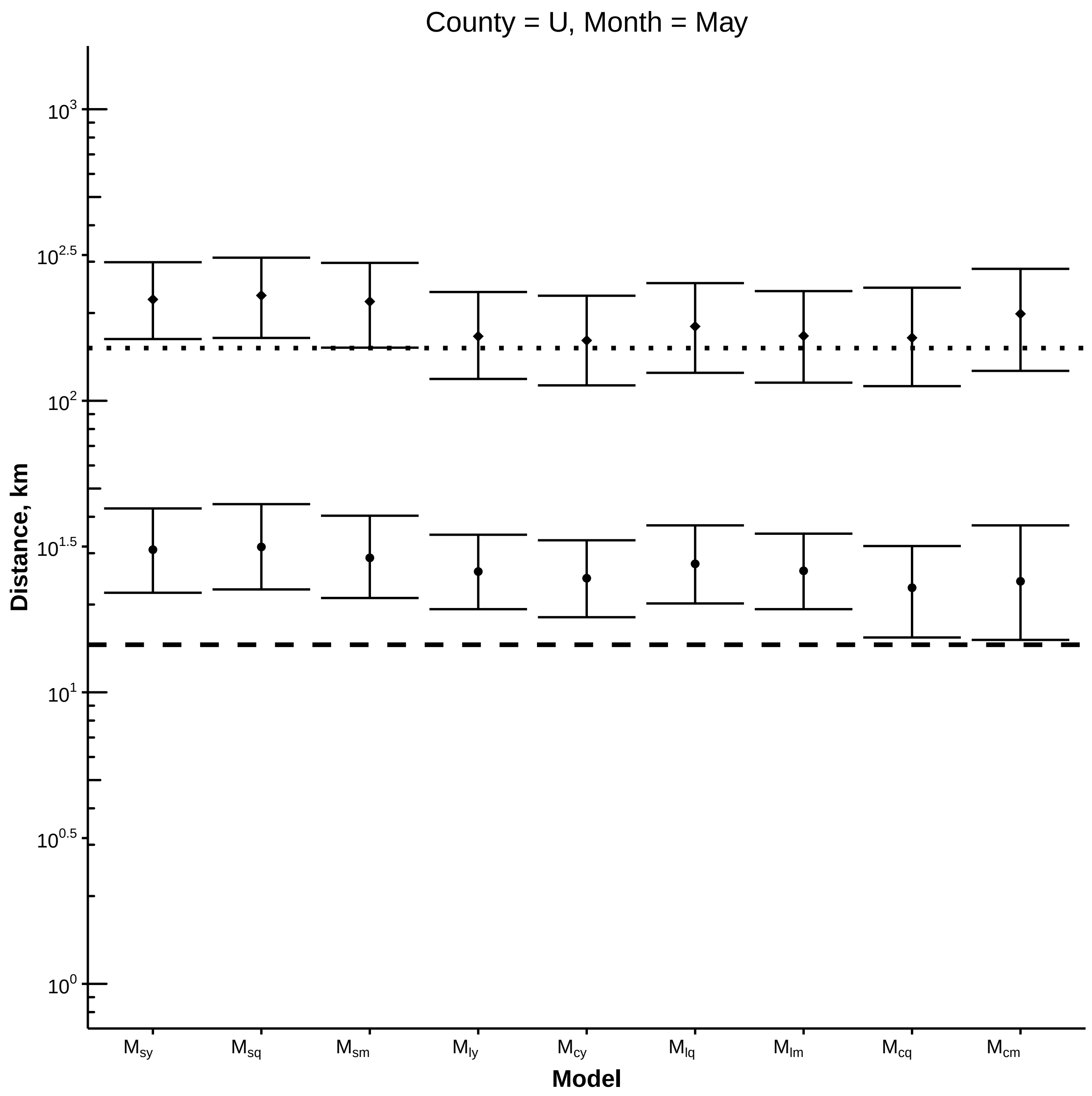

County = U, Month = May
Distance, km
10^3
$10^{2.5}$
10^2
$10^{1.5}$
10^1
$10^{0.5}$
10^0
M_{sy}
M_{sq}
M_{sm}
M_{ly}
M_{cy}
M_{lq}
M_{lm}
M_{cq}
M_{cm}
Model

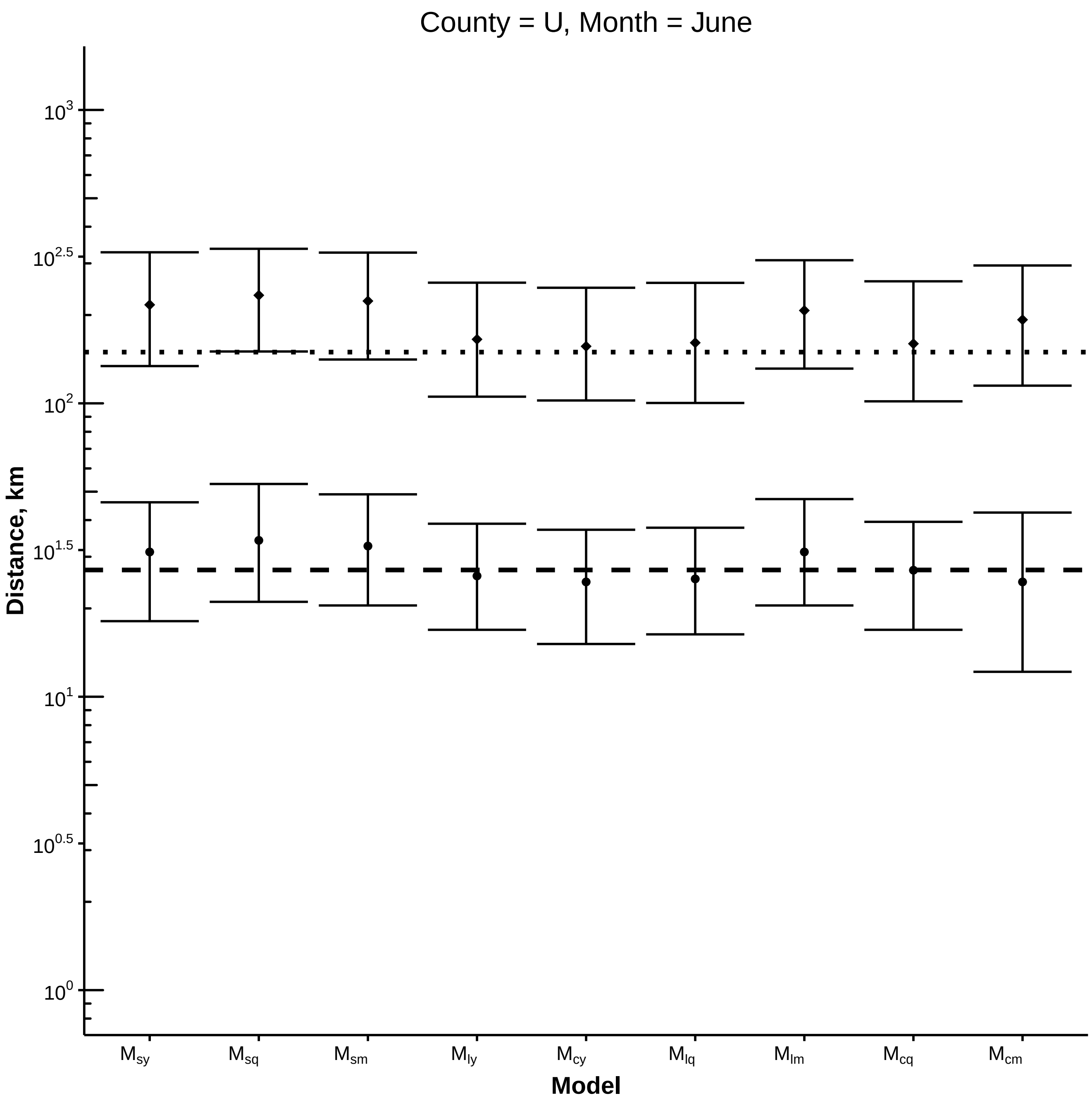
County = U, Month = June
Distance, km
10^3
$10^{2.5}$
10^2
$10^{1.5}$
10^1
$10^{0.5}$
10^0
M_{sy}
M_{sq}
M_{sm}
M_{ly}
M_{cy}
M_{lq}
M_{lm}
M_{cq}
M_{cm}
Model

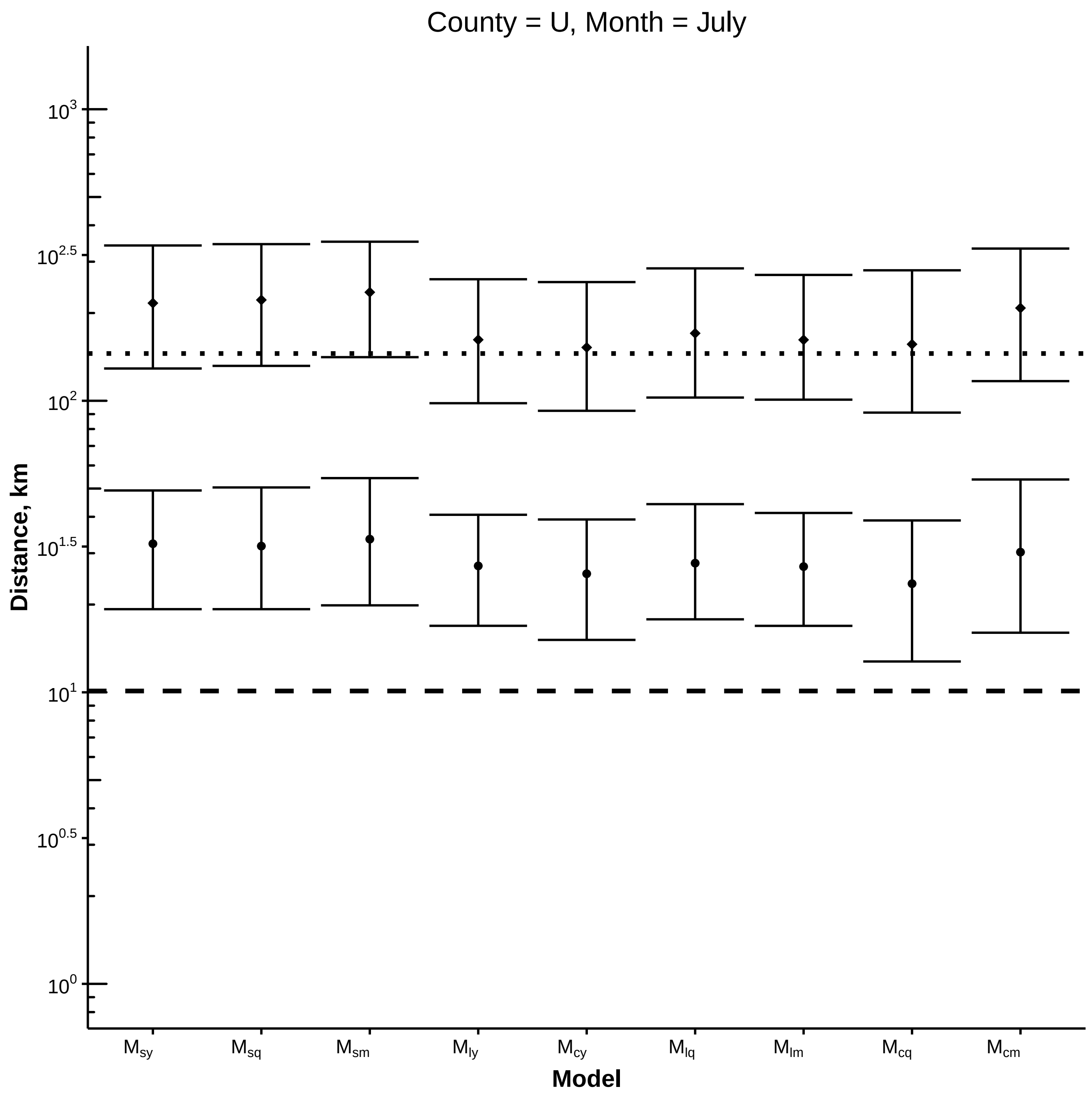

County = U, Month = July
Distance, km
10^3
$10^{2.5}$
10^2
$10^{1.5}$
10^1
$10^{0.5}$
10^0
M_{sy}
M_{sq}
M_{sm}
M_{ly}
M_{cy}
M_{lq}
M_{lm}
M_{cq}
M_{cm}
Model

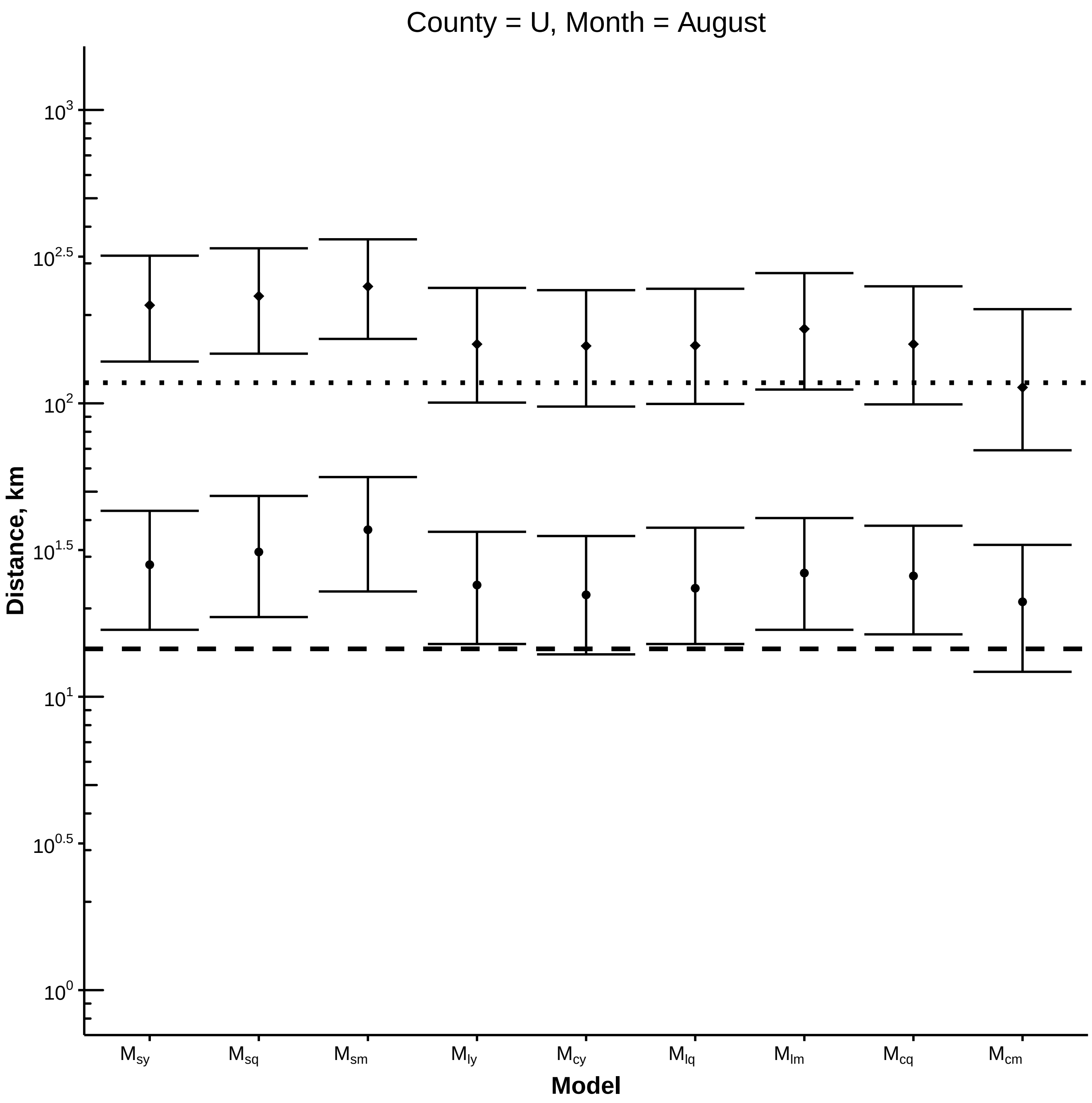
County = U, Month = August
Distance, km
10^3
$10^{2.5}$
10^2
$10^{1.5}$
10^1
$10^{0.5}$
10^0
M_{sy}
M_{sq}
M_{sm}
M_{ly}
M_{cy}
M_{lq}
M_{lm}
M_{cq}
M_{cm}
Model

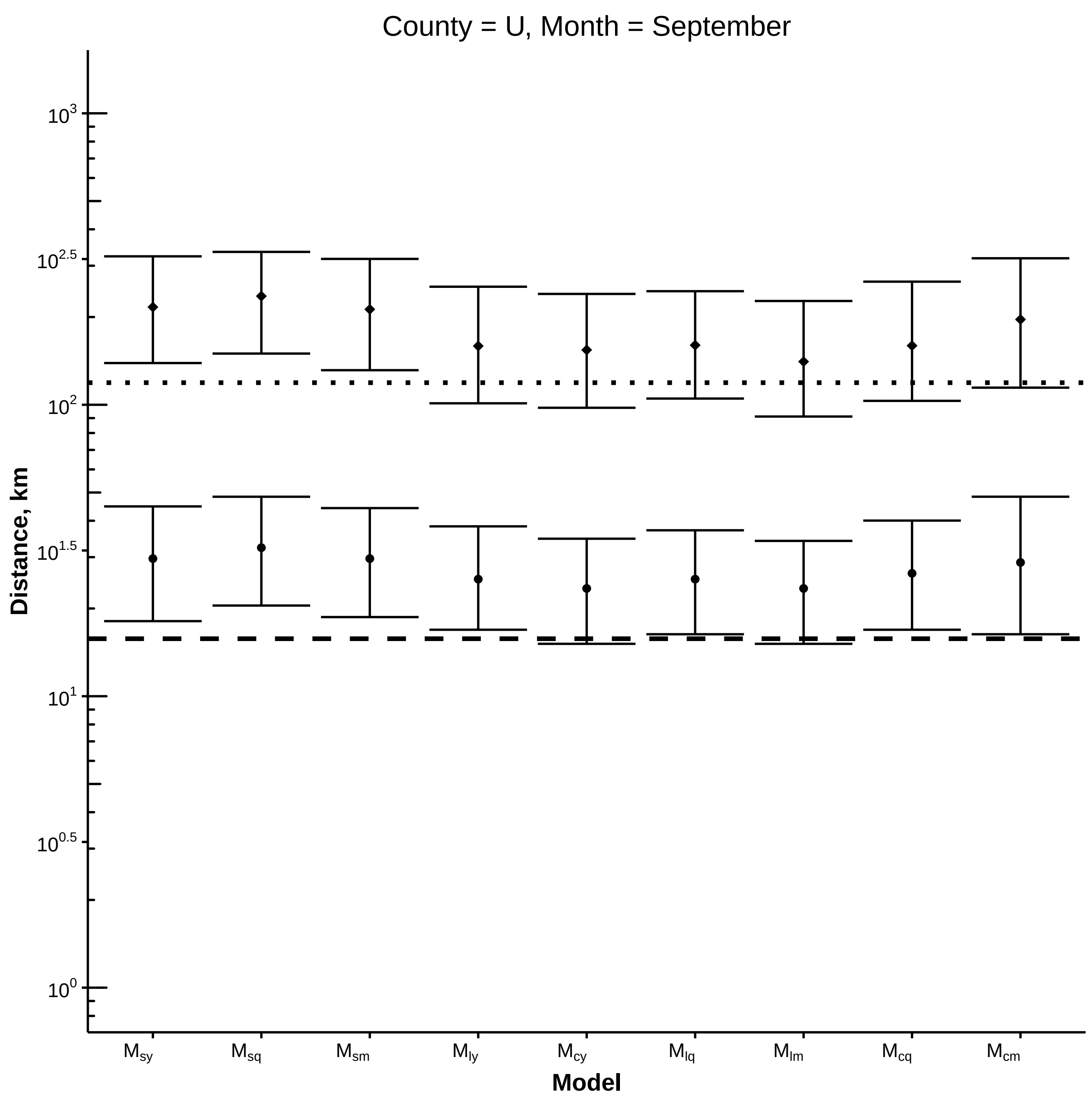

County = U, Month = September
Distance, km
10^3
$10^{2.5}$
10^2
$10^{1.5}$
10^1
$10^{0.5}$
10^0
M_{sy}
M_{sq}
M_{sm}
M_{ly}
M_{cy}
M_{lq}
M_{lm}
M_{cq}
M_{cm}
Model

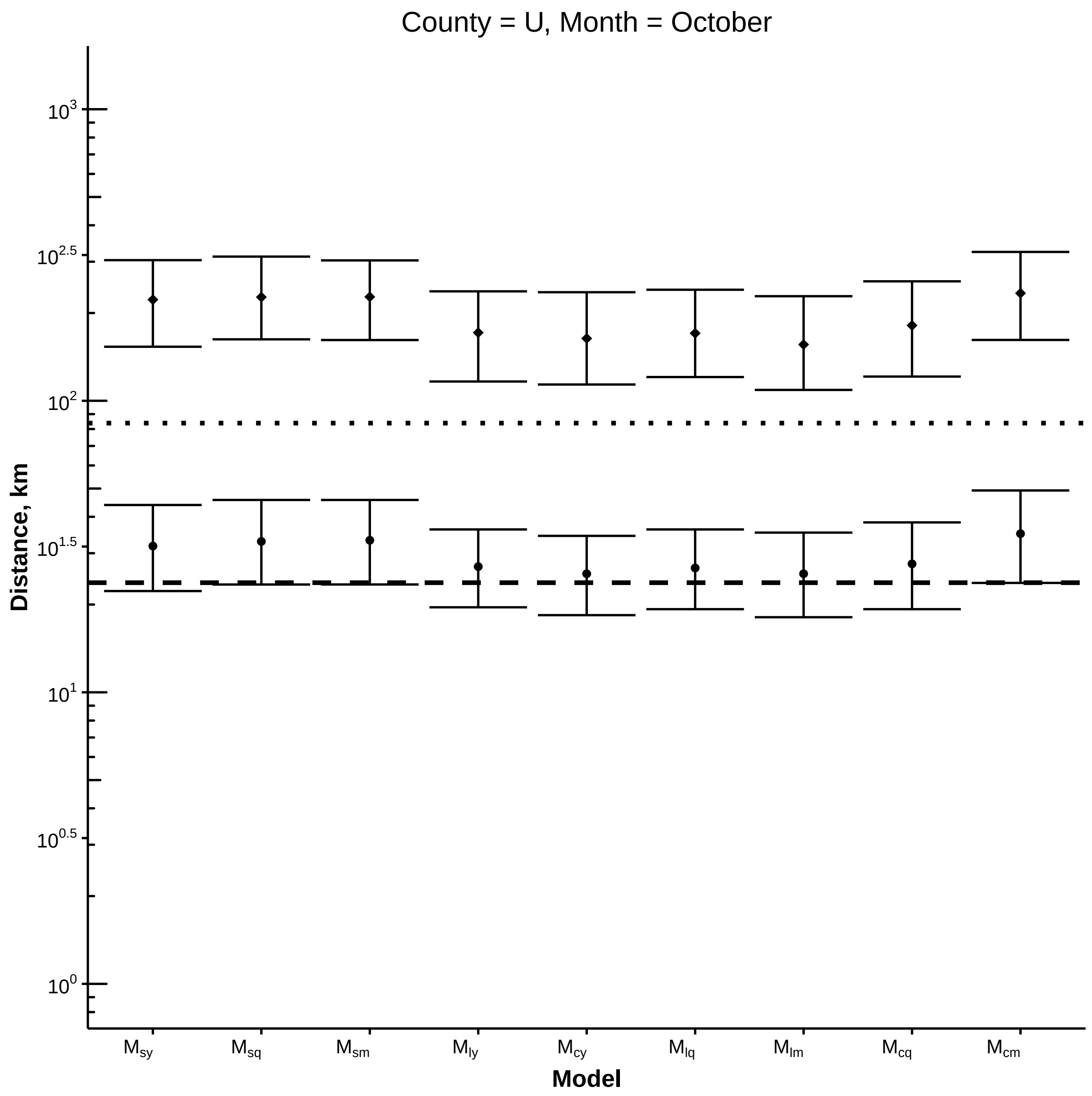
County = U, Month = October
Distance, km
10^3
$10^{2.5}$
10^2
$10^{1.5}$
10^1
$10^{0.5}$
10^0
M_{sy}
M_{sq}
M_{sm}
M_{ly}
M_{cy}
M_{lq}
M_{lm}
M_{cq}
M_{cm}
Model

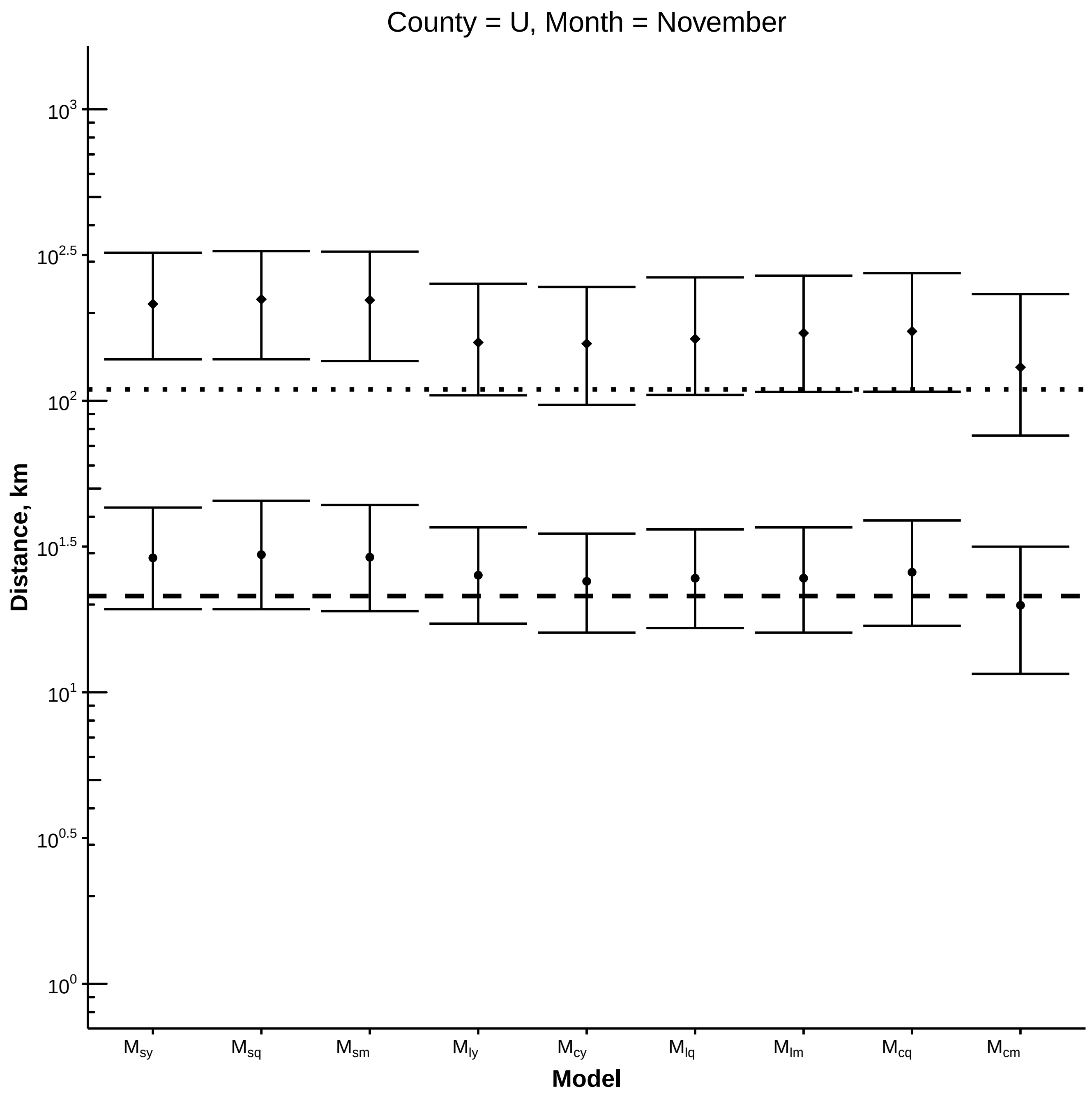

County = U, Month = November
Distance, km
10^3
$10^{2.5}$
10^2
$10^{1.5}$
10^1
$10^{0.5}$
10^0
M_{sy}
M_{sq}
M_{sm}
M_{ly}
M_{cy}
M_{lq}
M_{lm}
M_{cq}
M_{cm}
Model

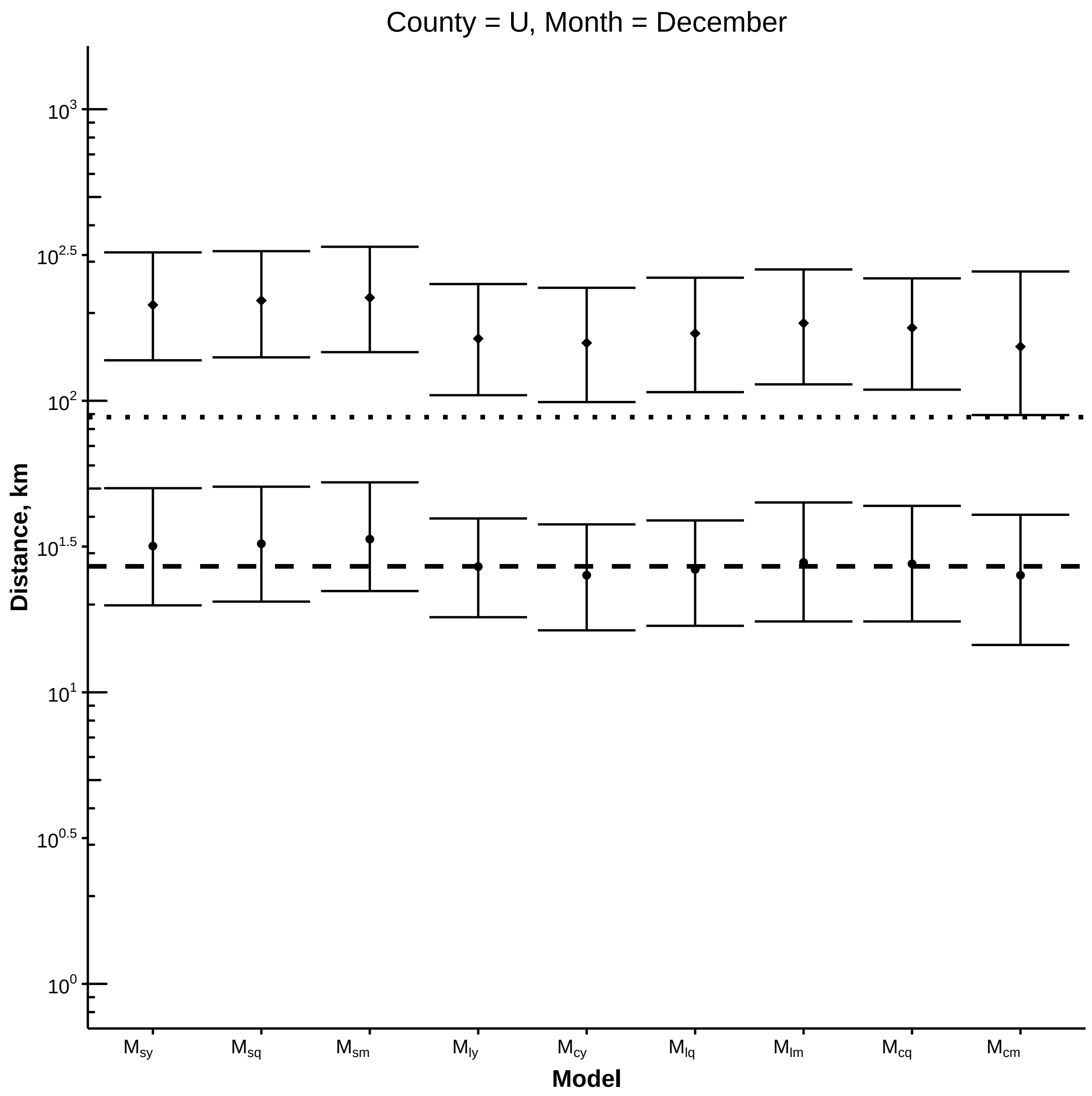

County = U, Month = December
Distance, km
10^3
10^2.5
10^2
10^1.5
10^1
10^0.5
10^0
Msy
Msq
Msm
Mly
Mcy
Mlq
Mlm
Mcq
Mcm
Model

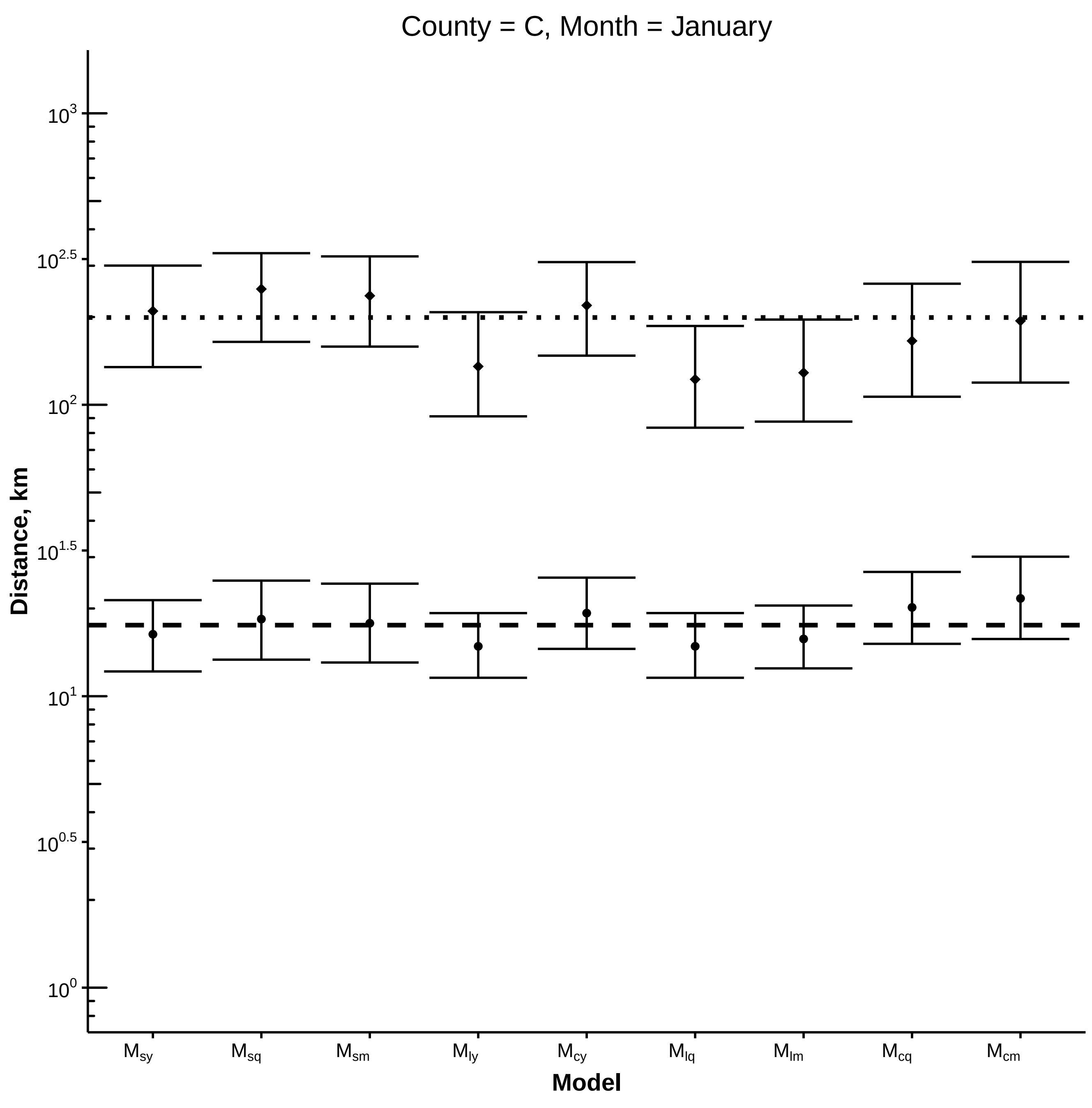

County = C, Month = January
Distance, km
10^3
$10^{2.5}$
10^2
$10^{1.5}$
10^1
$10^{0.5}$
10^0
M_{sy}
M_{sq}
M_{sm}
M_{ly}
M_{cy}
M_{lq}
M_{lm}
M_{cq}
M_{cm}
Model

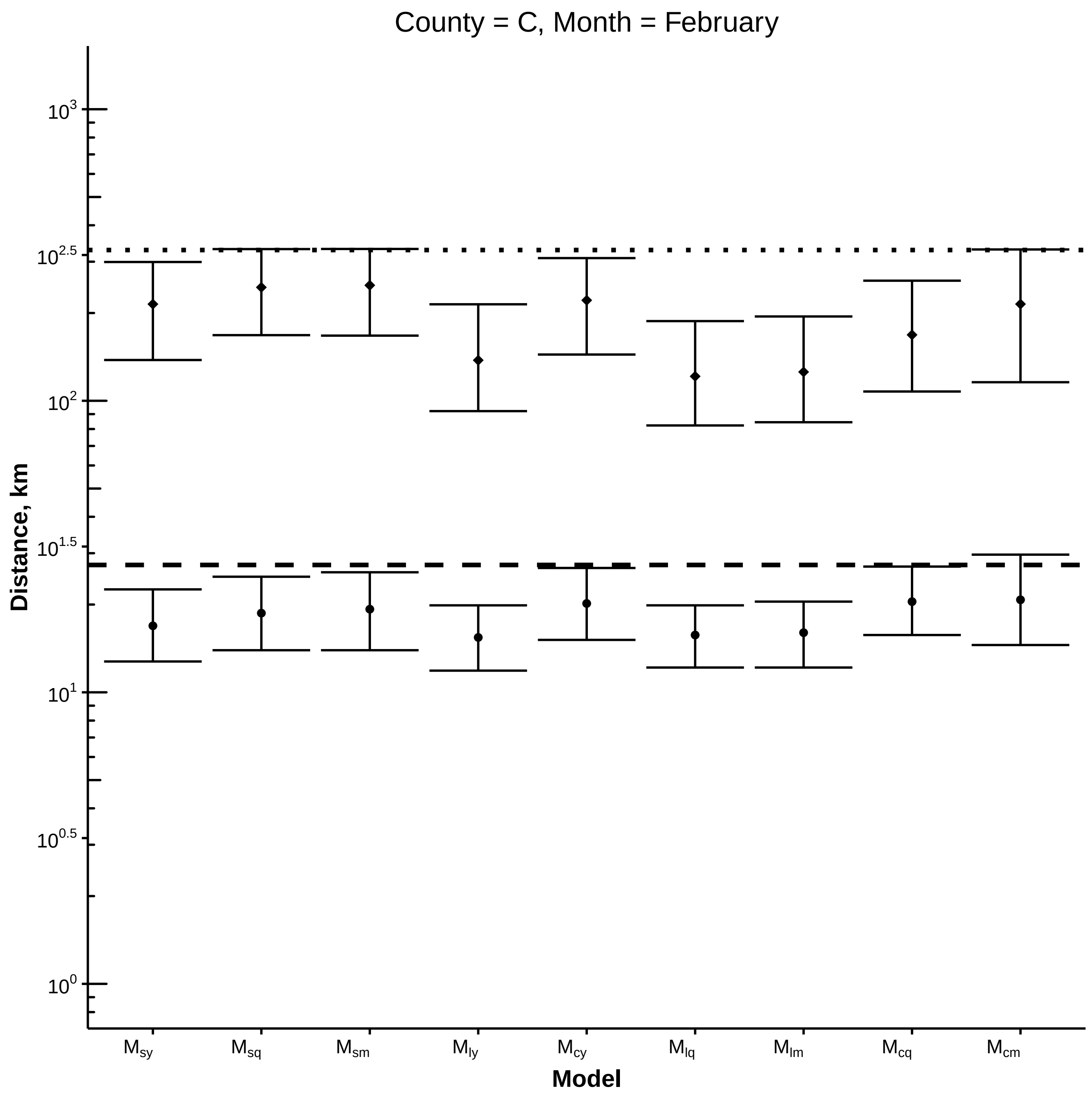

County = C, Month = February
Distance, km
10^3
$10^{2.5}$
10^2
$10^{1.5}$
10^1
$10^{0.5}$
10^0
M_{sy}
M_{sq}
M_{sm}
M_{ly}
M_{cy}
M_{lq}
M_{lm}
M_{cq}
M_{cm}
Model

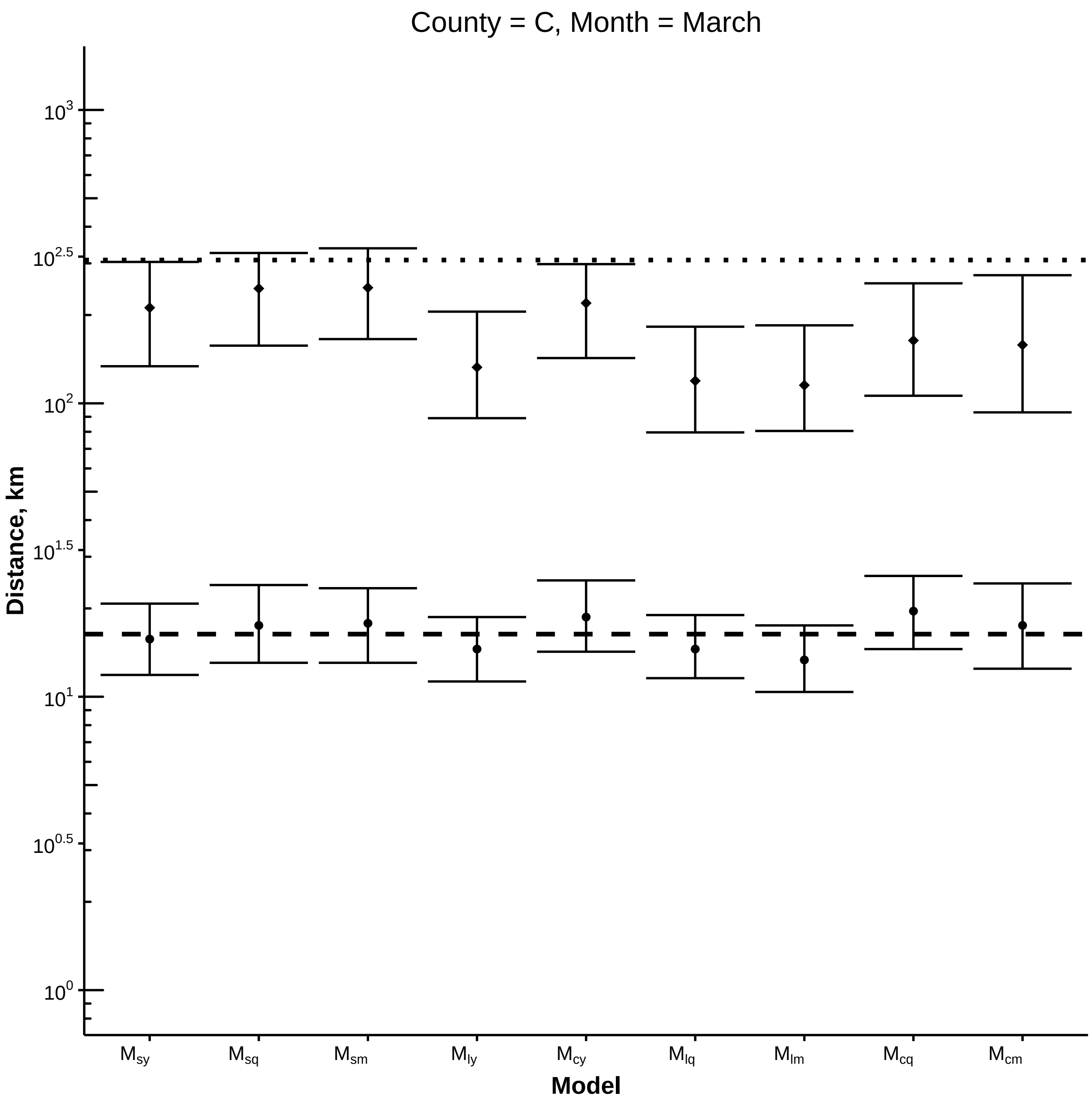

County = C, Month = March
Distance, km
10^3
$10^{2.5}$
10^2
$10^{1.5}$
10^1
$10^{0.5}$
10^0
M_{sy}
M_{sq}
M_{sm}
M_{ly}
M_{cy}
M_{lq}
M_{lm}
M_{cq}
M_{cm}
Model

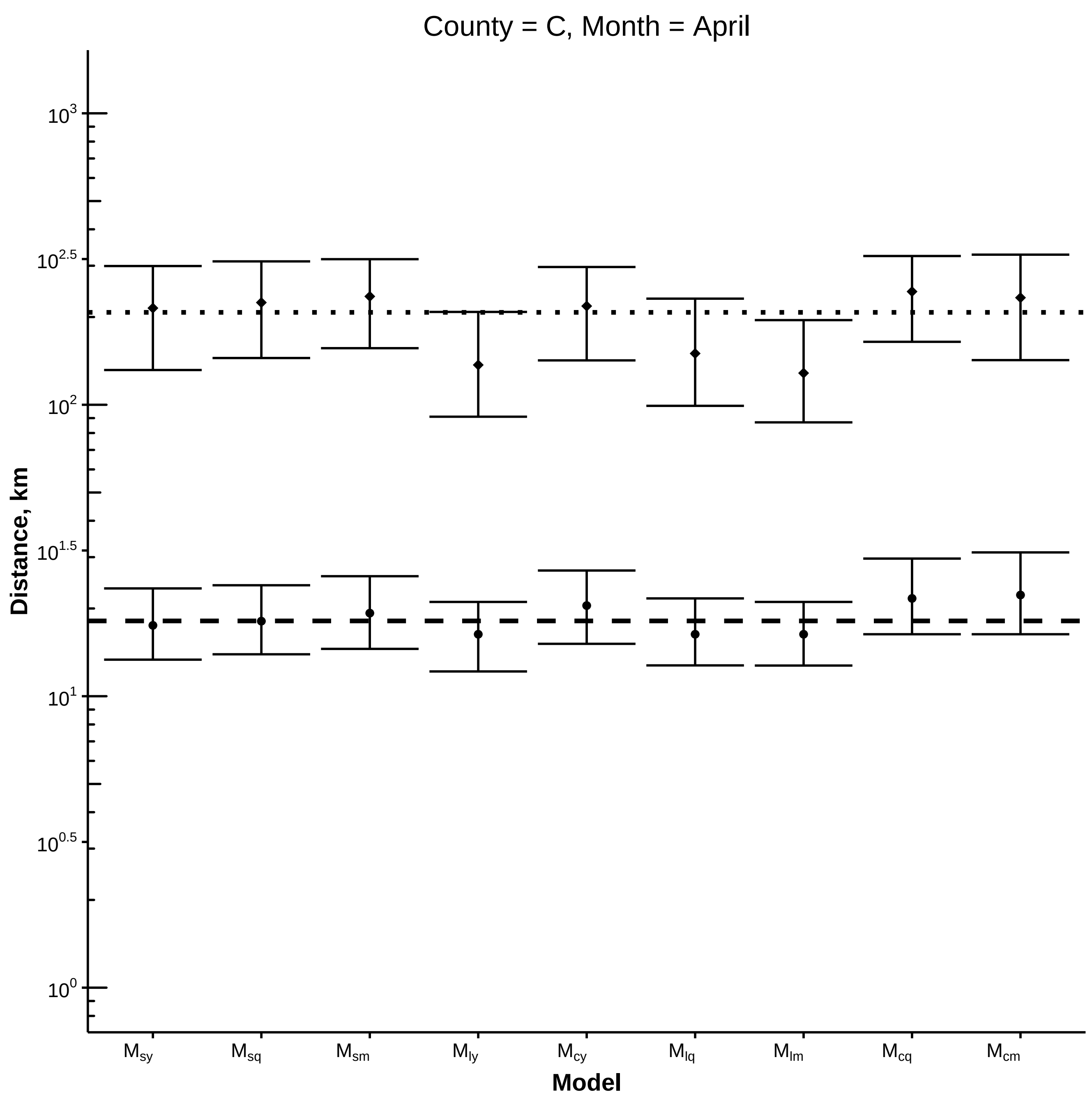

County = C, Month = April
Distance, km
10^3
$10^{2.5}$
10^2
$10^{1.5}$
10^1
$10^{0.5}$
10^0
M_{sy}
M_{sq}
M_{sm}
M_{ly}
M_{cy}
M_{lq}
M_{lm}
M_{cq}
M_{cm}
Model

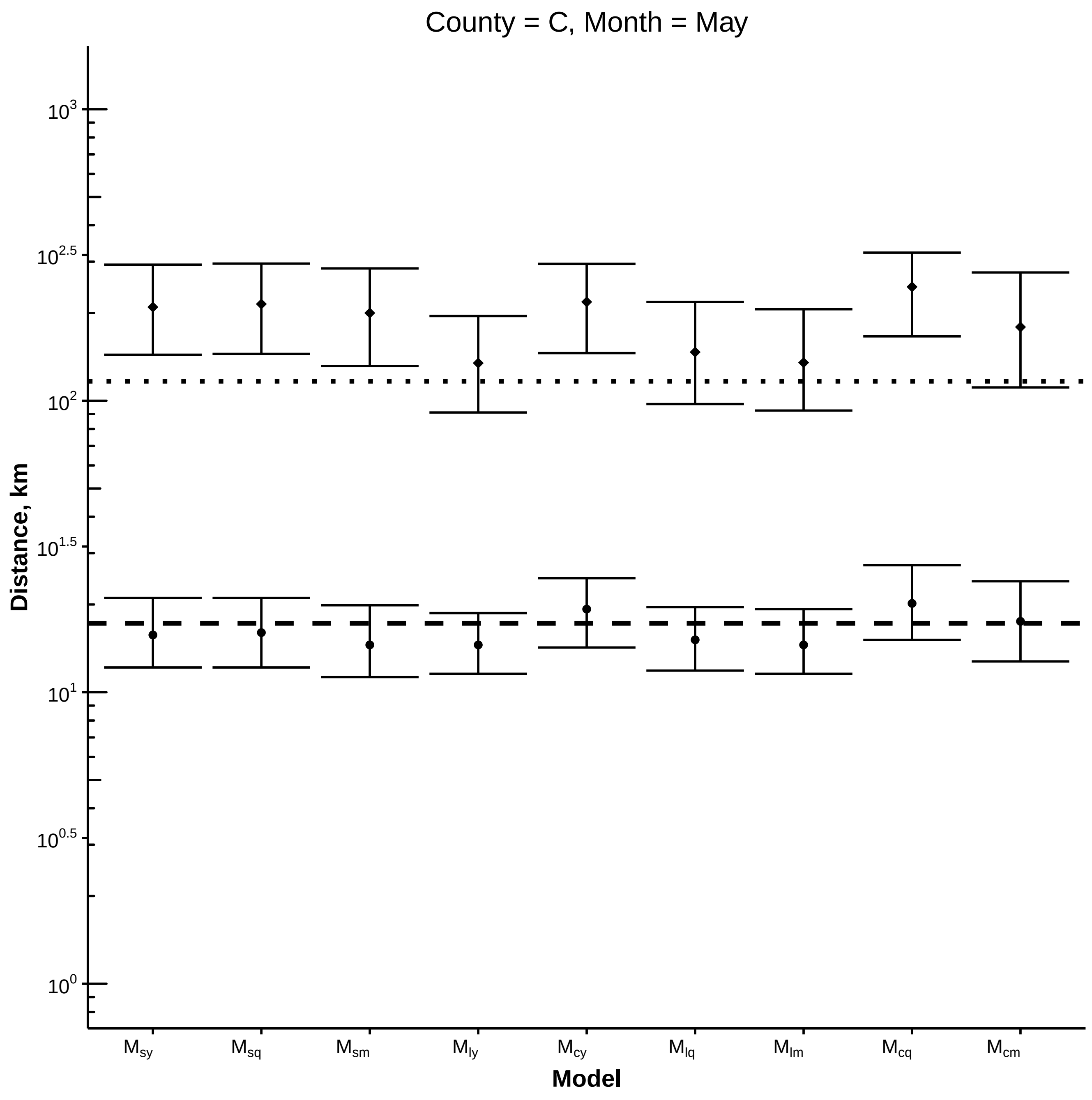
County = C, Month = May
Distance, km
10^3
$10^{2.5}$
10^2
$10^{1.5}$
10^1
$10^{0.5}$
10^0
M_{sy}
M_{sq}
M_{sm}
M_{ly}
M_{cy}
M_{lq}
M_{lm}
M_{cq}
M_{cm}
Model

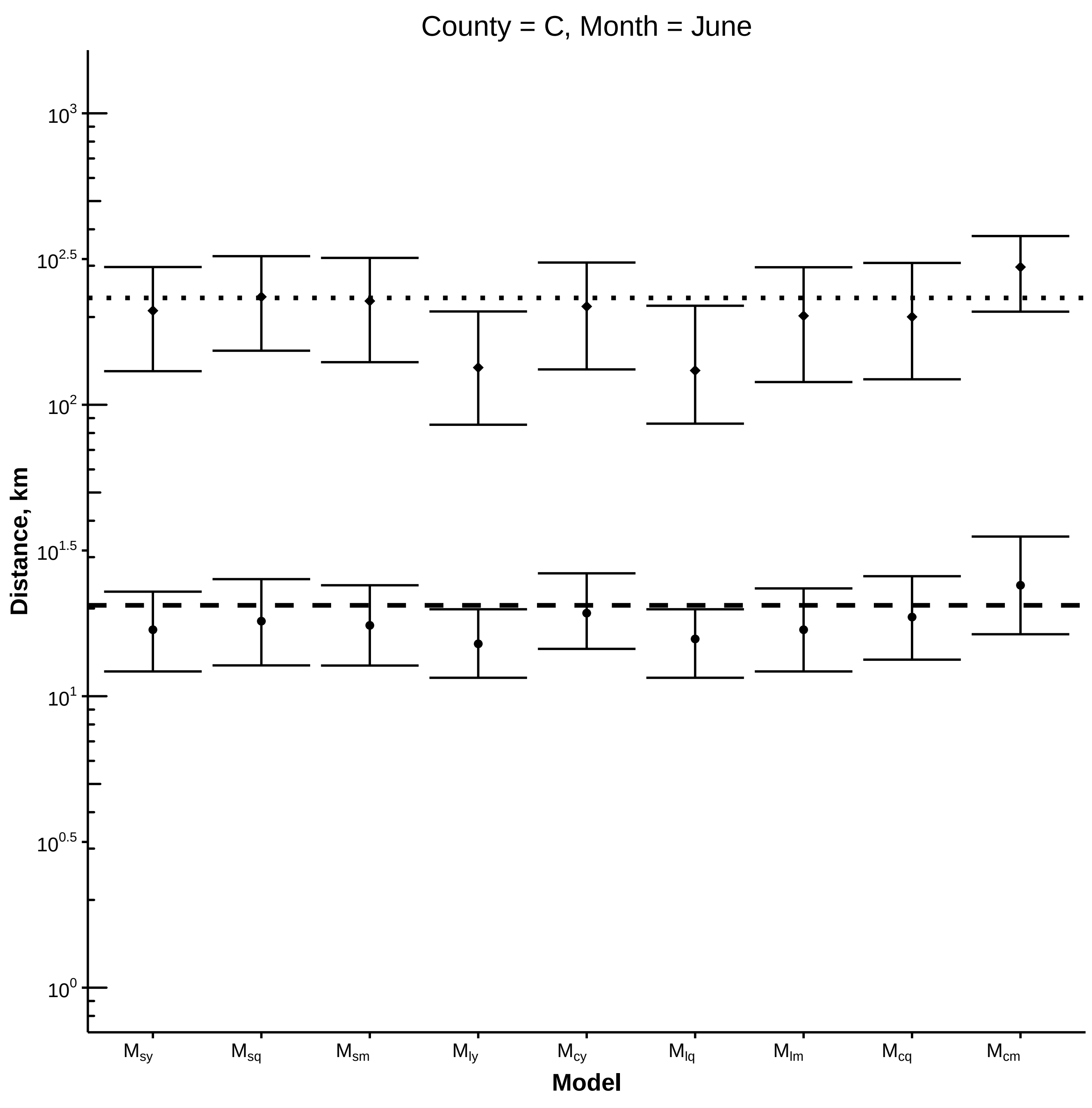

County = C, Month = June
Distance, km
10^3
$10^{2.5}$
10^2
$10^{1.5}$
10^1
$10^{0.5}$
10^0
M_{sy}
M_{sq}
M_{sm}
M_{ly}
M_{cy}
M_{lq}
M_{lm}
M_{cq}
M_{cm}
Model

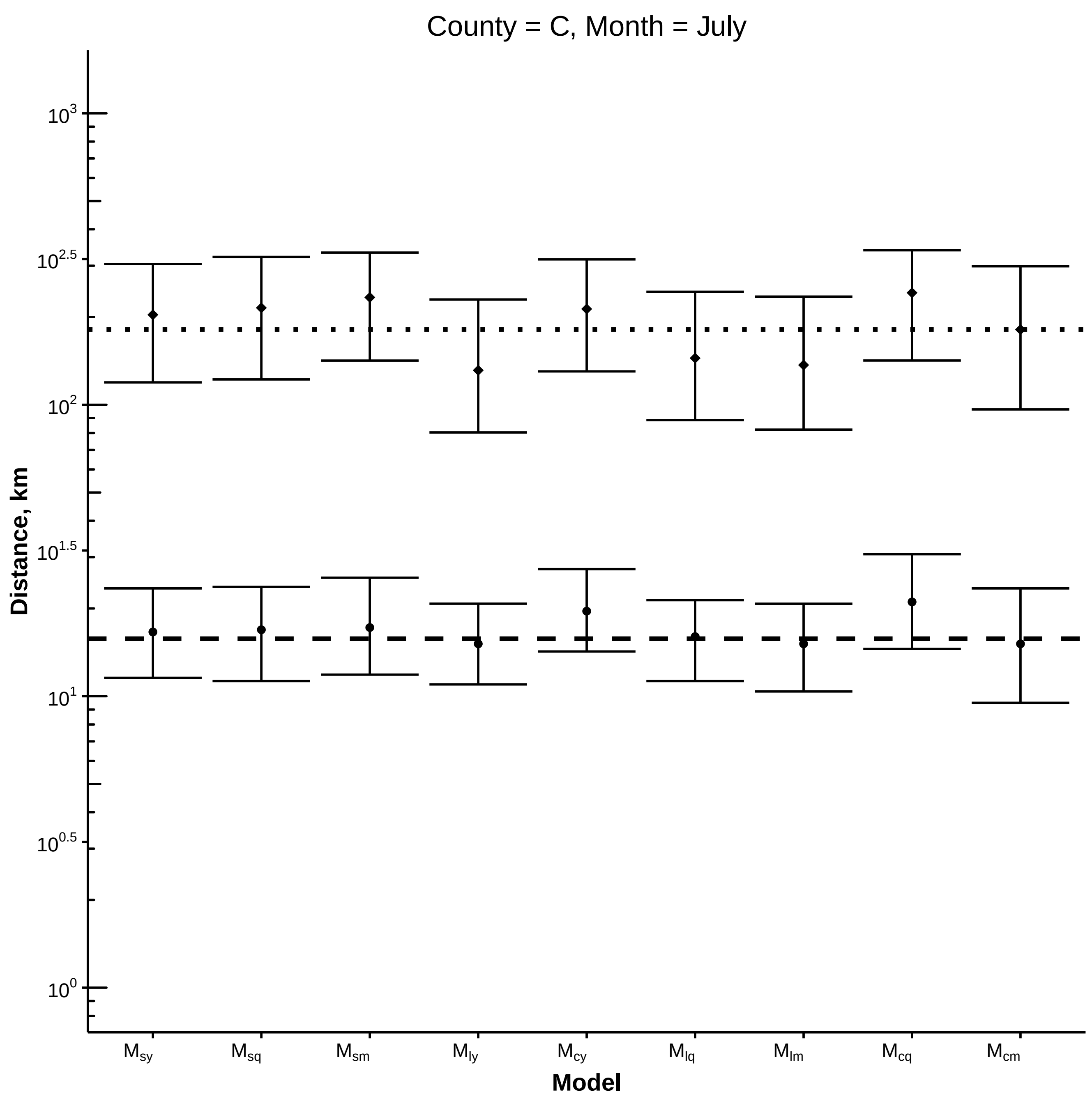
County = C, Month = July
Distance, km
10^3
10^2.5
10^2
10^1.5
10^1
10^0.5
10^0
Msy
Msq
Msm
Mly
Mcy
Mlq
Mlm
Mcq
Mcm
Model

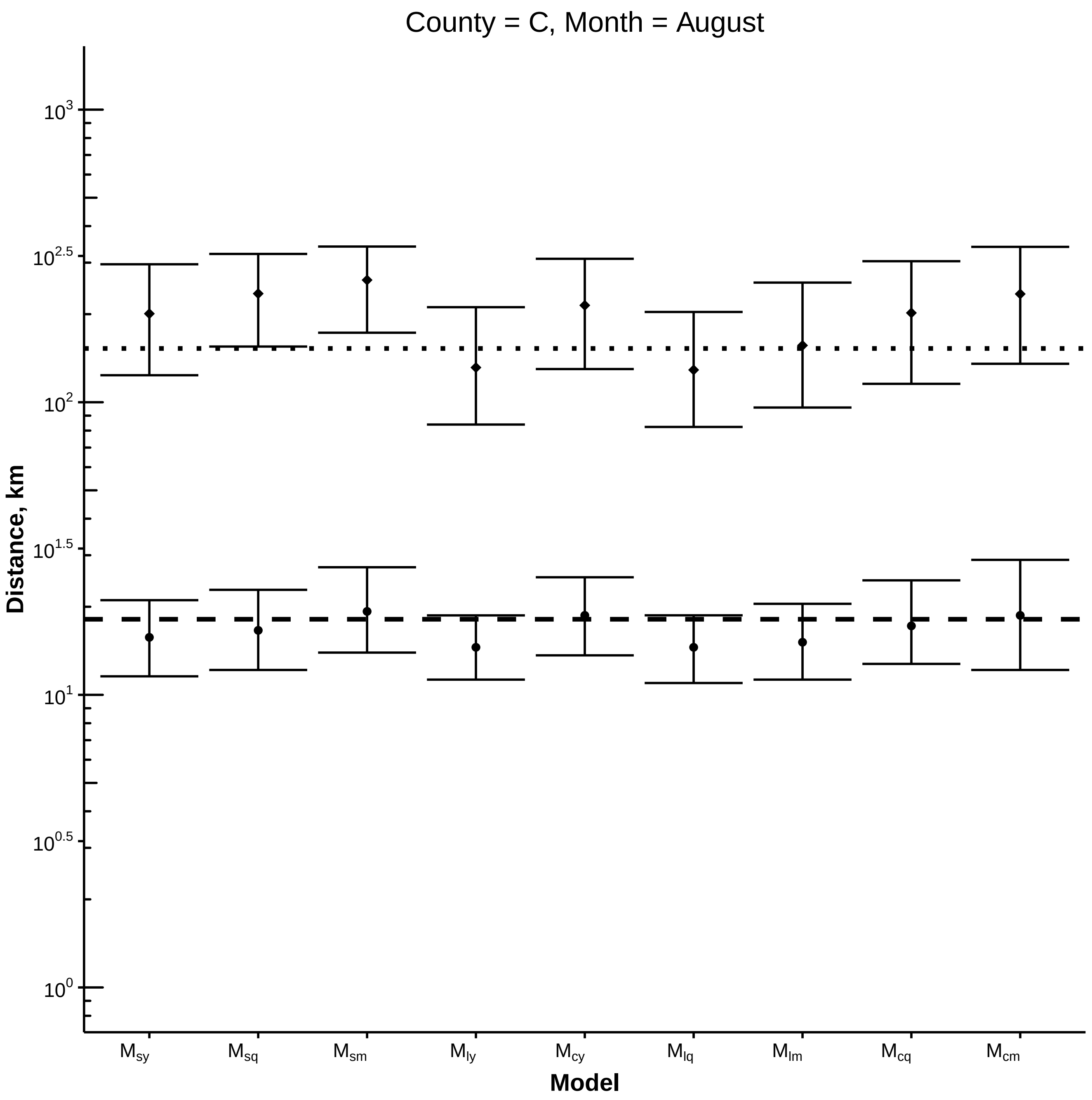
County = C, Month = August
Distance, km
10^3
$10^{2.5}$
10^2
$10^{1.5}$
10^1
$10^{0.5}$
10^0
M_{sy}
M_{sq}
M_{sm}
M_{ly}
M_{cy}
M_{lq}
M_{lm}
M_{cq}
M_{cm}
Model

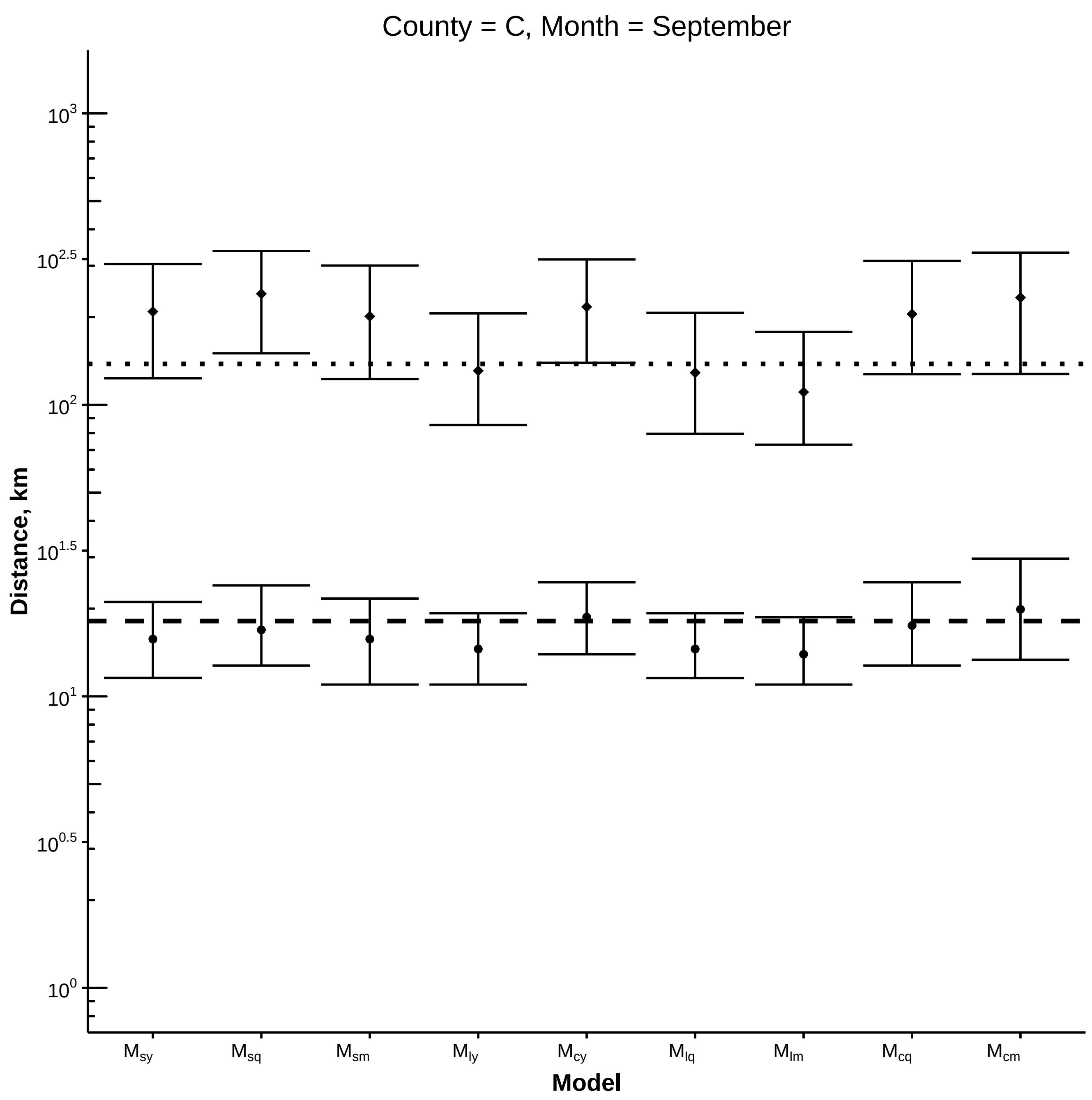

County = C, Month = September
Distance, km
10^3
10^2.5
10^2
10^1.5
10^1
10^0.5
10^0
M_sy
M_sq
M_sm
M_ly
M_cy
M_lq
M_lm
M_cq
M_cm
Model

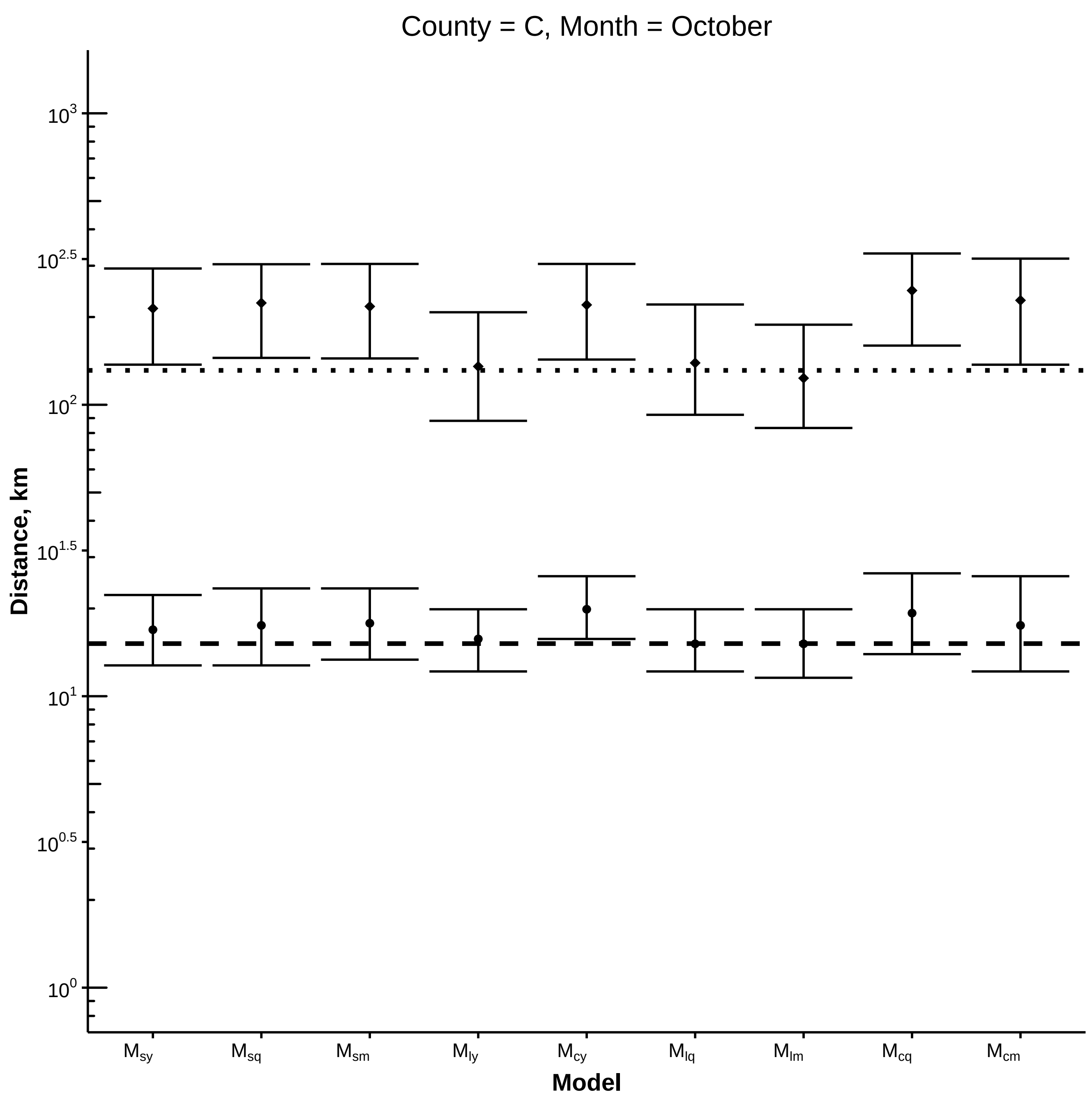

County = C, Month = October
Distance, km
10^3
10^2.5
10^2
10^1.5
10^1
10^0.5
10^0
M_sy
M_sq
M_sm
M_ly
M_cy
M_lq
M_lm
M_cq
M_cm
Model

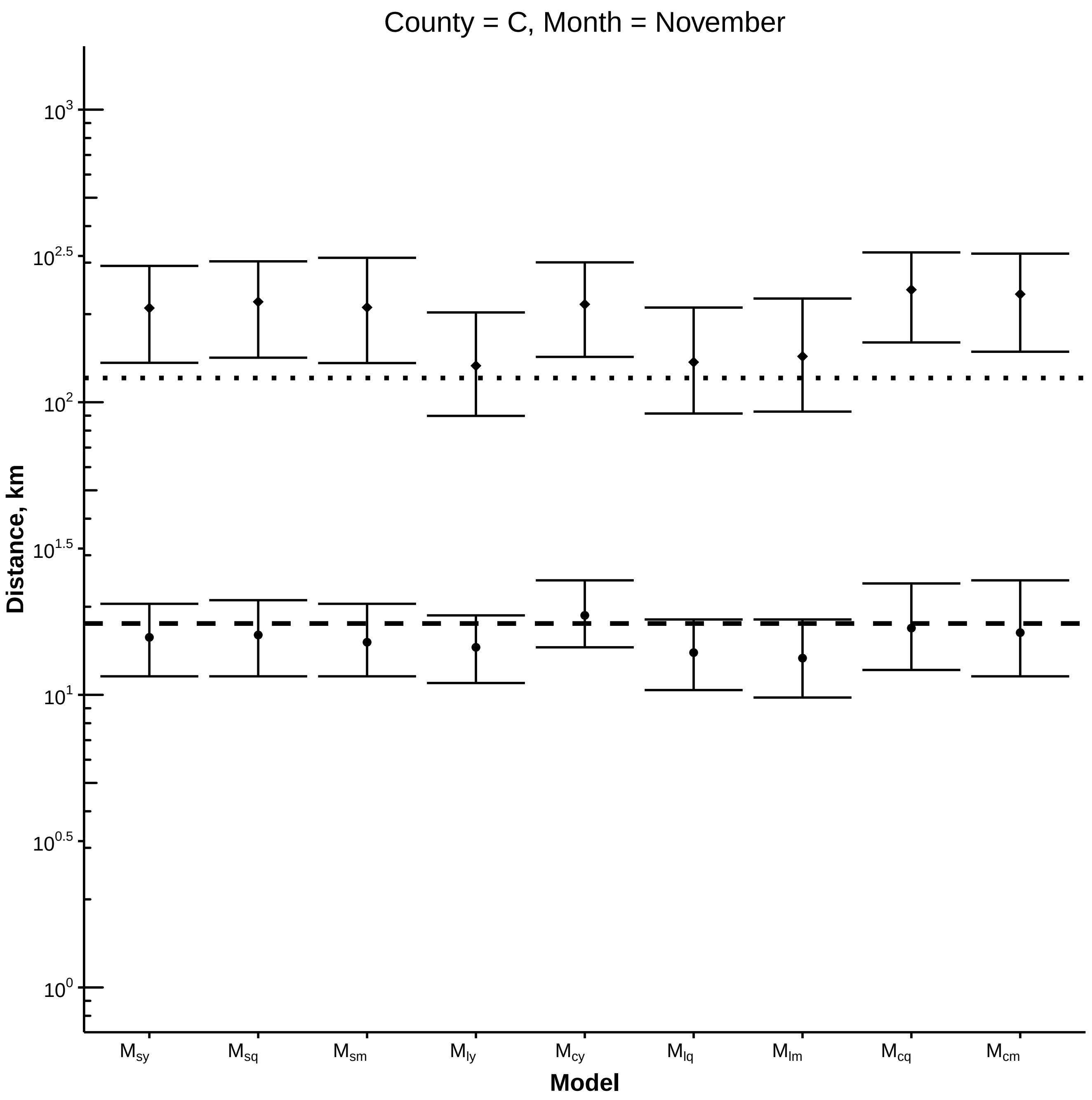

County = C, Month = November
Distance, km
10^{3}
$10^{2.5}$
10^{2}
$10^{1.5}$
10^{1}
$10^{0.5}$
10^{0}
M_{sy}
M_{sq}
M_{sm}
M_{ly}
M_{cy}
M_{lq}
M_{lm}
M_{cq}
M_{cm}
Model

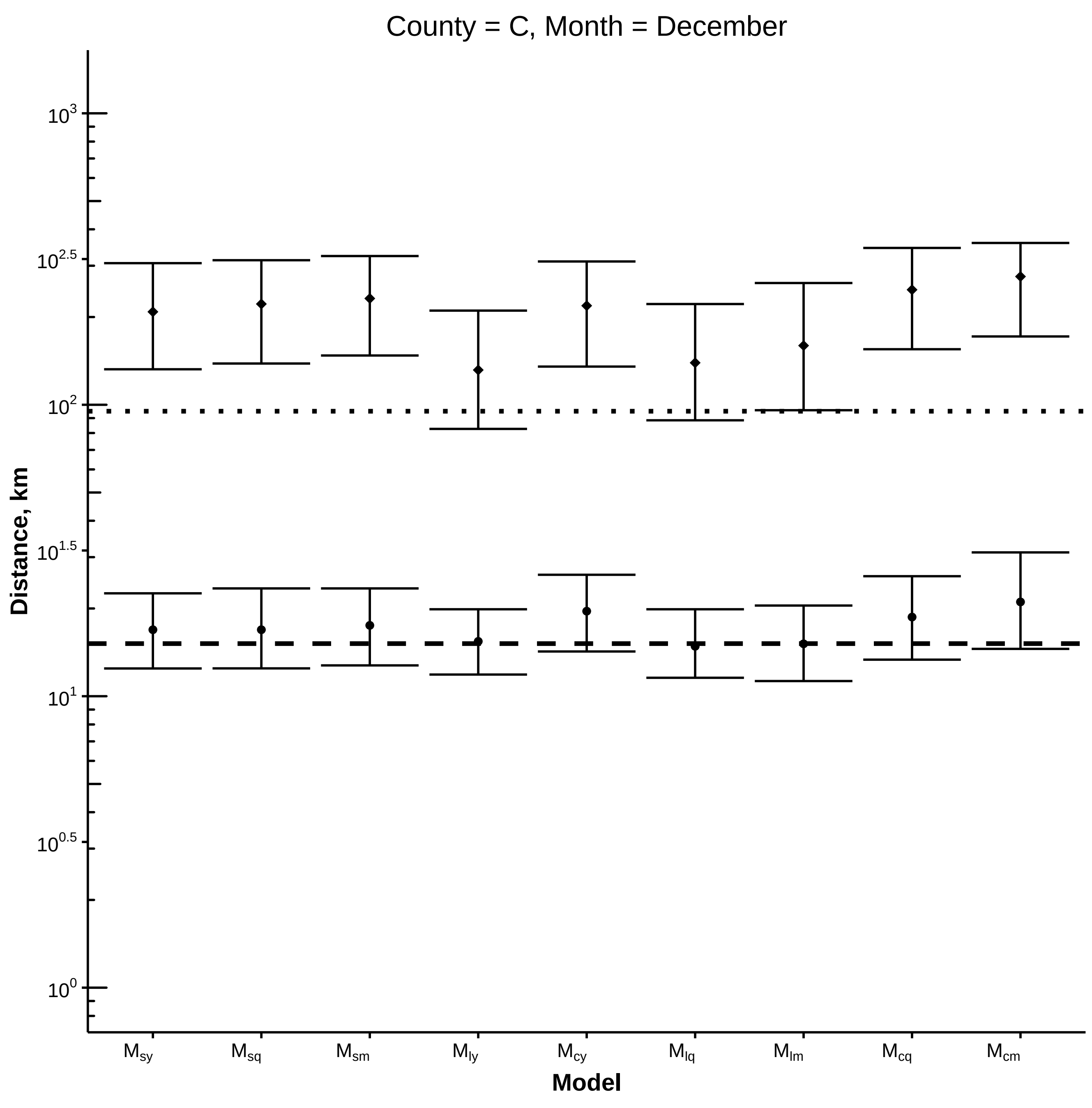

County = C, Month = December
Distance, km
10^{3}
$10^{2.5}$
10^{2}
$10^{1.5}$
10^{1}
$10^{0.5}$
10^{0}
M_{sy}
M_{sq}
M_{sm}
M_{ly}
M_{cy}
M_{lq}
M_{lm}
M_{cq}
M_{cm}
Model

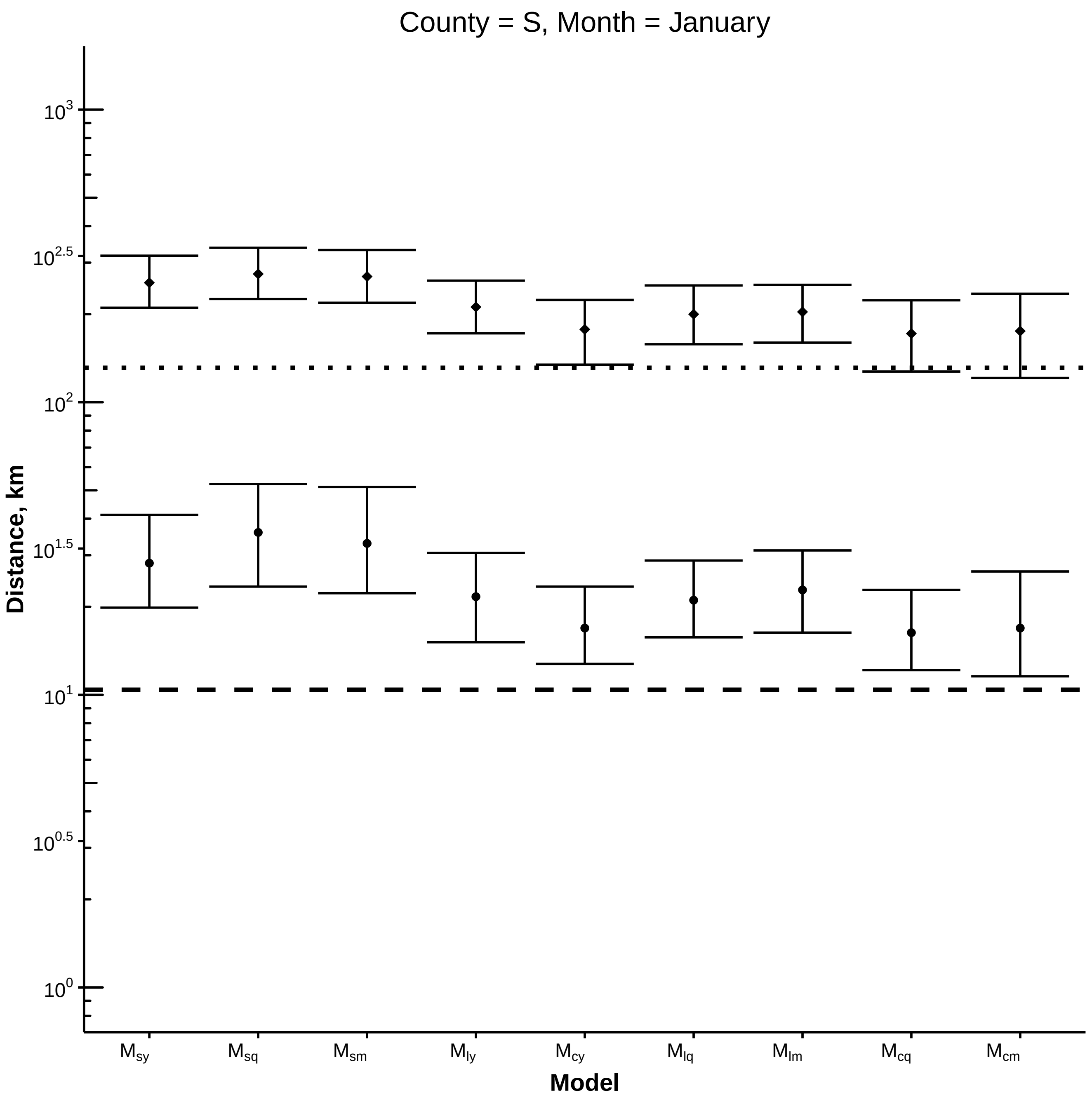
County = S, Month = January
Distance, km
10^3
$10^{2.5}$
10^2
$10^{1.5}$
10^1
$10^{0.5}$
10^0
M_{sy}
M_{sq}
M_{sm}
M_{ly}
M_{cy}
M_{lq}
M_{lm}
M_{cq}
M_{cm}
Model

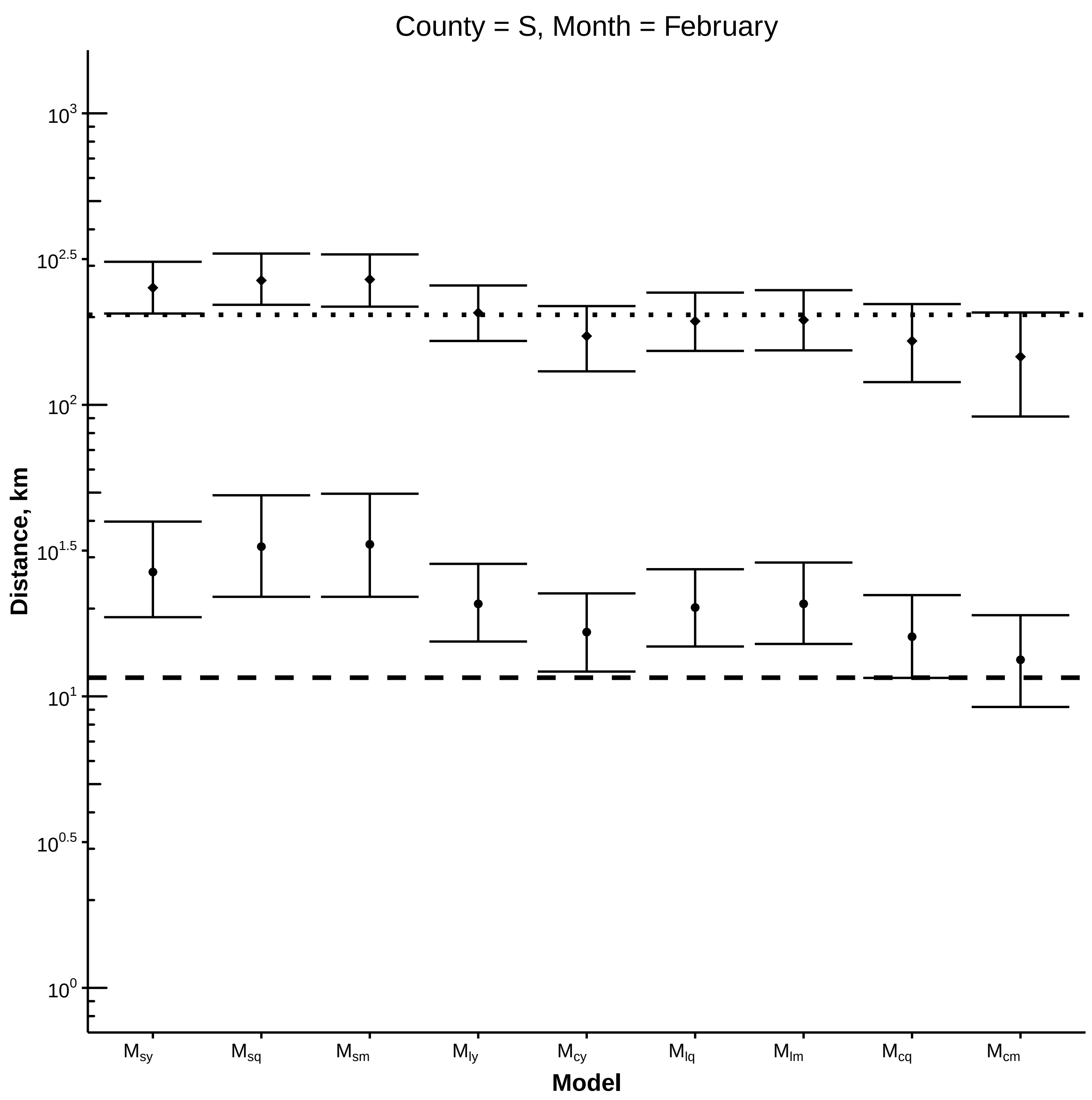

County = S, Month = February
Distance, km
10^3
$10^{2.5}$
10^2
$10^{1.5}$
10^1
$10^{0.5}$
10^0
M_{sy}
M_{sq}
M_{sm}
M_{ly}
M_{cy}
M_{lq}
M_{lm}
M_{cq}
M_{cm}
Model

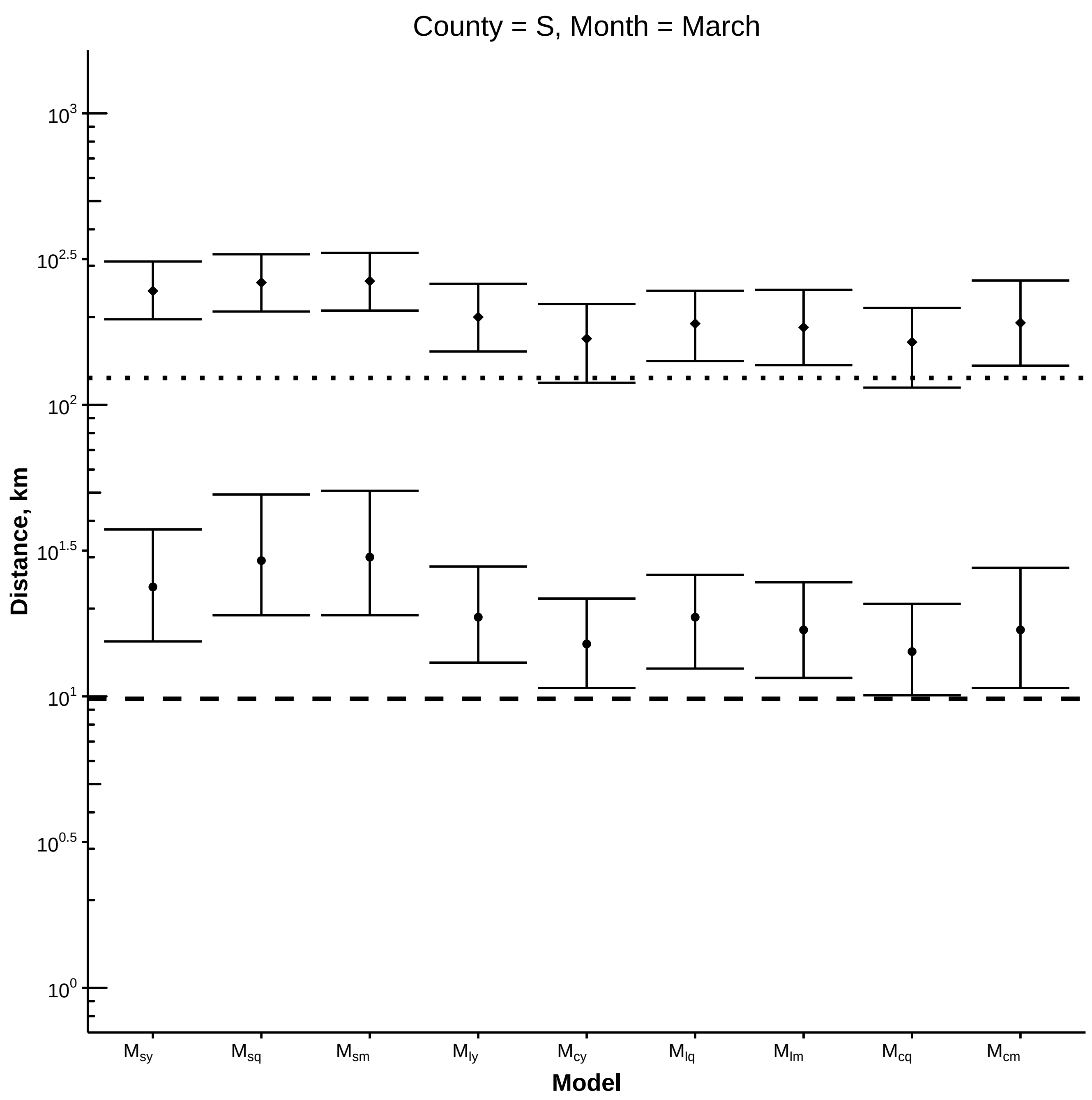
County = S, Month = March
Distance, km
10^3
$10^{2.5}$
10^2
$10^{1.5}$
10^1
$10^{0.5}$
10^0
M_{sy}
M_{sq}
M_{sm}
M_{ly}
M_{cy}
M_{lq}
M_{lm}
M_{cq}
M_{cm}
Model

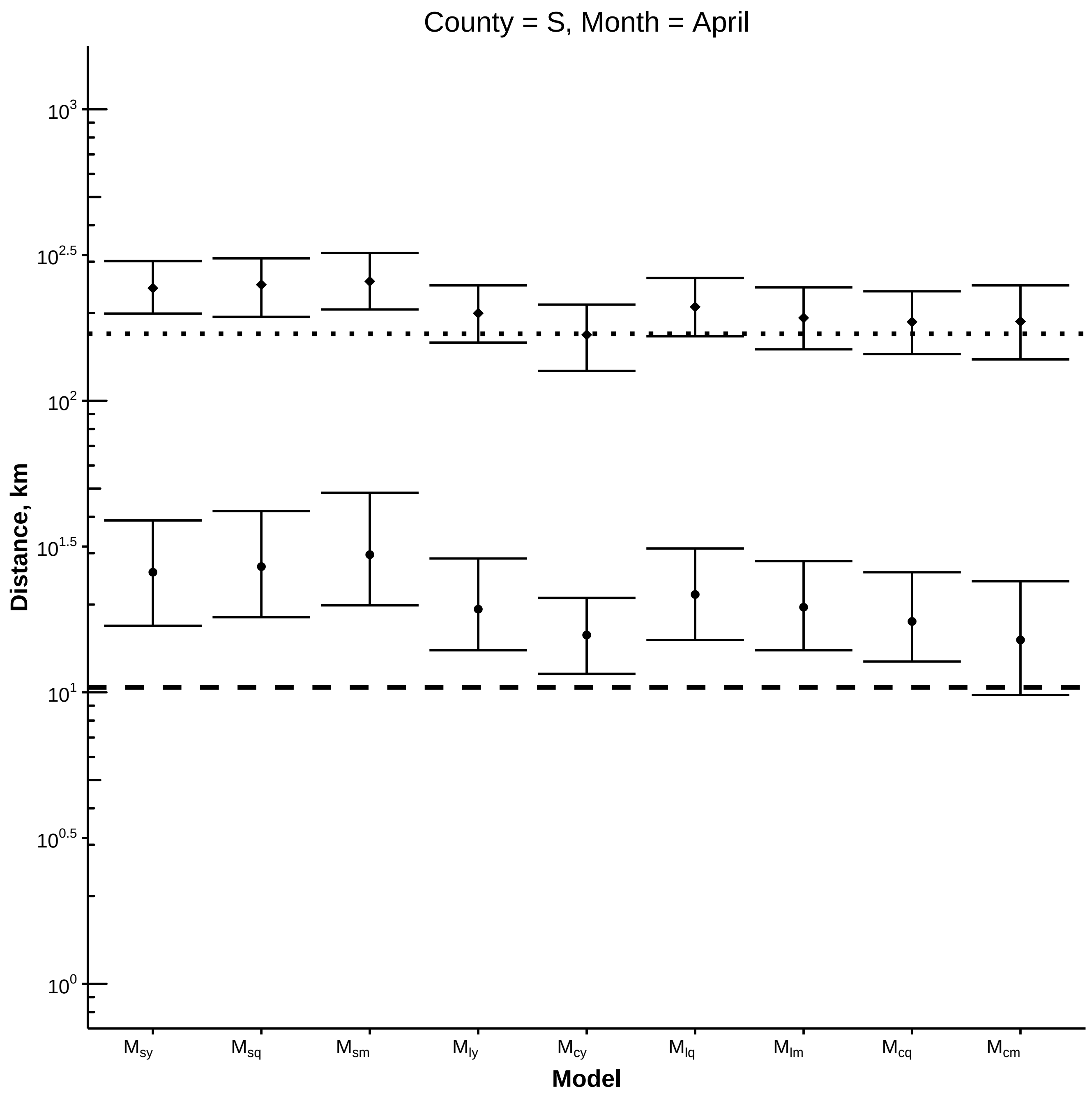
County = S, Month = April
Distance, km
10^3
$10^{2.5}$
10^2
$10^{1.5}$
10^1
$10^{0.5}$
10^0
M_{sy}
M_{sq}
M_{sm}
M_{ly}
M_{cy}
M_{lq}
M_{lm}
M_{cq}
M_{cm}
Model

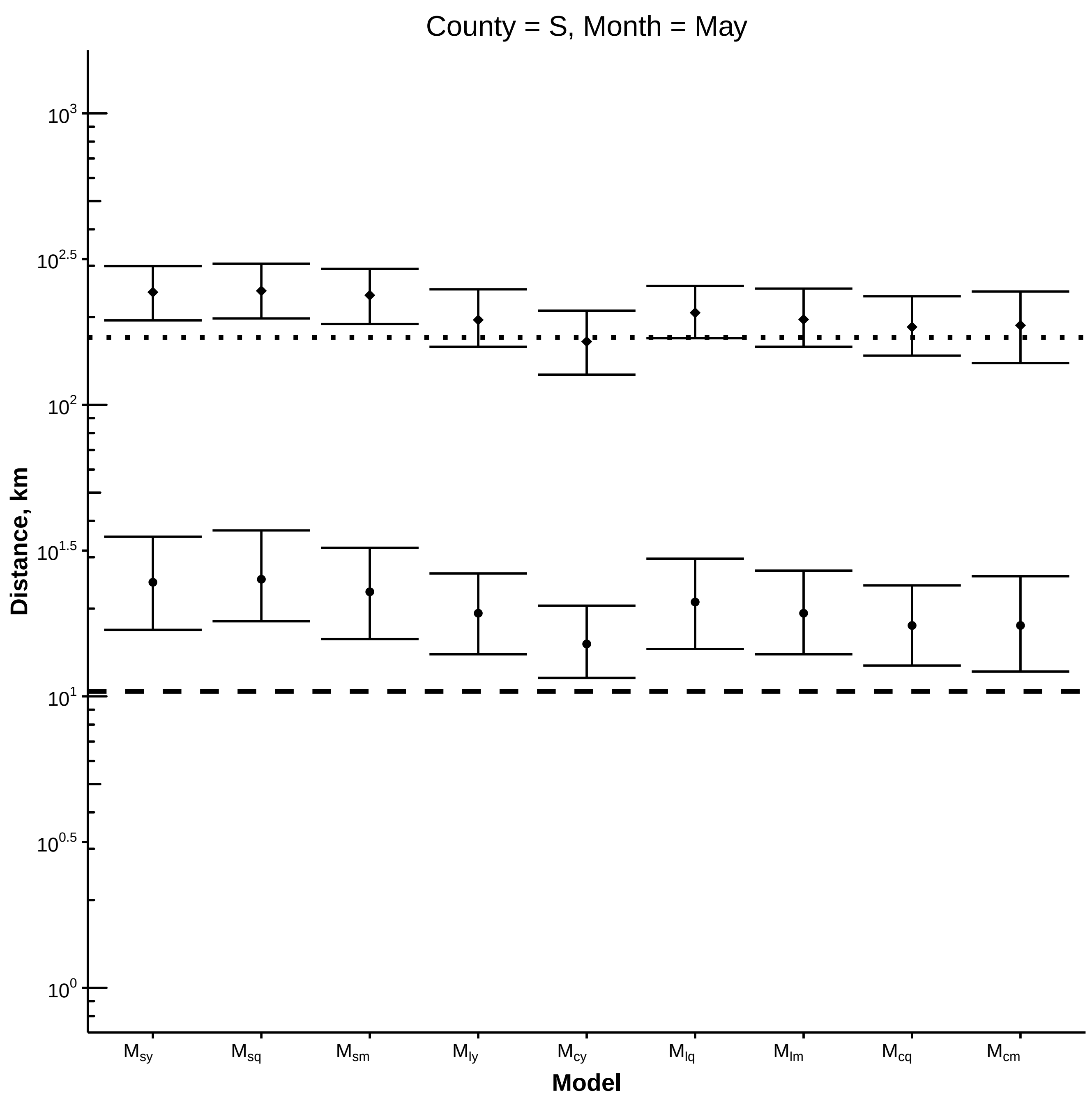

County = S, Month = May
Distance, km
10^3
$10^{2.5}$
10^2
$10^{1.5}$
10^1
$10^{0.5}$
10^0
M_{sy}
M_{sq}
M_{sm}
M_{ly}
M_{cy}
M_{lq}
M_{lm}
M_{cq}
M_{cm}
Model

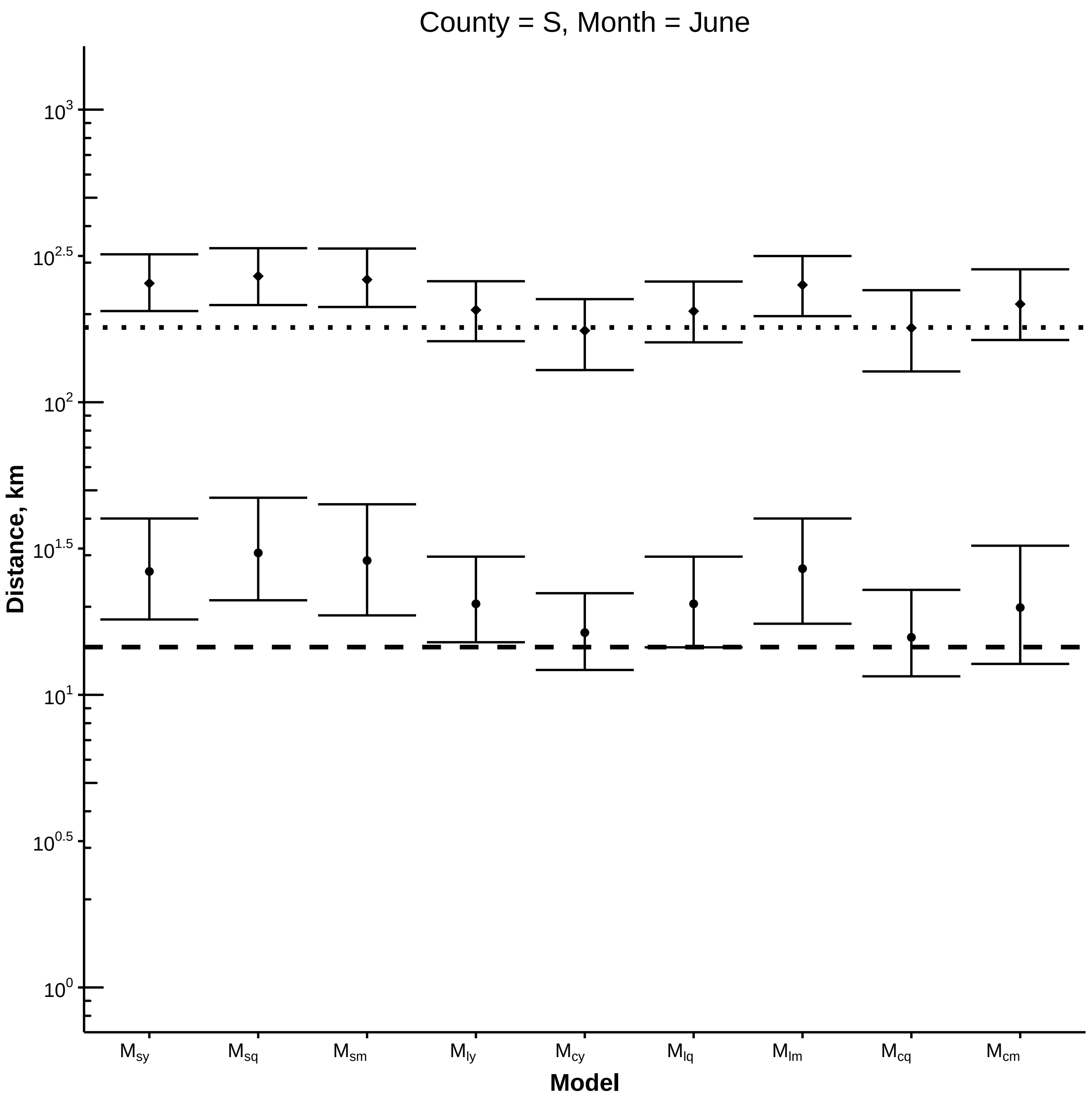

County = S, Month = June
Distance, km
Model
10^3
$10^{2.5}$
10^2
$10^{1.5}$
10^1
$10^{0.5}$
10^0
M_{sy}
M_{sq}
M_{sm}
M_{ly}
M_{cy}
M_{lq}
M_{lm}
M_{cq}
M_{cm}

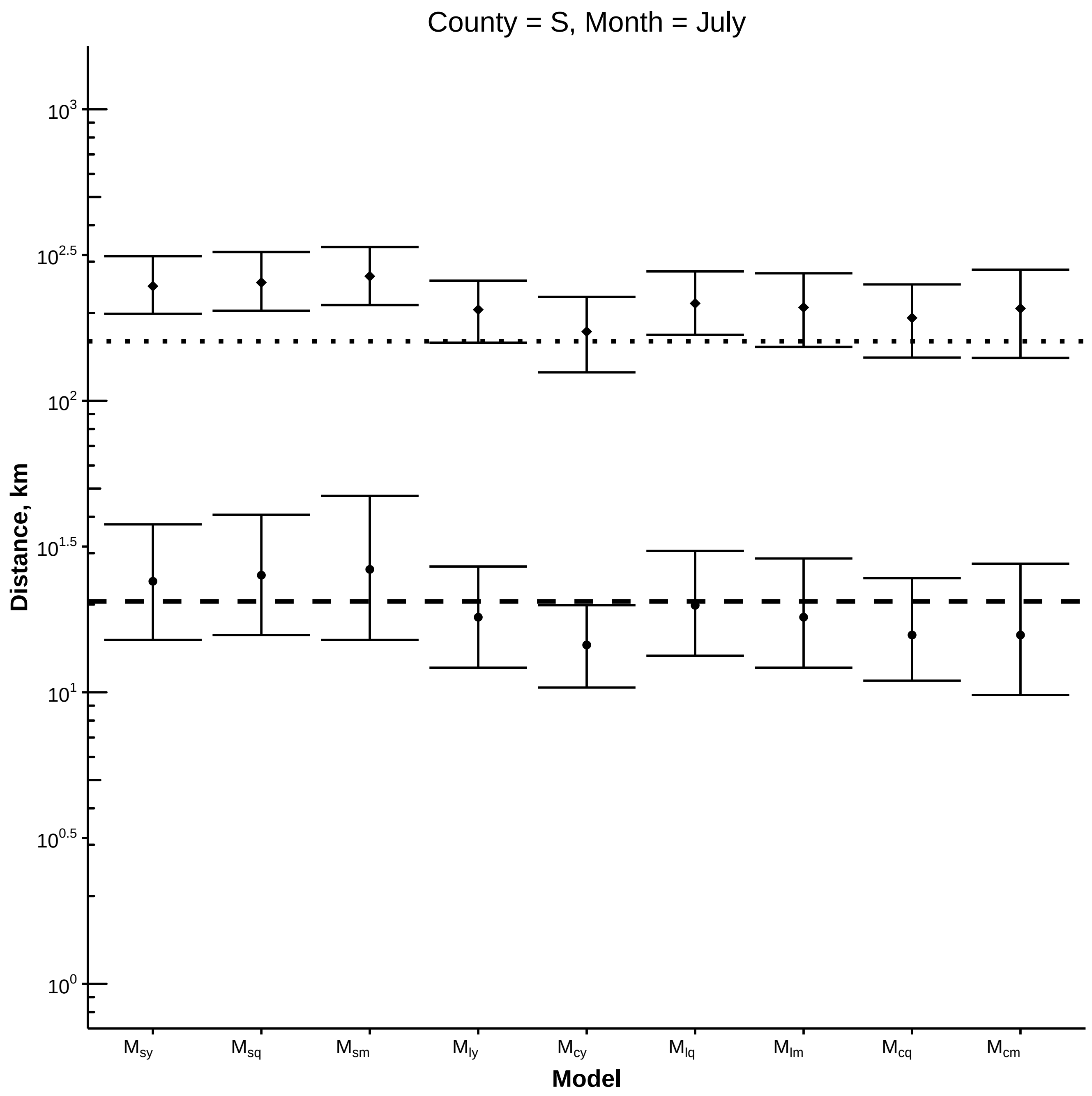

County = S, Month = July
Distance, km
10^3
$10^{2.5}$
10^2
$10^{1.5}$
10^1
$10^{0.5}$
10^0
M_{sy}
M_{sq}
M_{sm}
M_{ly}
M_{cy}
M_{lq}
M_{lm}
M_{cq}
M_{cm}
Model

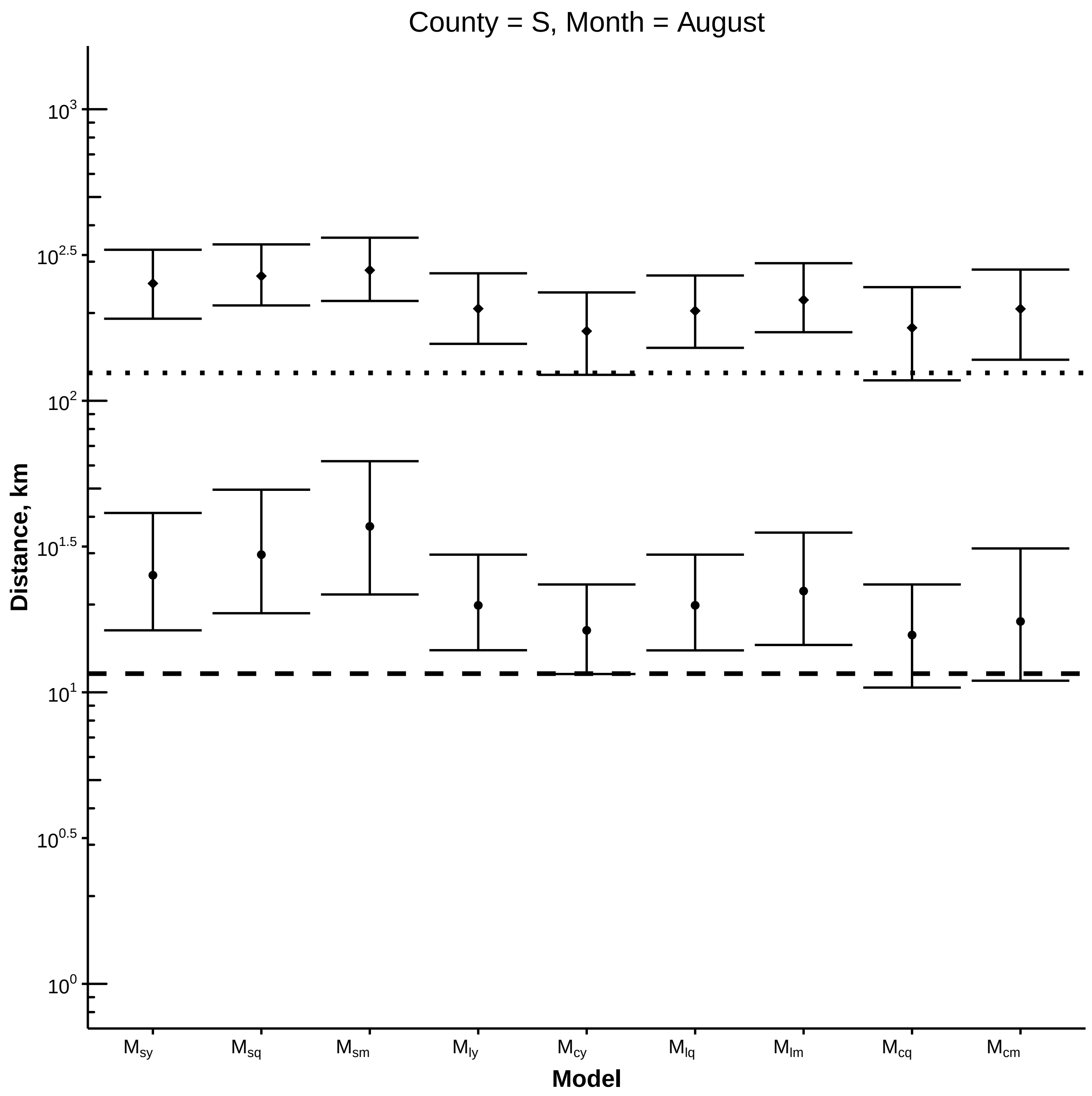

County = S, Month = August
Distance, km
10^3
$10^{2.5}$
10^2
$10^{1.5}$
10^1
$10^{0.5}$
10^0
M_{sy}
M_{sq}
M_{sm}
M_{ly}
M_{cy}
M_{lq}
M_{lm}
M_{cq}
M_{cm}
Model

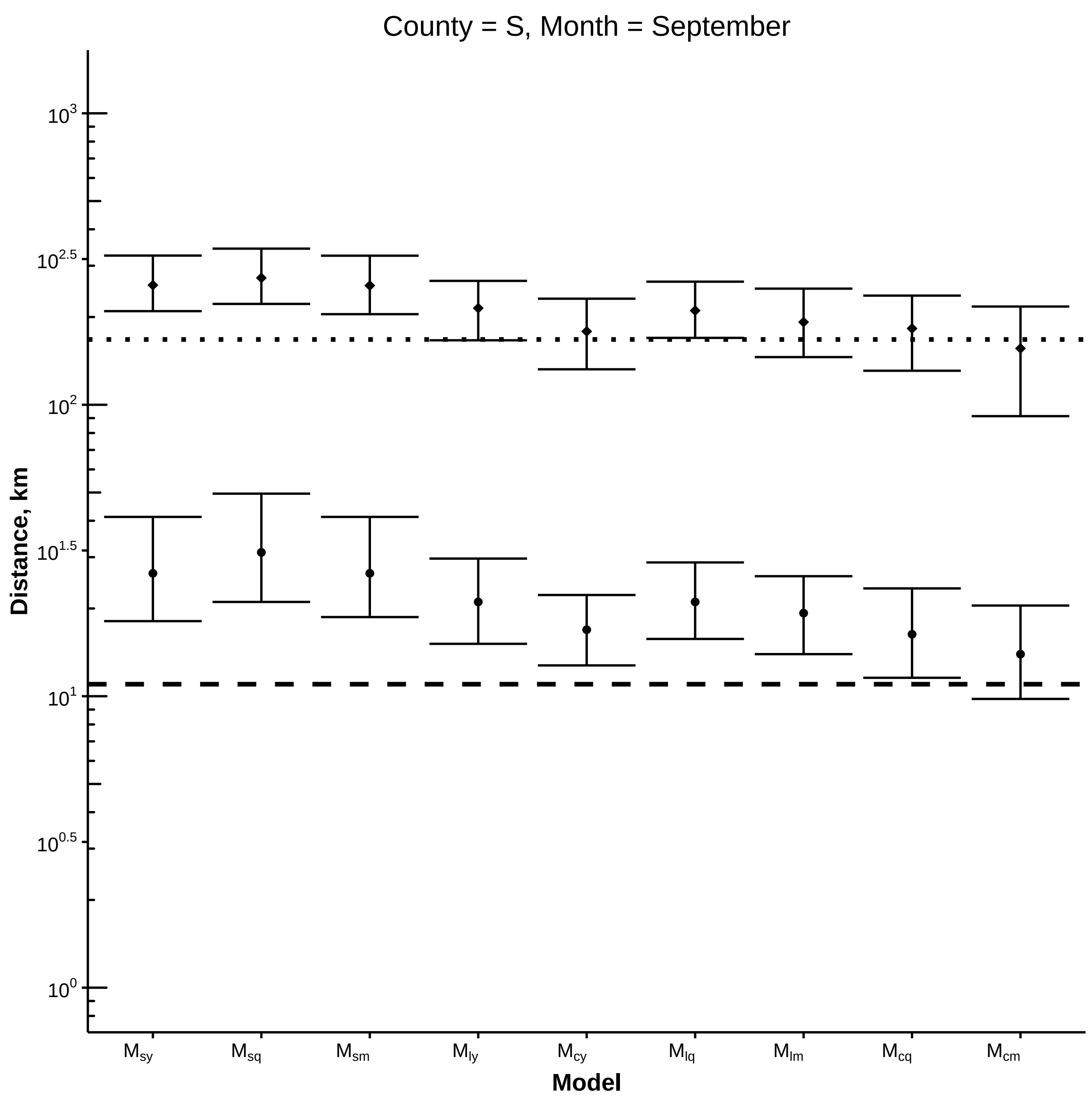

County = S, Month = September
Distance, km
10^3
$10^{2.5}$
10^2
$10^{1.5}$
10^1
$10^{0.5}$
10^0
M_{sy}
M_{sq}
M_{sm}
M_{ly}
M_{cy}
M_{lq}
M_{lm}
M_{cq}
M_{cm}
Model

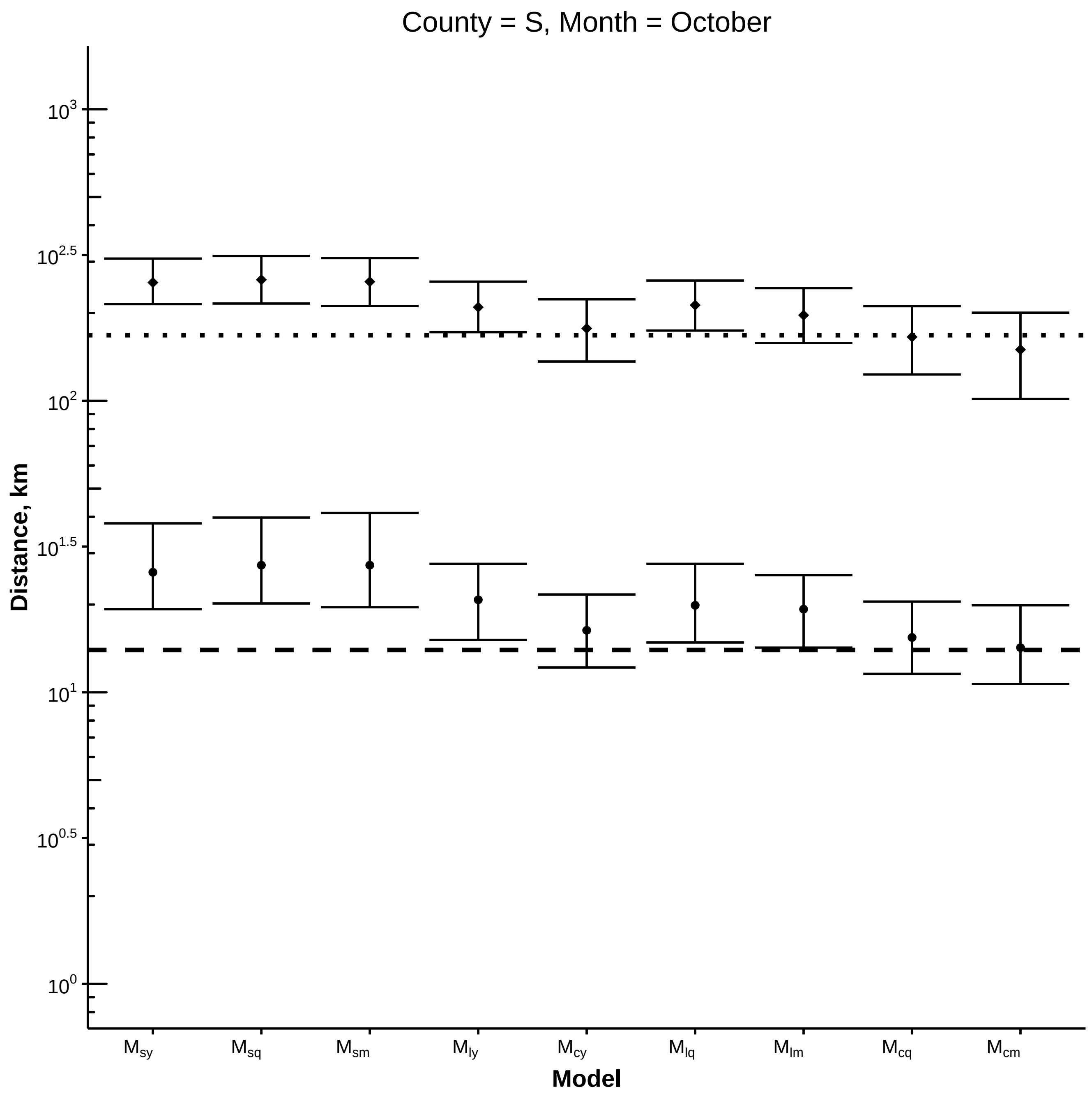

County = S, Month = October
Distance, km
10^{3}
$10^{2.5}$
10^{2}
$10^{1.5}$
10^{1}
$10^{0.5}$
10^{0}
M_{sy}
M_{sq}
M_{sm}
M_{ly}
M_{cy}
M_{lq}
M_{lm}
M_{cq}
M_{cm}
Model

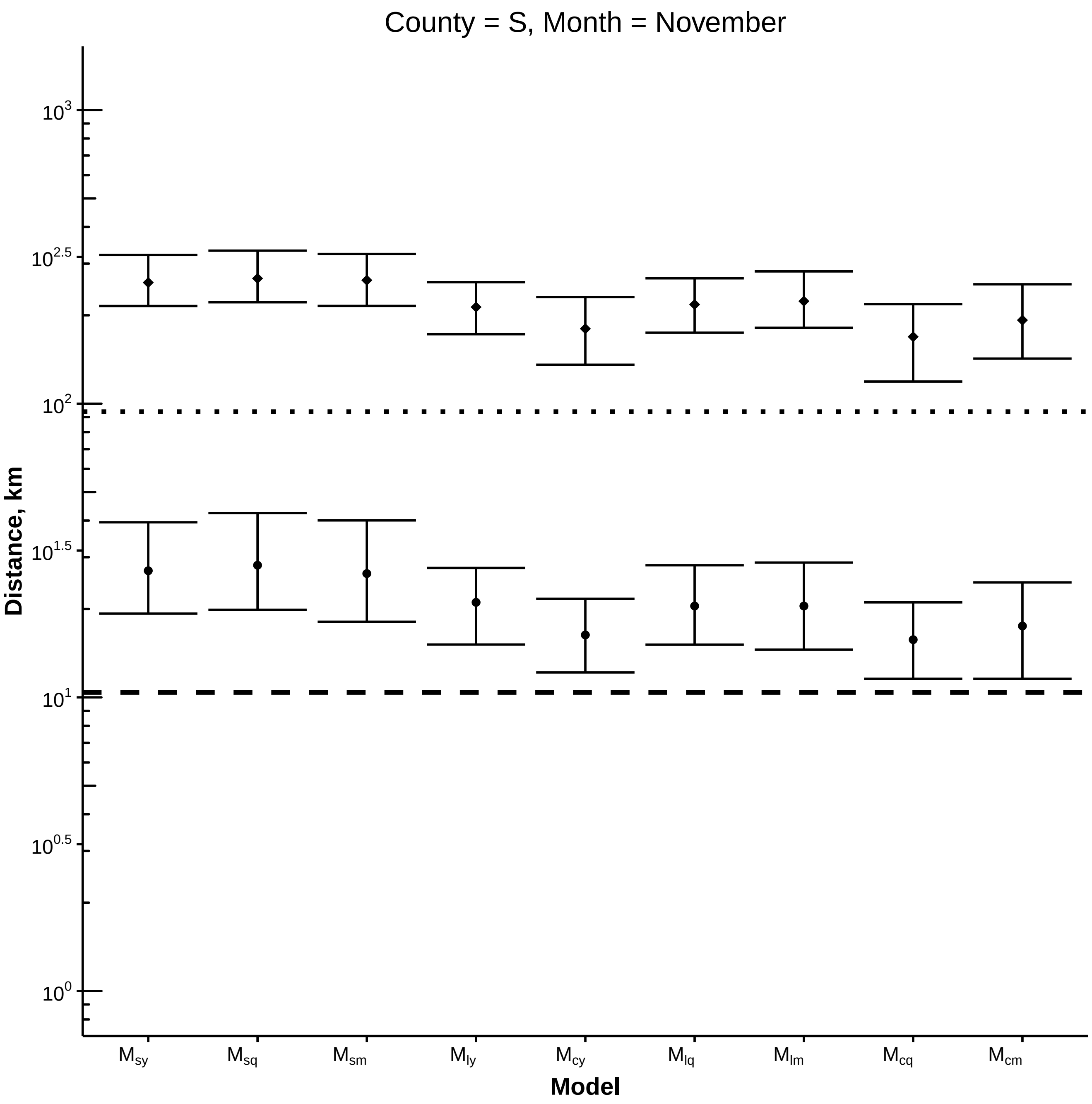

County = S, Month = November
Distance, km
10^3
$10^{2.5}$
10^2
$10^{1.5}$
10^1
$10^{0.5}$
10^0
M_{sy}
M_{sq}
M_{sm}
M_{ly}
M_{cy}
M_{lq}
M_{lm}
M_{cq}
M_{cm}
Model

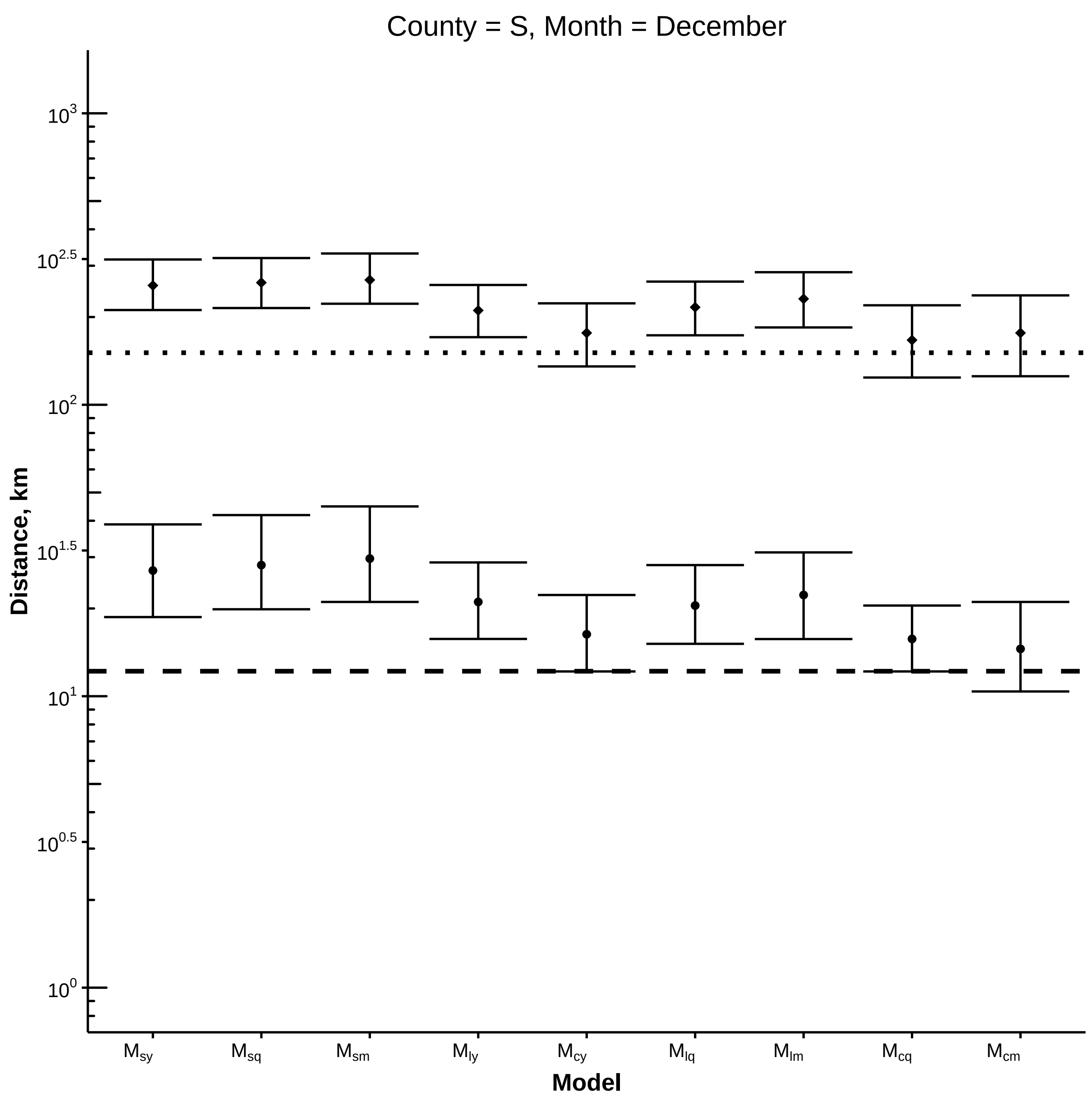
County = S, Month = December
Distance, km
10^3
$10^{2.5}$
10^2
$10^{1.5}$
10^1
$10^{0.5}$
10^0
M_{sy}
M_{sq}
M_{sm}
M_{ly}
M_{cy}
M_{lq}
M_{lm}
M_{cq}
M_{cm}
Model

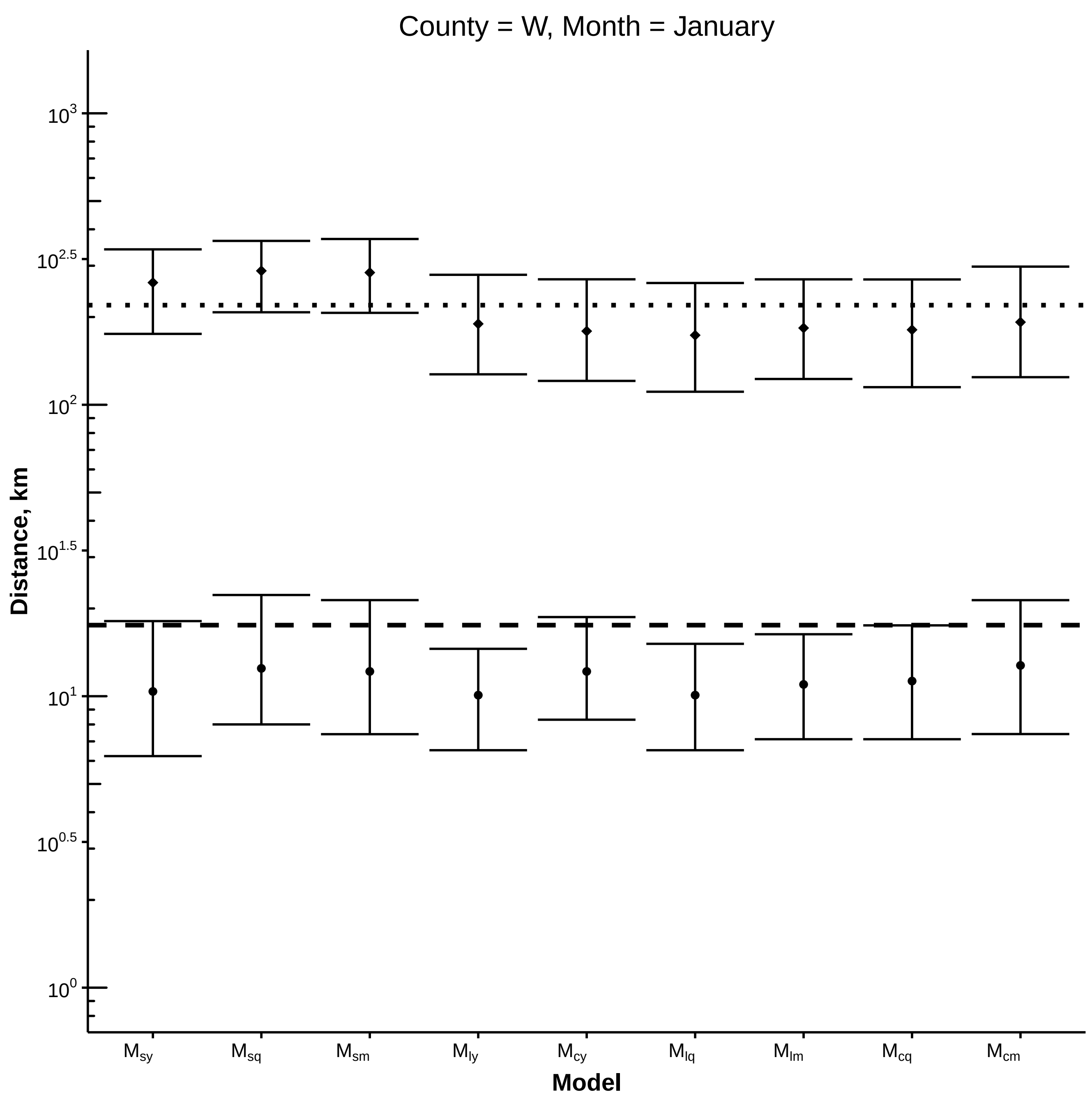

County = W, Month = January
Distance, km
10^3
$10^{2.5}$
10^2
$10^{1.5}$
10^1
$10^{0.5}$
10^0
M_{sy}
M_{sq}
M_{sm}
M_{ly}
M_{cy}
M_{lq}
M_{lm}
M_{cq}
M_{cm}
Model

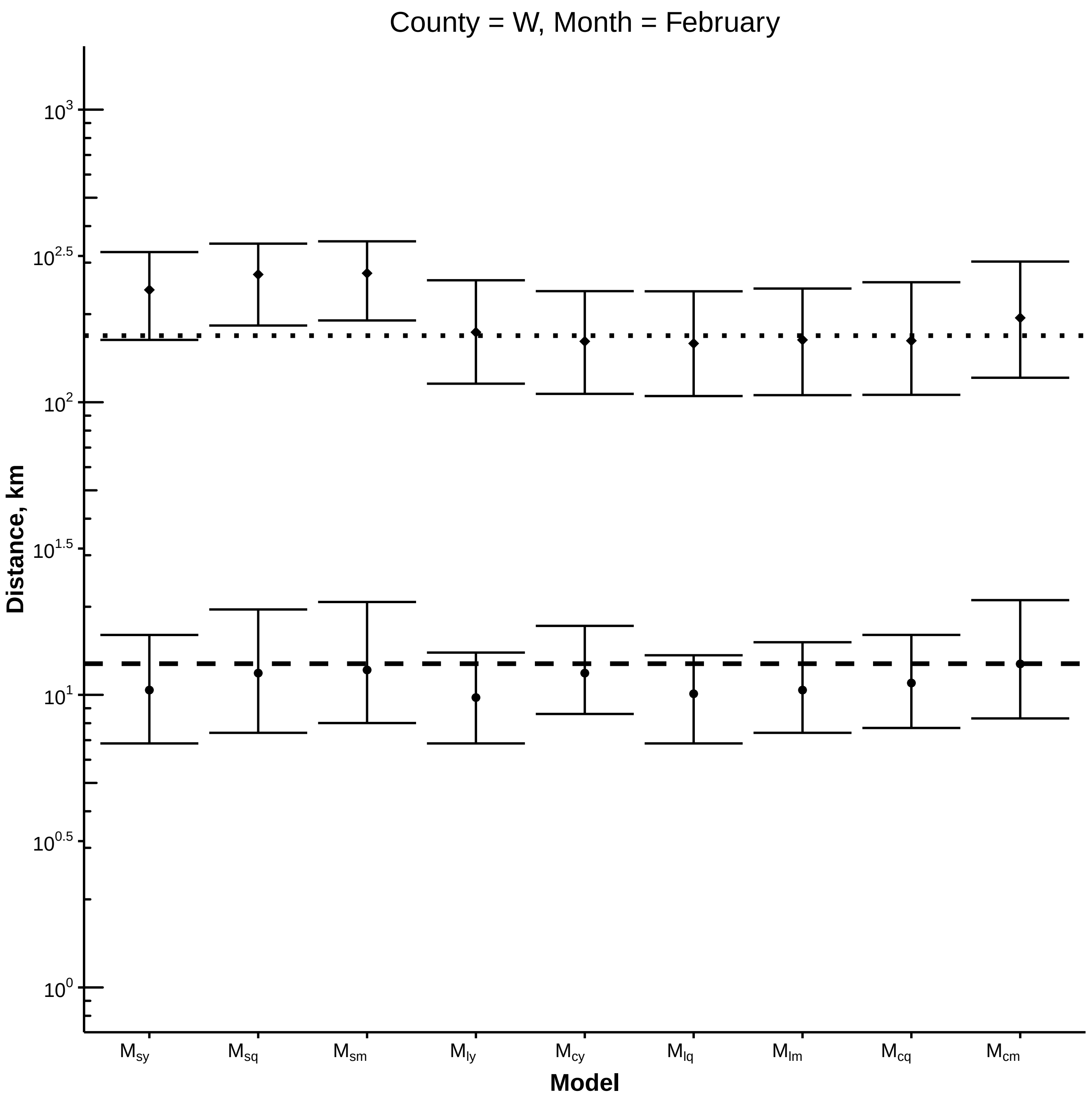

County = W, Month = February
Distance, km
10^{3}
$10^{2.5}$
10^{2}
$10^{1.5}$
10^{1}
$10^{0.5}$
10^{0}
M_{sy}
M_{sq}
M_{sm}
M_{ly}
M_{cy}
M_{lq}
M_{lm}
M_{cq}
M_{cm}
Model

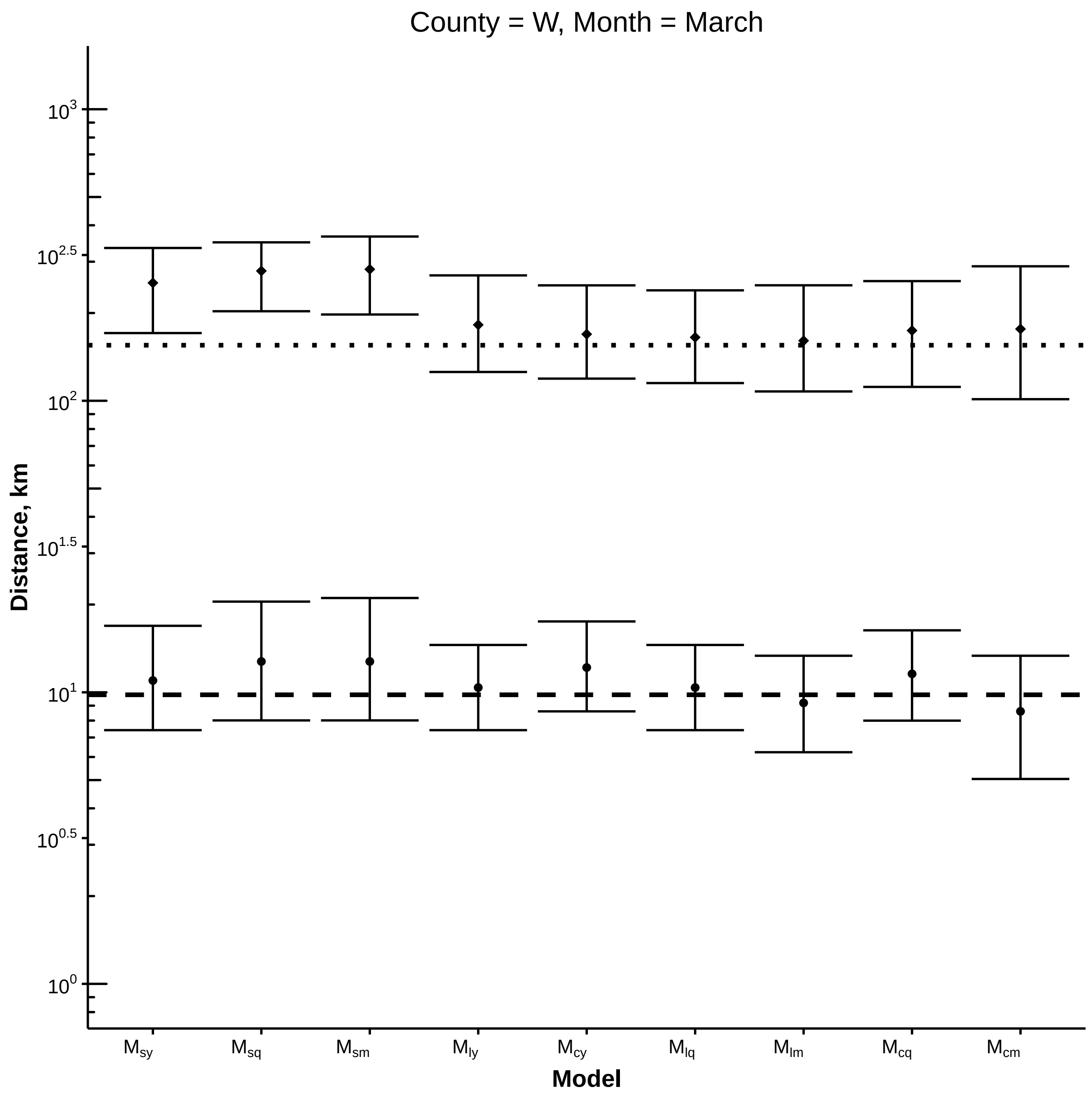
County = W, Month = March
Distance, km
10^{3}
$10^{2.5}$
10^{2}
$10^{1.5}$
10^{1}
$10^{0.5}$
10^{0}
M_{sy}
M_{sq}
M_{sm}
M_{ly}
M_{cy}
M_{lq}
M_{lm}
M_{cq}
M_{cm}
Model

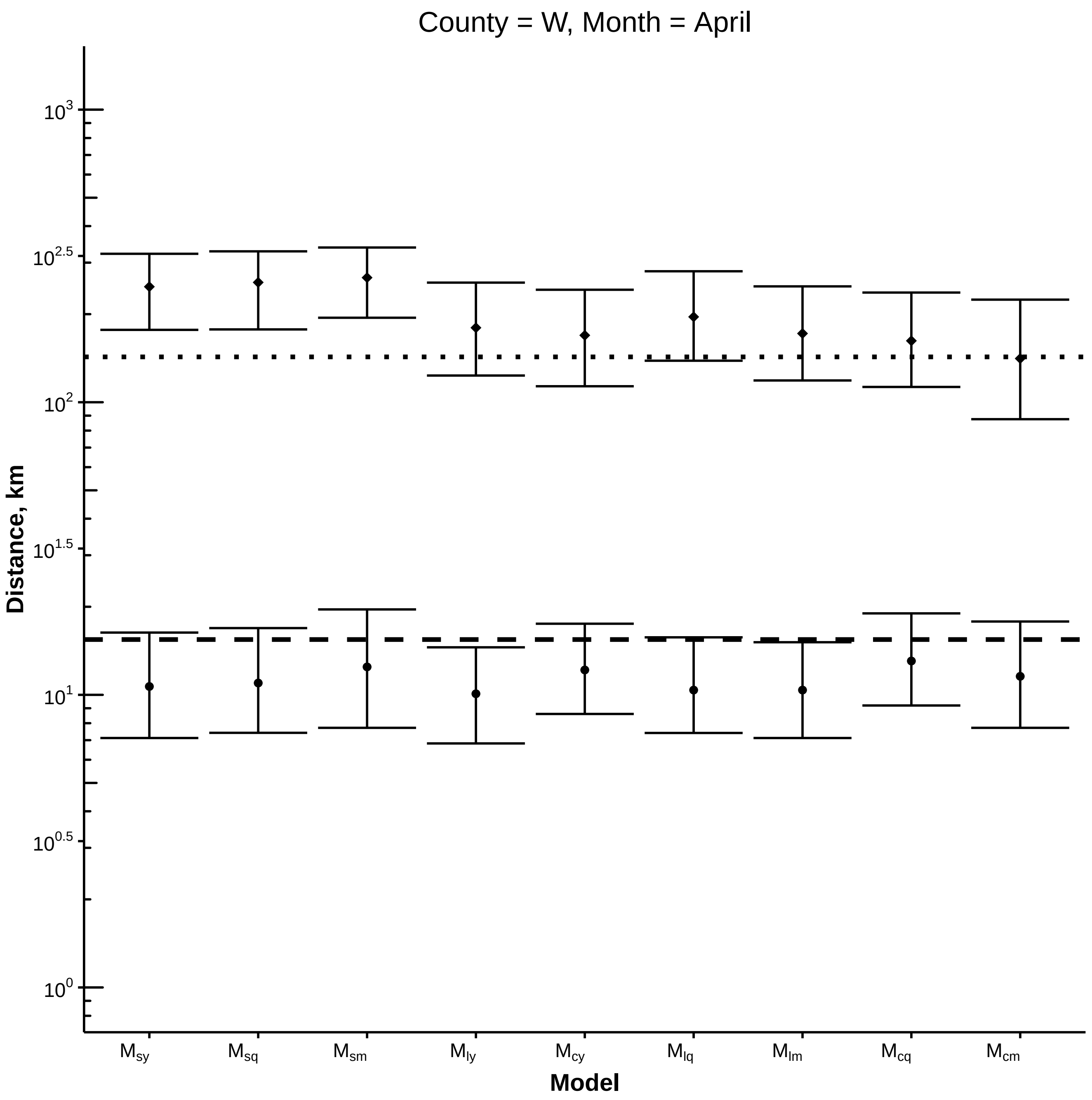
County = W, Month = April
Distance, km
10^3
$10^{2.5}$
10^2
$10^{1.5}$
10^1
$10^{0.5}$
10^0
M_{sy}
M_{sq}
M_{sm}
M_{ly}
M_{cy}
M_{lq}
M_{lm}
M_{cq}
M_{cm}
Model

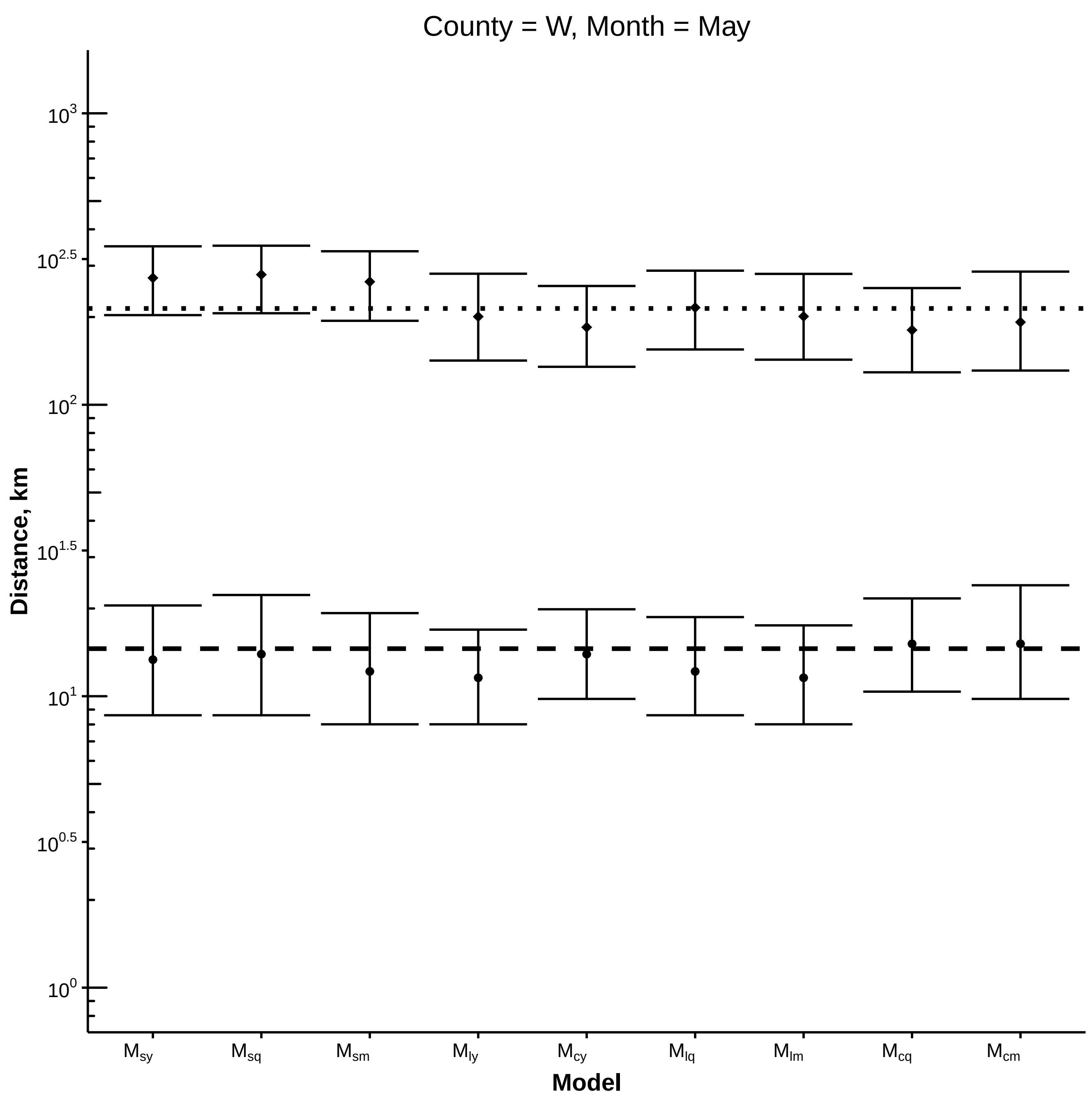

County = W, Month = May
Distance, km
10^3
$10^{2.5}$
10^2
$10^{1.5}$
10^1
$10^{0.5}$
10^0
M_{sy}
M_{sq}
M_{sm}
M_{ly}
M_{cy}
M_{lq}
M_{lm}
M_{cq}
M_{cm}
Model

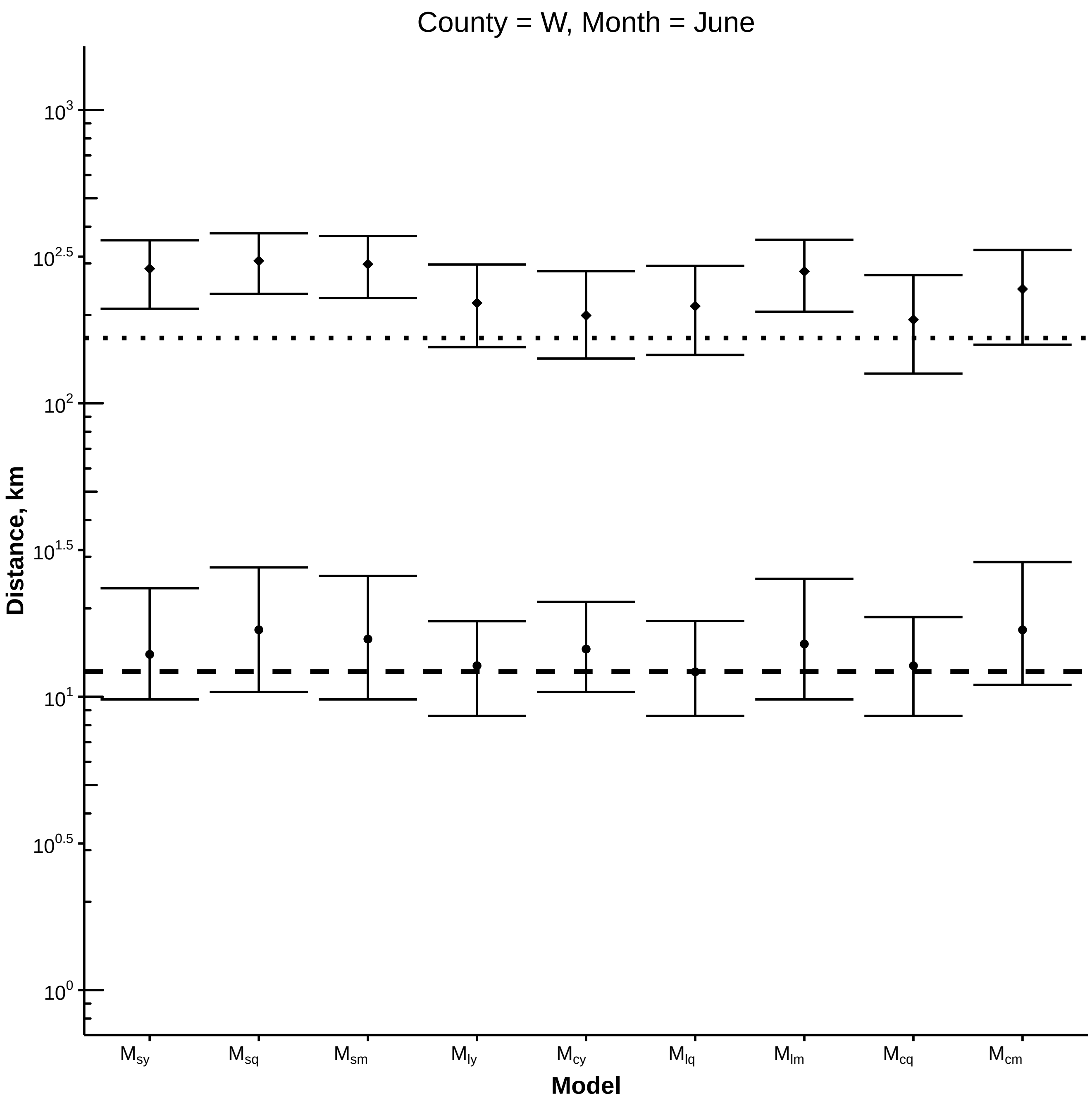
County = W, Month = June
Distance, km
10^3
10^2.5
10^2
10^1.5
10^1
10^0.5
10^0
M_sy
M_sq
M_sm
M_ly
M_cy
M_lq
M_lm
M_cq
M_cm
Model

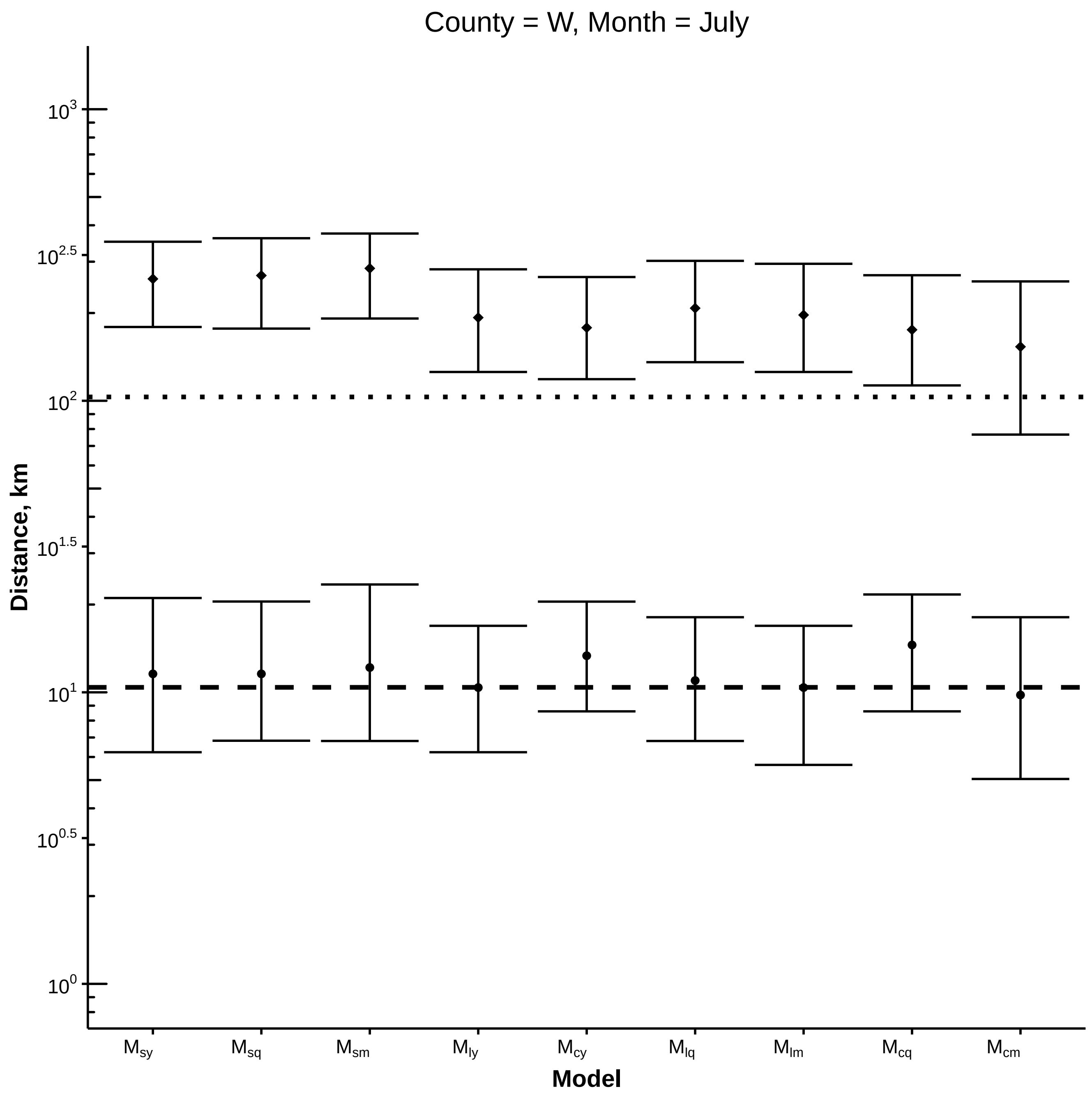

County = W, Month = July
Distance, km
10^3
$10^{2.5}$
10^2
$10^{1.5}$
10^1
$10^{0.5}$
10^0
M_{sy}
M_{sq}
M_{sm}
M_{ly}
M_{cy}
M_{lq}
M_{lm}
M_{cq}
M_{cm}
Model

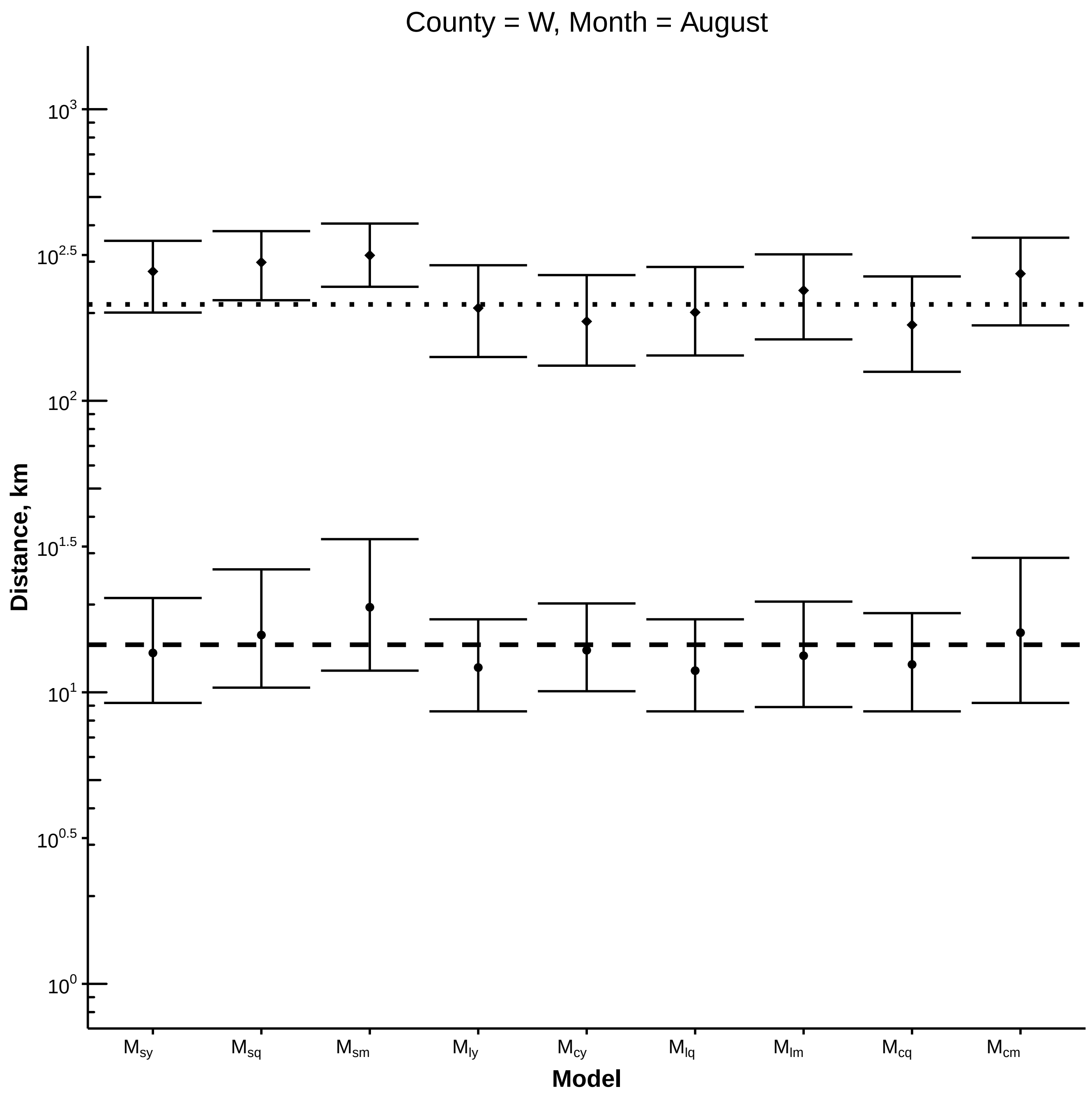

County = W, Month = August
Distance, km
10^3
$10^{2.5}$
10^2
$10^{1.5}$
10^1
$10^{0.5}$
10^0
M_{sy}
M_{sq}
M_{sm}
M_{ly}
M_{cy}
M_{lq}
M_{lm}
M_{cq}
M_{cm}
Model

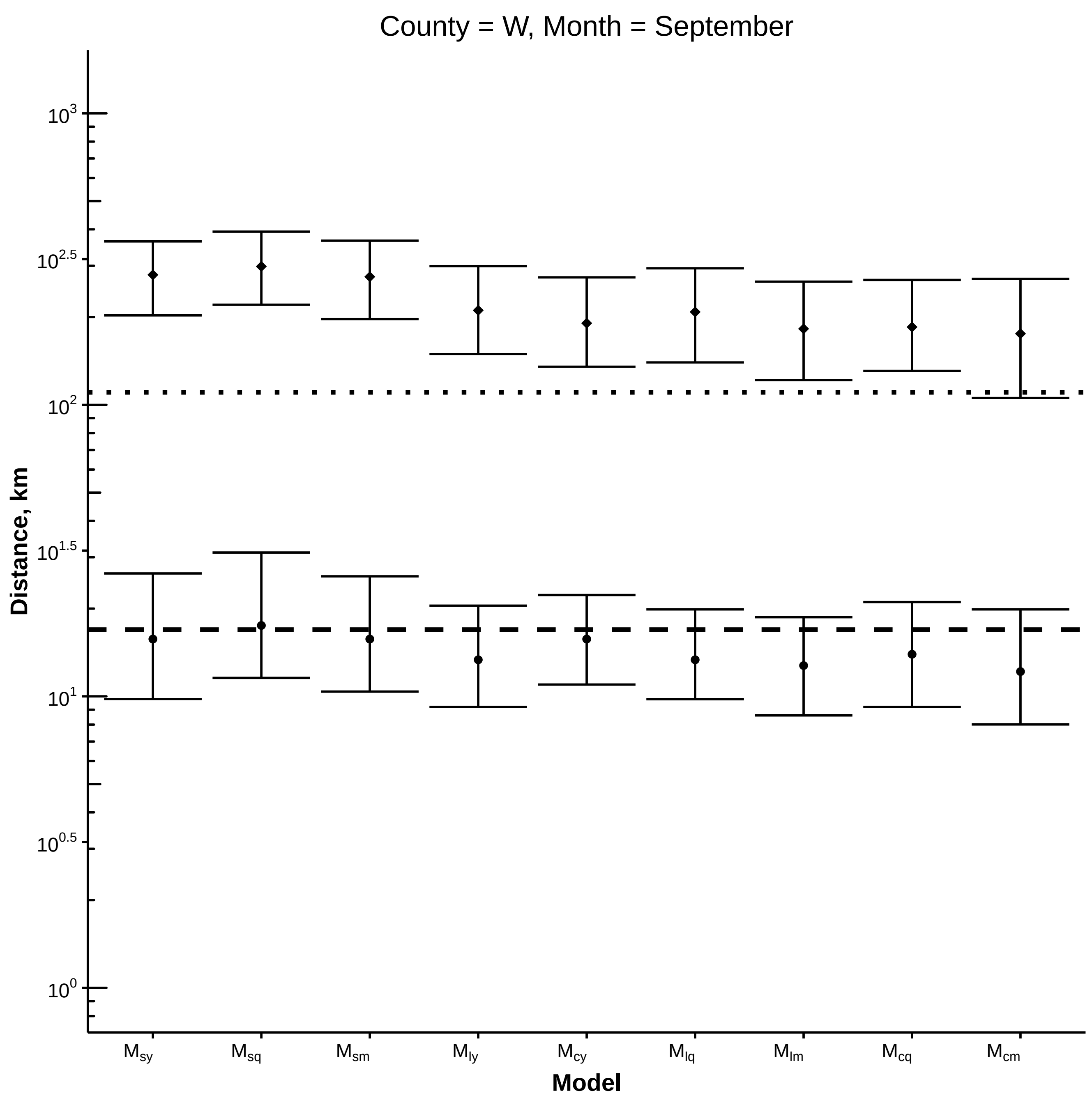

County = W, Month = September
Distance, km
10^3
$10^{2.5}$
10^2
$10^{1.5}$
10^1
$10^{0.5}$
10^0
M_{sy}
M_{sq}
M_{sm}
M_{ly}
M_{cy}
M_{lq}
M_{lm}
M_{cq}
M_{cm}
Model

County = W, Month = October

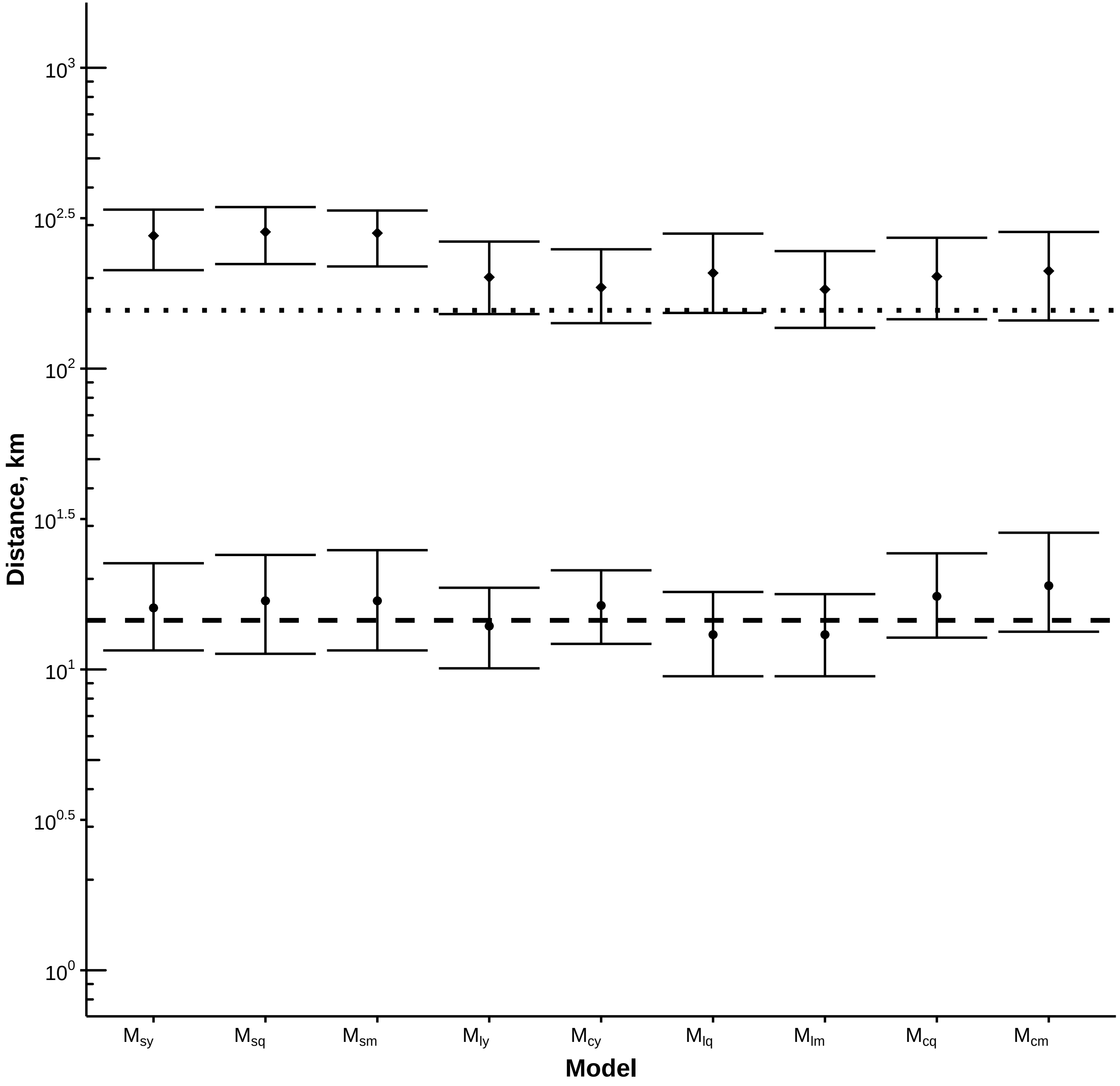

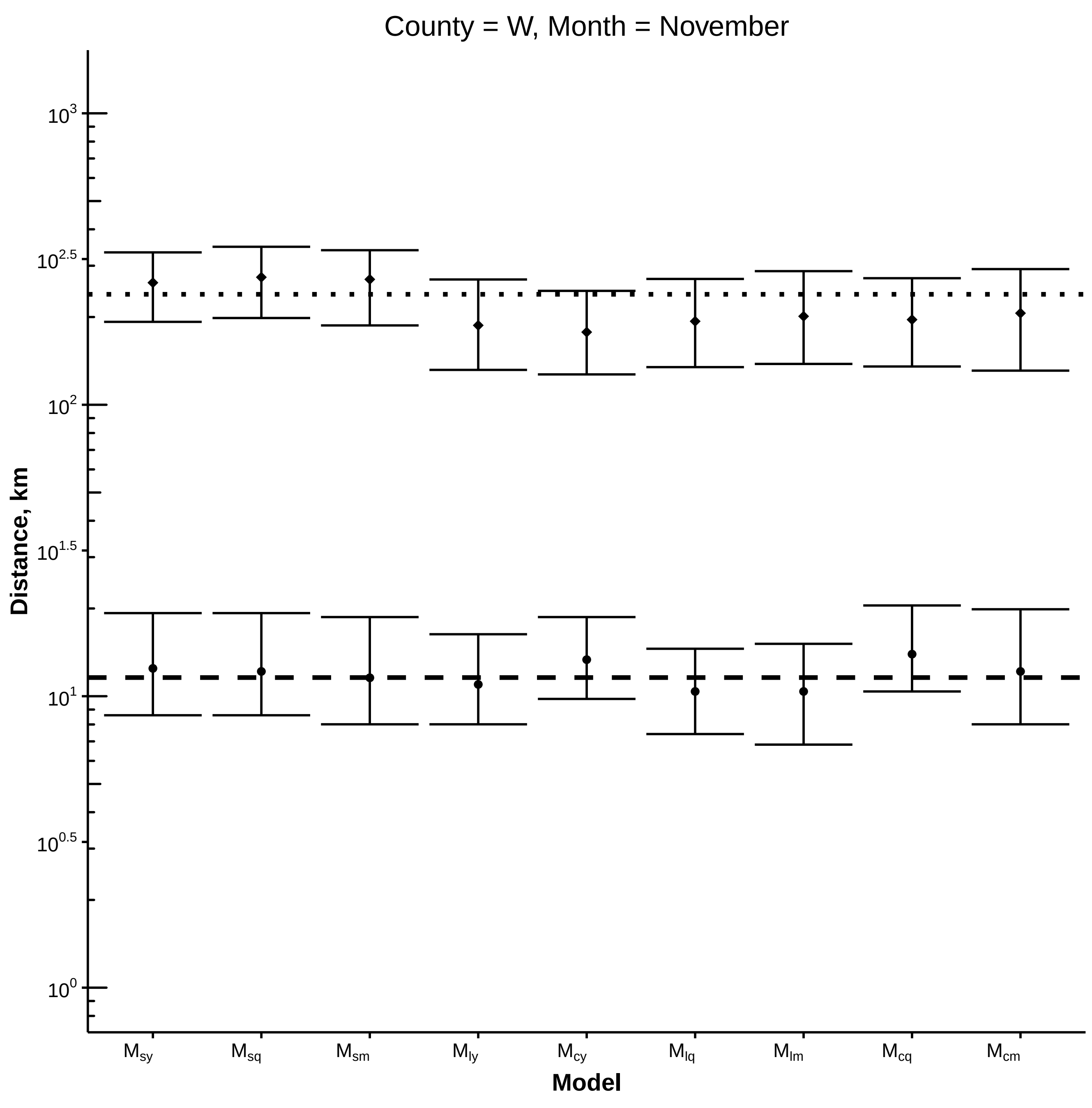

County = W, Month = November
Distance, km
10^3
$10^{2.5}$
10^2
$10^{1.5}$
10^1
$10^{0.5}$
10^0
M_{sy}
M_{sq}
M_{sm}
M_{ly}
M_{cy}
M_{lq}
M_{lm}
M_{cq}
M_{cm}
Model

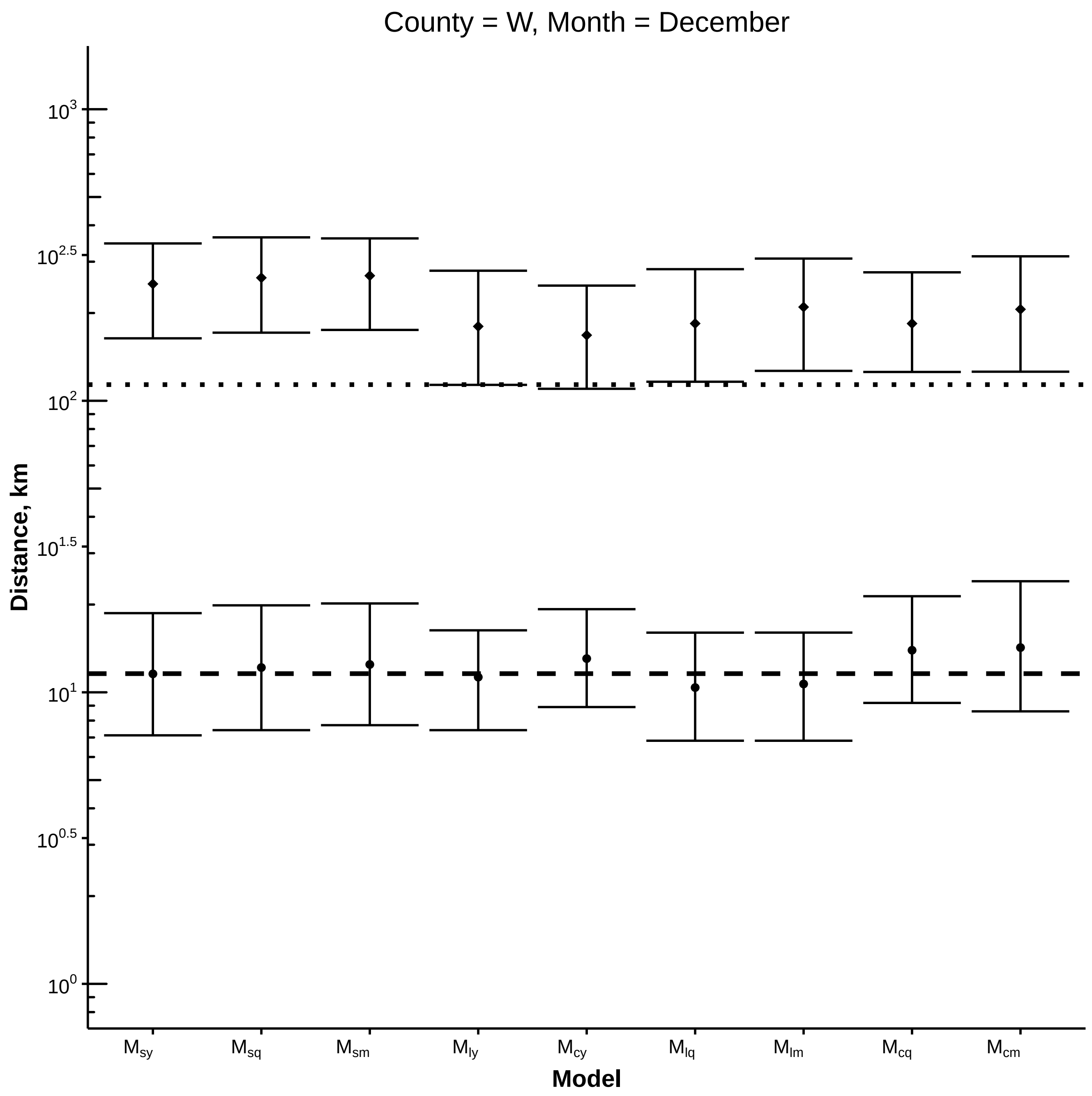
County = W, Month = December
Distance, km
10^3
10^2.5
10^2
10^1.5
10^1
10^0.5
10^0
Msy
Msq
Msm
Mly
Mcy
Mlq
Mlm
Mcq
Mcm
Model

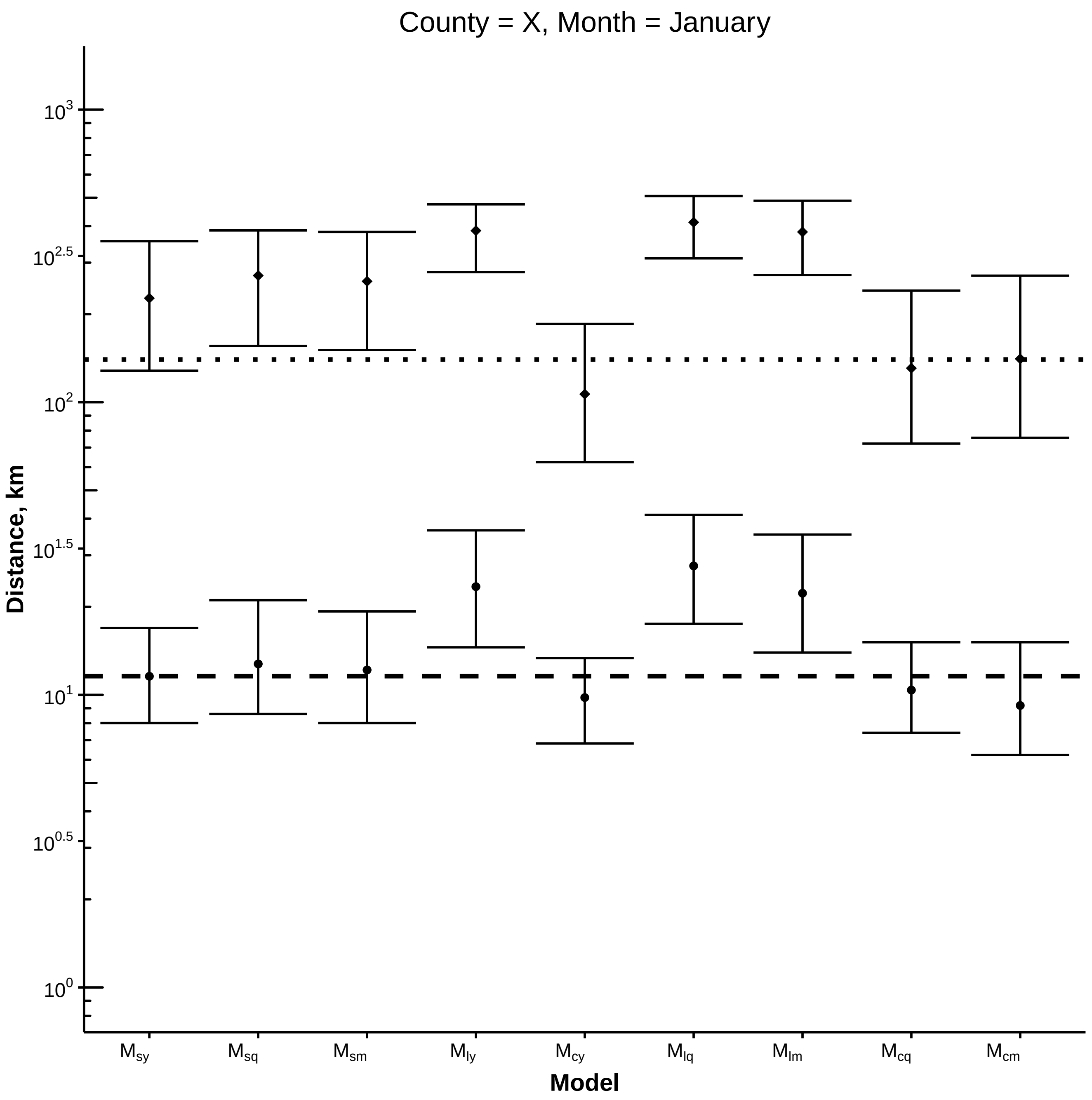

County = X, Month = January
Distance, km
10^3
$10^{2.5}$
10^2
$10^{1.5}$
10^1
$10^{0.5}$
10^0
M_{sy}
M_{sq}
M_{sm}
M_{ly}
M_{cy}
M_{lq}
M_{lm}
M_{cq}
M_{cm}
Model

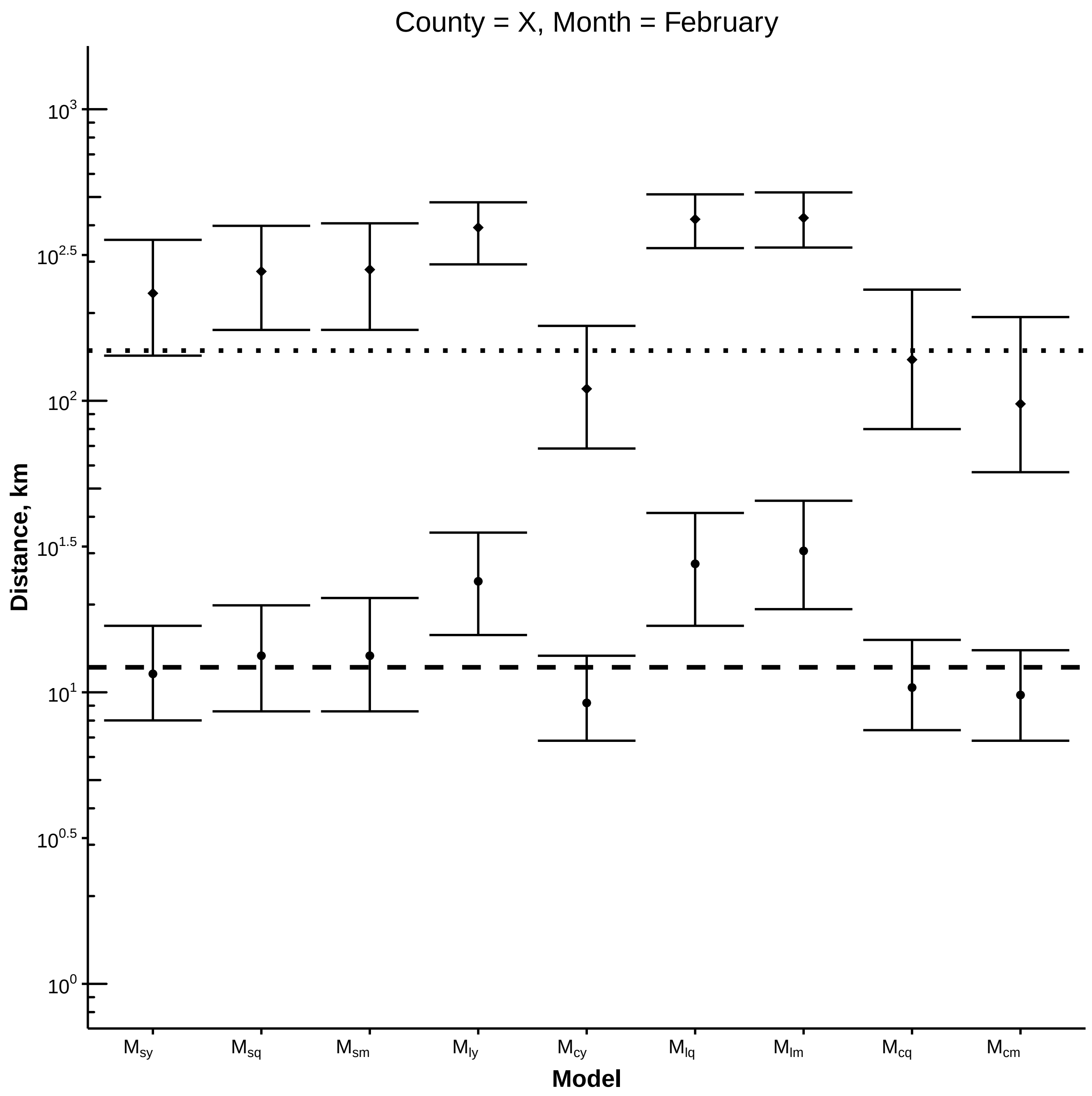

County = X, Month = February
Distance, km
10^3
$10^{2.5}$
10^2
$10^{1.5}$
10^1
$10^{0.5}$
10^0
M_{sy}
M_{sq}
M_{sm}
M_{ly}
M_{cy}
M_{lq}
M_{lm}
M_{cq}
M_{cm}
Model

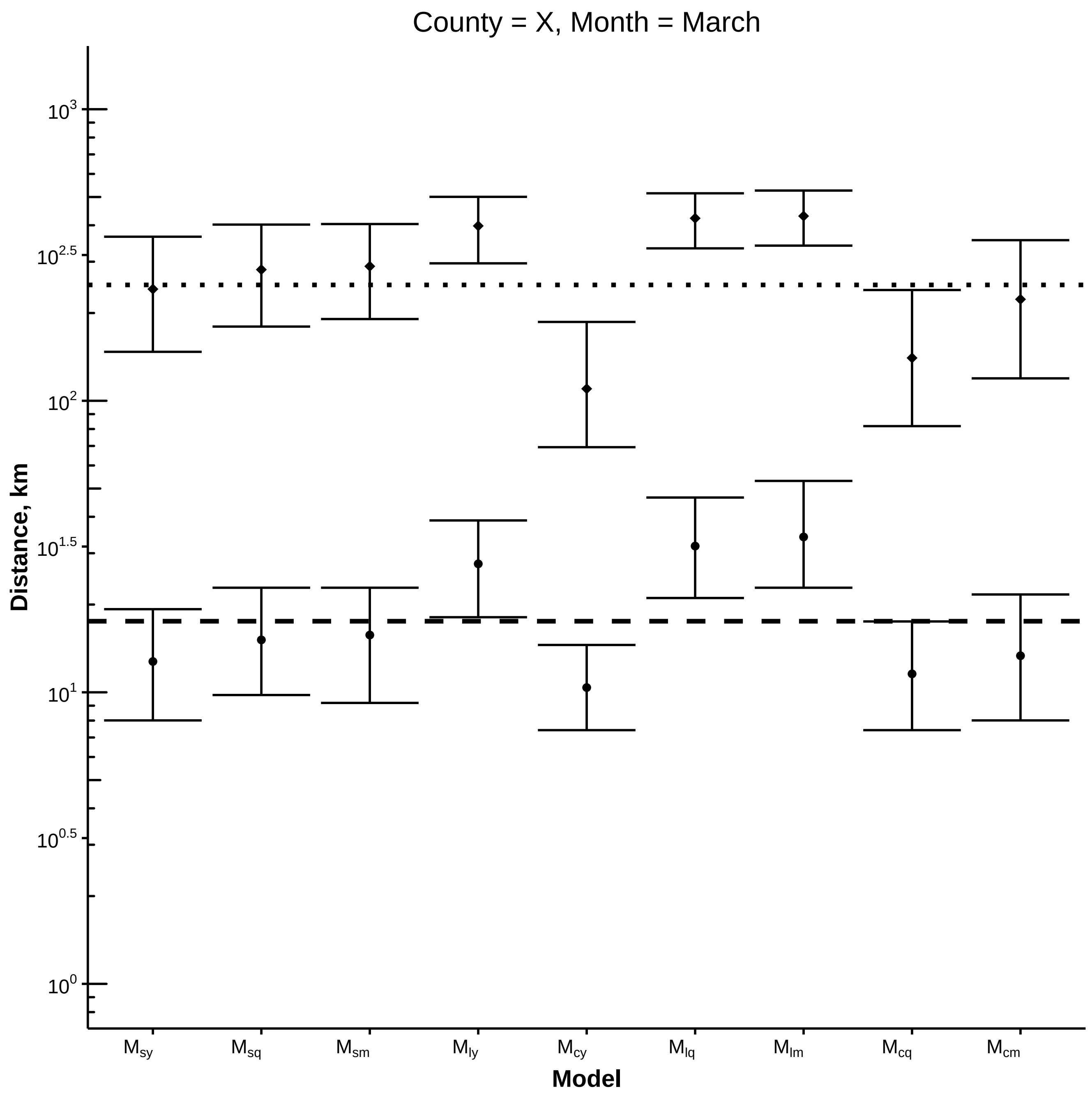

County = X, Month = March
Distance, km
10^3
$10^{2.5}$
10^2
$10^{1.5}$
10^1
$10^{0.5}$
10^0
M_{sy}
M_{sq}
M_{sm}
M_{ly}
M_{cy}
M_{lq}
M_{lm}
M_{cq}
M_{cm}
Model

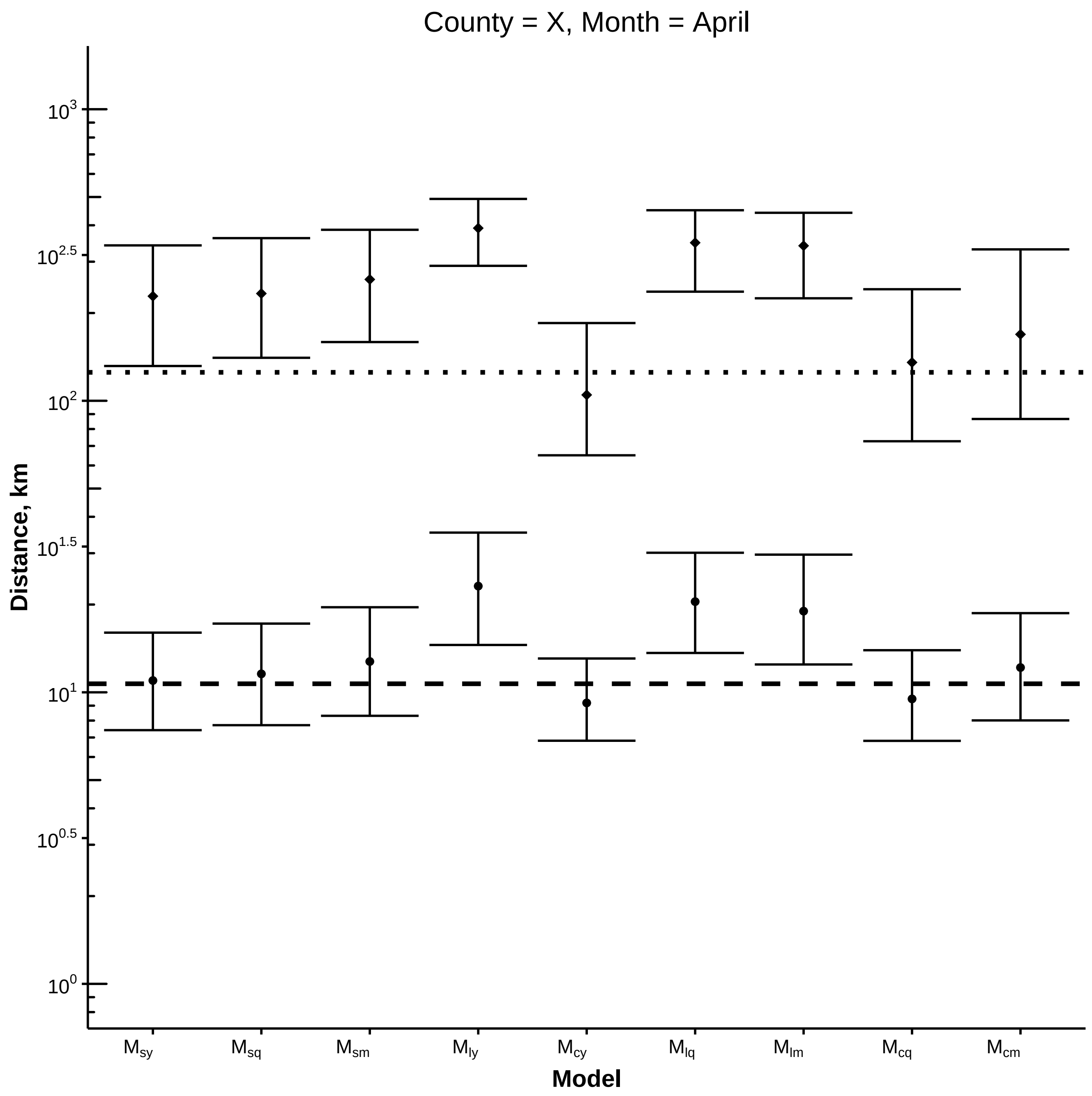
County = X, Month = April
Distance, km
10^{3}
$10^{2.5}$
10^{2}
$10^{1.5}$
10^{1}
$10^{0.5}$
10^{0}
M_{sy}
M_{sq}
M_{sm}
M_{ly}
M_{cy}
M_{lq}
M_{lm}
M_{cq}
M_{cm}
Model

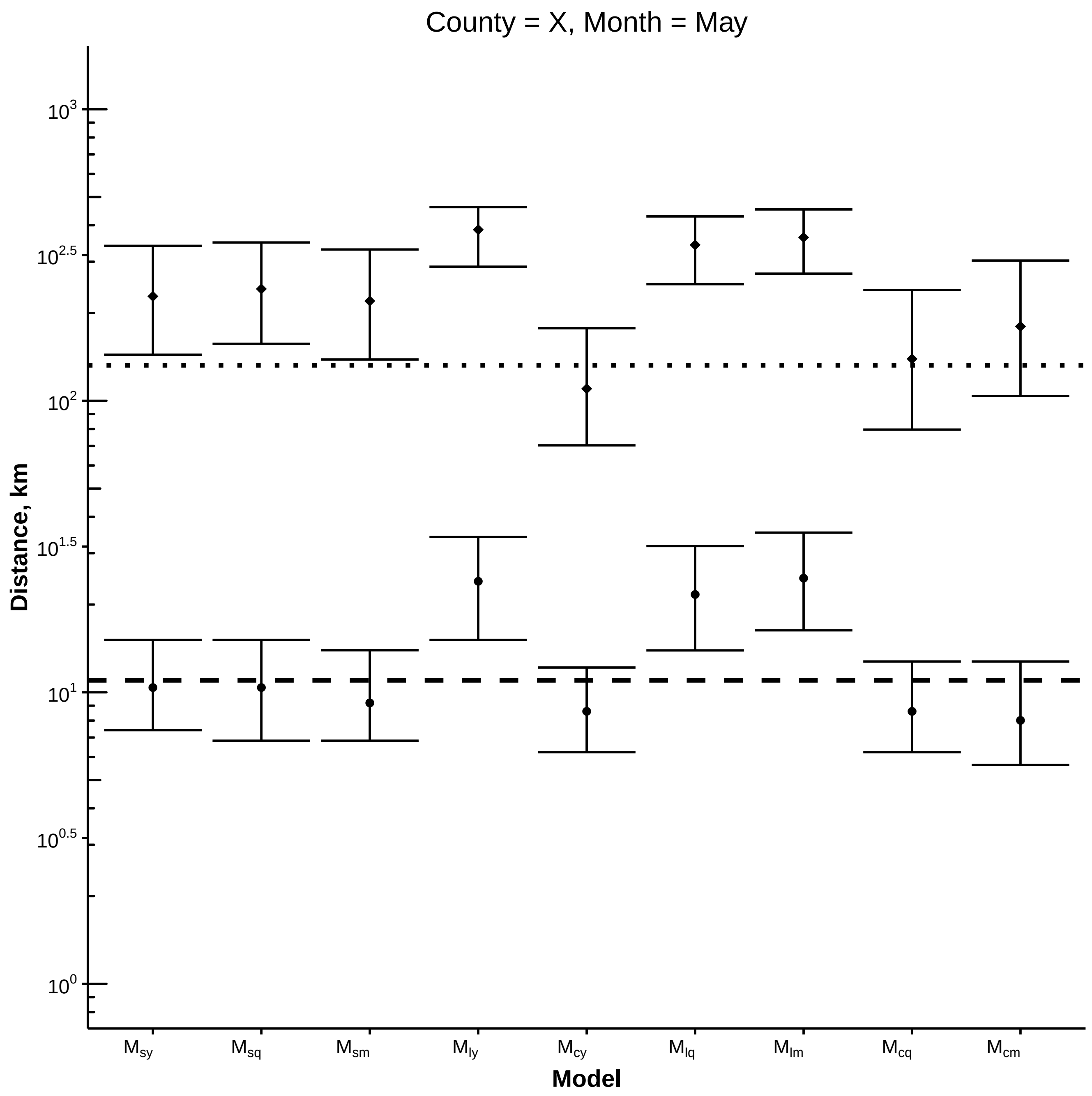

County = X, Month = May
Distance, km
10^3
$10^{2.5}$
10^2
$10^{1.5}$
10^1
$10^{0.5}$
10^0
M_{sy}
M_{sq}
M_{sm}
M_{ly}
M_{cy}
M_{lq}
M_{lm}
M_{cq}
M_{cm}
Model

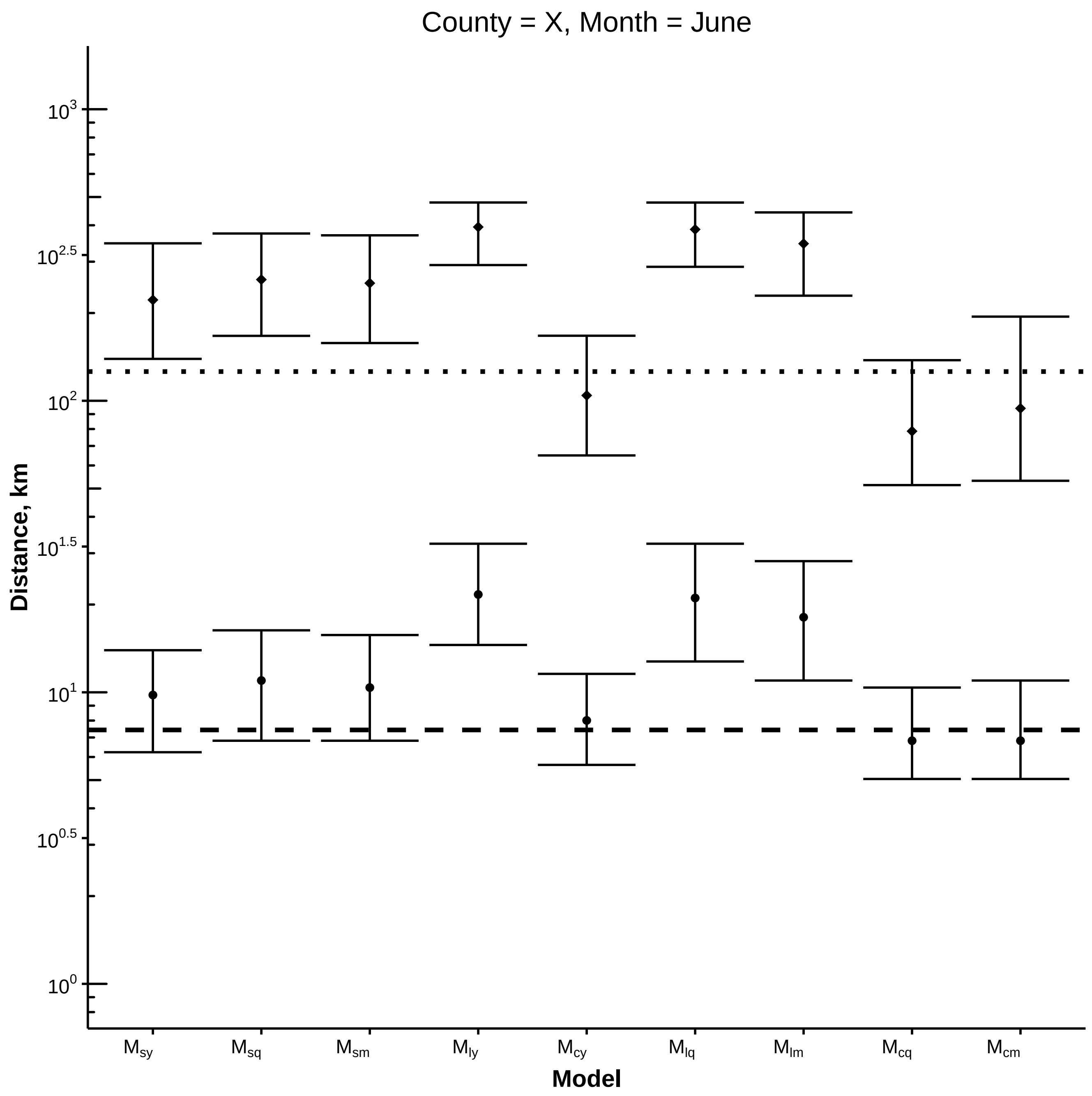

County = X, Month = June
Distance, km
10^3
$10^{2.5}$
10^2
$10^{1.5}$
10^1
$10^{0.5}$
10^0
M_{sy}
M_{sq}
M_{sm}
M_{ly}
M_{cy}
M_{lq}
M_{lm}
M_{cq}
M_{cm}
Model

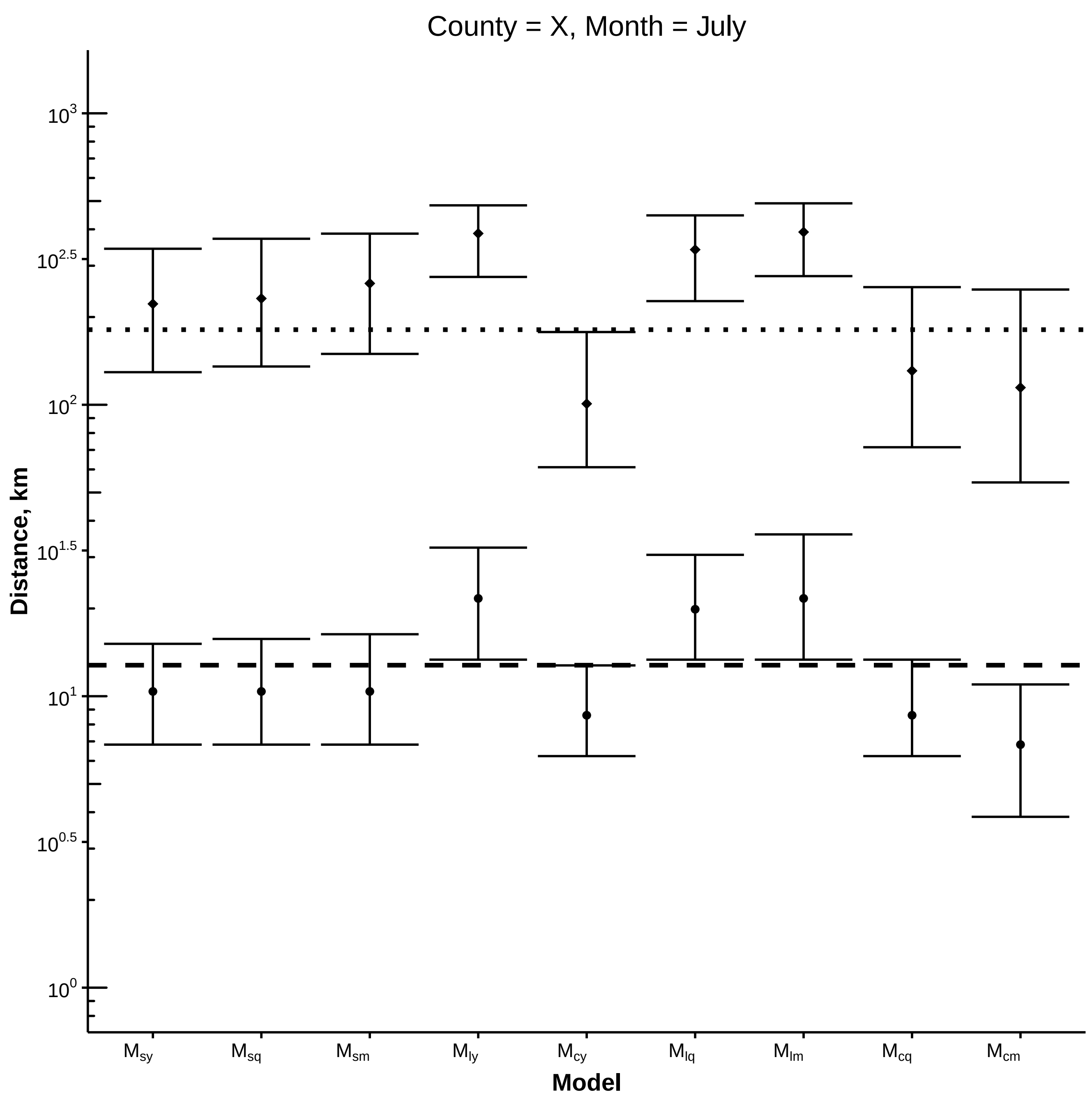

County = X, Month = July
Distance, km
10^3
$10^{2.5}$
10^2
$10^{1.5}$
10^1
$10^{0.5}$
10^0
M_{sy}
M_{sq}
M_{sm}
M_{ly}
M_{cy}
M_{lq}
M_{lm}
M_{cq}
M_{cm}
Model

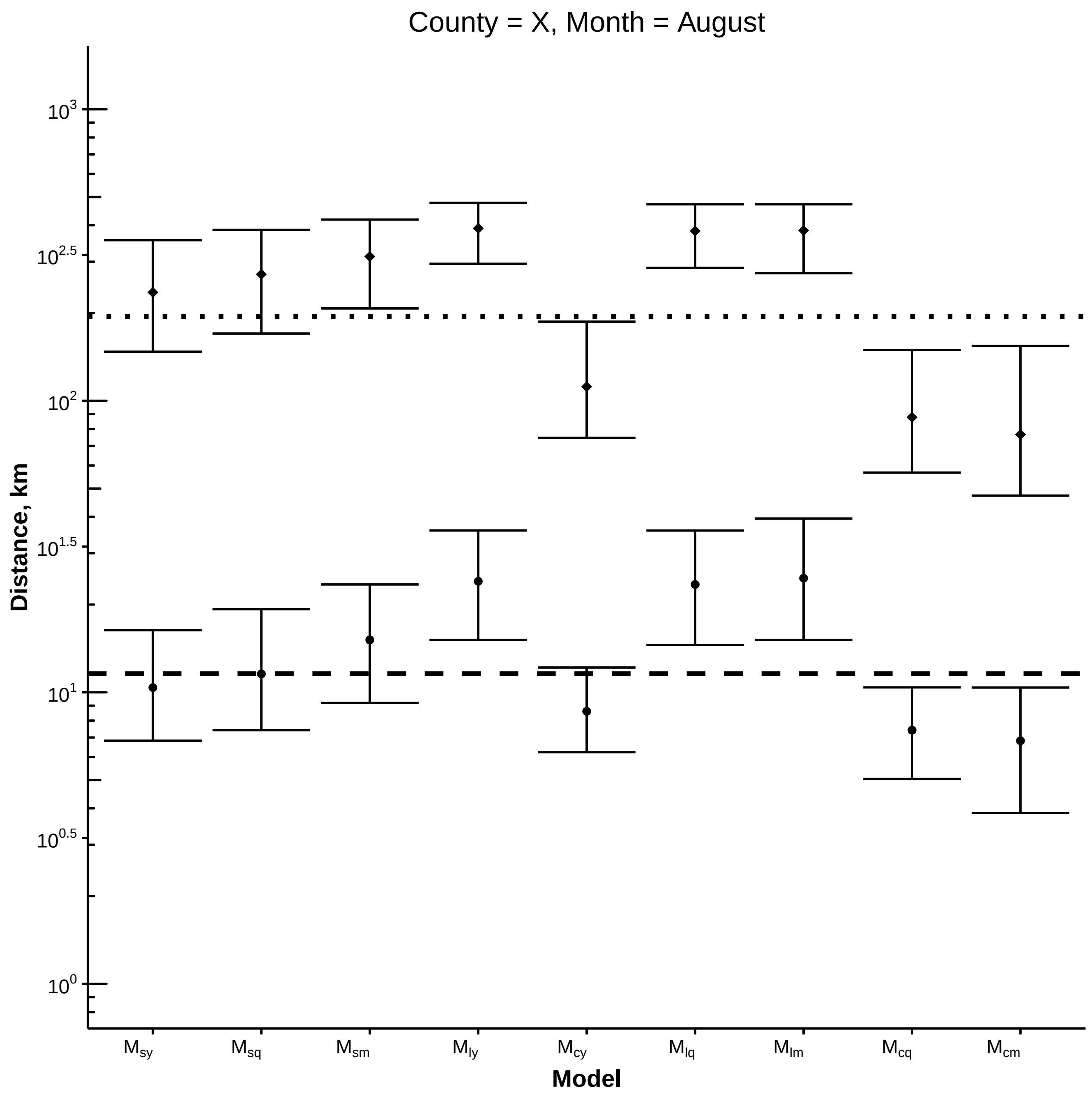

County = X, Month = August
Distance, km
10^3
$10^{2.5}$
10^2
$10^{1.5}$
10^1
$10^{0.5}$
10^0
M_{sy}
M_{sq}
M_{sm}
M_{ly}
M_{cy}
M_{lq}
M_{lm}
M_{cq}
M_{cm}
Model

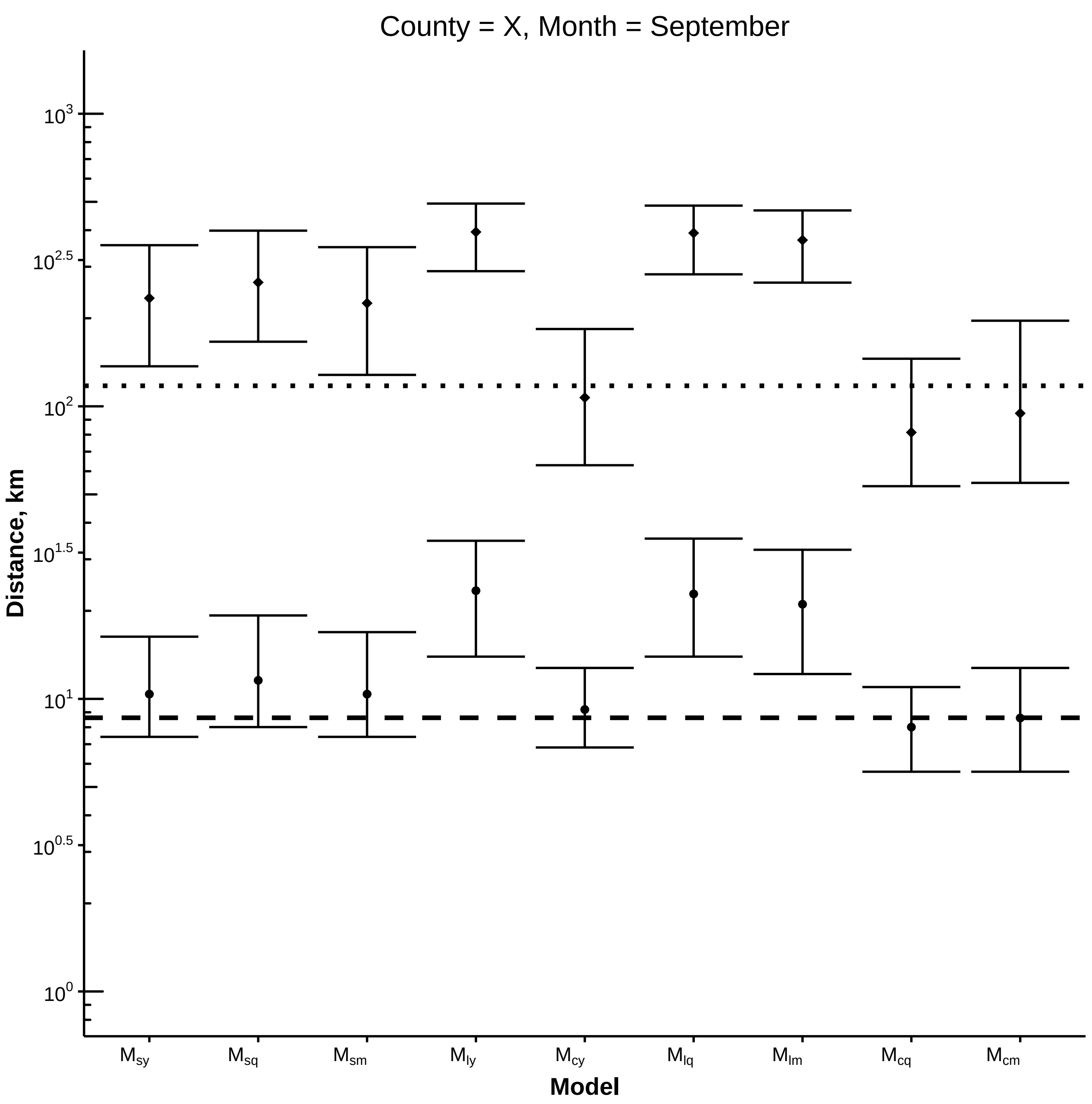

County = X, Month = September
Distance, km
10^3
10^2.5
10^2
10^1.5
10^1
10^0.5
10^0
M_sy
M_sq
M_sm
M_ly
M_cy
M_lq
M_lm
M_cq
M_cm
Model

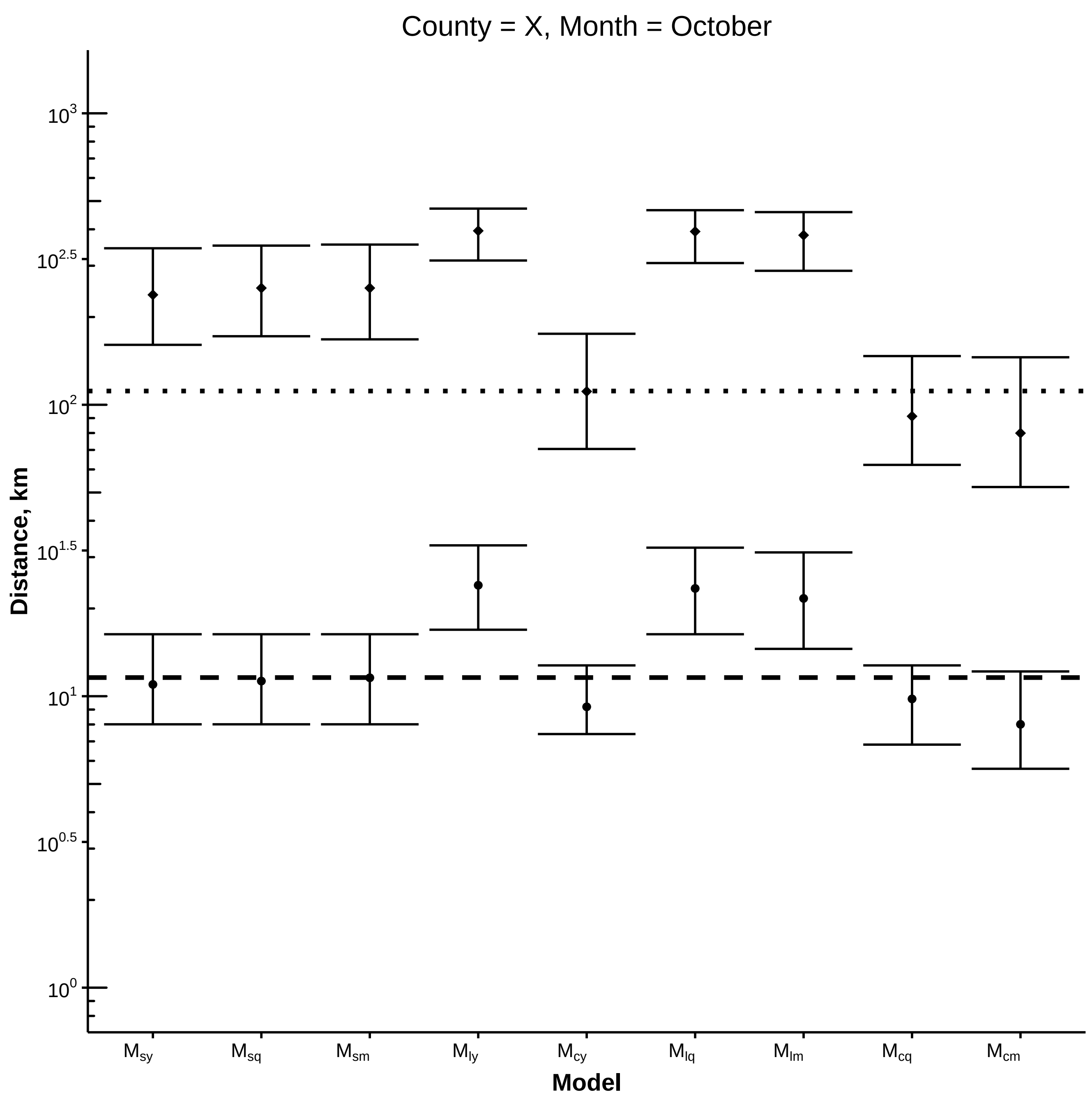
County = X, Month = October
Distance, km
10^3
$10^{2.5}$
10^2
$10^{1.5}$
10^1
$10^{0.5}$
10^0
M_{sy}
M_{sq}
M_{sm}
M_{ly}
M_{cy}
M_{lq}
M_{lm}
M_{cq}
M_{cm}
Model

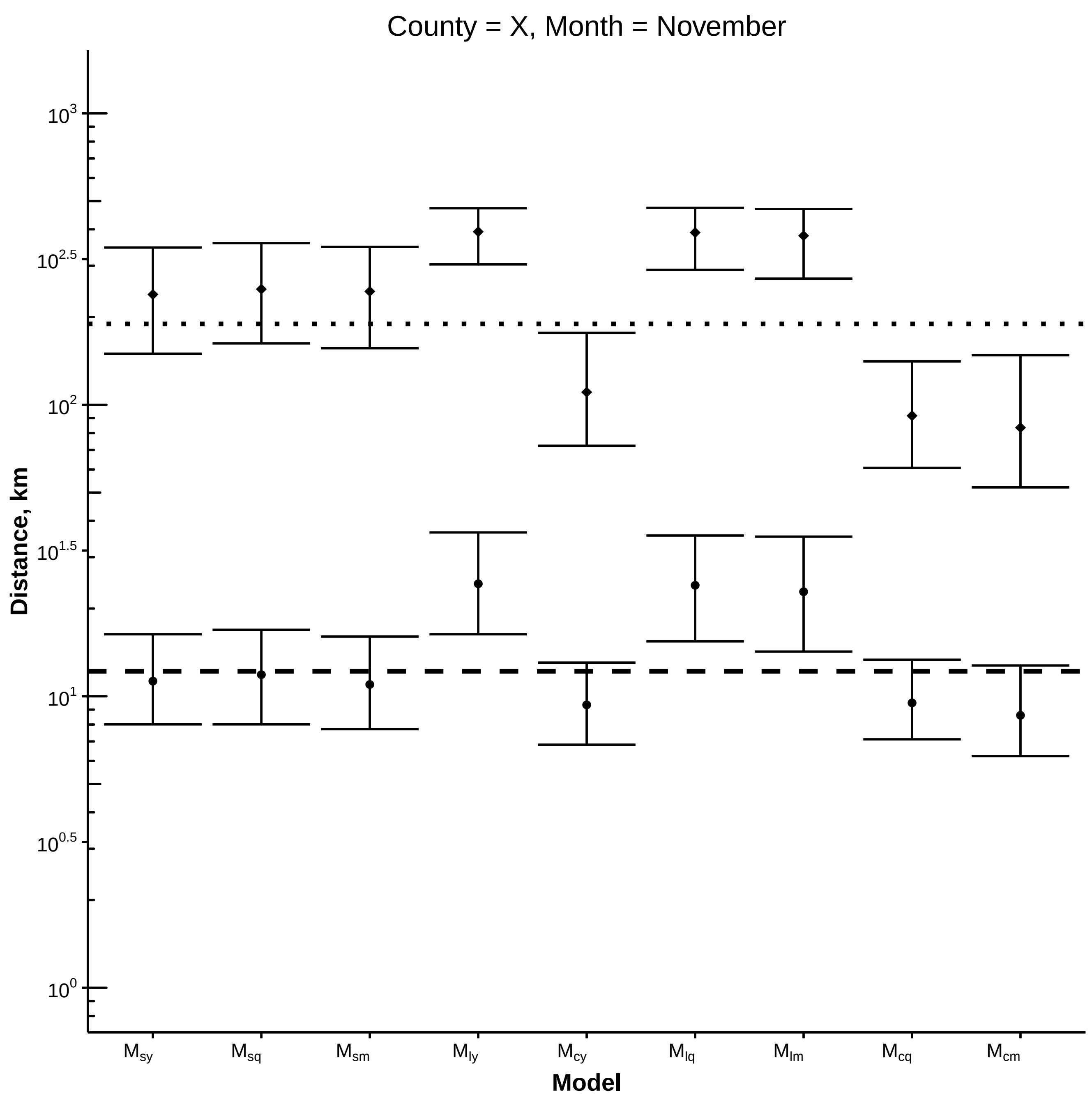

County = X, Month = November
Distance, km
Model
10^3
$10^{2.5}$
10^2
$10^{1.5}$
10^1
$10^{0.5}$
10^0
M_{sy}
M_{sq}
M_{sm}
M_{ly}
M_{cy}
M_{lq}
M_{lm}
M_{cq}
M_{cm}

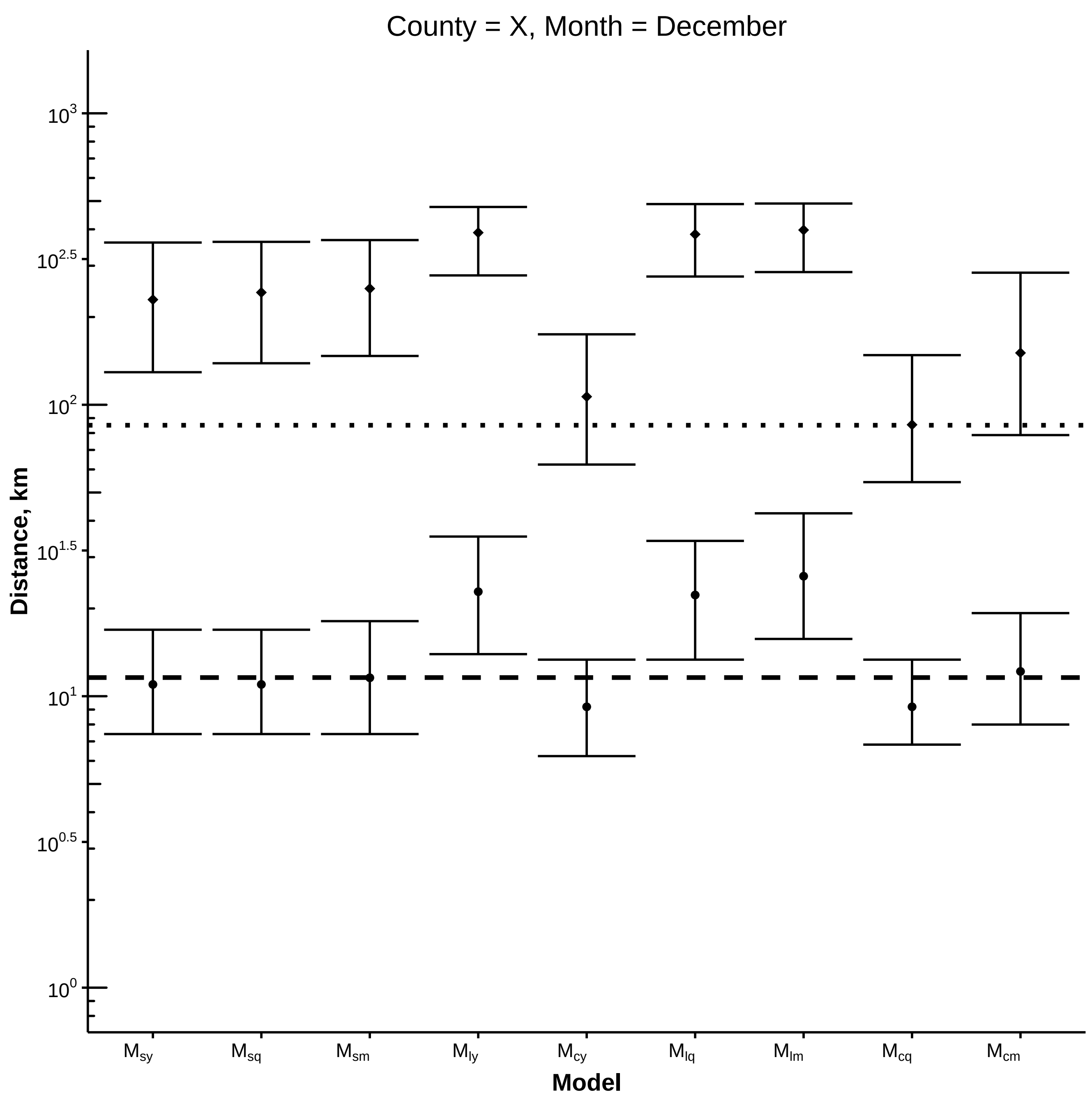
County = X, Month = December
Distance, km
10^3
$10^{2.5}$
10^2
$10^{1.5}$
10^1
$10^{0.5}$
10^0
M_{sy}
M_{sq}
M_{sm}
M_{ly}
M_{cy}
M_{lq}
M_{lm}
M_{cq}
M_{cm}
Model

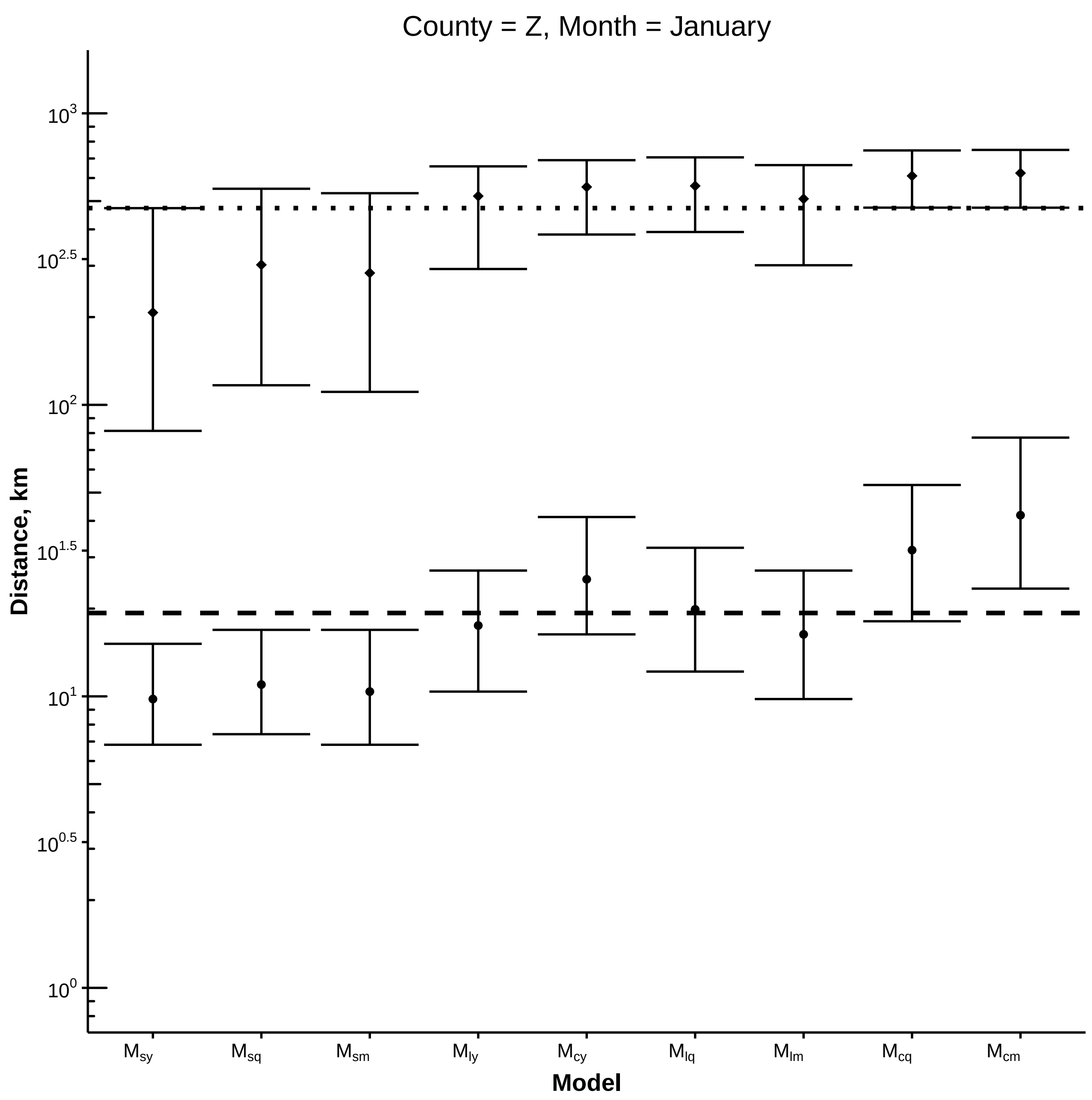
County = Z, Month = January
Distance, km
10^3
$10^{2.5}$
10^2
$10^{1.5}$
10^1
$10^{0.5}$
10^0
M_{sy}
M_{sq}
M_{sm}
M_{ly}
M_{cy}
M_{lq}
M_{lm}
M_{cq}
M_{cm}
Model

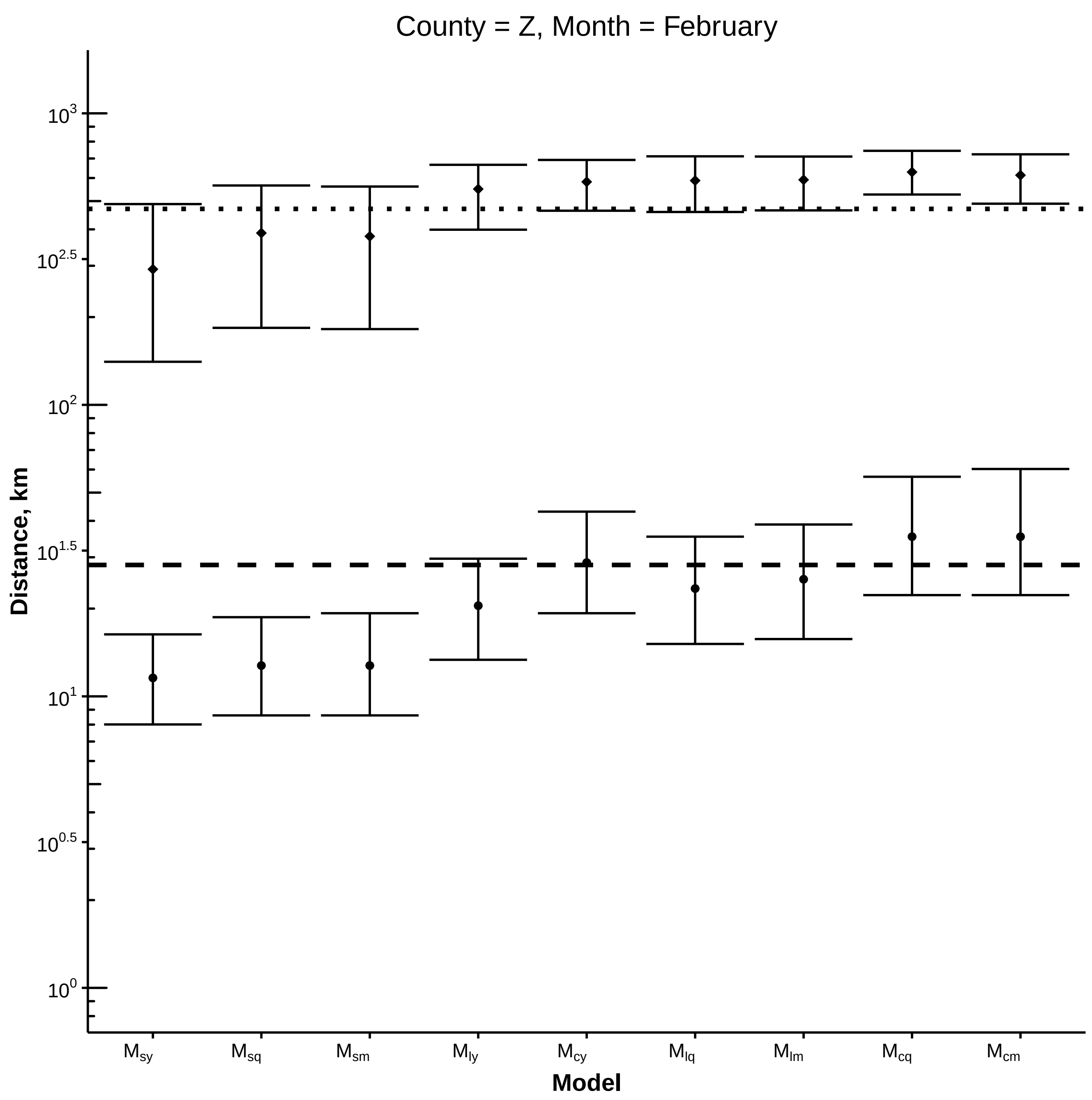
County = Z, Month = February
Distance, km
Model
10^3
$10^{2.5}$
10^2
$10^{1.5}$
10^1
$10^{0.5}$
10^0
M_{sy}
M_{sq}
M_{sm}
M_{ly}
M_{cy}
M_{lq}
M_{lm}
M_{cq}
M_{cm}

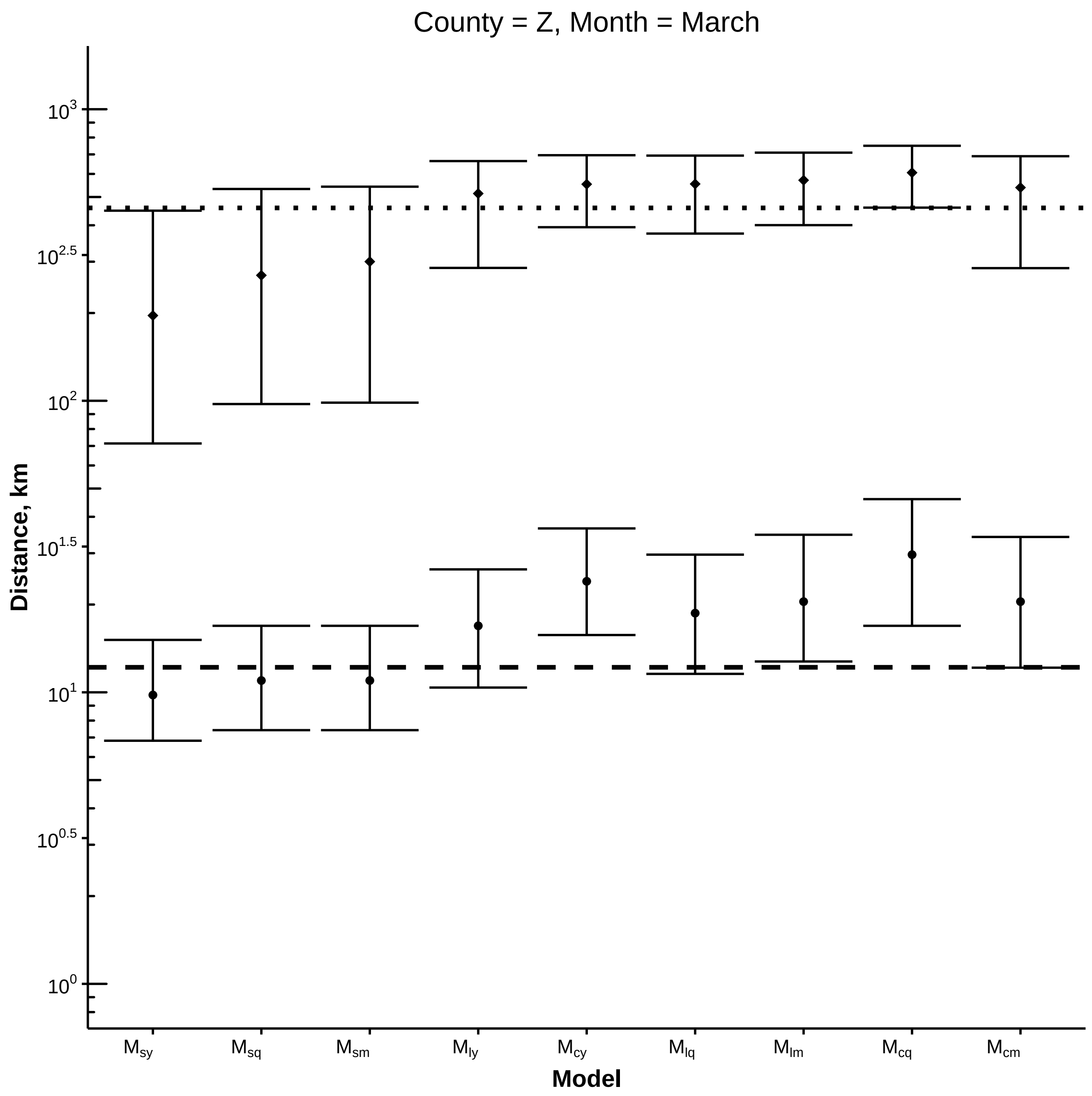
County = Z, Month = March
Distance, km
10^3
$10^{2.5}$
10^2
$10^{1.5}$
10^1
$10^{0.5}$
10^0
M_{sy}
M_{sq}
M_{sm}
M_{ly}
M_{cy}
M_{lq}
M_{lm}
M_{cq}
M_{cm}
Model

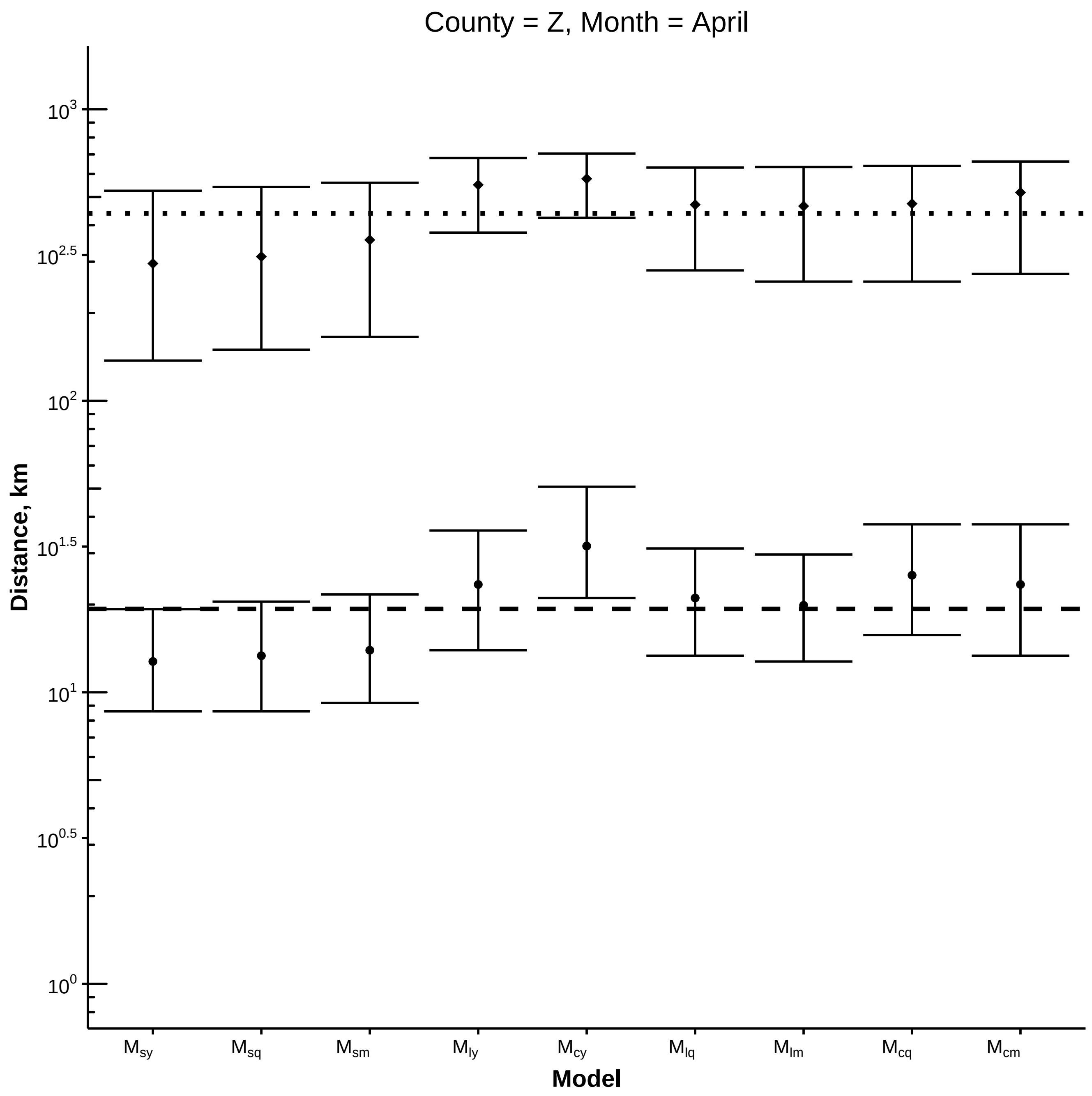
County = Z, Month = April
Distance, km
10^3
$10^{2.5}$
10^2
$10^{1.5}$
10^1
$10^{0.5}$
10^0
M_{sy}
M_{sq}
M_{sm}
M_{ly}
M_{cy}
M_{lq}
M_{lm}
M_{cq}
M_{cm}
Model

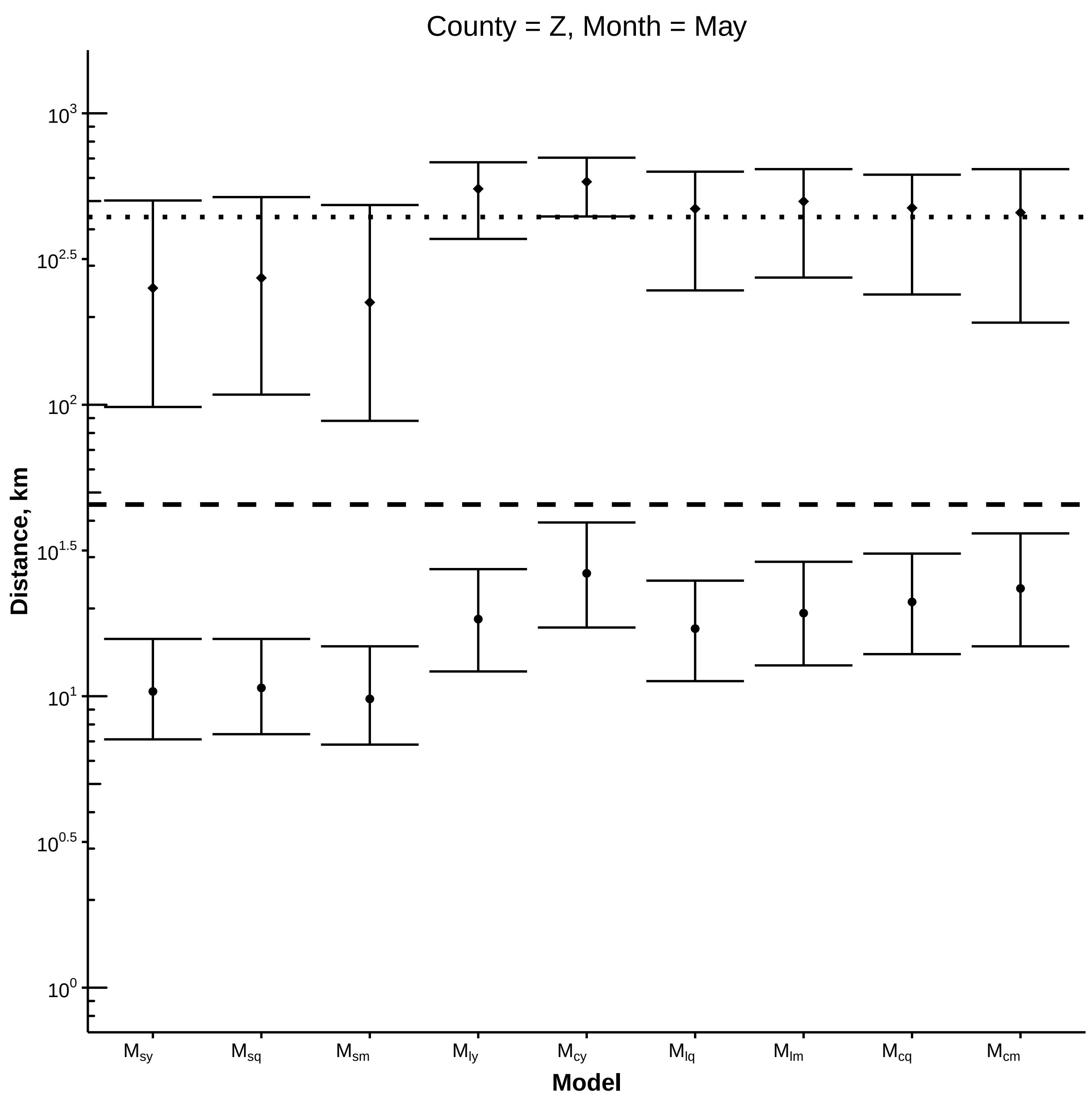

County = Z, Month = May
Distance, km
10^3
$10^{2.5}$
10^2
$10^{1.5}$
10^1
$10^{0.5}$
10^0
M_{sy}
M_{sq}
M_{sm}
M_{ly}
M_{cy}
M_{lq}
M_{lm}
M_{cq}
M_{cm}
Model

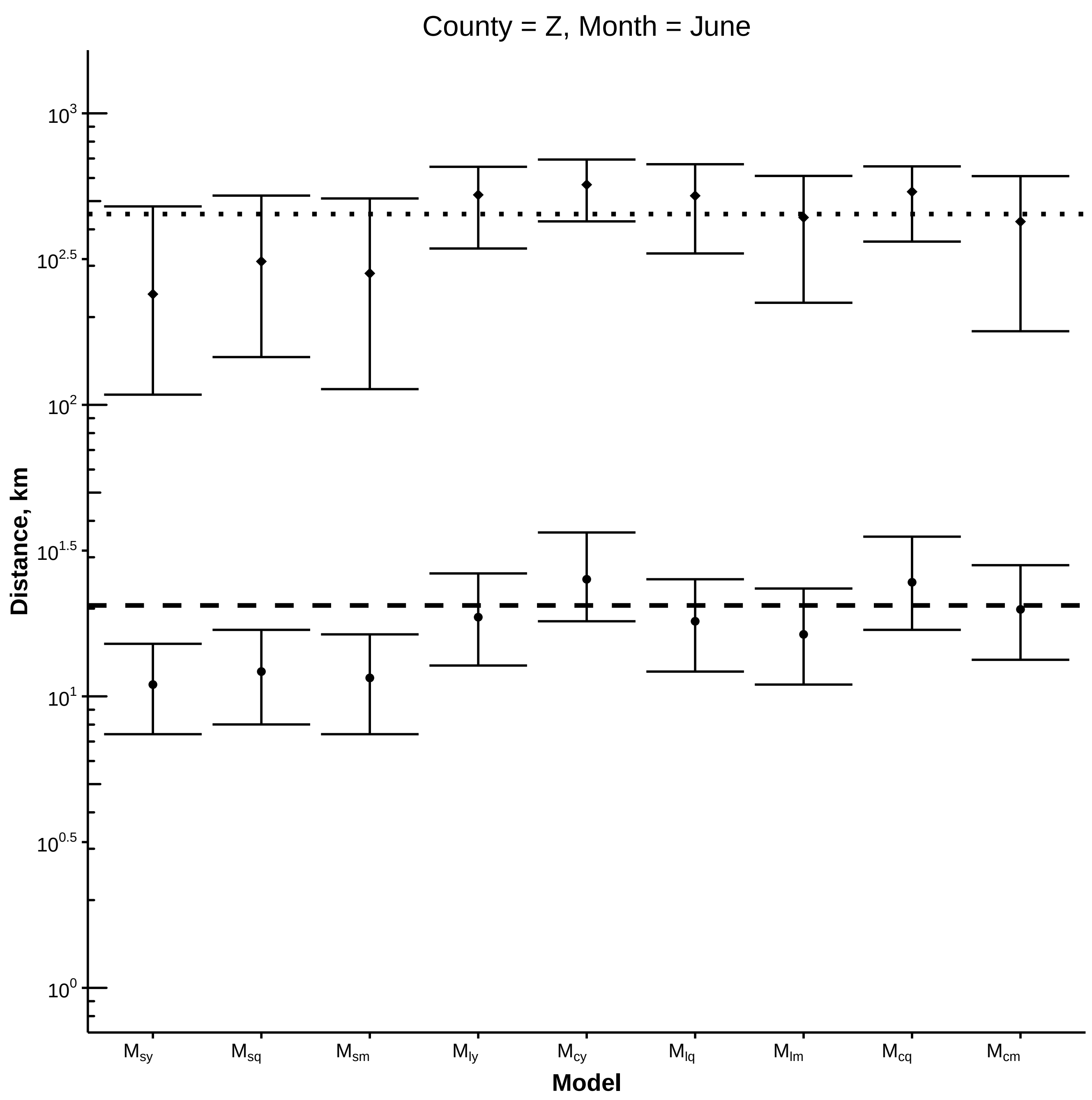
County = Z, Month = June
Distance, km
10^3
$10^{2.5}$
10^2
$10^{1.5}$
10^1
$10^{0.5}$
10^0
Model
M_{sy}
M_{sq}
M_{sm}
M_{ly}
M_{cy}
M_{lq}
M_{lm}
M_{cq}
M_{cm}

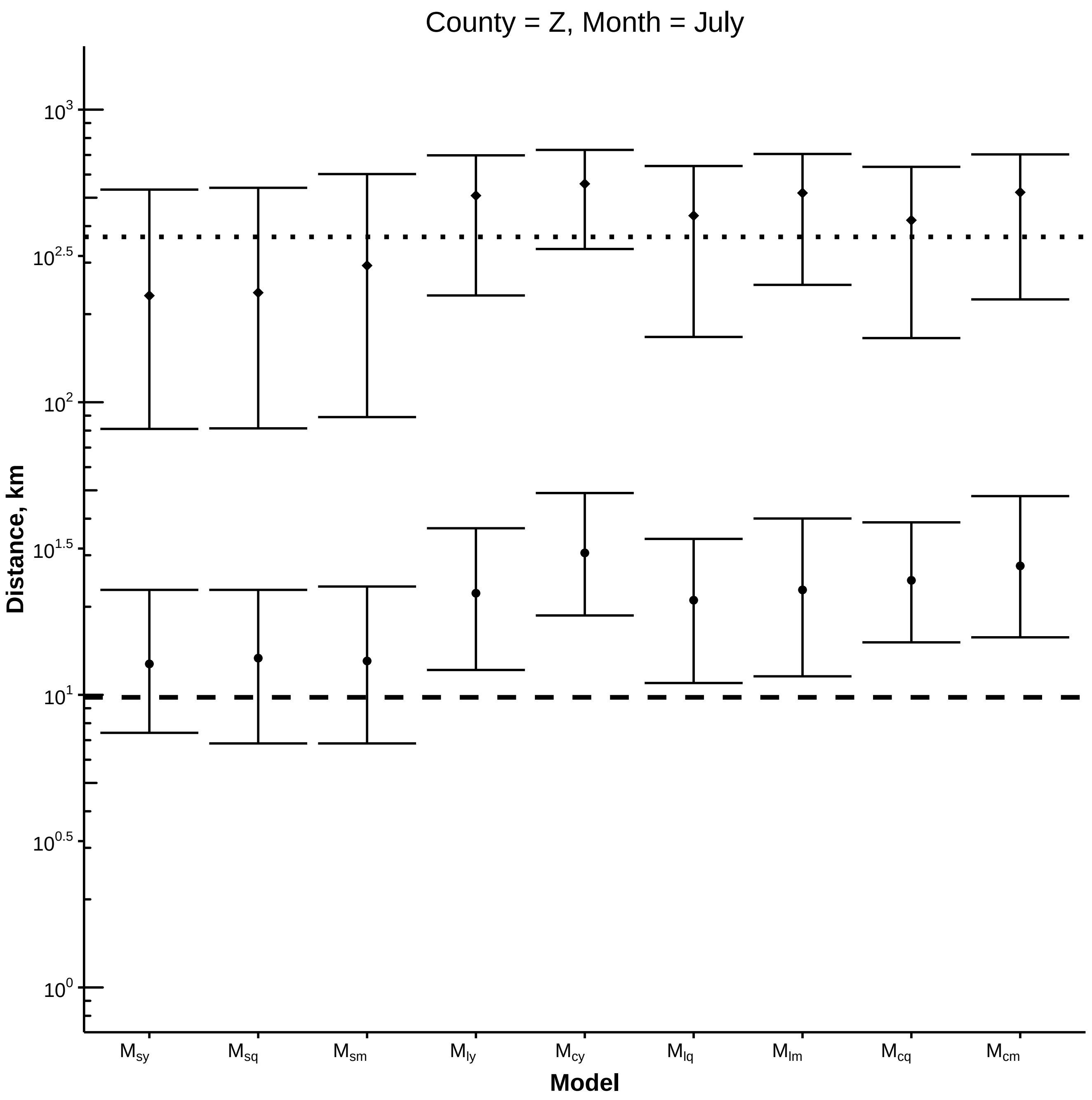
County = Z, Month = July
Distance, km
10^3
$10^{2.5}$
10^2
$10^{1.5}$
10^1
$10^{0.5}$
10^0
M_{sy}
M_{sq}
M_{sm}
M_{ly}
M_{cy}
M_{lq}
M_{lm}
M_{cq}
M_{cm}
Model

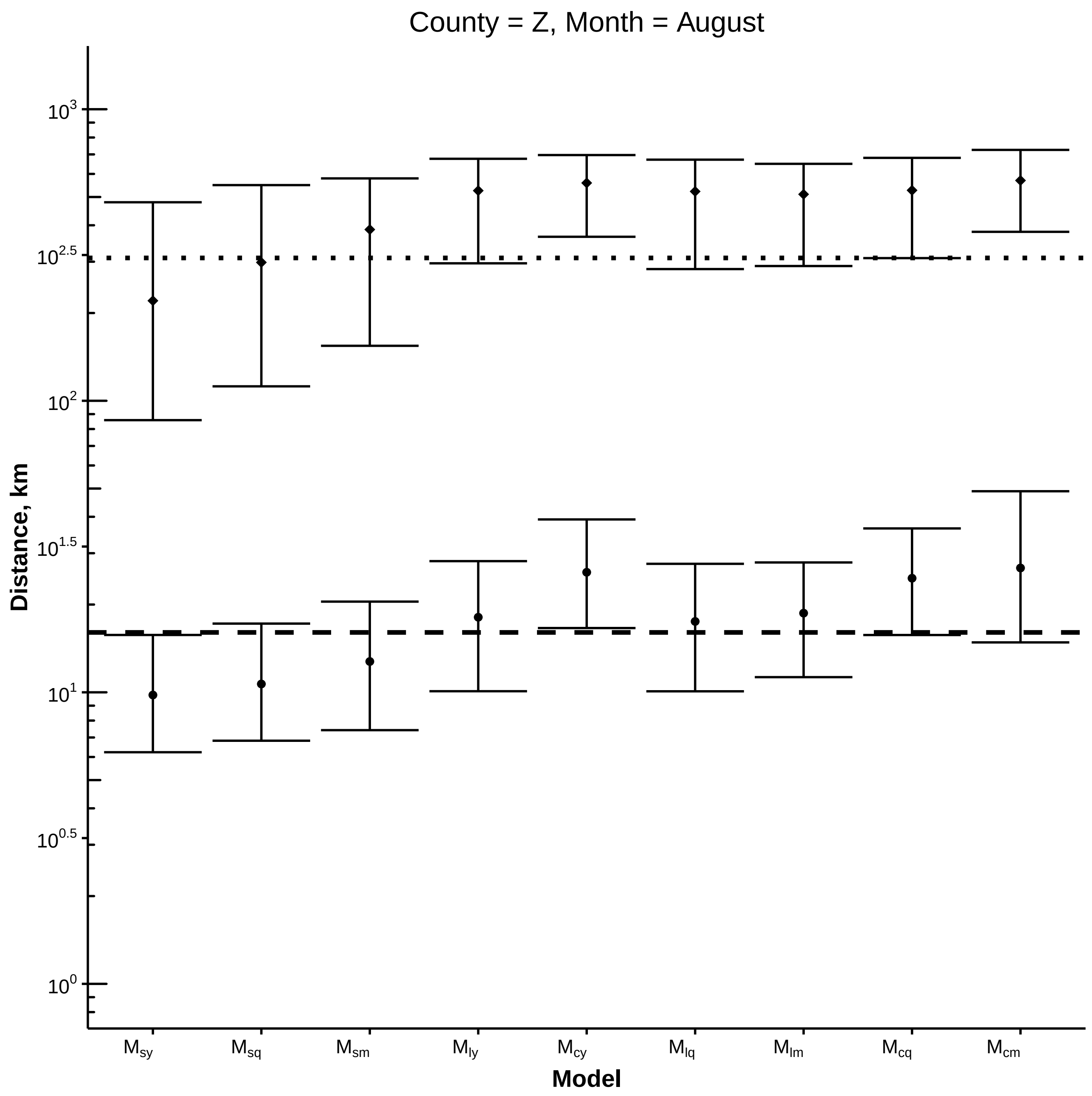
County = Z, Month = August
Distance, km
10^3
$10^{2.5}$
10^2
$10^{1.5}$
10^1
$10^{0.5}$
10^0
M_{sy}
M_{sq}
M_{sm}
M_{ly}
M_{cy}
M_{lq}
M_{lm}
M_{cq}
M_{cm}
Model

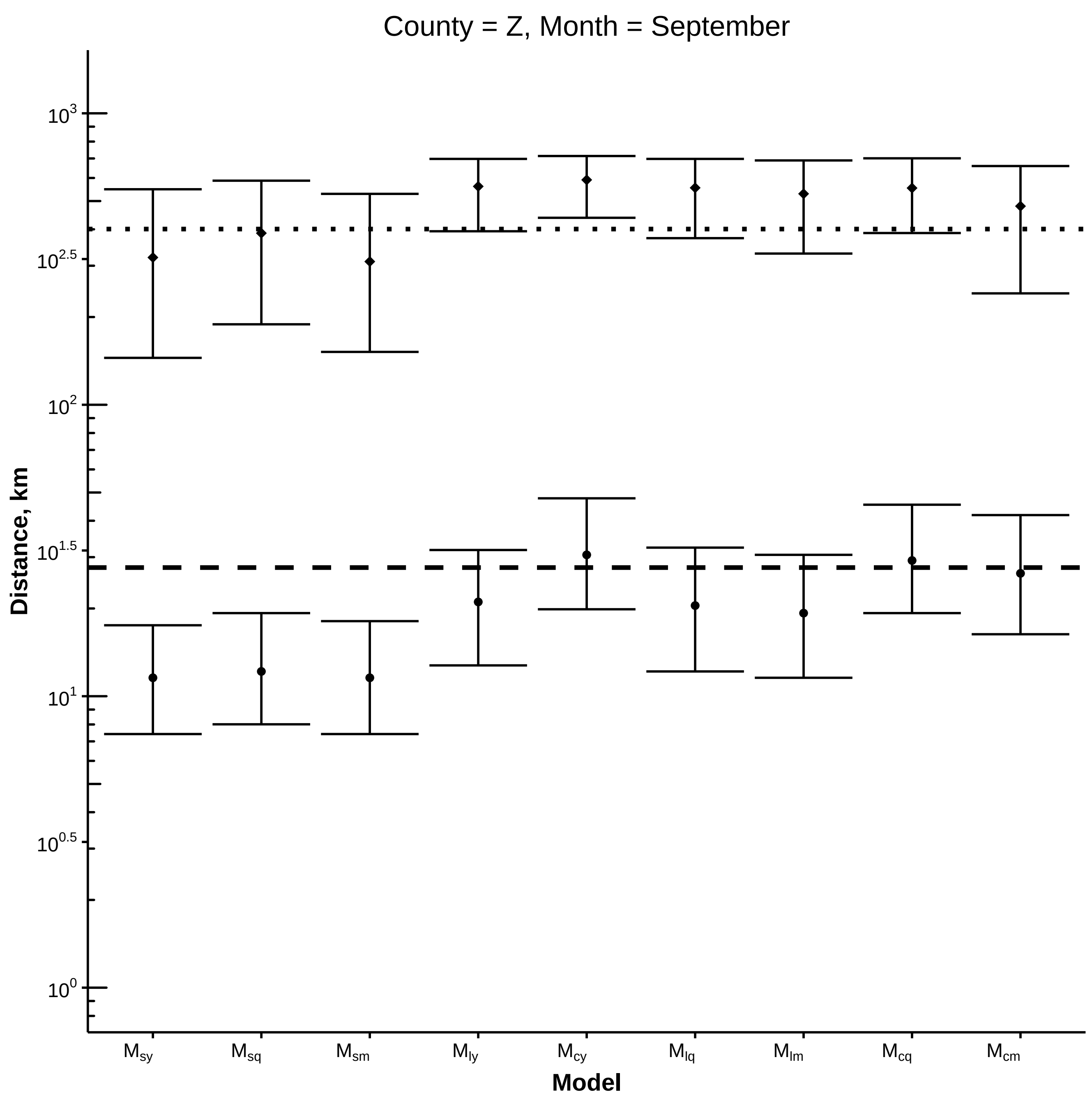

County = Z, Month = September
Distance, km
10^3
10^2.5
10^2
10^1.5
10^1
10^0.5
10^0
Msy
Msq
Msm
Mly
Mcy
Mlq
Mlm
Mcq
Mcm
Model

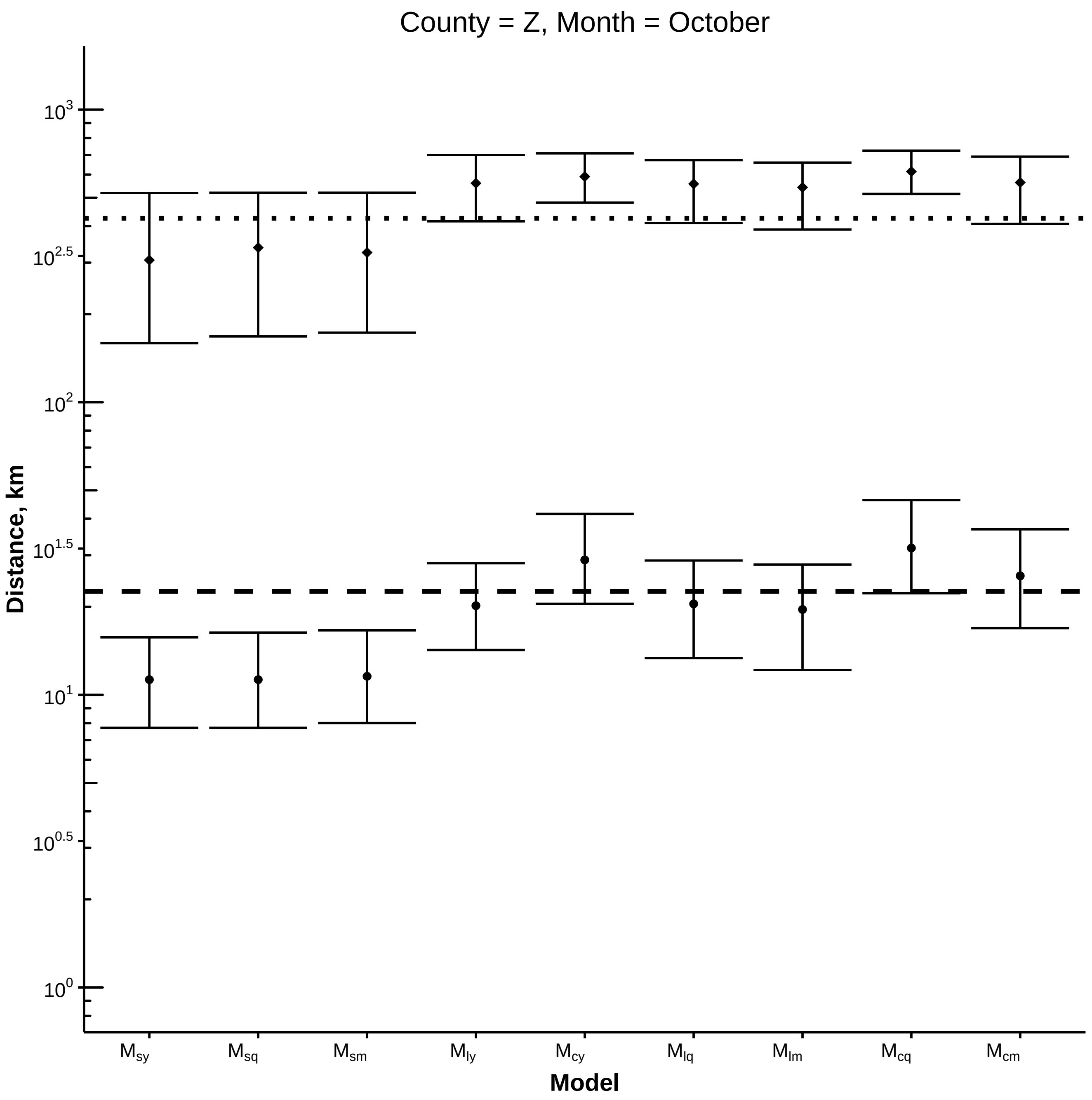

County = Z, Month = October
Distance, km
10^3
$10^{2.5}$
10^2
$10^{1.5}$
10^1
$10^{0.5}$
10^0
M_{sy}
M_{sq}
M_{sm}
M_{ly}
M_{cy}
M_{lq}
M_{lm}
M_{cq}
M_{cm}
Model

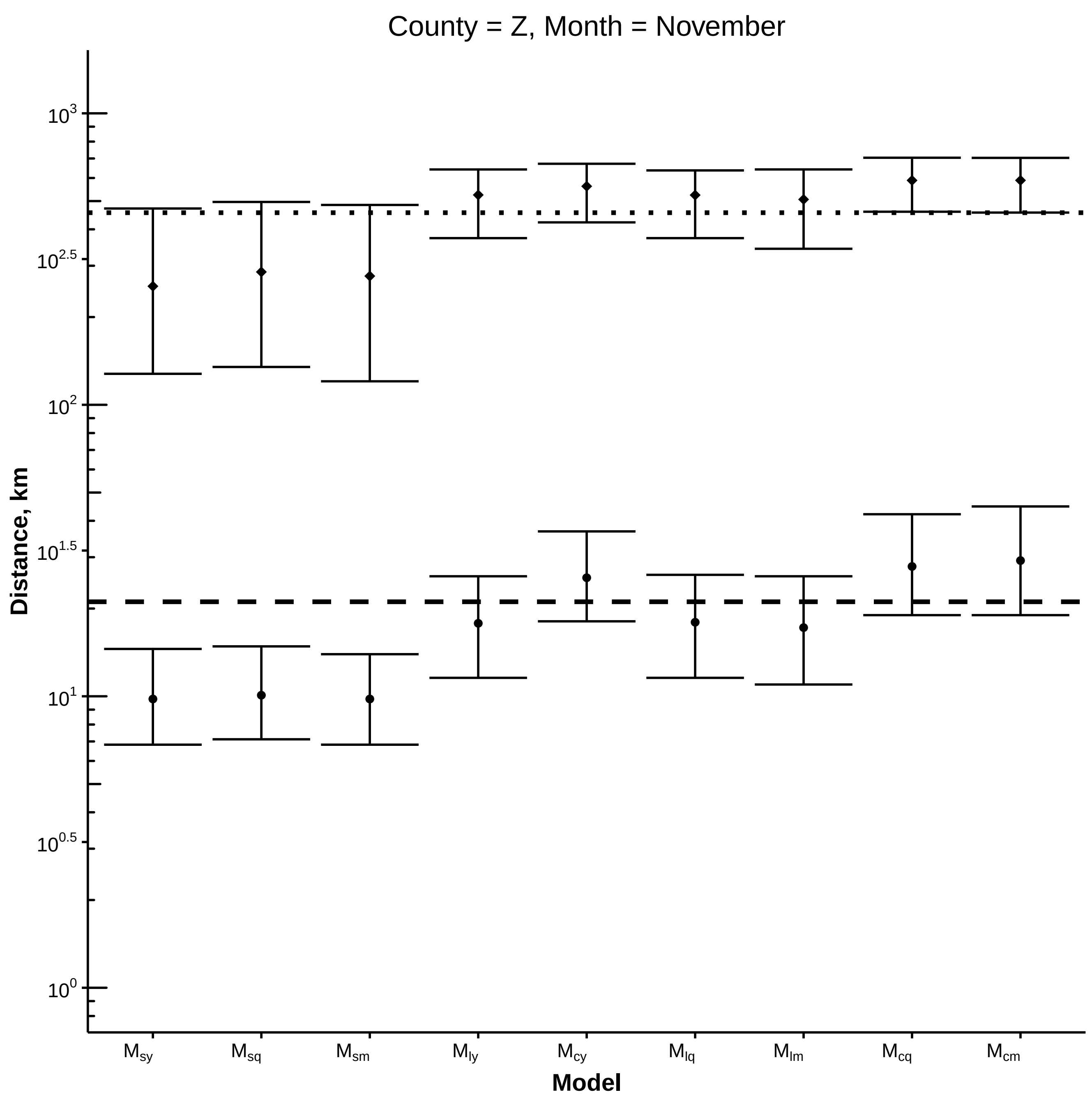

County = Z, Month = November
Distance, km
10^3
10^2.5
10^2
10^1.5
10^1
10^0.5
10^0
Msy
Msq
Msm
Mly
Mcy
Mlq
Mlm
Mcq
Mcm
Model

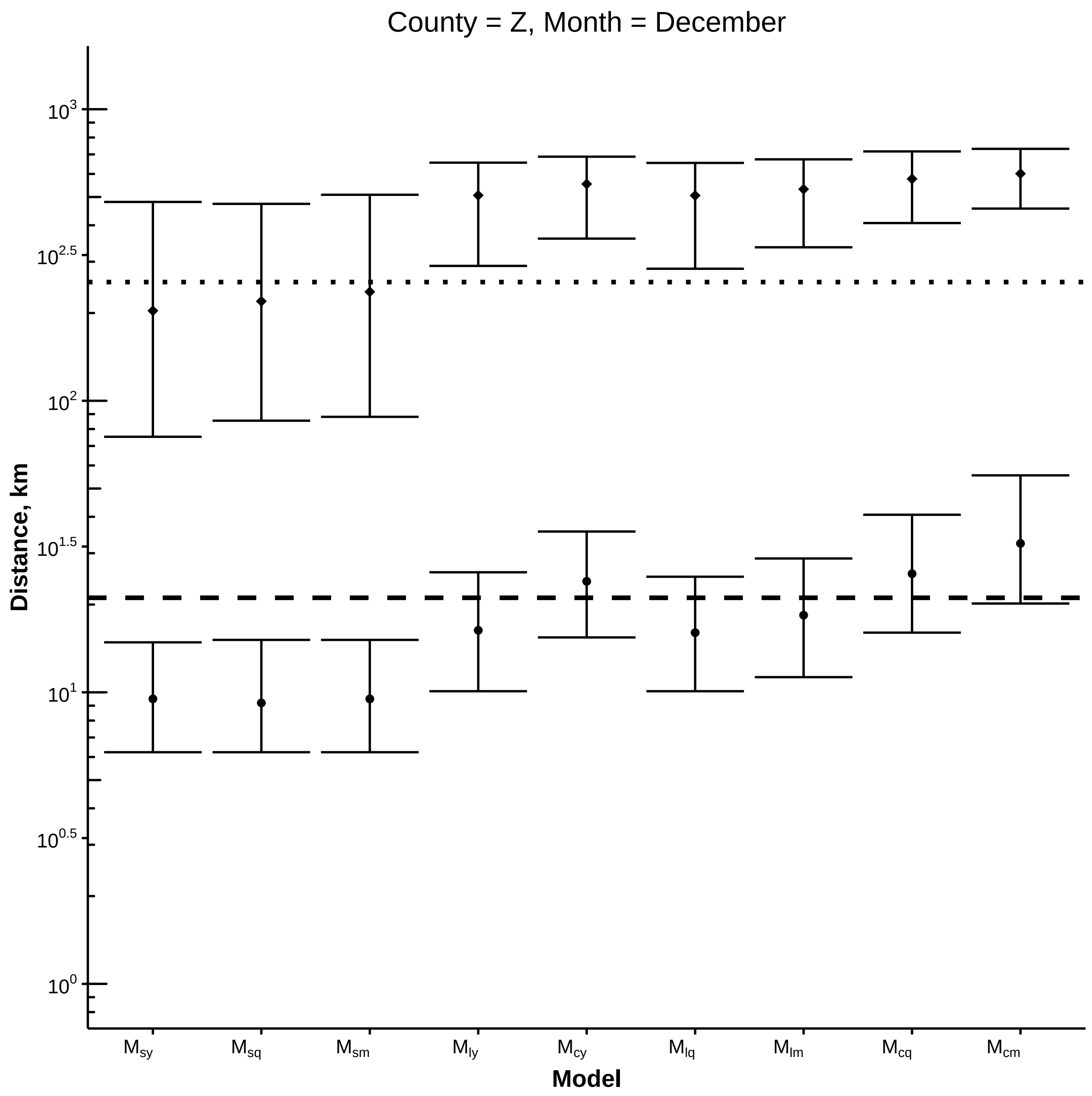

County = Z, Month = December
Distance, km
10^3
$10^{2.5}$
10^2
$10^{1.5}$
10^1
$10^{0.5}$
10^0
M_{sy}
M_{sq}
M_{sm}
M_{ly}
M_{cy}
M_{lq}
M_{lm}
M_{cq}
M_{cm}
Model

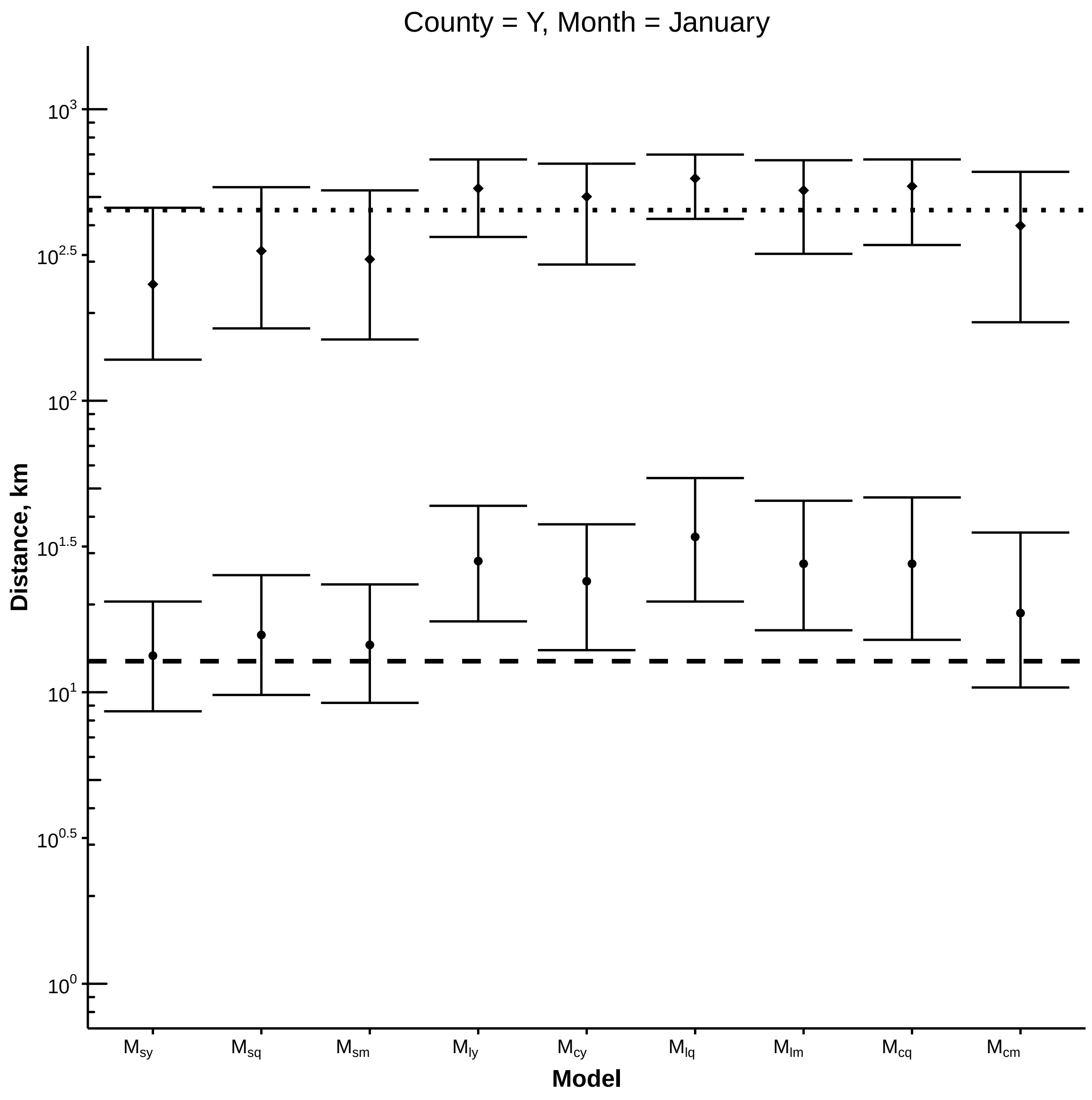

County = Y, Month = January
Distance, km
10^3
$10^{2.5}$
10^2
$10^{1.5}$
10^1
$10^{0.5}$
10^0
M_{sy}
M_{sq}
M_{sm}
M_{ly}
M_{cy}
M_{lq}
M_{lm}
M_{cq}
M_{cm}
Model

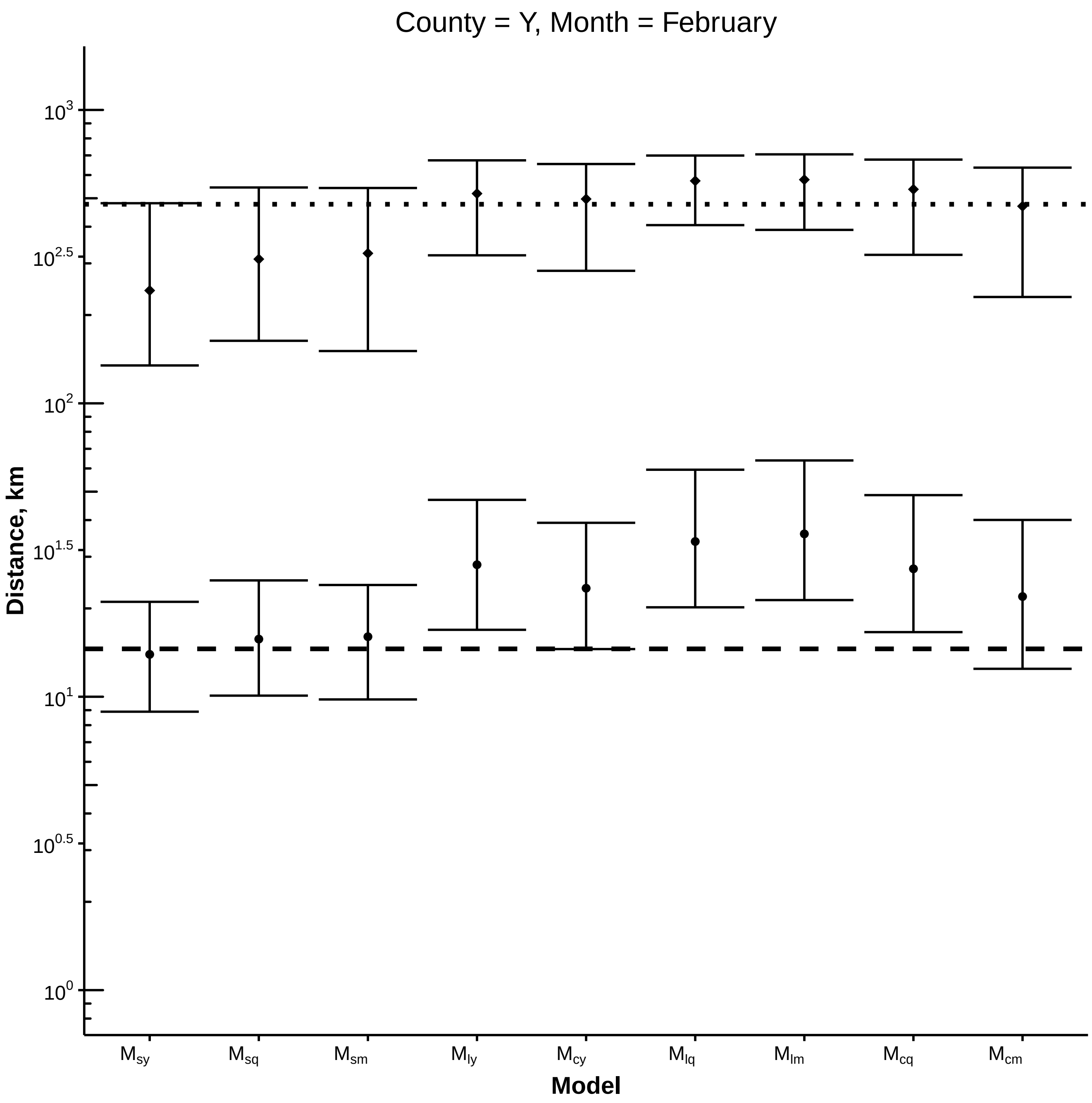

County = Y, Month = February
Distance, km
10^3
$10^{2.5}$
10^2
$10^{1.5}$
10^1
$10^{0.5}$
10^0
M_{sy}
M_{sq}
M_{sm}
M_{ly}
M_{cy}
M_{lq}
M_{lm}
M_{cq}
M_{cm}
Model

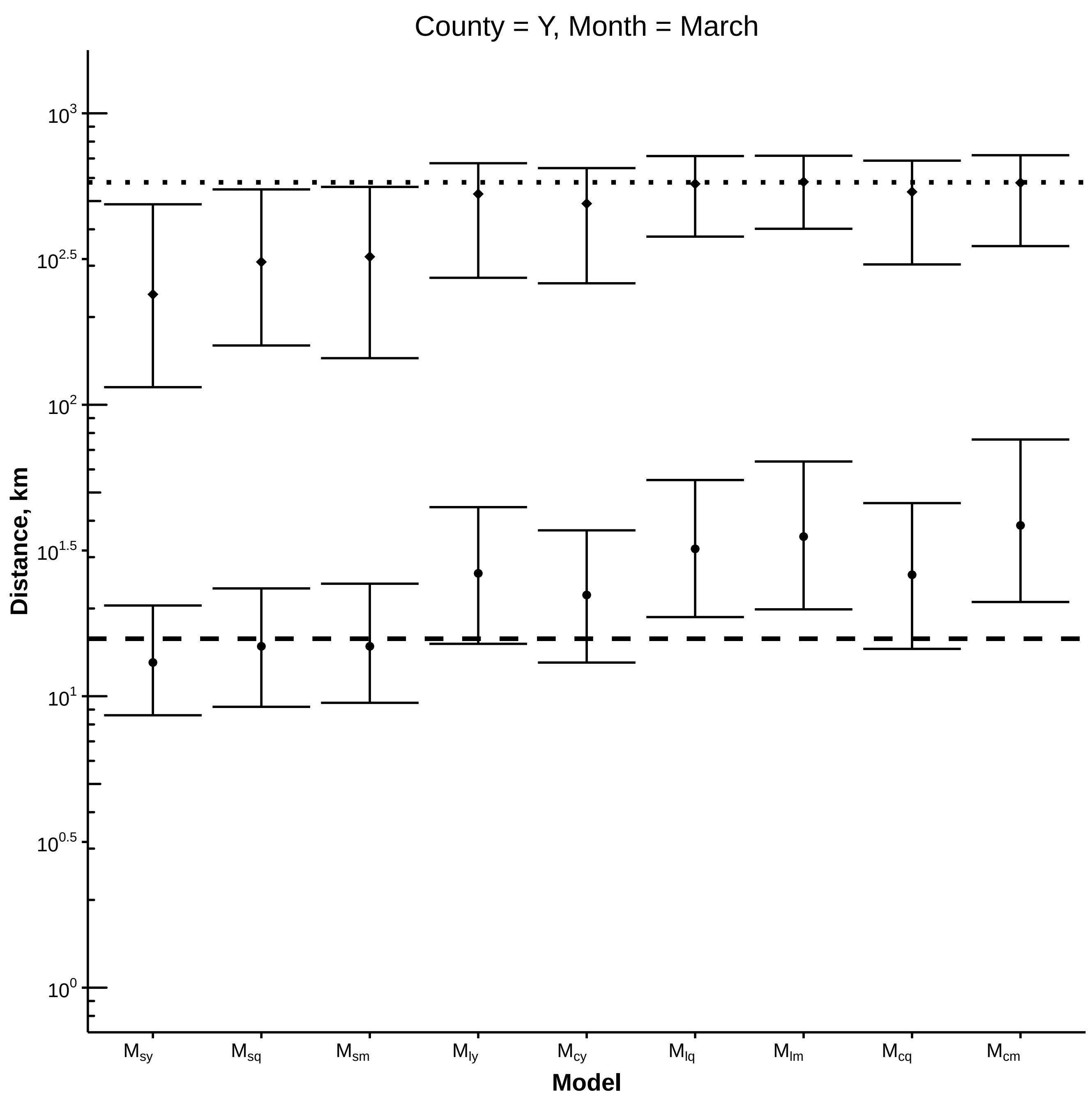

County = Y, Month = March
Distance, km
10^3
$10^{2.5}$
10^2
$10^{1.5}$
10^1
$10^{0.5}$
10^0
M_{sy}
M_{sq}
M_{sm}
M_{ly}
M_{cy}
M_{lq}
M_{lm}
M_{cq}
M_{cm}
Model

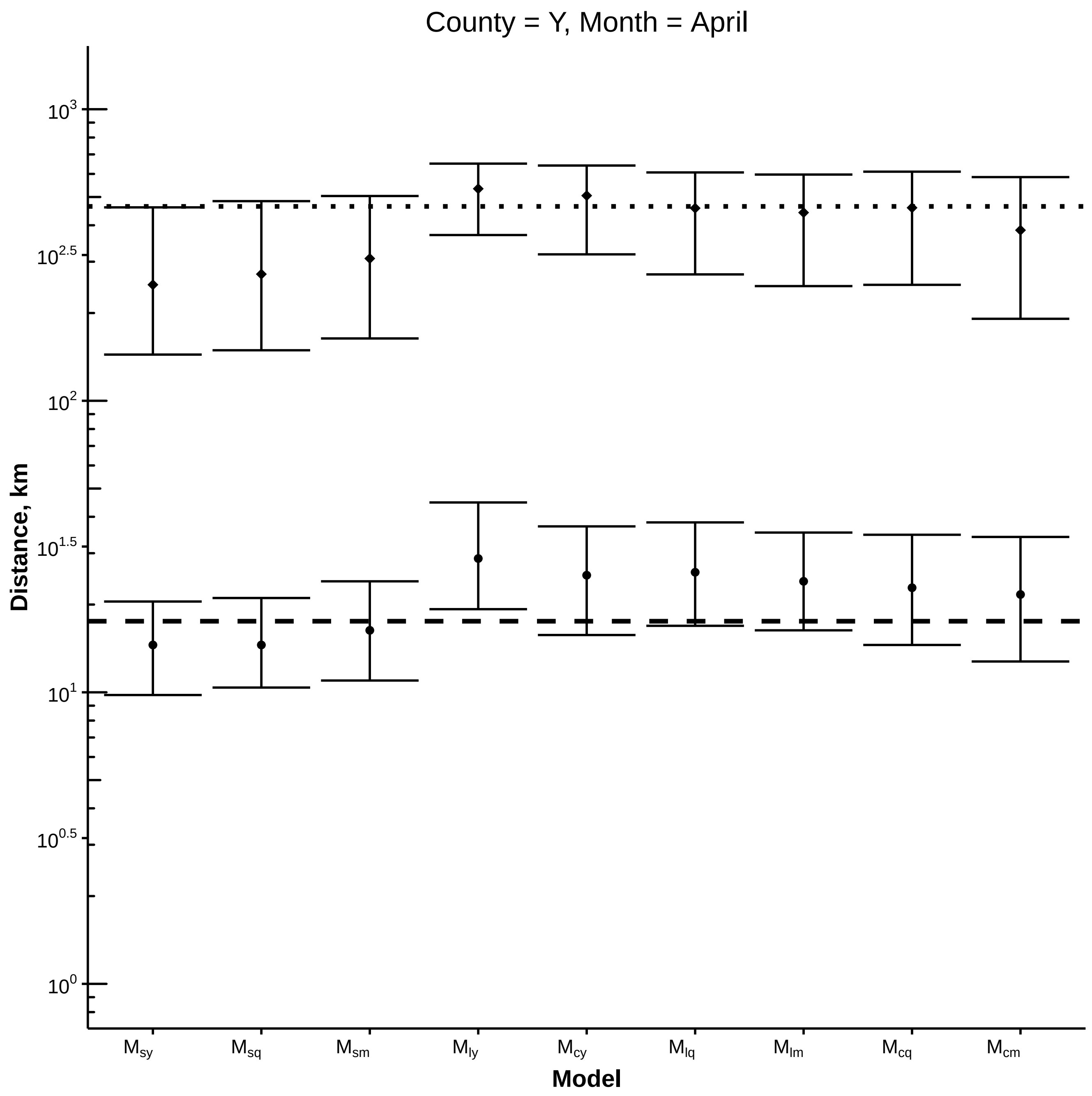

County = Y, Month = April
Distance, km
10^3
10^2.5
10^2
10^1.5
10^1
10^0.5
10^0
Msy
Msq
Msm
Mly
Mcy
Mlq
Mlm
Mcq
Mcm
Model

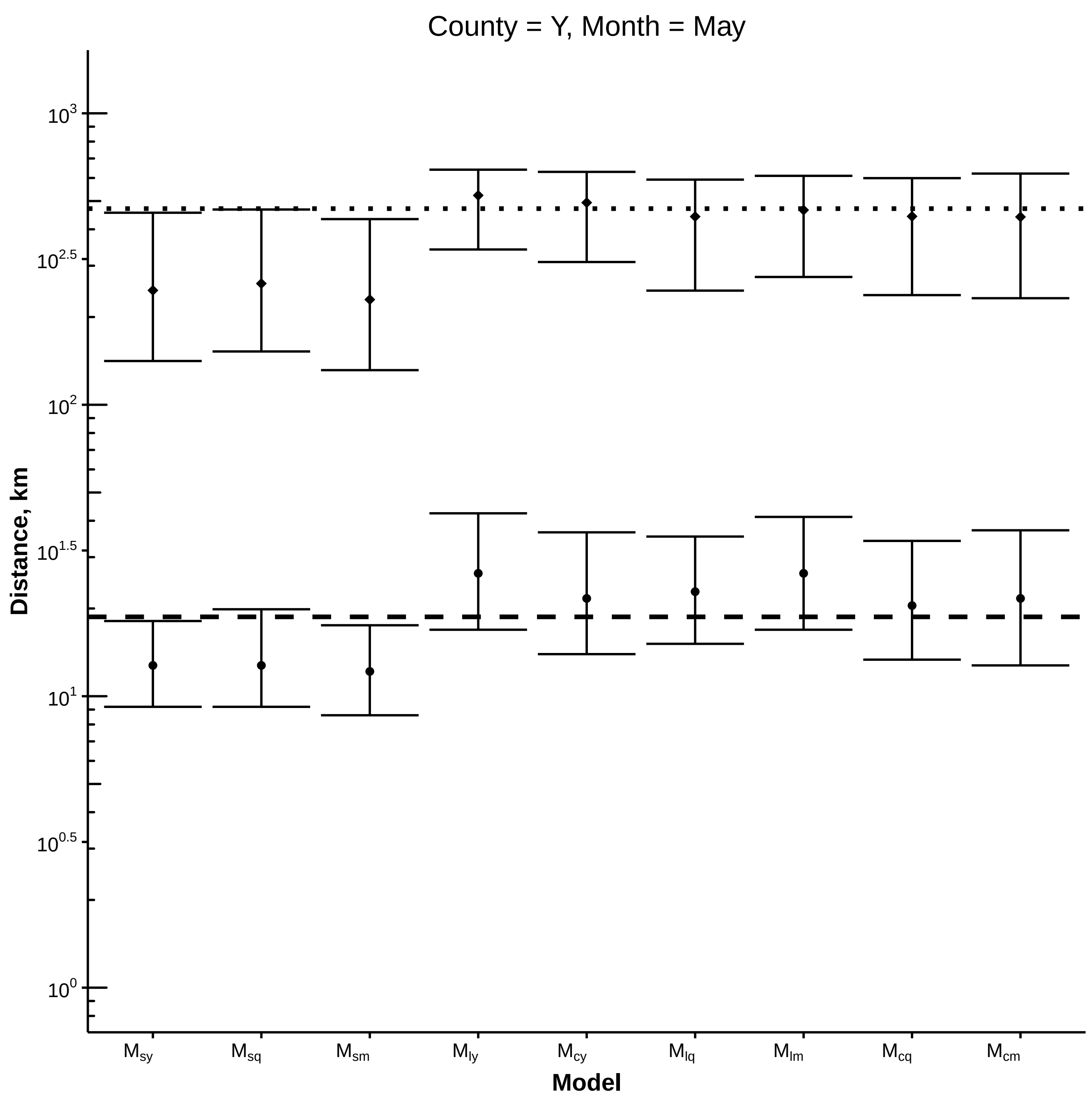

County = Y, Month = May
Distance, km
10^3
$10^{2.5}$
10^2
$10^{1.5}$
10^1
$10^{0.5}$
10^0
M_{sy}
M_{sq}
M_{sm}
M_{ly}
M_{cy}
M_{lq}
M_{lm}
M_{cq}
M_{cm}
Model

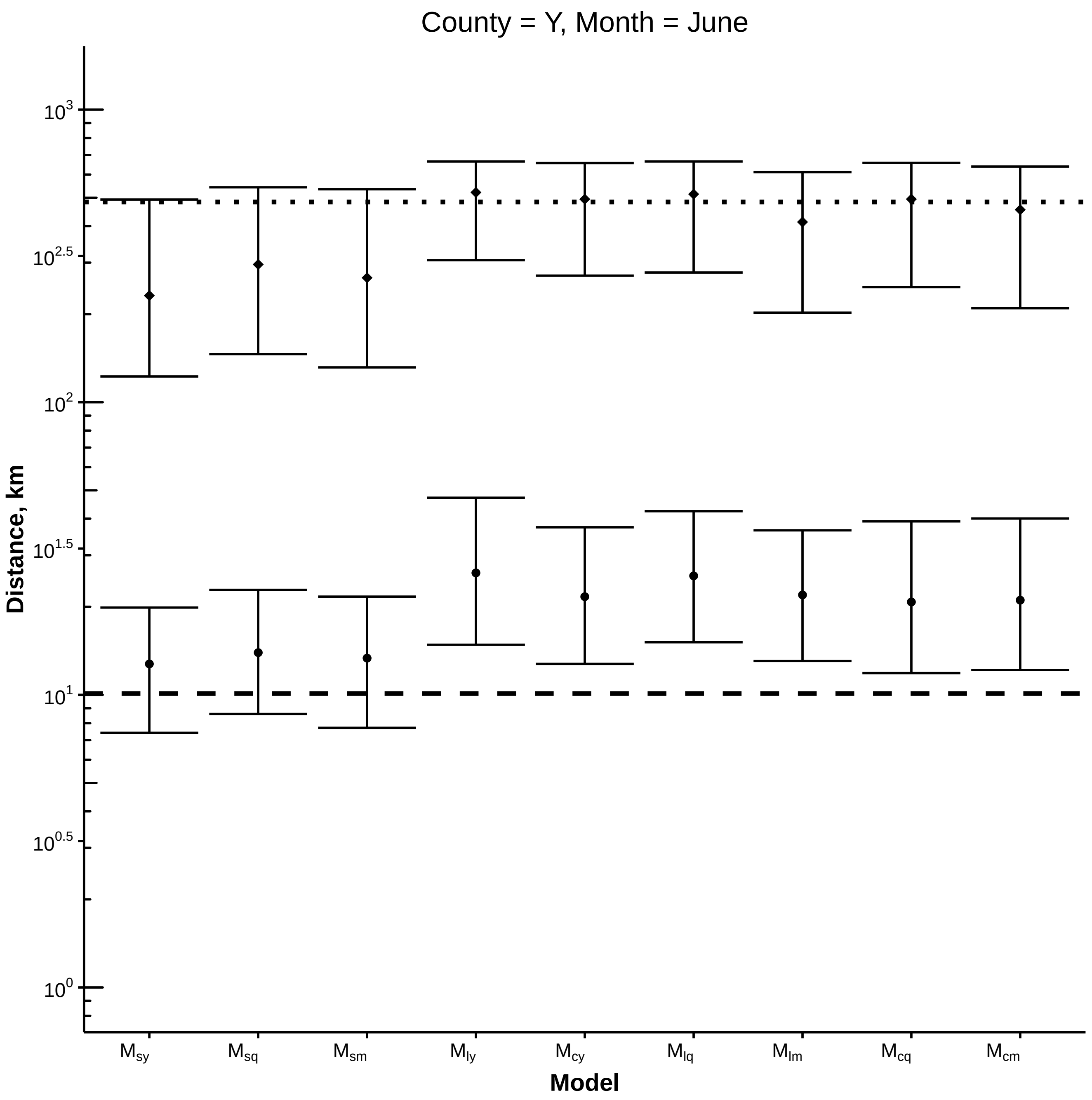

County = Y, Month = June
Distance, km
10^3
$10^{2.5}$
10^2
$10^{1.5}$
10^1
$10^{0.5}$
10^0
M_{sy}
M_{sq}
M_{sm}
M_{ly}
M_{cy}
M_{lq}
M_{lm}
M_{cq}
M_{cm}
Model

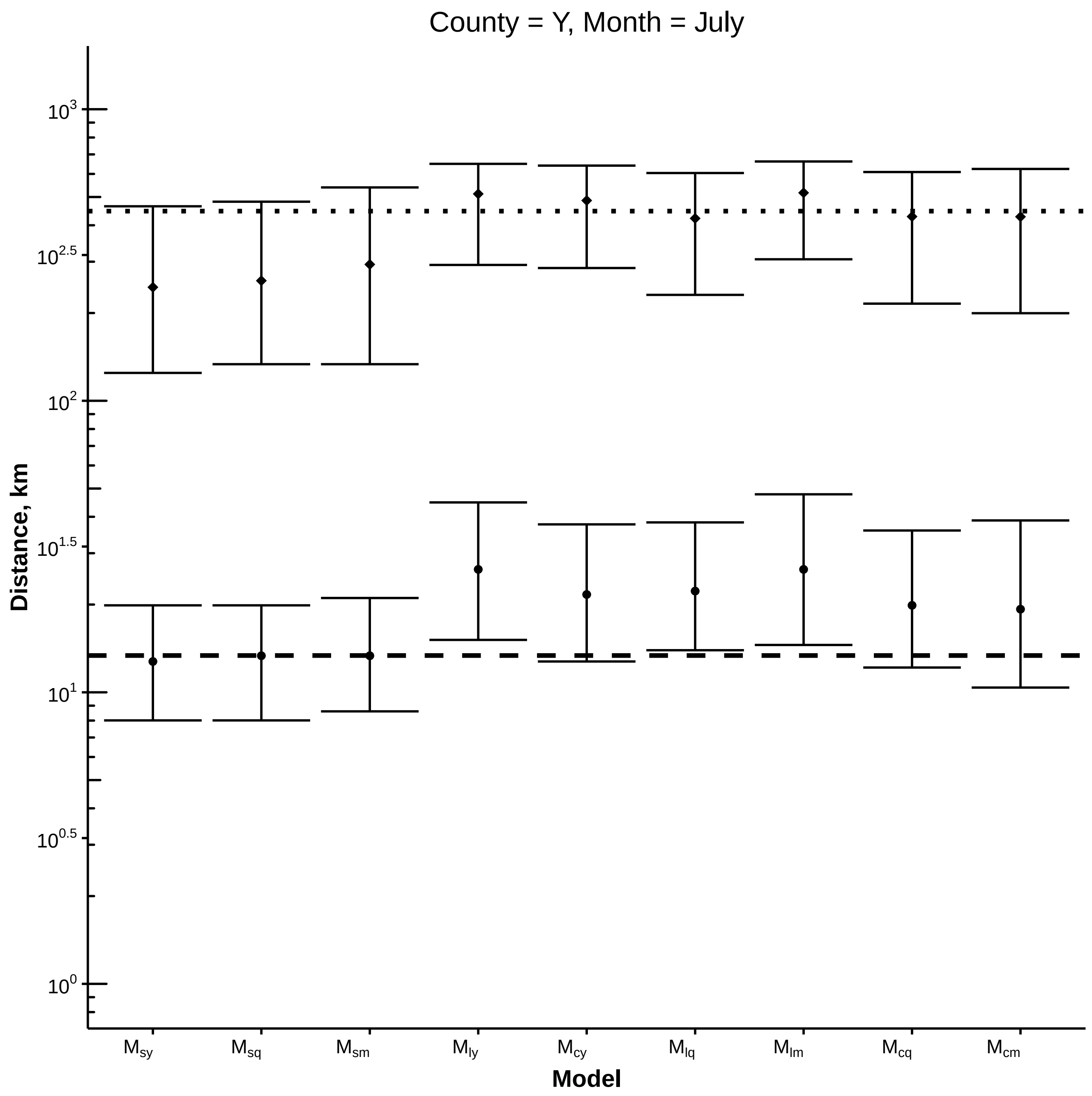
County = Y, Month = July
Distance, km
10^3
$10^{2.5}$
10^2
$10^{1.5}$
10^1
$10^{0.5}$
10^0
M_{sy}
M_{sq}
M_{sm}
M_{ly}
M_{cy}
M_{lq}
M_{lm}
M_{cq}
M_{cm}
Model

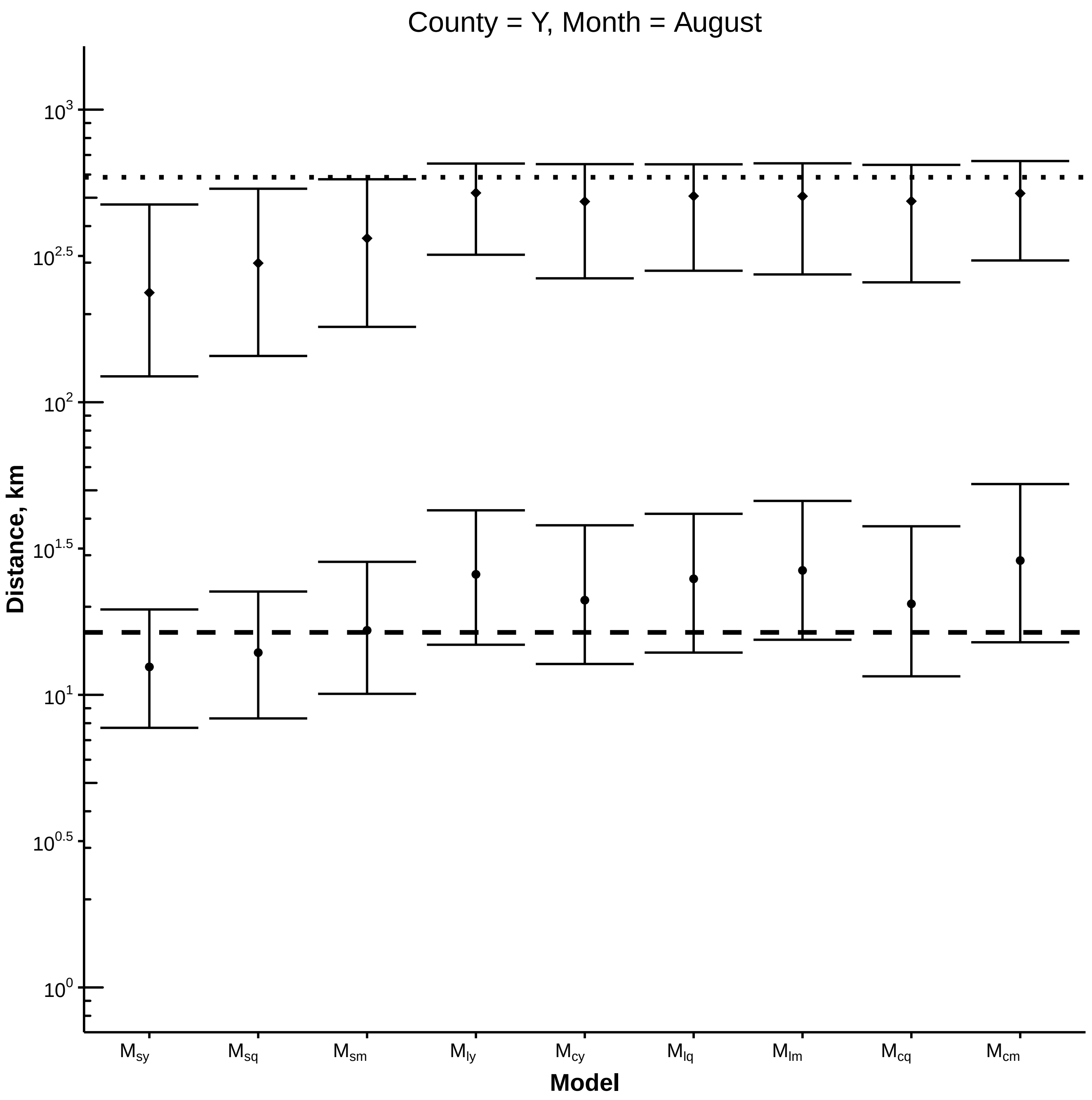

County = Y, Month = August
Distance, km
10^3
$10^{2.5}$
10^2
$10^{1.5}$
10^1
$10^{0.5}$
10^0
M_{sy}
M_{sq}
M_{sm}
M_{ly}
M_{cy}
M_{lq}
M_{lm}
M_{cq}
M_{cm}
Model

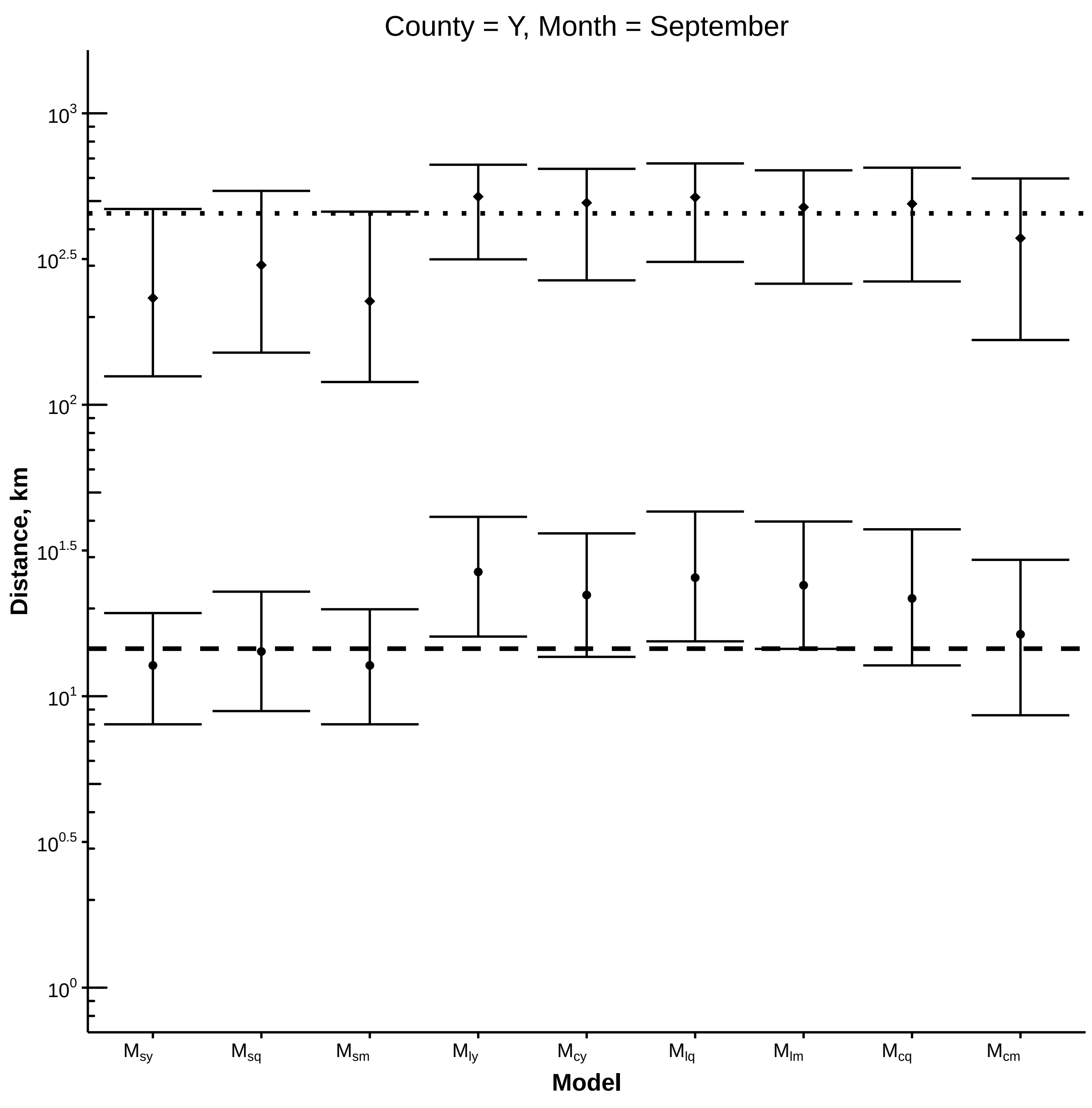

County = Y, Month = September
Distance, km
10^3
$10^{2.5}$
10^2
$10^{1.5}$
10^1
$10^{0.5}$
10^0
M_{sy}
M_{sq}
M_{sm}
M_{ly}
M_{cy}
M_{lq}
M_{lm}
M_{cq}
M_{cm}
Model

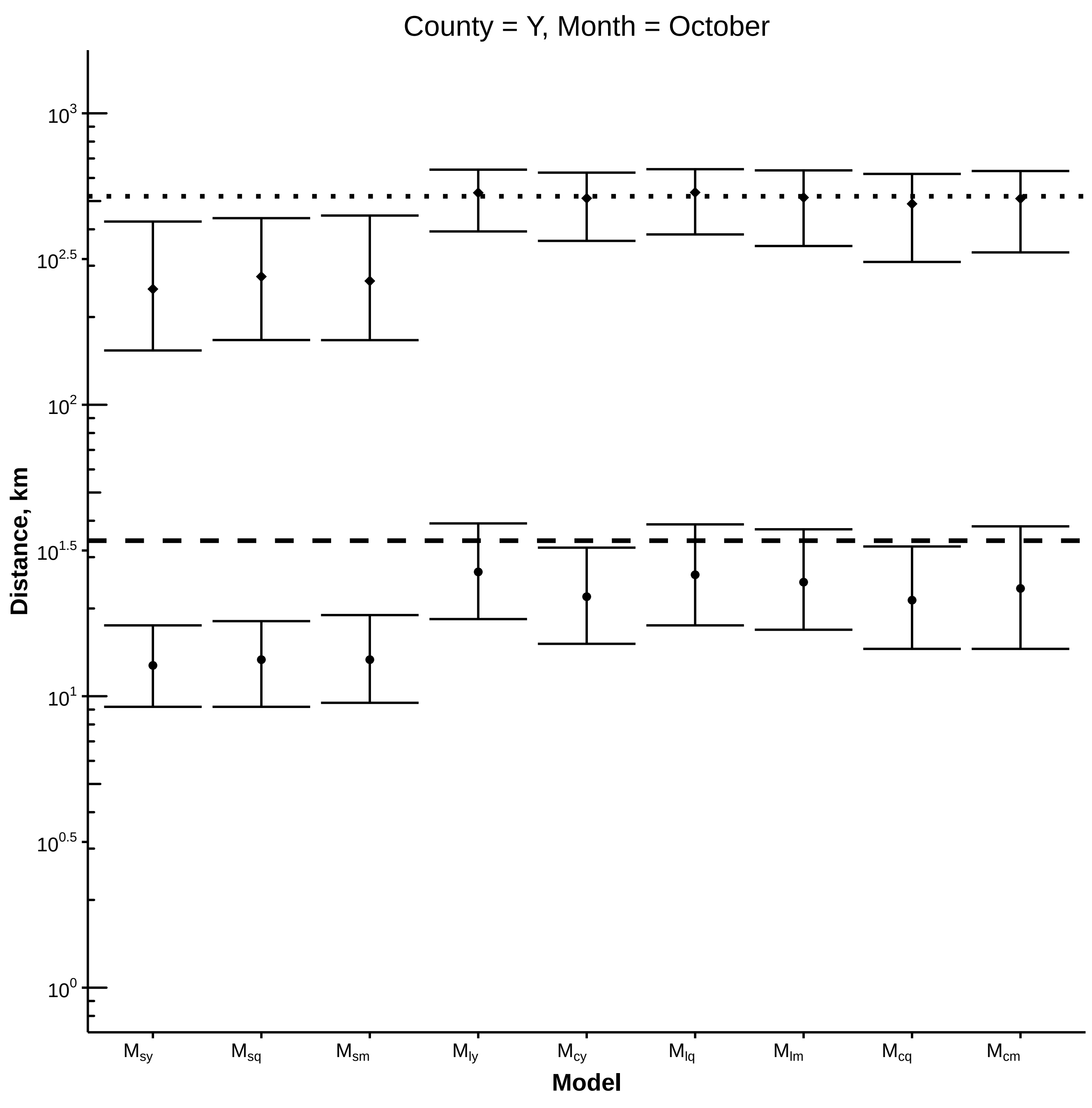

County = Y, Month = October
Distance, km
10^{3}
$10^{2.5}$
10^{2}
$10^{1.5}$
10^{1}
$10^{0.5}$
10^{0}
M_{sy}
M_{sq}
M_{sm}
M_{ly}
M_{cy}
M_{lq}
M_{lm}
M_{cq}
M_{cm}
Model

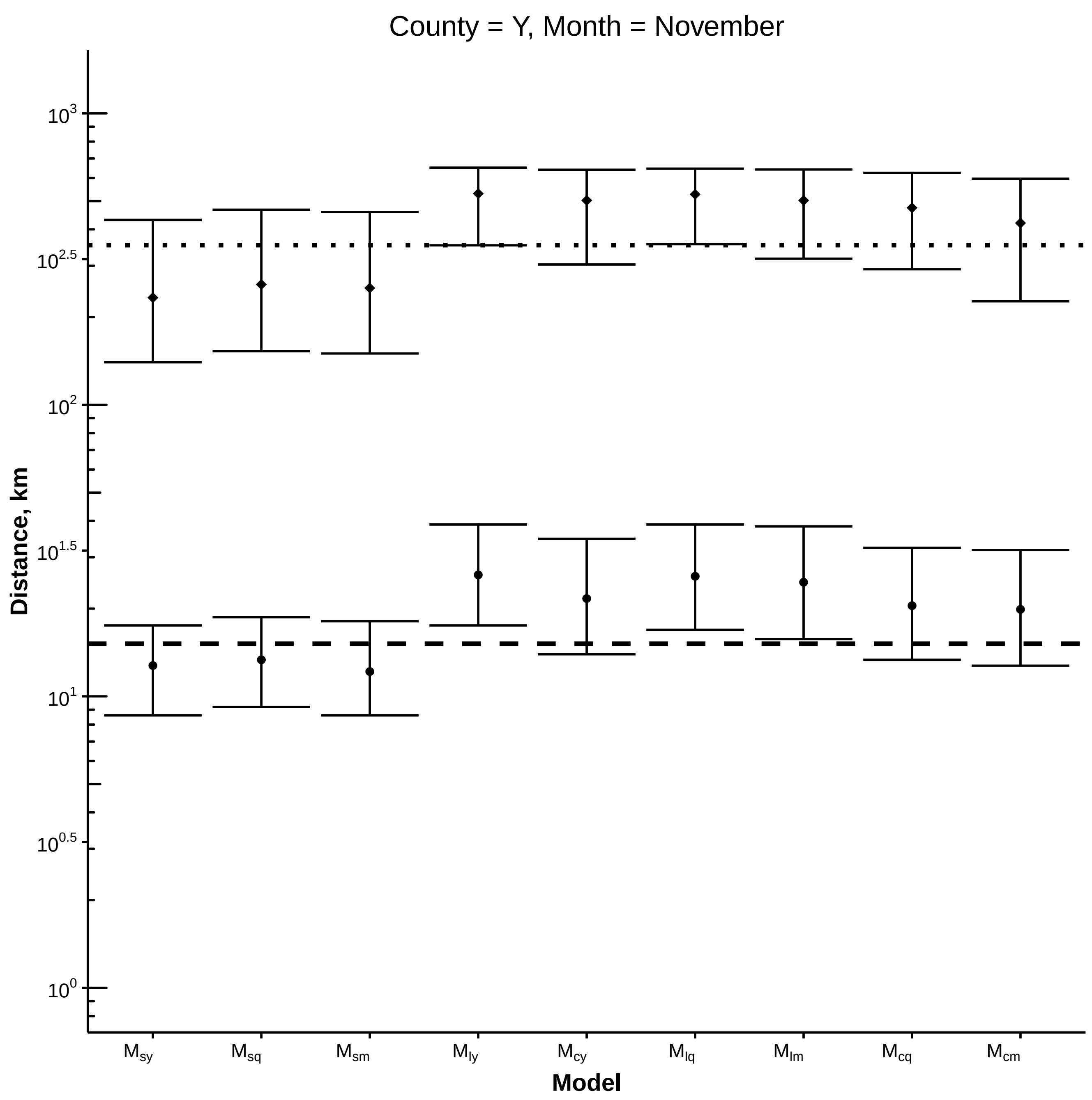
County = Y, Month = November
Distance, km
10^3
$10^{2.5}$
10^2
$10^{1.5}$
10^1
$10^{0.5}$
10^0
M_{sy}
M_{sq}
M_{sm}
M_{ly}
M_{cy}
M_{lq}
M_{lm}
M_{cq}
M_{cm}
Model

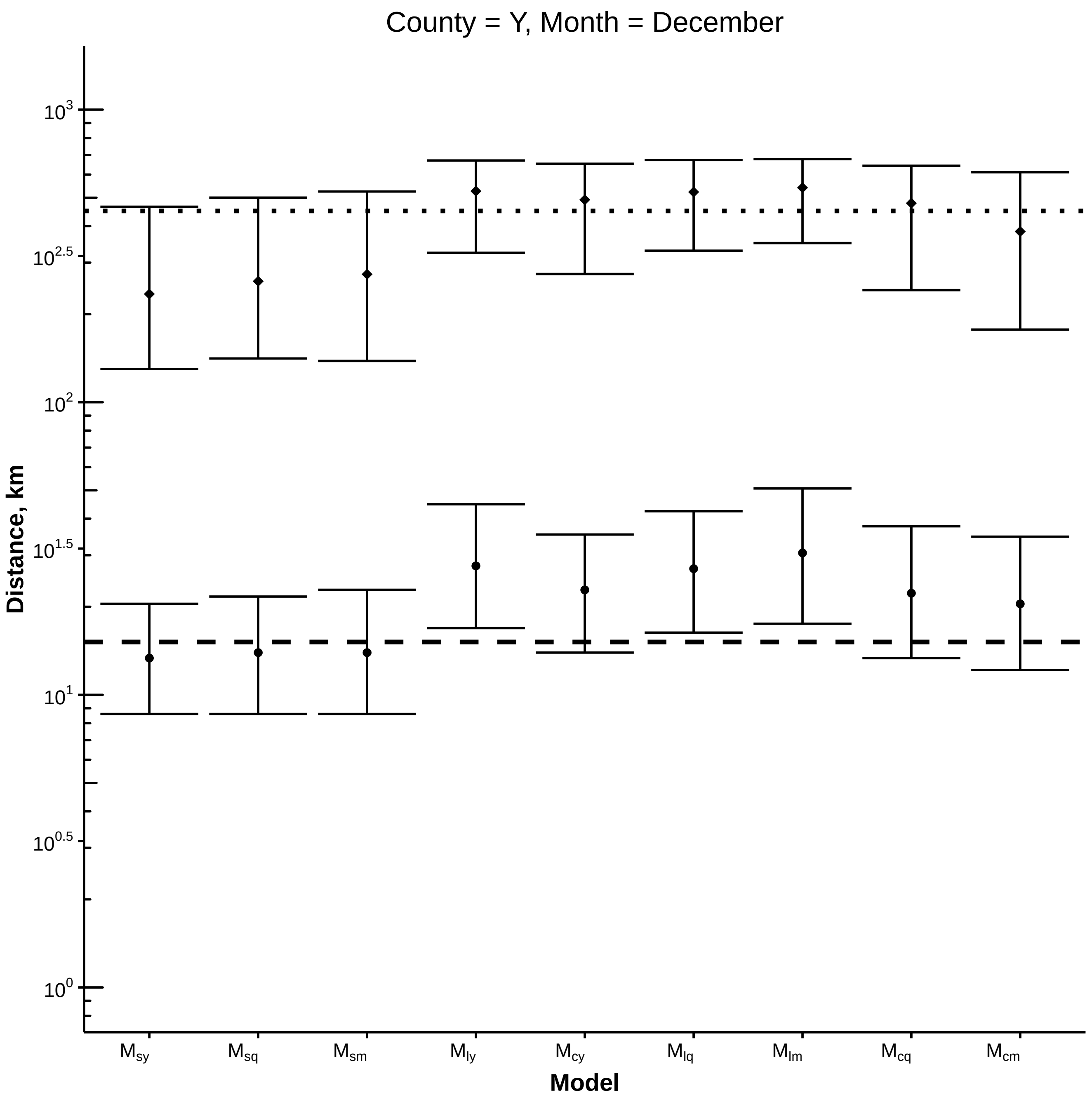

County = Y, Month = December
Distance, km
10^3
10^2.5
10^2
10^1.5
10^1
10^0.5
10^0
Msy
Msq
Msm
Mly
Mcy
Mlq
Mlm
Mcq
Mcm
Model

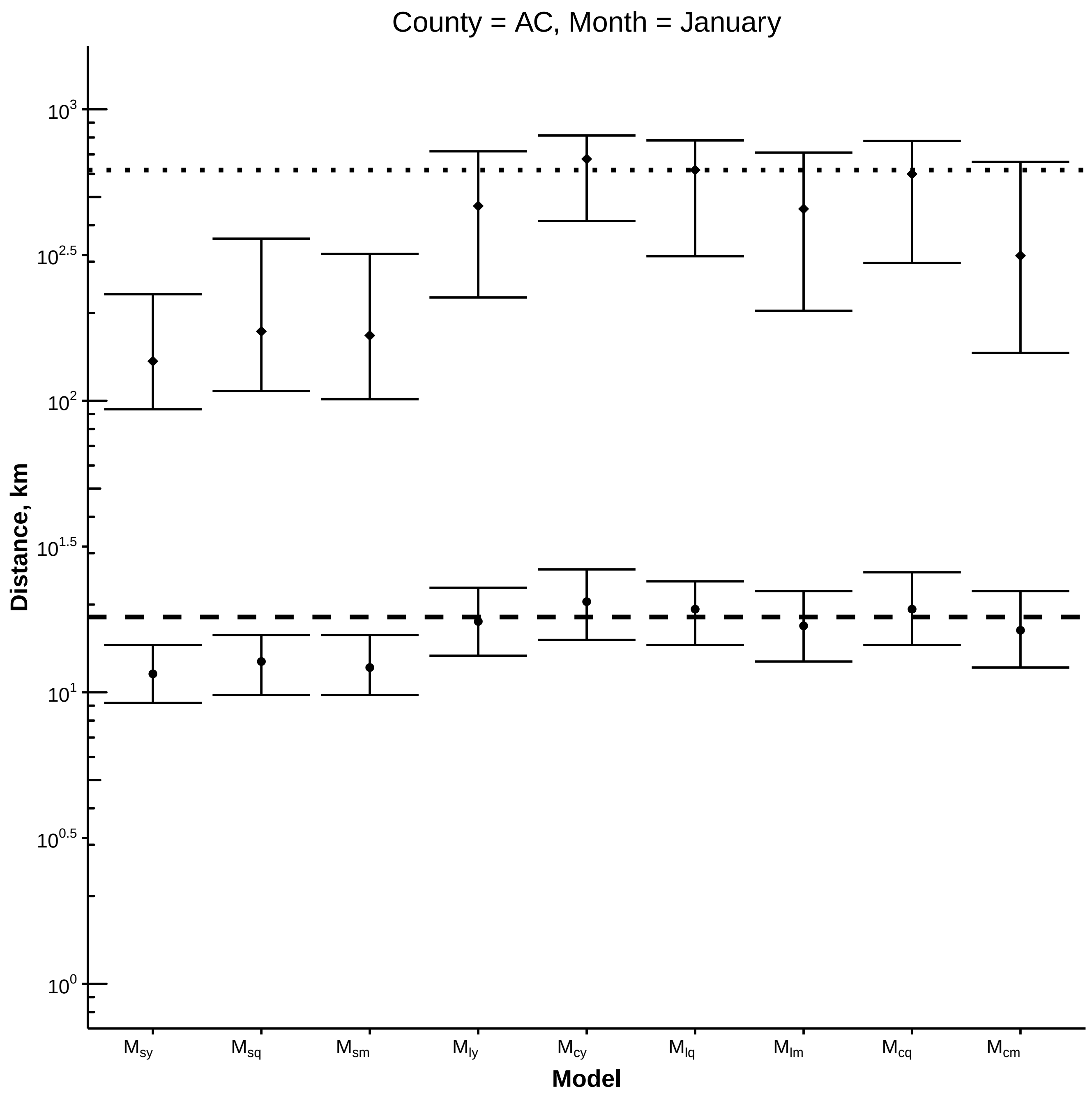

County = AC, Month = January
Distance, km
10^3
10^2.5
10^2
10^1.5
10^1
10^0.5
10^0
Msy
Msq
Msm
Mly
Mcy
Mlq
Mlm
Mcq
Mcm
Model

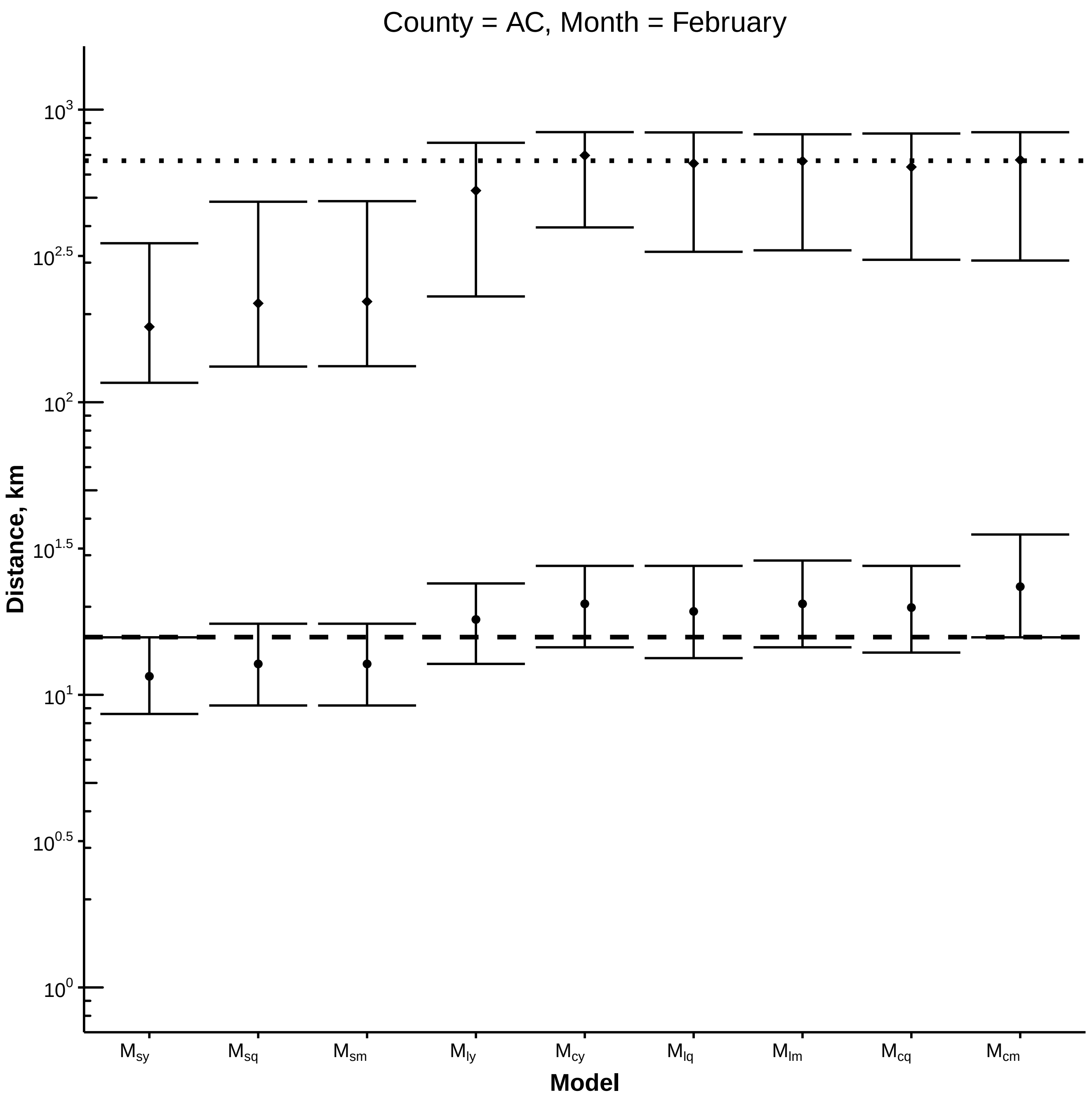
County = AC, Month = February
Distance, km
Model
10^3
10^2.5
10^2
10^1.5
10^1
10^0.5
10^0
M_sy
M_sq
M_sm
M_ly
M_cy
M_lq
M_lm
M_cq
M_cm

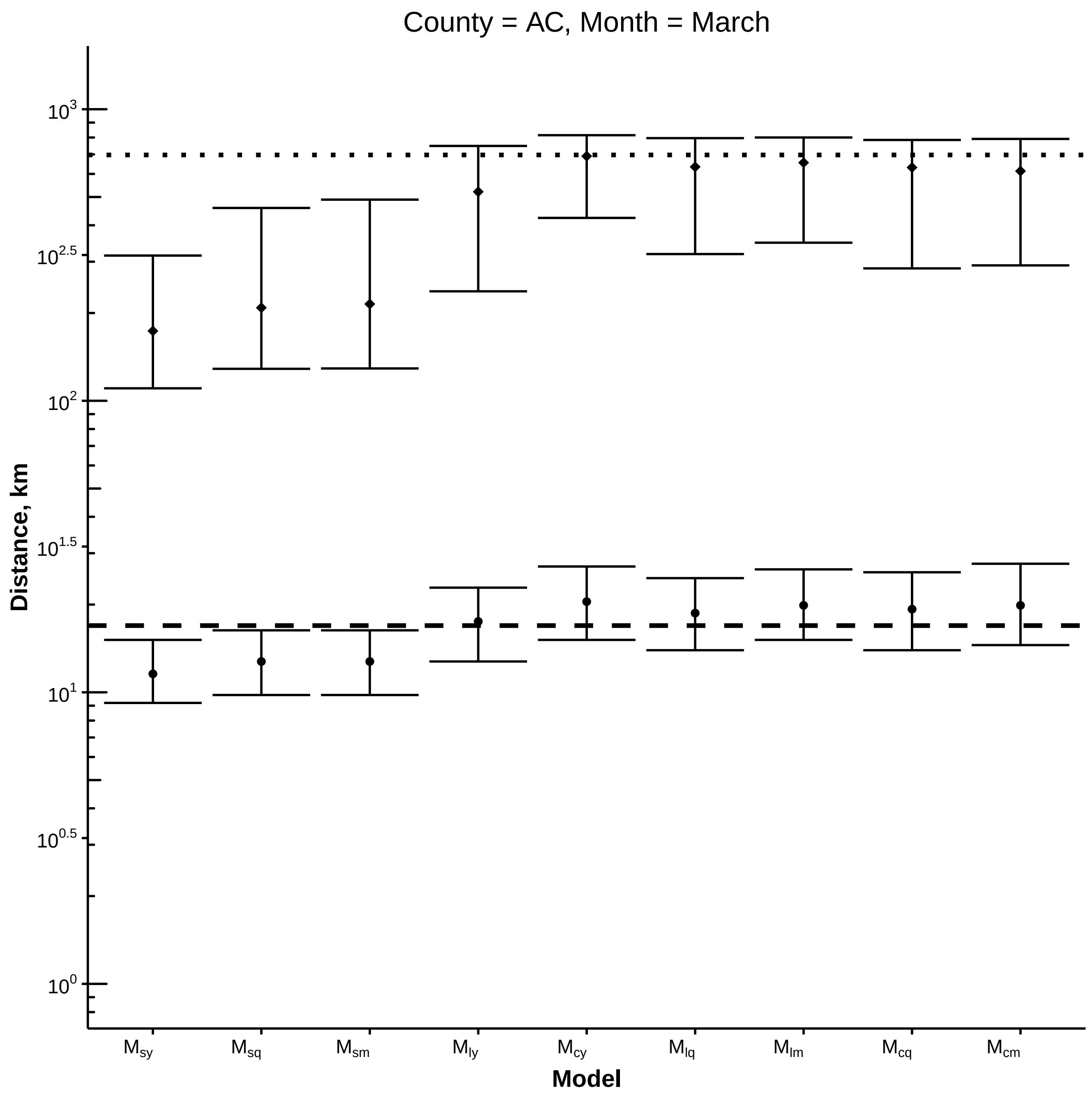
County = AC, Month = March
Distance, km
10^3
$10^{2.5}$
10^2
$10^{1.5}$
10^1
$10^{0.5}$
10^0
M_{sy}
M_{sq}
M_{sm}
M_{ly}
M_{cy}
M_{lq}
M_{lm}
M_{cq}
M_{cm}
Model

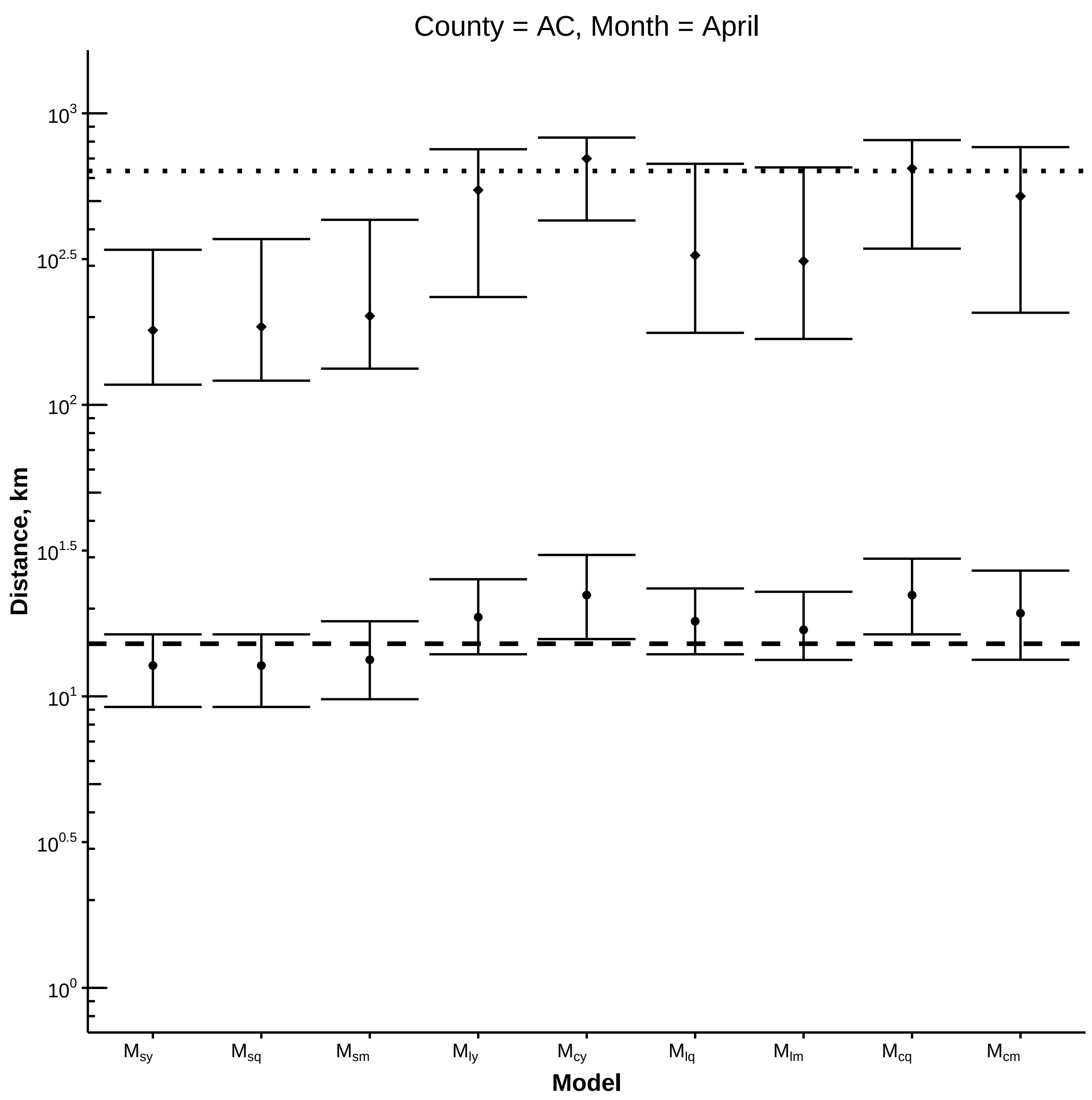

County = AC, Month = April
Distance, km
10^3
$10^{2.5}$
10^2
$10^{1.5}$
10^1
$10^{0.5}$
10^0
M_{sy}
M_{sq}
M_{sm}
M_{ly}
M_{cy}
M_{lq}
M_{lm}
M_{cq}
M_{cm}
Model

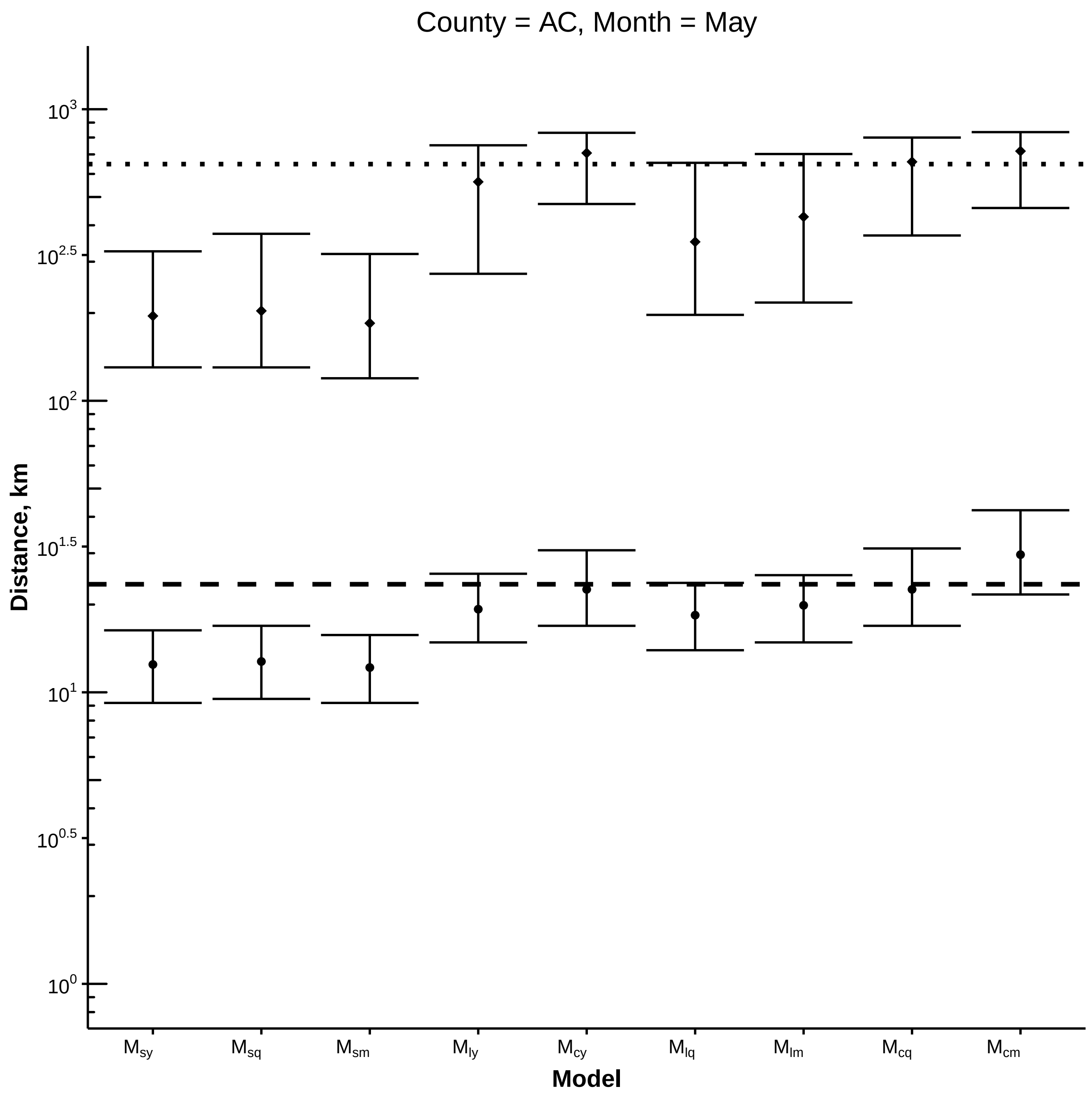

County = AC, Month = May
Distance, km
Model
10^0
$10^{0.5}$
10^1
$10^{1.5}$
10^2
$10^{2.5}$
10^3
M_{sy}
M_{sq}
M_{sm}
M_{ly}
M_{cy}
M_{lq}
M_{lm}
M_{cq}
M_{cm}

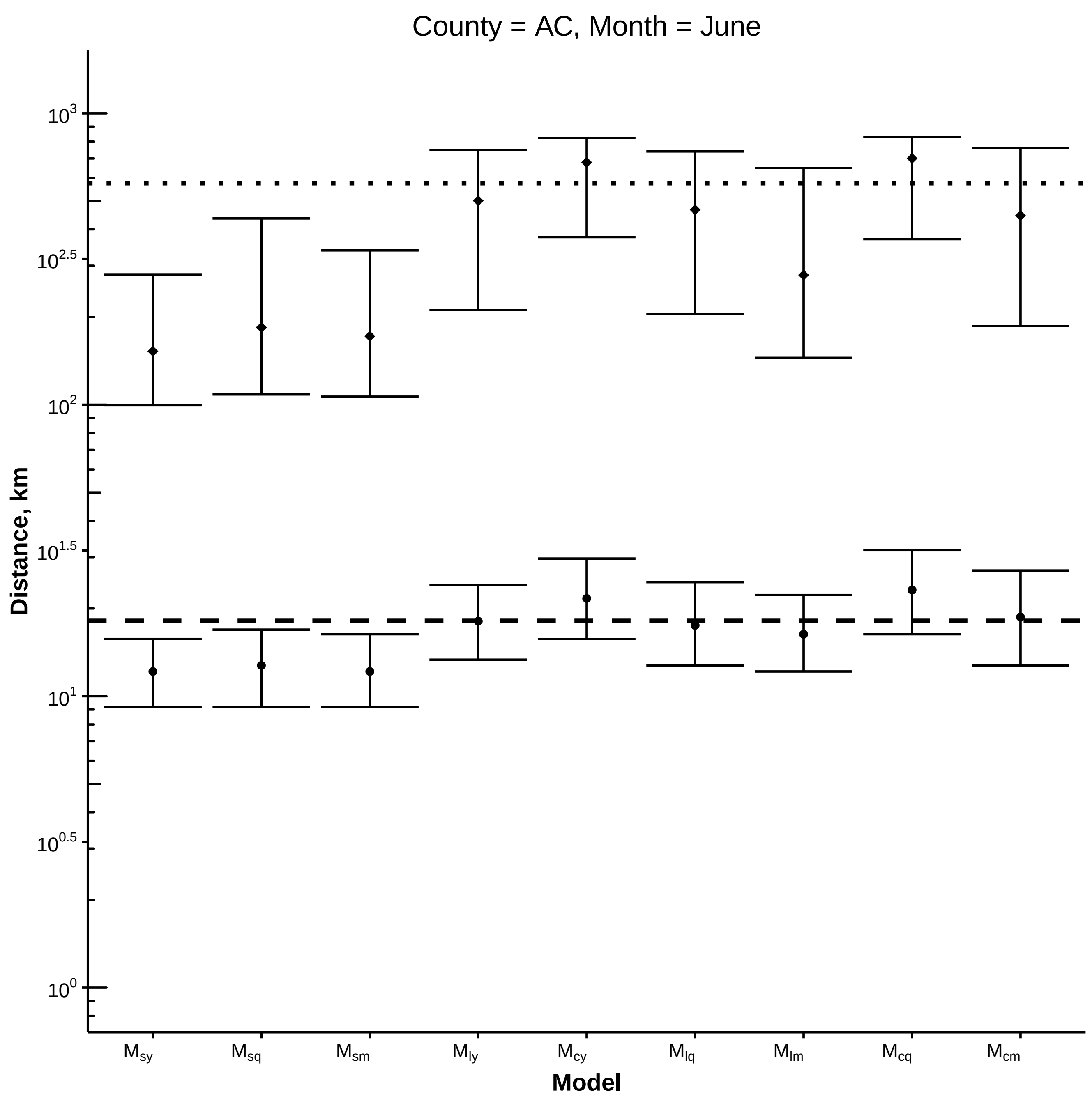

County = AC, Month = June
Distance, km
10^3
$10^{2.5}$
10^2
$10^{1.5}$
10^1
$10^{0.5}$
10^0
M_{sy}
M_{sq}
M_{sm}
M_{ly}
M_{cy}
M_{lq}
M_{lm}
M_{cq}
M_{cm}
Model

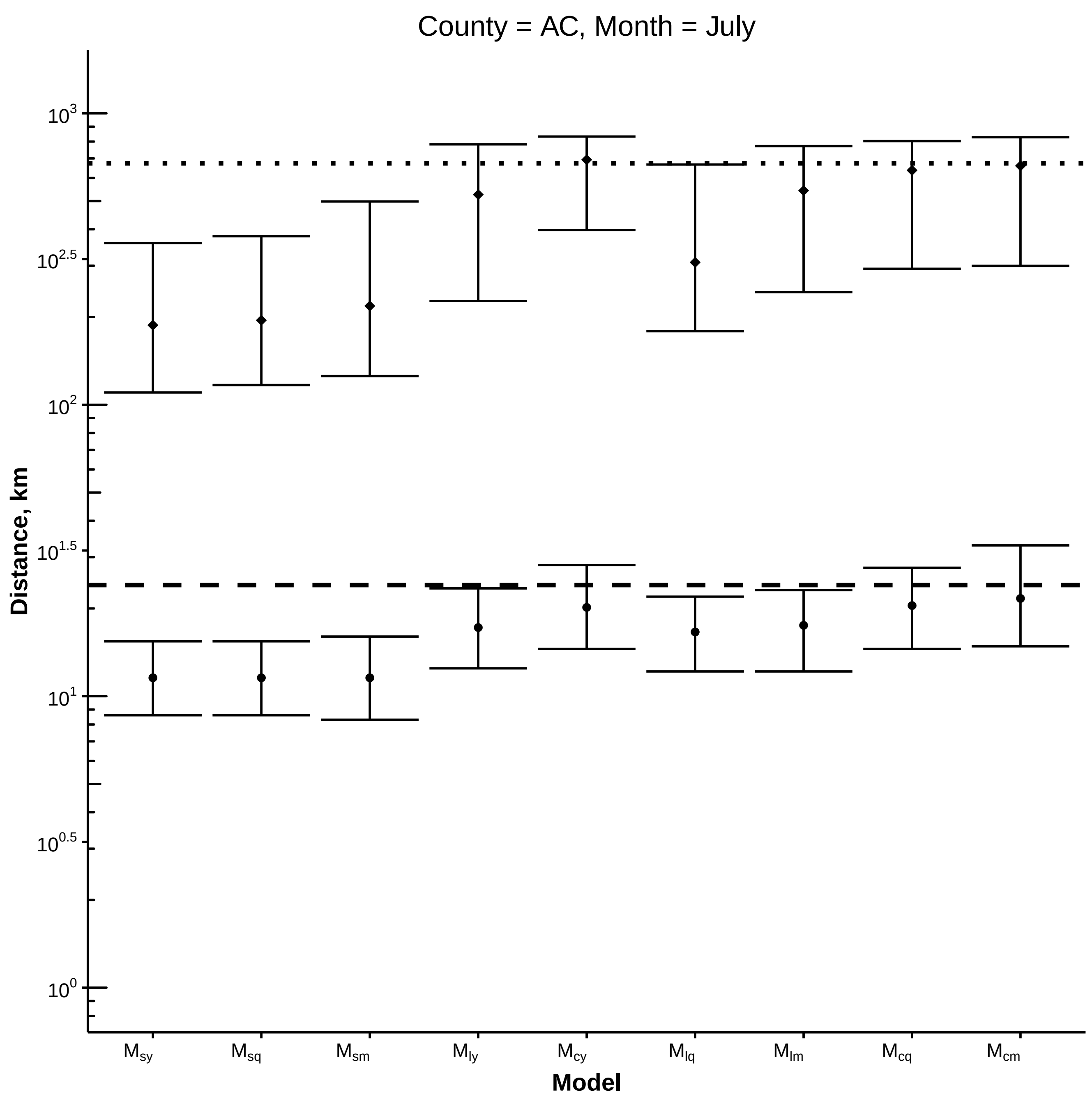

County = AC, Month = July
Distance, km
10^3
$10^{2.5}$
10^2
$10^{1.5}$
10^1
$10^{0.5}$
10^0
M_{sy}
M_{sq}
M_{sm}
M_{ly}
M_{cy}
M_{lq}
M_{lm}
M_{cq}
M_{cm}
Model

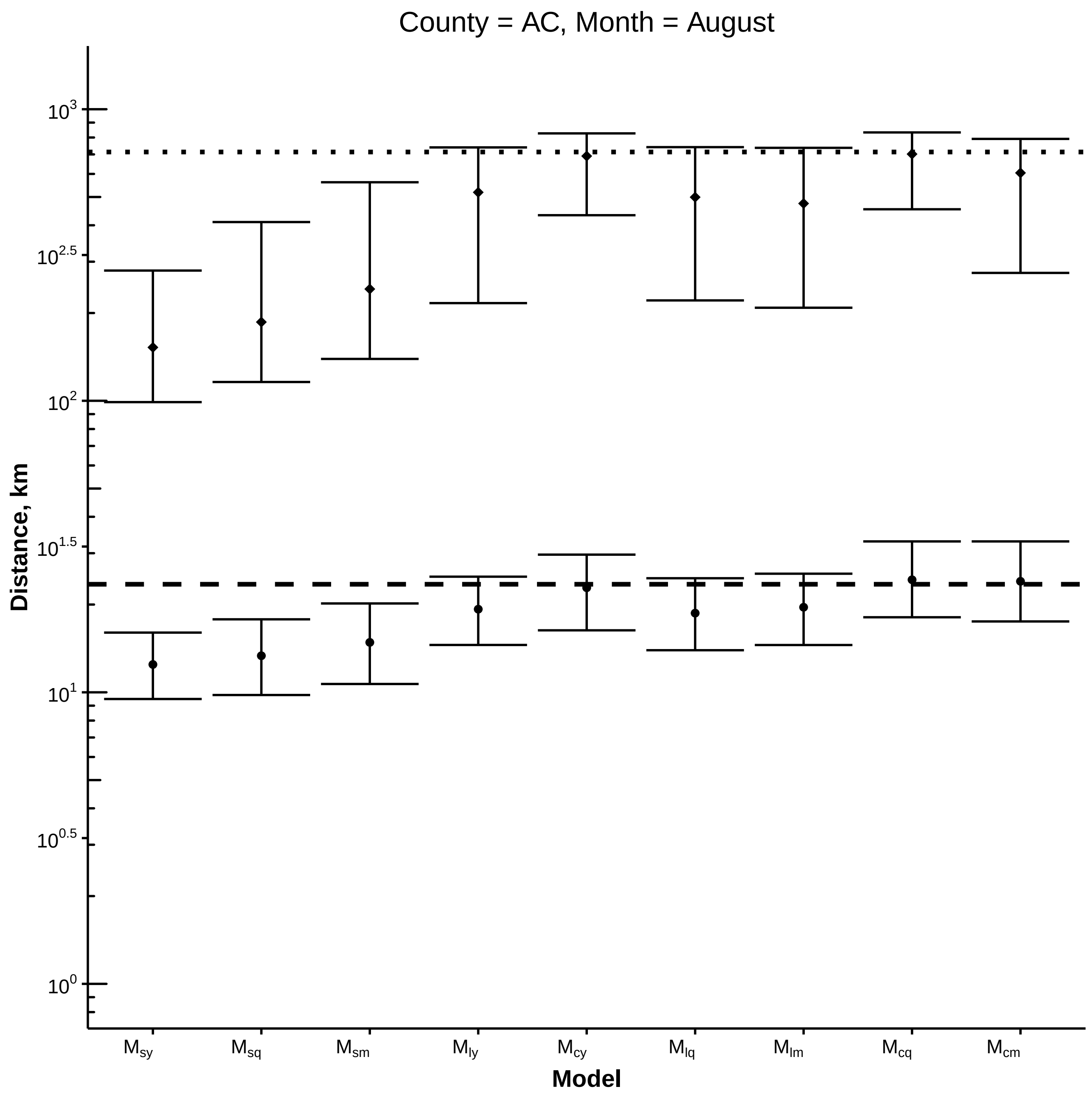
County = AC, Month = August
Distance, km
10^3
10^2.5
10^2
10^1.5
10^1
10^0.5
10^0
M_sy
M_sq
M_sm
M_ly
M_cy
M_lq
M_lm
M_cq
M_cm
Model

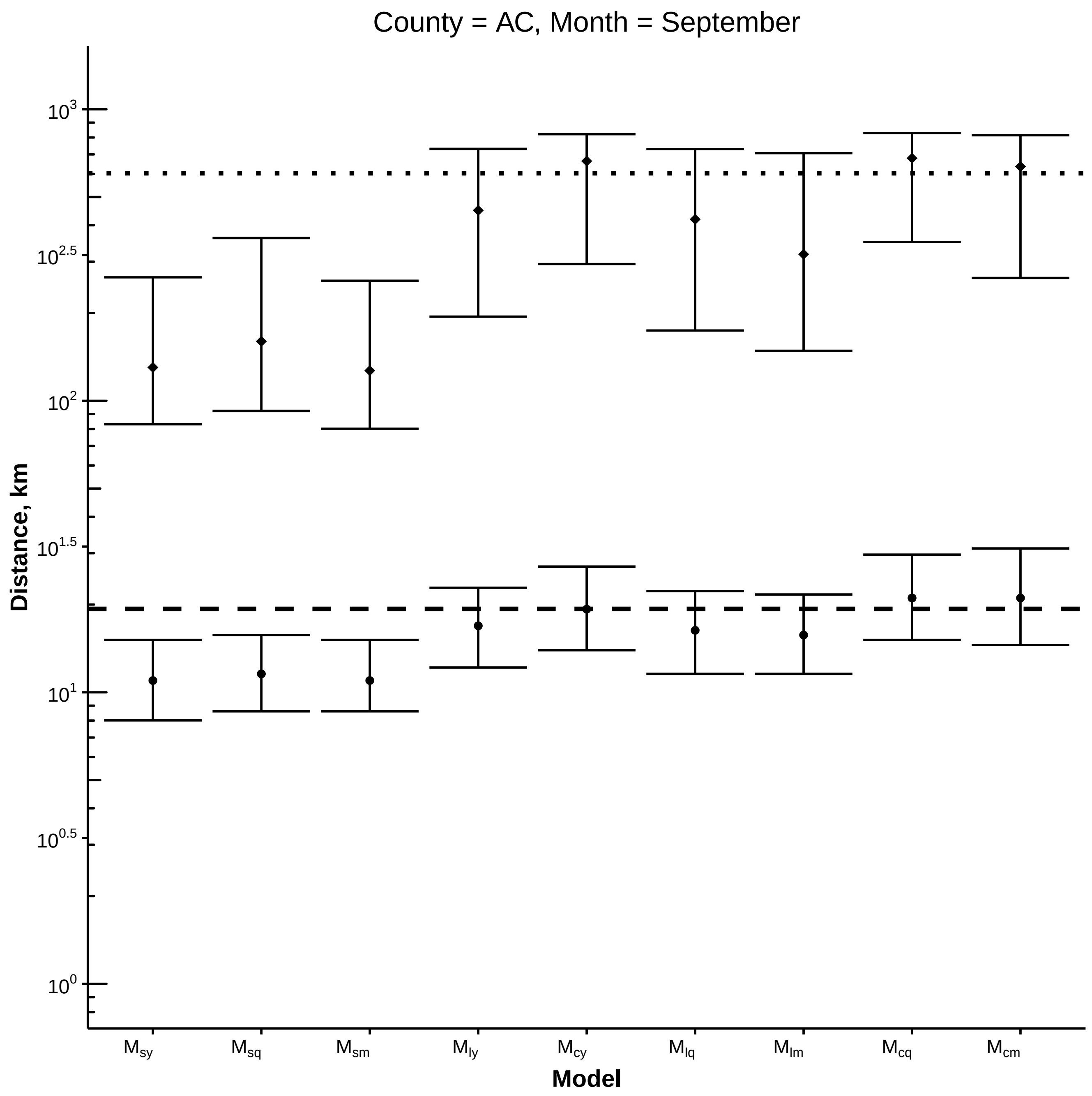
County = AC, Month = September
Distance, km
10^3
$10^{2.5}$
10^2
$10^{1.5}$
10^1
$10^{0.5}$
10^0
M_{sy}
M_{sq}
M_{sm}
M_{ly}
M_{cy}
M_{lq}
M_{lm}
M_{cq}
M_{cm}
Model

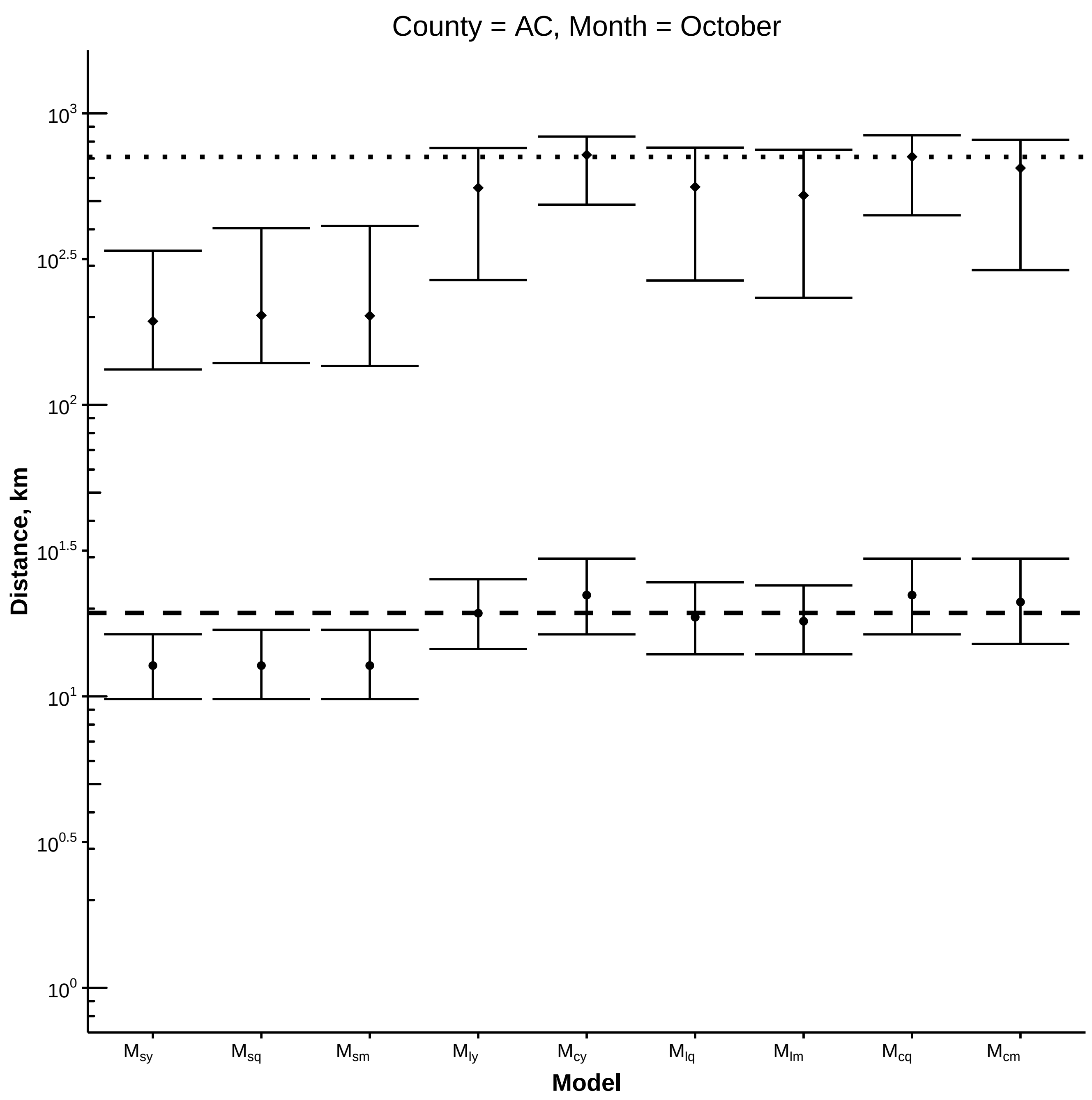
County = AC, Month = October
Distance, km
10^3
$10^{2.5}$
10^2
$10^{1.5}$
10^1
$10^{0.5}$
10^0
M_{sy}
M_{sq}
M_{sm}
M_{ly}
M_{cy}
M_{lq}
M_{lm}
M_{cq}
M_{cm}
Model

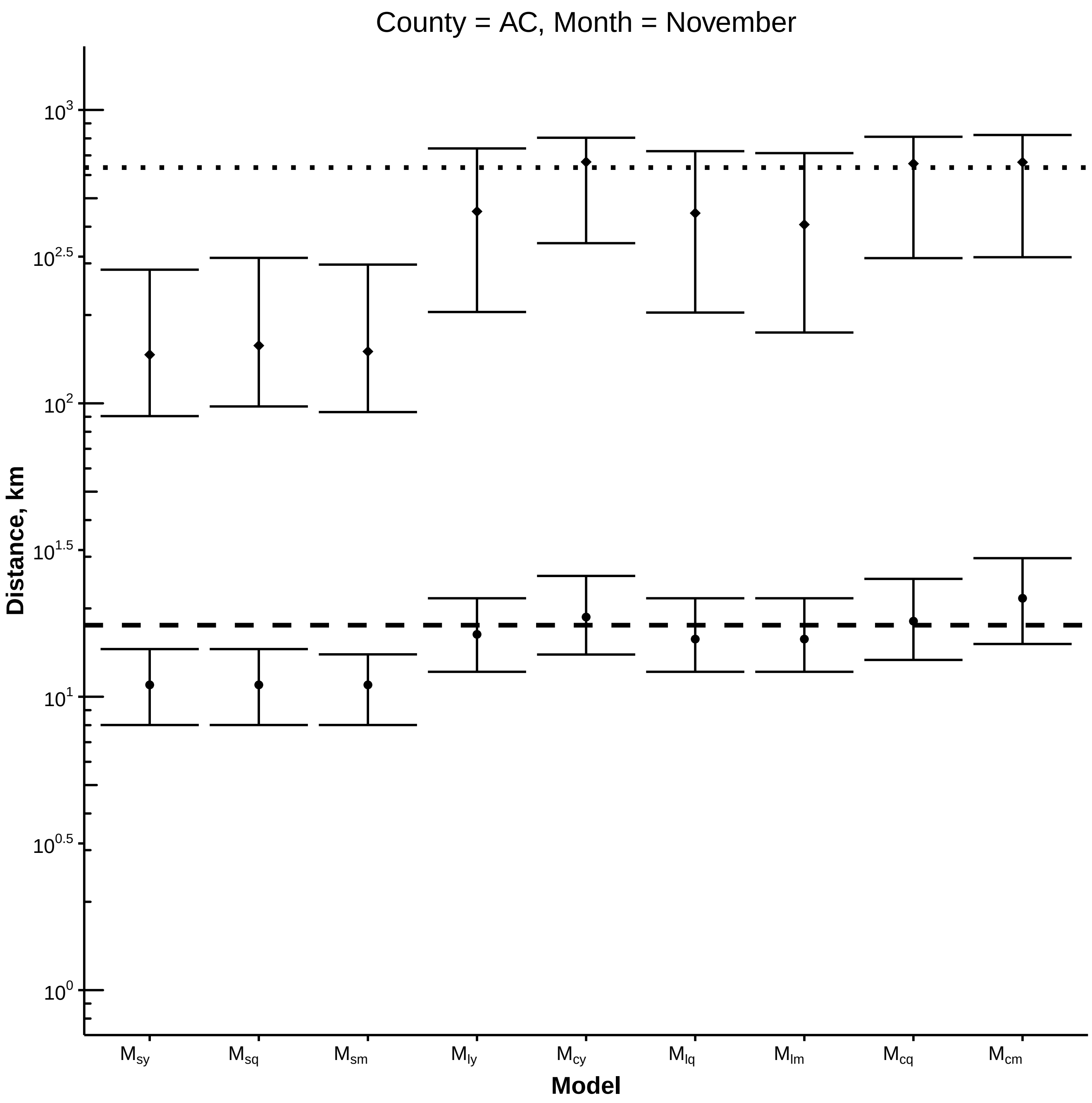
County = AC, Month = November
Distance, km
10^3
$10^{2.5}$
10^2
$10^{1.5}$
10^1
$10^{0.5}$
10^0
M_{sy}
M_{sq}
M_{sm}
M_{ly}
M_{cy}
M_{lq}
M_{lm}
M_{cq}
M_{cm}
Model

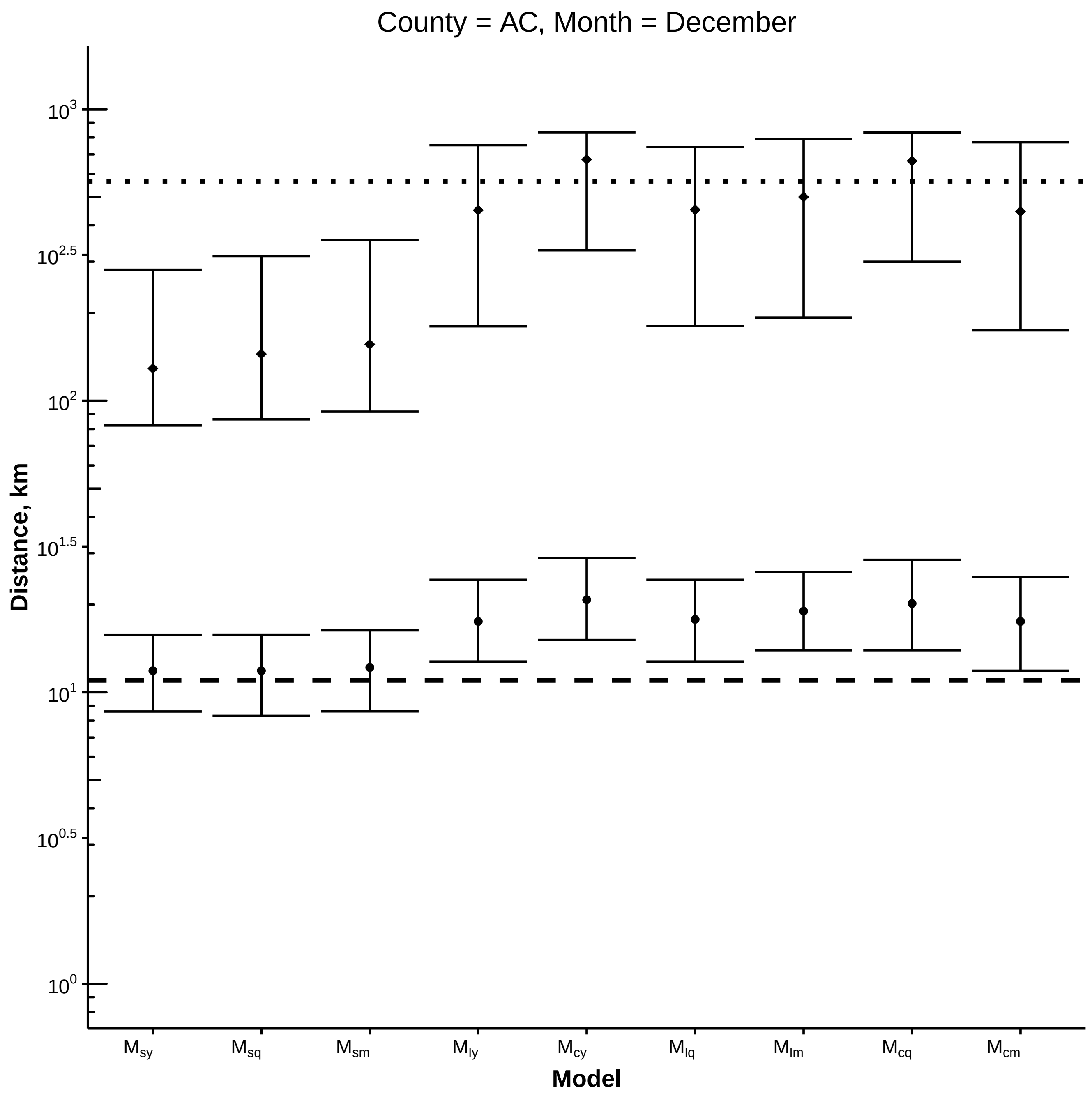
County = AC, Month = December
Distance, km
10^3
$10^{2.5}$
10^2
$10^{1.5}$
10^1
$10^{0.5}$
10^0
M_{sy} M_{sq} M_{sm} M_{ly} M_{cy} M_{lq} M_{lm} M_{cq} M_{cm}
Model

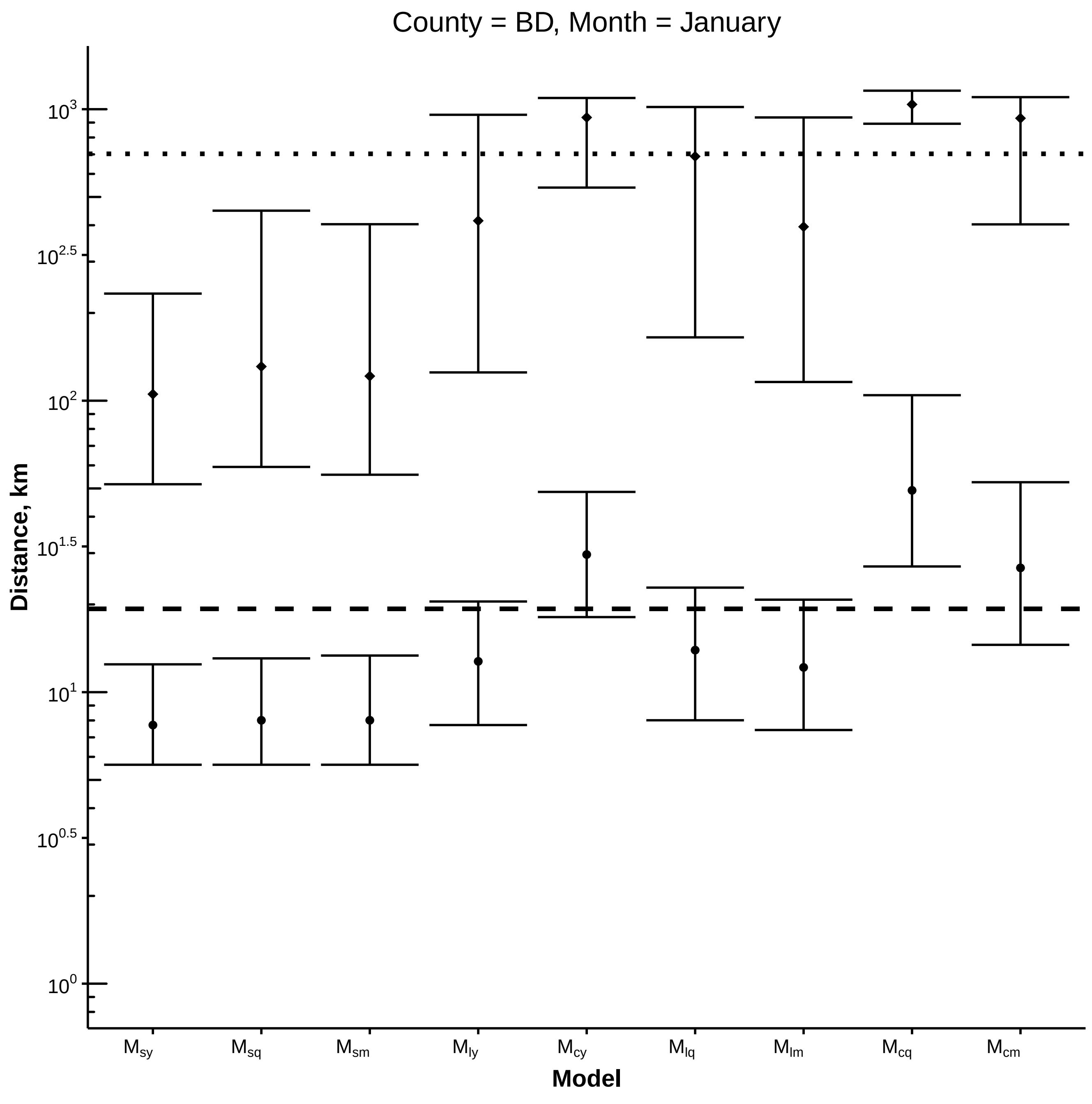

County = BD, Month = January
Distance, km
10^3
$10^{2.5}$
10^2
$10^{1.5}$
10^1
$10^{0.5}$
10^0
M_{sy}
M_{sq}
M_{sm}
M_{ly}
M_{cy}
M_{lq}
M_{lm}
M_{cq}
M_{cm}
Model

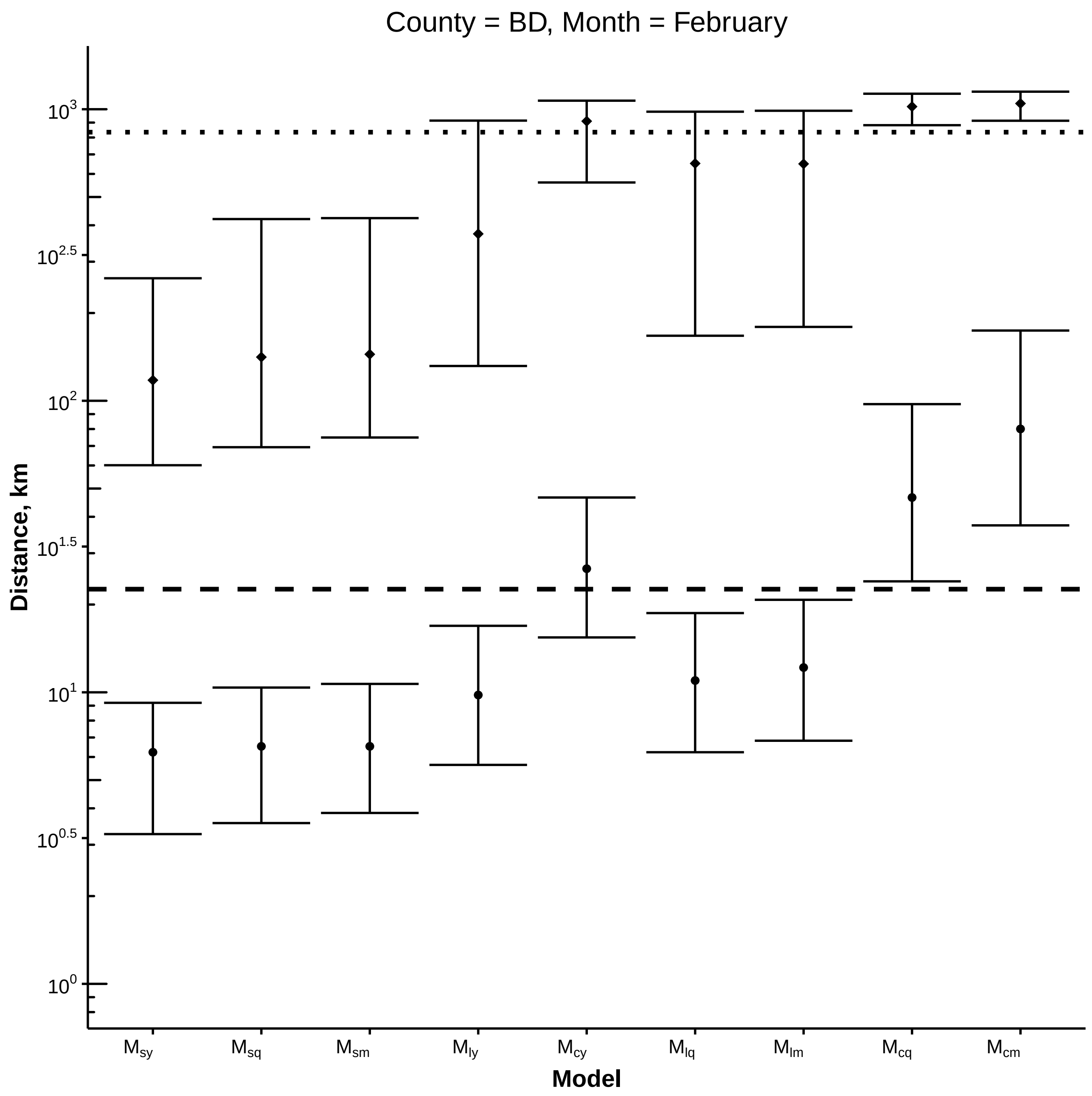

County = BD, Month = February
Distance, km
10^3
$10^{2.5}$
10^2
$10^{1.5}$
10^1
$10^{0.5}$
10^0
M_{sy}
M_{sq}
M_{sm}
M_{ly}
M_{cy}
M_{lq}
M_{lm}
M_{cq}
M_{cm}
Model

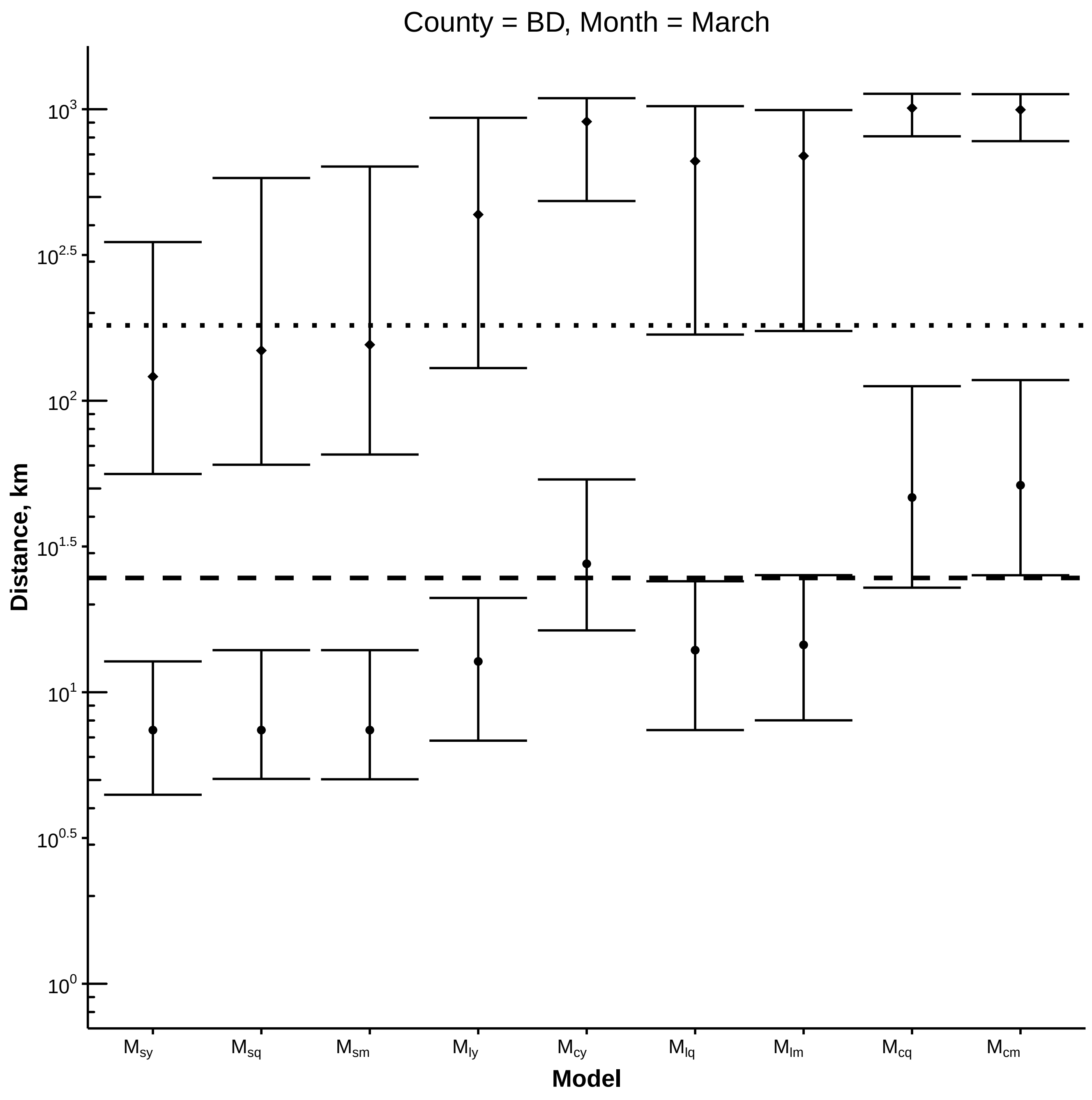
County = BD, Month = March
Distance, km
10^3
$10^{2.5}$
10^2
$10^{1.5}$
10^1
$10^{0.5}$
10^0
M_{sy}
M_{sq}
M_{sm}
M_{ly}
M_{cy}
M_{lq}
M_{lm}
M_{cq}
M_{cm}
Model

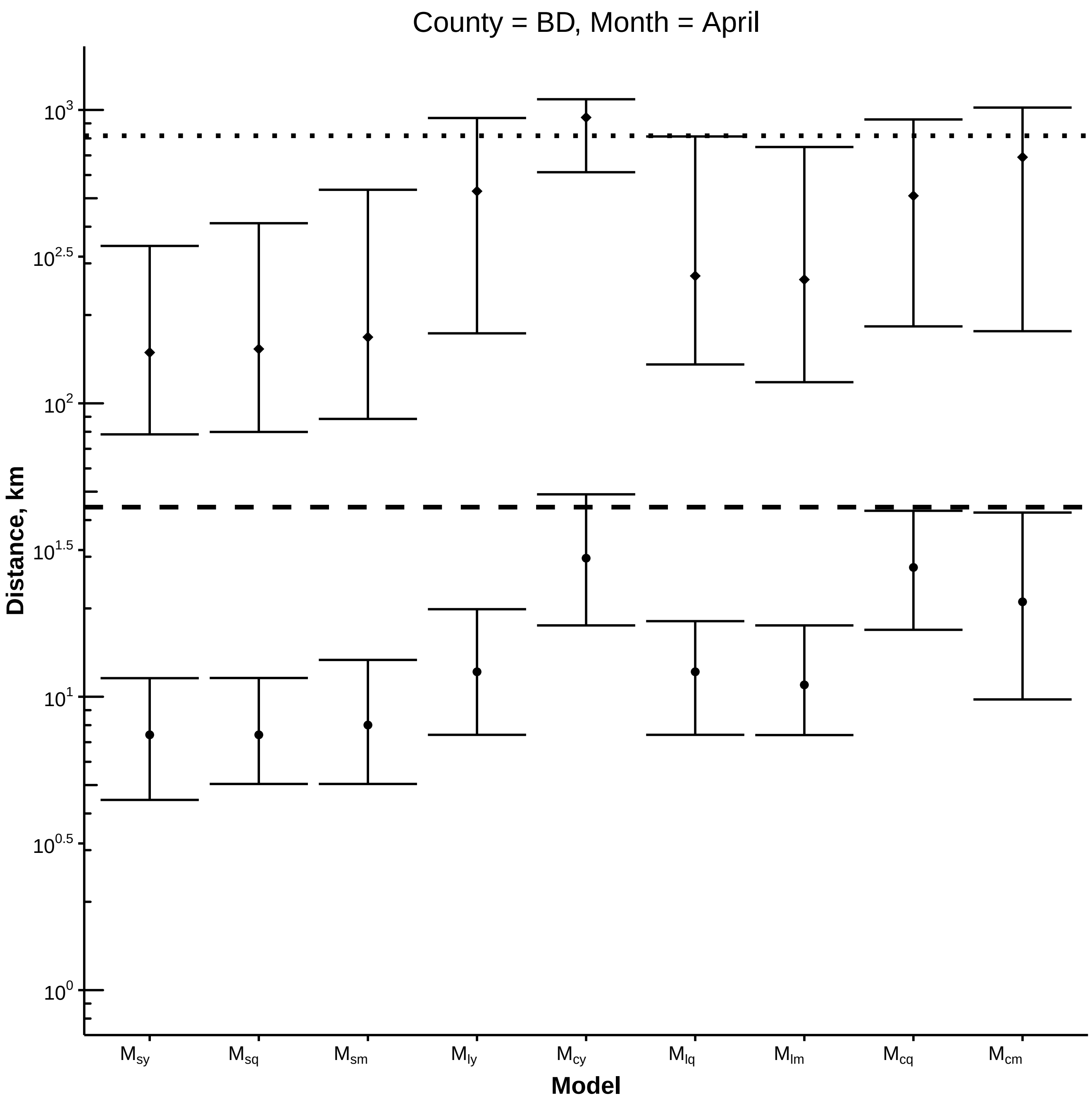

County = BD, Month = April
Distance, km
10^3
$10^{2.5}$
10^2
$10^{1.5}$
10^1
$10^{0.5}$
10^0
M_{sy}
M_{sq}
M_{sm}
M_{ly}
M_{cy}
M_{lq}
M_{lm}
M_{cq}
M_{cm}
Model

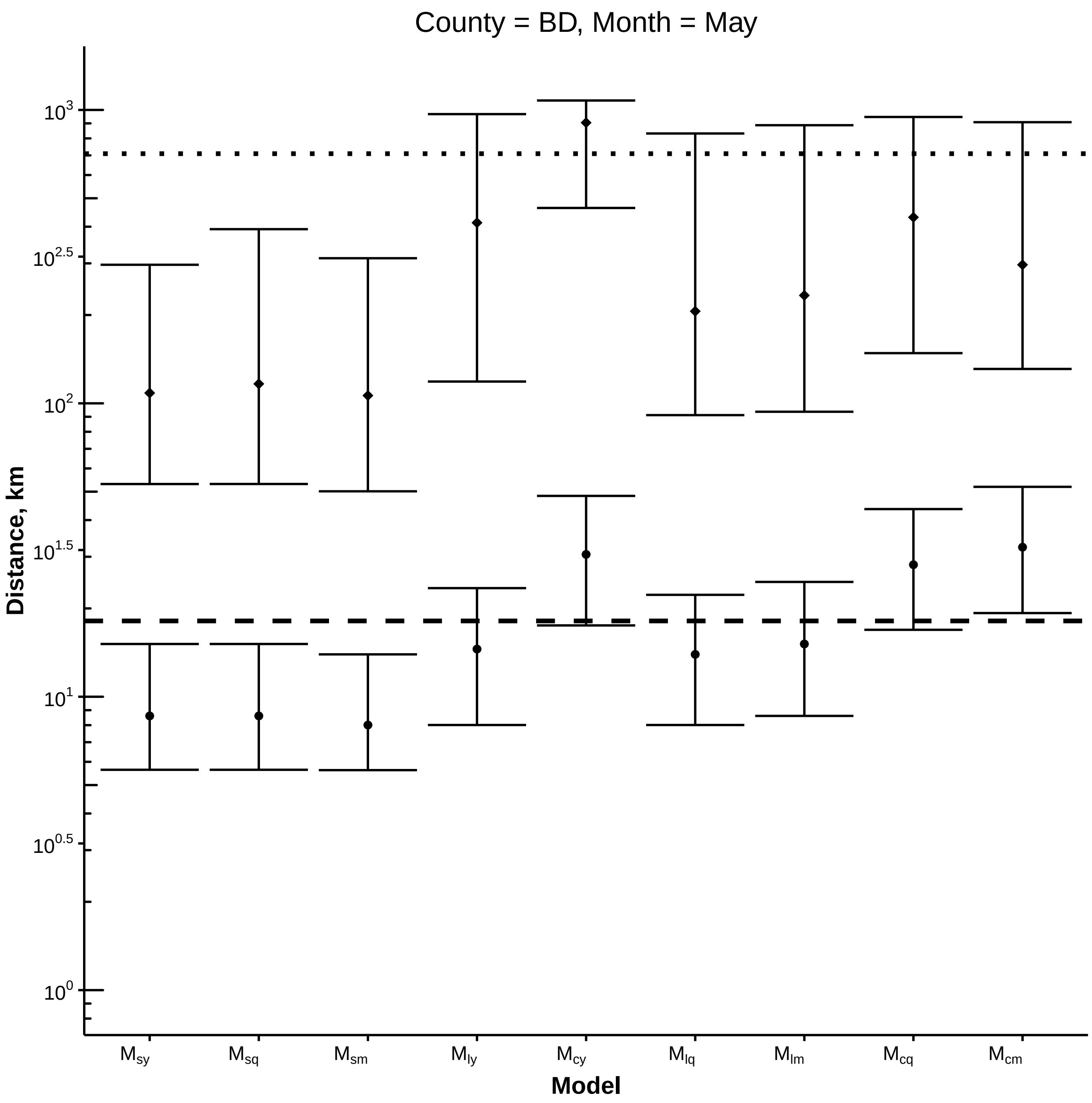

County = BD, Month = May
Distance, km
10^{3}
$10^{2.5}$
10^{2}
$10^{1.5}$
10^{1}
$10^{0.5}$
10^{0}
M_{sy}
M_{sq}
M_{sm}
M_{ly}
M_{cy}
M_{lq}
M_{lm}
M_{cq}
M_{cm}
Model

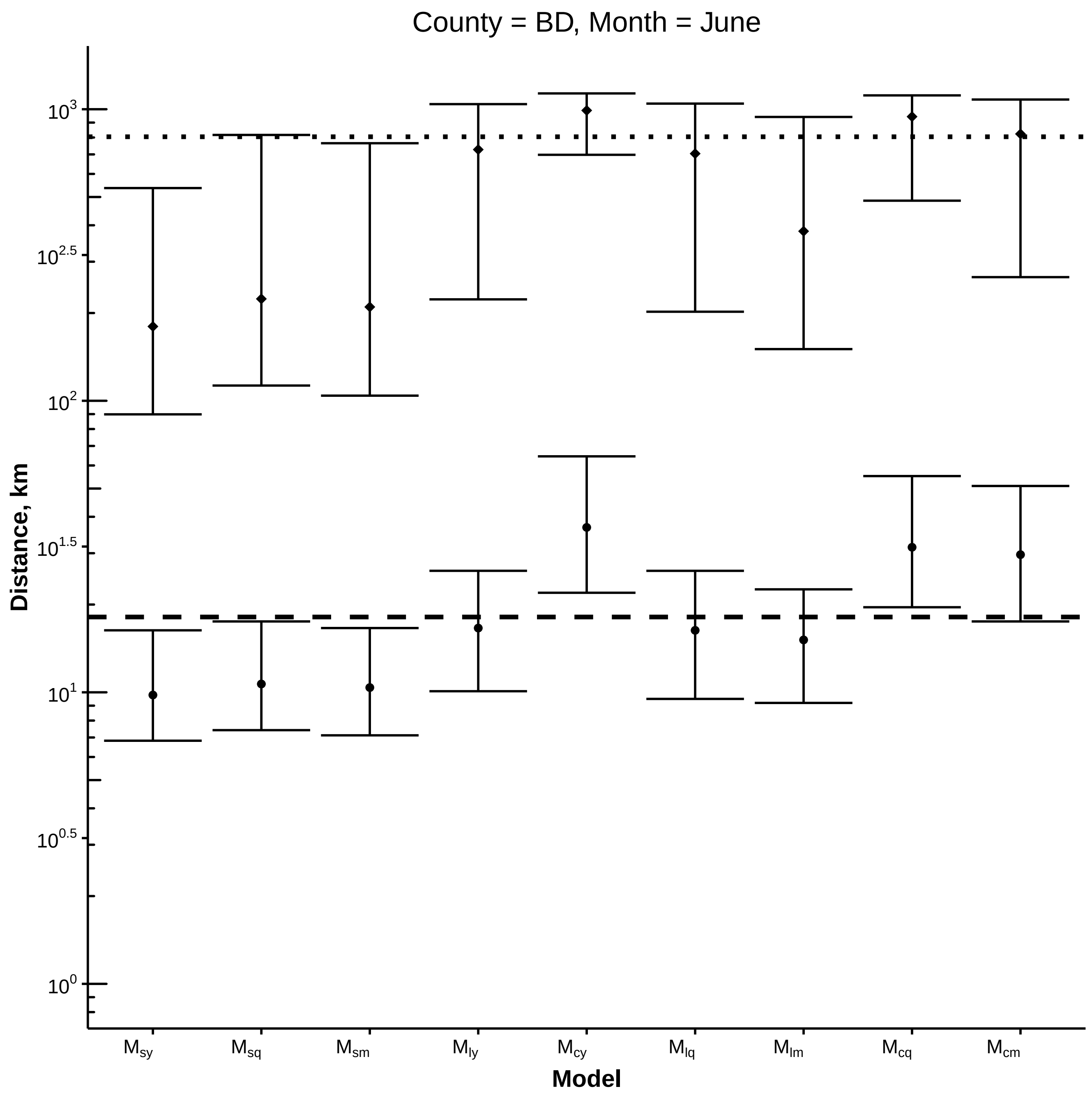
County = BD, Month = June
Distance, km
10^3
$10^{2.5}$
10^2
$10^{1.5}$
10^1
$10^{0.5}$
10^0
M_{sy}
M_{sq}
M_{sm}
M_{ly}
M_{cy}
M_{lq}
M_{lm}
M_{cq}
M_{cm}
Model

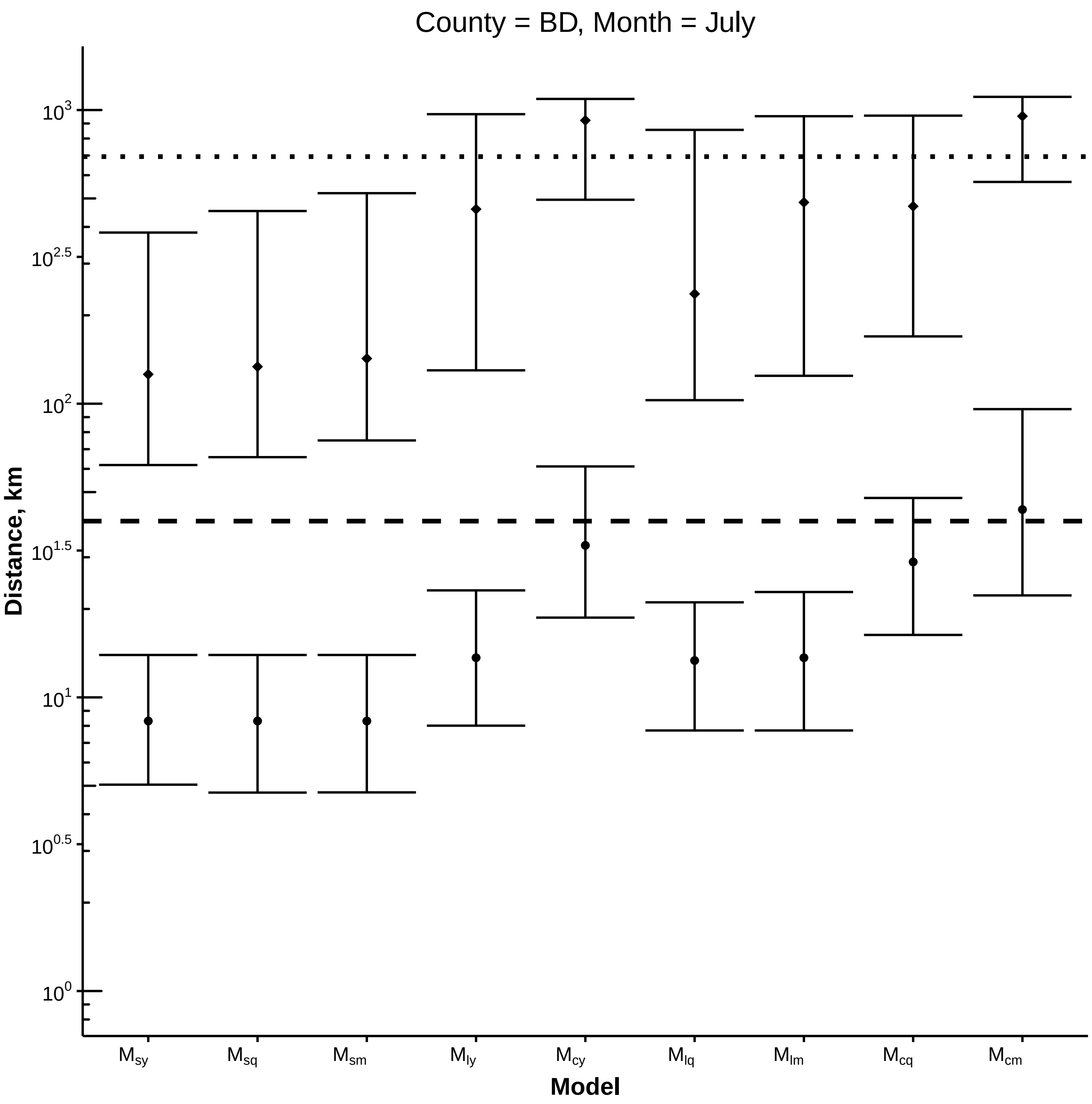

County = BD, Month = July
Distance, km
10^3
$10^{2.5}$
10^2
$10^{1.5}$
10^1
$10^{0.5}$
10^0
M_{sy}
M_{sq}
M_{sm}
M_{ly}
M_{cy}
M_{lq}
M_{lm}
M_{cq}
M_{cm}
Model

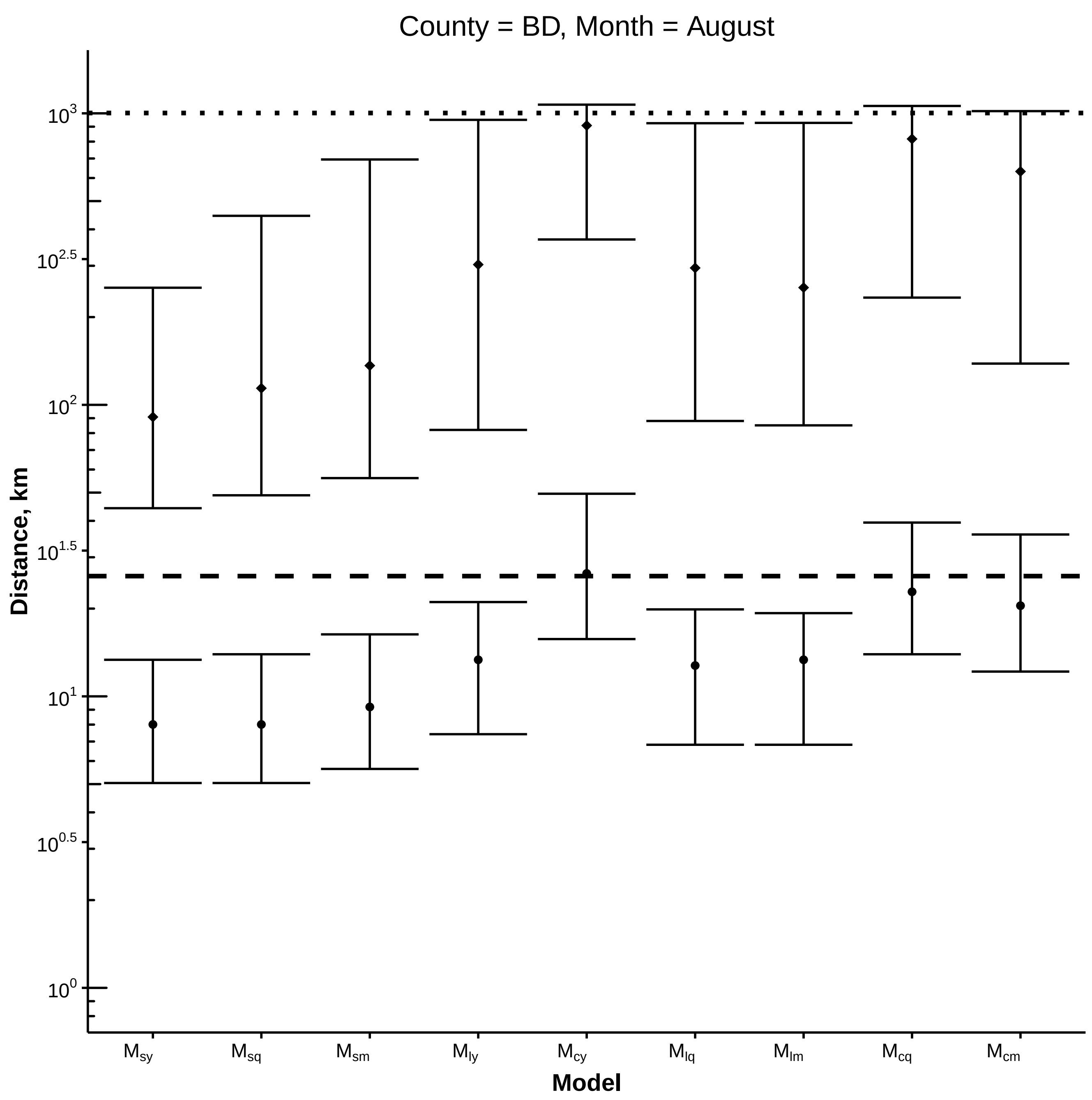
County = BD, Month = August
Distance, km
10^3
$10^{2.5}$
10^2
$10^{1.5}$
10^1
$10^{0.5}$
10^0
M_{sy}
M_{sq}
M_{sm}
M_{ly}
M_{cy}
M_{lq}
M_{lm}
M_{cq}
M_{cm}
Model

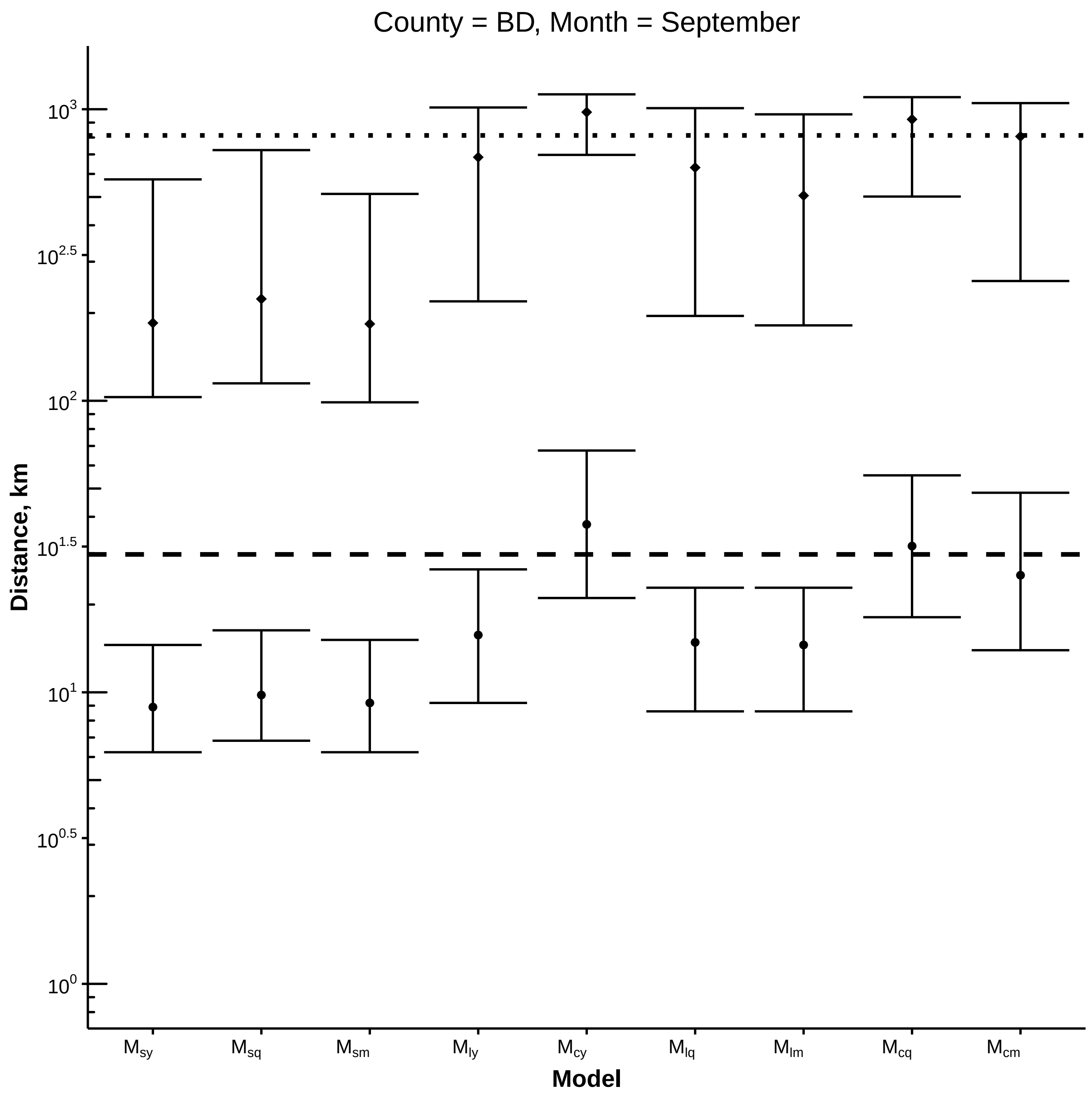

County = BD, Month = September
Distance, km
10^3
$10^{2.5}$
10^2
$10^{1.5}$
10^1
$10^{0.5}$
10^0
M_{sy}
M_{sq}
M_{sm}
M_{ly}
M_{cy}
M_{lq}
M_{lm}
M_{cq}
M_{cm}
Model

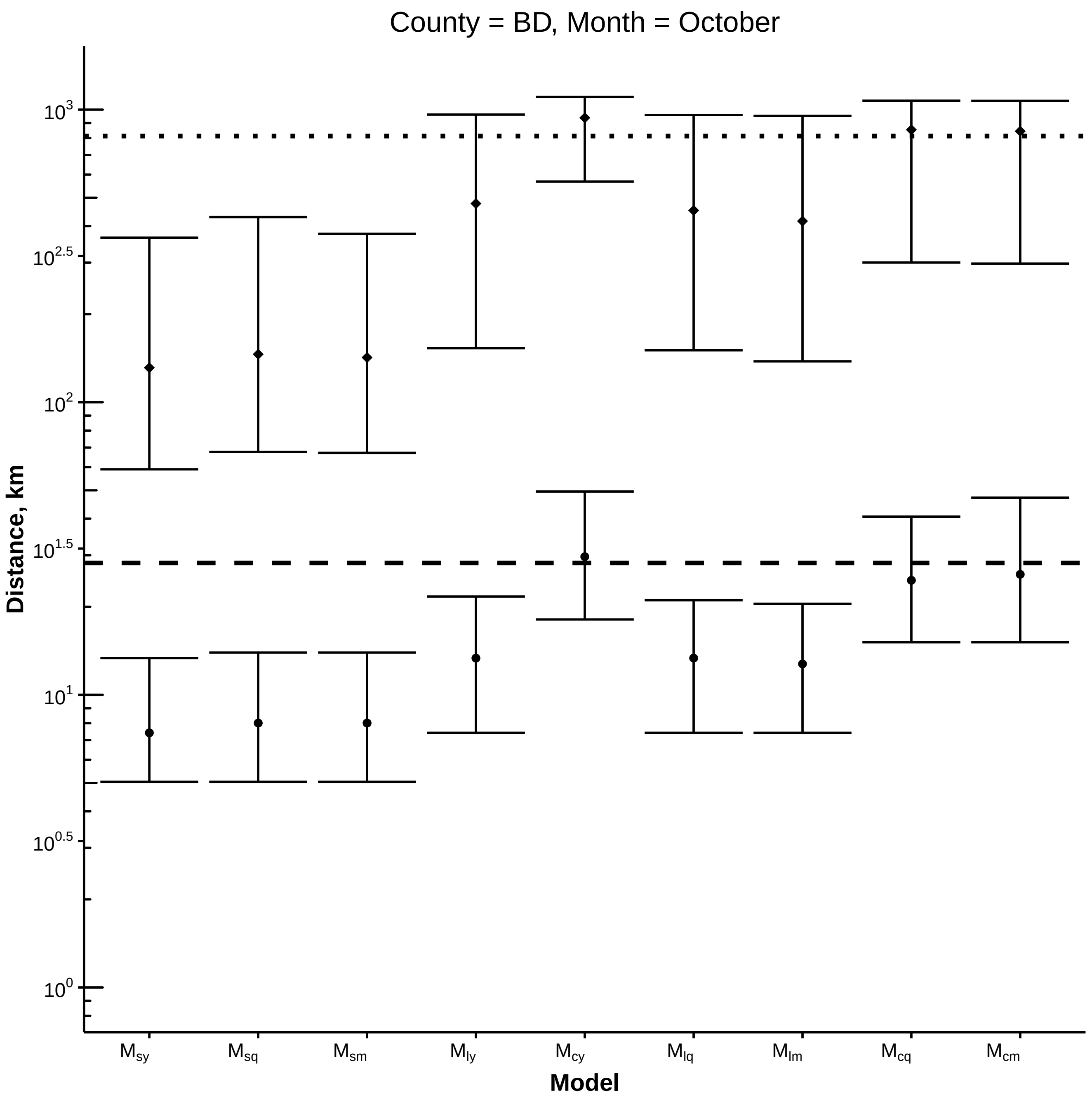
County = BD, Month = October
Distance, km
10^3
$10^{2.5}$
10^2
$10^{1.5}$
10^1
$10^{0.5}$
10^0
M_{sy}
M_{sq}
M_{sm}
M_{ly}
M_{cy}
M_{lq}
M_{lm}
M_{cq}
M_{cm}
Model

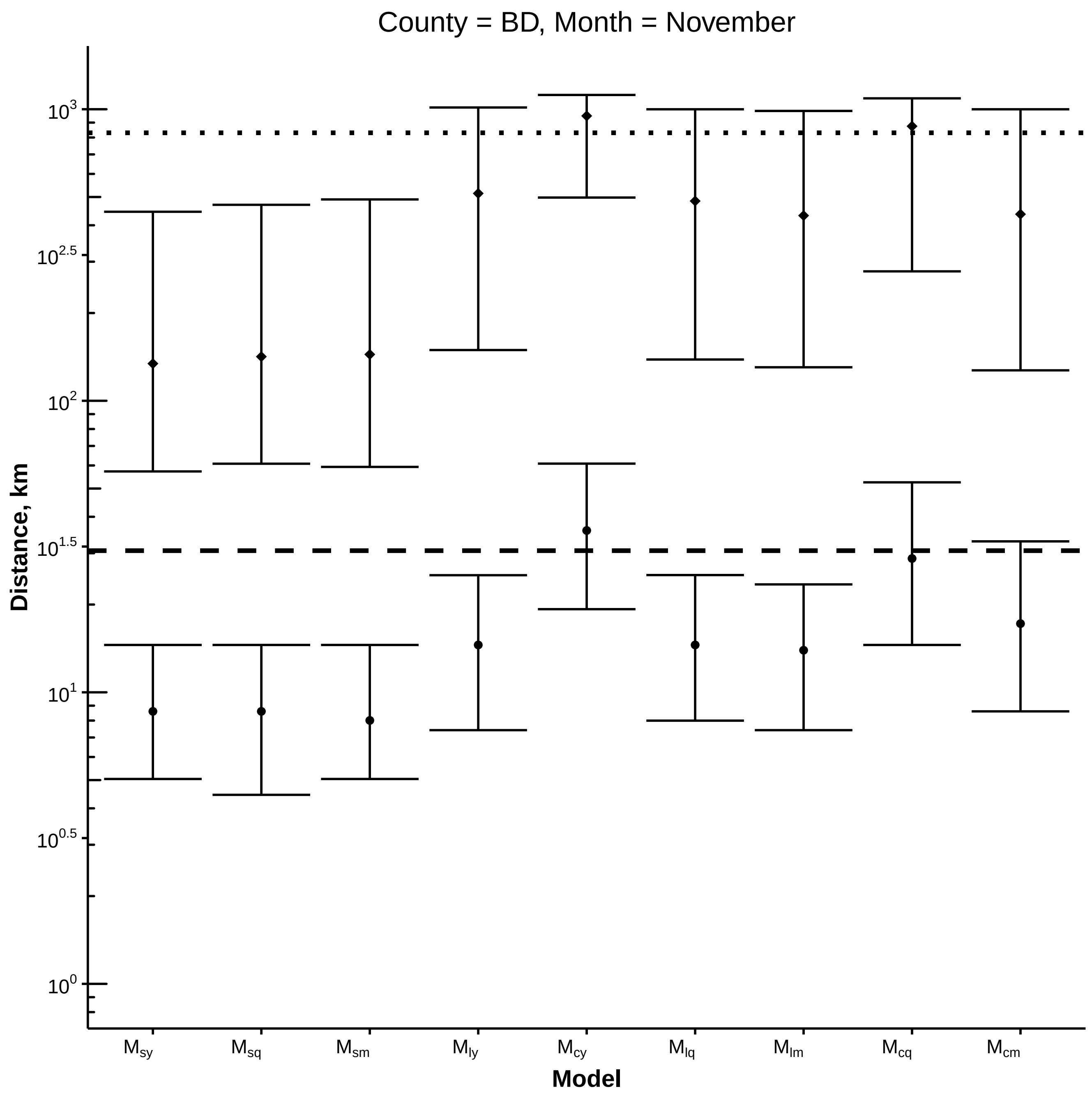
County = BD, Month = November
Distance, km
10^3
$10^{2.5}$
10^2
$10^{1.5}$
10^1
$10^{0.5}$
10^0
M_{sy}
M_{sq}
M_{sm}
M_{ly}
M_{cy}
M_{lq}
M_{lm}
M_{cq}
M_{cm}
Model

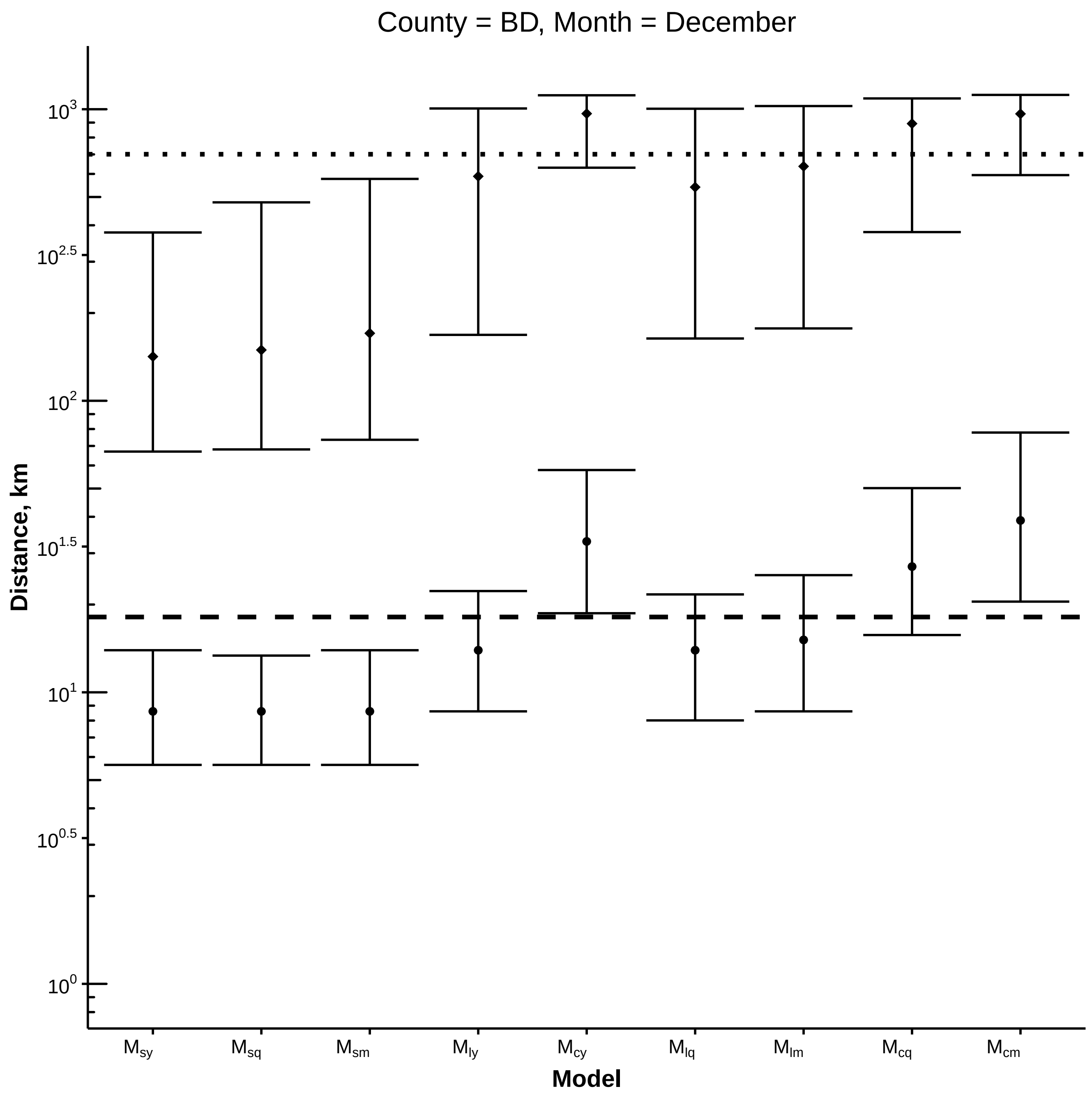

County = BD, Month = December
Distance, km
10^3
10^2.5
10^2
10^1.5
10^1
10^0.5
10^0
Msy
Msq
Msm
Mly
Mcy
Mlq
Mlm
Mcq
Mcm
Model
