# Supplementary figures and images for: Spatiotemporal Variation in Distance Dependent Animal Movement Contacts: One Size Doesn’t Fit All
Source: PLoS One. 2016 Oct 19;11(10):e0164008. doi: 10.1371/journal.pone.0164008 (PMC5070834; doi:10.1371/journal.pone.0164008)

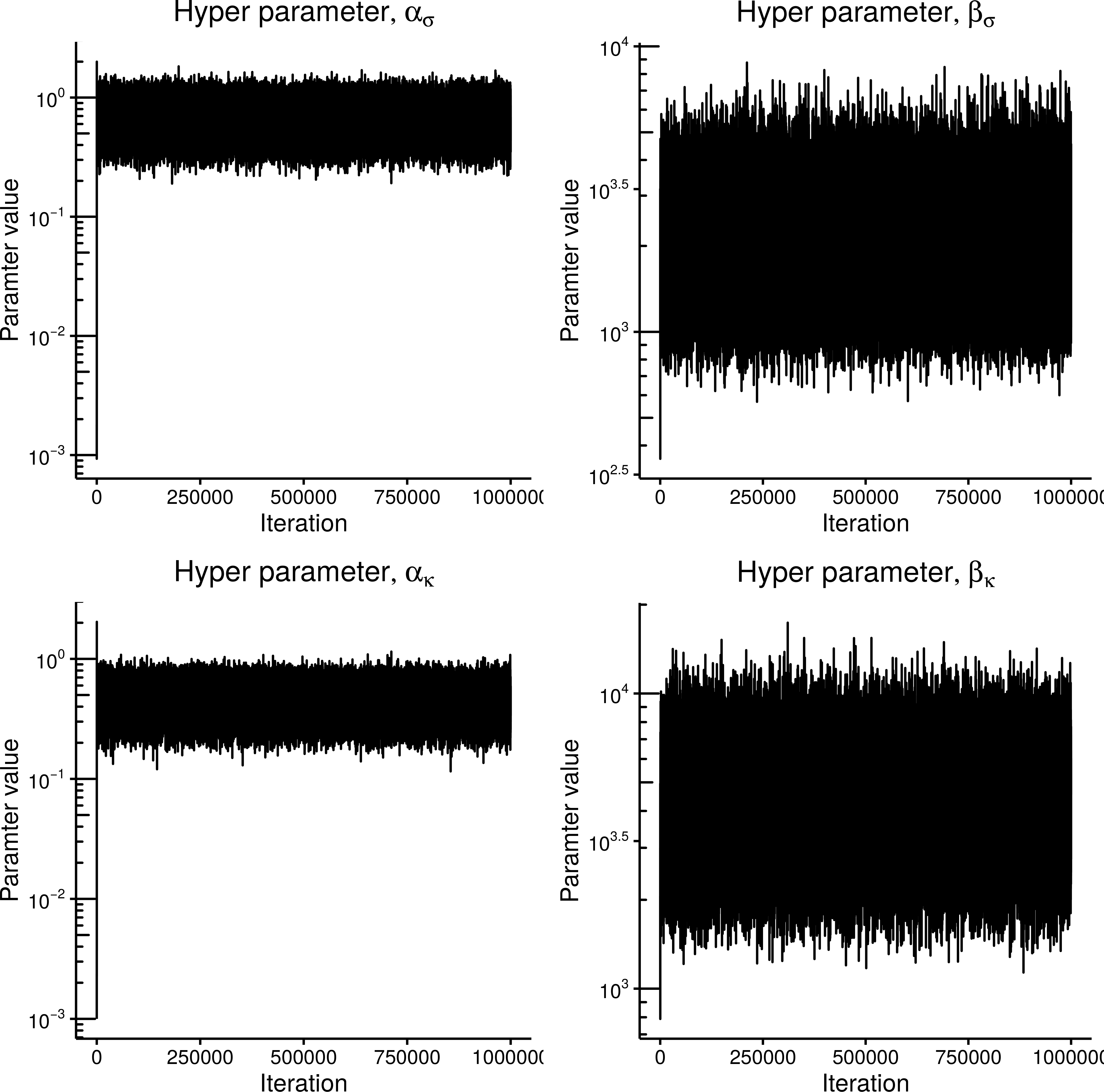

Supplement: S3 Fig — Top and bottom rows show, from left to right, hyper prior shape and scale parameters for σ and κ, respectively. (TIF) [file pone.0164008.s003.tif]
